# Targeting NUDT21-mediated alternative polyadenylation of oncogenes ameliorates colorectal cancer malignancy and metastasis

**DOI:** 10.1038/s41416-026-03451-9

**Published:** 2026-05-06

**Authors:** Shih-Chieh Lin, Ya-Chuan Tsai, Jui-Lin Wang, Hsian-Jean Chin, Ching-Chin Tsai, Shin-Chih Lin, Yi-Syuan Lin, Bo-Wen Lin, Shaw-Jenq Tsai

**Affiliations:** 1 Institute of Basic Medical Sciences, College of Medicine, National Cheng Kung University, Tainan, Taiwan, ROC; 2 Institute of Molecular Medicine, College of Medicine, National Cheng Kung University, Tainan, Taiwan, ROC; 3 Department of Physiology, College of Medicine, National Cheng Kung University, Tainan, Taiwan, ROC; 4 National Laboratory Animal Center, National Applied Research Laboratories, Tainan, Taiwan, ROC; 5 Department of Surgery, National Cheng Kung University Hospital, College of Medicine, National Cheng Kung University, Tainan, Taiwan, ROC; 6 Department of Biomedical Sciences, College of Science, National Chung Cheng University, Chiayi, Taiwan, ROC

**Keywords:** Colon cancer, Epithelial-mesenchymal transition

## Abstract

**Background:**

Colorectal cancer (CRC) is a disease leading cause of death worldwide. Lacking molecular markers for early detection and suitable candidates for targeted therapies are two main reasons for causing CRC malignancy.

**Objectives:**

Exploring the role of NUDT21 in colorectal cancer metastasis.

**Methods:**

A systematic bioinformatic analysis identified key genes involved in colorectal cancer, which were subsequently validated through loss-of-function and gain-of-function experiments conducted both in vitro and in vivo.

**Results:**

Overexpression of nucleoside diphosphate linked moiety X hydrolases-type motif 21 (NUDT21), a critical factor that regulates alternative polyadenylation, is observed in malignant polyps and in human and mouse CRC. Survival analysis reveals that high level of NUDT21 is associated with poor prognosis. NUDT21 knockdown not only inhibits cell growth but also reduces malignancy traits like anchorage-independent growth and cancer stemness. RNA-seq and RIP-seq results show NUDT21 preferentially binds to the proximal alternative polyadenylation site of numerous oncogenes to promote their expression and thus drive the progression of CRC. Treatment with re-purposing drugs targeting NUDT21 exhibit therapeutic potential in cell culture, organoid, orthotopic, and patient-derived xenografted CRC models.

**Conclusions:**

These findings demonstrate that NUDT21 is a critical regulator of colon cancer progression and a promising therapeutic target for CRC.

## Introduction

Colorectal cancer (CRC) is the fourth leading cause of death worldwide. It has been known that male sex and increasing age have strong associations with disease incidence of colon cancer. Moreover, genetic, lifestyle, obesity, and environmental factors also have some associations with colon cancer progression. However, the exact reasons for the increase of colon cancer prevalence are not completely understood [1]. Most of colon cancers arise from polyps. It begins with an aberrant crypt, evolves into a neoplastic precursor lesion, and progresses to colorectal cancer over an estimated 10 to 15 years period.

Despite the emergence of numerous screening programmes to reduce colon cancer incidence, nearly a quarter of patients are diagnosed at the advanced stages with metastases, which prevents the thorough removal of the cancerous lesion by surgery. For those patients with unresectable lesions, radiotherapy and chemotherapy are the leading strategies for the shrinkage of tumour size and suppression of further tumour spread [2]. However, chemotherapy is associated with certain limitations such as existing systemic toxicity, unsatisfying response rate, low tumour-specific selectivity, and chemoresistance. Therefore, investigating the regulatory mechanism that controls cancer progression is important for developing possible therapeutic agent and prevention strategy.

It is well known that RNA transcription is one of the central dogmas controlling gene expression. Precursor messenger RNA (pre-mRNA) can be processed to produce mature mRNA by post-transcriptional processes including addition of 5 prime capping to the beginning of the RNA, splicing of introns, and adding polyadenosines to the end of the RNA [3]. Briefly, 3′-untranslated regions (3’UTR) of transcribed pre-mRNA will be recognised and bound by complex of cleavage factors, cleaved, and added 150-250 adenine residues from the cleavage site to form the poly(A) tail. The function of poly(A) tail can increase stability of transcript and promote RNA transportation from nucleus to the cytosol [4]. Interestingly, several studies have shown that many mRNAs have different length of their 3′UTR from the same gene, and they are generated by choosing different signals of poly(A) sites (PAS). Furthermore, over 50% genes have more than one PAS signal in human genome. This particular RNA-processing mechanism is called alternative cleavage and polyadenylation (APA). It is a widespread and important mechanism to regulate gene expression at the post-transcriptional level. Alternative 3’UTR usage has been reported to regulate mRNA stability, translation, and localisation and involves in cell proliferation, differentiation and development of some diseases [5–7]. There are more than 70 proteins regulate 3’UTR APA events, including cleavage and polyadenylation specificity factor, cleavage stimulation factor, cleavage factor Im (CFIm) and CFIIm [8]. Among these complexes, CFIm complex plays an important regulatory role in PAS selection during APA.

CFIm family is composed of a smaller 25 kDa subunit (also called CPSF5 or NUDT21), a 59 kDa subunit (also called CPSF7), and a 68 kDa subunit (also known as CPSF6), which are highly conserved component in eukaryotes [7]. NUDT21 subunit interacts with CPSF6 and/or CPSF7 to form the heterotetrameric structure and binds to mRNA through its N-terminal RNA recognition motif (RRM). Each NUDT21 subunit can interact with mRNA by binding the UGUA element located around 50 nucleotides upstream of the cleavage site of pre-mRNA [9]. As an APA-associated protein, NUDT21 is involved in certain important biologic regulatory processes which can specifically interact with UGUA elements upstream of the PAS as a dimer and formed a loop, then prevented CPSF-mediated pre-mRNA cleavage [10]. Recently, there are several reports about NUDT21-mediated 3’UTR usage switch across various cancer types [11–20]. however, the findings have been controversial and inconsistent across different cancer types. Herein, we investigate the expression and function of NUDT21 in colorectal cancer and explore the therapeutic potential of targeting NUDT21 by re-purposing drugs.

## Methods

### Cell culture and drug treatment

Normal colon epithelial cells were cultured by Eagle’s Minimum Essential Medium with 10%FBS and colon cancer cell lines (Caco-2, HCT116, and COLO320DM) were maintained by RPMI1640 medium with 10% FBS. Antibiotics were also routinely added into the culture media (100 μg/ml streptomycin and 100U/ml penicillin G). All of cell lines used in this study were routinely tested for mycoplasma contamination by DNA staining and PCR. The identities of these cell lines were authenticated by STR analysis in the Center for Genomic Medicine, NCKU. Digoxin, digitoxigenin and ouabain (B2270, Selleckchem) were purchased to perform in vitro and in vivo experiments.

### siRNA, shRNA and sgRNA transfection

40 nM of siRNAs (control and NUDT21) purchased from Thermo Fisher Scientific and NUDT21 shRNA plasmid were transiently transfected into HCT116 cells by Lipofetamine^TM^ 2000 for two days. Next, HCT116 cells with NUDT21 shRNA or sgRNA (PX459 from addgene, #62988) targeting to proximal APA of YAP1 3’UTR region transfection was selected by puromycin (1 μg/ml) for 1 month to obtain stable knockout clones.

### RNA-Immunoprecipitation (RIP)

HCT116 cells were cross-linked by UV exposure (UV 2500 ×100 µJ/cm^2^) and cell pellets were lyzed RIPA (990 µl) and RNasin (10 µl). Equal amounts of primary antibodies (5 µg) were added to equal amount (500 µg) of samples and rotated overnight at 4 °C. After rotating, 50 µl of Dynabeads protein G suspension (Cat NO: 10004D, Invitrogen) was added to each sample. The mixtures were rotated again for 2 h at 4°C. The protein samples were placed on a magnetic base, washed the magnetic beads with 1 ml DEPC-PBS buffer for six times. After washing the magnetic beads, protein samples were divided into two groups for Western blotting (50 µl) and RNA extraction (450 µl).

### RNA isolation, RT-qPCR and 3’PCR

Total RNA was isolated by TRIzol^TM^ reagent according to the protocol provided by the manufacturer. In brief, 100 ng or 500 ng of total RNA was used to perform reverse transcription. Next, mRNA transcripts were quantified by Applied Biosystems StepOnePlus real-time PCR machine. 50 ng cDNA, 0.3 μM specific primer and 10 μl SYBR Green mix (Roche) were used in each reaction. RNA samples (10 µg) were fragmented by adding 1 µl of 10X fragmentation reagent (AM8740, Invitrogen) and incubating at 70 °C for 15 min. The reaction was stopped with 1 µl of 10X stop reagent (AM8740, Invitrogen), diluted to 400 μl with DEPC-treated water, and extracted using the acid-phenol-chloroform method. After overnight precipitation, the RNA was dissolved in 10.3 µl DEPC-treated water and subjected to reverse transcription using poly-dT primers for qRT-PCR [21]. Primer sequences were listed in the Supplementary Table S1.

### RNA-seq analysis

Total RNA (2 μg) isolated from HCT116 cells with or without NUDT21 knockdown by is siRNA (40 nM) for 48 h was sent to perform RNA-seq analysis by Illumina HiSeq instrument according to the manufacturer’s instructions (Illumina, San Diego, CA, USA). In addition, NUDT21 binding transcripts from RIP experiment were used for RNA-seq analysis by Nanopore sequencing platform (Oxford Nanopore Technologies, Oxford, UK). Original raw data and processed results had already submitted to the BioProject database (BioProject ID: PRJNA1137097).

### Western blot

Lowry assay was used to determine protein concentration. Next, 25 μg of protein was loaded into SDS-PAGE and gel was running for 1 h (140 volt). Then, proteins were transferred to PVDF membrane with Hoefer TE 70 semi-dry transfer unit at electronic current equal to 0.8 mA/cm^2^ of gel surface for 2 h. 5% non-fat milk/PBST was used to reduce nonspecific binding at room temperature for 1 h. The membrane was incubated in first antibody (NUDT21, YAP1, FLAG MYC and β-actin; diluent factor: 1 by 2000) at 4 °C overnight. After washing with PBST for three times and 5 min for each time, the membrane was incubated in secondary antibody (diluent factor: 1 by 5000) for 1 h. Finally, the membrane was washed with PBST for 12 times and 5 min for each time. Immunodetection was performed by using ECL. The detailed antibody information was listed in the Supplementary Table S2.

### MTS assay

CellTiter 96® AQueous One Solution Cell Proliferation Assay (MTS) (Promega, G3580) was used to detect cell proliferation or cell survival. Control and NUDT21 knockdown HCT116 cells or iNUDT21 Caco-2 cells were seeded into 96 well plate and incubate for 0, 1, 3, 5 days. MTS Assays were performed by directly adding 10 µl of MTS reagent to culture wells and incubating for 1 h to record the absorbance at 490 nm with ELISA reader.

### Cell migration assay

HCT116 cells (2.5 ×104) with either control or mutated YAP1 proximal APA sites or transduced with shCon or shNUDT21 with/without YAP1 restoration, were subjected to a 48-h transwell migration assay. Representative images were captured using a light microscope, and migratory areas from seven fields were quantified using ImageJ and expressed as the percentage of total area.

### Soft agar colony formation assay

The soft agar colony formation assay will be used to assay anchorage-independent growth (Au - Borowicz et al., 2014). 1 ml of 0.5% base agar (0.5% Agar, 1X Medium, 10% FBS, 1X Penicillin Streptomycin Solution) will plate into 6 well plate. The trypsinized single cells will mix in 0.35% top agar (0.35% Agar, 1X Medium, 10% FBS, 1X Penicillin Streptomycin Solution) and 1.5 ml of top agar will plate into 6 well plate. The soft agar will be incubated at 37°C in humidified incubator for 3 weeks and the medium will be changed every 2 days. The soft agar will be stained with crystal violet solution (0.5% crystal violate, 20% methanol in H_2_O) for 1 h.

### Immunohistochemistry staining

First, paraffin-embedded clinical and xenograft tumour samples used xylene to dewax and followed by immersing 100% ethanol for three times (5 min/time). Next, slides were sequentially immersed in different percentages of ethanol (95%, 80%, and 70%) and finally washed by double distilled water. Then, citrate buffer (pH 6.0) followed by a treatment with 3% H_2_O_2_ for 5 min were used to perform antigen retrieval for de-waxed samples. Next, tissue slides were hybridised with antibody (NUDT21, dilution 1:500, abcam# ab183660) in a humidity chamber at 4 °C for overnight. Colour was developed by an AEC substrate buffer (Bio SB, Inc., Santa Barbara, CA, USA) followed by hematoxylin staining (Bio SB, Inc., Santa Barbara, CA, USA) after finish of washing step for 1 h. Finally, samples were mounted with gelatin and results were scanned and quantified by TissueFaxs system in the core facility of NCKU. The detailed antibody information was listed in the Supplementary Table S2.

### Flow cytometry analysis

Cells were trypsinized by 0.25% trypsin and neutralised by FBS. After 600 xg centrifugation for 10 min, cell pellets were suspended and washed by using filtered Flow buffer (1x PBS containing 2 mM EDTA and 1% FBS). The cells were stained using PE-conjugated anti-CD133/2 (Clone 293C3, Miltenyi Biotec, Bergisch Gladbach, Germany) and APC-conjugated anti-CD44 (Miltenyi Biotec, Bergisch Gladbach, Germany) antibodies at 4°C in dark. After 20 min of incubation and subsequent washing, the cells were suspended in 400 µl Flow buffer. Cell suspension was immediately analysed by using flow cytometry BD FACSCanto II and FlowJo software (Becton-Dickinson, San Jose, CA, USA). The detail antibody information was listed in the Supplementary Table S2.

### Chromatin-Immunoprecipitation (ChIP)

Cells were exposed to 1% formaldehyde for 10 min with agitation, followed by the addition of ice-cold glycine to the culture medium (final concentration 125 mM) for 10 min to cease the cross-linking reaction. Subsequently, cells were harvested by scraping in 1 ml of 1X PBS and centrifuged for 5 min at 700 g. After discarding the supernatant, cells were lysed by adding 1% SDS lysis buffer (600 µl) and incubating on ice for 10 min. The cell lysates were then sonicated for 30 s with a 2-min pause between cycles, repeated nine times. Samples were centrifuged for 10 min at 16,000 *g*, and the supernatant was transferred. Samples were supplemented with 9X ChIP dilution buffer, and DNA concentration was measured using a Nanodrop system. Next, 200 µg of DNA for each sample, adjusted to a volume of 1500 µl with ChIP dilution buffer, was combined with an equal amount (5 µg) of primary antibody (YAP1 and control IgG) and rotated overnight at 4°C. After rotation, samples were mixed with 60 µl of Dynabeads protein A (Cat NO: 10002D, Invitrogen) and rotated for 2 h at 4°C. The samples bound to the magnetic protein A Dynabeads were collected and washed using a series of washing buffers, rotating for 5 min at 4°C on a magnetic base. Following washing, ChIP elution buffer (260 µl) was used to elute the sample twice with vortexing for 1 s. After rotating for 15 min, the supernatant was collected, and 5 M NaCl (20 µl) was added to reverse the cross-linking. Proteinase K buffer (32 µl) was then added to the sample, followed by treatment with phenol (pH 8.0, 550 µl) and centrifugation for 5 min at 16,000 g. The supernatant was combined with chloroform (500 µl) and centrifuged for another 5 min at 16,000 g. The resulting supernatant (450 µl) was collected and added to DNA precipitation buffer. After overnight incubation at -20°C to precipitate the DNA pellet, samples were centrifuged for 30 min at 16,000 g, the supernatant was removed, and the pellet was washed with 400 µl of 70% ethanol. Finally, the residual ethanol was removed, and the DNA pellet was dissolved in 35 µl of ddH^2^O. Next, YAP1 binding in different gene loci were determined by qPCR and primer sequences were listed in the Supplementary Table S1.

### Orthotopic injection mouse model of colorectal cancer

Luciferase gene was transfected into HCT116 cells with or without NUDT21 stable knockdown and hygromycin B (150 µg/ml) was used to select the stable clone for tracing tumour growth in vivo. Luciferase-labelled HCT116 NUDT21 stable knockdown and control cells (2×10^5^) or those with or without YAP1 restoration cells were suspended in 100 µl of 1X PBS and orthotopically injected into the wall of the male SCID mice caecum for 1 month. For azoxymethane (AOM)/dextran sodium sulfate (DSS)-induced mouse model of CRC, male B6 mice (6–8 weeks old) in the experimental group were treated with AOM working solution (10 mg/ kg body weight) while control mice were received sterile isotonic saline injection at day one. Then, DSS (MP Biomedicals, Santa Ana, CA, USA)-containing water (2%) was given at week 1, 4 and 7 to mice in the AOM group. Mice were sacrificed at 1, 2, 4, 8, 12 and 20 weeks after AOM injection. For PDX model of CRC, national laboratory animal centre in Taiwan helps us to set up this animal model. Briefly, fresh clinical specimens of CRC were washed by antibiotics for several times and then cut into several small fragments for subcutaneous inoculation in the back of immunodeficiency mice. PDX Mouse colorectal tissues were fixed by 3.7% paraformaldehyde and embedded in a paraffin wax block for IHC staining. A formal statistical sample size estimation was not performed for this animal study. Male mice were randomly assigned to different treatment groups in a blinded manner. Mice were housed in the barrier facilities on a 12-h light-dark cycle with food and water available *ad libitum*. All procedures were performed in accordance with the guidelines for the handling of laboratory animals of the Laboratory Animal Center, College of Medicine, National Cheng Kung University. The IVIS system was used to monitor tumour growth weekly. Briefly, luciferin (150 mg/kg body weight) was intraperitoneally injected into SCID mice and incubated for 10 min before imaging. Approval for all the animal studies was obtained from the Institutional Animal Care and Use Committee (IACUC: 107224) at the Laboratory Animal Center, NCKU.

### Patient-derived xenograft (PDX) mouse model of colon cancer

The PDX animal studies were performed following the guide for the animal use protocol approved by the Institutional Animal Care and Use Committee (IACUC number: NLAC(TN)-109-D-001 and NLAC(TN)-110-D-004). All mice were kept in a Specific pathogen-free (SPF) animal facility with a 12 h light/dark cycle and were grouped and housed in individually ventilated cages with autoclaved aspen chip bedding (TAPVEI®, 5 × 5 × 1 mm) and one puck of nesting material. The temperature was set to keep within 21 °C and 23 °C and the relative humidity were kept between 45–65%. Laboratory Autoclavable Rodent Diet 5010 (LabDiet® 5010) and autoclaved tap water were provided to the animals ad libitum. A formal statistical sample size estimation was not performed for this animal study. Male mice were randomly assigned to different treatment groups in a blinded manner. Female 6–10-week-old ASID (NOD.Cg-*Prkdc*^scid^*Il2rg*^tm1Wj*l*^/YckNarl) mice were provided by the National Laboratory Animal Center (NLAC), National Applied Research Laboratories (NARLabs), Taiwan for PDX model establishment. Fresh tumours were cut into small fragments of approximately 3 × 3 × 3 in mm and were subcutaneously grafted into the dorsal flank of female ASID mice. The length (*L*) and width (*W*) of tumours were measured with digital caliper twice a week. The tumour volume (TV) was calculated using the formula: TV = 0.5 × *L* × *W*^2^ and the relative tumour volume was calculated as the formula: RTV = TVt/TVf, where TVt is the TV on day t and TVf is the TV on the first dosing day. Once the tumours reached a size of 100 mm³ (124.2 ± 100.3 mm^3^), the mice were treated with either vehicle, ouabain (2.5 mg/kg), or digoxin (2.5 mg/kg) twice a week for 1 month. Tumour volume was represented as fold over control.

### Human normal and colorectal cancer organoids culture

Normal and cancerous colon tissues obtained from patients were cut into 5 mm pieces and washed eight times with ice-cold PBS containing antibiotics (Simply-CC501-0100). Normal colon tissue and cancerous tissue were separately digested with 100 U/ml collagenase XI, 0.125 mg/ml dispase II, and 0.035 mg/ml liberase (Roche- 5401119001) in HBSS. After 40 min of digestion at 37 °C, the reactions were halted by adding FBS and passing the cells through a 70 μm cell strainer. The strained cells were centrifuged and resuspended in Matrigel (Corning-354230) at a concentration of 2 × 10^5^ cells/ml. The resuspended Matrigel (10 μl) was seeded in 96-well plates and cultured in colon organoid medium (1 mM N-Acetylcysteine, 1X Glutamine, 1 nM Gastrin I, 50 ng/ml recombinant human EGF, 1 μg/ml recombinant human R-Spondin 1, 100 ng/ml recombinant human Noggin, 2 μM A83-01, 10 μM SB-202190, 60 ng/ml recombinant human Wnt-3a, 1X antibiotics, 1X N-2, 1X B-27, 10 mM HEPES, 0.1% BSA, 10 mM Nicotinamide, 10 μM Y-27632 and 2.5 μM CHIR-99021) for 1 week. The cultured organoids were then treated with 200 nM Digoxin or Ouabain and assessed using MTS Assay and microscopy analysis.

### Bioinformatics analysis

Gene expression profiles in the mouse and human CRC datasets were individually analysed from Gene Expression Omnibus (GEO) (GSE12049, GSE37182, GSE44861, GSE44076 and GSE21815) database via GEO2R platform. The first step was to assign the control and experimental groups individually, then click the analysis icon to download the complete results table. Significantly upregulated genes from these datasets (*p* < 0.001, fold change ≥2) were used to perform a Venn diagram analysis, which identified 100 genes shared across the five datasets. These 100 genes were then further analysed to determine key survival-related genes specific to colorectal cancer cell lines by downloading and examining dependency scores from DepMap (https://depmap.org/portal/). Correlation analysis between NUDT21 and YAP1 in clinical specimens of colorectal cancer (E-MTAB-990) was analysed by R2 analysis platform (https://hgserver1.amc.nl/cgi-bin/r2/main.cgi). Correlation analyses of YAP1, NUDT21/YAP1 downstream target genes and metastatic parameters were analysed in clinical colorectal cancer dataset (TCGA-COAD) dataset by using UCSC Xena platform (https://xena.ucsc.edu/) and results were presented as heat map by Gitools. Downregulation gene list (*p* < 0.05, Fold change ≥ 2) of NUDT21 knockdown in HCT116 cells was analysed by Connectivity Map tool (https://clue.io/about) to identify potential drug targeted to NUDT21. All the public datasets used in this study were listed in the Supplementary Table S3. The original raw data, along with the processed results of our RNA-seq analysis, have been submitted to the BioProject database under accession number PRJNA1137097.

### Statistical analysis

The data were expressed as means ± standard error of the mean and were analysed by either Student’s *t* test (for two groups) or one-way analysis of variance (for three or more groups) using GraphPad Prism 9.0 (GraphPad Software, Inc. La Jolla, CA, USA). Post-test analysis was performed using Tukey’s multiple comparison. A *p* value less than 0.05 was considered statistical significance.

## Results

### Overexpression of NUDT21 is a novel and crucial regulator for colon cancer

**Fig. 1:**
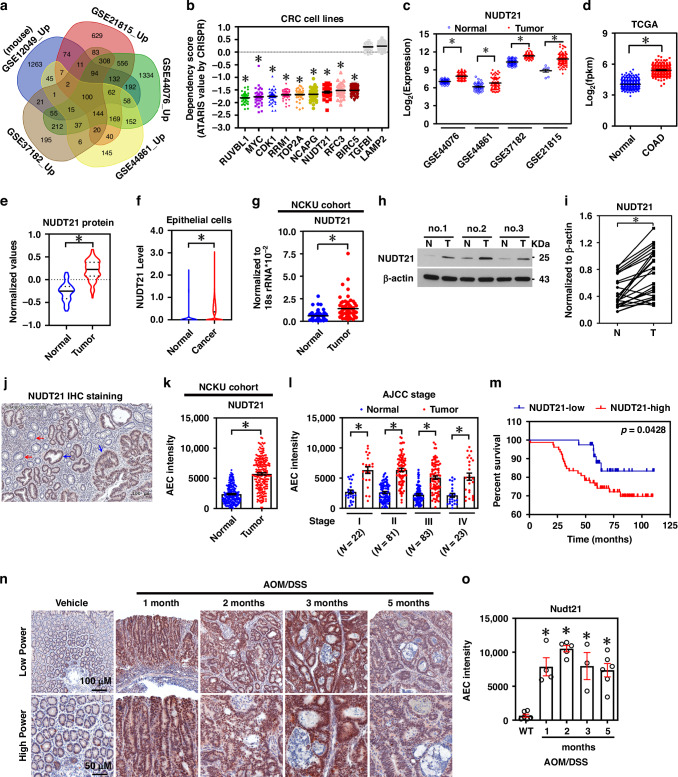
Overexpression of NUDT21 was a novel regulator involved in the pathogenesis of colorectal cancer. **a** Upregulation gene lists with significant changes from AOM/DSS-treated mouse model of CRC (GSE124029) and four different human datasets containing clinical specimens of CRC (GSE37182, GSE44861, GSE44076 and GSE21815) were used to cross-reference by using tool of Venn diagram. **b** Genes with dependency scores lower than cut-off value (−1.5) in all the colorectal cancer cell lines (*n* = 38) were shown and performed statistical analyses by comparing the results of LAMP2 gene. Both TGFB1 and LAMP2 were genes from the one hundred gene list with no obvious changes of dependency score after knockout by CRISPR/Cas9 system. NUDT21 expression levels were shown from four different human datasets containing clinical specimens of CRC (GSE37182, GSE44861, GSE44076 and GSE21815) (**c**) and TCGA-COAD dataset (normal, *n* = 345; COAD, *n* = 290) (**d**). **e**, **f** NUDT21 RNA and protein were determined by qRT-PCR (*n* = 63 pairs) (**g**) and Western blot (*n* = 21 pairs) (**h**), respectively. NUDT21 protein expression was quantified and normalised to β-actin level in CRC clinical specimens (**i**). Representative IHC image shows normal (red arrow) and cancerous (blue arrow) glands stained with anti-NUDT21 antibody (**j**). IHC staining results were scanned and quantified by the TissueFaxs system (*n* = 211 pairs) (**k**). Expression levels of NUDT21 in paired samples of CRC were presented according to the AJCC stage of CRC (**l**). **m** Survival curve analysis was performed by using NUDT21 expression according to the clinical parameter of CRC patients. Mice received a single intraperitoneal injection of AOM (10 mg/kg body weight) followed by three cycles of DSS (2%) supplemented water and then sacrificed at long-term time points (1, 2, 3 and 5 months) after AOM injection. Representative images show paraffin-embedded colon tissue sections derived from AOM/DSS-induced mouse colon cancer stained with anti-Nudt21 antibody for long-term time periods (**n**). IHC staining results from long-term time points (vehicle, *n* = 6; 1 month, *n* = 4; 2 months, *n* = 5; 3 months, *n* = 3; 5 months, *n* = 6) were scanned and quantified by the TissueFaxs system (**o**).

To identify a pivotal and novel regulator in CRC, datasets from the GEO database, including a CRC mouse model induced by AOM/DSS and four distinct human datasets comprising clinical specimens of normal and cancer tissues, were downloaded and analysed. One hundred genes were found to be consistently overexpressed across these datasets (Fig. 1a). Next, we utilised data deposited in the Project Achilles database to further validate the significance of these 100 genes in CRC development. After stringent analyses, only nine genes were found to be indispensable for the survival of all CRC cell lines, with a cutoff value of ≤ -1.5 (Fig. 1b). Among these, NUDT21 caught our attention due to its unique function in regulating RNA 3'UTR usage switch. Subsequent analysis revealed that the level of NUDT21 is elevated in colon cancer tissues across four distinct human CRC datasets (Fig. 1c) and in genetic and carcinogenic mouse models of colorectal cancer (Supplementary Fig. S1A and B). Similar results were observed in a large cohort of clinical specimens from The Cancer Genome Atlas Colon Adenocarcinoma (TCGA-COAD), LinkedOmics, meta-analysis data (Fig. 1d, e; Supplementary Fig. S1C), and other cancer types (Supplementary Fig. S1D). The bioinformatics findings were confirmed in CRC samples collected in our hospital (Fig. 1g–j). Quantitative analysis of IHC staining results (211 pairs of normal and cancerous tissues) demonstrated increased NUDT21 expression in CRC tissues across different cancer stages (Fig. 1k, l), with higher NUDT21 levels correlating with poorer survival outcomes for CRC patients (Fig. 1m). Moreover, induction of Nudt21 was confirmed in our in-house AOM/DSS-induced mouse model of colon cancer via IHC staining (Fig. 1n, o). Taken together, our findings suggest that NUDT21 may serve as a novel oncogenic regulator implicated in the pathogenesis of CRC.

### NUDT21 overexpression is an early-onset process in colorectal polyp and correlated with the malignancy of polyp subtype

**Fig. 2:**
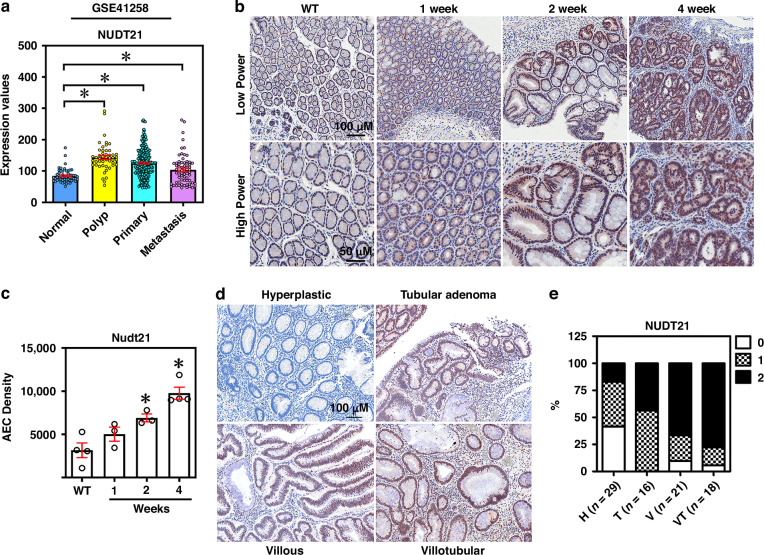
Overexpression of NUDT21 occurred in the early stage of colorectal polyp and correlated with potential of cancerous polyp subtypes. **a** NUD21 expression level was analysed in a public dataset containing human colorectal polyp (*n* = 49), primary cancer (*n* = 253), metastatic cancer (*n* = 67) and normal colorectal tissues (*n* = 54) (GSE41258). Mice received a single intraperitoneal injection of AOM (10 mg/kg body weight) followed by three cycles of DSS (2%) supplemented water and then sacrificed at 1, 2, and 4 weeks after AOM injection. Representative images show paraffin-embedded colon tissue sections derived from AOM/DSS-induced mouse colon cancer stained with anti-Nudt21 antibody (**b**). IHC staining results (WT, *n* = 4 mice; 1week, *n* = 3 mice; 2 weeks, *n* = 3 mice; 4 weeks, *n* = 4 mice) were scanned and quantified by the TissueFaxs system (**c**). Representative IHC images show immunoreactivity of NUDT21 in tissues of hyperplastic polyp (benign), tubular, villous and villotubular polyp, respectively (**d**). IHC staining results were presented as a percentage of staining intensity (**e**). 0: no signal; 1: weak signal; 2: strong signal. H: hyperplastic; T: tubular adenoma; V: villous; VT: villotubular.

During the examination of NUDT21 expression across various clinical specimens within the CRC dataset, we observed a significant elevation in NUDT21 levels in colorectal polyps compared to normal colon tissues (Fig. 2a), indicating the pivotal role of NUDT21 in the hyperplasia of colon epithelium at the early stage. To validate this hypothesis, colorectal tissues from AOM/DSS-treated mice over different time courses were analysed for NUDT21 expression. Intriguingly, NUDT21 expression began to rise after the transition of single-layer epithelium to multiple layers at two weeks after AOM/DSS treatment (Fig. 2b, c) and maintained elevated then after (Fig. 2b, c). Moreover, similar patterns were noted in colon tissues from APC^min/+^ transgenic mice, showing obvious increases after 3 months of birth, which further intensified at 6 and 9 months (Supplementary Fig. S2). Previously, it has been known that colorectal polyps are categorised into hyperplastic and neoplastic polyps, with only the latter posing a risk of developing colorectal cancer [22, 23]. To investigate the association between NUDT21 and polyp malignancy, its expression was assessed across various types of colorectal polyps. Our results clearly revealed elevations in NUDT21 expression levels in tubular, villous, and villotubular polyps, which carry a risk of malignancy, while its expression was barely detectable in hyperplastic polyps with low malignant potential (Fig. 2d, e). Collectively, our findings suggest that NUDT21 may play a critical role in the early stages of colorectal polyp development and correlate with its malignancy potential.

### Loss of NUDT21 attenuated the growth and malignancy of colon cancer

**Fig. 3:**
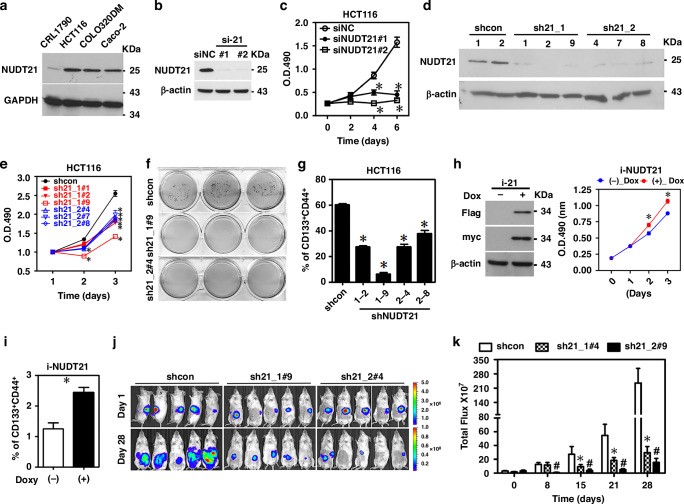
Loss of NUDT21 attenuated the growth and malignancy of colorectal cancer. **a** NUDT21 expression in normal colon epithelial (CRL1790) and different colon cancer cell lines was determined by Western blot. HCT116 was used to perform transient knockdown by NUDT21 siRNA (40 nM) for 48 h and NUDT21 expression was analysed by Western blot (**b**). Cell proliferation in HCT116 cells treated with or without NUDT21 siRNA was determined by MTS assay (*n* = 3) (**c**). HCT116 was used to set up stable knockdown clones (The numbers indicated the different clones) by NUDT21 shRNA and its expression was measured by Western blot (**d**). Cell proliferation was analysed in HCT116 with or without NUDT21 shRNA treatment by MTS assay (*n* = 3) (**e**). **f** Soft agar assay was performed by using NUDT21 stable knockdown clones of HCT116 cells and control. Representative pictures of colonies were shown after incubation for 2 weeks. **g** Cancer stemness population was determined by staining with fluorescence-labelled antibodies (anti-CD133 and anti-CD44) for 1 h and subjected to flowcytometry analysis in NUDT21 stable knockdown clones of HCT116 cells. Cell proliferation and cancer stemness were measured by using MTS assay (**h**, left panel) and flowcytometry (**i**) in Caco-2 cells with inducible-NUDT21-FLAG-MYC (i-NUDT21) expression system (*n* = 3). FLAG and myc antibodies were used to measure induction of exogenous NUDT21 by doxycycline (1 μg/ml) for 24 h (**h**, left panel). NUDT21 stable knockdown clones of HCT116 (sh21_1#9 and sh21_2#4) and control (shcon) cells were used to set up orthotopic injection mouse model of CRC for 1 month. Tumour growth was weekly monitored by IVIS imaging system (**j**) and quantified by measuring the intensity of luciferase (**k**).

To investigate the role of NUDT21 in colon cancer cells, expression levels of NUDT21 in different cell lines were determined by Western blot. Results showed that NUDT21 expression was increased in several colon cancer cell lines compared to CRL1790, a normal colon epithelial cell line (Fig. 3a). Therefore, HCT116 cells were used to perform NUDT21 knockdown by siRNA and set up stable knockdown clones by shRNA. Cell proliferative abilities were obviously decreased in both NUDT21 knockdown and stable knockdown clones (Fig. 3b-e). Furthermore, HCT116 cells with stable knockdown of NUDT21 not only reduced colony numbers in the soft agar assay (Fig. 3f) but also decreased cancer stemness population (Fig. 3g). In contrast, induction of NUDT21 by doxycycline significantly promoted cell growth and increased cancer stemness population in colorectal cancer cells (Fig. 3h, i). Next, orthotopic injection of HCT116 cells with or without NUDT21 stable knockdown showed that loss of NUDT21 markedly inhibited tumour growth (Fig. 3j, k). Taken together, these results indicate that NUDT21 overexpression might promote colon cancer progression and its malignancy.

### NUDT21 promotes colorectal cancer metastasis by enhancing epithelial-mesenchymal transition process

**Fig. 4:**
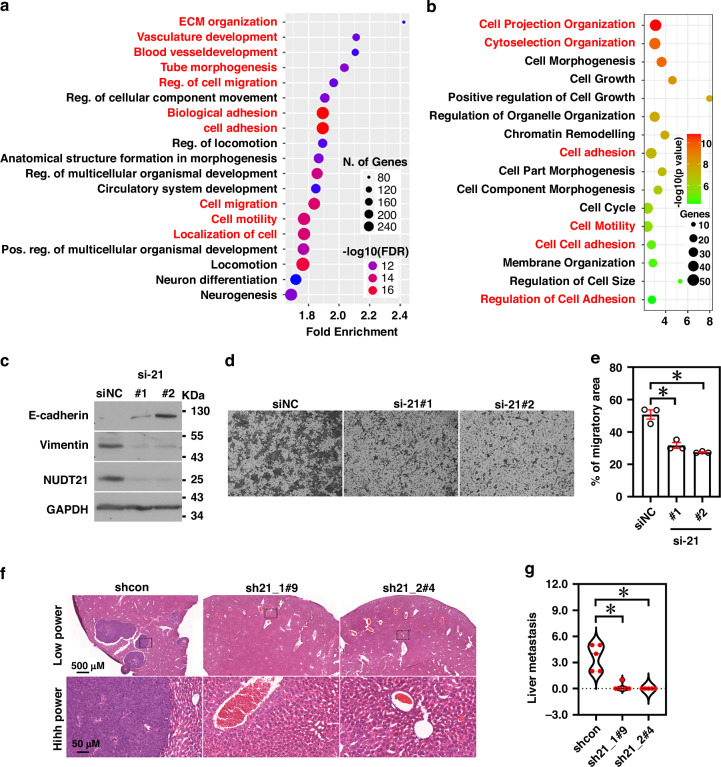
Loss of NUDT21 attenuated cancer metastasis by impairing EMT process in colorectal cancer. Downregulation gene list from NUDT21 knockdown in HCT116 cells (**a**) and NUDT21-RIP data (**b**) were individually analysed for biological processes of gene ontology by using ShinyGO 0.77 tool (http://bioinformatics.sdstate.edu/go/). **c** E-cadherin (epithelial marker) and Vimentin (mesenchymal marker) were measured in HCT116 with or without NUDT21 knockdown (40 nM for 48 h). HCT116 cells were knocked down NUDT21 by its siRNA (40 nM) for 48 h and then trypsinized cells were performed migration assay for another 24 h. Representative migratory pictures were shown (**d**) and quantified by Image J (**e**). Results were shown as percentage of migratory area (*n* = 3). Asterisks indicate significant difference at *p* < 0.05 by One-way ANOVA analysis. Metastatic nodules were analysed in the mouse liver section of CRC orthotopic mouse model by hematoxylin and eosin stain (**f**) and their numbers were counted in the livers of control and NUDT21 knockdown groups (**g**).

To further dissect the cellular function of NUDT21 in colon cancer cells, RNA-seq and RNA-immunoprecipitation (RIP)-seq experiments were conducted on HCT116 cells with or without NUDT21 knockdown, as well as on CRL1790 and HCT116 cells by using a NUDT21 antibody, respectively. Subsequently, the downregulated gene list resulting from NUDT21 knockdown and the gene list of transcripts specifically bound by NUDT21 in HCT116 cells were subjected to gene ontology analysis to elucidate their respective biological processes. Intriguingly, both analyses revealed consistent findings indicating that NUDT21 regulates cancer metastasis-related processes such as angiogenesis, cell adhesion, cell migration, and cell motility (Fig. 4a, b). Indeed, NUDT21 knockdown not only impaired the epithelial-to-mesenchymal transition (EMT) process (Fig. 4c) but also attenuated the migrating ability of HCT116 cells (Fig. 4d, e). More crucially, orthotopic mouse model of colon cancer revealed the number of liver metastatic lesions was significantly reduced in NUDT21 knockdown groups (Fig. 4f, g). Taken together, our findings indicate that NUDT21 is likely a critical factor promoting cancer metastasis in colon cancer.

### Loss of NUDT21 attenuated the growth and malignancy of colon cancer

**Fig. 5:**
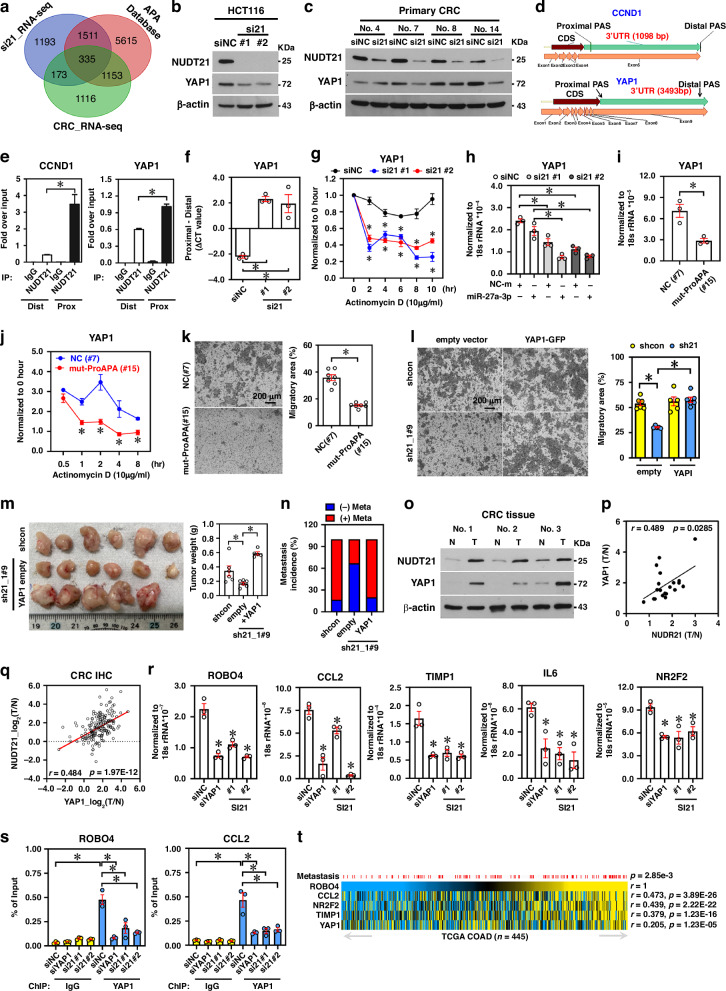
Loss of NUDT21 attenuated the growth and malignancy of colorectal cancer via YAP1 downregulation. **a** Venn diagram shows the relationships of differentially expressed genes in NUDT21 knockdown, colon cancer cells, and genes with multiple 3’PAS. Representative Western blot shows the levels of NUDT21, YAP1, and β-actin (as a loading control) in NUDT21-knocked down HCT116 (**b**) and primary colon cancer cells (**c**) as indicated. **d** Schematic illustration of CCND1 and YAP1 3’UTR with proximal and distal PAS as indicated. **e** NUDT21 and control IgG antibodies were individually performed RNA-immunoprecipitation (RIP) in HCT116 cells. Binding profiles of NUDT21 to proximal or distal PAS of CCND1 and YAP1 transcripts were measured by qRT-PCR (*n *= 3). **f** NUDT21 was knocked down by its two different siRNAs (40 nM) for 48 h. Short and long 3’UTR isoforms of YAP1 were specifically measured by 3’PCR methodology (*n* = 3). **g** YAP1 RNA stability was measured by qRT-PCR in HCT116 cells with NUDT21 knockdown (40 nM) for 48 h and then treated with actinomycin D (10 μg/ml) for indicated time points (*n* = 3). **h** HCT116 cells were treated with NUDT21 siRNA (40 nM) and miR-27a-3p (50 nM) for 48 h and YAP1 expression was measured by q-RT-PCR (*n* = 3). Proximal APA site of YAP1 3’UTR region was specifically mutated by CRISPR/Cas9 technique. Negative control (NC#7) and positive (mut-ProAPA#15) stable clones were used for measurements of YAP1 expression (**i**) and its RNA stability (**j**) by qRT-PCR (*n* = 3). Cell migration assays were performed for 48 h, and the representative images with quantified results are shown (**k**). Control (shcon) and shNUDT21 (sh21_1#9) HCT116cells were stably restored YAP1 expression and then performed cell migration assay for 48 h (**l**) and orthotopic injection mouse model of CRC for 1 month. Xenograft tumour image (**m**, left panel), tumour weight (**m**, right panel) and liver metastasis incidence (**n**) were shown. NUDT21 and YAP1 expression levels in paired colon cancer specimens were determined by Western blot. Representative pictures were shown (**o**). Fold change (tumour/normal) results of NUDT21 and YAP1 were performed Pearson’s correlation analysis (*n* = 21) (**p**). YAP1 expression was determined by using IHC staining. The expression levels of YAP1 and NUDT21 in CRC clinical specimens were used to perform correlation analysis (*n* = 188) (**q**). **r** HCT116 cells were individually knocked down NUDT21 or YAP1 by its siRNA (40 nM) for 48 h and then gene expression levels of ROBO4, CCL2, TIMP1, IL-6 and NR2F2 were determined by qRT-PCR (*n* = 3). **s** HCT116 cells were respectively performed ChIP-PCR by using YAP1 and control (IgG) antibodies after NUDT21 or YAP1 knockdown by its siRNA (40 nM) for 48 h. The bindings of YAP1 on ROBO4 and CCL2 loci were measured by q-PCR (*n* = 3). **t** The Pearson’s correlation analyses were performed by using expression levels of ROBO4, CCL2, IL-6, NR2F2, YAP1 and metastatic parameter in the dataset of TCGA COAD clinical colorectal cancer specimens (*n* = 445). Results were further presented as heat map.

Since NUDT21 is known to function as an APA mediator, our objective was to identify potential downstream targets of NUDT21 with clinical relevance. To achieve this, we cross-referenced the downregulated gene list from NUDT21-knocked down HCT116 cells (Supplementary Table S4), the upregulated gene list from CRC specimens (Supplementary Table S5), and a gene list featuring multiple polyadenylation sites from an APA database (Supplementary Table S6) (Fig. 5a). A total of 335 genes from the overlapping region of these three datasets were identified and subjected to expression correlation analyses in a large cohort of CRC samples. Numerous genes were shown to be positively correlated with NUDT21 (Supplementary Table S7). Considering the crucial roles of YAP1 in colon cancer progression [24, 25]. and the unexplored aspect of its 3’UTR usage regulation, we selected YAP1 to investigate its potential as a direct target of NUDT21 in colon cancer cells. Indeed, YAP1 and its downstream target genes exhibited a positive correlation with NUDT21 expression in CRC samples (Supplementary Fig. S3A). First, YAP1 expression decreased after NUDT21 knockdown (Fig. 5b) and increased in colorectal cancer cells with NUDT21 overexpression (Supplementary Fig. S3B). Similar results were observed in primary colon cancer cells isolated from clinical specimens of CRC patients (Fig. 5c). According to the APA database, the YAP1 transcript contains proximal and distal PAS within its 3’UTR region (Fig. 5d). RNA immunoprecipitation (RIP) using an NUDT21 antibody (Fig. 5e and Supplementary Fig. S3C) demonstrated its preferential binding to the proximal PAS site of the YAP1 transcript compared to the distal PAS site (Fig. 5e, right panel). The well-known downstream target of NUDT21, CCND1, was used as a positive control (Fig. 5e, left panel). Indeed, NUDT21 knockdown induced a shift in YAP1 3’UTR usage from the short to the long isoform (Fig. 5f and Supplementary Fig. S3D) and reduced YAP1 RNA stability in colorectal cancer cells (Fig. 5g), whereas NUDT21 overexpression increased YAP1 RNA stability (Supplementary Fig. S3E). To explore whether the longer YAP1 3’UTR is susceptible to miRNA-mediated repression, potential YAP1-targeting miRNAs were analysed using starBase. Among them, miR-27a was selected because its binding site is present only in the long 3’UTR isoform and it is overexpressed in colorectal cancer specimens (Supplementary Fig. S3F, G). Importantly, miR-27a overexpression did not alter YAP1 expression under basal conditions but significantly reduced YAP1 levels following NUDT21 knockdown (Fig. 5h), suggesting that NUDT21-driven shortening of the YAP1 3’UTR allows YAP1 to evade miR-27a-mediated suppression during cancer progression. To directly examine the function of the proximal APA site of YAP1, CRISPR/Cas9-mediated mutagenesis was performed (Supplementary Fig. S3H) in colorectal cancer cells. Mutation of this site reduced YAP1 expression and RNA stability and impaired colorectal cancer cell migration (Fig. 5i-k). Consistently, a YAP1 expression construct carrying the short 3’UTR isoform produced higher YAP1 protein levels and greater migratory ability than the construct with the long 3’UTR (Supplementary Fig. S3I, J), highlighting distinct functional roles of YAP1 transcripts differing in 3’UTR length. Since our current results revealed that NUDT21 promotes colorectal cancer metastasis in vitro and in vivo (Fig. 4d–g), we next examined whether YAP1 contributes to these effects. YAP1 was stably restored in one of NUDT21-knockdown clone (Supplementary Fig. S3K), and its overexpression not only rescued cell migration in vitro (Fig. 5l) but also enhanced tumour growth and liver metastasis in vivo (Fig. 5m, n). Supportively, our clinical relevance findings revealed that YAP1 expression was elevated in CRC tissues compared to their normal counterparts (Fig. 5o and Supplementary Fig. S3L-N) and it had positive correlation with NUDT21 levels in CRC specimens (Fig. 5p). Consistently, similar results were obtained using IHC staining to measure YAP1 expression and its relationship with NUDT21 in our CRC tissue array and CRC public dataset (Fig. 5q and Supplementary Fig. S3O). To explore the underlying mechanism of NUDT21-accelerated cancer metastasis in colorectal cancer, downstream targets of NUDT21 related to cancer metastasis processes from the downregulated gene list after NUDT21 knockdown in HCT116 cells were cross-referenced with YAP1-ChIP seq data from the ChIP-Atlas database. Among them, ROBO4, CCL2, TIMP1, IL-6, and NR2F2 were selected for further verification. Indeed, knockdown of both NUDT21 and YAP1 individually decreased their expression levels in HCT116 cells (Fig. 5r). Mechanistically, YAP1-ChIP-PCR results indicated that YAP1 bound to the promoter regions of gene loci, and both YAP1 and NUDT21 knockdown attenuated YAP1 binding ability at the gene loci of ROBO4 and CCL2 (Fig. 5s and Supplementary Fig. S3P). Importantly, ROBO4 expression levels were positively correlated not only with the expression levels of CCL2, NR2F2, IL-6, TIMP1, and YAP1 but also with metastatic events in clinical specimens of colorectal cancer (Fig. 5t). Overall, our findings suggest that NUDT21-promoted cancer metastasis might be mediated via the crucial function of YAP1 during colon cancer progression.

### Drugs targeted to NUDT21 show the therapeutic potentials in different models of colorectal cancer

**Fig. 6:**
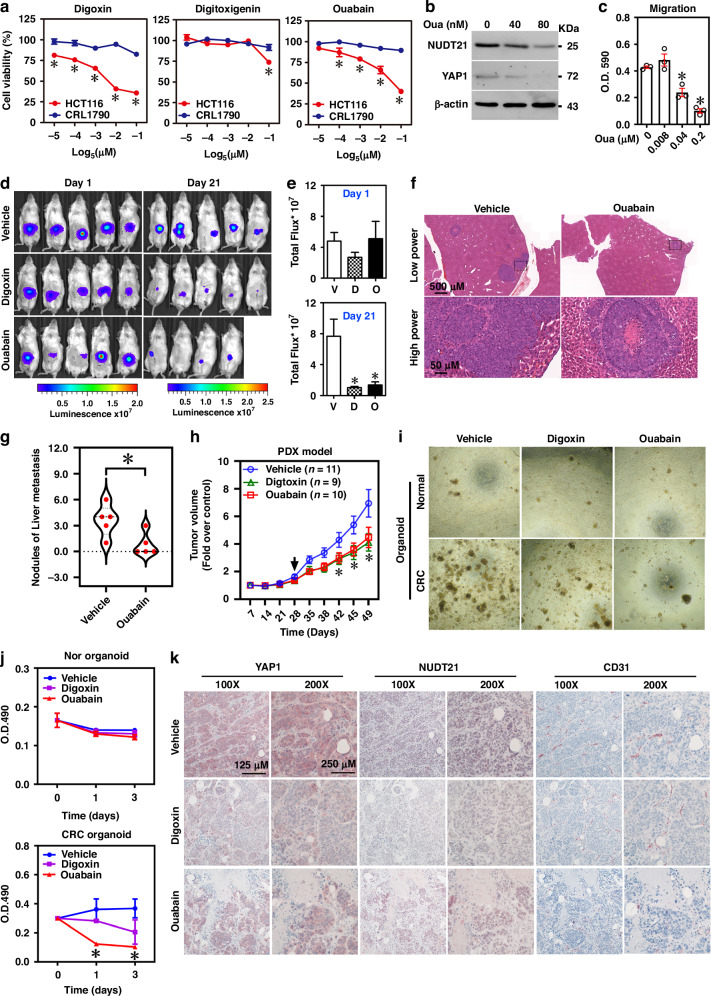
Drugs targeted to NUDT21 showed therapeutic potential in vitro and in vivo models of CRC. **a** Three compounds, including digoxin, digitoxigenin and ouabain, identified by bioinformatics analysis and cross-referenced with Connectivity Maps data, were used to treat normal colon cell line (CRL1790) and colon cancer cell line (HCT116) with different concentrations for 24 h and viability of cells was determined by MTS assay. Asterisks indicate significant difference at p < 0.05 by Student’s *t* test compared to CRL1790 at the same concentration of compounds, respectively. **b** Representative Western blot image shows level of NUDT21 and YAP1 were decreased by treatment with ouabain for 24 h. **c** HCT116 cells were treated with different doses of ouabain and then performed cell migration assay for 24 h. Migratory cells were measured by trypan blue staining and then quantified by measuring O.D. 590 nm (*n* = 3). HCT116 cells were used to perform orthotopic injection mouse model of CRC and both digoxin and ouabain treatments were intraperitoneally given twice a week for 2 weeks. Tumour growth was monitored (**d**) and quantified by IVIS imaging system (**e**). Metastatic nodules in the liver of CRC orthoepic mouse model were analysed by H&E staining (**f**) and their numbers were also counted in the control and ouabain treatment groups (**g**). CRC specimens from clinical patients were used to set up PDX mouse model of CRC and organoid models. Digoxin and ouabain treatments were individually and intraperitoneally given twice a week for 3 weeks when tumour size was researched to 100 mm^3^ and tumour size was measured twice a week (**h**). Both digoxin and ouabain were directly treated with organoid for 3 days (**i**) and measure their cell viability by MTS assay (**j**). **k** Expression levels of NUDT21, YAP1 and CD31 in PDX tumours were measured by IHC staining method and representative results were shown. CD31 was used as an angiogenic index.

We then uploaded the gene signature from RNA-seq results of NUDT21-knocked down HCT116 cells to the Connectivity Map to identify drug candidates to inhibit NUDT21’s pathological functions. Among them, three potential compounds were identified from the top 20 ranking list (Supplementary Table S8). To assess the therapeutic potential of these compounds for colon cancer, we treated HCT116 (colon cancer cells) and CRL1790 (normal colorectal epithelial cells) with these compounds individually and measured cell survival using an MTS assay. Interestingly, two of the three compounds (digoxin and ouabain) significantly decreased cell survival of HCT116 cells in a dose-dependent manner, while they had no effect on CRL1790 cell survival (Fig. 6a), suggesting these drugs may specifically target colorectal cancer cells. Furthermore, treatment with digoxin and ouabain reduced both NUDT21 and YAP1 expression levels (Fig. 6b and Supplementary Fig. S4A, B) and cell migratory ability (Fig. 6c) in HCT116 cells. Next, to evaluate the clinical potential of these drugs, we used two different mouse models of colorectal cancer to verify their treatment potential in vivo. First, HCT116 cells were orthotopically injected into the mouse caecum for 1 week and then individually received digoxin or ouabain treatments for another 2 weeks. Results showed that both digoxin and ouabain treatments significantly attenuated tumour growth in the orthotopic injection mouse model of CRC (Fig. 6d, e). Additionally, ouabain treatment further reduced metastatic events in the orthotopic injection mouse model of CRC (Fig. 6f, g). Then, patient-derived xenograft (PDX) mouse models of CRC, by subcutaneously inoculating tumour tissue, and organoid models from CRC clinical specimens, were used to test the therapeutic potential of digoxin and ouabain. Results showed that both digoxin and ouabain treatments inhibited tumour growth in the PDX mouse models of CRC (Fig. 6h) and CRC organoid models (Fig. 6i, j, lower panel), while they had no effect on normal colorectal organoids (Fig. 6i, j, upper panel). Additionally, the levels of NUDT21, YAP1, and CD31 (a marker of angiogenesis) were reduced in the PDX mouse model of CRC after digoxin and ouabain treatments (Fig. 6k). Taken together, these findings suggest that digoxin and ouabain, targeting NUDT21, may hold therapeutic potential for CRC patients in the future.

## Discussion

During the past several decades, numerous efforts had been tried to develop effective regimens to treat human cancers. However, the success rate in the clinical development of effective oncologic therapeutic drugs has been impressively poor. The most important reason for the poor performance of cancer drugs is the heterogeneous natures of cancer cells. The molecular characteristics of cancer cells are often dissimilar given that they are histologically identical. One way to overcome this hurdle is identifying a master regulator that regulates numerous genes based on a giving biological process. Recent advances in molecular biology and translational medicine indicate that APA is a critical mechanism to alter gene expression profile under the same genetic composition, which changes the biochemical nature of cells even without any detectable mutation. Herein, we report that NUDT21, an APA regulator, acts as a key player in the development and progression of CRC via regulating APA.

The levels of NUDT21 in different cancers are varied among studies as some papers reported upregulated but some show downregulated. To address these contradictory results, we employed an unbiased analysis of datasets deposited in GEO and TCGA and found that NUDT21 is overexpressed in most cancer cell types. Based on these meta-analysis data (*n* = 4036), it is clear that NUDT21 is overexpressed in most CRC samples. Moreover, we demonstrated NUDT21 overexpression in paired human CRC specimens (*n* = 211 pairs). Our data are more reliable as subject variation due to different genetic and environmental backgrounds was eliminated since tissues were taken from the same individuals. Our data show NUDT21 is not only highly expressed in clinical CRC specimens but also in pre-malignant colorectal polyps. In contrast, the normal colon tissues and benign polyp express very low levels of NUDT21. It is known that colon polyps are classified into two groups: the non-neoplastic polyps and the neoplastic polyps. The non-neoplastic polyps include hyperplastic, juvenile polyps, and inflammatory pseudopolyps, while the neoplastic polyps include villous, tubular and villotubular polyps [26]. In general, hyperplastic polyps have very low malignant potential while neoplastic polyps are more likely to develop into CRC along with time [26]. Our data show that NUDT21 is elevated in the neoplastic polyps, suggesting that NUDT21 may play an important role in causing malignant transformation of colon polyp to colorectal cancer. It also indicates that NUDT21 can be used as a biomarker to predict the malignancy potential of polyps.

The function of NUDT21 in cancer is also controversial. Some suggest that NUDT21 has tumor-suppressing function [11, 16, 17]. while the others indicate it is an oncogene [12, 14, 18, 20]. Loss of NUDT21 has been shown to increase tumorigenic properties in glioblastoma [11], cervical cancer [17], hepatocellular carcinoma [13], bladder cancer [16] and kidney renal clear cell carcinoma [19]. Conversely, it was also found that NUDT21 is overexpressed in glioblastoma [12], leukaemia [14, 18], and pancreatic cancer [20]. Knockdown of NUDT21 decreased cell proliferation and increased apoptosis in these cancer cells. The cause of such a discrepancy is not known and warrants further investigation. Our current data are in consistent with those showing NUDT21 exerts the oncogenic functions as we demonstrated that knockdown of NUDT21 inhibits cell proliferation, migration, stemness, and metastasis in vitro and in vivo.

To dissect the underlying mechanisms responsible for overexpression of NUDT21-mediated cancer malignancy, we performed RNA-seq and RIP-seq to investigate how NUDT21 regulates its potential downstream target genes. Results show that genes involved in cell adhesion, migration, extracellular matrix organisation, and blood vessel development are primarily bound and upregulated by NUDT21. Furthermore, our results showed that NUDT21 preferentially binds to the proximal APA site. To investigate the binding of NUDT21 to the proximal APA site of target gene indeed regulates target gene expression and contributes to CRC malignancy, we chose YAP1 as an example. YAP1 has been known to be overexpressed in CRC [27–29]. and plays crucial roles in CRC malignancy [24, 25, 30–33]. Our findings have revealed that NUDT21-mediated shorting of YAP1 3’UTR contributes to its evasion of miR-27a targeting in colorectal cancer. Furthermore, direct mutation of YAP1 proximal APA site reduces its expression and cell migratory ability. More importantly, we demonstrated that by controlling YAP1’s expression, genes involved in cell proliferation, adhesion, migration, blood vessel formation, and extracellular matrix organisation are all regulated by NUDT21 to promote CRC tumour growth and metastasis. These data provide strong evidence to support that NUDT21 is a key regulator in promoting CRC malignancy via a unique mechanism of minimising mRNA transcripts being degraded by destabilising factors such as miRNAs.

**Fig. 7:**
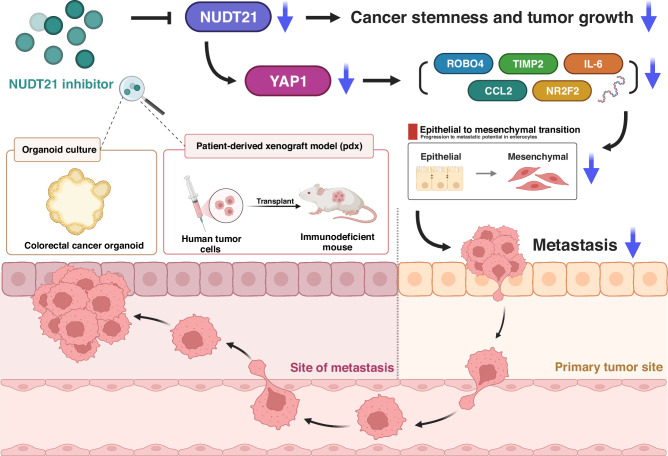
A cartoon that briefly summarises the involvement of NUDT21 in facilitating both tumour growth and the development of metastasis in colorectal cancer. The figure was created using BioRender.com.

Improving the treatment efficacy is an unmet medical need as drug resistance is the primary factor contributing to the failure of chemotherapies. As described above, identifying a modulator that regulates the key hub of biological processes is a way to overcome the hurdle of treatment failure. Since NUDT21 behaves like a master regulator in CRC progression, we then focused on targeting NUDT21 to revert the progression of CRC. Two FDA-approved drugs including digoxin and ouabain were identified to be effective in inhibiting NUDT21 expression in cancer cells and inducing cell death. Originally, digoxin and ouabain are cardiac glycosides, a class of naturally derived compounds best known for their effects on the Na⁺/K⁺-ATPase pump and for their long-standing clinical use in heart disease. Recently, several studies have demonstrated their cancer therapy potential in different types of cancer by elevating cytotoxicity, increasing the capacity of macrophages to kill cancer cells and repressing epithelial-mesenchymal transition (EMT) and circulating tumour cell (CTC) cluster to block cancer metastasis [34–37]. Consistently, our results revealed their specific cytotoxicity for CRC cells and their therapeutic potential in different CRC platforms. However, the underlying mechanism of NUDT21 repression by digoxin and ouabain is still not clearly explored yet. Previously, both digoxin and ouabain have shown to induce autophagy and lysosomal function [38, 39]. Ouabain can facilitate target protein degradation through the autophagosome-autolysosome pathway [38]. Moreover, ouabain also promotes ubiquitin-mediated proteasomal protein degradation through reactive oxygen species (ROS) generation [40]. In addition, digoxin has been shown to repress NRF2, an oxidative stress-responsive transcription factor, at the transcriptional level by inhibiting PI3K/Akt pathway in pancreatic cancer cells [41]. These findings indicate the future direction that digoxin- and ouabain-mediated repression of NUDT21 may occur through protein-level regulation via autophagy and ubiquitin–proteasome degradation, or through transcriptional regulation by NRF2. Taken together, NUDT21 has been revealed its crucial role for the progression of CRC, its association with malignant colon polyp transformation and its therapeutic potential for CRC patient (Fig. 7). Hopefully, our findings might provide the alternative therapy for CRC treatment in the future.

## Supplementary information

Supplementary figures

Supplementary Table S1, S2, S6 and S7

Supplementary Table S3

Supplementary Table S4

Supplementary Table S5

Supplementary Table S6

## Data Availability

All data supporting the findings of this study are available from the corresponding author upon reasonable request.
