# Supplementary material for: Targeting NUDT21-mediated alternative polyadenylation of oncogenes ameliorates colorectal cancer malignancy and metastasis
Source: Br J Cancer. 2026 May 6;135(4):518–31. doi: 10.1038/s41416-026-03451-9 (PMC13427831; doi:10.1038/s41416-026-03451-9)
Supplement: Supplementary file 1 — Supplementary figures [file 41416_2026_3451_MOESM1_ESM.docx]

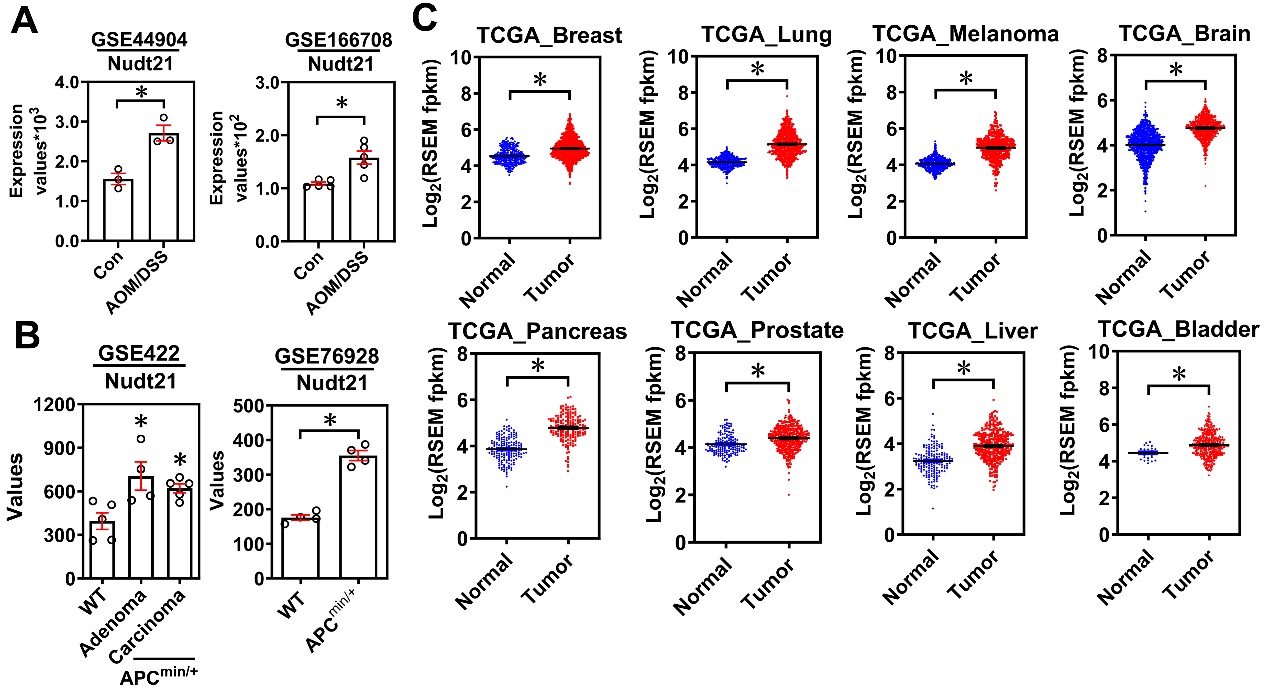


**Supplementary figure 1: The expression levels of NUDT21 were overexpressed in different mouse models of colorectal cancer and different types of cancer. (A, B)** The expression level of Nudt21 was individually analyzed in the GEO datasets containing AOM/DSS treatment-derived **(A)** and APCmin/+-derived colorectal cancer **(B)**. **(C)** The expression level of NUDT21 was analyzed in different cancer types by using TCGA datasets. Asterisks indicate significant difference at p < 0.05 by using un-paired t-test analysis.

**
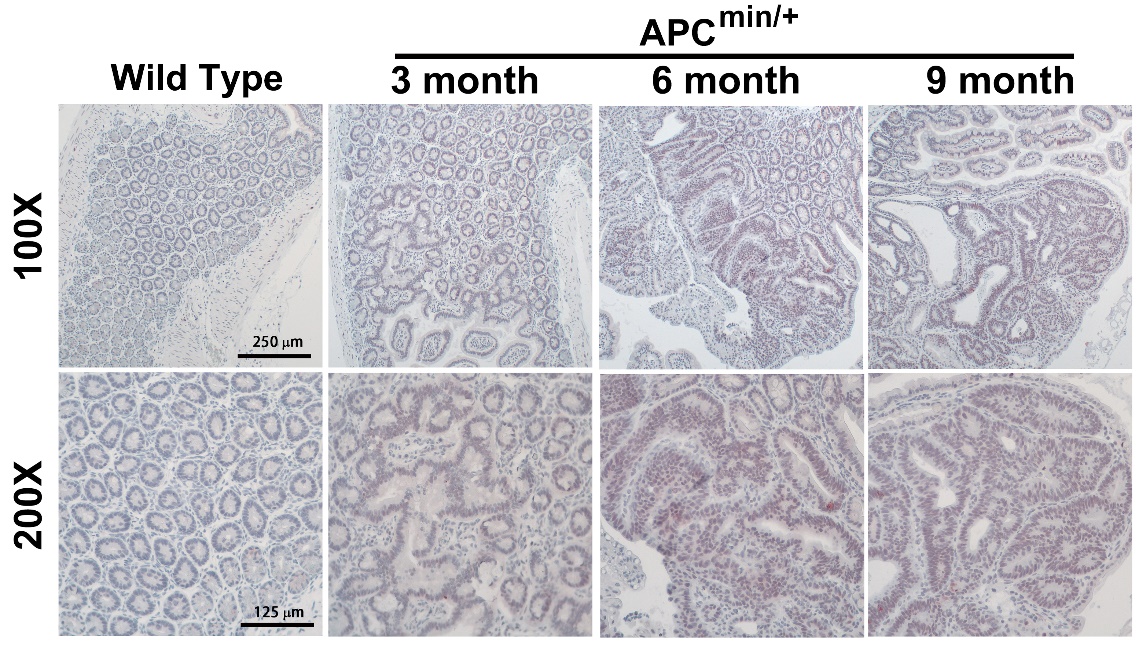
Supplementary figure 2: NUDT21 expression was elevated in the APC^min/+^ mouse model of colorectal cancer.** APC^min/+^ mice were sacrificed at different time points and then mouse colon tissues were collected to perform IHC staining by using NUDT21 antibody. Representative pictures were shown.

**
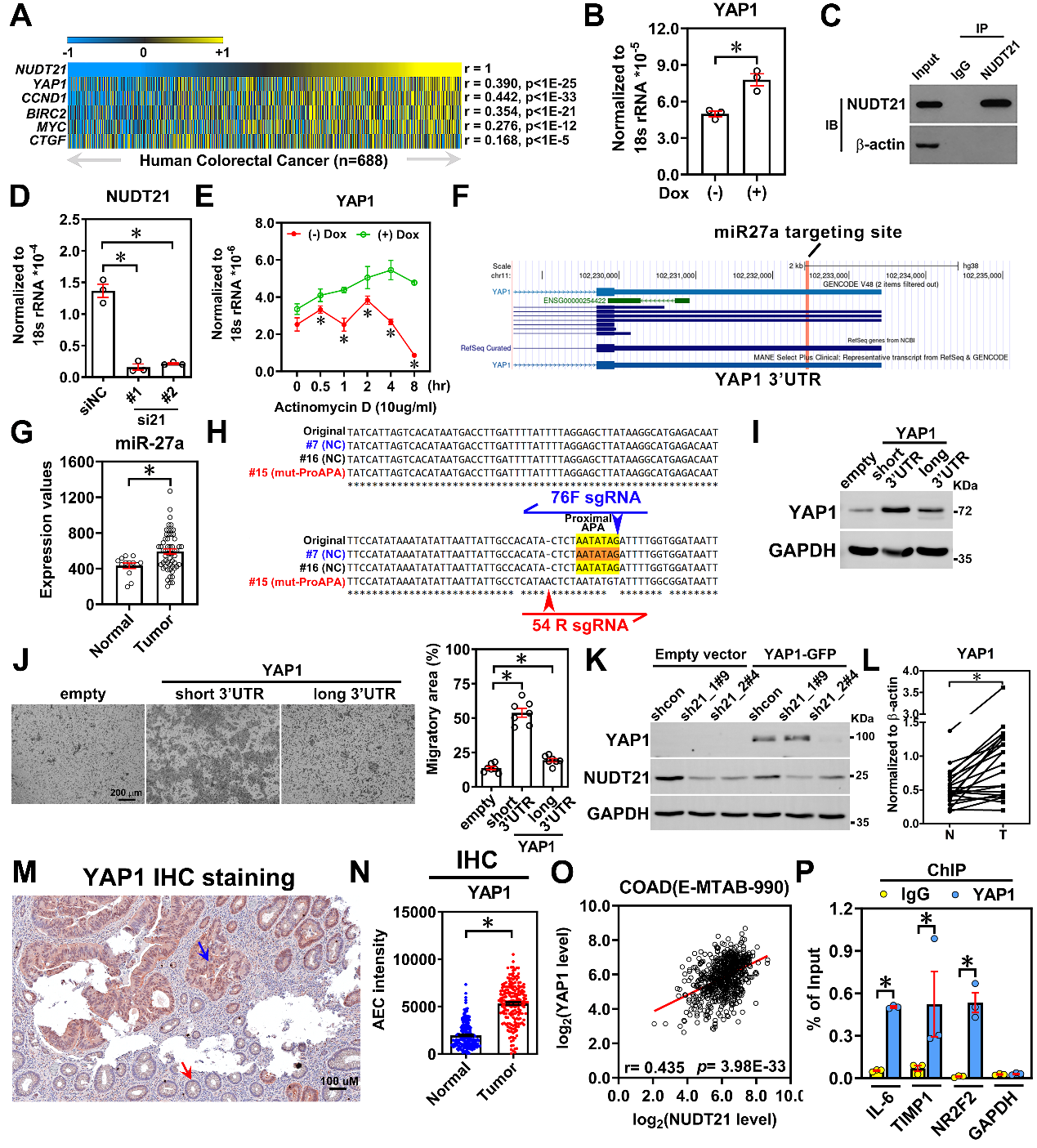
**

**Supplementary figure 3: NUDT21-mediated shortening of YAP1 3’UTR region increased its RNA stability by evading miRNA targeting and contributed to its overexpression in clinical specimens of CRC.**

**(A)** The Pearson’s correlation analyses were performed by using expression levels of NUDT21, YAP1 and YAP1 downstream target genes in the dataset from clinical colorectal cancer specimens (E-MTAB-990). Results were further presented as heat map. **(B)** YAP1 expression was measured in iNUDT21 Caco-2 cells received doxycycline (1 μg/ml) for 48 hours by qRT-PCR (n=3). **(C)** Western blot image shows the specificity of NUDT21 pull down by its specific antibody from HCT116 cells. **(D)** NUDT21 expression was measured in HCT116 received NUDT21 siRNA (40 nM) for 48hours by qRT-PCR (n=3). **(E)** YAP1 expression was detected in iNUDT21 Caco-2 cells received with doxycycline (1 μg/ml) for 48 hours and then treated with actinomycin D (10 μg/ml) for indicated time points by qRT-PCR (n=3). **(F)** miR-27a-3p targeting to YAP1 3’UTR region was analyzed AGO-CLIP data from starBase tool (https://rnasysu.com/encori/). (G) miR-27a expression levels were analyzed from a public GEO dataset (GSE41655) containing CRC (n=56) and normal colon (n=13) tissue specimens. **(H)** Sanger sequencing results showed the editing site change of two specific sgRNA targeting to YAP1 proximal APA site. Yellow color indicates YAP1 proximal APA site and arrow indicates the cleavage site of sgRNA. **(I, J)** YAP1 expression was detected by Western blot **(I)** and cell migration assay were performed for 48 hours **(J)** in HCT116 cells with control or stable expression of YAP1 cDNA construct with short or long 3’UTR region. **(K)** Both YAP1 and NUDT21 expression levels were measured in stable clones of control or shNUDT21 HCT116 cells with or without YAP1 overexpression by using Western blot. **(L)**. YAP1 expression levels in paired colon cancer specimens were determined by Western blot. Results were quantified and normalized to b-actin level (n=21). **(M, N)** YAP1 expression was determined by using IHC staining. Representative pictures of YAP1 expression in CRC clinical specimen **(M)** and results were quantified by the TissueFaxs system (n=211 pairs) **(N)**. **(O)** The expression levels of NUDT21 and YAP1 in the public dataset (E-MTAB-990, n=688) were performed Pearson collrecation analysis. **(P)** HCT116 cells were respectively performed ChIP-PCR by using YAP1 and control (IgG) antibodies. The bindings of YAP1 on IL-6, TIMP1 and NR2F2 gene loci were measured by q-PCR (n=3). GAPDH was used as negative control for absent of YAP1 binding gene locus.

**
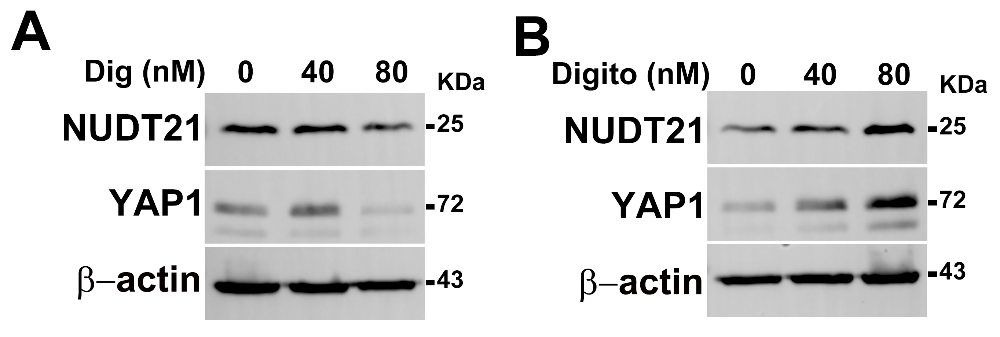
Supplementary figure 4: Digoxin reduced both NUDT21 and YAP1 expression in colorectal cancer cells.**

**(A, B)** HCT116 cells were treated with different concentrations of digoxin (Dig) **(A)** or Digitoxigenin (Digito) **(B)** for 24 hours. Both YAP1 and NUDT21 expression levels were measured by Western blot.
