# Supplementary material for: Targeting NUDT21-mediated alternative polyadenylation of oncogenes ameliorates colorectal cancer malignancy and metastasis
Source: Br J Cancer. 2026 May 6;135(4):518–31. doi: 10.1038/s41416-026-03451-9 (PMC13427831; doi:10.1038/s41416-026-03451-9)
Supplement: Supplementary file 2 — Supplementary Table S1, S2, S6 and S7 [file 41416_2026_3451_MOESM2_ESM.docx]

**Table S1. The list of primer sequences used in this study**

| **Gene** |  | **Sequence (5' to 3')** |
| --- | --- | --- |
| **NUDT21_ realtime** | **F** | **CCCAGGAGAAGATGAAGTTG** |
| **NUDT21_realtime** | **R** | **CCACCAGTTACCAATGCAA** |
| **H18s_ realtime** | **F** | **GTGTGCCTACCCTACG** |
| **H18s_ realtime** | **R** | **TGACCCGCACTTACTG** |
| **NR2F2_ realtime** | **F** | **CCTCAACTGCCACTCGTACCT** |
| **NR2F2_realtime** | **R** | **TCGCAAATGTTCTCGATACCC** |
| **YAP1_realtime** | **F** | **AGA GGC TGC GGC TGA AAC** |
| **YAP1_realtime** | **R** | **TTGCTGTGCTGGGATTGATA** |
| **ROBO4_ realtime** | **F** | **TTCCAAGAACCTTTCCCAAA** |
| **ROBO4_realtime** | **R** | **GAGATGACGAGTAACCAGCTCA** |
| **CCL2_realtime** | **F** | **AGCAGCCACCTTCATTCC** |
| **CCL2_realtime** | **R** | **GCTTCTTTGGGACACTTGCT** |
| **TIMP1_realtime** | **F** | **CTGTTGTTGCTGTGGCTGAT** |
| **TIMP1_realtime** | **R** | **TCTGGTTGACTTCTGGTGTCC** |
| **IL-6_realtime** | **F** | **ACAGCCACTCACCTCTTCA** |
| **IL-6_realtime** | **R** | **TCTTTGCTGCTTTCACACAT** |
| **YAP1 Proximal 3’PCR_realtime** | **F** | **CCA GTG GAA AAA CAT GAT TTA CTG GTC** |
| **YAP1 Distal 3’PCR_realtime** | **R** | **GGT TGT ATT GGG TAG CAT TGG GAT AAG** |
| **YAP1-ChIP_ROBO4_realtime** | **F** | **CCATCACTGAAACTATTGGAGGA** |
| **YAP1-ChIP_ROBO4_realtime** | **R** | **AGGCTCTTGGGACTGGAAAG** |
| **YAP1-ChIP_IL6_realtime** | **F** | **CTAGTTGTGTCTTGCCATGCT** |
| **YAP1-ChIP_IL6_realtime** | **R** | **ATCTTTGTTGGAGGGTGAGG** |
| **YAP1-ChIP_NR2F2_realtime** | **F** | **TGAGCTGATCGCGGAGAA** |
| **YAP1-ChIP_NR2F2_realtime** | **R** | **GGGAGAGGAGGAGGAGAAGA** |
| **YAP1-ChIP_CCL2_realtime** | **F** | **AGTTTCCTCGCTTCCTTCCT** |
| **YAP1-ChIP_CCL2_realtime** | **R** | **ACTGCTGAGACCAAATGAGC** |
| **YAP1-ChIP_TIMP1_realtime** | **F** | **TGCGGCCTCTAAGCTCTC** |
| **YAP1-ChIP_TIMP1_realtime** | **R** | **TACTCATCCACCCACCATCA** |
| **NC-ChIP_GAPDH_realtime** | **F** | **AGTGTTTCCCTCCCTCGTCT** |
| **NC-ChIP_GAPDH_realtime** | **R** | **GAAGTTGTGAGCCTGTCTGG** |
| **NUDT21 siRNA#1** | **4392421#s21770** | **GCACCAUUGUUUGAAUUGUtt** |
| **NUDT21 siRNA#2** | **4392421#s21771** | **CCAUCAACCUGUACCCUCUtt** |
| **NUDT21 siRNA#3** | **4392421#s21772** | **GGUCAUUGACGAUUGCAUUtt** |
| **sgRNA(76F) for Proximal APA YAP1 3’UTR** | **Sense** | **CACCGACATACTCTAATATAGATTT** |
| **sgRNA(76F) for Proximal APA YAP1 3’UTR** | **Antisense** | **AAACAAATCTATATTAGAGTATGTC** |
| **sgRNA(54R) for Proximal APA YAP1 3’UTR** | **Sense** | **CACCGAAAATCTATATTAGAGTATG** |
| **sgRNA(54R) for Proximal APA YAP1 3’UTR** | **Antisense** | **AAACCATACTCTAATATAGATTTTC** |
| **YAP1_promoxial 3’UTR_clone primers**  **(PCR size 820 bp)** | **F** | **(TTAATTAATTAATTAA) PacI RE site**  **TGCCACCAAGCTAGATAAAGAA** |
| **YAP1_promoxial 3’UTR_clone primers** | **R** | **(TTAATTAATTAATTAA) PacI RE site**  **AAACCAACCAACCAACCAAC** |
| **YAP1_distal 3’UTR_clone primers**  **(PCR size 2914 bp)** | **F** | **(TTAATTAATTAATTAA) PacI RE site**  **TGCCACCAAGCTAGATAAAGAA** |
| **YAP1_distal 3’UTR_clone primers** | **R** | **(TTAATTAATTAATTAA) PacI RE site**  **TGTGCTCCATCAACCATAAG** |

**Table S2. The list of Antibodies used in this study**

| **Antibody** | **Host** | **Source** | **Cat#** | **Dilution factor** |
| --- | --- | --- | --- | --- |
| **NUDT21** | **Mouse** | **Santa Curz** | **E1717** | **1:500 (WB);** |
| **β-actin** | **Mouse** | **Novus** | **NB600-501** | **1:10000 (WB)** |
| **GAPDH** | **Mouse** | **Proteintech** | **60004-1-IG** | **1:5000 (WB)** |
| **YAP1** | **Rabbit** | **Cell Signaling** | **14074** | **1:2000 (WB);** |
| **Flag** | **Mouse** | **Sigma-Aldrich** | **F1804** | **1:2000 (WB)** |
| **Myc-tag** | **Mouse** | **Cell Signaling** | **2276** | **1:2000 (WB)** |
| **E-cadherin** | **Rabbit** | **Cell Signaling** | **3195** | **1:2000 (WB)** |
| **Vimentin** | **Rabbit** | **Cell Signaling** | **5741S** | **1:2000 (WB)** |
| **Goat anti mouse IgG** |  | **Jackson Immnuno Research** | **115-036-062** |  |
| **Goat anti Rabbit IgG** |  | **Jackson Immnuno Research** | **110-035-003** |  |

**Table S7. The list of genes upregulated in colorectal cancer and their positive correlation with NUDT21**

| **Gene** | **Peason CORR** | **p value** |
| --- | --- | --- |
| **NUDT 21** | **1.000** | **0** |
| **SUV39H2** | **0.532** | **1.34E-22** |
| **GATC** | **0.470** | **2.51E-17** |
| **NUP155** | **0.437** | **5.86E-15** |
| **METTL9** | **0.433** | **1.18E-14** |
| **NUP54** | **0.428** | **2.34E-14** |
| **TMPO** | **0.418** | **1.09E-13** |
| **GART** | **0.413** | **2.14E-13** |
| **FGFR1OP** | **0.404** | **8.22E-13** |
| **BUB3** | **0.401** | **1.32E-12** |
| **PUS7L** | **0.381** | **1.99E-11** |
| **C4orf46** | **0.377** | **3.27E-11** |
| **NEIL3** | **0.360** | **2.61E-10** |
| **PTGES3** | **0.352** | **6.88E-10** |
| **CEP41** | **0.351** | **7.80E-10** |
| **PAICS** | **0.350** | **8.54E-10** |
| **RPGRIP1L** | **0.345** | **1.49E-09** |
| **POT1** | **0.344** | **1.82E-09** |
| **KIF2A** | **0.334** | **5.50E-09** |
| **PRPS2** | **0.332** | **6.76E-09** |
| **TSN** | **0.332** | **7.02E-09** |
| **MSH2** | **0.326** | **1.27E-08** |
| **SPIN4** | **0.320** | **2.54E-08** |
| **PRMT3** | **0.314** | **4.76E-08** |
| **VRK1** | **0.309** | **7.69E-08** |
| **TRAPPC13** | **0.303** | **1.41E-07** |
| **EPB41L5** | **0.303** | **1.46E-07** |
| **RFC3** | **0.302** | **1.61E-07** |
| **BAG4** | **0.293** | **3.65E-07** |
| **AP1S3** | **0.289** | **5.63E-07** |
| **TMEM126B** | **0.286** | **7.50E-07** |
| **PI4K2B** | **0.282** | **1.05E-06** |
| **ZFAND1** | **0.275** | **2.06E-06** |
| **DHFR** | **0.272** | **2.50E-06** |
| **SLC25A36** | **0.271** | **2.78E-06** |
| **TSEN15** | **0.269** | **3.27E-06** |
| **TMEM237** | **0.263** | **5.61E-06** |
| **RSL24D1** | **0.256** | **9.97E-06** |
| **FAM192A** | **0.256** | **1.01E-05** |
| **CDCA7** | **0.256** | **1.04E-05** |
| **CSE1L** | **0.255** | **1.08E-05** |
| **ZNF200** | **0.252** | **1.44E-05** |
| **TCAIM** | **0.252** | **1.44E-05** |
| **CD46** | **0.250** | **1.59E-05** |
| **NPM1** | **0.250** | **1.60E-05** |
| **PCMT1** | **0.250** | **1.65E-05** |
| **C17orf75** | **0.243** | **2.96E-05** |
| **BPHL** | **0.235** | **5.21E-05** |
| **CNOT1** | **0.234** | **5.80E-05** |
| **SETD6** | **0.230** | **7.78E-05** |
| **YAP1** | **0.229** | **8.16E-05** |
| **WDR73** | **0.225** | **1.15E-04** |
| **SRSF6** | **0.223** | **1.33E-04** |
| **DKC1** | **0.222** | **1.40E-04** |
| **CASK** | **0.220** | **1.61E-04** |
| **HAUS1** | **0.218** | **1.85E-04** |
| **AIG1** | **0.216** | **2.10E-04** |
| **LIMS1** | **0.215** | **2.33E-04** |
| **PSMB5** | **0.213** | **2.51E-04** |
| **COX20** | **0.206** | **4.17E-04** |
| **SUMO1** | **0.195** | **8.34E-04** |

| **Gene** | **Peason CORR** | **p value** |
| --- | --- | --- |
| **NUDT 21** | **1.000** | **0** |
| **SUV39H2** | **0.532** | **1.34E-22** |
| **GATC** | **0.470** | **2.51E-17** |
| **NUP155** | **0.437** | **5.86E-15** |
| **METTL9** | **0.433** | **1.18E-14** |
| **NUP54** | **0.428** | **2.34E-14** |
| **TMPO** | **0.418** | **1.09E-13** |
| **GART** | **0.413** | **2.14E-13** |
| **FGFR1OP** | **0.404** | **8.22E-13** |
| **BUB3** | **0.401** | **1.32E-12** |
| **PUS7L** | **0.381** | **1.99E-11** |
| **C4orf46** | **0.377** | **3.27E-11** |
| **NEIL3** | **0.360** | **2.61E-10** |
| **PTGES3** | **0.352** | **6.88E-10** |
| **CEP41** | **0.351** | **7.80E-10** |
| **PAICS** | **0.350** | **8.54E-10** |
| **RPGRIP1L** | **0.345** | **1.49E-09** |
| **POT1** | **0.344** | **1.82E-09** |
| **KIF2A** | **0.334** | **5.50E-09** |
| **PRPS2** | **0.332** | **6.76E-09** |
| **TSN** | **0.332** | **7.02E-09** |
| **MSH2** | **0.326** | **1.27E-08** |
| **SPIN4** | **0.320** | **2.54E-08** |
| **PRMT3** | **0.314** | **4.76E-08** |
| **VRK1** | **0.309** | **7.69E-08** |
| **TRAPPC13** | **0.303** | **1.41E-07** |
| **EPB41L5** | **0.303** | **1.46E-07** |
| **RFC3** | **0.302** | **1.61E-07** |
| **BAG4** | **0.293** | **3.65E-07** |
| **AP1S3** | **0.289** | **5.63E-07** |
| **TMEM126B** | **0.286** | **7.50E-07** |
| **PI4K2B** | **0.282** | **1.05E-06** |
| **ZFAND1** | **0.275** | **2.06E-06** |
| **DHFR** | **0.272** | **2.50E-06** |
| **SLC25A36** | **0.271** | **2.78E-06** |
| **TSEN15** | **0.269** | **3.27E-06** |
| **TMEM237** | **0.263** | **5.61E-06** |
| **RSL24D1** | **0.256** | **9.97E-06** |
| **FAM192A** | **0.256** | **1.01E-05** |
| **CDCA7** | **0.256** | **1.04E-05** |
| **CSE1L** | **0.255** | **1.08E-05** |
| **ZNF200** | **0.252** | **1.44E-05** |
| **TCAIM** | **0.252** | **1.44E-05** |
| **CD46** | **0.250** | **1.59E-05** |
| **NPM1** | **0.250** | **1.60E-05** |
| **PCMT1** | **0.250** | **1.65E-05** |
| **C17orf75** | **0.243** | **2.96E-05** |
| **BPHL** | **0.235** | **5.21E-05** |
| **CNOT1** | **0.234** | **5.80E-05** |
| **SETD6** | **0.230** | **7.78E-05** |
| **YAP1** | **0.229** | **8.16E-05** |
| **WDR73** | **0.225** | **1.15E-04** |
| **SRSF6** | **0.223** | **1.33E-04** |
| **DKC1** | **0.222** | **1.40E-04** |
| **CASK** | **0.220** | **1.61E-04** |
| **HAUS1** | **0.218** | **1.85E-04** |
| **AIG1** | **0.216** | **2.10E-04** |
| **LIMS1** | **0.215** | **2.33E-04** |
| **PSMB5** | **0.213** | **2.51E-04** |
| **COX20** | **0.206** | **4.17E-04** |
| **SUMO1** | **0.195** | **8.34E-04** |
| **SETMAR** | **0.192** | **1.02E-03** |
| **HMGB2** | **0.192** | **1.04E-03** |
| **GPR160** | **0.189** | **1.18E-03** |
| **TEX30** | **0.189** | **1.21E-03** |
| **SLC11A2** | **0.183** | **1.73E-03** |
| **RBM4** | **0.183** | **1.73E-03** |
| **MTA3** | **0.175** | **2.72E-03** |
| **ZNRF3** | **0.169** | **3.79E-03** |
| **GXYLT1** | **0.168** | **4.12E-03** |
| **SLC3A2** | **0.167** | **4.43E-03** |
| **BBS10** | **0.164** | **5.14E-03** |
| **ZNF107** | **0.164** | **5.22E-03** |
| **DHX40** | **0.163** | **5.49E-03** |
| **MAP3K7** | **0.160** | **6.27E-03** |
| **PREPL** | **0.157** | **7.57E-03** |
| **NUDT7** | **0.150** | **1.06E-02** |
| **CLCN5** | **0.139** | **1.79E-02** |
| **ARNTL2** | **0.139** | **1.79E-02** |
| **IMMP2L** | **0.137** | **1.97E-02** |
| **MTR** | **0.137** | **1.99E-02** |
| **SCAMP1** | **0.136** | **2.01E-02** |
| **CPSF4** | **0.118** | **4.55E-02** |
| **TRAPPC11** | **0.116** | **4.84E-02** |

**Table S8. The list of potential NUDT21 inhibitors from CMAP analysis**

| **Rank** | **cmap name and cell line** | **mean** | **n** | **enrichment** | **p value** |
| --- | --- | --- | --- | --- | --- |
| **1** | **helveticoside - MCF7** | **0.751** | **3** | **0.973** | **0.00004** |
| **2** | **bisacodyl - MCF7** | **0.853** | **2** | **0.992** | **0.00006** |
| **4** | **hexestrol - MCF7** | **0.901** | **2** | **0.988** | **0.00016** |
| **3** | **thiostrepton - MCF7** | **0.856** | **2** | **0.989** | **0.00016** |
| **5** | **securinine - MCF7** | **0.818** | **2** | **0.985** | **0.00044** |
| **6** | **ouabain - MCF7** | **0.803** | **2** | **0.985** | **0.00044** |
| **7** | **lanatoside C - MCF7** | **0.73** | **3** | **0.93** | **0.00062** |
| **8** | **digoxin - MCF7** | **0.806** | **2** | **0.98** | **0.0007** |
| **9** | **felbinac - MCF7** | **-0.804** | **2** | **-0.981** | **0.00076** |
| **10** | **tanespimycin - PC3** | **0.44** | **12** | **0.536** | **0.00082** |
| **11** | **cephaeline - MCF7** | **-0.615** | **3** | **-0.912** | **0.00118** |
| **12** | **cefmetazole - MCF7** | **0.739** | **2** | **0.968** | **0.00173** |
| **13** | **proscillaridin - MCF7** | **0.786** | **2** | **0.967** | **0.00185** |
| **14** | **digitoxigenin - MCF7** | **0.795** | **2** | **0.966** | **0.00205** |
| **15** | **astemizole - MCF7** | **0.719** | **2** | **0.965** | **0.00211** |
| **16** | **mianserin - PC3** | **0.692** | **2** | **0.956** | **0.00336** |
| **17** | **protriptyline - MCF7** | **0.783** | **2** | **0.955** | **0.00374** |
| **18** | **leflunomide - MCF7** | **-0.695** | **2** | **-0.957** | **0.00384** |
| **19** | **vincamine - MCF7** | **-0.4** | **3** | **-0.872** | **0.00415** |
| **20** | **CP-690334-01 - PC3** | **-0.336** | **4** | **-0.785** | **0.00428** |
