# Supplementary material for: Targeting NUDT21-mediated alternative polyadenylation of oncogenes ameliorates colorectal cancer malignancy and metastasis
Source: Br J Cancer. 2026 May 6;135(4):518–31. doi: 10.1038/s41416-026-03451-9 (PMC13427831; doi:10.1038/s41416-026-03451-9)
Supplement: Supplementary file 4 — Supplementary Table S4 [file 41416_2026_3451_MOESM4_ESM.pdf]

gene_id	GeneSymbol	Log2FC	pvalue	padj
ENSG00000137309	HMGA1	-2.62	0	0
ENSG00000167005	NUDT21	-4.31	0	0
ENSG00000189060	H1-0	-2.24	0	0
ENSG00000103257	SLC7A5	-2.09	1.3591E-264	6.7998E-261
ENSG00000125968	ID1	-2.29	1.0487E-248	4.1971E-245
ENSG00000135318	NT5E	2.44	3.3935E-229	1.1318E-225
ENSG00000187837	H1-2	3.30	3.6835E-227	1.053E-223
ENSG00000130164	LDLR	1.85	1.8492E-216	4.6258E-213
ENSG00000013306	SLC25A39	-2.18	2.0466E-187	4.5508E-184
ENSG00000124762	CDKN1A	-1.89	4.9486E-176	9.9031E-173
ENSG00000101057	MYBL2	-2.12	3.3446E-161	6.0847E-158
ENSG00000245532	NEAT1	-1.90	3.2476E-152	5.416E-149
ENSG00000122359	ANXA11	-1.94	8.9894E-144	1.3838E-140
ENSG00000130309	COLGALT1	-1.73	1.0143E-143	1.4498E-140
ENSG00000171346	KRT15	2.39	2.2724E-141	3.0316E-138
ENSG00000132470	ITGB4	-1.81	2.8352E-140	3.5461E-137
ENSG00000131653	TRAF7	-2.03	7.4456E-140	8.7648E-137
ENSG00000100889	PCK2	-2.01	7.5342E-138	8.3763E-135
ENSG00000167552	TUBA1A	2.28	1.8499E-137	1.9485E-134
ENSG00000070087	PFN2	-1.72	1.5447E-133	1.5456E-130
ENSG00000135074	ADAM19	-2.24	1.216E-131	1.1588E-128
ENSG00000158195	WASF2	-1.94	1.8182E-131	1.6539E-128
ENSG00000179134	SAMD4B	-2.15	1.994E-131	1.7349E-128
ENSG00000182481	KPNA2	1.47	2.9533E-124	2.4626E-121
ENSG00000166123	GPT2	-2.02	6.1668E-124	4.9364E-121
ENSG00000170540	ARL6IP1	1.34	8.623E-123	6.637E-120
ENSG00000101188	NTSR1	-1.58	6.7276E-122	4.9864E-119
ENSG00000120738	EGR1	1.91	2.8679E-116	2.0497E-113
ENSG00000204388	HSPA1B	1.69	2.4753E-115	1.7081E-112
ENSG00000142875	PRKACB	1.58	1.7817E-114	1.1885E-111
ENSG00000142657	PGD	-1.83	6.3281E-114	4.0851E-111
ENSG00000183723	CMTM4	-2.01	8.7302E-110	5.4596E-107
ENSG00000137818	RPLP1	-1.66	8.2369E-109	4.9951E-106
ENSG00000100297	MCM5	-1.67	4.011E-105	2.3608E-102
ENSG00000168209	DDIT4	-1.52	5.7767E-105	3.303E-102
ENSG00000123358	NR4A1	2.73	1.8158E-104	1.0094E-101
ENSG00000187514	PTMA	-1.29	2.5907E-103	1.4012E-100
ENSG00000062716	VMP1	1.34	4.494E-103	2.3667E-100
ENSG00000174903	RAB1B	-1.76	7.6393E-103	3.92E-100
ENSG00000148730	EIF4EBP2	-1.79	7.3261E-102	3.6653E-99
ENSG00000153551	CMTM7	-2.68	1.4043E-100	6.85453E-98
ENSG00000140564	FURIN	-2.16	9.64852E-98	4.59729E-95
ENSG00000144354	CDCA7	-2.21	1.04854E-96	4.87987E-94
ENSG00000086061	DNAJA1	1.34	1.42498E-95	6.48105E-93
ENSG00000116133	DHCR24	-1.33	3.76398E-95	1.67388E-92

ENSG00000176619	LMNB2	-1.27	1.32079E-93	5.746E-91
ENSG00000124766	SOX4	1.61	4.68757E-92	1.99591E-89
ENSG00000166913	YWHAB	-1.34	1.13796E-91	4.74436E-89
ENSG00000163898	LIPH	2.39	1.03998E-89	4.24734E-87
ENSG00000144821	MYH15	2.46	4.40283E-89	1.76219E-86
ENSG00000178952	TUFM	-1.41	4.65067E-89	1.82489E-86
ENSG00000237190	CDKN2AIPNL	-2.22	2.12113E-88	8.16307E-86
ENSG00000179632	MAF1	-1.53	1.19439E-86	4.50985E-84
ENSG00000205250	E2F4	-1.84	2.24559E-86	8.322E-84
ENSG00000121900	TMEM54	-2.74	3.35689E-86	1.22142E-83
ENSG00000092820	EZR	-1.28	3.91446E-86	1.39886E-83
ENSG00000128564	VGF	1.67	8.72965E-86	3.06487E-83
ENSG00000186480	INSIG1	1.53	1.24931E-85	4.31054E-83
ENSG00000161642	ZNF385A	-1.98	1.47957E-85	5.01849E-83
ENSG00000128342	LIF	-1.54	2.70339E-85	9.0167E-83
ENSG00000135924	DNAJB2	1.67	5.47003E-85	1.79453E-82
ENSG00000161638	ITGA5	-1.65	6.96196E-85	2.24714E-82
ENSG00000130520	LSM4	-1.48	1.59036E-84	5.0518E-82
ENSG00000143742	SRP9	-1.52	2.82452E-84	8.83191E-82
ENSG00000149532	CPSF7	-1.53	1.31733E-82	4.05574E-80
ENSG00000226950	DANCR	-1.98	1.34832E-82	4.08828E-80
ENSG00000106366	SERPINE1	2.02	3.35555E-82	1.00226E-79
ENSG00000122965	RBM19	1.78	1.31402E-81	3.86708E-79
ENSG00000002834	LASP1	-1.21	4.03121E-81	1.16917E-78
ENSG00000149948	HMGA2	1.30	5.18651E-81	1.48275E-78
ENSG00000105976	MET	1.43	8.51367E-81	2.39966E-78
ENSG00000164880	INTS1	-1.39	2.3068E-79	6.41163E-77
ENSG00000100106	TRIOBP	-1.24	2.28167E-78	6.25489E-76
ENSG00000138759	FRAS1	-1.68	5.28019E-78	1.42794E-75
ENSG00000184117	NIPSNAP1	-1.44	6.22634E-78	1.66135E-75
ENSG00000175224	ATG13	-2.04	6.77708E-78	1.78451E-75
ENSG00000026559	KCNG1	-2.27	4.58816E-77	1.19245E-74
ENSG00000011426	ANLN	1.40	4.76623E-77	1.22284E-74
ENSG00000125398	SOX9	-1.37	6.99403E-77	1.7717E-74
ENSG00000002745	WNT16	1.31	1.17693E-76	2.94409E-74
ENSG00000132698	RAB25	-2.52	1.26642E-76	3.12885E-74
ENSG00000142453	CARM1	-1.58	3.78413E-76	9.23511E-74
ENSG00000167355	OR51B5	-2.08	4.56403E-76	1.10043E-73
ENSG00000101444	AHCY	-1.17	5.01362E-76	1.19443E-73
ENSG00000132471	WBP2	-1.62	6.3397E-75	1.49259E-72
ENSG00000123562	MORF4L2	1.06	1.21001E-73	2.81565E-71
ENSG00000139514	SLC7A1	-1.21	1.71721E-73	3.94999E-71
ENSG00000137713	PPP2R1B	1.95	6.6006E-73	1.50104E-70
ENSG00000151929	BAG3	1.28	9.78736E-73	2.20073E-70
ENSG00000063245	EPN1	-1.70	1.52334E-72	3.38724E-70
ENSG00000066117	SMARCD1	-1.75	1.61537E-72	3.55238E-70

ENSG00000214078	CPNE1	-1.43	1.0004E-71	2.17608E-69
ENSG00000139146	SINHCAF	-1.46	1.14312E-71	2.4598E-69
ENSG00000204516	MICB	1.46	1.90716E-71	4.06023E-69
ENSG00000196878	LAMB3	1.38	2.00437E-71	4.22226E-69
ENSG00000080824	HSP90AA1	1.24	2.20501E-71	4.59653E-69
ENSG00000264112	AC015813.1	-1.45	2.45369E-71	5.0622E-69
ENSG00000135372	NAT10	-1.41	1.37157E-70	2.8008E-68
ENSG00000112576	CCND3	1.96	1.40863E-70	2.84743E-68
ENSG00000264364	DYNLL2	-1.06	1.50011E-70	3.00203E-68
ENSG00000167900	TK1	-1.35	2.99823E-69	5.94065E-67
ENSG00000172432	GTPBP2	-1.40	3.52127E-69	6.90859E-67
ENSG00000123159	GIPC1	-1.64	4.95211E-69	9.62153E-67
ENSG00000105971	CAV2	-1.45	7.75468E-69	1.49218E-66
ENSG00000141349	G6PC3	-2.28	3.87729E-68	7.38975E-66
ENSG00000072501	SMC1A	-1.28	4.99301E-68	9.42643E-66
ENSG00000108821	COL1A1	1.77	5.06665E-68	9.47606E-66
ENSG00000109680	TBC1D19	2.69	2.6618E-67	4.93222E-65
ENSG00000197858	GPAA1	-1.47	7.91605E-67	1.45336E-64
ENSG00000144063	MALL	-2.53	1.93103E-66	3.51307E-64
ENSG00000213281	NRAS	1.36	3.28985E-66	5.93121E-64
ENSG00000173020	GRK2	-1.47	3.53912E-66	6.32365E-64
ENSG00000003436	TFPI	1.90	4.87478E-66	8.6331E-64
ENSG00000135373	EHF	-1.77	1.88896E-65	3.31595E-63
ENSG00000062582	MRPS24	-3.06	2.64711E-65	4.60643E-63
ENSG00000152556	PFKM	-1.33	1.13666E-64	1.96094E-62
ENSG00000260032	NORAD	-1.39	1.63122E-64	2.79008E-62
ENSG00000161011	SQSTM1	1.02	4.02707E-64	6.82964E-62
ENSG00000142627	EPHA2	-1.15	4.15698E-64	6.99072E-62
ENSG00000124193	SRSF6	-1.23	5.16777E-64	8.61811E-62
ENSG00000170345	FOS	2.80	7.19263E-64	1.18958E-61
ENSG00000169715	MT1E	-1.35	1.02381E-63	1.67938E-61
ENSG00000108262	GIT1	-1.75	3.15585E-63	5.13454E-61
ENSG00000197879	MYO1C	-1.04	3.94385E-63	6.36487E-61
ENSG00000149782	PLCB3	-1.28	7.26556E-63	1.16319E-60
ENSG00000131016	AKAP12	0.99	1.83656E-62	2.91692E-60
ENSG00000117394	SLC2A1	-1.08	4.3071E-62	6.7869E-60
ENSG00000160803	UBQLN4	-1.59	5.67234E-62	8.86836E-60
ENSG00000227063	RPL41P1	-1.04	5.79475E-62	8.9895E-60
ENSG00000136153	LMO7	2.78	2.43973E-61	3.75568E-59
ENSG00000198911	SREBF2	-1.12	2.62613E-61	4.01176E-59
ENSG00000143575	HAX1	-1.53	4.57179E-61	6.9311E-59
ENSG00000187678	SPRY4	1.25	4.63882E-61	6.97985E-59
ENSG00000124145	SDC4	1.05	5.41956E-61	8.09375E-59
ENSG00000085998	POMGNT1	-1.56	7.23282E-61	1.07217E-58
ENSG00000143878	RHOB	-1.16	1.27937E-60	1.88256E-58
ENSG00000105011	ASF1B	-1.34	1.53276E-60	2.23895E-58

ENSG00000178035	IMPDH2	-0.95	1.91134E-60	2.77172E-58
ENSG00000088986	DYNLL1	1.28	4.03898E-60	5.81497E-58
ENSG00000011009	LYPLA2	-1.71	4.72449E-60	6.75332E-58
ENSG00000107130	NCS1	-1.35	4.79011E-60	6.79856E-58
ENSG00000214182	PTMAP5	-1.25	1.1644E-59	1.64098E-57
ENSG00000105355	PLIN3	1.00	2.93491E-58	4.10723E-56
ENSG00000112972	HMGCS1	1.23	3.65139E-58	5.07442E-56
ENSG00000132002	DNAJB1	0.99	4.61578E-58	6.37041E-56
ENSG00000163933	RFT1	-2.10	9.60278E-58	1.31624E-55
ENSG00000140497	SCAMP2	-1.90	1.40327E-57	1.91036E-55
ENSG00000171865	RNASEH1	-1.54	1.69328E-57	2.28959E-55
ENSG00000142949	PTPRF	-1.06	1.90625E-57	2.56025E-55
ENSG00000115944	COX7A2L	-1.38	4.62117E-57	6.16525E-55
ENSG00000080819	CPOX	-1.04	4.68049E-57	6.20305E-55
ENSG00000213064	SFT2D2	-1.61	7.18744E-57	9.46284E-55
ENSG00000158604	TMED4	-1.56	7.26533E-57	9.50286E-55
ENSG00000119383	PTPA	-1.18	7.34849E-57	9.54923E-55
ENSG00000099204	ABLIM1	-1.04	7.84607E-57	1.013E-54
ENSG00000171552	BCL2L1	-1.24	9.22518E-57	1.18342E-54
ENSG00000087269	NOP14	-1.32	9.9309E-57	1.26584E-54
ENSG00000162616	DNAJB4	1.69	2.36174E-56	2.99133E-54
ENSG00000133318	RTN3	-1.14	2.47249E-56	3.11191E-54
ENSG00000109971	HSPA8	0.92	8.75412E-56	1.09492E-53
ENSG00000148334	PTGES2	-1.34	1.40011E-55	1.7403E-53
ENSG00000132182	NUP210	-1.07	2.00329E-55	2.47468E-53
ENSG00000111481	COPZ1	-1.12	2.08108E-55	2.555E-53
ENSG00000109321	AREG	1.10	5.0527E-55	6.16553E-53
ENSG00000174238	PITPNA	-1.35	7.24134E-55	8.78265E-53
ENSG00000114529	C3orf52	1.65	7.79751E-55	9.40023E-53
ENSG00000105063	PPP6R1	-1.14	1.77464E-54	2.1266E-52
ENSG00000177084	POLE	-1.23	2.76656E-54	3.29549E-52
ENSG00000019186	CYP24A1	-1.04	4.03924E-54	4.78303E-52
ENSG00000167996	FTH1	-0.99	1.6973E-53	1.99802E-51
ENSG00000100401	RANGAP1	0.95	3.0925E-53	3.61913E-51
ENSG00000198925	ATG9A	-2.00	3.31996E-53	3.86274E-51
ENSG00000086730	LAT2	1.95	4.35613E-53	5.03901E-51
ENSG00000087274	ADD1	-1.12	5.20421E-53	5.98544E-51
ENSG00000152137	HSPB8	2.25	7.60562E-53	8.69735E-51
ENSG00000143321	HDGF	-0.98	8.97164E-53	1.02012E-50
ENSG00000100216	TOMM22	-1.37	1.29767E-52	1.46717E-50
ENSG00000162496	DHRS3	-1.70	1.59393E-52	1.79201E-50
ENSG00000073111	MCM2	-1.08	1.90085E-52	2.12513E-50
ENSG00000281398	SNHG4	-1.64	2.02248E-52	2.24854E-50
ENSG00000151553	FAM160B1	1.47	2.37714E-52	2.62825E-50
ENSG00000111605	CPSF6	1.08	2.62583E-52	2.88726E-50
ENSG00000149476	TKFC	-1.52	3.03915E-52	3.32347E-50

ENSG00000090861	AARS1	-0.92	3.21429E-52	3.49589E-50
ENSG00000172927	MYEOV	-1.20	6.25202E-52	6.763E-50
ENSG00000269893	SNHG8	-1.31	1.02513E-51	1.10295E-49
ENSG00000127528	KLF2	-1.59	1.08265E-51	1.15861E-49
ENSG00000166340	TPP1	-1.77	1.18659E-51	1.26308E-49
ENSG00000255248	MIR100HG	1.26	1.35488E-51	1.43459E-49
ENSG00000168003	SLC3A2	-0.94	1.45629E-51	1.53386E-49
ENSG00000168610	STAT3	1.15	1.49926E-51	1.57085E-49
ENSG00000159658	EFCAB14	-1.47	1.84642E-51	1.92451E-49
ENSG00000164818	DNAAF5	-1.41	3.04937E-51	3.16186E-49
ENSG00000143761	ARF1	-1.02	1.09903E-50	1.1337E-48
ENSG00000149328	GLB1L2	-1.63	1.53752E-50	1.57789E-48
ENSG00000007376	RPUSD1	-1.56	1.69782E-50	1.73351E-48
ENSG00000130816	DNMT1	-1.05	1.97445E-50	2.00572E-48
ENSG00000168906	MAT2A	-0.91	2.67014E-50	2.69873E-48
ENSG00000189159	JPT1	1.06	3.02106E-50	3.03806E-48
ENSG00000155463	OXA1L	-1.15	6.13188E-50	6.13556E-48
ENSG00000197903	H2BC12	2.40	8.79455E-50	8.75605E-48
ENSG00000174028	FAM3C2	1.34	9.26033E-50	9.17414E-48
ENSG00000180304	OAZ2	1.01	1.37069E-49	1.35125E-47
ENSG00000106785	TRIM14	-1.99	1.52693E-49	1.49789E-47
ENSG00000143811	PYCR2	-1.21	3.30254E-49	3.22392E-47
ENSG00000111676	ATN1	-1.19	4.04139E-49	3.92603E-47
ENSG00000155229	MMS19	-1.21	4.56507E-49	4.41334E-47
ENSG00000112033	PPARD	-1.47	5.03848E-49	4.8476E-47
ENSG00000141959	PFKL	-1.33	5.15102E-49	4.93216E-47
ENSG00000107731	UNC5B	-1.84	5.43625E-49	5.18049E-47
ENSG00000105053	VRK3	-2.01	6.65489E-49	6.31174E-47
ENSG00000272734	ADIRF-AS1	1.90	9.97526E-49	9.41627E-47
ENSG00000110092	CCND1	-0.88	2.43608E-48	2.28877E-46
ENSG00000196498	NCOR2	-1.35	3.31685E-48	3.10172E-46
ENSG00000091409	ITGA6	1.05	4.44781E-48	4.13998E-46
ENSG00000158850	B4GALT3	-1.94	8.0627E-48	7.46994E-46
ENSG00000083857	FAT1	0.96	1.29091E-47	1.19049E-45
ENSG00000181019	NQO1	-0.85	2.24862E-47	2.06419E-45
ENSG00000196642	RABL6	-1.25	2.59033E-47	2.36701E-45
ENSG00000203879	GDI1	1.07	2.80883E-47	2.55501E-45
ENSG00000117318	ID3	-1.46	2.94054E-47	2.66271E-45
ENSG00000005884	ITGA3	-0.91	4.15996E-47	3.74996E-45
ENSG00000070423	RNF126	-1.59	6.94565E-47	6.23302E-45
ENSG00000071127	WDR1	0.88	1.06907E-46	9.55103E-45
ENSG00000133313	CNDP2	-1.35	1.31816E-46	1.1724E-44
ENSG00000084774	CAD	-1.09	2.0094E-46	1.7793E-44
ENSG00000049239	H6PD	-1.95	2.8801E-46	2.53905E-44
ENSG00000135486	HNRNPA1	-0.83	4.69133E-46	4.11767E-44
ENSG00000184640	SEPTIN9	-0.88	6.8562E-46	5.99154E-44

ENSG00000134324	LPIN1	1.57	8.82208E-46	7.67598E-44
ENSG00000119812	FAM98A	1.16	1.11764E-45	9.68235E-44
ENSG00000168528	SERINC2	-1.66	2.63833E-45	2.27578E-43
ENSG00000143819	EPHX1	-1.63	3.04706E-45	2.61707E-43
ENSG00000159792	PSKH1	-2.65	3.06705E-45	2.62299E-43
ENSG00000006534	ALDH3B1	-2.12	6.29191E-45	5.35803E-43
ENSG00000065320	NTN1	2.08	1.11141E-44	9.42442E-43
ENSG00000126003	PLAGL2	-1.74	1.14303E-44	9.6516E-43
ENSG00000187867	PALM3	1.76	2.40974E-44	2.02621E-42
ENSG00000130702	LAMA5	-1.00	3.46678E-44	2.90281E-42
ENSG00000142227	EMP3	1.11	5.60365E-44	4.67251E-42
ENSG00000060656	PTPRU	-1.41	5.72628E-44	4.75495E-42
ENSG00000186594	MIR22HG	2.33	6.21866E-44	5.14248E-42
ENSG00000164611	PTTG1	0.92	6.52707E-44	5.3753E-42
ENSG00000050426	LETMD1	-1.46	6.57402E-44	5.39178E-42
ENSG00000169679	BUB1	1.07	1.10796E-43	9.04999E-42
ENSG00000138031	ADCY3	-1.57	1.36142E-43	1.10751E-41
ENSG00000164300	SERINC5	-1.69	2.70577E-43	2.19223E-41
ENSG00000148154	UGCG	1.22	4.24521E-43	3.42561E-41
ENSG00000115677	HDLBP	-0.79	5.36043E-43	4.30815E-41
ENSG00000197744	PTMAP2	-1.19	6.22051E-43	4.9794E-41
ENSG00000065559	MAP2K4	1.39	9.16119E-43	7.30413E-41
ENSG00000221890	NPTXR	-2.11	9.64908E-43	7.66259E-41
ENSG00000104518	GSDMD	-1.42	1.25965E-42	9.96366E-41
ENSG00000163235	TGFA	-1.35	1.39891E-42	1.10217E-40
ENSG00000184661	CDCA2	1.60	1.50098E-42	1.17794E-40
ENSG00000054690	PLEKHH1	-2.17	1.68818E-42	1.31968E-40
ENSG00000227500	SCAMP4	-1.50	2.70566E-42	2.10684E-40
ENSG00000182180	MRPS16	-1.13	3.15987E-42	2.45098E-40
ENSG00000186918	ZNF395	-1.74	3.44124E-42	2.65892E-40
ENSG00000157593	SLC35B2	-1.39	3.56754E-42	2.74192E-40
ENSG00000196352	CD55	1.20	3.57606E-42	2.74192E-40
ENSG00000065060	UHRF1BP1	-1.38	6.30352E-42	4.81473E-40
ENSG00000184363	PKP3	-1.17	7.04544E-42	5.36097E-40
ENSG00000214049	UCA1	-1.47	8.35477E-42	6.33317E-40
ENSG00000097021	ACOT7	-1.10	8.82704E-42	6.66591E-40
ENSG00000175556	LONRF3	3.17	1.08855E-41	8.18953E-40
ENSG00000088448	ANKRD10	0.95	1.2114E-41	9.07962E-40
ENSG00000275023	MLLT6	-1.15	1.2765E-41	9.53182E-40
ENSG00000103018	CYB5B	-0.81	1.77294E-41	1.31896E-39
ENSG00000128283	CDC42EP1	-1.09	1.94844E-41	1.44415E-39
ENSG00000162236	STX5	-1.75	2.20834E-41	1.63075E-39
ENSG00000169174	PCSK9	1.17	2.54304E-41	1.871E-39
ENSG00000101447	FAM83D	1.05	2.68988E-41	1.97179E-39
ENSG00000116478	HDAC1	-0.86	3.03872E-41	2.21938E-39
ENSG00000114416	FXR1	0.91	5.0329E-41	3.66248E-39

ENSG00000112658	SRF	-1.41	5.31909E-41	3.85673E-39
ENSG00000162522	KIAA1522	-1.33	5.36455E-41	3.87564E-39
ENSG00000162430	SELENON	-0.97	5.77405E-41	4.15648E-39
ENSG00000138757	G3BP2	0.98	8.24884E-41	5.91669E-39
ENSG00000198700	IPO9	-1.02	8.34419E-41	5.96371E-39
ENSG00000155660	PDIA4	0.81	9.25296E-41	6.58969E-39
ENSG00000108468	CBX1	0.80	1.03823E-40	7.36773E-39
ENSG00000132688	NES	1.09	1.34609E-40	9.51869E-39
ENSG00000105723	GSK3A	-1.26	1.78865E-40	1.26037E-38
ENSG00000024422	EHD2	-1.14	2.0192E-40	1.41783E-38
ENSG00000196372	ASB13	-2.41	2.28928E-40	1.60185E-38
ENSG00000072682	P4HA2	1.20	2.66433E-40	1.85779E-38
ENSG00000102996	MMP15	-1.19	2.83237E-40	1.9681E-38
ENSG00000196935	SRGAP1	-1.56	3.0567E-40	2.11663E-38
ENSG00000103067	ESRP2	-1.60	3.24818E-40	2.24147E-38
ENSG00000112183	RBM24	1.88	3.80825E-40	2.61892E-38
ENSG00000144655	CSRNP1	1.49	7.38418E-40	5.06069E-38
ENSG00000108846	ABCC3	-1.99	8.37175E-40	5.71793E-38
ENSG00000160014	CALM3	-0.91	1.36973E-39	9.32348E-38
ENSG00000033100	CHPF2	1.41	1.47665E-39	1.00172E-37
ENSG00000100418	DESI1	-1.44	2.04052E-39	1.37956E-37
ENSG00000103043	VAC14	-1.57	2.10112E-39	1.41575E-37
ENSG00000182004	SNRPE	-1.88	2.48732E-39	1.67034E-37
ENSG00000228705	LINC00659	2.60	2.71161E-39	1.81488E-37
ENSG00000099875	MKNK2	-0.92	3.36981E-39	2.24789E-37
ENSG00000138316	ADAMTS14	-2.06	3.93037E-39	2.61311E-37
ENSG00000104142	VPS18	1.27	4.01627E-39	2.66138E-37
ENSG00000130826	DKC1	-0.93	5.70861E-39	3.77032E-37
ENSG00000141682	PMAIP1	0.94	1.00059E-38	6.58678E-37
ENSG00000179912	R3HDM2	-1.96	1.38061E-38	9.05862E-37
ENSG00000158042	MRPL17	-1.74	1.40056E-38	9.15945E-37
ENSG00000002587	HS3ST1	-1.30	1.40712E-38	9.17238E-37
ENSG00000248323	LUCAT1	2.32	1.55105E-38	1.00778E-36
ENSG00000167123	CERCAM	-1.61	1.71954E-38	1.11364E-36
ENSG00000157502	PWWP3B	2.28	1.7317E-38	1.11789E-36
ENSG00000012660	ELOVL5	-1.03	2.27194E-38	1.46193E-36
ENSG00000160211	G6PD	-1.01	2.29858E-38	1.47433E-36
ENSG00000120694	HSPH1	0.99	2.38292E-38	1.52355E-36
ENSG00000100129	EIF3L	-0.90	2.51277E-38	1.60145E-36
ENSG00000118900	UBN1	-1.24	2.58433E-38	1.64183E-36
ENSG00000168476	REEP4	-1.16	2.93123E-38	1.85632E-36
ENSG00000110172	CHORDC1	1.04	3.72194E-38	2.34963E-36
ENSG00000110660	SLC35F2	0.99	4.49972E-38	2.83171E-36
ENSG00000006530	AGK	-1.71	4.57358E-38	2.86917E-36
ENSG00000159140	SON	0.90	4.60181E-38	2.87785E-36
ENSG00000156531	PHF6	-1.63	4.87812E-38	3.04115E-36

ENSG00000237686	AL109615.3	2.22	6.76302E-38	4.20316E-36
ENSG00000163257	DCAF16	-1.52	7.85439E-38	4.86632E-36
ENSG00000266472	MRPS21	-1.26	8.35194E-38	5.15861E-36
ENSG00000161999	JMJD8	-1.13	8.40605E-38	5.17606E-36
ENSG00000137166	FOXP4	-1.62	8.48654E-38	5.20959E-36
ENSG00000132199	ENOSF1	-1.31	9.53504E-38	5.83533E-36
ENSG00000150457	LATS2	1.54	9.72834E-38	5.93547E-36
ENSG00000105993	DNAJB6	0.89	1.05359E-37	6.40865E-36
ENSG00000166750	SLFN5	-1.79	1.10179E-37	6.68152E-36
ENSG00000103978	TMEM87A	1.15	1.30398E-37	7.88375E-36
ENSG00000165434	PGM2L1	1.98	1.8509E-37	1.11567E-35
ENSG00000088888	MAVS	-0.85	2.10826E-37	1.26698E-35
ENSG00000102265	TIMP1	1.19	2.61398E-37	1.5662E-35
ENSG00000103502	CDIPT	-1.37	2.70776E-37	1.61754E-35
ENSG00000159259	CHAF1B	-1.30	2.90356E-37	1.72935E-35
ENSG00000167680	SEMA6B	-0.98	3.06887E-37	1.82238E-35
ENSG00000229117	RPL41	-0.91	3.2476E-37	1.92281E-35
ENSG00000108106	UBE2S	0.90	3.81195E-37	2.25029E-35
ENSG00000128050	PAICS	-0.77	4.64677E-37	2.73503E-35
ENSG00000141002	TCF25	-0.91	5.58744E-37	3.27906E-35
ENSG00000103429	BFAR	-1.39	6.63054E-37	3.87984E-35
ENSG00000105329	TGFB1	1.97	1.09287E-36	6.37627E-35
ENSG00000143889	HNRNPLL	-1.74	1.10157E-36	6.40835E-35
ENSG00000107984	DKK1	1.00	2.10108E-36	1.21875E-34
ENSG00000165891	E2F7	-1.61	2.25065E-36	1.30174E-34
ENSG00000131149	GSE1	0.91	2.30144E-36	1.32727E-34
ENSG00000260917	AL158212.3	-2.37	2.6155E-36	1.50406E-34
ENSG00000273841	TAF9	-1.30	3.66613E-36	2.10219E-34
ENSG00000120265	PCMT1	0.89	3.68821E-36	2.10881E-34
ENSG00000182534	MXRA7	-1.15	4.13139E-36	2.35548E-34
ENSG00000144824	PHLDB2	-1.04	4.605E-36	2.61804E-34
ENSG00000090905	TNRC6A	1.00	8.82826E-36	5.00485E-34
ENSG00000068697	LAPTM4A	0.78	9.44411E-36	5.33886E-34
ENSG00000120438	TCP1	0.88	1.0374E-35	5.84799E-34
ENSG00000168078	PBK	0.93	1.06687E-35	5.99725E-34
ENSG00000171889	MIR31HG	3.87	1.4951E-35	8.38093E-34
ENSG00000188483	IER5L	-1.16	1.87578E-35	1.04855E-33
ENSG00000105357	MYH14	-1.03	2.65696E-35	1.48109E-33
ENSG00000115902	SLC1A4	-1.59	3.04887E-35	1.69483E-33
ENSG00000221914	PPP2R2A	0.93	3.13778E-35	1.73943E-33
ENSG00000143537	ADAM15	-0.93	3.68069E-35	2.03475E-33
ENSG00000164733	CTSB	0.81	3.77312E-35	2.0801E-33
ENSG00000165175	MID1IP1	-0.83	4.53895E-35	2.49542E-33
ENSG00000215301	DDX3X	0.91	4.77596E-35	2.61853E-33
ENSG00000102007	PLP2	-0.92	6.84926E-35	3.74501E-33
ENSG00000116688	MFN2	-0.84	7.73118E-35	4.2157E-33

ENSG00000171298	GAA	-1.08	8.65104E-35	4.70447E-33
ENSG00000163935	SFMBT1	1.39	8.76577E-35	4.75395E-33
ENSG00000239857	GET4	1.56	9.18111E-35	4.96574E-33
ENSG00000153879	CEBPG	-0.99	9.40495E-35	5.07309E-33
ENSG00000255717	SNHG1	-0.77	1.00589E-34	5.41127E-33
ENSG00000141456	PELP1	-1.13	1.04407E-34	5.60157E-33
ENSG00000228716	DHFR	-1.36	1.19107E-34	6.3732E-33
ENSG00000184254	ALDH1A3	-1.09	1.38597E-34	7.39628E-33
ENSG00000260804	LINC01963	-5.06	1.43241E-34	7.62379E-33
ENSG00000168259	DNAJC7	0.87	1.53344E-34	8.13986E-33
ENSG00000014216	CAPN1	-0.86	1.78639E-34	9.45746E-33
ENSG00000149418	ST14	0.79	2.24709E-34	1.18651E-32
ENSG00000198743	SLC5A3	-1.87	2.33191E-34	1.22806E-32
ENSG00000159216	RUNX1	-1.38	2.6891E-34	1.41245E-32
ENSG00000149218	ENDOD1	-1.30	2.73266E-34	1.43157E-32
ENSG00000164975	SNAPC3	1.20	3.09274E-34	1.61598E-32
ENSG00000171241	SHCBP1	0.94	3.25982E-34	1.69884E-32
ENSG00000114770	ABCC5	1.22	3.7597E-34	1.95426E-32
ENSG00000171130	ATP6V0E2	-1.59	3.96166E-34	2.0539E-32
ENSG00000130066	SAT1	0.77	4.04268E-34	2.0905E-32
ENSG00000204520	MICA	1.25	4.57347E-34	2.35887E-32
ENSG00000100664	EIF5	0.76	4.99629E-34	2.57033E-32
ENSG00000132591	ERAL1	-1.21	5.01625E-34	2.57398E-32
ENSG00000185262	UBALD2	1.16	7.35676E-34	3.7653E-32
ENSG00000011347	SYT7	-1.36	7.51496E-34	3.83646E-32
ENSG00000197170	PSMD12	0.83	9.68575E-34	4.93023E-32
ENSG00000145604	SKP2	-1.59	9.70674E-34	4.93023E-32
ENSG00000196792	STRN3	1.30	1.07048E-33	5.42341E-32
ENSG00000180573	H2AC6	2.14	1.16061E-33	5.86517E-32
ENSG00000108439	PNPO	-1.32	1.30336E-33	6.57E-32
ENSG00000160703	NLRX1	-1.96	1.94809E-33	9.79526E-32
ENSG00000115541	HSPE1	0.74	1.98421E-33	9.95188E-32
ENSG00000160932	LY6E	-1.09	2.23006E-33	1.1157E-31
ENSG00000171223	JUNB	0.96	3.0471E-33	1.52066E-31
ENSG00000106636	YKT6	0.76	3.55852E-33	1.77147E-31
ENSG00000159346	ADIPOR1	-0.94	4.80541E-33	2.38625E-31
ENSG00000138434	ITPRID2	0.79	5.3027E-33	2.62667E-31
ENSG00000109079	TNFAIP1	-1.48	5.41269E-33	2.67454E-31
ENSG00000079974	RABL2B	-1.86	5.88155E-33	2.89906E-31
ENSG00000142156	COL6A1	-0.93	7.19084E-33	3.5357E-31
ENSG00000126368	NR1D1	1.15	7.3662E-33	3.61305E-31
ENSG00000218283	MORF4L1P1	0.83	7.98851E-33	3.9087E-31
ENSG00000123066	MED13L	-1.10	8.54297E-33	4.1698E-31
ENSG00000074201	CLNS1A	-1.09	8.59219E-33	4.18362E-31
ENSG00000115520	COQ10B	1.30	9.1013E-33	4.42076E-31
ENSG00000092964	DPYSL2	1.04	1.02984E-32	4.99013E-31

ENSG00000173692	PSMD1	0.81	1.05974E-32	5.12259E-31
ENSG00000169188	APEX2	-1.45	1.20291E-32	5.80064E-31
ENSG00000072071	ADGRL1	-0.94	1.25454E-32	6.03506E-31
ENSG00000125952	MAX	-1.42	1.46301E-32	7.02105E-31
ENSG00000115648	MLPH	-1.25	1.83235E-32	8.77249E-31
ENSG00000142327	RNPEPL1	-1.55	1.88792E-32	9.01697E-31
ENSG00000089154	GCN1	-0.85	1.97277E-32	9.39978E-31
ENSG00000111670	GNPTAB	-1.43	2.31052E-32	1.09829E-30
ENSG00000006712	PAF1	1.04	2.32421E-32	1.10218E-30
ENSG00000011243	AKAP8L	1.05	2.81307E-32	1.33086E-30
ENSG00000146416	AIG1	-1.43	6.48447E-32	3.06055E-30
ENSG00000102781	KATNAL1	1.33	6.82715E-32	3.21471E-30
ENSG00000106348	IMPDH1	-1.06	8.5826E-32	4.03181E-30
ENSG00000198363	ASPH	0.72	8.79459E-32	4.12172E-30
ENSG00000106948	AKNA	-1.54	9.06091E-32	4.23661E-30
ENSG00000178537	SLC25A20	1.57	1.02413E-31	4.77738E-30
ENSG00000070081	NUCB2	1.15	1.10449E-31	5.14024E-30
ENSG00000144524	COPS7B	-1.22	1.89345E-31	8.79156E-30
ENSG00000233270	SNRPEP4	-2.55	2.10326E-31	9.74316E-30
ENSG00000117360	PRPF3	0.98	2.21121E-31	1.02196E-29
ENSG00000134013	LOXL2	-1.44	2.70184E-31	1.24583E-29
ENSG00000166508	MCM7	-0.71	3.17799E-31	1.46202E-29
ENSG00000164292	RHOBTB3	-0.92	3.26967E-31	1.50075E-29
ENSG00000143401	ANP32E	0.82	3.60846E-31	1.65246E-29
ENSG00000103381	CPPED1	1.63	3.77544E-31	1.72498E-29
ENSG00000121073	SLC35B1	0.85	4.08213E-31	1.86085E-29
ENSG00000175063	UBE2C	0.86	4.33597E-31	1.97208E-29
ENSG00000116698	SMG7	-0.99	4.95502E-31	2.24852E-29
ENSG00000182979	MTA1	-0.90	5.667E-31	2.56579E-29
ENSG00000165802	NSMF	-1.21	5.96344E-31	2.69391E-29
ENSG00000128973	CLN6	-1.58	6.5447E-31	2.94983E-29
ENSG00000172500	FIBP	-1.15	6.9545E-31	3.12749E-29
ENSG00000116985	BMP8B	-1.88	7.96908E-31	3.57572E-29
ENSG00000164070	HSPA4L	1.36	8.59881E-31	3.84965E-29
ENSG00000105341	DMAC2	-1.20	9.58566E-31	4.28188E-29
ENSG00000110107	PRPF19	-0.87	9.68426E-31	4.31629E-29
ENSG00000120833	SOCS2	1.44	9.72446E-31	4.32457E-29
ENSG00000185787	MORF4L1	0.77	9.89236E-31	4.38949E-29
ENSG00000115738	ID2	-1.17	1.07947E-30	4.77929E-29
ENSG00000171988	JMJD1C	1.19	1.08609E-30	4.79796E-29
ENSG00000160949	TONSL	-0.97	1.33604E-30	5.88916E-29
ENSG00000196961	AP2A1	-0.96	1.44193E-30	6.34195E-29
ENSG00000138758	SEPTIN11	-0.98	1.48256E-30	6.50634E-29
ENSG00000223768	LINC00205	-1.88	1.5085E-30	6.60571E-29
ENSG00000136997	MYC	-0.88	1.57336E-30	6.87469E-29
ENSG00000108424	KPNB1	0.67	1.64627E-30	7.1776E-29

ENSG00000179091	CYC1	-0.83	2.06311E-30	8.97542E-29
ENSG00000179046	TRIML2	1.76	2.06999E-30	8.98581E-29
ENSG00000185361	TNFAIP8L1	1.12	2.28459E-30	9.89595E-29
ENSG00000102178	UBL4A	-1.26	2.33978E-30	1.01131E-28
ENSG00000172992	DCAKD	-2.13	2.50009E-30	1.07827E-28
ENSG00000008130	NADK	-0.99	2.55597E-30	1.1E-28
ENSG00000105726	ATP13A1	-1.19	2.63454E-30	1.13138E-28
ENSG00000139318	DUSP6	-1.01	3.19664E-30	1.36983E-28
ENSG00000160310	PRMT2	0.94	3.23115E-30	1.38166E-28
ENSG00000106868	SUSD1	1.76	3.35853E-30	1.43307E-28
ENSG00000137106	GRHPR	-0.99	3.53042E-30	1.50191E-28
ENSG00000169398	PTK2	-0.94	3.53489E-30	1.50191E-28
ENSG00000035403	VCL	-0.90	3.82737E-30	1.62274E-28
ENSG00000102034	ELF4	-1.45	3.94817E-30	1.67042E-28
ENSG00000149269	PAK1	-1.19	4.09968E-30	1.73086E-28
ENSG00000166900	STX3	0.88	4.49745E-30	1.8948E-28
ENSG00000078140	UBE2K	-0.89	5.35061E-30	2.24951E-28
ENSG00000134107	BHLHE40	1.95	5.56673E-30	2.33546E-28
ENSG00000197702	PARVA	-1.49	5.81594E-30	2.43491E-28
ENSG00000161714	PLCD3	-1.02	5.95566E-30	2.4882E-28
ENSG00000253719	ATXN7L3B	-0.88	6.16885E-30	2.57189E-28
ENSG00000272391	POM121C	-1.02	6.85415E-30	2.85167E-28
ENSG00000143294	PRCC	-1.11	8.34707E-30	3.46559E-28
ENSG00000089006	SNX5	-0.86	9.49169E-30	3.93267E-28
ENSG00000162736	NCSTN	-1.06	1.03258E-29	4.2694E-28
ENSG00000100304	TTLL12	-0.87	1.06122E-29	4.37378E-28
ENSG00000136295	TTYH3	-1.10	1.06219E-29	4.37378E-28
ENSG00000114019	AMOTL2	-0.98	1.06542E-29	4.37807E-28
ENSG00000197989	SNHG12	-1.23	1.14972E-29	4.71481E-28
ENSG00000135916	ITM2C	0.77	1.20197E-29	4.91899E-28
ENSG00000169136	ATF5	-1.32	1.27854E-29	5.22167E-28
ENSG00000116161	CACYBP	0.78	1.60395E-29	6.53732E-28
ENSG00000135451	TROAP	0.96	1.60862E-29	6.54303E-28
ENSG00000166803	PCLAF	-1.11	1.63256E-29	6.62692E-28
ENSG00000083312	TNPO1	0.84	1.71209E-29	6.9357E-28
ENSG00000117632	STMN1	0.69	1.78884E-29	7.23198E-28
ENSG00000169180	XPO6	-0.78	2.01579E-29	8.13305E-28
ENSG00000164520	RAET1E	2.64	2.26313E-29	9.11262E-28
ENSG00000087111	PIGS	-1.11	2.32262E-29	9.3334E-28
ENSG00000167880	EVPL	0.96	2.58964E-29	1.03856E-27
ENSG00000150722	PPP1R1C	1.78	2.71708E-29	1.08748E-27
ENSG00000144560	VGLL4	1.42	2.75117E-29	1.09893E-27
ENSG00000143702	CEP170	1.27	2.95287E-29	1.17715E-27
ENSG00000085231	AK6	1.54	3.30795E-29	1.31608E-27
ENSG00000136542	GALNT5	1.92	3.48774E-29	1.38485E-27
ENSG00000164949	GEM	1.80	5.74013E-29	2.27468E-27

ENSG00000130270	ATP8B3	1.35	5.99326E-29	2.3703E-27
ENSG00000183963	SMTN	0.81	6.41954E-29	2.53388E-27
ENSG00000172292	CERS6	1.13	6.92027E-29	2.72615E-27
ENSG00000187741	FANCA	-1.04	7.37379E-29	2.8991E-27
ENSG00000184743	ATL3	0.87	7.39689E-29	2.90248E-27
ENSG00000173113	TRMT112	0.85	8.59851E-29	3.36739E-27
ENSG00000111145	ELK3	1.07	9.23541E-29	3.60975E-27
ENSG00000100591	AHSA1	0.79	9.5851E-29	3.73912E-27
ENSG00000177565	TBL1XR1	0.85	9.63586E-29	3.75161E-27
ENSG00000214517	PPME1	0.85	1.32292E-28	5.14062E-27
ENSG00000136830	NIBAN2	-0.72	1.38518E-28	5.37213E-27
ENSG00000186815	TPCN1	-1.62	1.47091E-28	5.69359E-27
ENSG00000131368	MRPS25	-1.38	1.53082E-28	5.907E-27
ENSG00000137575	SDCBP	0.78	1.53195E-28	5.907E-27
ENSG00000175826	CTDNEP1	-1.01	1.60162E-28	6.16379E-27
ENSG00000198663	C6orf89	-1.00	1.64286E-28	6.31036E-27
ENSG00000156639	ZFAND3	0.92	1.67645E-28	6.42704E-27
ENSG00000136930	PSMB7	0.73	1.68625E-28	6.45226E-27
ENSG00000134802	SLC43A3	-1.64	1.83912E-28	7.02376E-27
ENSG00000170190	SLC16A5	-1.22	1.97843E-28	7.5414E-27
ENSG00000100994	PYGB	-0.76	2.32291E-28	8.83765E-27
ENSG00000287064	AL606500.1	2.95	2.43534E-28	9.24784E-27
ENSG00000165283	STOML2	-0.91	2.4952E-28	9.4572E-27
ENSG00000105486	LIG1	-0.92	2.57622E-28	9.74579E-27
ENSG00000099284	MACROH2A2	-1.09	2.61334E-28	9.86756E-27
ENSG00000104964	TLE5	0.81	2.99444E-28	1.12853E-26
ENSG00000160410	SHKBP1	-1.05	3.21912E-28	1.21092E-26
ENSG00000135046	ANXA1	0.66	3.83189E-28	1.43872E-26
ENSG00000165916	PSMC3	0.68	3.94146E-28	1.47709E-26
ENSG00000142687	KIAA0319L	-1.19	5.33353E-28	1.99504E-26
ENSG00000101104	PABPC1L	-1.03	6.46946E-28	2.41542E-26
ENSG00000189339	SLC35E2B	-1.60	7.16904E-28	2.67164E-26
ENSG00000071539	TRIP13	-1.17	7.62226E-28	2.83526E-26
ENSG00000116221	MRPL37	-0.93	7.91028E-28	2.93693E-26
ENSG00000173546	CSPG4	0.91	8.59441E-28	3.1839E-26
ENSG00000012048	BRCA1	-1.28	8.60728E-28	3.1839E-26
ENSG00000116584	ARHGEF2	-0.78	8.65429E-28	3.19538E-26
ENSG00000162065	TBC1D24	-1.54	9.00339E-28	3.31816E-26
ENSG00000124466	LYPD3	2.31	1.08247E-27	3.97651E-26
ENSG00000110218	PANX1	1.38	1.08305E-27	3.97651E-26
ENSG00000077380	DYNC1I2	1.13	1.08494E-27	3.97651E-26
ENSG00000134153	EMC7	1.04	1.14052E-27	4.17259E-26
ENSG00000058085	LAMC2	1.08	1.23348E-27	4.50447E-26
ENSG00000166924	NYAP1	1.78	1.27697E-27	4.65479E-26
ENSG00000126088	UROD	-1.09	1.37355E-27	4.99773E-26
ENSG00000125304	TM9SF2	0.70	1.3847E-27	5.02915E-26

ENSG00000130193	THEM6	-1.66	1.52486E-27	5.52816E-26
ENSG00000105364	MRPL4	-0.91	1.53929E-27	5.57038E-26
ENSG00000091436	MAP3K20	1.00	1.80195E-27	6.50913E-26
ENSG00000283498	MIR1244-2	-1.05	1.87881E-27	6.77456E-26
ENSG00000119689	DLST	-0.79	1.90956E-27	6.87304E-26
ENSG00000132535	DLG4	1.57	1.94576E-27	6.99075E-26
ENSG00000105810	CDK6	-1.86	1.96148E-27	7.03461E-26
ENSG00000163297	ANTXR2	2.20	2.23799E-27	8.01191E-26
ENSG00000126934	MAP2K2	-0.95	2.28407E-27	8.16227E-26
ENSG00000198793	MTOR	-0.90	2.39807E-27	8.55441E-26
ENSG00000143420	ENSA	0.71	2.634E-27	9.37927E-26
ENSG00000160877	NACC1	-0.73	2.66981E-27	9.48993E-26
ENSG00000183741	CBX6	-0.71	2.6876E-27	9.53622E-26
ENSG00000145920	CPLX2	3.96	2.72519E-27	9.65248E-26
ENSG00000142541	RPL13A	-0.67	2.95198E-27	1.04373E-25
ENSG00000090520	DNAJB11	0.77	3.002E-27	1.05954E-25
ENSG00000176393	RNPEP	-0.87	3.77792E-27	1.33105E-25
ENSG00000256269	HMBS	-1.03	3.94498E-27	1.38747E-25
ENSG00000143013	LMO4	-1.24	4.77555E-27	1.67664E-25
ENSG00000119042	SATB2	1.49	5.73984E-27	2.01166E-25
ENSG00000162344	FGF19	-0.97	5.88087E-27	2.05748E-25
ENSG00000109084	TMEM97	-1.10	6.198E-27	2.16465E-25
ENSG00000261373	VPS9D1-AS1	-1.54	6.56246E-27	2.28794E-25
ENSG00000102098	SCML2	1.60	7.11033E-27	2.47464E-25
ENSG00000104413	ESRP1	-0.77	7.90314E-27	2.74579E-25
ENSG00000179941	BBS10	-1.49	7.97264E-27	2.76514E-25
ENSG00000135862	LAMC1	0.70	8.48426E-27	2.93749E-25
ENSG00000123485	HJURP	0.93	8.59877E-27	2.972E-25
ENSG00000160957	RECQL4	-0.78	9.47839E-27	3.27037E-25
ENSG00000142599	RERE	-1.36	9.55692E-27	3.29179E-25
ENSG00000167470	MIDN	0.74	1.18134E-26	4.06203E-25
ENSG00000142765	SYTL1	-1.17	1.19326E-26	4.09598E-25
ENSG00000186174	BCL9L	-0.91	1.27004E-26	4.35207E-25
ENSG00000132963	POMP	0.74	1.48453E-26	5.07836E-25
ENSG00000134970	TMED7	-0.90	1.6406E-26	5.60268E-25
ENSG00000183010	PYCR1	-0.74	1.67028E-26	5.69433E-25
ENSG00000050405	LIMA1	0.83	1.71669E-26	5.84257E-25
ENSG00000054967	RELT	1.33	1.76787E-26	6.00656E-25
ENSG00000089820	ARHGAP4	-1.23	1.84488E-26	6.2576E-25
ENSG00000104756	KCTD9	1.00	1.89951E-26	6.432E-25
ENSG00000183508	TENT5C	2.47	1.95275E-26	6.60109E-25
ENSG00000124171	PARD6B	1.33	2.14572E-26	7.24116E-25
ENSG00000168288	MMADHC	0.71	2.28568E-26	7.70051E-25
ENSG00000138111	MFSD13A	-1.39	2.58857E-26	8.70629E-25
ENSG00000104969	SGTA	-0.78	2.59571E-26	8.71567E-25
ENSG00000161179	YDJC	-1.21	2.68994E-26	9.01692E-25

ENSG00000072518	MARK2	-1.08	3.19695E-26	1.06986E-24
ENSG00000135506	OS9	-0.79	4.11869E-26	1.37601E-24
ENSG00000288066	AC097448.1	-2.72	4.34567E-26	1.44943E-24
ENSG00000139112	GABARAPL1	0.90	4.66975E-26	1.55493E-24
ENSG00000159423	ALDH4A1	-1.52	4.71789E-26	1.56835E-24
ENSG00000026025	VIM	1.58	4.73874E-26	1.57267E-24
ENSG00000258441	LINC00641	-1.71	5.08239E-26	1.68392E-24
ENSG00000182628	SKA2	0.78	5.38197E-26	1.78023E-24
ENSG00000181929	PRKAG1	-1.02	6.08453E-26	2.0093E-24
ENSG00000170348	TMED10	-0.65	6.21892E-26	2.0503E-24
ENSG00000108578	BLMH	0.86	6.85388E-26	2.25592E-24
ENSG00000170759	KIF5B	0.67	6.88724E-26	2.26318E-24
ENSG00000175727	MLXIP	-0.96	7.47487E-26	2.44943E-24
ENSG00000162512	SDC3	-1.07	7.47852E-26	2.44943E-24
ENSG00000274750	H3C6	3.04	9.34287E-26	3.05506E-24
ENSG00000177302	TOP3A	-1.15	9.69088E-26	3.16369E-24
ENSG00000178381	ZFAND2A	1.54	1.06043E-25	3.45626E-24
ENSG00000112984	KIF20A	0.82	1.07399E-25	3.49475E-24
ENSG00000113369	ARRDC3	1.32	1.10973E-25	3.60518E-24
ENSG00000167930	FAM234A	-1.50	1.12343E-25	3.64378E-24
ENSG00000074696	HACD3	0.85	1.508E-25	4.8832E-24
ENSG00000106367	AP1S1	0.77	1.53478E-25	4.96187E-24
ENSG00000148290	SURF1	-1.04	1.55283E-25	5.01212E-24
ENSG00000143369	ECM1	2.52	1.63762E-25	5.2773E-24
ENSG00000163507	CIP2A	1.09	1.65036E-25	5.30982E-24
ENSG00000067798	NAV3	1.73	1.72074E-25	5.52736E-24
ENSG00000148187	MRRF	-1.03	1.7281E-25	5.5421E-24
ENSG00000215492	HNRNPA1P7	-0.82	2.0596E-25	6.59467E-24
ENSG00000110090	CPT1A	0.80	2.17989E-25	6.96868E-24
ENSG00000196576	PLXNB2	-0.64	2.33836E-25	7.46335E-24
ENSG00000065970	FOXJ2	-1.17	2.36639E-25	7.54079E-24
ENSG00000075415	SLC25A3	-0.70	2.41546E-25	7.68494E-24
ENSG00000072135	PTPN18	-1.33	2.56726E-25	8.15493E-24
ENSG00000129250	KIF1C	0.63	2.65457E-25	8.40596E-24
ENSG00000226752	CUTALP	-1.97	2.65469E-25	8.40596E-24
ENSG00000138107	ACTR1A	0.70	2.90638E-25	9.18839E-24
ENSG00000103356	EARS2	0.91	3.10242E-25	9.79268E-24
ENSG00000101255	TRIB3	-0.75	3.24238E-25	1.02184E-23
ENSG00000205336	ADGRG1	-1.09	3.51306E-25	1.1054E-23
ENSG00000170860	LSM3	0.93	3.52035E-25	1.10595E-23
ENSG00000179750	APOBEC3B	1.38	3.6561E-25	1.1468E-23
ENSG00000067955	CBFB	0.70	3.98696E-25	1.24862E-23
ENSG00000168758	SEMA4C	1.12	4.06202E-25	1.27014E-23
ENSG00000166598	HSP90B1	0.60	4.39214E-25	1.37123E-23
ENSG00000167258	CDK12	0.85	4.45692E-25	1.38928E-23
ENSG00000169100	SLC25A6	-0.74	4.48309E-25	1.39527E-23

ENSG00000178719	GRINA	-0.96	4.55989E-25	1.41696E-23
ENSG00000143164	DCAF6	0.98	4.88728E-25	1.51635E-23
ENSG00000077150	NFKB2	0.76	5.03332E-25	1.55924E-23
ENSG00000111640	GAPDH	-0.91	5.4488E-25	1.68534E-23
ENSG00000101412	E2F1	-1.09	6.83617E-25	2.11119E-23
ENSG00000175906	ARL4D	1.49	7.79806E-25	2.40454E-23
ENSG00000135828	RNASEL	2.15	8.47554E-25	2.60942E-23
ENSG00000176273	SLC35G1	-2.11	9.03994E-25	2.77891E-23
ENSG00000196313	POM121	-1.06	9.18135E-25	2.81806E-23
ENSG00000119969	HELLS	-0.84	9.72204E-25	2.97846E-23
ENSG00000129195	PIMREG	0.95	9.73373E-25	2.97846E-23
ENSG00000278535	DHRS11	-1.79	1.03391E-24	3.15888E-23
ENSG00000144867	SRPRB	0.79	1.1244E-24	3.43011E-23
ENSG00000100296	THOC5	0.84	1.26055E-24	3.83959E-23
ENSG00000129925	PGAP6	-1.42	1.27278E-24	3.87096E-23
ENSG00000160193	WDR4	1.15	1.41934E-24	4.31013E-23
ENSG00000141298	SSH2	1.04	1.47285E-24	4.46585E-23
ENSG00000113719	ERGIC1	-0.80	1.49416E-24	4.52361E-23
ENSG00000160789	LMNA	0.61	1.5074E-24	4.5568E-23
ENSG00000242125	SNHG3	-0.83	1.51177E-24	4.56144E-23
ENSG00000178209	PLEC	-0.63	1.51349E-24	4.56144E-23
ENSG00000183684	ALYREF	0.67	1.52692E-24	4.595E-23
ENSG00000181192	DHTKD1	-1.58	1.56391E-24	4.69925E-23
ENSG00000143384	MCL1	0.68	1.61092E-24	4.83324E-23
ENSG00000166436	TRIM66	-1.61	1.82605E-24	5.47049E-23
ENSG00000100823	APEX1	-0.67	1.84629E-24	5.52288E-23
ENSG00000111678	C12orf57	0.93	1.89901E-24	5.67207E-23
ENSG00000103479	RBL2	-1.66	1.94683E-24	5.80625E-23
ENSG00000166197	NOLC1	-0.63	1.9986E-24	5.95179E-23
ENSG00000170004	CHD3	0.72	2.0118E-24	5.9822E-23
ENSG00000140105	WARS1	-0.76	2.20539E-24	6.54811E-23
ENSG00000167695	TLCD3A	-1.26	2.246E-24	6.65881E-23
ENSG00000147416	ATP6V1B2	-0.93	2.27207E-24	6.72614E-23
ENSG00000140474	ULK3	-1.05	2.37776E-24	7.02861E-23
ENSG00000152291	TGOLN2	-0.86	2.44206E-24	7.20803E-23
ENSG00000105649	RAB3A	1.87	2.55541E-24	7.53149E-23
ENSG00000250899	AC125807.2	-2.90	2.64919E-24	7.79641E-23
ENSG00000107864	CPEB3	2.03	2.79315E-24	8.20801E-23
ENSG00000072958	AP1M1	-1.09	2.87943E-24	8.44915E-23
ENSG00000168461	RAB31	0.93	3.52577E-24	1.03306E-22
ENSG00000118689	FOXO3	-1.09	3.82814E-24	1.12001E-22
ENSG00000265808	SEC22B	0.93	3.83945E-24	1.12168E-22
ENSG00000065154	OAT	0.70	3.88512E-24	1.13337E-22
ENSG00000186106	ANKRD46	1.47	3.96645E-24	1.15541E-22
ENSG00000189334	S100A14	-0.86	4.163E-24	1.2109E-22
ENSG00000105655	ISYNA1	-0.75	4.33138E-24	1.25805E-22

ENSG00000173473	SMARCC1	-0.68	4.43068E-24	1.28503E-22
ENSG00000221926	TRIM16	1.03	5.49898E-24	1.59255E-22
ENSG00000087502	ERGIC2	1.04	5.876E-24	1.69928E-22
ENSG00000100242	SUN2	0.69	6.22173E-24	1.79667E-22
ENSG00000053371	AKR7A2	-0.91	7.56294E-24	2.18083E-22
ENSG00000042286	AIFM2	0.91	8.36514E-24	2.40868E-22
ENSG00000188706	ZDHHC9	-1.37	8.48111E-24	2.43856E-22
ENSG00000178971	CTC1	-1.03	9.51008E-24	2.7305E-22
ENSG00000091164	TXNL1	0.78	1.20207E-23	3.44639E-22
ENSG00000094916	CBX5	-0.71	1.25776E-23	3.60089E-22
ENSG00000130508	PXDN	1.18	1.25977E-23	3.60151E-22
ENSG00000117650	NEK2	0.99	1.33272E-23	3.80461E-22
ENSG00000010438	PRSS3	1.11	1.38717E-23	3.95444E-22
ENSG00000272636	DOC2B	-1.29	1.44947E-23	4.12616E-22
ENSG00000120337	TNFSF18	-3.92	1.58893E-23	4.51671E-22
ENSG00000186432	KPNA4	0.84	1.61331E-23	4.5795E-22
ENSG00000120549	KIAA1217	1.58	1.82237E-23	5.16561E-22
ENSG00000110422	HIPK3	0.91	1.88896E-23	5.34211E-22
ENSG00000145050	MANF	0.77	1.88997E-23	5.34211E-22
ENSG00000143179	UCK2	-0.81	1.90902E-23	5.38834E-22
ENSG00000197226	TBC1D9B	-0.75	1.99352E-23	5.61891E-22
ENSG00000104783	KCNN4	-1.18	2.04854E-23	5.76589E-22
ENSG00000174989	FBXW8	-1.79	2.11995E-23	5.95849E-22
ENSG00000175550	DRAP1	0.70	2.12814E-23	5.97312E-22
ENSG00000134779	TPGS2	-0.86	2.19823E-23	6.16119E-22
ENSG00000179454	KLHL28	1.59	2.41437E-23	6.75755E-22
ENSG00000169057	MECP2	-0.98	2.45424E-23	6.85953E-22
ENSG00000113552	GNPDA1	0.80	2.58266E-23	7.2084E-22
ENSG00000167747	C19orf48	-0.78	2.59296E-23	7.22707E-22
ENSG00000157978	LDLRAP1	-1.28	2.96972E-23	8.26566E-22
ENSG00000160570	DEDD2	1.05	3.10274E-23	8.62388E-22
ENSG00000186350	RXRA	-0.93	3.19776E-23	8.87568E-22
ENSG00000176490	DIRAS1	-1.18	3.51337E-23	9.73817E-22
ENSG00000125901	MRPS26	-1.08	3.63537E-23	1.00624E-21
ENSG00000178802	MPI	-1.13	3.73148E-23	1.03141E-21
ENSG00000187522	HSPA14	1.21	3.74309E-23	1.0332E-21
ENSG00000101856	PGRMC1	-1.00	4.19731E-23	1.15698E-21
ENSG00000148925	BTBD10	1.11	4.20535E-23	1.1576E-21
ENSG00000140365	COMMD4	-1.04	4.7373E-23	1.30224E-21
ENSG00000213722	DDAH2	0.99	5.76437E-23	1.5824E-21
ENSG00000139354	GAS2L3	1.14	6.88232E-23	1.8867E-21
ENSG00000089280	FUS	-0.59	8.17084E-23	2.23686E-21
ENSG00000188290	HES4	-1.20	8.20198E-23	2.24232E-21
ENSG00000168701	TMEM208	1.02	8.46833E-23	2.31198E-21
ENSG00000110321	EIF4G2	0.57	8.48342E-23	2.31295E-21
ENSG00000116489	CAPZA1	0.69	8.67756E-23	2.36266E-21

ENSG00000197343	ZNF655	-1.08	8.74656E-23	2.37821E-21
ENSG00000174684	B4GAT1	1.52	9.00462E-23	2.44505E-21
ENSG00000135912	TTLL4	-1.19	9.27534E-23	2.51515E-21
ENSG00000062725	APPBP2	0.92	9.70037E-23	2.62684E-21
ENSG00000172009	THOP1	-0.77	1.00124E-22	2.70769E-21
ENSG00000141569	TRIM65	-1.07	1.00504E-22	2.7143E-21
ENSG00000168411	RFWD3	0.71	1.02748E-22	2.77114E-21
ENSG00000116977	LGALS8	1.21	1.25107E-22	3.36521E-21
ENSG00000011422	PLAUR	1.04	1.25111E-22	3.36521E-21
ENSG00000136108	CKAP2	0.81	1.28371E-22	3.44827E-21
ENSG00000040633	PHF23	0.83	1.35931E-22	3.64646E-21
ENSG00000084463	WBP11	0.66	1.40356E-22	3.7601E-21
ENSG00000198171	DDRGK1	1.10	1.48245E-22	3.96616E-21
ENSG00000168056	LTBP3	-0.71	1.56133E-22	4.17162E-21
ENSG00000146842	TMEM209	-1.26	1.58788E-22	4.23688E-21
ENSG00000143476	DTL	-1.13	1.64226E-22	4.37615E-21
ENSG00000172819	RARG	-1.10	1.88003E-22	5.00307E-21
ENSG00000197019	SERTAD1	1.07	2.11441E-22	5.61934E-21
ENSG00000133703	KRAS	-0.96	2.23113E-22	5.92166E-21
ENSG00000023445	BIRC3	1.71	2.37515E-22	6.29556E-21
ENSG00000134508	CABLES1	-2.12	2.46046E-22	6.51306E-21
ENSG00000132846	ZBED3	-1.60	2.82756E-22	7.47491E-21
ENSG00000170175	CHRNB1	1.16	3.00676E-22	7.93815E-21
ENSG00000104695	PPP2CB	0.69	3.39863E-22	8.96093E-21
ENSG00000117500	TMED5	0.89	3.47906E-22	9.16092E-21
ENSG00000107175	CREB3	0.94	3.54324E-22	9.31765E-21
ENSG00000104219	ZDHHC2	1.02	3.59781E-22	9.44873E-21
ENSG00000134809	TIMM10	0.91	4.0183E-22	1.05392E-20
ENSG00000170627	GTSF1	-0.72	4.25084E-22	1.11345E-20
ENSG00000156510	HKDC1	-2.23	4.92767E-22	1.28905E-20
ENSG00000136731	UGGT1	-0.80	4.99434E-22	1.30479E-20
ENSG00000281376	ABALON	-1.65	5.19217E-22	1.35409E-20
ENSG00000162636	FAM102B	1.05	5.1966E-22	1.35409E-20
ENSG00000163535	SGO2	1.09	5.38494E-22	1.40135E-20
ENSG00000170315	UBB	0.62	5.86151E-22	1.52338E-20
ENSG00000132254	ARFIP2	-1.06	6.00013E-22	1.55739E-20
ENSG00000155100	OTUD6B	-1.31	7.75083E-22	2.00919E-20
ENSG00000150753	CCT5	0.57	7.85395E-22	2.03329E-20
ENSG00000114745	GORASP1	-1.30	8.5877E-22	2.22038E-20
ENSG00000165806	CASP7	0.85	9.20056E-22	2.37576E-20
ENSG00000117411	B4GALT2	-1.00	9.23397E-22	2.38132E-20
ENSG00000089053	ANAPC5	-0.75	1.05212E-21	2.70978E-20
ENSG00000167658	EEF2	-0.64	1.06181E-21	2.73123E-20
ENSG00000105568	PPP2R1A	-0.71	1.07031E-21	2.74955E-20
ENSG00000111602	TIMELESS	-0.74	1.08373E-21	2.78045E-20
ENSG00000122565	CBX3	0.60	1.09406E-21	2.80338E-20

ENSG00000137094	DNAJB5	1.47	1.1161E-21	2.85618E-20
ENSG00000178773	CPNE7	-0.80	1.15805E-21	2.95976E-20
ENSG00000105755	ETHE1	-1.20	1.17149E-21	2.99028E-20
ENSG00000242498	ARPIN	-1.38	1.25147E-21	3.19038E-20
ENSG00000135404	CD63	0.67	1.28408E-21	3.26934E-20
ENSG00000167264	DUS2	-1.58	1.31068E-21	3.33283E-20
ENSG00000125944	HNRNPR	-0.63	1.35009E-21	3.42868E-20
ENSG00000170606	HSPA4	0.61	1.35275E-21	3.43109E-20
ENSG00000042753	AP2S1	0.73	1.44407E-21	3.65808E-20
ENSG00000170689	HOXB9	-1.01	1.44624E-21	3.65893E-20
ENSG00000169727	GPS1	-0.76	1.55204E-21	3.92164E-20
ENSG00000104738	MCM4	-0.71	1.57228E-21	3.96777E-20
ENSG00000234741	GAS5	-0.62	1.61058E-21	4.05932E-20
ENSG00000061794	MRPS35	-0.89	1.6573E-21	4.17182E-20
ENSG00000257218	GATC	1.08	1.82743E-21	4.59428E-20
ENSG00000110906	KCTD10	0.79	1.86322E-21	4.67838E-20
ENSG00000101361	NOP56	-0.60	1.90917E-21	4.78776E-20
ENSG00000100983	GSS	-0.78	1.97452E-21	4.94544E-20
ENSG00000051128	HOMER3	1.00	2.09782E-21	5.24768E-20
ENSG00000123975	CKS2	0.65	2.24716E-21	5.61424E-20
ENSG00000089057	SLC23A2	-1.29	2.33189E-21	5.81868E-20
ENSG00000213085	CFAP45	3.69	2.35083E-21	5.85862E-20
ENSG00000170832	USP32	0.86	2.51434E-21	6.25834E-20
ENSG00000116128	BCL9	-1.24	2.64374E-21	6.57224E-20
ENSG00000149357	LAMTOR1	-1.08	2.73569E-21	6.79238E-20
ENSG00000166887	VPS39	-1.11	2.77785E-21	6.88852E-20
ENSG00000102804	TSC22D1	-0.57	2.81066E-21	6.96126E-20
ENSG00000123080	CDKN2C	1.14	3.20413E-21	7.92061E-20
ENSG00000104852	SNRNP70	0.63	3.20593E-21	7.92061E-20
ENSG00000127955	GNAI1	0.73	3.27881E-21	8.09071E-20
ENSG00000160208	RRP1B	-0.83	3.28813E-21	8.1037E-20
ENSG00000163468	CCT3	0.55	3.3011E-21	8.12565E-20
ENSG00000170445	HARS1	0.68	3.36603E-21	8.2753E-20
ENSG00000197780	TAF13	1.04	3.41828E-21	8.39344E-20
ENSG00000138095	LRPPRC	-0.63	3.44801E-21	8.45609E-20
ENSG00000106628	POLD2	-0.73	3.71243E-21	9.09342E-20
ENSG00000196517	SLC6A9	-1.45	4.09928E-21	1.0025E-19
ENSG00000012232	EXTL3	-0.76	4.10277E-21	1.0025E-19
ENSG00000183864	TOB2	-0.94	4.31498E-21	1.05306E-19
ENSG00000169992	NLGN2	-0.91	4.63211E-21	1.12908E-19
ENSG00000128335	APOL2	1.41	5.00887E-21	1.21944E-19
ENSG00000105438	KDELR1	0.64	5.43597E-21	1.32032E-19
ENSG00000100596	SPTLC2	-0.85	5.43644E-21	1.32032E-19
ENSG00000105281	SLC1A5	-0.64	5.52182E-21	1.33943E-19
ENSG00000132612	VPS4A	-0.89	6.61138E-21	1.60178E-19
ENSG00000164904	ALDH7A1	-0.81	6.88438E-21	1.6659E-19

ENSG00000158796	DEDD	-1.23	7.70827E-21	1.86302E-19
ENSG00000116001	TIA1	-1.03	7.84821E-21	1.89455E-19
ENSG00000105245	NUMBL	-1.27	7.86191E-21	1.89557E-19
ENSG00000182093	GET1	-1.52	7.90884E-21	1.90459E-19
ENSG00000094975	SUCO	0.92	8.33376E-21	2.00451E-19
ENSG00000122515	ZMIZ2	-0.88	8.5899E-21	2.06364E-19
ENSG00000136444	RSAD1	-0.86	8.86E-21	2.12597E-19
ENSG00000196839	ADA	-1.59	9.74674E-21	2.33595E-19
ENSG00000055332	EIF2AK2	0.79	1.11861E-20	2.6777E-19
ENSG00000101000	PROCR	0.89	1.14154E-20	2.72933E-19
ENSG00000111358	GTF2H3	-1.00	1.14428E-20	2.73261E-19
ENSG00000143033	MTF2	1.01	1.14808E-20	2.73842E-19
ENSG00000113360	DROSHA	-1.02	1.15059E-20	2.74113E-19
ENSG00000088367	EPB41L1	0.95	1.18855E-20	2.82821E-19
ENSG00000111843	TMEM14C	-0.87	1.19616E-20	2.84293E-19
ENSG00000131503	ANKHD1	1.62	1.22852E-20	2.91639E-19
ENSG00000137843	PAK6	-1.50	1.23323E-20	2.9241E-19
ENSG00000162337	LRP5	-0.85	1.2692E-20	3.00583E-19
ENSG00000120685	PROSER1	-1.00	1.2854E-20	3.0406E-19
ENSG00000184992	BRI3BP	-0.80	1.28766E-20	3.04234E-19
ENSG00000125505	MBOAT7	-0.80	1.34341E-20	3.17033E-19
ENSG00000164104	HMGB2	0.57	1.34589E-20	3.17243E-19
ENSG00000151148	UBE3B	-1.10	1.37133E-20	3.22859E-19
ENSG00000250920	AC105460.1	-1.37	1.42968E-20	3.36203E-19
ENSG00000082781	ITGB5	0.71	1.47076E-20	3.45455E-19
ENSG00000131652	THOC6	-0.93	1.47911E-20	3.47011E-19
ENSG00000198900	TOP1	0.57	1.56786E-20	3.67401E-19
ENSG00000228782	MRPL45P2	1.65	1.57805E-20	3.69357E-19
ENSG00000159086	PAXBP1	-0.96	1.59704E-20	3.73365E-19
ENSG00000126254	RBM42	0.74	1.81276E-20	4.23301E-19
ENSG00000154237	LRRK1	-1.59	1.84167E-20	4.2955E-19
ENSG00000115484	CCT4	0.53	1.86531E-20	4.34558E-19
ENSG00000139722	VPS37B	0.71	2.07798E-20	4.83541E-19
ENSG00000100883	SRP54	0.78	2.08108E-20	4.837E-19
ENSG00000104689	TNFRSF10A	0.98	2.08718E-20	4.84555E-19
ENSG00000182944	EWSR1	-0.54	2.12192E-20	4.9205E-19
ENSG00000164309	CMYA5	3.12	2.31706E-20	5.36678E-19
ENSG00000110917	MLEC	-0.58	2.41901E-20	5.59644E-19
ENSG00000073614	KDM5A	0.98	2.5611E-20	5.91833E-19
ENSG00000073605	GSDMB	-1.23	2.71049E-20	6.25631E-19
ENSG00000116030	SUMO1	-0.66	2.78385E-20	6.41824E-19
ENSG00000077782	FGFR1	-0.92	2.80595E-20	6.46177E-19
ENSG00000099904	ZDHHC8	-1.04	2.93183E-20	6.74388E-19
ENSG00000136273	HUS1	1.35	3.01528E-20	6.92788E-19
ENSG00000087085	ACHE	2.58	3.16658E-20	7.26715E-19
ENSG00000006327	TNFRSF12A	0.71	3.1754E-20	7.27905E-19

ENSG00000164649	CDCA7L	-1.09	3.46312E-20	7.92951E-19
ENSG00000159363	ATP13A2	-0.82	3.63394E-20	8.31114E-19
ENSG00000127585	FBXL16	-1.01	3.80985E-20	8.70351E-19
ENSG00000153487	ING1	1.05	3.9357E-20	8.97873E-19
ENSG00000151532	VTI1A	0.80	3.9393E-20	8.97873E-19
ENSG00000065057	NTHL1	-1.36	4.11245E-20	9.36272E-19
ENSG00000146963	LUC7L2	0.79	4.275E-20	9.72174E-19
ENSG00000113312	TTC1	0.86	4.47923E-20	1.01746E-18
ENSG00000160796	NBEAL2	-1.02	4.49706E-20	1.02035E-18
ENSG00000182768	NGRN	0.92	4.57584E-20	1.03705E-18
ENSG00000031081	ARHGAP31	2.24	4.7061E-20	1.06537E-18
ENSG00000157214	STEAP2	-1.23	4.94915E-20	1.11912E-18
ENSG00000090006	LTBP4	-0.73	5.24054E-20	1.18368E-18
ENSG00000172175	MALT1	-0.79	5.2856E-20	1.19121E-18
ENSG00000272068	AL365181.2	-2.20	5.28579E-20	1.19121E-18
ENSG00000111321	LTBR	0.69	5.37801E-20	1.21063E-18
ENSG00000215271	HOMEZ	-1.40	5.63346E-20	1.26671E-18
ENSG00000127564	PKMYT1	-0.83	5.6715E-20	1.27383E-18
ENSG00000066084	DIP2B	0.84	5.71314E-20	1.28174E-18
ENSG00000171224	FAM241B	1.17	5.83403E-20	1.3074E-18
ENSG00000162384	CZIB	-1.35	5.92762E-20	1.32689E-18
ENSG00000157870	PRXL2B	-1.21	6.02305E-20	1.34674E-18
ENSG00000163902	RPN1	0.53	6.33494E-20	1.4149E-18
ENSG00000176890	TYMS	-0.63	6.58281E-20	1.46862E-18
ENSG00000168883	USP39	-0.68	6.60776E-20	1.47255E-18
ENSG00000185344	ATP6V0A2	-1.24	6.79194E-20	1.5119E-18
ENSG00000172775	PSME3IP1	-0.89	6.89181E-20	1.53243E-18
ENSG00000085978	ATG16L1	0.86	7.32512E-20	1.62697E-18
ENSG00000086475	SEPHS1	0.70	7.8764E-20	1.74748E-18
ENSG00000182742	HOXB4	-1.20	8.94753E-20	1.98292E-18
ENSG00000111450	STX2	-1.19	9.05299E-20	2.00408E-18
ENSG00000175130	MARCKSL1	-0.80	9.3256E-20	2.06214E-18
ENSG00000196535	MYO18A	-1.04	9.71174E-20	2.14516E-18
ENSG00000168743	NPNT	-1.48	9.97831E-20	2.20161E-18
ENSG00000103353	UBFD1	-0.69	1.00766E-19	2.22084E-18
ENSG00000068912	ERLEC1	1.07	1.01071E-19	2.22512E-18
ENSG00000127870	RNF6	1.04	1.03929E-19	2.28553E-18
ENSG00000204463	BAG6	0.55	1.12727E-19	2.47627E-18
ENSG00000176973	FAM89B	-1.66	1.16357E-19	2.55323E-18
ENSG00000198858	R3HDM4	-0.76	1.19016E-19	2.60871E-18
ENSG00000185697	MYBL1	1.14	1.24737E-19	2.7311E-18
ENSG00000057468	MSH4	2.47	1.25436E-19	2.74341E-18
ENSG00000103066	PLA2G15	1.37	1.29384E-19	2.82668E-18
ENSG00000176108	CHMP6	1.11	1.32991E-19	2.9023E-18
ENSG00000198157	HMGN5	1.36	1.39862E-19	3.04894E-18
ENSG00000155506	LARP1	-0.61	1.4348E-19	3.12439E-18

ENSG00000278041	AL133325.3	-2.21	1.44181E-19	3.13625E-18
ENSG00000134531	EMP1	-0.84	1.65194E-19	3.58942E-18
ENSG00000139613	SMARCC2	-0.76	1.74105E-19	3.77895E-18
ENSG00000108588	CCDC47	0.61	1.8465E-19	4.00349E-18
ENSG00000109919	MTCH2	-0.73	1.85884E-19	4.02587E-18
ENSG00000154529	CNTNAP3B	1.54	1.88726E-19	4.08301E-18
ENSG00000063601	MTMR1	-1.16	1.94491E-19	4.20319E-18
ENSG00000110536	PTPMT1	1.28	2.1361E-19	4.61139E-18
ENSG00000171928	TVP23B	0.81	2.28578E-19	4.9292E-18
ENSG00000099622	CIRBP	-0.67	2.33455E-19	5.02895E-18
ENSG00000103249	CLCN7	-1.12	2.35469E-19	5.06689E-18
ENSG00000162734	PEA15	0.69	2.35997E-19	5.0728E-18
ENSG00000148572	NRBF2	1.62	2.54032E-19	5.4546E-18
ENSG00000137700	SLC37A4	-0.89	2.60029E-19	5.57738E-18
ENSG00000068650	ATP11A	-0.91	2.64409E-19	5.65929E-18
ENSG00000205476	CCDC85C	-1.22	2.64413E-19	5.65929E-18
ENSG00000160007	ARHGAP35	-0.74	2.66338E-19	5.69439E-18
ENSG00000154582	ELOC	0.67	2.76623E-19	5.90799E-18
ENSG00000139178	C1RL	-1.57	2.77381E-19	5.91786E-18
ENSG00000153814	JAZF1	1.71	2.85697E-19	6.08879E-18
ENSG00000158615	PPP1R15B	0.64	2.89252E-19	6.15799E-18
ENSG00000108946	PRKAR1A	0.60	2.94407E-19	6.26107E-18
ENSG00000163605	PPP4R2	0.78	2.99848E-19	6.37003E-18
ENSG00000241360	PDXP	-1.74	3.02189E-19	6.41295E-18
ENSG00000158109	TPRG1L	0.87	3.03938E-19	6.44323E-18
ENSG00000272335	AC093297.2	-3.18	3.13029E-19	6.62893E-18
ENSG00000077312	SNRPA	-0.72	3.21629E-19	6.80385E-18
ENSG00000167986	DDB1	-0.55	3.2919E-19	6.95644E-18
ENSG00000143674	MAP3K21	-1.39	3.34018E-19	7.05099E-18
ENSG00000226887	ERVMER34-1	-1.76	3.34369E-19	7.05099E-18
ENSG00000164896	FASTK	-0.86	3.45556E-19	7.27922E-18
ENSG00000099785	MARCHF2	-1.79	3.52591E-19	7.41962E-18
ENSG00000130402	ACTN4	-0.52	3.59704E-19	7.56134E-18
ENSG00000133678	TMEM254	-1.88	3.74432E-19	7.86268E-18
ENSG00000174437	ATP2A2	0.54	3.85478E-19	8.08615E-18
ENSG00000177383	MAGEF1	-0.89	3.87108E-19	8.11185E-18
ENSG00000133134	BEX2	-1.07	3.98457E-19	8.34093E-18
ENSG00000169223	LMAN2	0.57	4.21107E-19	8.80585E-18
ENSG00000006634	DBF4	0.91	4.22031E-19	8.81596E-18
ENSG00000182095	TNRC18	-0.63	4.2985E-19	8.96993E-18
ENSG00000153885	KCTD15	-1.26	4.32404E-19	9.01382E-18
ENSG00000104833	TUBB4A	1.07	4.33364E-19	9.02443E-18
ENSG00000103335	PIEZO1	-0.62	4.43834E-19	9.23286E-18
ENSG00000215252	GOLGA8B	-1.18	4.59514E-19	9.54911E-18
ENSG00000184500	PROS1	1.47	4.63378E-19	9.61942E-18
ENSG00000111886	GABRR2	3.60	4.83893E-19	1.00349E-17

ENSG00000105404	RABAC1	0.96	4.91275E-19	1.01774E-17
ENSG00000221867	MAGEA3	0.78	5.17332E-19	1.07062E-17
ENSG00000197771	MCMBP	-0.70	5.35567E-19	1.10721E-17
ENSG00000004142	POLDIP2	-0.74	5.47745E-19	1.13121E-17
ENSG00000138081	FBXO11	0.86	5.65369E-19	1.16641E-17
ENSG00000072110	ACTN1	0.62	5.92688E-19	1.22151E-17
ENSG00000112763	BTN2A1	1.09	6.03531E-19	1.24258E-17
ENSG00000120656	TAF12	-1.12	6.28354E-19	1.29236E-17
ENSG00000167632	TRAPPC9	-1.19	6.30725E-19	1.2959E-17
ENSG00000108852	MPP2	-1.36	6.91014E-19	1.41831E-17
ENSG00000164164	OTUD4	-0.93	7.10837E-19	1.45751E-17
ENSG00000162923	WDR26	0.68	7.16094E-19	1.46678E-17
ENSG00000126709	IFI6	1.35	7.30283E-19	1.49432E-17
ENSG00000089685	BIRC5	0.66	8.57016E-19	1.75162E-17
ENSG00000176102	CSTF3	0.89	8.57778E-19	1.75162E-17
ENSG00000065802	ASB1	-0.76	8.63533E-19	1.76157E-17
ENSG00000181284	TMEM102	1.21	8.66862E-19	1.76656E-17
ENSG00000187079	TEAD1	-0.70	8.72121E-19	1.77547E-17
ENSG00000198585	NUDT16	-1.16	8.75938E-19	1.78143E-17
ENSG00000197457	STMN3	-0.65	8.77508E-19	1.78281E-17
ENSG00000161013	MGAT4B	-0.77	8.97067E-19	1.8207E-17
ENSG00000122642	FKBP9	-0.67	9.31985E-19	1.88965E-17
ENSG00000136937	NCBP1	-0.86	1.02733E-18	2.08086E-17
ENSG00000197279	ZNF165	1.60	1.03335E-18	2.09094E-17
ENSG00000160216	AGPAT3	-0.78	1.04942E-18	2.12131E-17
ENSG00000129353	SLC44A2	-0.74	1.05211E-18	2.12461E-17
ENSG00000129083	COPB1	0.69	1.07465E-18	2.16794E-17
ENSG00000112335	SNX3	0.63	1.10228E-18	2.22144E-17
ENSG00000276293	PIP4K2B	-0.78	1.17884E-18	2.37334E-17
ENSG00000198837	DENND4B	-0.90	1.1958E-18	2.40505E-17
ENSG00000141664	ZCCHC2	1.19	1.20667E-18	2.42448E-17
ENSG00000177731	FLII	-0.62	1.22445E-18	2.45775E-17
ENSG00000172977	KAT5	0.86	1.28766E-18	2.58203E-17
ENSG00000047249	ATP6V1H	1.00	1.2961E-18	2.59636E-17
ENSG00000167291	TBC1D16	-1.00	1.30695E-18	2.61548E-17
ENSG00000182504	CEP97	1.22	1.39613E-18	2.79115E-17
ENSG00000116350	SRSF4	0.63	1.40132E-18	2.79873E-17
ENSG00000160606	TLCD1	-1.40	1.65225E-18	3.2966E-17
ENSG00000142910	TINAGL1	-0.77	1.67957E-18	3.34777E-17
ENSG00000169429	CXCL8	2.49	1.68662E-18	3.35847E-17
ENSG00000104856	RELB	1.23	1.73247E-18	3.44634E-17
ENSG00000100354	TNRC6B	0.87	1.79725E-18	3.57165E-17
ENSG00000101457	DNTTIP1	-1.03	1.8472E-18	3.66728E-17
ENSG00000186166	CCDC84	0.92	1.85477E-18	3.67866E-17
ENSG00000141971	MVB12A	-0.93	1.88145E-18	3.72787E-17
ENSG00000085063	CD59	0.63	1.9358E-18	3.83177E-17

ENSG00000176871	WSB2	0.64	1.95641E-18	3.86874E-17
ENSG00000016391	CHDH	-1.56	2.03114E-18	4.01256E-17
ENSG00000129451	KLK10	-2.24	2.0543E-18	4.0543E-17
ENSG00000118263	KLF7	-1.35	2.10252E-18	4.14539E-17
ENSG00000002919	SNX11	0.94	2.14462E-18	4.22423E-17
ENSG00000175115	PACS1	-0.99	2.19423E-18	4.3177E-17
ENSG00000206418	RAB12	0.75	2.2759E-18	4.474E-17
ENSG00000198742	SMURF1	0.72	2.29982E-18	4.51658E-17
ENSG00000130517	PGPEP1	0.84	2.31388E-18	4.53975E-17
ENSG00000139269	INHBE	1.95	2.37305E-18	4.65128E-17
ENSG00000175073	VCPIP1	1.01	2.41491E-18	4.72796E-17
ENSG00000182173	TSEN54	-0.82	2.4169E-18	4.72796E-17
ENSG00000262943	ALOX12P2	1.57	2.55905E-18	5.00115E-17
ENSG00000048828	FAM120A	-0.63	2.6361E-18	5.1467E-17
ENSG00000138614	INTS14	-0.83	2.68465E-18	5.23638E-17
ENSG00000093009	CDC45	-0.95	2.72075E-18	5.30162E-17
ENSG00000130313	PGLS	-1.10	2.8275E-18	5.50427E-17
ENSG00000010292	NCAPD2	0.51	2.85616E-18	5.55465E-17
ENSG00000101972	STAG2	0.83	2.88397E-18	5.60331E-17
ENSG00000174574	AKIRIN1	0.63	2.91064E-18	5.64964E-17
ENSG00000080371	RAB21	-0.91	3.04884E-18	5.91214E-17
ENSG00000108826	MRPL27	0.68	3.11829E-18	6.04097E-17
ENSG00000135926	TMBIM1	0.73	3.15362E-18	6.10351E-17
ENSG00000134824	FADS2	-1.34	3.37202E-18	6.5199E-17
ENSG00000044574	HSPA5	0.50	3.37561E-18	6.52053E-17
ENSG00000167674	HDGFL2	0.64	3.41623E-18	6.59263E-17
ENSG00000105447	GRWD1	0.70	3.43934E-18	6.63084E-17
ENSG00000160712	IL6R	1.12	3.60369E-18	6.941E-17
ENSG00000188807	TMEM201	-1.22	3.62658E-18	6.97839E-17
ENSG00000069482	GAL	0.87	3.87215E-18	7.44375E-17
ENSG00000121022	COPS5	0.74	4.06037E-18	7.79585E-17
ENSG00000233695	GAS6-AS1	-2.30	4.0631E-18	7.79585E-17
ENSG00000142546	NOSIP	0.65	4.09794E-18	7.85518E-17
ENSG00000148840	PPRC1	-0.69	4.11106E-18	7.87277E-17
ENSG00000130382	MLLT1	-0.82	4.3111E-18	8.24797E-17
ENSG00000100395	L3MBTL2	-1.05	4.46387E-18	8.5321E-17
ENSG00000139291	TMEM19	0.98	4.68004E-18	8.93672E-17
ENSG00000145495	MARCHF6	0.59	4.73934E-18	9.04135E-17
ENSG00000114779	ABHD14B	-0.94	5.00024E-18	9.52998E-17
ENSG00000137714	FDX1	-1.05	5.14229E-18	9.7914E-17
ENSG00000188042	ARL4C	1.20	5.27996E-18	1.0044E-16
ENSG00000050438	SLC4A8	2.38	5.63305E-18	1.07055E-16
ENSG00000055483	USP36	-0.77	5.858E-18	1.11224E-16
ENSG00000112306	RPS12	-0.60	6.08132E-18	1.15355E-16
ENSG00000196911	KPNA5	1.43	6.12127E-18	1.16003E-16
ENSG00000131504	DIAPH1	-0.55	6.29599E-18	1.19201E-16

ENSG00000166780	BMERB1	1.02	6.38062E-18	1.20689E-16
ENSG00000109180	OCIAD1	0.62	6.4404E-18	1.21705E-16
ENSG00000058866	DGKG	1.52	6.72474E-18	1.26958E-16
ENSG00000111897	SERINC1	0.74	6.74878E-18	1.27292E-16
ENSG00000101210	EEF1A2	-0.60	6.94953E-18	1.30955E-16
ENSG00000166348	USP54	-1.27	7.04438E-18	1.32617E-16
ENSG00000151893	CACUL1	-0.67	7.08498E-18	1.33256E-16
ENSG00000166847	DCTN5	0.65	7.12721E-18	1.33925E-16
ENSG00000101365	IDH3B	0.67	7.22598E-18	1.35653E-16
ENSG00000104960	PTOV1	0.64	7.35364E-18	1.3792E-16
ENSG00000100372	SLC25A17	-1.31	7.43435E-18	1.39304E-16
ENSG00000112118	MCM3	-0.51	7.72262E-18	1.4457E-16
ENSG00000130741	EIF2S3	-0.55	7.79528E-18	1.45794E-16
ENSG00000168374	ARF4	0.58	7.83245E-18	1.46352E-16
ENSG00000255455	AP003486.1	-2.66	7.96256E-18	1.48644E-16
ENSG00000162241	SLC25A45	2.24	8.02218E-18	1.49618E-16
ENSG00000238105	GOLGA2P5	-1.59	8.63185E-18	1.60838E-16
ENSG00000167553	TUBA1C	0.55	8.86321E-18	1.64881E-16
ENSG00000134287	ARF3	-0.56	8.8653E-18	1.64881E-16
ENSG00000188612	SUMO2	0.52	8.9965E-18	1.67166E-16
ENSG00000152422	XRCC4	1.29	9.725E-18	1.80535E-16
ENSG00000126803	HSPA2	1.95	1.01094E-17	1.87497E-16
ENSG00000184162	NR2C2AP	-1.04	1.05323E-17	1.95159E-16
ENSG00000103148	NPRL3	-1.38	1.07891E-17	1.99732E-16
ENSG00000123091	RNF11	0.73	1.08202E-17	2.00125E-16
ENSG00000166965	RCCD1	-0.96	1.08653E-17	2.00772E-16
ENSG00000010803	SCMH1	-1.26	1.10417E-17	2.03844E-16
ENSG00000013275	PSMC4	0.64	1.13229E-17	2.08843E-16
ENSG00000078618	NRDC	0.55	1.16251E-17	2.14219E-16
ENSG00000236144	TMEM147-AS1	-1.28	1.19873E-17	2.2069E-16
ENSG00000138119	MYOF	-0.65	1.20247E-17	2.21174E-16
ENSG00000128242	GAL3ST1	3.11	1.20518E-17	2.21469E-16
ENSG00000163636	PSMD6	0.65	1.25504E-17	2.30421E-16
ENSG00000269743	SLC25A53	2.77	1.2838E-17	2.35484E-16
ENSG00000148634	HERC4	0.96	1.31734E-17	2.41416E-16
ENSG00000115091	ACTR3	0.56	1.36299E-17	2.49552E-16
ENSG00000146918	NCAPG2	0.62	1.41449E-17	2.58745E-16
ENSG00000140688	RUSF1	-1.01	1.42723E-17	2.60837E-16
ENSG00000175376	EIF1AD	0.76	1.44544E-17	2.63925E-16
ENSG00000142507	PSMB6	0.65	1.45214E-17	2.64907E-16
ENSG00000254858	MPV17L2	-1.15	1.46429E-17	2.66879E-16
ENSG00000196396	PTPN1	0.65	1.50818E-17	2.74628E-16
ENSG00000197885	NKIRAS1	1.21	1.57323E-17	2.86214E-16
ENSG00000135519	KCNH3	-1.03	1.68802E-17	3.06818E-16
ENSG00000079459	FDFT1	0.59	1.71805E-17	3.11994E-16
ENSG00000158747	NBL1	-0.89	1.76248E-17	3.19771E-16

ENSG00000153574	RPIA	-0.81	1.77751E-17	3.22205E-16
ENSG00000198053	SIRPA	-1.15	1.91015E-17	3.45936E-16
ENSG00000120915	EPHX2	-1.53	1.91261E-17	3.46068E-16
ENSG00000179604	CDC42EP4	-0.77	1.95148E-17	3.52782E-16
ENSG00000127054	INTS11	-0.69	2.04016E-17	3.68481E-16
ENSG00000158373	H2BC5	2.18	2.04276E-17	3.68618E-16
ENSG00000175643	RMI2	-1.34	2.13009E-17	3.8403E-16
ENSG00000125503	PPP1R12C	-0.91	2.25243E-17	4.05721E-16
ENSG00000204387	SNHG32	-0.59	2.38231E-17	4.2873E-16
ENSG00000224597	SVIL-AS1	1.20	2.45979E-17	4.42276E-16
ENSG00000114354	TFG	0.59	2.50154E-17	4.49379E-16
ENSG00000148308	GTF3C5	0.62	2.53274E-17	4.54576E-16
ENSG00000009830	POMT2	-1.13	2.53832E-17	4.55169E-16
ENSG00000103021	CCDC113	-1.51	2.55378E-17	4.57531E-16
ENSG00000173436	MICOS10	0.72	2.56751E-17	4.5958E-16
ENSG00000115233	PSMD14	0.59	2.59202E-17	4.63553E-16
ENSG00000148229	POLE3	0.58	2.68686E-17	4.80084E-16
ENSG00000151014	NOCT	1.01	2.77672E-17	4.95697E-16
ENSG00000158402	CDC25C	1.01	2.7963E-17	4.98749E-16
ENSG00000204592	HLA-E	0.61	3.03942E-17	5.41628E-16
ENSG00000119711	ALDH6A1	-1.48	3.06236E-17	5.4523E-16
ENSG00000164294	GPX8	0.84	3.11002E-17	5.53224E-16
ENSG00000070413	DGCR2	-0.77	3.18713E-17	5.66436E-16
ENSG00000149657	LSM14B	-0.77	3.30437E-17	5.86753E-16
ENSG00000185847	LINC01405	-2.52	3.42518E-17	6.07667E-16
ENSG00000025772	TOMM34	-0.71	3.51807E-17	6.23593E-16
ENSG00000181031	RPH3AL	-1.89	3.54018E-17	6.26956E-16
ENSG00000183087	GAS6	0.82	3.54337E-17	6.26967E-16
ENSG00000167600	CYP2S1	-1.39	3.59173E-17	6.34962E-16
ENSG00000118454	ANKRD13C	1.04	3.79703E-17	6.70664E-16
ENSG00000063660	GPC1	-0.75	4.01362E-17	7.08294E-16
ENSG00000076984	MAP2K7	-0.85	4.10061E-17	7.23007E-16
ENSG00000143228	NUF2	0.87	4.14683E-17	7.30513E-16
ENSG00000182158	CREB3L2	-0.75	4.15611E-17	7.31505E-16
ENSG00000136270	TBRG4	-0.64	4.16203E-17	7.31903E-16
ENSG00000062194	GPBP1	0.80	4.42548E-17	7.77548E-16
ENSG00000277443	MARCKS	0.69	4.64881E-17	8.15939E-16
ENSG00000159166	LAD1	-0.95	4.65214E-17	8.15939E-16
ENSG00000096092	TMEM14A	0.73	4.69539E-17	8.22804E-16
ENSG00000129757	CDKN1C	1.30	4.72063E-17	8.26502E-16
ENSG00000173295	FAM86B3P	-2.33	4.77047E-17	8.34499E-16
ENSG00000168993	CPLX1	-2.19	5.03122E-17	8.79343E-16
ENSG00000108854	SMURF2	-0.58	5.06993E-17	8.85335E-16
ENSG00000072778	ACADVL	-0.55	5.31472E-17	9.27273E-16
ENSG00000119333	WDR34	-0.73	5.3548E-17	9.33452E-16
ENSG00000196141	SPATS2L	0.61	5.50998E-17	9.59667E-16

ENSG00000146859	TMEM140	-2.30	5.52903E-17	9.62148E-16
ENSG00000092036	HAUS4	-1.20	5.55059E-17	9.6506E-16
ENSG00000175197	DDIT3	-0.91	5.76545E-17	1.00155E-15
ENSG00000186395	KRT10	0.80	5.88952E-17	1.02221E-15
ENSG00000105397	TYK2	-0.78	5.91858E-17	1.02637E-15
ENSG00000117448	AKR1A1	-0.67	6.12364E-17	1.06101E-15
ENSG00000005020	SKAP2	-1.01	6.25217E-17	1.08234E-15
ENSG00000135002	RFK	0.90	6.42144E-17	1.11068E-15
ENSG00000126524	SBDS	0.81	6.50708E-17	1.12452E-15
ENSG00000235823	OLMALINC	0.77	6.54173E-17	1.12954E-15
ENSG00000132694	ARHGEF11	-1.08	6.66723E-17	1.15021E-15
ENSG00000275342	PRAG1	-1.06	6.77681E-17	1.16811E-15
ENSG00000011451	WIZ	-1.01	6.78918E-17	1.16923E-15
ENSG00000187051	RPS19BP1	0.73	6.87813E-17	1.18263E-15
ENSG00000179409	GEMIN4	-0.75	6.87877E-17	1.18263E-15
ENSG00000204574	ABCF1	0.60	7.13797E-17	1.22614E-15
ENSG00000115310	RTN4	0.52	7.20006E-17	1.23574E-15
ENSG00000173065	FAM222B	-1.21	7.44805E-17	1.27721E-15
ENSG00000130477	UNC13A	-0.75	7.61308E-17	1.30439E-15
ENSG00000064666	CNN2	-0.58	7.76091E-17	1.32858E-15
ENSG00000186897	C1QL4	1.28	7.80534E-17	1.33505E-15
ENSG00000165689	ENTR1	-0.78	8.06178E-17	1.37773E-15
ENSG00000113273	ARSB	-1.84	8.37363E-17	1.4298E-15
ENSG00000273033	LINC02035	-2.08	8.64812E-17	1.47542E-15
ENSG00000135317	SNX14	0.83	9.13077E-17	1.55643E-15
ENSG00000154059	IMPACT	-1.01	9.24906E-17	1.57525E-15
ENSG00000100796	PPP4R3A	0.69	9.26551E-17	1.57671E-15
ENSG00000158941	CCAR2	-0.64	9.39748E-17	1.59781E-15
ENSG00000168944	CEP120	-1.05	9.57949E-17	1.62738E-15
ENSG00000161513	FDXR	-0.69	9.6357E-17	1.63554E-15
ENSG00000137100	DCTN3	0.85	9.93012E-17	1.68408E-15
ENSG00000113356	POLR3G	-0.91	9.98289E-17	1.6916E-15
ENSG00000203880	PCMTD2	-1.08	1.03072E-16	1.74507E-15
ENSG00000130775	THEMIS2	1.34	1.04192E-16	1.76254E-15
ENSG00000064419	TNPO3	0.59	1.0563E-16	1.78536E-15
ENSG00000118518	RNF146	1.20	1.0712E-16	1.80797E-15
ENSG00000101367	MAPRE1	0.52	1.07149E-16	1.80797E-15
ENSG00000104897	SF3A2	-0.73	1.13698E-16	1.91688E-15
ENSG00000132589	FLOT2	-0.81	1.20423E-16	2.02853E-15
ENSG00000111639	MRPL51	0.60	1.21145E-16	2.03898E-15
ENSG00000159131	GART	-0.66	1.23623E-16	2.07895E-15
ENSG00000181444	ZNF467	1.43	1.26067E-16	2.11827E-15
ENSG00000041357	PSMA4	0.59	1.27978E-16	2.14858E-15
ENSG00000170542	SERPINB9	0.65	1.30514E-16	2.1876E-15
ENSG00000180357	ZNF609	-0.81	1.30521E-16	2.1876E-15
ENSG00000145725	PPIP5K2	1.02	1.33331E-16	2.23283E-15

ENSG00000113811	SELENOK	0.83	1.34319E-16	2.24573E-15
ENSG00000157227	MMP14	0.98	1.34326E-16	2.24573E-15
ENSG00000115194	SLC30A3	-1.32	1.38128E-16	2.30737E-15
ENSG00000188229	TUBB4B	0.58	1.48109E-16	2.47141E-15
ENSG00000166444	DENND2B	-2.18	1.48196E-16	2.47141E-15
ENSG00000135829	DHX9	0.54	1.4973E-16	2.49491E-15
ENSG00000105197	TIMM50	-0.69	1.58196E-16	2.6338E-15
ENSG00000126787	DLGAP5	0.76	1.61292E-16	2.6831E-15
ENSG00000165215	CLDN3	1.22	1.6156E-16	2.68533E-15
ENSG00000180694	TMEM64	-0.78	1.62612E-16	2.70057E-15
ENSG00000075785	RAB7A	0.58	1.72243E-16	2.85815E-15
ENSG00000164251	F2RL1	0.62	1.73274E-16	2.87288E-15
ENSG00000176953	NFATC2IP	-0.87	1.79641E-16	2.97597E-15
ENSG00000131069	ACSS2	-0.91	1.85429E-16	3.06932E-15
ENSG00000143870	PDIA6	0.51	1.91901E-16	3.17382E-15
ENSG00000119977	TCTN3	-0.85	1.94635E-16	3.21638E-15
ENSG00000108465	CDK5RAP3	-0.81	1.95728E-16	3.23177E-15
ENSG00000142002	DPP9	0.63	1.99557E-16	3.29229E-15
ENSG00000215021	PHB2	-0.50	2.03977E-16	3.36243E-15
ENSG00000099194	SCD	-0.54	2.04274E-16	3.36456E-15
ENSG00000224578	HNRNPA1P48	-0.79	2.05563E-16	3.383E-15
ENSG00000166483	WEE1	0.70	2.14369E-16	3.52502E-15
ENSG00000006451	RALA	-0.79	2.30387E-16	3.78531E-15
ENSG00000278129	ZNF8	-1.46	2.34151E-16	3.84399E-15
ENSG00000147955	SIGMAR1	-0.72	2.34619E-16	3.84853E-15
ENSG00000100029	PES1	-0.56	2.38801E-16	3.91392E-15
ENSG00000250067	YJEFN3	-1.89	2.46641E-16	4.03911E-15
ENSG00000130560	UBAC1	-0.86	2.52247E-16	4.12753E-15
ENSG00000177628	GBA	0.97	2.60509E-16	4.25924E-15
ENSG00000177728	TMEM94	-0.96	2.63683E-16	4.30762E-15
ENSG00000100294	MCAT	-1.17	2.67681E-16	4.36936E-15
ENSG00000185825	BCAP31	0.58	2.73219E-16	4.45612E-15
ENSG00000107929	LARP4B	-0.66	2.74435E-16	4.47232E-15
ENSG00000134057	CCNB1	0.53	2.98479E-16	4.86017E-15
ENSG00000100243	CYB5R3	-0.62	3.0549E-16	4.97029E-15
ENSG00000153767	GTF2E1	1.01	3.14677E-16	5.1156E-15
ENSG00000073969	NSF	-0.85	3.16883E-16	5.1473E-15
ENSG00000066926	FECH	-0.76	3.1738E-16	5.15119E-15
ENSG00000090339	ICAM1	1.70	3.33149E-16	5.40274E-15
ENSG00000266088	AC004585.1	3.73	3.39218E-16	5.4967E-15
ENSG00000123416	TUBA1B	0.49	3.40477E-16	5.51264E-15
ENSG00000163807	KIAA1143	-1.22	3.42328E-16	5.53813E-15
ENSG00000135480	KRT7	-1.78	3.44175E-16	5.56351E-15
ENSG00000102172	SMS	0.54	3.46829E-16	5.60189E-15
ENSG00000198189	HSD17B11	0.86	3.47952E-16	5.6155E-15
ENSG00000177425	PAWR	-0.84	3.48433E-16	5.61873E-15

ENSG00000123240	OPTN	0.95	3.62225E-16	5.83644E-15
ENSG00000160633	SAFB	0.55	3.82366E-16	6.156E-15
ENSG00000251003	ZFPM2-AS1	1.29	3.89622E-16	6.26779E-15
ENSG00000159958	TNFRSF13C	-2.85	4.15998E-16	6.68671E-15
ENSG00000114554	PLXNA1	-0.85	4.20443E-16	6.75273E-15
ENSG00000181904	C5orf24	-0.90	4.25596E-16	6.83002E-15
ENSG00000092931	MFSD11	0.91	4.33528E-16	6.95173E-15
ENSG00000005206	SPPL2B	-1.03	4.41256E-16	7.06999E-15
ENSG00000075643	MOCOS	-1.07	4.46761E-16	7.15247E-15
ENSG00000109654	TRIM2	-1.05	4.50551E-16	7.20737E-15
ENSG00000170421	KRT8	-0.58	4.88959E-16	7.81554E-15
ENSG00000105223	PLD3	-0.70	4.89681E-16	7.82083E-15
ENSG00000175084	DES	1.68	5.18481E-16	8.27419E-15
ENSG00000171617	ENC1	0.83	5.19102E-16	8.27751E-15
ENSG00000120885	CLU	0.60	5.20848E-16	8.29874E-15
ENSG00000165102	HGSNAT	-1.49	5.21633E-16	8.30463E-15
ENSG00000129562	DAD1	0.61	5.24512E-16	8.34382E-15
ENSG00000197261	C6orf141	1.36	5.25547E-16	8.35366E-15
ENSG00000176533	GNG7	-1.27	5.47595E-16	8.69075E-15
ENSG00000185551	NR2F2	0.77	5.47623E-16	8.69075E-15
ENSG00000123815	COQ8B	-1.17	5.82181E-16	9.23187E-15
ENSG00000118564	FBXL5	0.90	6.20576E-16	9.83292E-15
ENSG00000225760	LINC00431	1.94	6.49178E-16	1.0278E-14
ENSG00000186591	UBE2H	-0.61	6.53359E-16	1.03281E-14
ENSG00000273230	AC102953.2	-3.86	6.53376E-16	1.03281E-14
ENSG00000107263	RAPGEF1	-0.70	6.59078E-16	1.041E-14
ENSG00000139410	SDSL	-1.41	6.83574E-16	1.07884E-14
ENSG00000110711	AIP	0.74	7.17306E-16	1.13118E-14
ENSG00000134250	NOTCH2	0.65	7.39711E-16	1.16478E-14
ENSG00000010278	CD9	0.52	7.39775E-16	1.16478E-14
ENSG00000128891	CCDC32	1.35	7.44507E-16	1.17131E-14
ENSG00000236552	RPL13AP5	-0.49	7.6722E-16	1.2061E-14
ENSG00000197106	SLC6A17	-1.25	7.82948E-16	1.22985E-14
ENSG00000176720	BOK	-1.06	8.57126E-16	1.34532E-14
ENSG00000068024	HDAC4	-0.98	8.88194E-16	1.39299E-14
ENSG00000134291	TMEM106C	0.54	8.99409E-16	1.40947E-14
ENSG00000168175	MAPK1IP1L	-0.70	9.12775E-16	1.4293E-14
ENSG00000124571	XPO5	-0.64	1.00344E-15	1.57004E-14
ENSG00000160392	C19orf47	1.05	1.01343E-15	1.58444E-14
ENSG00000177479	ARIH2	-0.61	1.05549E-15	1.64891E-14
ENSG00000125170	DOK4	1.02	1.06058E-15	1.65556E-14
ENSG00000066248	NGEF	1.32	1.06801E-15	1.66587E-14
ENSG00000113384	GOLPH3	-0.73	1.11737E-15	1.7415E-14
ENSG00000162433	AK4	-0.76	1.17911E-15	1.8363E-14
ENSG00000214223	HNRNPA1P10	-0.82	1.18608E-15	1.84571E-14
ENSG00000103111	MON1B	-1.00	1.28112E-15	1.99206E-14

ENSG00000103591	AAGAB	0.64	1.29578E-15	2.01329E-14
ENSG00000268089	GABRQ	1.02	1.30565E-15	2.02704E-14
ENSG00000173530	TNFRSF10D	0.64	1.3382E-15	2.07597E-14
ENSG00000136783	NIPSNAP3A	-1.59	1.34419E-15	2.08365E-14
ENSG00000196937	FAM3C	0.49	1.43685E-15	2.22557E-14
ENSG00000075420	FNDC3B	0.73	1.4762E-15	2.28474E-14
ENSG00000164916	FOXK1	-0.66	1.53556E-15	2.37477E-14
ENSG00000170537	TMC7	1.40	1.55607E-15	2.40464E-14
ENSG00000123989	CHPF	-0.58	1.56946E-15	2.42346E-14
ENSG00000165912	PACSIN3	-0.96	1.57876E-15	2.43595E-14
ENSG00000095539	SEMA4G	-1.29	1.60762E-15	2.47855E-14
ENSG00000173894	CBX2	0.71	1.62594E-15	2.50487E-14
ENSG00000142082	SIRT3	-1.26	1.63765E-15	2.52098E-14
ENSG00000172939	OXSR1	0.66	1.67045E-15	2.56949E-14
ENSG00000143390	RFX5	0.69	1.81267E-15	2.7861E-14
ENSG00000176014	TUBB6	0.61	1.81927E-15	2.79411E-14
ENSG00000176894	PXMP2	-1.09	1.82333E-15	2.79819E-14
ENSG00000189067	LITAF	0.59	1.84376E-15	2.82738E-14
ENSG00000100139	MICALL1	-1.10	1.86268E-15	2.8542E-14
ENSG00000175221	MED16	-0.88	1.89295E-15	2.89837E-14
ENSG00000007944	MYLIP	1.29	1.96855E-15	3.01182E-14
ENSG00000150347	ARID5B	1.28	1.98536E-15	3.03521E-14
ENSG00000261799	AC007406.5	-2.95	2.04138E-15	3.11848E-14
ENSG00000143486	EIF2D	-0.74	2.05034E-15	3.12978E-14
ENSG00000198894	CIPC	-1.18	2.08785E-15	3.18461E-14
ENSG00000144043	TEX261	-0.77	2.24705E-15	3.42483E-14
ENSG00000203950	RTL8A	-0.85	2.2527E-15	3.43082E-14
ENSG00000002586	CD99	0.75	2.30745E-15	3.51154E-14
ENSG00000174669	SLC29A2	-0.97	2.46573E-15	3.74955E-14
ENSG00000113810	SMC4	0.62	2.51877E-15	3.8273E-14
ENSG00000115414	FN1	0.88	2.52294E-15	3.83073E-14
ENSG00000123983	ACSL3	0.53	2.5624E-15	3.8877E-14
ENSG00000096433	ITPR3	-0.64	2.69027E-15	4.07861E-14
ENSG00000203668	CHML	-1.53	2.70754E-15	4.10168E-14
ENSG00000172269	DPAGT1	-0.99	2.71536E-15	4.11043E-14
ENSG00000102753	KPNA3	0.64	2.75223E-15	4.16309E-14
ENSG00000114316	USP4	0.74	2.80458E-15	4.23907E-14
ENSG00000117713	ARID1A	-0.70	2.87392E-15	4.34059E-14
ENSG00000160691	SHC1	-0.53	2.89717E-15	4.37241E-14
ENSG00000118515	SGK1	2.11	2.90718E-15	4.38421E-14
ENSG00000133119	RFC3	-0.81	2.95441E-15	4.45208E-14
ENSG00000009335	UBE3C	0.52	3.01084E-15	4.5337E-14
ENSG00000116525	TRIM62	-2.07	3.07156E-15	4.62167E-14
ENSG00000137038	DMAC1	-1.07	3.07399E-15	4.62184E-14
ENSG00000165475	CRYL1	-1.24	3.11926E-15	4.68639E-14
ENSG00000119915	ELOVL3	1.27	3.27027E-15	4.90958E-14

ENSG00000111077	TNS2	-1.20	3.29621E-15	4.94482E-14
ENSG00000162585	FAAP20	0.78	3.33661E-15	5.00166E-14
ENSG00000232119	MCTS1	0.71	3.53974E-15	5.30219E-14
ENSG00000069956	MAPK6	0.70	3.55477E-15	5.32037E-14
ENSG00000121691	CAT	-0.87	3.5572E-15	5.32037E-14
ENSG00000137193	PIM1	0.86	3.68331E-15	5.50489E-14
ENSG00000175318	GRAMD2A	-1.98	3.70296E-15	5.53011E-14
ENSG00000142459	EVI5L	-0.95	3.73403E-15	5.57237E-14
ENSG00000114956	DGUOK	0.69	3.7438E-15	5.58278E-14
ENSG00000176974	SHMT1	-0.75	3.75307E-15	5.59243E-14
ENSG00000166233	ARIH1	-0.70	3.86081E-15	5.74871E-14
ENSG00000180011	ZADH2	-1.13	3.92776E-15	5.84403E-14
ENSG00000136982	DSCC1	-0.89	3.97604E-15	5.91148E-14
ENSG00000160447	PKN3	-0.79	4.23398E-15	6.2903E-14
ENSG00000071553	ATP6AP1	0.64	4.38364E-15	6.50782E-14
ENSG00000111540	RAB5B	0.66	4.49042E-15	6.66141E-14
ENSG00000184995	IFNE	3.76	4.57418E-15	6.78063E-14
ENSG00000100097	LGALS1	0.56	4.67241E-15	6.92112E-14
ENSG00000123992	DNPEP	-0.84	4.69129E-15	6.94395E-14
ENSG00000100580	TMED8	-0.76	4.9366E-15	7.30165E-14
ENSG00000167461	RAB8A	0.64	5.02775E-15	7.43097E-14
ENSG00000162998	FRZB	2.47	5.25493E-15	7.76101E-14
ENSG00000165724	ZMYND19	-0.87	5.35351E-15	7.90077E-14
ENSG00000070495	JMJD6	0.73	5.41441E-15	7.98475E-14
ENSG00000213719	CLIC1	0.51	5.52699E-15	8.14478E-14
ENSG00000160767	FAM189B	-0.88	5.98953E-15	8.8199E-14
ENSG00000084234	APLP2	-0.45	6.17216E-15	9.08216E-14
ENSG00000119403	PHF19	0.64	6.23803E-15	9.17234E-14
ENSG00000259330	INAFM2	-1.33	6.31362E-15	9.27666E-14
ENSG00000138071	ACTR2	0.53	6.43079E-15	9.44188E-14
ENSG00000167657	DAPK3	0.83	6.44929E-15	9.46211E-14
ENSG00000130748	TMEM160	0.84	6.61708E-15	9.70118E-14
ENSG00000146731	CCT6A	0.48	6.68659E-15	9.79493E-14
ENSG00000070770	CSNK2A2	0.80	6.69082E-15	9.79493E-14
ENSG00000179195	ZNF664	-0.56	6.74034E-15	9.86022E-14
ENSG00000049245	VAMP3	-0.62	6.88852E-15	1.00696E-13
ENSG00000185359	HGS	-0.54	6.90453E-15	1.00857E-13
ENSG00000049759	NEDD4L	0.65	7.10809E-15	1.03754E-13
ENSG00000006744	ELAC2	0.54	7.19665E-15	1.0497E-13
ENSG00000171475	WIPF2	-0.74	7.27873E-15	1.0609E-13
ENSG00000054277	OPN3	3.65	7.305E-15	1.06396E-13
ENSG00000112339	HBS1L	0.74	7.42445E-15	1.08057E-13
ENSG00000267761	MIR4527HG	-1.03	7.8712E-15	1.14476E-13
ENSG00000157014	TATDN2	-1.26	8.01215E-15	1.16389E-13
ENSG00000138760	SCARB2	0.61	8.01437E-15	1.16389E-13
ENSG00000136699	SMPD4	-0.61	8.26771E-15	1.19981E-13

ENSG00000228300	FAM174C	0.83	8.31786E-15	1.20621E-13
ENSG00000138771	SHROOM3	-0.89	8.40359E-15	1.21776E-13
ENSG00000131899	LLGL1	-0.77	8.73751E-15	1.26523E-13
ENSG00000114346	ECT2	0.66	9.63372E-15	1.394E-13
ENSG00000117266	CDK18	-1.20	9.89659E-15	1.431E-13
ENSG00000142444	TIMM29	1.11	9.92384E-15	1.43391E-13
ENSG00000103121	CMC2	0.73	1.01724E-14	1.46877E-13
ENSG00000169213	RAB3B	0.64	1.05142E-14	1.51696E-13
ENSG00000108423	TUBD1	1.30	1.05214E-14	1.51696E-13
ENSG00000204271	SPIN3	-1.48	1.11918E-14	1.61245E-13
ENSG00000138678	GPAT3	0.60	1.12336E-14	1.61732E-13
ENSG00000105323	HNRNPUL1	-0.49	1.13884E-14	1.63842E-13
ENSG00000069812	HES2	0.99	1.15728E-14	1.66376E-13
ENSG00000100814	CCNB1IP1	-0.73	1.1833E-14	1.69994E-13
ENSG00000165280	VCP	0.47	1.20007E-14	1.7228E-13
ENSG00000185033	SEMA4B	-0.87	1.21378E-14	1.74123E-13
ENSG00000166831	RBPMS2	-2.06	1.22371E-14	1.75422E-13
ENSG00000172053	QARS1	-0.56	1.22528E-14	1.75521E-13
ENSG00000198719	DLL1	1.49	1.25682E-14	1.7991E-13
ENSG00000171992	SYNPO	-1.81	1.32967E-14	1.90203E-13
ENSG00000133961	NUMB	0.74	1.33205E-14	1.90407E-13
ENSG00000173653	RCE1	0.89	1.37226E-14	1.96014E-13
ENSG00000116830	TTF2	-0.97	1.38244E-14	1.97328E-13
ENSG00000174460	ZCCHC12	2.01	1.42212E-14	2.02847E-13
ENSG00000165782	PIP4P1	-0.92	1.48677E-14	2.11918E-13
ENSG00000111142	METAP2	-0.59	1.52553E-14	2.17288E-13
ENSG00000156453	PCDH1	-0.93	1.5903E-14	2.26352E-13
ENSG00000032444	PNPLA6	0.60	1.66313E-14	2.36549E-13
ENSG00000034152	MAP2K3	0.65	1.71343E-14	2.43471E-13
ENSG00000067829	IDH3G	0.84	1.71422E-14	2.43471E-13
ENSG00000258634	AL160006.1	-1.31	1.90908E-14	2.70954E-13
ENSG00000128989	ARPP19	-0.57	1.94287E-14	2.75554E-13
ENSG00000119801	YPEL5	0.91	1.9863E-14	2.81514E-13
ENSG00000176531	PHLDB3	1.34	1.99036E-14	2.8189E-13
ENSG00000148200	NR6A1	0.94	1.99787E-14	2.82754E-13
ENSG00000068137	PLEKHH3	0.65	2.04179E-14	2.88766E-13
ENSG00000159322	ADPGK	-0.79	2.0467E-14	2.89256E-13
ENSG00000265817	FSBP	-3.89	2.07908E-14	2.93597E-13
ENSG00000168283	BMI1	0.72	2.08035E-14	2.93597E-13
ENSG00000103351	CLUAP1	-1.40	2.10683E-14	2.97124E-13
ENSG00000104967	NOVA2	1.24	2.13317E-14	3.00627E-13
ENSG00000169035	KLK7	-1.89	2.14197E-14	3.01654E-13
ENSG00000089693	MLF2	0.58	2.15923E-14	3.03872E-13
ENSG00000074356	NCBP3	-0.79	2.22743E-14	3.13249E-13
ENSG00000187017	ESPN	-0.88	2.25698E-14	3.17181E-13
ENSG00000171953	ATPAF2	-1.03	2.28427E-14	3.20791E-13

ENSG00000144711	IQSEC1	-1.04	2.39119E-14	3.35572E-13
ENSG00000107331	ABCA2	-0.61	2.40184E-14	3.36784E-13
ENSG00000137825	ITPKA	1.36	2.4032E-14	3.36784E-13
ENSG00000104497	SNX16	1.43	2.41088E-14	3.37624E-13
ENSG00000196365	LONP1	-0.50	2.43931E-14	3.41147E-13
ENSG00000241878	PISD	0.70	2.43944E-14	3.41147E-13
ENSG00000070010	UFD1	0.61	2.46861E-14	3.44985E-13
ENSG00000126067	PSMB2	0.56	2.57634E-14	3.59788E-13
ENSG00000075043	KCNQ2	-0.88	2.59421E-14	3.62032E-13
ENSG00000221988	PPT2	1.17	2.60613E-14	3.63441E-13
ENSG00000181789	COPG1	-0.51	2.62479E-14	3.65789E-13
ENSG00000213066	CEP43	1.01	2.62887E-14	3.66103E-13
ENSG00000100099	HPS4	-0.97	2.71394E-14	3.77557E-13
ENSG00000125826	RBCK1	-1.21	2.71489E-14	3.77557E-13
ENSG00000273079	GRIN2B	-1.07	2.76324E-14	3.84013E-13
ENSG00000122882	ECD	0.93	2.90626E-14	4.03609E-13
ENSG00000159374	M1AP	-1.13	2.91606E-14	4.0469E-13
ENSG00000167778	SPRYD3	-0.67	2.97739E-14	4.12914E-13
ENSG00000135473	PAN2	-0.94	3.17812E-14	4.40447E-13
ENSG00000198887	SMC5	-1.00	3.18041E-14	4.40459E-13
ENSG00000183283	DAZAP2	-0.49	3.19088E-14	4.41604E-13
ENSG00000219545	UMAD1	-1.89	3.25029E-14	4.49515E-13
ENSG00000214753	HNRNPUL2	-0.66	3.27664E-14	4.52846E-13
ENSG00000166848	TERF2IP	0.64	3.28345E-14	4.53475E-13
ENSG00000169981	ZNF35	1.68	3.31975E-14	4.58171E-13
ENSG00000164574	GALNT10	-0.71	3.41214E-14	4.70598E-13
ENSG00000068724	TTC7A	-0.93	3.41743E-14	4.71003E-13
ENSG00000084764	MAPRE3	0.87	3.438E-14	4.73512E-13
ENSG00000133818	RRAS2	-0.84	3.52355E-14	4.8496E-13
ENSG00000106615	RHEB	0.61	3.676E-14	5.05595E-13
ENSG00000220205	VAMP2	-0.96	3.6821E-14	5.06086E-13
ENSG00000110171	TRIM3	1.15	3.88359E-14	5.33413E-13
ENSG00000280798	LINC00294	-1.47	3.91099E-14	5.36808E-13
ENSG00000143067	ZNF697	1.10	4.0711E-14	5.58402E-13
ENSG00000142166	IFNAR1	0.76	4.14758E-14	5.68502E-13
ENSG00000119979	DENND10	-1.31	4.18711E-14	5.73528E-13
ENSG00000112237	CCNC	0.65	4.20782E-14	5.7597E-13
ENSG00000160888	IER2	0.53	4.3641E-14	5.96858E-13
ENSG00000156973	PDE6D	-1.13	4.36638E-14	5.96858E-13
ENSG00000177105	RHOG	-1.03	4.40291E-14	6.0144E-13
ENSG00000172830	SSH3	-0.98	4.43373E-14	6.05238E-13
ENSG00000115073	ACTR1B	-0.93	4.43894E-14	6.05535E-13
ENSG00000196715	VKORC1L1	-0.67	4.44433E-14	6.05858E-13
ENSG00000090060	PAPOLA	0.50	4.45878E-14	6.07414E-13
ENSG00000136560	TANK	0.89	4.48724E-14	6.10875E-13
ENSG00000071575	TRIB2	-1.29	4.89644E-14	6.66129E-13

ENSG00000183255	PTTG1IP	0.51	5.03509E-14	6.84527E-13
ENSG00000175029	CTBP2	-0.59	5.16159E-14	7.01247E-13
ENSG00000182704	TSKU	-1.05	5.25312E-14	7.13199E-13
ENSG00000080189	SLC35C2	-0.87	5.34143E-14	7.24696E-13
ENSG00000108219	TSPAN14	-0.59	5.43876E-14	7.37401E-13
ENSG00000106554	CHCHD3	-0.71	5.6856E-14	7.70347E-13
ENSG00000126653	NSRP1	0.81	5.71788E-14	7.74196E-13
ENSG00000089775	ZBTB25	-1.20	5.89205E-14	7.9724E-13
ENSG00000146281	PM20D2	-0.95	6.05303E-14	8.18468E-13
ENSG00000184381	PLA2G6	-1.39	6.08155E-14	8.21768E-13
ENSG00000115415	STAT1	0.74	6.10005E-14	8.23713E-13
ENSG00000120889	TNFRSF10B	-0.47	6.33266E-14	8.54546E-13
ENSG00000197119	SLC25A29	-0.89	6.47707E-14	8.73444E-13
ENSG00000135334	AKIRIN2	0.70	6.49244E-14	8.74928E-13
ENSG00000084444	FAM234B	-0.99	6.66811E-14	8.97996E-13
ENSG00000287778	AC006230.1	-3.66	6.82545E-14	9.18567E-13
ENSG00000165617	DACT1	2.21	7.03178E-14	9.45699E-13
ENSG00000138646	HERC5	1.24	7.40961E-14	9.95843E-13
ENSG00000177410	ZFAS1	-0.51	7.44621E-14	1.00009E-12
ENSG00000197444	OGDHL	-1.18	7.4611E-14	1.00142E-12
ENSG00000116852	KIF21B	-1.22	7.69313E-14	1.03187E-12
ENSG00000163382	NAXE	-0.59	7.85648E-14	1.05307E-12
ENSG00000141905	NFIC	-0.59	7.86636E-14	1.05369E-12
ENSG00000069702	TGFBR3	1.29	8.03497E-14	1.07556E-12
ENSG00000121058	COIL	0.75	8.15634E-14	1.09107E-12
ENSG00000166226	CCT2	0.51	8.20171E-14	1.09641E-12
ENSG00000010404	IDS	1.21	8.31281E-14	1.11052E-12
ENSG00000149679	CABLES2	-1.28	8.36039E-14	1.11613E-12
ENSG00000120093	HOXB3	-1.22	8.60776E-14	1.14839E-12
ENSG00000270647	TAF15	-0.44	8.78011E-14	1.1706E-12
ENSG00000132467	UTP3	0.66	8.92596E-14	1.18926E-12
ENSG00000154310	TNIK	0.81	8.94397E-14	1.19086E-12
ENSG00000167969	ECI1	-0.65	9.09993E-14	1.21082E-12
ENSG00000080503	SMARCA2	0.92	9.4435E-14	1.2557E-12
ENSG00000227403	LINC01806	-1.58	9.5068E-14	1.26328E-12
ENSG00000171303	KCNK3	2.67	9.81086E-14	1.30282E-12
ENSG00000180891	CUEDC1	-0.51	9.83576E-14	1.30526E-12
ENSG00000102038	SMARCA1	0.74	9.86878E-14	1.30877E-12
ENSG00000131238	PPT1	0.66	1.00241E-13	1.32849E-12
ENSG00000110841	PPFIBP1	0.56	1.00458E-13	1.33049E-12
ENSG00000109436	TBC1D9	0.89	1.00753E-13	1.33351E-12
ENSG00000099399	MAGEB2	0.51	1.0112E-13	1.33749E-12
ENSG00000125967	NECAB3	-1.08	1.01695E-13	1.34421E-12
ENSG00000157379	DHRS1	-1.27	1.02256E-13	1.34999E-12
ENSG00000124635	H2BC11	3.07	1.02268E-13	1.34999E-12
ENSG00000149658	YTHDF1	-0.66	1.02341E-13	1.35006E-12

ENSG00000124789	NUP153	0.60	1.05666E-13	1.39301E-12
ENSG00000251322	SHANK3	-1.05	1.05752E-13	1.39322E-12
ENSG00000136802	LRRC8A	0.71	1.06393E-13	1.40075E-12
ENSG00000113269	RNF130	-0.85	1.1044E-13	1.45308E-12
ENSG00000125755	SYMPK	-0.55	1.11162E-13	1.46162E-12
ENSG00000172409	CLP1	0.93	1.12122E-13	1.47327E-12
ENSG00000182054	IDH2	-0.80	1.17265E-13	1.53983E-12
ENSG00000162368	CMPK1	0.51	1.19891E-13	1.57328E-12
ENSG00000129991	TNNI3	1.05	1.21174E-13	1.58908E-12
ENSG00000142208	AKT1	-0.53	1.24512E-13	1.63179E-12
ENSG00000160781	PAQR6	-1.79	1.26609E-13	1.65819E-12
ENSG00000104524	PYCR3	-1.11	1.2709E-13	1.66339E-12
ENSG00000104231	ZFAND1	-0.61	1.32261E-13	1.72994E-12
ENSG00000006016	CRLF1	-0.99	1.32545E-13	1.73252E-12
ENSG00000198786	MT-ND5	-0.50	1.36416E-13	1.78196E-12
ENSG00000186472	PCLO	-2.30	1.36508E-13	1.78199E-12
ENSG00000157570	TSPAN18	-1.28	1.39228E-13	1.8158E-12
ENSG00000138744	NAAA	-1.43	1.39279E-13	1.8158E-12
ENSG00000130733	YIPF2	-1.02	1.40468E-13	1.83011E-12
ENSG00000177469	CAVIN1	0.49	1.46434E-13	1.9066E-12
ENSG00000133393	CEP20	0.63	1.56399E-13	2.03501E-12
ENSG00000071054	MAP4K4	0.51	1.58464E-13	2.06054E-12
ENSG00000139687	RB1	0.68	1.63826E-13	2.12848E-12
ENSG00000037637	FBXO42	-0.86	1.63901E-13	2.12848E-12
ENSG00000162819	BROX	0.79	1.65267E-13	2.14482E-12
ENSG00000121957	GPSM2	0.77	1.66734E-13	2.16247E-12
ENSG00000162231	NXF1	0.69	1.69734E-13	2.19995E-12
ENSG00000185189	NRBP2	-1.02	1.70525E-13	2.20877E-12
ENSG00000110400	NECTIN1	-0.99	1.76328E-13	2.28245E-12
ENSG00000108557	RAI1	-0.73	1.7704E-13	2.29019E-12
ENSG00000161996	WDR90	-1.21	1.85298E-13	2.39547E-12
ENSG00000114383	TUSC2	0.72	1.86004E-13	2.40304E-12
ENSG00000119414	PPP6C	-1.55	1.86775E-13	2.41145E-12
ENSG00000115762	PLEKHB2	0.46	1.88199E-13	2.42827E-12
ENSG00000129355	CDKN2D	1.13	1.93446E-13	2.49436E-12
ENSG00000009950	MLXIPL	-0.81	1.94051E-13	2.50055E-12
ENSG00000101911	PRPS2	-0.77	2.01602E-13	2.59617E-12
ENSG00000169764	UGP2	0.71	2.04497E-13	2.63009E-12
ENSG00000176170	SPHK1	1.60	2.04499E-13	2.63009E-12
ENSG00000198646	NCOA6	-0.76	2.0633E-13	2.65195E-12
ENSG00000189306	RRP7A	-0.72	2.08662E-13	2.68019E-12
ENSG00000017483	SLC38A5	-0.95	2.13989E-13	2.74685E-12
ENSG00000164542	KIAA0895	1.13	2.14421E-13	2.75063E-12
ENSG00000146109	ABT1	0.66	2.16596E-13	2.77676E-12
ENSG00000124164	VAPB	0.60	2.19191E-13	2.80823E-12
ENSG00000122257	RBBP6	0.63	2.20197E-13	2.81931E-12

ENSG00000178177	LCORL	0.95	2.29268E-13	2.93357E-12
ENSG00000128534	LSM8	-0.74	2.29683E-13	2.93701E-12
ENSG00000135709	KIAA0513	0.98	2.30101E-13	2.94048E-12
ENSG00000086065	CHMP5	0.69	2.44101E-13	3.11739E-12
ENSG00000156261	CCT8	0.43	2.46933E-13	3.15155E-12
ENSG00000111665	CDCA3	0.70	2.49141E-13	3.1777E-12
ENSG00000164323	CFAP97	-0.91	2.5242E-13	3.21747E-12
ENSG00000214046	SMIM7	0.70	2.53157E-13	3.22482E-12
ENSG00000163798	SLC4A1AP	0.93	2.57162E-13	3.27374E-12
ENSG00000186767	SPIN4	-1.17	2.64244E-13	3.36177E-12
ENSG00000176148	TCP11L1	1.25	2.71264E-13	3.44887E-12
ENSG00000141736	ERBB2	-0.68	2.73495E-13	3.47504E-12
ENSG00000138134	STAMBPL1	1.21	2.81477E-13	3.57419E-12
ENSG00000128191	DGCR8	-0.65	2.86228E-13	3.63221E-12
ENSG00000173614	NMNAT1	-1.39	2.9401E-13	3.7286E-12
ENSG00000126698	DNAJC8	0.59	2.94859E-13	3.737E-12
ENSG00000267279	AC090409.1	2.29	2.99103E-13	3.78839E-12
ENSG00000198909	MAP3K3	-0.83	3.01845E-13	3.8207E-12
ENSG00000108061	SHOC2	0.69	3.08634E-13	3.90416E-12
ENSG00000152795	HNRNPDL	-0.49	3.11505E-13	3.93799E-12
ENSG00000163900	TMEM41A	-1.02	3.13881E-13	3.96553E-12
ENSG00000102390	PBDC1	1.06	3.17476E-13	4.00841E-12
ENSG00000088826	SMOX	-0.77	3.22046E-13	4.06354E-12
ENSG00000076067	RBMS2	-0.69	3.34782E-13	4.22158E-12
ENSG00000172602	RND1	2.11	3.35352E-13	4.22611E-12
ENSG00000117305	HMGCL	-0.88	3.37233E-13	4.24714E-12
ENSG00000278259	MYO19	-0.54	3.39268E-13	4.27009E-12
ENSG00000163374	YY1AP1	-0.84	3.40723E-13	4.28569E-12
ENSG00000069011	PITX1	-0.97	3.42106E-13	4.3004E-12
ENSG00000170242	USP47	0.91	3.4749E-13	4.36533E-12
ENSG00000205544	TMEM256	-0.89	3.64896E-13	4.58111E-12
ENSG00000126456	IRF3	-0.73	3.65725E-13	4.58865E-12
ENSG00000175806	MSRA	1.49	3.73838E-13	4.6875E-12
ENSG00000137198	GMPR	0.86	3.78351E-13	4.74111E-12
ENSG00000165905	LARGE2	-0.76	3.83808E-13	4.80648E-12
ENSG00000110697	PITPNM1	-0.69	3.84152E-13	4.80779E-12
ENSG00000102125	TAZ	-1.38	3.88485E-13	4.85898E-12
ENSG00000116761	CTH	-1.34	3.90836E-13	4.88533E-12
ENSG00000158793	NIT1	-0.80	3.91409E-13	4.88944E-12
ENSG00000132640	BTBD3	-1.18	3.91813E-13	4.89143E-12
ENSG00000160172	FAM86C2P	-1.85	3.95317E-13	4.9321E-12
ENSG00000013573	DDX11	-0.61	3.96143E-13	4.93932E-12
ENSG00000179832	MROH1	-0.82	4.08215E-13	5.08668E-12
ENSG00000106261	ZKSCAN1	-0.78	4.11433E-13	5.12359E-12
ENSG00000174125	TLR1	3.14	4.19144E-13	5.21636E-12
ENSG00000133226	SRRM1	0.49	4.28362E-13	5.32776E-12

ENSG00000117569	PTBP2	1.10	4.38863E-13	5.45499E-12
ENSG00000149480	MTA2	-0.55	4.42682E-13	5.49904E-12
ENSG00000111196	MAGOHB	-0.91	4.59596E-13	5.70561E-12
ENSG00000183853	KIRREL1	-0.74	4.60046E-13	5.70765E-12
ENSG00000135966	TGFBRAP1	-0.95	4.72087E-13	5.85341E-12
ENSG00000196449	YRDC	0.66	4.79039E-13	5.93593E-12
ENSG00000068366	ACSL4	0.72	4.88644E-13	6.05121E-12
ENSG00000167085	PHB	0.49	5.05343E-13	6.25413E-12
ENSG00000119514	GALNT12	-1.14	5.09359E-13	6.29993E-12
ENSG00000204410	MSH5	-1.17	5.12725E-13	6.33765E-12
ENSG00000155324	GRAMD2B	1.44	5.31115E-13	6.56091E-12
ENSG00000010818	HIVEP2	1.08	5.35403E-13	6.60909E-12
ENSG00000161547	SRSF2	-0.52	5.35676E-13	6.60909E-12
ENSG00000230131	LINC02641	2.81	5.60076E-13	6.90588E-12
ENSG00000213676	ATF6B	-0.68	5.83348E-13	7.1884E-12
ENSG00000126261	UBA2	-0.65	5.9218E-13	7.29275E-12
ENSG00000131725	WDR44	0.91	5.92994E-13	7.29827E-12
ENSG00000167895	TMC8	1.27	5.95677E-13	7.32679E-12
ENSG00000108179	PPIF	0.51	5.96771E-13	7.33574E-12
ENSG00000104979	C19orf53	0.65	6.13615E-13	7.53816E-12
ENSG00000116017	ARID3A	1.82	6.23211E-13	7.65135E-12
ENSG00000132953	XPO4	-0.80	6.35702E-13	7.79992E-12
ENSG00000170915	PAQR8	-1.89	6.42719E-13	7.88119E-12
ENSG00000149503	INCENP	0.60	6.47038E-13	7.92928E-12
ENSG00000102317	RBM3	-0.42	6.69228E-13	8.1962E-12
ENSG00000203760	CENPW	0.86	6.72829E-13	8.23527E-12
ENSG00000151835	SACS	-0.79	6.7435E-13	8.24884E-12
ENSG00000101928	MOSPD1	0.75	6.88823E-13	8.42072E-12
ENSG00000140990	NDUFB10	-0.69	6.9527E-13	8.49435E-12
ENSG00000166710	B2M	0.43	6.98752E-13	8.53168E-12
ENSG00000204576	PRR3	-1.08	7.09177E-13	8.65369E-12
ENSG00000107819	SFXN3	0.69	7.0998E-13	8.65821E-12
ENSG00000101146	RAE1	0.58	7.21744E-13	8.79631E-12
ENSG00000156398	SFXN2	-1.11	7.40256E-13	9.01644E-12
ENSG00000105662	CRTC1	-1.19	7.53977E-13	9.17797E-12
ENSG00000125753	VASP	0.61	7.6812E-13	9.34361E-12
ENSG00000165271	NOL6	-0.76	7.68518E-13	9.34361E-12
ENSG00000159267	HLCS	-0.90	8.00198E-13	9.72286E-12
ENSG00000156515	HK1	0.53	8.00936E-13	9.72593E-12
ENSG00000134574	DDB2	-0.76	8.11897E-13	9.85306E-12
ENSG00000159388	BTG2	-0.83	8.3018E-13	1.00688E-11
ENSG00000163701	IL17RE	1.31	8.37985E-13	1.01573E-11
ENSG00000131323	TRAF3	-0.75	8.52947E-13	1.03324E-11
ENSG00000186063	AIDA	0.79	8.77498E-13	1.06234E-11
ENSG00000197299	BLM	-1.05	8.9052E-13	1.07745E-11
ENSG00000067334	DNTTIP2	0.70	8.95722E-13	1.08309E-11

ENSG00000177225	GATD1	-0.70	9.05221E-13	1.09392E-11
ENSG00000163536	SERPINI1	1.50	9.41772E-13	1.13672E-11
ENSG00000112787	FBRSL1	-0.67	9.41774E-13	1.13672E-11
ENSG00000085491	SLC25A24	0.76	9.54144E-13	1.15095E-11
ENSG00000076382	SPAG5	0.59	9.57314E-13	1.15408E-11
ENSG00000025708	TYMP	-0.87	9.73013E-13	1.1723E-11
ENSG00000171604	CXXC5	-0.82	9.91312E-13	1.19363E-11
ENSG00000148450	MSRB2	-1.46	9.98505E-13	1.20157E-11
ENSG00000173457	PPP1R14B	0.54	1.00282E-12	1.20604E-11
ENSG00000082146	STRADB	-1.12	1.00702E-12	1.21036E-11
ENSG00000170852	KBTBD2	0.82	1.03047E-12	1.23781E-11
ENSG00000171302	CANT1	-0.57	1.03129E-12	1.23804E-11
ENSG00000137288	UQCC2	-0.81	1.08826E-12	1.30565E-11
ENSG00000123636	BAZ2B	1.12	1.09391E-12	1.31165E-11
ENSG00000166225	FRS2	0.97	1.11253E-12	1.33317E-11
ENSG00000186111	PIP5K1C	-0.81	1.15066E-12	1.37804E-11
ENSG00000235106	BRD3OS	-1.17	1.16931E-12	1.39953E-11
ENSG00000157456	CCNB2	0.55	1.21478E-12	1.45309E-11
ENSG00000124191	TOX2	-0.88	1.23407E-12	1.47505E-11
ENSG00000130304	SLC27A1	-0.91	1.23461E-12	1.47505E-11
ENSG00000128609	NDUFA5	-0.82	1.33469E-12	1.59367E-11
ENSG00000185122	HSF1	-0.67	1.35559E-12	1.61765E-11
ENSG00000058729	RIOK2	-1.27	1.51455E-12	1.80627E-11
ENSG00000027001	MIPEP	-0.97	1.53379E-12	1.82812E-11
ENSG00000187608	ISG15	0.65	1.54627E-12	1.8419E-11
ENSG00000119953	SMNDC1	0.61	1.57083E-12	1.87005E-11
ENSG00000210196	MT-TP	-1.19	1.57258E-12	1.87101E-11
ENSG00000152056	AP1S3	0.64	1.57425E-12	1.87189E-11
ENSG00000008441	NFIX	-0.82	1.58723E-12	1.88621E-11
ENSG00000106484	MEST	0.81	1.62639E-12	1.93159E-11
ENSG00000198689	SLC9A6	0.89	1.6453E-12	1.95277E-11
ENSG00000269343	ZNF587B	-1.84	1.64617E-12	1.95277E-11
ENSG00000010072	SPRTN	1.10	1.68076E-12	1.99262E-11
ENSG00000204160	ZDHHC18	-0.73	1.75347E-12	2.07759E-11
ENSG00000188677	PARVB	-1.03	1.77483E-12	2.10165E-11
ENSG00000130522	JUND	-1.29	1.78222E-12	2.10916E-11
ENSG00000254166	CASC19	-1.31	1.82294E-12	2.15607E-11
ENSG00000198492	YTHDF2	0.53	1.85094E-12	2.18789E-11
ENSG00000117640	MTFR1L	-0.93	1.86153E-12	2.19911E-11
ENSG00000253368	TRNP1	-0.68	1.86834E-12	2.20585E-11
ENSG00000005513	SOX8	1.34	1.9073E-12	2.25052E-11
ENSG00000038427	VCAN	0.61	1.91237E-12	2.25518E-11
ENSG00000143458	GABPB2	-1.12	1.94459E-12	2.29182E-11
ENSG00000173575	CHD2	0.57	1.95439E-12	2.30201E-11
ENSG00000169612	RAMAC	1.08	1.95712E-12	2.30387E-11
ENSG00000198843	SELENOT	0.54	1.9593E-12	2.30509E-11

ENSG00000088682	COQ9	-0.84	2.00406E-12	2.35636E-11
ENSG00000151466	SCLT1	1.13	2.07406E-12	2.43723E-11
ENSG00000090863	GLG1	0.42	2.08043E-12	2.44328E-11
ENSG00000140263	SORD	-0.52	2.11477E-12	2.48216E-11
ENSG00000101745	ANKRD12	1.08	2.11986E-12	2.48667E-11
ENSG00000153936	HS2ST1	-1.00	2.17008E-12	2.54275E-11
ENSG00000175220	ARHGAP1	-0.77	2.17021E-12	2.54275E-11
ENSG00000120075	HOXB5	-1.31	2.17239E-12	2.54382E-11
ENSG00000104381	GDAP1	-1.29	2.17731E-12	2.54808E-11
ENSG00000161057	PSMC2	0.51	2.19013E-12	2.56159E-11
ENSG00000135454	B4GALNT1	0.69	2.21088E-12	2.58436E-11
ENSG00000168488	ATXN2L	-0.46	2.21598E-12	2.5888E-11
ENSG00000137563	GGH	0.60	2.27263E-12	2.65211E-11
ENSG00000111412	C12orf49	0.61	2.27282E-12	2.65211E-11
ENSG00000075240	GRAMD4	-0.99	2.2952E-12	2.67666E-11
ENSG00000160469	BRSK1	0.97	2.31452E-12	2.69762E-11
ENSG00000148339	SLC25A25	0.76	2.35066E-12	2.73815E-11
ENSG00000110013	SIAE	-0.94	2.39759E-12	2.79119E-11
ENSG00000033327	GAB2	1.37	2.40926E-12	2.80314E-11
ENSG00000113119	TMCO6	-1.29	2.46953E-12	2.8716E-11
ENSG00000137807	KIF23	0.53	2.4999E-12	2.90523E-11
ENSG00000139921	TMX1	-0.62	2.51274E-12	2.91845E-11
ENSG00000178814	OPLAH	-1.02	2.51957E-12	2.92469E-11
ENSG00000072506	HSD17B10	-0.67	2.52876E-12	2.93366E-11
ENSG00000121064	SCPEP1	0.75	2.59935E-12	3.0138E-11
ENSG00000167977	KCTD5	0.60	2.63888E-12	3.05786E-11
ENSG00000173559	NABP1	0.75	2.6464E-12	3.0648E-11
ENSG00000197077	KIAA1671	-0.67	2.6923E-12	3.11616E-11
ENSG00000151092	NGLY1	-1.12	2.69535E-12	3.11788E-11
ENSG00000109685	NSD2	-0.49	2.73398E-12	3.16073E-11
ENSG00000111707	SUDS3	-0.65	2.73721E-12	3.16265E-11
ENSG00000169047	IRS1	-0.66	2.79719E-12	3.23009E-11
ENSG00000065268	WDR18	-0.66	2.80221E-12	3.23402E-11
ENSG00000160285	LSS	-0.57	2.87165E-12	3.31224E-11
ENSG00000086232	EIF2AK1	-0.47	2.87866E-12	3.31842E-11
ENSG00000161647	MPP3	0.93	2.90459E-12	3.34638E-11
ENSG00000165046	LETM2	0.88	2.96122E-12	3.40967E-11
ENSG00000160326	SLC2A6	1.01	2.99396E-12	3.44538E-11
ENSG00000141367	CLTC	0.48	3.04367E-12	3.50057E-11
ENSG00000285106	AC016831.6	1.65	3.13033E-12	3.59818E-11
ENSG00000175215	CTDSP2	-0.52	3.18858E-12	3.66302E-11
ENSG00000100316	RPL3	-0.45	3.21918E-12	3.69606E-11
ENSG00000169783	LINGO1	-1.11	3.22983E-12	3.70615E-11
ENSG00000175662	TOM1L2	-0.96	3.2505E-12	3.72773E-11
ENSG00000106070	GRB10	-0.70	3.25338E-12	3.72891E-11
ENSG00000101670	LIPG	1.18	3.3911E-12	3.88453E-11

ENSG00000144283	PKP4	-0.62	3.39686E-12	3.8889E-11
ENSG00000115107	STEAP3	-0.81	3.42465E-12	3.91848E-11
ENSG00000008710	PKD1	-0.60	3.44719E-12	3.94201E-11
ENSG00000008294	SPAG9	0.60	3.47117E-12	3.96717E-11
ENSG00000203499	IQANK1	-0.86	3.49217E-12	3.98889E-11
ENSG00000110048	OSBP	0.50	3.61758E-12	4.12977E-11
ENSG00000164674	SYTL3	-0.58	3.62602E-12	4.13705E-11
ENSG00000275216	AL161431.1	-0.77	3.70005E-12	4.21911E-11
ENSG00000183496	MEX3B	1.89	3.71587E-12	4.23474E-11
ENSG00000168936	TMEM129	-1.07	3.73874E-12	4.25838E-11
ENSG00000185989	RASA3	0.87	3.77133E-12	4.29305E-11
ENSG00000140044	JDP2	-0.79	3.95567E-12	4.50033E-11
ENSG00000163931	TKT	-0.49	4.02147E-12	4.5726E-11
ENSG00000111786	SRSF9	0.45	4.11579E-12	4.67718E-11
ENSG00000154743	TSEN2	-1.29	4.39992E-12	4.99724E-11
ENSG00000174500	GCSAM	1.96	4.43945E-12	5.03926E-11
ENSG00000117408	IPO13	-0.91	4.52838E-12	5.1373E-11
ENSG00000064490	RFXANK	-0.83	4.58517E-12	5.19877E-11
ENSG00000185245	GP1BA	-3.20	4.59192E-12	5.20348E-11
ENSG00000112159	MDN1	-0.61	4.65294E-12	5.26964E-11
ENSG00000156990	RPUSD3	-0.90	4.69548E-12	5.31482E-11
ENSG00000196689	TRPV1	-1.76	4.75367E-12	5.37764E-11
ENSG00000099810	MTAP	-0.52	4.77842E-12	5.40125E-11
ENSG00000115365	LANCL1	-0.58	4.77994E-12	5.40125E-11
ENSG00000101782	RIOK3	0.66	4.79105E-12	5.41075E-11
ENSG00000145337	PYURF	-0.65	4.858E-12	5.48327E-11
ENSG00000100504	PYGL	-0.46	4.92445E-12	5.55514E-11
ENSG00000138593	SECISBP2L	0.87	5.01405E-12	5.65302E-11
ENSG00000134590	RTL8C	-0.77	5.03139E-12	5.66937E-11
ENSG00000125731	SH2D3A	-0.71	5.10542E-12	5.74956E-11
ENSG00000124882	EREG	0.54	5.23043E-12	5.88703E-11
ENSG00000124767	GLO1	0.46	5.23558E-12	5.88951E-11
ENSG00000241839	PLEKHO2	0.96	5.28058E-12	5.93443E-11
ENSG00000118894	EEF2KMT	-1.08	5.28144E-12	5.93443E-11
ENSG00000175455	CCDC14	-0.59	5.4299E-12	6.09782E-11
ENSG00000235173	HGH1	-0.91	5.46467E-12	6.13343E-11
ENSG00000108671	PSMD11	0.44	5.47148E-12	6.13762E-11
ENSG00000182022	CHST15	-0.60	5.5233E-12	6.19228E-11
ENSG00000119888	EPCAM	0.46	5.56202E-12	6.2322E-11
ENSG00000104081	BMF	2.27	5.57645E-12	6.24488E-11
ENSG00000178896	EXOSC4	-0.85	5.70299E-12	6.38301E-11
ENSG00000120875	DUSP4	0.45	5.77099E-12	6.45551E-11
ENSG00000157191	NECAP2	0.72	5.82838E-12	6.51606E-11
ENSG00000164830	OXR1	0.82	5.86974E-12	6.55864E-11
ENSG00000167670	CHAF1A	-0.53	5.87439E-12	6.56017E-11
ENSG00000165171	METTL27	-1.33	5.91216E-12	6.59867E-11

ENSG00000114812	VIPR1	-1.97	5.92513E-12	6.60946E-11
ENSG00000197971	MBP	-0.87	5.94127E-12	6.62377E-11
ENSG00000186205	MTARC1	-1.21	5.9595E-12	6.6404E-11
ENSG00000170502	NUDT9	0.92	6.03416E-12	6.71985E-11
ENSG00000167767	KRT80	-1.13	6.13392E-12	6.82715E-11
ENSG00000152944	MED21	0.89	6.3088E-12	7.01788E-11
ENSG00000167524	RSKR	-2.24	6.3389E-12	7.04745E-11
ENSG00000189337	KAZN	-1.04	6.36118E-12	7.06829E-11
ENSG00000277791	PSMB3	-0.53	6.39372E-12	7.10051E-11
ENSG00000157869	RAB28	-1.21	6.49351E-12	7.20733E-11
ENSG00000188130	MAPK12	-0.63	6.525E-12	7.23827E-11
ENSG00000133069	TMCC2	1.79	6.52994E-12	7.23974E-11
ENSG00000141933	TPGS1	1.32	6.53668E-12	7.24319E-11
ENSG00000143375	CGN	0.78	6.5937E-12	7.30233E-11
ENSG00000179526	SHARPIN	-0.86	6.61625E-12	7.32325E-11
ENSG00000103995	CEP152	-1.02	6.6217E-12	7.32524E-11
ENSG00000111011	RSRC2	0.63	6.65006E-12	7.35254E-11
ENSG00000152359	POC5	1.10	6.68068E-12	7.38232E-11
ENSG00000102870	ZNF629	-0.84	6.95179E-12	7.67766E-11
ENSG00000179364	PACS2	-0.76	7.44457E-12	8.21735E-11
ENSG00000129810	SGO1	0.91	7.51185E-12	8.28705E-11
ENSG00000152700	SAR1B	0.68	7.62731E-12	8.4098E-11
ENSG00000175548	ALG10B	-1.42	7.63563E-12	8.41433E-11
ENSG00000227473	TSSK5P	-3.33	7.70501E-12	8.48611E-11
ENSG00000124214	STAU1	-0.49	7.71603E-12	8.49103E-11
ENSG00000154447	SH3RF1	0.58	7.71796E-12	8.49103E-11
ENSG00000262001	DLGAP1-AS2	-1.18	7.82074E-12	8.59938E-11
ENSG00000113763	UNC5A	-1.56	7.87181E-12	8.65077E-11
ENSG00000162613	FUBP1	-0.60	7.90372E-12	8.67776E-11
ENSG00000196584	XRCC2	-0.96	7.90504E-12	8.67776E-11
ENSG00000182700	IGIP	-1.82	7.9116E-12	8.6802E-11
ENSG00000197766	CFD	0.73	7.92774E-12	8.69314E-11
ENSG00000186184	POLR1D	-0.56	8.01155E-12	8.78023E-11
ENSG00000159720	ATP6V0D1	-0.80	8.06294E-12	8.83172E-11
ENSG00000106609	TMEM248	-0.57	8.08535E-12	8.85143E-11
ENSG00000134077	THUMPD3	0.65	8.50944E-12	9.3106E-11
ENSG00000070814	TCOF1	-0.50	8.52906E-12	9.32697E-11
ENSG00000119965	C10orf88	0.89	8.62217E-12	9.42364E-11
ENSG00000004961	HCCS	0.65	8.76182E-12	9.57104E-11
ENSG00000130204	TOMM40	-0.52	9.01543E-12	9.8427E-11
ENSG00000196227	FAM217B	1.18	9.10186E-12	9.93165E-11
ENSG00000163950	SLBP	-0.51	9.11596E-12	9.93898E-11
ENSG00000094804	CDC6	-0.52	9.11851E-12	9.93898E-11
ENSG00000261801	LOXL1-AS1	-1.42	9.2732E-12	1.01021E-10
ENSG00000133816	MICAL2	-0.79	9.34561E-12	1.01754E-10
ENSG00000034510	TMSB10	0.52	9.40966E-12	1.02396E-10

ENSG00000075826	SEC31B	-1.12	9.47143E-12	1.03012E-10
ENSG00000125351	UPF3B	0.75	9.75598E-12	1.06049E-10
ENSG00000144468	RHBDD1	-0.70	9.85751E-12	1.07095E-10
ENSG00000143774	GUK1	0.56	1.04753E-11	1.13745E-10
ENSG00000064607	SUGP2	0.50	1.05454E-11	1.14444E-10
ENSG00000110066	KMT5B	0.75	1.05965E-11	1.14936E-10
ENSG00000144231	POLR2D	-0.57	1.06348E-11	1.15289E-10
ENSG00000110237	ARHGEF17	-0.85	1.06777E-11	1.15691E-10
ENSG00000065621	GSTO2	-0.55	1.08971E-11	1.18005E-10
ENSG00000122694	GLIPR2	-1.40	1.10826E-11	1.19949E-10
ENSG00000196235	SUPT5H	0.47	1.12189E-11	1.21358E-10
ENSG00000067082	KLF6	0.52	1.12692E-11	1.21774E-10
ENSG00000166619	BLCAP	0.64	1.12695E-11	1.21774E-10
ENSG00000185745	IFIT1	1.93	1.1291E-11	1.2194E-10
ENSG00000074590	NUAK1	0.87	1.1603E-11	1.25242E-10
ENSG00000124116	WFDC3	2.61	1.1697E-11	1.26188E-10
ENSG00000181038	METTL23	-0.84	1.17647E-11	1.26851E-10
ENSG00000253293	HOXA10	0.91	1.18402E-11	1.27597E-10
ENSG00000179051	RCC2	0.39	1.19232E-11	1.28422E-10
ENSG00000163877	SNIP1	0.79	1.21789E-11	1.31105E-10
ENSG00000112234	FBXL4	-1.32	1.23024E-11	1.32363E-10
ENSG00000196542	SPTSSB	2.29	1.23136E-11	1.32413E-10
ENSG00000078304	PPP2R5C	0.49	1.23407E-11	1.32633E-10
ENSG00000143862	ARL8A	0.76	1.26527E-11	1.35913E-10
ENSG00000204335	SP5	2.12	1.29904E-11	1.39466E-10
ENSG00000130703	OSBPL2	0.69	1.30067E-11	1.39565E-10
ENSG00000134369	NAV1	-0.79	1.32905E-11	1.42534E-10
ENSG00000133135	RNF128	1.47	1.33044E-11	1.42607E-10
ENSG00000173915	ATP5MD	0.48	1.34607E-11	1.44205E-10
ENSG00000106028	SSBP1	0.58	1.38527E-11	1.48326E-10
ENSG00000026508	CD44	-0.43	1.38656E-11	1.48384E-10
ENSG00000173868	PHOSPHO1	1.83	1.39149E-11	1.48832E-10
ENSG00000172183	ISG20	1.39	1.41267E-11	1.51017E-10
ENSG00000071537	SEL1L	0.63	1.42143E-11	1.51872E-10
ENSG00000166822	TMEM170A	-0.76	1.42859E-11	1.52556E-10
ENSG00000117308	GALE	-0.55	1.43096E-11	1.52727E-10
ENSG00000180398	MCFD2	0.51	1.44739E-11	1.54399E-10
ENSG00000132623	ANKEF1	0.80	1.46026E-11	1.55688E-10
ENSG00000071626	DAZAP1	-0.45	1.51655E-11	1.61603E-10
ENSG00000180901	KCTD2	-0.66	1.54152E-11	1.64177E-10
ENSG00000184281	TSSC4	0.75	1.55491E-11	1.65515E-10
ENSG00000106144	CASP2	-0.55	1.56088E-11	1.66063E-10
ENSG00000187688	TRPV2	2.13	1.57342E-11	1.67308E-10
ENSG00000168040	FADD	0.79	1.57519E-11	1.67406E-10
ENSG00000127586	CHTF18	-0.61	1.58056E-11	1.67888E-10
ENSG00000145375	SPATA5	0.90	1.59741E-11	1.69588E-10

ENSG00000167513	CDT1	-0.50	1.65262E-11	1.75357E-10
ENSG00000123600	METTL8	-1.08	1.65981E-11	1.75948E-10
ENSG00000237330	RNF223	1.59	1.65995E-11	1.75948E-10
ENSG00000062822	POLD1	-0.57	1.6634E-11	1.7622E-10
ENSG00000165389	SPTSSA	-0.66	1.66853E-11	1.7667E-10
ENSG00000136603	SKIL	1.09	1.67466E-11	1.77226E-10
ENSG00000066279	ASPM	0.68	1.70185E-11	1.80007E-10
ENSG00000180182	MED14	-0.64	1.70447E-11	1.8019E-10
ENSG00000115112	TFCP2L1	-0.93	1.73795E-11	1.83632E-10
ENSG00000100564	PIGH	-1.16	1.75695E-11	1.85541E-10
ENSG00000143845	ETNK2	-0.98	1.78755E-11	1.88673E-10
ENSG00000114315	HES1	-1.09	1.80293E-11	1.90197E-10
ENSG00000281649	EBLN3P	-0.72	1.8124E-11	1.91094E-10
ENSG00000157540	DYRK1A	0.70	1.84118E-11	1.94027E-10
ENSG00000240972	MIF	0.54	1.9007E-11	2.00194E-10
ENSG00000136492	BRIP1	-0.97	1.91213E-11	2.01292E-10
ENSG00000136888	ATP6V1G1	0.52	1.9471E-11	2.04866E-10
ENSG00000103932	RPAP1	-0.78	1.96396E-11	2.0653E-10
ENSG00000112655	PTK7	-0.54	1.97224E-11	2.07292E-10
ENSG00000108963	DPH1	-1.11	2.00099E-11	2.10204E-10
ENSG00000138069	RAB1A	0.43	2.00996E-11	2.11035E-10
ENSG00000183762	KREMEN1	-0.71	2.078E-11	2.18065E-10
ENSG00000224870	MRPL20-AS1	-0.96	2.14817E-11	2.2531E-10
ENSG00000255529	POLR2M	-0.99	2.16397E-11	2.26848E-10
ENSG00000168394	TAP1	0.68	2.18139E-11	2.28555E-10
ENSG00000104825	NFKBIB	0.65	2.18833E-11	2.29162E-10
ENSG00000014164	ZC3H3	-0.86	2.19912E-11	2.30172E-10
ENSG00000163584	RPL22L1	-0.46	2.21423E-11	2.31631E-10
ENSG00000064545	TMEM161A	-0.70	2.22392E-11	2.32524E-10
ENSG00000073849	ST6GAL1	-1.19	2.22716E-11	2.32741E-10
ENSG00000183309	ZNF623	-0.82	2.32925E-11	2.43283E-10
ENSG00000187535	IFT140	-0.92	2.34435E-11	2.44732E-10
ENSG00000140319	SRP14	0.45	2.35962E-11	2.46198E-10
ENSG00000125817	CENPB	-0.55	2.37042E-11	2.47195E-10
ENSG00000082515	MRPL22	0.66	2.39254E-11	2.49373E-10
ENSG00000025434	NR1H3	-1.29	2.3944E-11	2.49436E-10
ENSG00000168303	MPLKIP	-0.86	2.442E-11	2.54262E-10
ENSG00000171084	FAM86JP	-2.14	2.48046E-11	2.58133E-10
ENSG00000100403	ZC3H7B	-0.55	2.50969E-11	2.61039E-10
ENSG00000103042	SLC38A7	-1.23	2.56201E-11	2.66342E-10
ENSG00000129667	RHBDF2	-0.83	2.62608E-11	2.72862E-10
ENSG00000198833	UBE2J1	-0.78	2.62839E-11	2.72959E-10
ENSG00000103647	CORO2B	1.66	2.64967E-11	2.75027E-10
ENSG00000198954	KIFBP	-0.79	2.7026E-11	2.80375E-10
ENSG00000171497	PPID	0.59	2.79435E-11	2.89744E-10
ENSG00000143933	CALM2	0.45	2.81784E-11	2.92028E-10

ENSG00000087586	AURKA	0.53	2.87909E-11	2.98221E-10
ENSG00000138078	PREPL	-0.80	2.90864E-11	3.01127E-10
ENSG00000235655	H3P6	0.76	2.91616E-11	3.01749E-10
ENSG00000268858	AL118506.1	1.60	2.92403E-11	3.02407E-10
ENSG00000182831	C16orf72	0.54	2.93449E-11	3.03332E-10
ENSG00000125166	GOT2	-0.45	2.94628E-11	3.04393E-10
ENSG00000100380	ST13	-0.42	3.0194E-11	3.11787E-10
ENSG00000167110	GOLGA2	0.50	3.04249E-11	3.14009E-10
ENSG00000136807	CDK9	-0.71	3.05006E-11	3.14628E-10
ENSG00000044446	PHKA2	-0.94	3.06371E-11	3.15873E-10
ENSG00000183760	ACP7	1.35	3.06724E-11	3.16074E-10
ENSG00000111725	PRKAB1	0.79	3.07099E-11	3.16298E-10
ENSG00000022277	RTF2	0.52	3.15326E-11	3.24514E-10
ENSG00000116990	MYCL	-1.33	3.15401E-11	3.24514E-10
ENSG00000255737	AGAP2-AS1	-1.54	3.24906E-11	3.33955E-10
ENSG00000101557	USP14	0.55	3.2491E-11	3.33955E-10
ENSG00000120913	PDLIM2	-1.08	3.31996E-11	3.41063E-10
ENSG00000163435	ELF3	-0.73	3.34987E-11	3.43959E-10
ENSG00000132964	CDK8	0.85	3.40785E-11	3.49732E-10
ENSG00000155366	RHOC	0.50	3.62061E-11	3.71377E-10
ENSG00000165895	ARHGAP42	0.97	3.68679E-11	3.77971E-10
ENSG00000080561	MID2	1.59	3.76626E-11	3.85921E-10
ENSG00000109099	PMP22	0.59	3.81018E-11	3.90222E-10
ENSG00000166145	SPINT1	0.52	3.82323E-11	3.91358E-10
ENSG00000205155	PSENEN	0.68	3.86066E-11	3.94971E-10
ENSG00000166478	ZNF143	1.10	3.86247E-11	3.94971E-10
ENSG00000147255	IGSF1	2.23	3.89913E-11	3.98515E-10
ENSG00000113583	C5orf15	-0.64	3.94572E-11	4.03072E-10
ENSG00000133872	SARAF	0.47	3.98002E-11	4.06368E-10
ENSG00000204308	RNF5	0.60	4.11562E-11	4.19998E-10
ENSG00000187840	EIF4EBP1	-0.53	4.21183E-11	4.29598E-10
ENSG00000120942	UBIAD1	-0.94	4.26532E-11	4.34833E-10
ENSG00000106399	RPA3	-0.59	4.26992E-11	4.3508E-10
ENSG00000121060	TRIM25	-0.45	4.27295E-11	4.35167E-10
ENSG00000172890	NADSYN1	-0.83	4.28453E-11	4.36124E-10
ENSG00000094631	HDAC6	-0.69	4.29939E-11	4.37414E-10
ENSG00000185222	TCEAL9	0.71	4.31029E-11	4.38109E-10
ENSG00000100075	SLC25A1	-0.58	4.31059E-11	4.38109E-10
ENSG00000116337	AMPD2	0.65	4.38189E-11	4.45129E-10
ENSG00000130939	UBE4B	-0.60	4.38996E-11	4.45722E-10
ENSG00000188643	S100A16	0.47	4.42819E-11	4.49376E-10
ENSG00000151239	TWF1	0.52	4.45331E-11	4.51696E-10
ENSG00000183779	ZNF703	1.31	4.46094E-11	4.52241E-10
ENSG00000197140	ADAM32	2.17	4.48007E-11	4.5395E-10
ENSG00000085840	ORC1	-0.79	4.5232E-11	4.58089E-10
ENSG00000130669	PAK4	-0.62	4.55792E-11	4.61371E-10

ENSG00000171703	TCEA2	-1.04	4.8745E-11	4.93167E-10
ENSG00000268205	AC005261.1	-0.77	4.8972E-11	4.95214E-10
ENSG00000117399	CDC20	0.45	4.90285E-11	4.95534E-10
ENSG00000226314	ZNF192P1	2.33	4.93385E-11	4.98416E-10
ENSG00000148426	PROSER2	-0.94	4.99058E-11	5.03892E-10
ENSG00000126247	CAPNS1	0.46	5.10724E-11	5.15412E-10
ENSG00000095066	HOOK2	-0.70	5.15189E-11	5.19656E-10
ENSG00000166856	GPR182	3.21	5.21105E-11	5.25357E-10
ENSG00000124380	SNRNP27	0.72	5.39525E-11	5.43654E-10
ENSG00000182670	TTC3	0.56	5.42117E-11	5.45991E-10
ENSG00000144118	RALB	0.56	5.56404E-11	5.60099E-10
ENSG00000259868	AL163952.1	3.26	5.60426E-11	5.63863E-10
ENSG00000197576	HOXA4	1.97	5.65064E-11	5.68244E-10
ENSG00000260342	AC138811.2	4.88	5.67546E-11	5.70454E-10
ENSG00000110911	SLC11A2	-0.69	6.0049E-11	6.03263E-10
ENSG00000108669	CYTH1	0.65	6.21343E-11	6.239E-10
ENSG00000105829	BET1	-1.10	6.26902E-11	6.29166E-10
ENSG00000137267	TUBB2A	0.79	6.34831E-11	6.36804E-10
ENSG00000115875	SRSF7	-0.46	6.36116E-11	6.37773E-10
ENSG00000162302	RPS6KA4	-0.59	6.36898E-11	6.38237E-10
ENSG00000181924	COA4	-0.61	6.50913E-11	6.51955E-10
ENSG00000279348	AC012513.3	-4.86	6.54553E-11	6.55274E-10
ENSG00000167106	FAM102A	-0.74	6.61595E-11	6.61992E-10
ENSG00000127666	TICAM1	0.85	6.76354E-11	6.76422E-10
ENSG00000167881	SRP68	0.43	6.77483E-11	6.77213E-10
ENSG00000119632	IFI27L2	0.94	6.88782E-11	6.88163E-10
ENSG00000148606	POLR3A	0.55	6.90786E-11	6.89821E-10
ENSG00000100519	PSMC6	0.60	6.92752E-11	6.91439E-10
ENSG00000113575	PPP2CA	0.46	7.08231E-11	7.06536E-10
ENSG00000164171	ITGA2	0.90	7.21688E-11	7.19602E-10
ENSG00000143549	TPM3	0.40	7.24644E-11	7.2219E-10
ENSG00000196652	ZKSCAN5	0.85	7.31152E-11	7.28313E-10
ENSG00000197324	LRP10	0.52	7.44863E-11	7.41601E-10
ENSG00000008083	JARID2	0.76	7.5745E-11	7.53758E-10
ENSG00000196547	MAN2A2	-0.72	7.62478E-11	7.58385E-10
ENSG00000167645	YIF1B	0.62	7.69008E-11	7.645E-10
ENSG00000108523	RNF167	0.52	7.71119E-11	7.66218E-10
ENSG00000140259	MFAP1	0.59	7.73679E-11	7.6838E-10
ENSG00000230124	ACBD6	0.65	7.74561E-11	7.68845E-10
ENSG00000218739	CEBPZOS	-0.78	7.74915E-11	7.68845E-10
ENSG00000158717	RNF166	-0.75	7.88889E-11	7.82322E-10
ENSG00000173727	AP000769.1	1.15	7.99478E-11	7.92429E-10
ENSG00000151726	ACSL1	0.89	8.00809E-11	7.93355E-10
ENSG00000085662	AKR1B1	-0.61	8.03754E-11	7.95879E-10
ENSG00000135439	AGAP2	0.76	8.09972E-11	8.0164E-10
ENSG00000204406	MBD5	1.11	8.12432E-11	8.03677E-10

ENSG00000124151	NCOA3	0.65	8.14251E-11	8.05079E-10
ENSG00000198160	MIER1	0.72	8.17733E-11	8.08122E-10
ENSG00000113161	HMGCR	0.52	8.31315E-11	8.21139E-10
ENSG00000166897	ELFN2	-0.55	8.45986E-11	8.35218E-10
ENSG00000145916	RMND5B	-0.78	8.61878E-11	8.50489E-10
ENSG00000142798	HSPG2	-1.48	8.69342E-11	8.57431E-10
ENSG00000188931	CFAP126	-1.88	8.81556E-11	8.69049E-10
ENSG00000141854	MISP3	1.26	8.85455E-11	8.72463E-10
ENSG00000143183	TMCO1	0.57	8.89694E-11	8.76027E-10
ENSG00000111667	USP5	0.41	8.89947E-11	8.76027E-10
ENSG00000145730	PAM	0.41	9.0058E-11	8.86057E-10
ENSG00000146830	GIGYF1	-0.60	9.12641E-11	8.97483E-10
ENSG00000164754	RAD21	0.38	9.32511E-11	9.16572E-10
ENSG00000100271	TTLL1	-2.17	9.3492E-11	9.18489E-10
ENSG00000134070	IRAK2	1.44	9.36978E-11	9.20059E-10
ENSG00000112977	DAP	0.55	9.6937E-11	9.51399E-10
ENSG00000166170	BAG5	-0.69	9.70046E-11	9.51596E-10
ENSG00000205060	SLC35B4	-0.81	9.79148E-11	9.60055E-10
ENSG00000135114	OASL	1.91	1.00993E-10	9.89749E-10
ENSG00000106789	CORO2A	-1.03	1.01672E-10	9.9592E-10
ENSG00000106993	CDC37L1	0.89	1.01813E-10	9.96812E-10
ENSG00000030582	GRN	0.52	1.02212E-10	1.00023E-09
ENSG00000144677	CTDSPL	-0.71	1.03658E-10	1.01388E-09
ENSG00000095906	NUBP2	-0.66	1.04009E-10	1.01682E-09
ENSG00000155850	SLC26A2	-1.14	1.05106E-10	1.02704E-09
ENSG00000135953	MFSD9	0.90	1.05811E-10	1.03343E-09
ENSG00000154639	CXADR	0.65	1.0592E-10	1.03399E-09
ENSG00000163349	HIPK1	-0.66	1.06993E-10	1.04395E-09
ENSG00000134222	PSRC1	0.63	1.09099E-10	1.06398E-09
ENSG00000165449	SLC16A9	-1.26	1.10072E-10	1.07295E-09
ENSG00000131871	SELENOS	0.62	1.11839E-10	1.08964E-09
ENSG00000070610	GBA2	-0.58	1.12029E-10	1.09096E-09
ENSG00000100211	CBY1	-0.86	1.12451E-10	1.09453E-09
ENSG00000007312	CD79B	2.79	1.14431E-10	1.11327E-09
ENSG00000185721	DRG1	0.49	1.14715E-10	1.11549E-09
ENSG00000103197	TSC2	-0.70	1.14906E-10	1.11681E-09
ENSG00000073350	LLGL2	-0.55	1.16073E-10	1.1276E-09
ENSG00000146083	RNF44	-0.90	1.20417E-10	1.16924E-09
ENSG00000100311	PDGFB	-0.72	1.24852E-10	1.21171E-09
ENSG00000139746	RBM26	0.52	1.26965E-10	1.23162E-09
ENSG00000155959	VBP1	0.52	1.30926E-10	1.26943E-09
ENSG00000139645	ANKRD52	0.46	1.33378E-10	1.29257E-09
ENSG00000038382	TRIO	0.61	1.33566E-10	1.29377E-09
ENSG00000112742	TTK	0.64	1.35907E-10	1.31556E-09
ENSG00000103248	MTHFSD	-0.99	1.35948E-10	1.31556E-09
ENSG00000213672	NCKIPSD	-0.76	1.37049E-10	1.32558E-09

ENSG00000135655	USP15	0.71	1.37435E-10	1.32867E-09
ENSG00000101265	RASSF2	1.01	1.37841E-10	1.33195E-09
ENSG00000177879	AP3S1	-0.48	1.39519E-10	1.34751E-09
ENSG00000147224	PRPS1	0.45	1.42779E-10	1.37834E-09
ENSG00000125686	MED1	0.53	1.45657E-10	1.40544E-09
ENSG00000184602	SNN	0.59	1.46069E-10	1.40874E-09
ENSG00000123179	EBPL	-0.71	1.48549E-10	1.43197E-09
ENSG00000168672	LRATD2	-0.69	1.50574E-10	1.45079E-09
ENSG00000228474	OST4	0.57	1.5069E-10	1.45121E-09
ENSG00000075142	SRI	-0.56	1.51749E-10	1.4607E-09
ENSG00000007202	KIAA0100	-0.50	1.51854E-10	1.46101E-09
ENSG00000080854	IGSF9B	-0.86	1.53467E-10	1.47582E-09
ENSG00000197958	RPL12	-0.42	1.56623E-10	1.50544E-09
ENSG00000186260	MRTFB	-0.82	1.5867E-10	1.52439E-09
ENSG00000224536	AC096677.1	-2.71	1.59368E-10	1.53037E-09
ENSG00000162073	PAQR4	-0.80	1.61033E-10	1.54561E-09
ENSG00000101844	ATG4A	1.26	1.61743E-10	1.55168E-09
ENSG00000103876	FAH	-1.04	1.63953E-10	1.57213E-09
ENSG00000255112	CHMP1B	-0.81	1.67651E-10	1.60681E-09
ENSG00000196670	ZFP62	-1.06	1.68464E-10	1.61383E-09
ENSG00000205765	C5orf51	-0.85	1.6931E-10	1.62116E-09
ENSG00000133243	BTBD2	-1.19	1.69626E-10	1.62341E-09
ENSG00000161677	JOSD2	-1.00	1.7034E-10	1.62946E-09
ENSG00000163376	KBTBD8	1.75	1.73091E-10	1.65499E-09
ENSG00000143147	GPR161	-0.99	1.74352E-10	1.66626E-09
ENSG00000182827	ACBD3	0.66	1.77229E-10	1.69294E-09
ENSG00000012963	UBR7	-0.66	1.79759E-10	1.71629E-09
ENSG00000240476	LINC00973	1.41	1.79869E-10	1.71652E-09
ENSG00000161243	FBXO27	-1.26	1.81004E-10	1.72652E-09
ENSG00000152684	PELO	0.62	1.83474E-10	1.74925E-09
ENSG00000183137	CEP57L1	1.14	1.85707E-10	1.7697E-09
ENSG00000142634	EFHD2	0.52	1.87289E-10	1.78393E-09
ENSG00000137947	GTF2B	0.82	1.88681E-10	1.79633E-09
ENSG00000188997	KCTD21	1.38	1.96302E-10	1.868E-09
ENSG00000108953	YWHAE	0.36	1.97138E-10	1.87497E-09
ENSG00000120868	APAF1	0.78	1.97222E-10	1.87497E-09
ENSG00000247077	PGAM5	-0.47	1.98358E-10	1.88487E-09
ENSG00000154845	PPP4R1	0.55	1.98562E-10	1.88591E-09
ENSG00000276234	TADA2A	-0.92	2.00157E-10	1.90016E-09
ENSG00000088854	C20orf194	0.98	2.01553E-10	1.91251E-09
ENSG00000198712	MT-CO2	-0.64	2.02108E-10	1.91686E-09
ENSG00000172239	PAIP1	0.57	2.02224E-10	1.91706E-09
ENSG00000127922	SEM1	0.50	2.05298E-10	1.94528E-09
ENSG00000104442	ARMC1	-0.81	2.0658E-10	1.9565E-09
ENSG00000065548	ZC3H15	0.52	2.08528E-10	1.97402E-09
ENSG00000111364	DDX55	-0.71	2.12536E-10	2.011E-09

ENSG00000197081	IGF2R	0.47	2.13307E-10	2.01734E-09
ENSG00000235194	PPP1R3E	-1.61	2.15796E-10	2.03992E-09
ENSG00000146433	TMEM181	-0.65	2.16017E-10	2.04105E-09
ENSG00000174564	IL20RB	-1.36	2.17266E-10	2.05187E-09
ENSG00000070366	SMG6	-1.01	2.1815E-10	2.05926E-09
ENSG00000138767	CNOT6L	-0.88	2.18514E-10	2.06172E-09
ENSG00000109686	SH3D19	-0.68	2.20412E-10	2.07864E-09
ENSG00000241553	ARPC4	0.50	2.25481E-10	2.12545E-09
ENSG00000100325	ASCC2	-0.58	2.26863E-10	2.13747E-09
ENSG00000119392	GLE1	-0.58	2.28238E-10	2.14941E-09
ENSG00000147454	SLC25A37	-0.53	2.33164E-10	2.19477E-09
ENSG00000134086	VHL	-0.54	2.33309E-10	2.1951E-09
ENSG00000179833	SERTAD2	-0.52	2.3376E-10	2.19831E-09
ENSG00000008283	CYB561	0.51	2.34718E-10	2.20628E-09
ENSG00000172057	ORMDL3	0.65	2.35392E-10	2.21158E-09
ENSG00000182308	DCAF4L1	2.17	2.35783E-10	2.21373E-09
ENSG00000179151	EDC3	-0.64	2.35842E-10	2.21373E-09
ENSG00000277072	STAG3L2	1.21	2.36256E-10	2.21658E-09
ENSG00000142669	SH3BGRL3	0.56	2.43796E-10	2.28624E-09
ENSG00000061273	HDAC7	-0.79	2.449E-10	2.29553E-09
ENSG00000223501	VPS52	-0.64	2.45546E-10	2.3005E-09
ENSG00000243943	ZNF512	-0.70	2.47588E-10	2.31854E-09
ENSG00000198554	WDHD1	-0.78	2.51373E-10	2.35289E-09
ENSG00000149428	HYOU1	-0.37	2.55931E-10	2.39444E-09
ENSG00000167964	RAB26	-0.89	2.60343E-10	2.43457E-09
ENSG00000105939	ZC3HAV1	0.59	2.6112E-10	2.4407E-09
ENSG00000168140	VASN	1.32	2.66353E-10	2.48845E-09
ENSG00000167978	SRRM2	-0.43	2.66606E-10	2.48965E-09
ENSG00000179241	LDLRAD3	-0.77	2.66768E-10	2.49E-09
ENSG00000100599	RIN3	-0.99	2.67344E-10	2.49422E-09
ENSG00000184524	CEND1	3.73	2.67842E-10	2.49769E-09
ENSG00000099864	PALM	-0.80	2.68428E-10	2.50199E-09
ENSG00000115652	UXS1	-0.68	2.73344E-10	2.54663E-09
ENSG00000197256	KANK2	-0.61	2.73485E-10	2.54676E-09
ENSG00000172965	MIR4435-2HG	0.63	2.77167E-10	2.57984E-09
ENSG00000219665	ZNF433-AS1	1.78	2.87121E-10	2.67126E-09
ENSG00000204103	MAFB	3.18	2.8751E-10	2.67363E-09
ENSG00000102100	SLC35A2	-0.82	2.91642E-10	2.7108E-09
ENSG00000183495	EP400	-0.53	2.93163E-10	2.72367E-09
ENSG00000197021	EOLA2	0.94	2.93912E-10	2.72936E-09
ENSG00000160917	CPSF4	-0.65	2.94777E-10	2.73612E-09
ENSG00000144746	ARL6IP5	0.67	2.98599E-10	2.77031E-09
ENSG00000185453	ZSWIM9	-1.02	2.99007E-10	2.77282E-09
ENSG00000117122	MFAP2	0.65	3.02808E-10	2.80676E-09
ENSG00000085365	SCAMP1	-0.80	3.04565E-10	2.82174E-09
ENSG00000113407	TARS1	0.39	3.06965E-10	2.84247E-09

ENSG00000107897	ACBD5	0.76	3.07087E-10	2.84247E-09
ENSG00000103194	USP10	-0.45	3.10601E-10	2.87367E-09
ENSG00000160688	FLAD1	-0.63	3.14083E-10	2.90454E-09
ENSG00000087266	SH3BP2	-0.63	3.14555E-10	2.90756E-09
ENSG00000164885	CDK5	0.90	3.15639E-10	2.91502E-09
ENSG00000134748	PRPF38A	0.59	3.15653E-10	2.91502E-09
ENSG00000129932	DOHH	0.77	3.18076E-10	2.93604E-09
ENSG00000090447	TFAP4	-0.89	3.19997E-10	2.95241E-09
ENSG00000258311	AC009779.3	-3.25	3.22879E-10	2.97763E-09
ENSG00000136848	DAB2IP	-0.81	3.26324E-10	3.00801E-09
ENSG00000111237	VPS29	0.59	3.29287E-10	3.03393E-09
ENSG00000116209	TMEM59	0.54	3.34126E-10	3.0771E-09
ENSG00000155760	FZD7	0.75	3.34386E-10	3.07807E-09
ENSG00000141738	GRB7	-0.96	3.43112E-10	3.15695E-09
ENSG00000204590	GNL1	0.44	3.49109E-10	3.21065E-09
ENSG00000112773	TENT5A	0.83	3.55397E-10	3.26697E-09
ENSG00000257621	PSMA3-AS1	-0.90	3.56192E-10	3.27278E-09
ENSG00000144357	UBR3	0.75	3.60758E-10	3.31322E-09
ENSG00000103196	CRISPLD2	1.33	3.67963E-10	3.37783E-09
ENSG00000119401	TRIM32	0.69	3.69946E-10	3.39448E-09
ENSG00000083807	SLC27A5	-1.52	3.75789E-10	3.44652E-09
ENSG00000169925	BRD3	-0.81	3.76284E-10	3.44947E-09
ENSG00000112514	CUTA	0.53	3.84411E-10	3.52236E-09
ENSG00000148225	WDR31	-1.72	3.91146E-10	3.58243E-09
ENSG00000136819	C9orf78	0.54	3.98517E-10	3.64827E-09
ENSG00000165948	IFI27L1	0.91	3.99903E-10	3.65929E-09
ENSG00000231365	WARS2-AS1	-1.33	4.02034E-10	3.6771E-09
ENSG00000156256	USP16	0.65	4.03519E-10	3.689E-09
ENSG00000185201	IFITM2	0.67	4.04853E-10	3.6995E-09
ENSG00000099910	KLHL22	-1.24	4.05446E-10	3.70323E-09
ENSG00000145214	DGKQ	-0.75	4.11774E-10	3.75932E-09
ENSG00000164172	MOCS2	0.70	4.14338E-10	3.781E-09
ENSG00000186298	PPP1CC	0.39	4.14983E-10	3.78365E-09
ENSG00000256268	LINC02454	1.95	4.15007E-10	3.78365E-09
ENSG00000099901	RANBP1	-0.40	4.15245E-10	3.7841E-09
ENSG00000011052	NME1-NME2	1.03	4.18417E-10	3.81127E-09
ENSG00000087510	TFAP2C	1.17	4.20452E-10	3.82807E-09
ENSG00000116747	RO60	0.55	4.24897E-10	3.86677E-09
ENSG00000104671	DCTN6	0.69	4.31512E-10	3.92519E-09
ENSG00000084731	KIF3C	1.01	4.37206E-10	3.97518E-09
ENSG00000247134	AC090204.1	1.14	4.43081E-10	4.02676E-09
ENSG00000100526	CDKN3	0.70	4.51501E-10	4.10143E-09
ENSG00000164576	SAP30L	-0.92	4.52257E-10	4.10632E-09
ENSG00000133789	SWAP70	0.64	4.5245E-10	4.10632E-09
ENSG00000130827	PLXNA3	-0.72	4.54659E-10	4.1245E-09
ENSG00000105699	LSR	0.46	4.60285E-10	4.17364E-09

ENSG00000135069	PSAT1	-0.43	4.62271E-10	4.18975E-09
ENSG00000111727	HCFC2	1.39	4.6441E-10	4.20578E-09
ENSG00000138750	NUP54	0.66	4.6446E-10	4.20578E-09
ENSG00000123064	DDX54	-0.54	4.68143E-10	4.23721E-09
ENSG00000167447	SMG8	0.63	4.70166E-10	4.2536E-09
ENSG00000020181	ADGRA2	-1.34	4.7048E-10	4.25452E-09
ENSG00000110063	DCPS	-0.75	4.71061E-10	4.25784E-09
ENSG00000071994	PDCD2	-0.50	4.78488E-10	4.32302E-09
ENSG00000164096	C4orf3	0.63	4.83791E-10	4.36896E-09
ENSG00000104998	IL27RA	0.70	4.89228E-10	4.41607E-09
ENSG00000086666	ZFAND6	0.59	4.91682E-10	4.43539E-09
ENSG00000125864	BFSP1	1.14	4.91812E-10	4.43539E-09
ENSG00000157600	TMEM164	-0.81	5.04093E-10	4.5441E-09
ENSG00000221823	PPP3R1	0.50	5.05258E-10	4.55255E-09
ENSG00000171163	ZNF692	-0.78	5.17147E-10	4.65758E-09
ENSG00000265681	RPL17	0.59	5.19059E-10	4.6727E-09
ENSG00000140829	DHX38	0.47	5.36926E-10	4.83137E-09
ENSG00000100941	PNN	-0.47	5.41506E-10	4.87039E-09
ENSG00000157510	AFAP1L1	1.17	5.4684E-10	4.91616E-09
ENSG00000172943	PHF8	-0.83	5.5202E-10	4.9605E-09
ENSG00000076554	TPD52	-0.52	5.55024E-10	4.98525E-09
ENSG00000241258	CRCP	0.69	5.57026E-10	4.99979E-09
ENSG00000160695	VPS11	-0.88	5.57142E-10	4.99979E-09
ENSG00000164758	MED30	-0.82	5.66841E-10	5.08455E-09
ENSG00000159840	ZYX	-0.54	5.77472E-10	5.17759E-09
ENSG00000168077	SCARA3	-0.70	5.78312E-10	5.18279E-09
ENSG00000163814	CDCP1	0.38	5.88435E-10	5.27115E-09
ENSG00000118816	CCNI	-0.39	5.90084E-10	5.28357E-09
ENSG00000260565	ERVK13-1	1.26	5.97346E-10	5.3462E-09
ENSG00000006831	ADIPOR2	0.40	6.10963E-10	5.46562E-09
ENSG00000107789	MINPP1	0.78	6.12334E-10	5.47544E-09
ENSG00000100226	GTPBP1	-0.67	6.15691E-10	5.503E-09
ENSG00000104164	BLOC1S6	-0.65	6.1864E-10	5.52689E-09
ENSG00000137449	CPEB2	1.39	6.18955E-10	5.52723E-09
ENSG00000054654	SYNE2	-0.86	6.22708E-10	5.55827E-09
ENSG00000115364	MRPL19	0.58	6.2389E-10	5.56633E-09
ENSG00000101298	SNPH	1.02	6.28483E-10	5.60358E-09
ENSG00000166311	SMPD1	1.15	6.28625E-10	5.60358E-09
ENSG00000130340	SNX9	0.50	6.30012E-10	5.61345E-09
ENSG00000155115	GTF3C6	0.83	6.30773E-10	5.61772E-09
ENSG00000100324	TAB1	-0.90	6.32642E-10	5.63186E-09
ENSG00000115884	SDC1	0.47	6.39466E-10	5.69008E-09
ENSG00000124508	BTN2A2	0.91	6.39878E-10	5.69121E-09
ENSG00000184445	KNTC1	-0.69	6.40318E-10	5.6926E-09
ENSG00000103319	EEF2K	-0.68	6.59305E-10	5.8588E-09
ENSG00000054118	THRAP3	-0.39	6.61794E-10	5.8783E-09

ENSG00000140987	ZSCAN32	-1.00	6.69178E-10	5.94125E-09
ENSG00000143569	UBAP2L	0.41	6.73174E-10	5.97408E-09
ENSG00000147457	CHMP7	0.52	6.73892E-10	5.9778E-09
ENSG00000122786	CALD1	-0.57	6.77587E-10	6.00792E-09
ENSG00000198805	PNP	0.50	6.78459E-10	6.01299E-09
ENSG00000133997	MED6	0.60	6.84705E-10	6.06565E-09
ENSG00000198399	ITSN2	0.71	6.88932E-10	6.10041E-09
ENSG00000165630	PRPF18	1.15	7.03598E-10	6.22751E-09
ENSG00000118898	PPL	-0.68	7.06808E-10	6.25316E-09
ENSG00000140740	UQCRC2	-0.44	7.12195E-10	6.29803E-09
ENSG00000103342	GSPT1	0.42	7.2319E-10	6.39244E-09
ENSG00000169564	PCBP1	0.42	7.42583E-10	6.56096E-09
ENSG00000187944	C2orf66	4.13	7.53573E-10	6.65512E-09
ENSG00000139579	NABP2	-0.56	7.61257E-10	6.72002E-09
ENSG00000115307	AUP1	0.45	7.6457E-10	6.74629E-09
ENSG00000133477	FAM83F	-0.74	7.6797E-10	6.7733E-09
ENSG00000115694	STK25	-0.54	7.68682E-10	6.7766E-09
ENSG00000103044	HAS3	-0.70	7.70902E-10	6.79317E-09
ENSG00000169967	MAP3K2	0.69	7.73414E-10	6.8123E-09
ENSG00000079332	SAR1A	0.45	7.7536E-10	6.82645E-09
ENSG00000116254	CHD5	0.88	7.88048E-10	6.9351E-09
ENSG00000178028	DMAP1	0.66	7.89146E-10	6.94171E-09
ENSG00000175344	CHRNA7	1.41	7.96661E-10	7.00474E-09
ENSG00000152242	C18orf25	-0.66	8.04315E-10	7.06893E-09
ENSG00000108559	NUP88	0.55	8.12492E-10	7.13766E-09
ENSG00000137486	ARRB1	0.75	8.24547E-10	7.24038E-09
ENSG00000184986	TMEM121	1.40	8.36744E-10	7.34426E-09
ENSG00000133114	GPALPP1	-1.05	8.47743E-10	7.43754E-09
ENSG00000088205	DDX18	0.51	8.49483E-10	7.44954E-09
ENSG00000102316	MAGED2	0.59	8.58349E-10	7.524E-09
ENSG00000136146	MED4	0.71	8.78408E-10	7.69645E-09
ENSG00000083123	BCKDHB	-1.52	8.86022E-10	7.75977E-09
ENSG00000158019	BABAM2	-0.86	8.87667E-10	7.77078E-09
ENSG00000085433	WDR47	1.11	8.88203E-10	7.77207E-09
ENSG00000135900	MRPL44	0.53	8.93723E-10	7.81695E-09
ENSG00000069345	DNAJA2	0.52	9.03307E-10	7.89732E-09
ENSG00000173207	CKS1B	0.45	9.07458E-10	7.93016E-09
ENSG00000117139	KDM5B	0.72	9.11507E-10	7.96206E-09
ENSG00000184900	SUMO3	0.61	9.1319E-10	7.97328E-09
ENSG00000106211	HSPB1	0.97	9.16277E-10	7.99675E-09
ENSG00000116679	IVNS1ABP	-0.54	9.1987E-10	8.0246E-09
ENSG00000168591	TMUB2	0.72	9.32829E-10	8.13072E-09
ENSG00000101126	ADNP	0.47	9.32847E-10	8.13072E-09
ENSG00000166971	AKTIP	0.83	9.37931E-10	8.17147E-09
ENSG00000148908	RGS10	-0.62	9.47773E-10	8.25363E-09
ENSG00000204231	RXRB	-0.64	9.49403E-10	8.26242E-09

ENSG00000169126	ARMC4	-1.44	9.49608E-10	8.26242E-09
ENSG00000164068	RNF123	-0.70	9.57969E-10	8.33154E-09
ENSG00000167105	TMEM92	1.23	1.00619E-09	8.7471E-09
ENSG00000061936	SFSWAP	0.55	1.00838E-09	8.76232E-09
ENSG00000275052	PPP4R3B	0.58	1.00889E-09	8.76297E-09
ENSG00000163743	RCHY1	0.75	1.02446E-09	8.89439E-09
ENSG00000175054	ATR	-0.88	1.02725E-09	8.91472E-09
ENSG00000174606	ANGEL2	-0.84	1.02831E-09	8.92006E-09
ENSG00000224877	NDUFAF8	0.58	1.04278E-09	9.04167E-09
ENSG00000197498	RPF2	-0.67	1.05224E-09	9.11968E-09
ENSG00000129028	THAP10	1.73	1.06595E-09	9.23458E-09
ENSG00000118873	RAB3GAP2	0.72	1.07531E-09	9.3116E-09
ENSG00000104899	AMH	-1.11	1.07724E-09	9.32424E-09
ENSG00000164924	YWHAZ	0.39	1.08137E-09	9.35598E-09
ENSG00000131116	ZNF428	0.62	1.09184E-09	9.44245E-09
ENSG00000108349	CASC3	0.42	1.09409E-09	9.45781E-09
ENSG00000010017	RANBP9	0.54	1.10239E-09	9.5255E-09
ENSG00000267575	AC006504.5	1.33	1.11163E-09	9.60119E-09
ENSG00000002330	BAD	0.74	1.13103E-09	9.76449E-09
ENSG00000261490	AC005674.2	-2.33	1.14262E-09	9.86029E-09
ENSG00000198730	CTR9	0.54	1.14765E-09	9.89948E-09
ENSG00000131979	GCH1	0.65	1.15151E-09	9.92847E-09
ENSG00000100387	RBX1	0.50	1.16469E-09	1.00378E-08
ENSG00000280670	CCDC163	1.42	1.18409E-09	1.02E-08
ENSG00000134815	DHX34	-0.68	1.18452E-09	1.02E-08
ENSG00000179335	CLK3	0.56	1.19028E-09	1.02451E-08
ENSG00000114120	SLC25A36	-0.58	1.19306E-09	1.02646E-08
ENSG00000189043	NDUFA4	0.42	1.21521E-09	1.04507E-08
ENSG00000185404	SP140L	0.85	1.2225E-09	1.05088E-08
ENSG00000108175	ZMIZ1	-0.64	1.22623E-09	1.05365E-08
ENSG00000158186	MRAS	-0.96	1.23846E-09	1.06369E-08
ENSG00000064309	CDON	0.98	1.24118E-09	1.06557E-08
ENSG00000205730	ITPRIPL2	-1.20	1.24335E-09	1.06698E-08
ENSG00000275395	FCGBP	-2.29	1.26113E-09	1.08177E-08
ENSG00000162300	ZFPL1	-1.10	1.27651E-09	1.0945E-08
ENSG00000188566	NDOR1	-0.90	1.28191E-09	1.09866E-08
ENSG00000075223	SEMA3C	0.65	1.29558E-09	1.1099E-08
ENSG00000110851	PRDM4	-0.78	1.31983E-09	1.13018E-08
ENSG00000198496	NBR2	-1.40	1.3322E-09	1.14029E-08
ENSG00000177646	ACAD9	-0.58	1.33304E-09	1.14052E-08
ENSG00000009307	CSDE1	0.38	1.35247E-09	1.15665E-08
ENSG00000089902	RCOR1	0.53	1.3673E-09	1.16852E-08
ENSG00000198755	RPL10A	-0.40	1.36752E-09	1.16852E-08
ENSG00000213585	VDAC1	-0.40	1.38356E-09	1.18172E-08
ENSG00000152127	MGAT5	-0.59	1.39109E-09	1.18765E-08
ENSG00000110075	PPP6R3	-0.52	1.42584E-09	1.2168E-08

ENSG00000198223	CSF2RA	1.53	1.47918E-09	1.26178E-08
ENSG00000187244	BCAM	-0.70	1.49112E-09	1.27143E-08
ENSG00000106605	BLVRA	0.65	1.49482E-09	1.27404E-08
ENSG00000079616	KIF22	0.42	1.5414E-09	1.31317E-08
ENSG00000227671	AL390728.4	0.86	1.59773E-09	1.36011E-08
ENSG00000162069	BICDL2	-1.39	1.59785E-09	1.36011E-08
ENSG00000115756	HPCAL1	0.51	1.61397E-09	1.37325E-08
ENSG00000131153	GINS2	-0.46	1.62264E-09	1.38004E-08
ENSG00000173744	AGFG1	0.53	1.67992E-09	1.42814E-08
ENSG00000122884	P4HA1	0.68	1.6843E-09	1.43126E-08
ENSG00000134318	ROCK2	-0.64	1.69123E-09	1.43654E-08
ENSG00000197181	PIWIL2	2.98	1.69813E-09	1.44179E-08
ENSG00000116678	LEPR	1.05	1.7299E-09	1.46814E-08
ENSG00000107021	TBC1D13	-1.01	1.79044E-09	1.51887E-08
ENSG00000080986	NDC80	0.64	1.82435E-09	1.54699E-08
ENSG00000141198	TOM1L1	0.63	1.82734E-09	1.54887E-08
ENSG00000137171	KLC4	-1.33	1.84749E-09	1.56529E-08
ENSG00000134690	CDCA8	-0.54	1.86188E-09	1.5768E-08
ENSG00000085741	WNT11	2.58	1.86488E-09	1.57868E-08
ENSG00000186998	EMID1	-1.52	1.88047E-09	1.5912E-08
ENSG00000170921	TANC2	0.57	1.8841E-09	1.5936E-08
ENSG00000096696	DSP	-0.42	1.90565E-09	1.61114E-08
ENSG00000139131	YARS2	0.76	1.92853E-09	1.62981E-08
ENSG00000103415	HMOX2	-0.68	1.93864E-09	1.63765E-08
ENSG00000100116	GCAT	-0.56	1.93975E-09	1.6379E-08
ENSG00000126226	PCID2	-0.55	1.96162E-09	1.65567E-08
ENSG00000205643	CDPF1	-1.14	1.97778E-09	1.66861E-08
ENSG00000110315	RNF141	-0.46	2.00263E-09	1.68886E-08
ENSG00000136986	DERL1	0.47	2.02833E-09	1.70981E-08
ENSG00000176834	VSIG10	-0.62	2.03465E-09	1.71442E-08
ENSG00000104356	POP1	0.57	2.05207E-09	1.72837E-08
ENSG00000197961	ZNF121	-0.51	2.05809E-09	1.73271E-08
ENSG00000100023	PPIL2	-0.63	2.06328E-09	1.73634E-08
ENSG00000173334	TRIB1	-0.39	2.07406E-09	1.74468E-08
ENSG00000105963	ADAP1	-1.15	2.08183E-09	1.75049E-08
ENSG00000175274	TP53I11	-0.67	2.08349E-09	1.75114E-08
ENSG00000072864	NDE1	0.57	2.14689E-09	1.80368E-08
ENSG00000167004	PDIA3	0.35	2.19687E-09	1.84489E-08
ENSG00000185880	TRIM69	0.84	2.24065E-09	1.88087E-08
ENSG00000138834	MAPK8IP3	-0.63	2.2499E-09	1.88722E-08
ENSG00000100321	SYNGR1	0.67	2.25011E-09	1.88722E-08
ENSG00000204498	NFKBIL1	0.74	2.25449E-09	1.89011E-08
ENSG00000100221	JOSD1	-0.47	2.27517E-09	1.90665E-08
ENSG00000154146	NRGN	0.81	2.28332E-09	1.91267E-08
ENSG00000163577	EIF5A2	0.85	2.28818E-09	1.91538E-08
ENSG00000151883	PARP8	0.85	2.28846E-09	1.91538E-08

ENSG00000136709	WDR33	-0.52	2.3203E-09	1.94121E-08
ENSG00000164808	SPIDR	-0.72	2.35858E-09	1.97242E-08
ENSG00000105255	FSD1	0.83	2.41072E-09	2.01517E-08
ENSG00000267156	TPMTP1	-3.49	2.41398E-09	2.01706E-08
ENSG00000151006	PRSS53	-1.66	2.41732E-09	2.01901E-08
ENSG00000132801	ZSWIM3	1.72	2.48277E-09	2.07281E-08
ENSG00000162490	DRAXIN	1.69	2.50157E-09	2.08763E-08
ENSG00000167548	KMT2D	-0.49	2.52867E-09	2.10936E-08
ENSG00000276180	H4C9	1.44	2.53145E-09	2.11081E-08
ENSG00000198055	GRK6	-0.50	2.53773E-09	2.11517E-08
ENSG00000164024	METAP1	-0.45	2.55232E-09	2.12644E-08
ENSG00000198380	GFPT1	0.44	2.56866E-09	2.13916E-08
ENSG00000106524	ANKMY2	0.71	2.66713E-09	2.22025E-08
ENSG00000099282	TSPAN15	-0.83	2.67574E-09	2.22648E-08
ENSG00000117410	ATP6V0B	-0.51	2.69224E-09	2.23928E-08
ENSG00000123131	PRDX4	0.47	2.69554E-09	2.24109E-08
ENSG00000140320	BAHD1	-0.64	2.71844E-09	2.2592E-08
ENSG00000118369	USP35	1.09	2.73222E-09	2.26971E-08
ENSG00000125148	MT2A	-0.81	2.74382E-09	2.2784E-08
ENSG00000161542	PRPSAP1	0.49	2.7644E-09	2.29453E-08
ENSG00000071894	CPSF1	-0.42	2.77351E-09	2.30114E-08
ENSG00000132842	AP3B1	0.52	2.78603E-09	2.31057E-08
ENSG00000125266	EFNB2	-0.68	2.80635E-09	2.32646E-08
ENSG00000090615	GOLGA3	0.49	2.83965E-09	2.35309E-08
ENSG00000006740	ARHGAP44	-1.32	2.85031E-09	2.36094E-08
ENSG00000110492	MDK	-0.44	2.89333E-09	2.39558E-08
ENSG00000172667	ZMAT3	-0.80	2.92267E-09	2.41888E-08
ENSG00000101335	MYL9	0.67	2.93284E-09	2.4263E-08
ENSG00000179820	MYADM	0.47	2.93761E-09	2.42923E-08
ENSG00000138180	CEP55	0.49	2.97183E-09	2.45652E-08
ENSG00000186523	FAM86B1	-2.44	2.99977E-09	2.47859E-08
ENSG00000020922	MRE11	-0.82	3.00542E-09	2.48223E-08
ENSG00000102897	LYRM1	-0.95	3.05263E-09	2.52018E-08
ENSG00000135776	ABCB10	-0.96	3.0546E-09	2.52077E-08
ENSG00000141380	SS18	0.52	3.07015E-09	2.53256E-08
ENSG00000279012	OR51B2	-4.26	3.0965E-09	2.55324E-08
ENSG00000247315	ZCCHC3	0.54	3.16657E-09	2.60994E-08
ENSG00000135047	CTSL	0.49	3.17992E-09	2.61986E-08
ENSG00000078319	PMS2P1	0.69	3.19961E-09	2.635E-08
ENSG00000011523	CEP68	-0.94	3.21038E-09	2.64279E-08
ENSG00000135604	STX11	1.77	3.26089E-09	2.68326E-08
ENSG00000099804	CDC34	0.52	3.26874E-09	2.68862E-08
ENSG00000161526	SAP30BP	0.46	3.32306E-09	2.73217E-08
ENSG00000128311	TST	-0.87	3.3771E-09	2.77546E-08
ENSG00000036257	CUL3	0.46	3.40428E-09	2.79665E-08
ENSG00000119681	LTBP2	1.33	3.42805E-09	2.81502E-08

ENSG00000121274	TENT4B	0.79	3.44377E-09	2.82677E-08
ENSG00000105656	ELL	0.72	3.50375E-09	2.87483E-08
ENSG00000198752	CDC42BPB	-0.43	3.50791E-09	2.87706E-08
ENSG00000142207	URB1	-0.51	3.51615E-09	2.88264E-08
ENSG00000182253	SYNM	-1.49	3.52361E-09	2.88757E-08
ENSG00000047578	KIAA0556	-0.83	3.53087E-09	2.89233E-08
ENSG00000136770	DNAJC1	0.76	3.53476E-09	2.89434E-08
ENSG00000223745	CCDC18-AS1	-1.52	3.57832E-09	2.92881E-08
ENSG00000111276	CDKN1B	0.61	3.58798E-09	2.93551E-08
ENSG00000142731	PLK4	0.64	3.60846E-09	2.95107E-08
ENSG00000168291	PDHB	0.50	3.62783E-09	2.96566E-08
ENSG00000127191	TRAF2	0.62	3.62928E-09	2.96566E-08
ENSG00000062524	LTK	1.05	3.6414E-09	2.97436E-08
ENSG00000105048	TNNT1	0.44	3.6503E-09	2.98041E-08
ENSG00000120616	EPC1	0.88	3.67499E-09	2.99934E-08
ENSG00000139505	MTMR6	0.77	3.72396E-09	3.03807E-08
ENSG00000138018	SELENOI	-0.47	3.7279E-09	3.0397E-08
ENSG00000104904	OAZ1	0.45	3.729E-09	3.0397E-08
ENSG00000130734	ATG4D	-0.77	3.78803E-09	3.08657E-08
ENSG00000134352	IL6ST	0.70	3.82353E-09	3.11422E-08
ENSG00000177156	TALDO1	-0.45	3.84763E-09	3.13258E-08
ENSG00000175166	PSMD2	0.36	3.87153E-09	3.15063E-08
ENSG00000109046	WSB1	0.63	3.87295E-09	3.15063E-08
ENSG00000151502	VPS26B	-0.64	3.91882E-09	3.18665E-08
ENSG00000184465	WDR27	-1.04	3.94984E-09	3.21057E-08
ENSG00000170571	EMB	-1.18	4.14357E-09	3.36667E-08
ENSG00000136868	SLC31A1	0.56	4.1886E-09	3.40188E-08
ENSG00000148120	AOPEP	1.19	4.21142E-09	3.41902E-08
ENSG00000105254	TBCB	0.50	4.31263E-09	3.49977E-08
ENSG00000177542	SLC25A22	-0.61	4.32058E-09	3.5048E-08
ENSG00000108666	C17orf75	-0.98	4.33709E-09	3.51677E-08
ENSG00000178149	DALRD3	-0.73	4.34481E-09	3.5216E-08
ENSG00000120314	WDR55	-0.65	4.34968E-09	3.52412E-08
ENSG00000269044	AC024075.2	-1.93	4.43779E-09	3.59405E-08
ENSG00000181222	POLR2A	0.38	4.53842E-09	3.67406E-08
ENSG00000136811	ODF2	-0.51	4.55043E-09	3.6823E-08
ENSG00000242193	CRYZL2P	-2.04	4.71004E-09	3.80992E-08
ENSG00000137103	TMEM8B	-1.69	4.73455E-09	3.82819E-08
ENSG00000167191	GPRC5B	0.84	4.74748E-09	3.8371E-08
ENSG00000215012	RTL10	-0.79	4.86904E-09	3.93376E-08
ENSG00000143590	EFNA3	0.86	4.88458E-09	3.94335E-08
ENSG00000177943	MAMDC4	-1.75	4.88485E-09	3.94335E-08
ENSG00000104325	DECR1	0.60	4.9174E-09	3.96803E-08
ENSG00000163659	TIPARP	-0.68	4.92758E-09	3.97463E-08
ENSG00000118965	WDR35	-1.09	4.97647E-09	4.01245E-08
ENSG00000156831	NSMCE2	0.90	4.98373E-09	4.01669E-08

ENSG00000171444	MCC	0.64	5.18767E-09	4.17938E-08
ENSG00000060642	PIGV	-0.97	5.2013E-09	4.18867E-08
ENSG00000064012	CASP8	0.67	5.30012E-09	4.26654E-08
ENSG00000119630	PGF	1.22	5.32518E-09	4.28451E-08
ENSG00000205744	DENND1C	-0.79	5.32673E-09	4.28451E-08
ENSG00000124523	SIRT5	-0.95	5.39209E-09	4.33374E-08
ENSG00000160908	ZNF394	0.89	5.39227E-09	4.33374E-08
ENSG00000096968	JAK2	1.22	5.46474E-09	4.39022E-08
ENSG00000137692	DCUN1D5	0.48	5.52176E-09	4.43424E-08
ENSG00000151150	ANK3	-1.83	5.53451E-09	4.44271E-08
ENSG00000127990	SGCE	0.85	5.57612E-09	4.47431E-08
ENSG00000169242	EFNA1	0.91	5.57943E-09	4.47517E-08
ENSG00000154767	XPC	-0.67	5.59512E-09	4.48543E-08
ENSG00000058056	USP13	-0.62	5.5967E-09	4.48543E-08
ENSG00000219529	AP000580.1	1.84	5.67091E-09	4.54309E-08
ENSG00000144034	TPRKB	0.81	5.67988E-09	4.54845E-08
ENSG00000137133	HINT2	-0.91	5.70683E-09	4.5682E-08
ENSG00000259959	AC107068.1	-2.78	5.7312E-09	4.58588E-08
ENSG00000240342	RPS2P5	-0.40	5.84554E-09	4.67231E-08
ENSG00000144815	NXPE3	0.86	5.84576E-09	4.67231E-08
ENSG00000062650	WAPL	0.53	5.84623E-09	4.67231E-08
ENSG00000102531	FNDC3A	0.63	5.91161E-09	4.72268E-08
ENSG00000104368	PLAT	0.94	6.00058E-09	4.79184E-08
ENSG00000198108	CHSY3	1.75	6.06643E-09	4.84249E-08
ENSG00000166925	TSC22D4	0.76	6.08391E-09	4.85451E-08
ENSG00000073712	FERMT2	0.65	6.21728E-09	4.95896E-08
ENSG00000049323	LTBP1	1.13	6.24893E-09	4.98222E-08
ENSG00000115159	GPD2	-0.51	6.34684E-09	5.05826E-08
ENSG00000176046	NUPR1	-2.23	6.43149E-09	5.12369E-08
ENSG00000163125	RPRD2	-0.66	6.53875E-09	5.20707E-08
ENSG00000138663	COPS4	0.69	6.59267E-09	5.24791E-08
ENSG00000134759	ELP2	-0.53	6.60764E-09	5.25774E-08
ENSG00000012211	PRICKLE3	-0.84	6.70453E-09	5.33222E-08
ENSG00000115548	KDM3A	0.74	6.70658E-09	5.33222E-08
ENSG00000214226	C17orf67	1.61	6.74304E-09	5.35795E-08
ENSG00000182759	MAFA	1.52	6.74429E-09	5.35795E-08
ENSG00000143545	RAB13	0.58	6.78687E-09	5.38963E-08
ENSG00000110619	CARS1	-0.38	6.89893E-09	5.47438E-08
ENSG00000151276	MAGI1	0.69	6.89906E-09	5.47438E-08
ENSG00000130511	SSBP4	-0.66	6.97091E-09	5.52921E-08
ENSG00000141101	NOB1	-0.46	7.05945E-09	5.59721E-08
ENSG00000170955	CAVIN3	0.78	7.11192E-09	5.63659E-08
ENSG00000138604	GLCE	-0.82	7.2642E-09	5.75499E-08
ENSG00000131584	ACAP3	-0.71	7.27211E-09	5.75898E-08
ENSG00000170677	SOCS6	0.54	7.35758E-09	5.82437E-08
ENSG00000226479	TMEM185B	-0.61	7.41244E-09	5.86547E-08

ENSG00000068745	IP6K2	0.50	7.41893E-09	5.86828E-08
ENSG00000153815	CMIP	-0.61	7.48555E-09	5.91864E-08
ENSG00000122126	OCRL	-0.69	7.5058E-09	5.93231E-08
ENSG00000105514	RAB3D	0.67	7.5414E-09	5.95809E-08
ENSG00000122386	ZNF205	0.82	7.71924E-09	6.09619E-08
ENSG00000138131	LOXL4	-1.74	7.73229E-09	6.10409E-08
ENSG00000124181	PLCG1	0.55	7.73952E-09	6.10739E-08
ENSG00000151247	EIF4E	0.49	7.84492E-09	6.18812E-08
ENSG00000213839	TMX2P1	-1.49	7.93791E-09	6.259E-08
ENSG00000111875	ASF1A	0.74	7.98007E-09	6.28859E-08
ENSG00000040275	SPDL1	-0.80	7.98172E-09	6.28859E-08
ENSG00000116984	MTR	-0.67	8.04334E-09	6.33465E-08
ENSG00000165006	UBAP1	0.52	8.09114E-09	6.36978E-08
ENSG00000105135	ILVBL	-0.57	8.11617E-09	6.38697E-08
ENSG00000175920	DOK7	2.06	8.12254E-09	6.38947E-08
ENSG00000119720	NRDE2	0.82	8.26151E-09	6.49624E-08
ENSG00000153234	NR4A2	0.90	8.31379E-09	6.53478E-08
ENSG00000197070	ARRDC1	-0.66	8.32736E-09	6.54288E-08
ENSG00000253729	PRKDC	-0.39	8.36854E-09	6.57265E-08
ENSG00000175634	RPS6KB2	-0.62	8.43973E-09	6.62597E-08
ENSG00000111252	SH2B3	0.48	8.60347E-09	6.75187E-08
ENSG00000070756	PABPC1	-0.36	8.75967E-09	6.87176E-08
ENSG00000004059	ARF5	0.43	8.94112E-09	7.01135E-08
ENSG00000110074	FOXRED1	-0.63	9.06116E-09	7.1027E-08
ENSG00000144381	HSPD1	0.36	9.14143E-09	7.16281E-08
ENSG00000120334	CENPL	0.92	9.28563E-09	7.27295E-08
ENSG00000092841	MYL6	0.39	9.35154E-09	7.32172E-08
ENSG00000135956	TMEM127	-0.56	9.52602E-09	7.4554E-08
ENSG00000174010	KLHL15	0.79	9.61385E-09	7.5212E-08
ENSG00000049130	KITLG	-0.82	9.61839E-09	7.52181E-08
ENSG00000108443	RPS6KB1	0.50	9.74938E-09	7.62127E-08
ENSG00000143393	PI4KB	0.50	9.94742E-09	7.77305E-08
ENSG00000143303	RRNAD1	-0.89	1.00346E-08	7.83623E-08
ENSG00000227440	ATP5MC1P4	3.99	1.00361E-08	7.83623E-08
ENSG00000162542	TMCO4	-1.02	1.00428E-08	7.83842E-08
ENSG00000108370	RGS9	0.97	1.01069E-08	7.88538E-08
ENSG00000116353	MECR	-0.72	1.01284E-08	7.89902E-08
ENSG00000111775	COX6A1	-0.40	1.01925E-08	7.94596E-08
ENSG00000172059	KLF11	1.06	1.0369E-08	8.08041E-08
ENSG00000116649	SRM	-1.38	1.0392E-08	8.09517E-08
ENSG00000104812	GYS1	-0.62	1.04098E-08	8.10583E-08
ENSG00000127947	PTPN12	0.45	1.05324E-08	8.19814E-08
ENSG00000164023	SGMS2	-0.80	1.05785E-08	8.23083E-08
ENSG00000164442	CITED2	0.65	1.0777E-08	8.38204E-08
ENSG00000130227	XPO7	-0.54	1.09711E-08	8.52966E-08
ENSG00000005486	RHBDD2	0.56	1.10578E-08	8.59375E-08

ENSG00000110958	PTGES3	0.37	1.11248E-08	8.64246E-08
ENSG00000168887	C2orf68	-0.78	1.11775E-08	8.68005E-08
ENSG00000181788	SIAH2	-0.55	1.11858E-08	8.68307E-08
ENSG00000182310	SPACA6	1.48	1.12035E-08	8.69344E-08
ENSG00000068120	COASY	-0.46	1.12514E-08	8.72728E-08
ENSG00000132334	PTPRE	0.98	1.14095E-08	8.84647E-08
ENSG00000213024	NUP62	0.39	1.15182E-08	8.92724E-08
ENSG00000182952	HMGN4	-0.49	1.16167E-08	9.00014E-08
ENSG00000213699	SLC35F6	-0.58	1.16337E-08	9.0098E-08
ENSG00000156802	ATAD2	-0.44	1.16893E-08	9.04934E-08
ENSG00000147889	CDKN2A	0.50	1.17037E-08	9.05701E-08
ENSG00000161203	AP2M1	-0.37	1.19152E-08	9.21714E-08
ENSG00000204899	MZT1	0.56	1.19312E-08	9.22591E-08
ENSG00000169683	LRRC45	-0.71	1.20296E-08	9.29844E-08
ENSG00000074842	MYDGF	0.81	1.22414E-08	9.45851E-08
ENSG00000145476	CYP4V2	-1.20	1.23345E-08	9.52676E-08
ENSG00000127663	KDM4B	-0.66	1.23525E-08	9.53694E-08
ENSG00000205542	TMSB4X	0.38	1.24537E-08	9.61139E-08
ENSG00000278053	DDX52	0.54	1.2769E-08	9.8509E-08
ENSG00000180667	YOD1	0.60	1.2789E-08	9.86253E-08
ENSG00000113716	HMGXB3	-0.49	1.29245E-08	9.9632E-08
ENSG00000151923	TIAL1	0.39	1.30523E-08	1.00579E-07
ENSG00000108828	VAT1	0.40	1.32002E-08	1.01679E-07
ENSG00000140743	CDR2	0.65	1.34241E-08	1.03364E-07
ENSG00000136205	TNS3	-1.01	1.35949E-08	1.04639E-07
ENSG00000127946	HIP1	-0.63	1.36476E-08	1.05004E-07
ENSG00000163320	CGGBP1	-0.54	1.37018E-08	1.0538E-07
ENSG00000070614	NDST1	-0.57	1.3722E-08	1.05495E-07
ENSG00000102385	DRP2	1.83	1.38746E-08	1.06628E-07
ENSG00000135111	TBX3	1.11	1.39319E-08	1.07027E-07
ENSG00000187097	ENTPD5	-0.79	1.39702E-08	1.07202E-07
ENSG00000187730	GABRD	-0.98	1.39706E-08	1.07202E-07
ENSG00000010318	PHF7	1.29	1.39707E-08	1.07202E-07
ENSG00000166479	TMX3	-0.68	1.40174E-08	1.07519E-07
ENSG00000071859	FAM50A	0.53	1.40258E-08	1.07542E-07
ENSG00000070367	EXOC5	0.56	1.41948E-08	1.08796E-07
ENSG00000138942	RNF185	-0.69	1.44248E-08	1.10516E-07
ENSG00000131759	RARA	-0.89	1.47503E-08	1.12967E-07
ENSG00000165507	DEPP1	1.74	1.48243E-08	1.13491E-07
ENSG00000132356	PRKAA1	-0.68	1.48708E-08	1.13803E-07
ENSG00000273344	PAXIP1-AS1	-1.34	1.50076E-08	1.14806E-07
ENSG00000122877	EGR2	2.41	1.51209E-08	1.15629E-07
ENSG00000270069	MIR222HG	0.87	1.55244E-08	1.18668E-07
ENSG00000132912	DCTN4	0.45	1.56366E-08	1.19481E-07
ENSG00000129055	ANAPC13	0.61	1.57904E-08	1.20609E-07
ENSG00000175866	BAIAP2	0.48	1.60594E-08	1.22617E-07

ENSG00000100207	TCF20	-0.47	1.6069E-08	1.22644E-07
ENSG00000196428	TSC22D2	0.73	1.63262E-08	1.24559E-07
ENSG00000169660	HEXD	-0.94	1.63492E-08	1.24688E-07
ENSG00000013619	MAMLD1	-1.20	1.65107E-08	1.25871E-07
ENSG00000038358	EDC4	0.48	1.66505E-08	1.26889E-07
ENSG00000171316	CHD7	-0.62	1.67389E-08	1.27502E-07
ENSG00000119487	MAPKAP1	0.53	1.67443E-08	1.27502E-07
ENSG00000106330	MOSPD3	-1.04	1.67501E-08	1.27502E-07
ENSG00000108518	PFN1	0.46	1.68388E-08	1.28128E-07
ENSG00000119541	VPS4B	0.53	1.69644E-08	1.29035E-07
ENSG00000198331	HYLS1	0.77	1.70457E-08	1.29605E-07
ENSG00000106080	FKBP14	0.86	1.72292E-08	1.3095E-07
ENSG00000105321	CCDC9	0.53	1.74095E-08	1.3227E-07
ENSG00000136933	RABEPK	-0.52	1.74509E-08	1.32501E-07
ENSG00000003137	CYP26B1	0.61	1.74532E-08	1.32501E-07
ENSG00000236088	COX10-AS1	-1.21	1.75094E-08	1.32878E-07
ENSG00000287707	AC115284.4	-2.37	1.75416E-08	1.33072E-07
ENSG00000105327	BBC3	-0.69	1.78182E-08	1.35118E-07
ENSG00000253352	TUG1	-0.49	1.79994E-08	1.36441E-07
ENSG00000166889	PATL1	0.43	1.80707E-08	1.3693E-07
ENSG00000101577	LPIN2	-0.88	1.81162E-08	1.37222E-07
ENSG00000092148	HECTD1	0.41	1.82834E-08	1.38436E-07
ENSG00000155016	CYP2U1	-1.43	1.83213E-08	1.38671E-07
ENSG00000126821	SGPP1	-0.72	1.84464E-08	1.39565E-07
ENSG00000135720	DYNC1LI2	0.49	1.84779E-08	1.3975E-07
ENSG00000131697	NPHP4	-0.97	1.85095E-08	1.39937E-07
ENSG00000162623	TYW3	0.68	1.85191E-08	1.39957E-07
ENSG00000266910	AC008507.1	-3.25	1.8564E-08	1.40242E-07
ENSG00000065978	YBX1	0.33	1.87675E-08	1.41727E-07
ENSG00000068323	TFE3	0.59	1.87966E-08	1.41893E-07
ENSG00000165698	SPACA9	-2.06	1.88157E-08	1.41983E-07
ENSG00000153187	HNRNPU	-0.31	1.89588E-08	1.43009E-07
ENSG00000151694	ADAM17	0.56	1.89761E-08	1.43085E-07
ENSG00000115128	SF3B6	0.49	1.89934E-08	1.43162E-07
ENSG00000198815	FOXJ3	0.44	1.91111E-08	1.43995E-07
ENSG00000185650	ZFP36L1	-0.42	1.9698E-08	1.48361E-07
ENSG00000115084	SLC35F5	-0.88	1.97613E-08	1.48782E-07
ENSG00000169032	MAP2K1	0.45	1.99574E-08	1.50202E-07
ENSG00000082898	XPO1	0.38	2.00128E-08	1.50562E-07
ENSG00000125846	ZNF133	-1.49	2.03245E-08	1.5285E-07
ENSG00000142230	SAE1	0.34	2.05034E-08	1.54137E-07
ENSG00000014914	MTMR11	0.77	2.05767E-08	1.54631E-07
ENSG00000215883	CYB5RL	-0.90	2.05893E-08	1.54667E-07
ENSG00000147065	MSN	-0.33	2.15506E-08	1.61818E-07
ENSG00000117505	DR1	-0.50	2.15573E-08	1.61818E-07
ENSG00000170365	SMAD1	-0.79	2.19139E-08	1.64432E-07

ENSG00000120333	MRPS14	0.64	2.20305E-08	1.65245E-07
ENSG00000160179	ABCG1	1.38	2.21091E-08	1.65772E-07
ENSG00000224470	ATXN1L	-0.61	2.22522E-08	1.66783E-07
ENSG00000102401	ARMCX3	0.72	2.27002E-08	1.70077E-07
ENSG00000213923	CSNK1E	0.41	2.34111E-08	1.75312E-07
ENSG00000112039	FANCE	-0.81	2.34163E-08	1.75312E-07
ENSG00000144426	NBEAL1	-0.41	2.35356E-08	1.76139E-07
ENSG00000140750	ARHGAP17	0.59	2.39135E-08	1.789E-07
ENSG00000115241	PPM1G	-0.37	2.39248E-08	1.78917E-07
ENSG00000248527	MTATP6P1	-0.44	2.39562E-08	1.79036E-07
ENSG00000168280	KIF5C	0.87	2.39585E-08	1.79036E-07
ENSG00000186994	KANK3	-1.00	2.40101E-08	1.79354E-07
ENSG00000008952	SEC62	0.52	2.40481E-08	1.79571E-07
ENSG00000158710	TAGLN2	0.78	2.40839E-08	1.79772E-07
ENSG00000225808	DNAJC19P5	3.08	2.42663E-08	1.81065E-07
ENSG00000116750	UCHL5	-0.50	2.44214E-08	1.82154E-07
ENSG00000121236	TRIM6	1.06	2.50122E-08	1.86492E-07
ENSG00000119900	OGFRL1	-0.93	2.51089E-08	1.87143E-07
ENSG00000145386	CCNA2	0.41	2.52039E-08	1.87782E-07
ENSG00000120837	NFYB	-0.78	2.52222E-08	1.87848E-07
ENSG00000115641	FHL2	-0.63	2.54132E-08	1.892E-07
ENSG00000125246	CLYBL	-1.15	2.56915E-08	1.91201E-07
ENSG00000146232	NFKBIE	0.81	2.58729E-08	1.92479E-07
ENSG00000040487	SLC66A1	0.86	2.61063E-08	1.94143E-07
ENSG00000159479	MED8	0.54	2.61368E-08	1.94298E-07
ENSG00000117036	ETV3	0.59	2.62274E-08	1.94837E-07
ENSG00000183780	SLC35F3	-0.96	2.62288E-08	1.94837E-07
ENSG00000177666	PNPLA2	-0.49	2.64941E-08	1.9669E-07
ENSG00000170779	CDCA4	-0.43	2.64979E-08	1.9669E-07
ENSG00000262003	AC087392.1	1.83	2.6626E-08	1.97568E-07
ENSG00000106692	FKTN	-0.99	2.69404E-08	1.99826E-07
ENSG00000104691	UBXN8	0.82	2.69998E-08	2.00193E-07
ENSG00000103227	LMF1	-1.52	2.71012E-08	2.0087E-07
ENSG00000146263	MMS22L	-0.67	2.76248E-08	2.04675E-07
ENSG00000174748	RPL15	-0.35	2.76519E-08	2.048E-07
ENSG00000155368	DBI	0.40	2.76976E-08	2.05063E-07
ENSG00000117000	RLF	0.67	2.78465E-08	2.06089E-07
ENSG00000006468	ETV1	0.73	2.78665E-08	2.06161E-07
ENSG00000148835	TAF5	0.80	2.80054E-08	2.07112E-07
ENSG00000234327	AC012146.1	0.87	2.80534E-08	2.0739E-07
ENSG00000260260	SNHG19	-0.69	2.84183E-08	2.1001E-07
ENSG00000108100	CCNY	-0.51	2.88947E-08	2.13452E-07
ENSG00000148680	HTR7	0.74	2.8978E-08	2.13988E-07
ENSG00000115137	DNAJC27	1.16	2.9138E-08	2.15091E-07
ENSG00000158055	GRHL3	3.42	2.9181E-08	2.15328E-07
ENSG00000063046	EIF4B	-0.31	2.92006E-08	2.15394E-07

ENSG00000014919	COX15	-0.49	2.93771E-08	2.16616E-07
ENSG00000214274	ANG	1.37	2.94236E-08	2.16878E-07
ENSG00000048991	R3HDM1	-0.52	2.95067E-08	2.17411E-07
ENSG00000172893	DHCR7	-0.51	2.98083E-08	2.19552E-07
ENSG00000102384	CENPI	0.87	2.98328E-08	2.19652E-07
ENSG00000129696	TTI2	0.69	2.99559E-08	2.20477E-07
ENSG00000277879	AL391988.1	1.64	3.02353E-08	2.22452E-07
ENSG00000235269	LINC02331	-1.39	3.04802E-08	2.24171E-07
ENSG00000124783	SSR1	0.38	3.09766E-08	2.27738E-07
ENSG00000143924	EML4	-0.57	3.12735E-08	2.29837E-07
ENSG00000117620	SLC35A3	-0.99	3.14066E-08	2.3073E-07
ENSG00000148344	PTGES	0.92	3.15645E-08	2.31805E-07
ENSG00000119402	FBXW2	-0.46	3.16585E-08	2.3241E-07
ENSG00000129911	KLF16	-0.56	3.17909E-08	2.33296E-07
ENSG00000134294	SLC38A2	0.38	3.22812E-08	2.36808E-07
ENSG00000164744	SUN3	1.02	3.24022E-08	2.37608E-07
ENSG00000109265	CRACD	0.86	3.25901E-08	2.38899E-07
ENSG00000106415	GLCCI1	1.28	3.26536E-08	2.39277E-07
ENSG00000153406	NMRAL1	-0.56	3.29084E-08	2.41056E-07
ENSG00000123415	SMUG1	-0.76	3.32277E-08	2.43305E-07
ENSG00000187720	THSD4	-1.07	3.33764E-08	2.44305E-07
ENSG00000161800	RACGAP1	0.41	3.33924E-08	2.44333E-07
ENSG00000100416	TRMU	0.49	3.3847E-08	2.47568E-07
ENSG00000143515	ATP8B2	0.51	3.4099E-08	2.4932E-07
ENSG00000144504	ANKMY1	-0.91	3.41621E-08	2.4969E-07
ENSG00000154114	TBCEL	-0.82	3.44649E-08	2.51812E-07
ENSG00000173545	ZNF622	0.58	3.46049E-08	2.52742E-07
ENSG00000214026	MRPL23	0.86	3.47361E-08	2.53608E-07
ENSG00000198832	SELENOM	0.66	3.50281E-08	2.55647E-07
ENSG00000110931	CAMKK2	-0.59	3.52446E-08	2.57133E-07
ENSG00000004779	NDUFAB1	0.41	3.74429E-08	2.73071E-07
ENSG00000087074	PPP1R15A	0.39	3.75557E-08	2.73794E-07
ENSG00000078808	SDF4	0.40	3.75756E-08	2.73839E-07
ENSG00000134684	YARS1	-0.34	3.78229E-08	2.75542E-07
ENSG00000196668	LINC00173	-0.96	3.80345E-08	2.76982E-07
ENSG00000147533	GOLGA7	0.80	3.90559E-08	2.84316E-07
ENSG00000277258	PCGF2	-0.75	3.95813E-08	2.88037E-07
ENSG00000204389	HSPA1A	1.72	4.03763E-08	2.93715E-07
ENSG00000082153	BZW1	-0.40	4.08743E-08	2.9723E-07
ENSG00000147439	BIN3	-0.90	4.11177E-08	2.98891E-07
ENSG00000085563	ABCB1	1.27	4.14326E-08	3.01071E-07
ENSG00000132522	GPS2	-0.89	4.18549E-08	3.04029E-07
ENSG00000011105	TSPAN9	0.60	4.19261E-08	3.04436E-07
ENSG00000177707	NECTIN3	-0.61	4.2134E-08	3.05771E-07
ENSG00000251095	AC093866.1	-1.89	4.21406E-08	3.05771E-07
ENSG00000196865	NHLRC2	-0.64	4.26653E-08	3.09466E-07

ENSG00000229944	EIF4EP2	0.80	4.28177E-08	3.10459E-07
ENSG00000115239	ASB3	0.84	4.29557E-08	3.11347E-07
ENSG00000164983	TMEM65	0.64	4.29865E-08	3.11458E-07
ENSG00000115295	CLIP4	0.61	4.30079E-08	3.115E-07
ENSG00000186834	HEXIM1	0.37	4.35008E-08	3.14956E-07
ENSG00000104375	STK3	0.64	4.35831E-08	3.15437E-07
ENSG00000106245	BUD31	0.43	4.37417E-08	3.16411E-07
ENSG00000166938	DIS3L	-0.74	4.37493E-08	3.16411E-07
ENSG00000239900	ADSL	-0.51	4.38802E-08	3.17136E-07
ENSG00000168273	SMIM4	1.03	4.38812E-08	3.17136E-07
ENSG00000182362	YBEY	-0.86	4.42107E-08	3.19403E-07
ENSG00000197043	ANXA6	0.38	4.4788E-08	3.23456E-07
ENSG00000074370	ATP2A3	-0.70	4.50352E-08	3.25124E-07
ENSG00000143315	PIGM	-0.94	4.54632E-08	3.28095E-07
ENSG00000147144	CCDC120	-1.11	4.5709E-08	3.29751E-07
ENSG00000164855	TMEM184A	-0.68	4.60326E-08	3.31966E-07
ENSG00000136940	PDCL	0.72	4.66097E-08	3.36007E-07
ENSG00000123384	LRP1	1.04	4.67073E-08	3.36588E-07
ENSG00000253710	ALG11	1.00	4.69769E-08	3.38409E-07
ENSG00000066654	THUMPD1	0.59	4.75612E-08	3.42496E-07
ENSG00000154920	EME1	-0.70	4.79903E-08	3.45461E-07
ENSG00000041802	LSG1	0.51	4.90091E-08	3.52668E-07
ENSG00000110768	GTF2H1	0.69	4.94849E-08	3.55964E-07
ENSG00000075618	FSCN1	0.38	4.95602E-08	3.56378E-07
ENSG00000146425	DYNLT1	-0.48	5.00301E-08	3.59628E-07
ENSG00000141279	NPEPPS	0.35	5.02485E-08	3.60939E-07
ENSG00000168092	PAFAH1B2	0.35	5.02486E-08	3.60939E-07
ENSG00000076351	SLC46A1	-1.10	5.05575E-08	3.63027E-07
ENSG00000111271	ACAD10	-0.84	5.07503E-08	3.64281E-07
ENSG00000107281	NPDC1	-0.59	5.09548E-08	3.65618E-07
ENSG00000025800	KPNA6	-0.39	5.13624E-08	3.6841E-07
ENSG00000078900	TP73	-0.83	5.15285E-08	3.69469E-07
ENSG00000132507	EIF5A	-0.38	5.15676E-08	3.69617E-07
ENSG00000112110	MRPL18	0.49	5.19055E-08	3.71906E-07
ENSG00000150093	ITGB1	0.38	5.20541E-08	3.72837E-07
ENSG00000001036	FUCA2	0.38	5.25949E-08	3.76576E-07
ENSG00000169641	LUZP1	-0.61	5.2652E-08	3.76849E-07
ENSG00000260368	AC027373.1	3.23	5.27251E-08	3.77238E-07
ENSG00000152219	ARL14EP	-0.94	5.29989E-08	3.79062E-07
ENSG00000160190	SLC37A1	0.76	5.31278E-08	3.79848E-07
ENSG00000124702	KLHDC3	0.49	5.36883E-08	3.8367E-07
ENSG00000085719	CPNE3	-0.51	5.37008E-08	3.8367E-07
ENSG00000116745	RPE65	-1.25	5.40355E-08	3.85924E-07
ENSG00000165169	DYNLT3	0.73	5.4164E-08	3.86704E-07
ENSG00000196950	SLC39A10	0.66	5.42339E-08	3.87064E-07
ENSG00000076053	RBM7	0.76	5.43376E-08	3.87666E-07

ENSG00000245648	AC022075.1	-1.95	5.63505E-08	4.01884E-07
ENSG00000110200	ANAPC15	0.78	5.80494E-08	4.13853E-07
ENSG00000237649	KIFC1	0.41	5.83773E-08	4.15943E-07
ENSG00000168477	TNXB	-1.58	5.83841E-08	4.15943E-07
ENSG00000141480	ARRB2	-0.51	5.85881E-08	4.17247E-07
ENSG00000171448	ZBTB26	-1.26	5.89717E-08	4.1983E-07
ENSG00000078687	TNRC6C	0.72	5.90286E-08	4.20001E-07
ENSG00000065833	ME1	0.43	5.90377E-08	4.20001E-07
ENSG00000107338	SHB	-0.81	5.92184E-08	4.21137E-07
ENSG00000148737	TCF7L2	-0.42	6.03141E-08	4.28776E-07
ENSG00000116791	CRYZ	0.63	6.15542E-08	4.37437E-07
ENSG00000015475	BID	0.57	6.17223E-08	4.38476E-07
ENSG00000133121	STARD13	1.34	6.17486E-08	4.38507E-07
ENSG00000169155	ZBTB43	0.73	6.2328E-08	4.42464E-07
ENSG00000137996	RTCA	0.58	6.23556E-08	4.42504E-07
ENSG00000029993	HMGB3	-0.39	6.24193E-08	4.42799E-07
ENSG00000179295	PTPN11	-0.37	6.33303E-08	4.49102E-07
ENSG00000135365	PHF21A	0.57	6.34618E-08	4.49875E-07
ENSG00000142784	WDTC1	-0.61	6.36987E-08	4.51394E-07
ENSG00000186577	SMIM29	-1.00	6.39635E-08	4.5311E-07
ENSG00000175105	ZNF654	1.09	6.39956E-08	4.53177E-07
ENSG00000222009	BTBD19	-1.79	6.42278E-08	4.54661E-07
ENSG00000128578	STRIP2	-0.94	6.43559E-08	4.55336E-07
ENSG00000176903	PNMA1	-0.63	6.43687E-08	4.55336E-07
ENSG00000163510	CWC22	0.66	6.49497E-08	4.59284E-07
ENSG00000180739	S1PR5	1.13	6.52245E-08	4.61064E-07
ENSG00000152620	NADK2	-0.81	6.54657E-08	4.62605E-07
ENSG00000101199	ARFGAP1	-0.47	6.57265E-08	4.64285E-07
ENSG00000185090	MANEAL	-0.86	6.5898E-08	4.65332E-07
ENSG00000215712	TMEM242	0.71	6.59924E-08	4.65834E-07
ENSG00000117054	ACADM	0.51	6.60719E-08	4.66231E-07
ENSG00000078177	N4BP2	1.11	6.6471E-08	4.68881E-07
ENSG00000132109	TRIM21	0.92	6.92337E-08	4.88197E-07
ENSG00000102977	ACD	-0.60	6.93701E-08	4.88987E-07
ENSG00000119684	MLH3	0.96	6.95746E-08	4.90256E-07
ENSG00000166272	WBP1L	0.54	7.0412E-08	4.95982E-07
ENSG00000132485	ZRANB2	-0.47	7.11453E-08	5.00971E-07
ENSG00000138029	HADHB	0.44	7.14078E-08	5.02643E-07
ENSG00000198000	NOL8	-0.80	7.18076E-08	5.05279E-07
ENSG00000166851	PLK1	-0.39	7.21522E-08	5.07525E-07
ENSG00000117385	P3H1	0.53	7.26872E-08	5.11109E-07
ENSG00000214279	SCART1	-2.28	7.28889E-08	5.12347E-07
ENSG00000039523	RIPOR1	-0.65	7.34981E-08	5.16448E-07
ENSG00000057757	PITHD1	0.44	7.36744E-08	5.17505E-07
ENSG00000018236	CNTN1	1.85	7.44089E-08	5.22481E-07
ENSG00000149091	DGKZ	-0.53	7.49666E-08	5.26213E-07

ENSG00000198089	SFI1	-0.70	7.52214E-08	5.27816E-07
ENSG00000143776	CDC42BPA	-0.51	7.56245E-08	5.30458E-07
ENSG00000103168	TAF1C	-0.51	7.56722E-08	5.30607E-07
ENSG00000197147	LRRC8B	-0.92	7.57388E-08	5.30888E-07
ENSG00000177885	GRB2	0.33	7.6036E-08	5.32785E-07
ENSG00000079337	RAPGEF3	2.41	7.64312E-08	5.35366E-07
ENSG00000245025	AC107959.1	1.72	7.69914E-08	5.39102E-07
ENSG00000131475	VPS25	0.45	7.79123E-08	5.45359E-07
ENSG00000116251	RPL22	-0.32	7.81694E-08	5.46967E-07
ENSG00000153044	CENPH	0.80	7.88387E-08	5.51458E-07
ENSG00000151914	DST	0.37	7.93104E-08	5.54563E-07
ENSG00000172164	SNTB1	-1.52	8.0216E-08	5.60699E-07
ENSG00000135679	MDM2	0.40	8.14941E-08	5.69434E-07
ENSG00000136378	ADAMTS7	-1.21	8.22985E-08	5.74854E-07
ENSG00000155846	PPARGC1B	-0.89	8.35014E-08	5.83053E-07
ENSG00000111684	LPCAT3	-0.78	8.37667E-08	5.84701E-07
ENSG00000008838	MED24	-0.46	8.4202E-08	5.87535E-07
ENSG00000105290	APLP1	0.55	8.43933E-08	5.88664E-07
ENSG00000186280	KDM4D	1.53	8.47372E-08	5.90858E-07
ENSG00000100348	TXN2	-0.52	8.65186E-08	6.03069E-07
ENSG00000111247	RAD51AP1	-0.81	8.84208E-08	6.16113E-07
ENSG00000150867	PIP4K2A	0.44	8.91648E-08	6.21081E-07
ENSG00000059378	PARP12	-0.72	8.94075E-08	6.22555E-07
ENSG00000111229	ARPC3	0.45	8.96601E-08	6.24097E-07
ENSG00000123739	PLA2G12A	-0.74	9.01526E-08	6.27307E-07
ENSG00000129128	SPCS3	-0.96	9.16896E-08	6.3778E-07
ENSG00000130005	GAMT	-0.54	9.21253E-08	6.40588E-07
ENSG00000218336	TENM3	1.26	9.259E-08	6.43517E-07
ENSG00000133193	FAM104A	0.52	9.26109E-08	6.43517E-07
ENSG00000005175	RPAP3	0.67	9.34103E-08	6.48846E-07
ENSG00000105617	LENG1	0.88	9.41893E-08	6.54031E-07
ENSG00000079462	PAFAH1B3	0.46	9.42558E-08	6.54266E-07
ENSG00000126215	XRCC3	-0.63	9.47819E-08	6.57689E-07
ENSG00000080802	CNOT4	0.74	9.54193E-08	6.61882E-07
ENSG00000125875	TBC1D20	-0.45	9.60876E-08	6.66287E-07
ENSG00000164180	TMEM161B	-0.88	9.64194E-08	6.68356E-07
ENSG00000131037	EPS8L1	0.72	9.79064E-08	6.78429E-07
ENSG00000179546	HTR1D	-1.18	9.80197E-08	6.78979E-07
ENSG00000086189	DIMT1	-0.73	9.83952E-08	6.81344E-07
ENSG00000255152	MSH5-SAPCD1	-1.39	9.87124E-08	6.83304E-07
ENSG00000135842	NIBAN1	-0.64	9.91106E-08	6.85823E-07
ENSG00000185298	CCDC137	0.40	9.92021E-08	6.8622E-07
ENSG00000172936	MYD88	-0.91	9.99549E-08	6.91188E-07
ENSG00000182149	IST1	0.37	1.00153E-07	6.92318E-07
ENSG00000172731	LRRC20	-0.59	1.00757E-07	6.96251E-07
ENSG00000280120	AC073857.1	-1.50	1.01232E-07	6.99292E-07

ENSG00000130312	MRPL34	-0.62	1.03362E-07	7.13763E-07
ENSG00000156976	EIF4A2	-0.35	1.04058E-07	7.18319E-07
ENSG00000188707	ZBED6CL	-1.58	1.0483E-07	7.234E-07
ENSG00000172901	LVRN	2.44	1.05469E-07	7.27557E-07
ENSG00000125650	PSPN	-1.74	1.05648E-07	7.28539E-07
ENSG00000130766	SESN2	-0.48	1.06422E-07	7.33628E-07
ENSG00000164647	STEAP1	-0.72	1.07021E-07	7.37498E-07
ENSG00000115216	NRBP1	-0.43	1.07254E-07	7.38851E-07
ENSG00000213516	RBMXL1	-0.89	1.07667E-07	7.41442E-07
ENSG00000102554	KLF5	0.52	1.07912E-07	7.42684E-07
ENSG00000138430	OLA1	0.43	1.07921E-07	7.42684E-07
ENSG00000183655	KLHL25	0.92	1.08259E-07	7.44747E-07
ENSG00000159256	MORC3	0.72	1.09452E-07	7.52698E-07
ENSG00000145439	CBR4	-0.68	1.10027E-07	7.56219E-07
ENSG00000125637	PSD4	-1.07	1.10039E-07	7.56219E-07
ENSG00000129472	RAB2B	0.65	1.10423E-07	7.58597E-07
ENSG00000069275	NUCKS1	-0.35	1.10953E-07	7.61974E-07
ENSG00000130821	SLC6A8	-0.43	1.12925E-07	7.7525E-07
ENSG00000125037	EMC3	0.83	1.13038E-07	7.75759E-07
ENSG00000188895	MSL1	0.45	1.14344E-07	7.84453E-07
ENSG00000173542	MOB1B	0.58	1.14497E-07	7.85232E-07
ENSG00000189007	ADAT2	-0.83	1.14849E-07	7.87381E-07
ENSG00000163191	S100A11	0.44	1.15125E-07	7.88999E-07
ENSG00000188177	ZC3H6	-1.64	1.15514E-07	7.91398E-07
ENSG00000164181	ELOVL7	-0.75	1.15687E-07	7.92309E-07
ENSG00000124209	RAB22A	0.65	1.16E-07	7.94183E-07
ENSG00000162813	BPNT1	0.63	1.16046E-07	7.94225E-07
ENSG00000151743	AMN1	0.73	1.17428E-07	8.03406E-07
ENSG00000029364	SLC39A9	0.39	1.18335E-07	8.0934E-07
ENSG00000132563	REEP2	1.10	1.18488E-07	8.10105E-07
ENSG00000198146	ZNF770	-0.73	1.18817E-07	8.12079E-07
ENSG00000147123	NDUFB11	0.44	1.20235E-07	8.21491E-07
ENSG00000151693	ASAP2	0.75	1.20543E-07	8.23314E-07
ENSG00000270977	AC015849.5	-2.33	1.211E-07	8.26835E-07
ENSG00000185896	LAMP1	0.39	1.21477E-07	8.29127E-07
ENSG00000123146	ADGRE5	0.40	1.22535E-07	8.36064E-07
ENSG00000094880	CDC23	0.50	1.22705E-07	8.3694E-07
ENSG00000272077	AC124045.1	-2.57	1.22798E-07	8.37286E-07
ENSG00000145555	MYO10	0.47	1.23333E-07	8.4065E-07
ENSG00000171488	LRRC8C	-0.86	1.2393E-07	8.44431E-07
ENSG00000132837	DMGDH	-2.97	1.24698E-07	8.4937E-07
ENSG00000165688	PMPCA	0.45	1.25658E-07	8.55622E-07
ENSG00000164088	PPM1M	-0.80	1.26282E-07	8.59575E-07
ENSG00000136261	BZW2	-0.40	1.27601E-07	8.68257E-07
ENSG00000131236	CAP1	0.33	1.28025E-07	8.70845E-07
ENSG00000198431	TXNRD1	0.32	1.28819E-07	8.7595E-07

ENSG00000111490	TBC1D30	-1.27	1.30313E-07	8.85811E-07
ENSG00000125821	DTD1	-0.63	1.30448E-07	8.86424E-07
ENSG00000128683	GAD1	1.05	1.32036E-07	8.96912E-07
ENSG00000287562	AL109615.4	-2.49	1.33579E-07	9.07087E-07
ENSG00000173715	C11orf80	0.87	1.33643E-07	9.07213E-07
ENSG00000078114	NEBL	-0.82	1.33784E-07	9.07859E-07
ENSG00000136810	TXN	0.32	1.36074E-07	9.23087E-07
ENSG00000130958	SLC35D2	0.63	1.36142E-07	9.23235E-07
ENSG00000129422	MTUS1	-0.46	1.36345E-07	9.24303E-07
ENSG00000261253	AC137932.2	2.54	1.37722E-07	9.33052E-07
ENSG00000105518	TMEM205	0.56	1.37729E-07	9.33052E-07
ENSG00000204138	PHACTR4	-0.55	1.38349E-07	9.36934E-07
ENSG00000267424	AC020934.1	1.98	1.3978E-07	9.46304E-07
ENSG00000123473	STIL	0.56	1.42187E-07	9.62272E-07
ENSG00000244462	RBM12	-0.40	1.42929E-07	9.66969E-07
ENSG00000262454	MIR193BHG	1.49	1.47981E-07	1.00081E-06
ENSG00000158716	DUSP23	-1.09	1.48956E-07	1.00706E-06
ENSG00000037965	HOXC8	-3.33	1.49233E-07	1.00847E-06
ENSG00000154978	VOPP1	0.52	1.49265E-07	1.00847E-06
ENSG00000117519	CNN3	0.37	1.49704E-07	1.01109E-06
ENSG00000186318	BACE1	-1.23	1.50178E-07	1.01396E-06
ENSG00000148057	IDNK	-3.99	1.50776E-07	1.01765E-06
ENSG00000155090	KLF10	0.43	1.52572E-07	1.02942E-06
ENSG00000023734	STRAP	0.33	1.53261E-07	1.03372E-06
ENSG00000137504	CREBZF	-0.60	1.55624E-07	1.04931E-06
ENSG00000133627	ACTR3B	-0.79	1.56095E-07	1.05213E-06
ENSG00000006282	SPATA20	-0.59	1.56176E-07	1.05232E-06
ENSG00000055950	MRPL43	0.44	1.5701E-07	1.05758E-06
ENSG00000176155	CCDC57	-0.48	1.60252E-07	1.07906E-06
ENSG00000137261	KIAA0319	1.30	1.61927E-07	1.08997E-06
ENSG00000188811	NHLRC3	0.75	1.62191E-07	1.09128E-06
ENSG00000180921	FAM83H	-0.39	1.6223E-07	1.09128E-06
ENSG00000157833	GAREM2	-0.86	1.64022E-07	1.10296E-06
ENSG00000185507	IRF7	0.99	1.64081E-07	1.10299E-06
ENSG00000130513	GDF15	-0.39	1.64194E-07	1.10338E-06
ENSG00000141556	TBCD	0.37	1.64267E-07	1.1035E-06
ENSG00000130935	NOL11	0.41	1.6646E-07	1.11784E-06
ENSG00000158863	FAM160B2	-0.64	1.66514E-07	1.11784E-06
ENSG00000176407	KCMF1	0.43	1.68395E-07	1.13009E-06
ENSG00000102580	DNAJC3	0.56	1.69803E-07	1.13915E-06
ENSG00000087077	TRIP6	0.40	1.70559E-07	1.14384E-06
ENSG00000104529	EEF1D	-0.35	1.71132E-07	1.1473E-06
ENSG00000260257	AL035071.1	-1.18	1.71376E-07	1.14856E-06
ENSG00000126773	PCNX4	-0.71	1.73618E-07	1.16319E-06
ENSG00000162517	PEF1	-0.55	1.74123E-07	1.16618E-06
ENSG00000168036	CTNNB1	0.36	1.75375E-07	1.17417E-06

ENSG00000217128	FNIP1	0.78	1.76012E-07	1.17805E-06
ENSG00000213983	AP1G2	-0.63	1.77001E-07	1.18427E-06
ENSG00000124733	MEA1	-0.45	1.77466E-07	1.18698E-06
ENSG00000149150	SLC43A1	-1.03	1.80153E-07	1.20455E-06
ENSG00000186871	ERCC6L	0.78	1.8216E-07	1.21756E-06
ENSG00000204310	AGPAT1	-0.50	1.82632E-07	1.22031E-06
ENSG00000087338	GMCL1	-0.61	1.84777E-07	1.23423E-06
ENSG00000069509	FUNDC1	0.81	1.85133E-07	1.2362E-06
ENSG00000163399	ATP1A1	-0.30	1.8555E-07	1.23857E-06
ENSG00000160445	ZER1	-0.74	1.85689E-07	1.23908E-06
ENSG00000177192	PUS1	-0.51	1.88932E-07	1.26031E-06
ENSG00000084636	COL16A1	1.55	1.91407E-07	1.27639E-06
ENSG00000196312	MFSD14C	-0.57	1.91727E-07	1.2781E-06
ENSG00000066322	ELOVL1	0.43	1.92189E-07	1.28075E-06
ENSG00000147536	GINS4	-0.69	1.92647E-07	1.28337E-06
ENSG00000135048	CEMIP2	0.52	1.94644E-07	1.29625E-06
ENSG00000140450	ARRDC4	-1.42	1.94846E-07	1.29716E-06
ENSG00000169902	TPST1	0.71	1.95011E-07	1.29783E-06
ENSG00000102763	VWA8	-0.71	1.98538E-07	1.32086E-06
ENSG00000213741	RPS29	-0.37	1.9877E-07	1.32196E-06
ENSG00000184787	UBE2G2	-0.39	1.9965E-07	1.32737E-06
ENSG00000095139	ARCN1	0.34	2.01402E-07	1.33858E-06
ENSG00000101407	TTI1	-0.60	2.04281E-07	1.35726E-06
ENSG00000140983	RHOT2	-0.55	2.05324E-07	1.36374E-06
ENSG00000109184	DCUN1D4	0.50	2.0628E-07	1.36964E-06
ENSG00000083307	GRHL2	-1.15	2.06627E-07	1.37148E-06
ENSG00000114698	PLSCR4	1.48	2.0759E-07	1.37742E-06
ENSG00000052802	MSMO1	0.43	2.11368E-07	1.40202E-06
ENSG00000184207	PGP	-0.50	2.1735E-07	1.44122E-06
ENSG00000107521	HPS1	-0.58	2.17477E-07	1.44159E-06
ENSG00000141756	FKBP10	-0.50	2.1758E-07	1.44179E-06
ENSG00000127603	MACF1	0.39	2.19581E-07	1.45457E-06
ENSG00000069974	RAB27A	0.98	2.20171E-07	1.458E-06
ENSG00000198034	RPS4X	-0.31	2.21028E-07	1.46319E-06
ENSG00000143190	POU2F1	-0.59	2.22915E-07	1.47519E-06
ENSG00000164253	WDR41	0.47	2.2382E-07	1.48069E-06
ENSG00000170619	COMMD5	0.65	2.24604E-07	1.4851E-06
ENSG00000004478	FKBP4	0.32	2.24635E-07	1.4851E-06
ENSG00000138606	SHF	2.00	2.24768E-07	1.48549E-06
ENSG00000165359	INTS6L	1.16	2.27958E-07	1.50607E-06
ENSG00000145901	TNIP1	-0.56	2.2848E-07	1.50903E-06
ENSG00000177494	ZBED2	-0.86	2.30426E-07	1.52137E-06
ENSG00000103051	COG4	-0.48	2.38569E-07	1.57462E-06
ENSG00000111269	CREBL2	0.68	2.41716E-07	1.59486E-06
ENSG00000122971	ACADS	-0.78	2.42324E-07	1.59835E-06
ENSG00000088833	NSFL1C	0.39	2.43923E-07	1.60837E-06

ENSG00000125449	ARMC7	0.59	2.48639E-07	1.63892E-06
ENSG00000133706	LARS1	-0.36	2.4886E-07	1.63984E-06
ENSG00000204152	TIMM23B	-1.24	2.52397E-07	1.6626E-06
ENSG00000154134	ROBO3	-0.70	2.53356E-07	1.66837E-06
ENSG00000095319	NUP188	-0.35	2.54041E-07	1.67233E-06
ENSG00000100908	EMC9	-0.62	2.55399E-07	1.68071E-06
ENSG00000120129	DUSP1	0.87	2.55576E-07	1.68132E-06
ENSG00000130202	NECTIN2	-0.43	2.6207E-07	1.72326E-06
ENSG00000177432	NAP1L5	1.38	2.62123E-07	1.72326E-06
ENSG00000172086	KRCC1	-1.83	2.62973E-07	1.72828E-06
ENSG00000124299	PEPD	-0.55	2.64979E-07	1.7409E-06
ENSG00000140391	TSPAN3	0.37	2.66647E-07	1.75127E-06
ENSG00000163029	SMC6	-0.53	2.69659E-07	1.77048E-06
ENSG00000160293	VAV2	-0.49	2.69808E-07	1.77087E-06
ENSG00000173011	TADA2B	0.74	2.71879E-07	1.78388E-06
ENSG00000071073	MGAT4A	0.97	2.76026E-07	1.8105E-06
ENSG00000175832	ETV4	0.48	2.76388E-07	1.81228E-06
ENSG00000079950	STX7	-0.66	2.77429E-07	1.81851E-06
ENSG00000039068	CDH1	-0.45	2.79323E-07	1.83033E-06
ENSG00000197976	AKAP17A	0.58	2.7949E-07	1.83082E-06
ENSG00000164236	ANKRD33B	0.65	2.82303E-07	1.84823E-06
ENSG00000105173	CCNE1	-0.68	2.82333E-07	1.84823E-06
ENSG00000173210	ABLIM3	-0.71	2.8304E-07	1.85226E-06
ENSG00000129559	NEDD8	-0.43	2.8382E-07	1.85675E-06
ENSG00000000003	TSPAN6	-0.65	2.8447E-07	1.86039E-06
ENSG00000240875	LINC00886	-2.98	2.8901E-07	1.88947E-06
ENSG00000126777	KTN1	-0.45	2.90312E-07	1.89736E-06
ENSG00000106479	ZNF862	-1.31	2.93194E-07	1.91557E-06
ENSG00000101166	PRELID3B	-0.43	2.93888E-07	1.91948E-06
ENSG00000204977	TRIM13	-0.79	2.97513E-07	1.94252E-06
ENSG00000169169	CPT1C	1.06	2.98358E-07	1.94741E-06
ENSG00000167380	ZNF226	0.86	3.04075E-07	1.98407E-06
ENSG00000281406	BLACAT1	-1.01	3.05825E-07	1.9945E-06
ENSG00000119013	NDUFB3	0.51	3.05872E-07	1.9945E-06
ENSG00000076716	GPC4	0.58	3.07854E-07	2.00676E-06
ENSG00000211584	SLC48A1	0.63	3.08218E-07	2.00849E-06
ENSG00000128708	HAT1	0.45	3.09504E-07	2.01621E-06
ENSG00000087088	BAX	-0.51	3.13734E-07	2.0431E-06
ENSG00000077348	EXOSC5	-0.59	3.14065E-07	2.04459E-06
ENSG00000110435	PDHX	0.59	3.14241E-07	2.04507E-06
ENSG00000244045	TMEM199	0.76	3.15223E-07	2.05079E-06
ENSG00000178104	PDE4DIP	0.74	3.17266E-07	2.06342E-06
ENSG00000132196	HSD17B7	0.83	3.19188E-07	2.07524E-06
ENSG00000130158	DOCK6	-0.60	3.19507E-07	2.07664E-06
ENSG00000067992	PDK3	-0.72	3.19798E-07	2.07786E-06
ENSG00000188846	RPL14	-0.31	3.21009E-07	2.08505E-06

ENSG00000176715	ACSF3	-0.51	3.25832E-07	2.11569E-06
ENSG00000188690	UROS	-0.54	3.26033E-07	2.1162E-06
ENSG00000081377	CDC14B	-0.71	3.26123E-07	2.1162E-06
ENSG00000184277	TM2D3	0.57	3.28695E-07	2.13221E-06
ENSG00000188486	H2AX	-0.38	3.29635E-07	2.13761E-06
ENSG00000173950	XXYLT1	0.81	3.31416E-07	2.14846E-06
ENSG00000117479	SLC19A2	-0.71	3.32521E-07	2.15493E-06
ENSG00000135736	CCDC102A	1.15	3.32919E-07	2.15681E-06
ENSG00000179119	SPTY2D1	0.58	3.33884E-07	2.16236E-06
ENSG00000081189	MEF2C	1.20	3.37356E-07	2.18414E-06
ENSG00000128626	MRPS12	-0.55	3.38247E-07	2.1892E-06
ENSG00000077514	POLD3	-0.88	3.41198E-07	2.20758E-06
ENSG00000107949	BCCIP	0.40	3.42421E-07	2.21478E-06
ENSG00000189266	PNRC2	0.43	3.43642E-07	2.22196E-06
ENSG00000143493	INTS7	0.52	3.43903E-07	2.22293E-06
ENSG00000164663	USP49	-0.85	3.4592E-07	2.23524E-06
ENSG00000175711	B3GNTL1	0.69	3.46758E-07	2.23993E-06
ENSG00000141258	SGSM2	-0.49	3.47644E-07	2.24494E-06
ENSG00000204922	UQCC3	-0.71	3.48815E-07	2.25177E-06
ENSG00000124615	MOCS1	-0.97	3.50305E-07	2.26066E-06
ENSG00000176454	LPCAT4	-0.64	3.5387E-07	2.28293E-06
ENSG00000023191	RNH1	0.38	3.59054E-07	2.31563E-06
ENSG00000033170	FUT8	0.78	3.61584E-07	2.33119E-06
ENSG00000114503	NCBP2	0.41	3.62959E-07	2.3393E-06
ENSG00000116062	MSH6	-0.40	3.63416E-07	2.34149E-06
ENSG00000135632	SMYD5	-0.49	3.65937E-07	2.35686E-06
ENSG00000179967	PPP1R14BP3	0.59	3.66036E-07	2.35686E-06
ENSG00000159445	THEM4	-0.70	3.67511E-07	2.36559E-06
ENSG00000065328	MCM10	-0.52	3.67962E-07	2.36773E-06
ENSG00000001497	LAS1L	-0.44	3.68804E-07	2.37239E-06
ENSG00000123575	FAM199X	0.50	3.71324E-07	2.38783E-06
ENSG00000235954	TTC28-AS1	0.86	3.74322E-07	2.40634E-06
ENSG00000050820	BCAR1	0.46	3.74458E-07	2.40644E-06
ENSG00000182871	COL18A1	-0.40	3.75957E-07	2.4153E-06
ENSG00000108561	C1QBP	-0.31	3.78905E-07	2.43345E-06
ENSG00000090975	PITPNM2	-0.79	3.82171E-07	2.45364E-06
ENSG00000126562	WNK4	-1.91	3.85552E-07	2.47456E-06
ENSG00000139697	SBNO1	0.48	3.85682E-07	2.4746E-06
ENSG00000167280	ENGASE	0.54	3.85842E-07	2.47483E-06
ENSG00000108592	FTSJ3	-0.41	3.88111E-07	2.48858E-06
ENSG00000102225	CDK16	-0.38	3.89866E-07	2.49904E-06
ENSG00000135549	PKIB	1.22	3.90086E-07	2.49965E-06
ENSG00000158201	ABHD3	0.57	3.92681E-07	2.51547E-06
ENSG00000144566	RAB5A	0.55	3.95365E-07	2.53186E-06
ENSG00000101843	PSMD10	0.44	3.98898E-07	2.55366E-06
ENSG00000095787	WAC	0.36	3.99194E-07	2.55474E-06

ENSG00000254681	PKD1P5	-1.10	4.01164E-07	2.56653E-06
ENSG00000143756	FBXO28	0.53	4.01322E-07	2.56672E-06
ENSG00000077585	GPR137B	1.00	4.03407E-07	2.57923E-06
ENSG00000104427	ZC2HC1A	-0.98	4.0563E-07	2.59261E-06
ENSG00000124444	ZNF576	0.94	4.05886E-07	2.59342E-06
ENSG00000152926	ZNF117	1.21	4.0963E-07	2.61609E-06
ENSG00000140548	ZNF710	-1.04	4.09695E-07	2.61609E-06
ENSG00000280193	AC132219.2	-2.65	4.10299E-07	2.61911E-06
ENSG00000113732	ATP6V0E1	0.43	4.13855E-07	2.64096E-06
ENSG00000166169	POLL	-0.65	4.17078E-07	2.66068E-06
ENSG00000276570	AC010327.6	-2.00	4.19295E-07	2.67397E-06
ENSG00000109771	LRP2BP	0.98	4.2662E-07	2.71902E-06
ENSG00000058453	CROCC	-0.74	4.2663E-07	2.71902E-06
ENSG00000114353	GNAI2	0.34	4.29882E-07	2.73887E-06
ENSG00000188878	FBF1	-1.09	4.30685E-07	2.74312E-06
ENSG00000257497	AC121761.1	1.49	4.32463E-07	2.75356E-06
ENSG00000270157	AC004918.3	2.14	4.32871E-07	2.75529E-06
ENSG00000266402	SNHG25	-2.05	4.35456E-07	2.77086E-06
ENSG00000119048	UBE2B	0.43	4.3609E-07	2.77401E-06
ENSG00000165416	SUGT1	0.43	4.38354E-07	2.78752E-06
ENSG00000198721	ECI2	0.49	4.41034E-07	2.80367E-06
ENSG00000162076	FLYWCH2	-0.80	4.41886E-07	2.8082E-06
ENSG00000136504	KAT7	-0.43	4.44983E-07	2.82698E-06
ENSG00000072310	SREBF1	0.34	4.52315E-07	2.87201E-06
ENSG00000169016	E2F6	0.63	4.52358E-07	2.87201E-06
ENSG00000006118	TMEM132A	-0.41	4.5507E-07	2.88832E-06
ENSG00000188976	NOC2L	-0.34	4.56708E-07	2.89779E-06
ENSG00000141499	WRAP53	0.54	4.60843E-07	2.9231E-06
ENSG00000146701	MDH2	-0.38	4.6189E-07	2.92881E-06
ENSG00000178425	NT5DC1	-0.62	4.62641E-07	2.93265E-06
ENSG00000143578	CREB3L4	-1.05	4.6359E-07	2.93774E-06
ENSG00000151718	WWC2	-0.72	4.72772E-07	2.99497E-06
ENSG00000263528	IKBKE	1.17	4.73759E-07	3.00028E-06
ENSG00000109534	GAR1	0.47	4.7407E-07	3.0013E-06
ENSG00000100802	C14orf93	-1.15	4.78353E-07	3.02745E-06
ENSG00000110881	ASIC1	-0.58	4.79735E-07	3.03524E-06
ENSG00000014824	SLC30A9	-0.50	4.80726E-07	3.04055E-06
ENSG00000230202	AL450405.1	-0.35	4.84776E-07	3.06519E-06
ENSG00000237523	LINC00857	0.74	4.85753E-07	3.0704E-06
ENSG00000163644	PPM1K	1.08	4.88125E-07	3.08442E-06
ENSG00000106780	MEGF9	0.76	4.90636E-07	3.09931E-06
ENSG00000027697	IFNGR1	0.68	4.94558E-07	3.12309E-06
ENSG00000167220	HDHD2	-1.35	4.94837E-07	3.12387E-06
ENSG00000164506	STXBP5	0.82	5.03785E-07	3.17936E-06
ENSG00000198816	ZNF358	-1.08	5.04822E-07	3.1849E-06
ENSG00000123353	ORMDL2	0.61	5.05098E-07	3.18564E-06

ENSG00000188342	GTF2F2	0.48	5.09526E-07	3.21255E-06
ENSG00000110880	CORO1C	0.34	5.1022E-07	3.21591E-06
ENSG00000087087	SRRT	0.33	5.10767E-07	3.21834E-06
ENSG00000240344	PPIL3	0.57	5.13065E-07	3.23181E-06
ENSG00000139842	CUL4A	-0.41	5.15206E-07	3.24428E-06
ENSG00000105643	ARRDC2	0.56	5.16165E-07	3.24929E-06
ENSG00000166669	ATF7IP2	0.99	5.19043E-07	3.26638E-06
ENSG00000118007	STAG1	0.62	5.25097E-07	3.30344E-06
ENSG00000100239	PPP6R2	0.39	5.27876E-07	3.3196E-06
ENSG00000129484	PARP2	-0.46	5.27998E-07	3.3196E-06
ENSG00000229544	NKX1-2	1.14	5.28402E-07	3.3211E-06
ENSG00000164011	ZNF691	-0.99	5.37744E-07	3.37876E-06
ENSG00000159111	MRPL10	-0.48	5.45114E-07	3.42399E-06
ENSG00000150760	DOCK1	-0.55	5.46847E-07	3.43324E-06
ENSG00000156735	BAG4	-0.73	5.46931E-07	3.43324E-06
ENSG00000047634	SCML1	0.68	5.59647E-07	3.51196E-06
ENSG00000198369	SPRED2	0.39	5.64526E-07	3.54147E-06
ENSG00000119185	ITGB1BP1	0.47	5.68715E-07	3.56663E-06
ENSG00000196981	WDR5B	-1.17	5.6909E-07	3.56787E-06
ENSG00000128731	HERC2	-0.40	5.69837E-07	3.57143E-06
ENSG00000143751	SDE2	0.49	5.75312E-07	3.60462E-06
ENSG00000039123	MTREX	0.44	5.75857E-07	3.6069E-06
ENSG00000168256	NKIRAS2	0.48	5.76789E-07	3.61161E-06
ENSG00000113643	RARS1	0.49	5.77022E-07	3.61194E-06
ENSG00000280088	AC126474.2	-3.13	5.84248E-07	3.65603E-06
ENSG00000165898	ISCA2	-0.62	5.85169E-07	3.66064E-06
ENSG00000173638	SLC19A1	0.66	5.88225E-07	3.67861E-06
ENSG00000158856	DMTN	-0.51	5.90168E-07	3.68961E-06
ENSG00000205517	RGL3	-0.65	5.93089E-07	3.70671E-06
ENSG00000103423	DNAJA3	-0.37	6.01053E-07	3.75531E-06
ENSG00000133433	GSTT2B	-1.01	6.0131E-07	3.75575E-06
ENSG00000005022	SLC25A5	-0.37	6.09017E-07	3.8027E-06
ENSG00000136381	IREB2	0.48	6.1247E-07	3.82306E-06
ENSG00000147459	DOCK5	-0.47	6.13995E-07	3.83139E-06
ENSG00000164654	MIOS	-0.79	6.15953E-07	3.84241E-06
ENSG00000167081	PBX3	0.89	6.21144E-07	3.87358E-06
ENSG00000188153	COL4A5	0.73	6.23778E-07	3.8888E-06
ENSG00000156232	WHAMM	0.74	6.25123E-07	3.89597E-06
ENSG00000198899	MT-ATP6	-0.45	6.26359E-07	3.90186E-06
ENSG00000115317	HTRA2	0.58	6.26458E-07	3.90186E-06
ENSG00000197182	MIRLET7BHG	1.17	6.33801E-07	3.94637E-06
ENSG00000114378	HYAL1	-1.30	6.45417E-07	4.01745E-06
ENSG00000178531	CTXN1	0.69	6.55111E-07	4.07652E-06
ENSG00000104213	PDGFRL	0.95	6.6018E-07	4.10642E-06
ENSG00000165861	ZFYVE1	0.80	6.60327E-07	4.10642E-06
ENSG00000198888	MT-ND1	0.45	6.66299E-07	4.14228E-06

ENSG00000163291	PAQR3	-0.62	6.71211E-07	4.17151E-06
ENSG00000130363	RSPH3	-0.72	6.73682E-07	4.18557E-06
ENSG00000225470	JPX	1.20	6.77742E-07	4.20949E-06
ENSG00000182325	FBXL6	-0.55	6.80441E-07	4.22411E-06
ENSG00000139324	TMTC3	0.56	6.80518E-07	4.22411E-06
ENSG00000278784	AL136295.7	-1.82	6.80746E-07	4.22422E-06
ENSG00000158169	FANCC	-0.57	6.88265E-07	4.26955E-06
ENSG00000168522	FNTA	0.59	6.89873E-07	4.2782E-06
ENSG00000005238	FAM214B	-0.81	6.94716E-07	4.30689E-06
ENSG00000144645	OSBPL10	0.61	6.95517E-07	4.31053E-06
ENSG00000138375	SMARCAL1	-0.80	7.01618E-07	4.34699E-06
ENSG00000067167	TRAM1	0.35	7.0444E-07	4.36312E-06
ENSG00000101849	TBL1X	-0.44	7.0575E-07	4.36989E-06
ENSG00000175745	NR2F1	0.48	7.07995E-07	4.38243E-06
ENSG00000121753	ADGRB2	0.86	7.11295E-07	4.40149E-06
ENSG00000112149	CD83	0.78	7.2731E-07	4.4992E-06
ENSG00000171530	TBCA	0.35	7.31589E-07	4.52428E-06
ENSG00000197798	FAM118B	0.73	7.33061E-07	4.53198E-06
ENSG00000260027	HOXB7	-0.54	7.34309E-07	4.53829E-06
ENSG00000069869	NEDD4	0.67	7.35494E-07	4.54421E-06
ENSG00000275832	ARHGAP23	-0.51	7.37224E-07	4.55323E-06
ENSG00000139718	SETD1B	-0.59	7.37409E-07	4.55323E-06
ENSG00000196591	HDAC2	0.35	7.3864E-07	4.55943E-06
ENSG00000214160	ALG3	-0.70	7.39086E-07	4.56078E-06
ENSG00000165868	HSPA12A	-0.62	7.41523E-07	4.5744E-06
ENSG00000168214	RBPJ	0.50	7.47831E-07	4.61189E-06
ENSG00000119820	YIPF4	0.47	7.51758E-07	4.63469E-06
ENSG00000170296	GABARAP	0.44	7.52902E-07	4.64031E-06
ENSG00000071242	RPS6KA2	-0.91	7.54096E-07	4.64623E-06
ENSG00000161813	LARP4	0.46	7.5684E-07	4.66171E-06
ENSG00000187735	TCEA1	-0.36	7.57871E-07	4.66662E-06
ENSG00000023318	ERP44	0.57	7.63925E-07	4.70245E-06
ENSG00000116741	RGS2	0.76	7.66011E-07	4.71384E-06
ENSG00000117724	CENPF	0.35	7.67009E-07	4.71853E-06
ENSG00000100804	PSMB5	0.37	7.68275E-07	4.72487E-06
ENSG00000140859	KIFC3	0.54	7.75697E-07	4.76905E-06
ENSG00000132394	EEFSEC	-0.80	7.81538E-07	4.80348E-06
ENSG00000115524	SF3B1	0.37	7.86871E-07	4.83477E-06
ENSG00000010244	ZNF207	0.33	7.91693E-07	4.86291E-06
ENSG00000165632	TAF3	0.63	7.94204E-07	4.87684E-06
ENSG00000154328	NEIL2	0.49	8.02683E-07	4.92739E-06
ENSG00000107140	TESK1	-0.77	8.06752E-07	4.95008E-06
ENSG00000159210	SNF8	0.34	8.06874E-07	4.95008E-06
ENSG00000057252	SOAT1	-0.50	8.0874E-07	4.96001E-06
ENSG00000119682	AREL1	-0.42	8.15481E-07	4.99982E-06
ENSG00000172534	HCFC1	0.40	8.21111E-07	5.03279E-06

ENSG00000188996	HUS1B	4.34	8.23875E-07	5.04819E-06
ENSG00000159459	UBR1	0.66	8.26682E-07	5.06384E-06
ENSG00000173846	PLK3	0.46	8.27797E-07	5.06911E-06
ENSG00000228727	SAPCD1	-1.74	8.30115E-07	5.08096E-06
ENSG00000132670	PTPRA	-0.45	8.30239E-07	5.08096E-06
ENSG00000123472	ATPAF1	-0.51	8.32344E-07	5.09229E-06
ENSG00000235162	C12orf75	0.41	8.42625E-07	5.15361E-06
ENSG00000051596	THOC3	-0.59	8.59021E-07	5.25228E-06
ENSG00000081059	TCF7	-0.64	8.61096E-07	5.26336E-06
ENSG00000154832	CXXC1	-0.48	8.61621E-07	5.26496E-06
ENSG00000163491	NEK10	2.76	8.69892E-07	5.31389E-06
ENSG00000204220	PFDN6	0.44	8.79276E-07	5.36957E-06
ENSG00000155363	MOV10	-0.42	8.80502E-07	5.37541E-06
ENSG00000144120	TMEM177	-0.78	8.89525E-07	5.42884E-06
ENSG00000124535	WRNIP1	-0.38	8.91088E-07	5.43672E-06
ENSG00000091157	WDR7	0.79	8.99866E-07	5.48861E-06
ENSG00000227354	RBM26-AS1	-1.48	9.0033E-07	5.48977E-06
ENSG00000197183	NOL4L	0.89	9.064E-07	5.52509E-06
ENSG00000168439	STIP1	0.29	9.16603E-07	5.58559E-06
ENSG00000102890	ELMO3	-0.65	9.2203E-07	5.61695E-06
ENSG00000167995	BEST1	-1.57	9.27167E-07	5.64652E-06
ENSG00000174720	LARP7	0.52	9.31475E-07	5.67103E-06
ENSG00000174231	PRPF8	-0.28	9.32018E-07	5.67261E-06
ENSG00000119004	CYP20A1	-1.07	9.32577E-07	5.67429E-06
ENSG00000166224	SGPL1	0.33	9.34063E-07	5.6816E-06
ENSG00000251022	THAP9-AS1	-0.48	9.37094E-07	5.69831E-06
ENSG00000166333	ILK	0.68	9.39901E-07	5.71364E-06
ENSG00000086289	EPDR1	0.63	9.45746E-07	5.74743E-06
ENSG00000102030	NAA10	-0.58	9.4721E-07	5.75457E-06
ENSG00000178982	EIF3K	-0.33	9.49573E-07	5.76718E-06
ENSG00000178467	P4HTM	-0.51	9.52272E-07	5.78182E-06
ENSG00000172748	ZNF596	1.28	9.54708E-07	5.79485E-06
ENSG00000100558	PLEK2	0.51	9.61019E-07	5.83138E-06
ENSG00000165699	TSC1	0.52	9.62096E-07	5.83615E-06
ENSG00000090316	MAEA	-0.38	9.64652E-07	5.84988E-06
ENSG00000134882	UBAC2	-0.49	9.6668E-07	5.86041E-06
ENSG00000196154	S100A4	-0.44	9.67916E-07	5.86612E-06
ENSG00000115806	GORASP2	-0.38	9.81842E-07	5.94872E-06
ENSG00000232838	PET117	-1.02	9.85265E-07	5.96765E-06
ENSG00000087191	PSMC5	0.32	9.86093E-07	5.96948E-06
ENSG00000116406	EDEM3	0.73	9.86163E-07	5.96948E-06
ENSG00000164402	SEPTIN8	-0.46	9.92973E-07	6.00888E-06
ENSG00000153560	UBP1	-0.38	9.96889E-07	6.03075E-06
ENSG00000165731	RET	1.09	1.01388E-06	6.13168E-06
ENSG00000120690	ELF1	0.61	1.01881E-06	6.15857E-06
ENSG00000129455	KLK8	-1.24	1.01894E-06	6.15857E-06

ENSG00000109674	NEIL3	-0.87	1.02667E-06	6.20341E-06
ENSG00000175334	BANF1	0.40	1.03272E-06	6.23809E-06
ENSG00000154813	DPH3	0.68	1.03319E-06	6.23903E-06
ENSG00000124120	TTPAL	-0.55	1.04124E-06	6.28578E-06
ENSG00000278619	MRM1	-1.23	1.04518E-06	6.30764E-06
ENSG00000285128	AC092306.1	-2.17	1.04588E-06	6.30996E-06
ENSG00000004399	PLXND1	-0.56	1.04889E-06	6.32619E-06
ENSG00000115657	ABCB6	0.82	1.04963E-06	6.32875E-06
ENSG00000183778	B3GALT5	-0.80	1.0591E-06	6.38395E-06
ENSG00000136950	ARPC5L	0.41	1.06048E-06	6.39033E-06
ENSG00000198131	ZNF544	0.70	1.06606E-06	6.42202E-06
ENSG00000170191	NANP	-0.84	1.06668E-06	6.42385E-06
ENSG00000141934	PLPP2	0.43	1.06757E-06	6.42726E-06
ENSG00000111737	RAB35	0.38	1.0828E-06	6.51698E-06
ENSG00000253313	C1orf210	-1.76	1.08312E-06	6.51698E-06
ENSG00000127463	EMC1	0.33	1.08447E-06	6.52315E-06
ENSG00000075914	EXOSC7	-0.50	1.09511E-06	6.58488E-06
ENSG00000240024	LINC00888	0.99	1.0954E-06	6.58488E-06
ENSG00000100852	ARHGAP5	0.81	1.09655E-06	6.58987E-06
ENSG00000258102	MAP1LC3B2	0.90	1.10061E-06	6.61227E-06
ENSG00000162976	SLC66A3	-0.77	1.1087E-06	6.65888E-06
ENSG00000074582	BCS1L	-0.50	1.11716E-06	6.70764E-06
ENSG00000146828	SLC12A9	-0.64	1.11772E-06	6.70903E-06
ENSG00000014641	MDH1	0.31	1.12675E-06	6.76118E-06
ENSG00000143061	IGSF3	0.43	1.12909E-06	6.7732E-06
ENSG00000136002	ARHGEF4	-0.88	1.13139E-06	6.78346E-06
ENSG00000198898	CAPZA2	0.45	1.13148E-06	6.78346E-06
ENSG00000261663	AC009065.8	1.43	1.13231E-06	6.78638E-06
ENSG00000089063	TMEM230	-0.40	1.13905E-06	6.82476E-06
ENSG00000257084	MIR200CHG	1.55	1.14079E-06	6.83315E-06
ENSG00000116717	GADD45A	-0.44	1.15164E-06	6.89605E-06
ENSG00000109189	USP46	0.52	1.15283E-06	6.90114E-06
ENSG00000136854	STXBP1	-0.53	1.1713E-06	7.00961E-06
ENSG00000198838	RYR3	-2.76	1.17562E-06	7.03332E-06
ENSG00000099617	EFNA2	-1.15	1.18133E-06	7.06538E-06
ENSG00000050393	MCUR1	-0.56	1.18514E-06	7.08608E-06
ENSG00000196739	COL27A1	-0.95	1.19633E-06	7.15085E-06
ENSG00000198015	MRPL42	-0.44	1.19964E-06	7.16848E-06
ENSG00000159082	SYNJ1	0.92	1.20477E-06	7.19697E-06
ENSG00000249115	HAUS5	-0.53	1.20879E-06	7.21883E-06
ENSG00000258768	AL356019.2	-2.65	1.22117E-06	7.29061E-06
ENSG00000167685	ZNF444	0.64	1.22257E-06	7.29675E-06
ENSG00000184185	KCNJ12	0.81	1.22549E-06	7.31201E-06
ENSG00000244879	GABPB1-AS1	-0.54	1.22997E-06	7.33657E-06
ENSG00000156599	ZDHHC5	-0.35	1.25681E-06	7.49442E-06
ENSG00000161618	ALDH16A1	-0.63	1.26141E-06	7.51962E-06

ENSG00000122512	PMS2	-0.73	1.26576E-06	7.54331E-06
ENSG00000169220	RGS14	0.91	1.26883E-06	7.55936E-06
ENSG00000134108	ARL8B	0.40	1.27047E-06	7.56684E-06
ENSG00000198382	UVRAG	-1.02	1.28785E-06	7.66807E-06
ENSG00000157916	RER1	-0.35	1.29123E-06	7.68592E-06
ENSG00000226287	TMEM191A	1.35	1.29503E-06	7.70623E-06
ENSG00000075303	SLC25A40	0.55	1.29847E-06	7.72444E-06
ENSG00000047315	POLR2B	0.37	1.30232E-06	7.74505E-06
ENSG00000105821	DNAJC2	0.50	1.31275E-06	7.80475E-06
ENSG00000172159	FRMD3	2.78	1.31723E-06	7.82903E-06
ENSG00000167130	DOLPP1	-0.63	1.33188E-06	7.91378E-06
ENSG00000159753	CARMIL2	-0.85	1.33353E-06	7.92121E-06
ENSG00000153832	FBXO36	-1.97	1.33842E-06	7.94793E-06
ENSG00000105722	ERF	-0.41	1.34135E-06	7.96293E-06
ENSG00000106477	CEP41	-0.82	1.34178E-06	7.96313E-06
ENSG00000100865	CINP	0.57	1.34589E-06	7.98514E-06
ENSG00000273038	AL365203.2	1.08	1.34731E-06	7.99121E-06
ENSG00000246089	AC016065.1	-1.25	1.37298E-06	8.14103E-06
ENSG00000152253	SPC25	0.54	1.37788E-06	8.1677E-06
ENSG00000176105	YES1	0.41	1.37945E-06	8.17455E-06
ENSG00000185379	RAD51D	-0.73	1.38457E-06	8.2025E-06
ENSG00000100138	SNU13	-0.41	1.38559E-06	8.20611E-06
ENSG00000162222	TTC9C	0.60	1.38768E-06	8.21607E-06
ENSG00000140961	OSGIN1	0.53	1.40897E-06	8.33963E-06
ENSG00000185324	CDK10	-0.48	1.41247E-06	8.3579E-06
ENSG00000070759	TESK2	1.09	1.41709E-06	8.38273E-06
ENSG00000162889	MAPKAPK2	-0.45	1.44003E-06	8.51592E-06
ENSG00000179104	TMTC2	-0.86	1.44164E-06	8.52291E-06
ENSG00000167526	RPL13	-0.37	1.44919E-06	8.565E-06
ENSG00000130023	ERMARD	-0.75	1.45339E-06	8.58729E-06
ENSG00000165156	ZHX1	0.73	1.45723E-06	8.60749E-06
ENSG00000196557	CACNA1H	-0.61	1.46388E-06	8.64419E-06
ENSG00000144134	RABL2A	-1.26	1.47223E-06	8.69096E-06
ENSG00000077147	TM9SF3	0.34	1.47603E-06	8.71081E-06
ENSG00000130764	LRRC47	-0.41	1.49376E-06	8.81283E-06
ENSG00000177606	JUN	0.53	1.49618E-06	8.82449E-06
ENSG00000164603	BMT2	0.91	1.51454E-06	8.93018E-06
ENSG00000162591	MEGF6	-0.74	1.52031E-06	8.96154E-06
ENSG00000112308	C6orf62	0.39	1.52513E-06	8.9873E-06
ENSG00000186792	HYAL3	-1.41	1.52826E-06	9.00308E-06
ENSG00000164715	LMTK2	0.47	1.53023E-06	9.01204E-06
ENSG00000115687	PASK	-0.57	1.53281E-06	9.02458E-06
ENSG00000188419	CHM	0.83	1.53767E-06	9.05056E-06
ENSG00000116151	MORN1	-1.15	1.53905E-06	9.05601E-06
ENSG00000182963	GJC1	-0.40	1.55163E-06	9.12736E-06
ENSG00000085832	EPS15	0.50	1.55501E-06	9.14455E-06

ENSG00000180773	SLC36A4	0.65	1.55962E-06	9.16894E-06
ENSG00000145882	PCYOX1L	-0.59	1.56277E-06	9.18476E-06
ENSG00000246705	H2AJ	0.66	1.56954E-06	9.22181E-06
ENSG00000159348	CYB5R1	0.56	1.56999E-06	9.22181E-06
ENSG00000196810	CTBP1-DT	-0.89	1.57256E-06	9.23419E-06
ENSG00000189171	S100A13	0.48	1.57408E-06	9.24039E-06
ENSG00000119929	CUTC	-0.51	1.57763E-06	9.25854E-06
ENSG00000149541	B3GAT3	0.49	1.57898E-06	9.26372E-06
ENSG00000080822	CLDND1	-0.33	1.58194E-06	9.27837E-06
ENSG00000152818	UTRN	0.64	1.5893E-06	9.31881E-06
ENSG00000186468	RPS23	-0.30	1.59372E-06	9.34196E-06
ENSG00000141252	VPS53	-0.57	1.6108E-06	9.43932E-06
ENSG00000136003	ISCU	0.40	1.62074E-06	9.49479E-06
ENSG00000102312	PORCN	-1.32	1.62534E-06	9.51899E-06
ENSG00000205531	NAP1L4	0.33	1.62845E-06	9.53437E-06
ENSG00000138303	ASCC1	0.55	1.63006E-06	9.541E-06
ENSG00000083444	PLOD1	0.33	1.63653E-06	9.5761E-06
ENSG00000164951	PDP1	0.37	1.65378E-06	9.67418E-06
ENSG00000143256	PFDN2	0.33	1.66756E-06	9.75196E-06
ENSG00000154240	CEP112	0.99	1.67692E-06	9.80385E-06
ENSG00000205413	SAMD9	1.44	1.68301E-06	9.83655E-06
ENSG00000143624	INTS3	-0.48	1.68767E-06	9.8609E-06
ENSG00000240849	TMEM189	-0.56	1.69106E-06	9.87785E-06
ENSG00000179115	FARSA	-0.42	1.74001E-06	1.01608E-05
ENSG00000002016	RAD52	-1.00	1.74868E-06	1.02084E-05
ENSG00000136875	PRPF4	-0.47	1.75013E-06	1.0214E-05
ENSG00000010310	GIPR	0.81	1.77342E-06	1.03469E-05
ENSG00000198938	MT-CO3	-0.49	1.77565E-06	1.03568E-05
ENSG00000135940	COX5B	-0.37	1.77918E-06	1.03744E-05
ENSG00000159579	RSPRY1	0.70	1.79245E-06	1.04487E-05
ENSG00000076248	UNG	-0.39	1.80206E-06	1.05017E-05
ENSG00000212907	MT-ND4L	-0.29	1.81507E-06	1.05744E-05
ENSG00000144659	SLC25A38	-0.53	1.83182E-06	1.06689E-05
ENSG00000258168	AC025569.1	-0.98	1.83465E-06	1.06823E-05
ENSG00000198056	PRIM1	-0.60	1.84433E-06	1.07355E-05
ENSG00000130770	ATP5IF1	0.35	1.84744E-06	1.07505E-05
ENSG00000163162	RNF149	-0.54	1.85145E-06	1.07707E-05
ENSG00000135622	SEMA4F	0.62	1.88671E-06	1.09726E-05
ENSG00000116954	RRAGC	0.70	1.89047E-06	1.09913E-05
ENSG00000155744	FAM126B	0.75	1.90396E-06	1.10665E-05
ENSG00000147874	HAUS6	-0.51	1.9122E-06	1.11112E-05
ENSG00000110108	TMEM109	0.40	1.93421E-06	1.12358E-05
ENSG00000197943	PLCG2	-0.60	1.96708E-06	1.14235E-05
ENSG00000142192	APP	0.27	1.97017E-06	1.1438E-05
ENSG00000172663	TMEM134	-0.85	1.97346E-06	1.14539E-05
ENSG00000011132	APBA3	-0.72	1.97617E-06	1.14663E-05

ENSG00000047849	MAP4	-0.29	1.97933E-06	1.14813E-05
ENSG00000122591	FAM126A	0.61	2.00822E-06	1.16455E-05
ENSG00000185883	ATP6V0C	-0.61	2.00937E-06	1.16488E-05
ENSG00000140386	SCAPER	1.04	2.01069E-06	1.1653E-05
ENSG00000132581	SDF2	0.53	2.01404E-06	1.16691E-05
ENSG00000153147	SMARCA5	0.34	2.01493E-06	1.16708E-05
ENSG00000012124	CD22	-1.40	2.03808E-06	1.18015E-05
ENSG00000162409	PRKAA2	0.62	2.04368E-06	1.18305E-05
ENSG00000076043	REXO2	0.41	2.05053E-06	1.18667E-05
ENSG00000130347	RTN4IP1	0.79	2.08093E-06	1.20392E-05
ENSG00000172530	BANP	0.63	2.08365E-06	1.20515E-05
ENSG00000136738	STAM	0.51	2.11927E-06	1.22539E-05
ENSG00000175482	POLD4	0.71	2.13713E-06	1.23536E-05
ENSG00000074054	CLASP1	0.45	2.1422E-06	1.23794E-05
ENSG00000158290	CUL4B	0.43	2.15488E-06	1.2449E-05
ENSG00000119314	PTBP3	-0.41	2.15934E-06	1.24702E-05
ENSG00000024526	DEPDC1	0.40	2.15978E-06	1.24702E-05
ENSG00000219201	AC138392.1	0.85	2.19888E-06	1.26922E-05
ENSG00000065600	PACC1	0.59	2.20868E-06	1.27451E-05
ENSG00000168795	ZBTB5	0.45	2.21198E-06	1.27605E-05
ENSG00000116752	BCAS2	0.53	2.24225E-06	1.29314E-05
ENSG00000153113	CAST	0.33	2.27355E-06	1.31081E-05
ENSG00000167792	NDUFV1	-0.33	2.27592E-06	1.31171E-05
ENSG00000167315	ACAA2	0.66	2.27642E-06	1.31171E-05
ENSG00000151364	KCTD14	-0.82	2.28324E-06	1.31526E-05
ENSG00000101384	JAG1	0.44	2.30485E-06	1.32733E-05
ENSG00000197136	PCNX3	-0.42	2.35127E-06	1.35367E-05
ENSG00000159873	CCDC117	0.48	2.35747E-06	1.35685E-05
ENSG00000108469	RECQL5	-0.49	2.37213E-06	1.3649E-05
ENSG00000149499	EML3	-0.44	2.3769E-06	1.36724E-05
ENSG00000141543	EIF4A3	0.30	2.37919E-06	1.36817E-05
ENSG00000101311	FERMT1	-0.53	2.38725E-06	1.37241E-05
ENSG00000198276	UCKL1	-0.52	2.38911E-06	1.37309E-05
ENSG00000097096	SYDE2	1.36	2.39432E-06	1.37568E-05
ENSG00000214114	MYCBP	-0.56	2.40076E-06	1.37899E-05
ENSG00000131089	ARHGEF9	-0.77	2.40647E-06	1.38187E-05
ENSG00000128524	ATP6V1F	-0.38	2.42238E-06	1.39061E-05
ENSG00000198373	WWP2	-0.55	2.42395E-06	1.39111E-05
ENSG00000177951	BET1L	-0.48	2.43459E-06	1.39682E-05
ENSG00000137075	RNF38	0.53	2.4536E-06	1.40732E-05
ENSG00000278540	ACACA	-0.29	2.47835E-06	1.42111E-05
ENSG00000148483	TMEM236	3.19	2.48688E-06	1.42559E-05
ENSG00000198356	GET3	-0.41	2.53383E-06	1.45177E-05
ENSG00000175265	GOLGA8A	-0.72	2.53399E-06	1.45177E-05
ENSG00000015532	XYLT2	-0.49	2.53984E-06	1.4547E-05
ENSG00000104472	CHRAC1	-0.43	2.55392E-06	1.46235E-05

ENSG00000011304	PTBP1	-0.29	2.55915E-06	1.46492E-05
ENSG00000040933	INPP4A	-0.69	2.56939E-06	1.47037E-05
ENSG00000073464	CLCN4	0.92	2.59194E-06	1.48284E-05
ENSG00000165678	GHITM	0.29	2.6158E-06	1.49607E-05
ENSG00000183161	FANCF	-1.07	2.61993E-06	1.498E-05
ENSG00000267673	FDX2	1.16	2.64235E-06	1.51039E-05
ENSG00000115468	EFHD1	2.00	2.65638E-06	1.51797E-05
ENSG00000137817	PARP6	-0.52	2.66273E-06	1.52117E-05
ENSG00000164258	NDUFS4	0.49	2.68316E-06	1.5324E-05
ENSG00000105856	HBP1	0.76	2.72707E-06	1.55703E-05
ENSG00000251136	AF117829.1	0.75	2.73679E-06	1.56214E-05
ENSG00000138796	HADH	-0.55	2.74499E-06	1.56638E-05
ENSG00000117262	GPR89A	1.03	2.76192E-06	1.57559E-05
ENSG00000163975	MELTF	-0.47	2.76719E-06	1.57814E-05
ENSG00000159596	TMEM69	-0.53	2.78309E-06	1.58676E-05
ENSG00000109089	CDR2L	0.52	2.82348E-06	1.60933E-05
ENSG00000212123	PRR22	-1.72	2.83069E-06	1.61298E-05
ENSG00000114331	ACAP2	0.46	2.83594E-06	1.61534E-05
ENSG00000105974	CAV1	-0.30	2.83644E-06	1.61534E-05
ENSG00000155034	FBXL18	0.63	2.84092E-06	1.61743E-05
ENSG00000125970	RALY	0.37	2.84884E-06	1.62147E-05
ENSG00000286058	AC018865.2	-2.04	2.85024E-06	1.62181E-05
ENSG00000185947	ZNF267	0.81	2.85171E-06	1.62218E-05
ENSG00000087365	SF3B2	0.29	2.88097E-06	1.63836E-05
ENSG00000112208	BAG2	0.49	2.88876E-06	1.64232E-05
ENSG00000144635	DYNC1LI1	0.46	2.92285E-06	1.66123E-05
ENSG00000079819	EPB41L2	-0.35	2.92946E-06	1.66452E-05
ENSG00000184454	NCMAP	1.14	2.93068E-06	1.66474E-05
ENSG00000170906	NDUFA3	0.54	2.94327E-06	1.67141E-05
ENSG00000120742	SERP1	-0.37	2.95499E-06	1.6776E-05
ENSG00000111700	SLCO1B3	1.69	2.95628E-06	1.67785E-05
ENSG00000139546	TARBP2	0.59	2.96731E-06	1.68363E-05
ENSG00000090539	CHRD	0.74	2.97303E-06	1.6864E-05
ENSG00000135018	UBQLN1	0.30	2.97504E-06	1.68706E-05
ENSG00000149177	PTPRJ	0.42	2.97648E-06	1.6874E-05
ENSG00000120158	RCL1	-0.63	2.98482E-06	1.69165E-05
ENSG00000198792	TMEM184B	-0.38	2.9906E-06	1.69445E-05
ENSG00000134058	CDK7	-0.55	2.99182E-06	1.69466E-05
ENSG00000143570	SLC39A1	-1.07	2.99674E-06	1.69696E-05
ENSG00000095303	PTGS1	0.98	3.01234E-06	1.70532E-05
ENSG00000140650	PMM2	-0.58	3.01352E-06	1.7055E-05
ENSG00000127452	FBXL12	0.70	3.01911E-06	1.70818E-05
ENSG00000120705	ETF1	-0.30	3.06623E-06	1.73435E-05
ENSG00000132823	OSER1	0.54	3.06846E-06	1.73513E-05
ENSG00000271936	AC012073.1	-1.69	3.09291E-06	1.74846E-05
ENSG00000225032	AL162586.1	-3.07	3.11366E-06	1.75969E-05

ENSG00000076924	XAB2	0.32	3.11636E-06	1.76036E-05
ENSG00000150433	TMEM218	-0.71	3.11661E-06	1.76036E-05
ENSG00000179918	SEPHS2	-0.42	3.1268E-06	1.76562E-05
ENSG00000121671	CRY2	0.70	3.1352E-06	1.76986E-05
ENSG00000138185	ENTPD1	-1.32	3.14703E-06	1.77604E-05
ENSG00000160679	CHTOP	-0.34	3.16116E-06	1.7833E-05
ENSG00000130749	ZC3H4	0.48	3.16168E-06	1.7833E-05
ENSG00000126767	ELK1	0.48	3.16682E-06	1.7857E-05
ENSG00000185686	PRAME	0.50	3.17985E-06	1.79254E-05
ENSG00000129255	MPDU1	0.43	3.20468E-06	1.80603E-05
ENSG00000164237	CMBL	0.51	3.24532E-06	1.82842E-05
ENSG00000185875	THNSL1	-1.06	3.32033E-06	1.87015E-05
ENSG00000134982	APC	0.62	3.34213E-06	1.8819E-05
ENSG00000131242	RAB11FIP4	-0.70	3.42514E-06	1.9281E-05
ENSG00000132781	MUTYH	-1.06	3.44638E-06	1.93951E-05
ENSG00000164944	VIRMA	0.41	3.46388E-06	1.94881E-05
ENSG00000178458	H3P16	0.85	3.47242E-06	1.95307E-05
ENSG00000165804	ZNF219	0.70	3.48016E-06	1.95687E-05
ENSG00000251138	LINC02882	1.24	3.48395E-06	1.95845E-05
ENSG00000163660	CCNL1	-0.41	3.48683E-06	1.95952E-05
ENSG00000228509	AC006460.1	2.40	3.48916E-06	1.96028E-05
ENSG00000170185	USP38	0.55	3.49894E-06	1.96522E-05
ENSG00000161395	PGAP3	-0.99	3.51114E-06	1.97152E-05
ENSG00000243449	C4orf48	0.70	3.51322E-06	1.97213E-05
ENSG00000164087	POC1A	0.47	3.52328E-06	1.97723E-05
ENSG00000076003	MCM6	-0.32	3.53142E-06	1.98124E-05
ENSG00000100934	SEC23A	0.38	3.53598E-06	1.98324E-05
ENSG00000162600	OMA1	-0.83	3.54278E-06	1.9865E-05
ENSG00000116497	S100PBP	-0.59	3.58179E-06	2.00781E-05
ENSG00000104853	CLPTM1	0.33	3.59531E-06	2.01482E-05
ENSG00000112941	TENT4A	-0.46	3.59761E-06	2.01555E-05
ENSG00000163866	SMIM12	0.53	3.59984E-06	2.01623E-05
ENSG00000257337	AC068888.1	-1.18	3.60485E-06	2.01847E-05
ENSG00000148950	IMMP1L	0.64	3.61001E-06	2.0208E-05
ENSG00000165406	MARCHF8	-0.63	3.63255E-06	2.03285E-05
ENSG00000180884	ZNF792	1.54	3.63779E-06	2.03521E-05
ENSG00000066044	ELAVL1	-0.32	3.63911E-06	2.03538E-05
ENSG00000160131	VMA21	-0.41	3.64143E-06	2.03611E-05
ENSG00000100307	CBX7	-0.66	3.64316E-06	2.03651E-05
ENSG00000175592	FOSL1	0.37	3.6642E-06	2.0477E-05
ENSG00000125741	OPA3	-0.60	3.66842E-06	2.04948E-05
ENSG00000138363	ATIC	-0.37	3.67375E-06	2.05189E-05
ENSG00000109458	GAB1	1.22	3.68034E-06	2.055E-05
ENSG00000197448	GSTK1	-0.46	3.68642E-06	2.05781E-05
ENSG00000204580	DDR1	-0.31	3.71762E-06	2.07465E-05
ENSG00000197223	C1D	0.72	3.72601E-06	2.07875E-05

ENSG00000159556	ISL2	0.74	3.74472E-06	2.08861E-05
ENSG00000167797	CDK2AP2	0.44	3.75312E-06	2.09271E-05
ENSG00000088538	DOCK3	0.64	3.75575E-06	2.0936E-05
ENSG00000249328	AC036214.1	2.60	3.76175E-06	2.09631E-05
ENSG00000132613	MTSS2	-0.71	3.76271E-06	2.09631E-05
ENSG00000100142	POLR2F	0.43	3.76915E-06	2.09931E-05
ENSG00000080546	SESN1	0.84	3.77525E-06	2.10212E-05
ENSG00000145743	FBXL17	0.77	3.79668E-06	2.11347E-05
ENSG00000177873	ZNF619	-1.34	3.80214E-06	2.11592E-05
ENSG00000163348	PYGO2	-0.52	3.82551E-06	2.12781E-05
ENSG00000280385	AP000648.4	-1.19	3.82614E-06	2.12781E-05
ENSG00000159202	UBE2Z	0.32	3.82669E-06	2.12781E-05
ENSG00000119899	SLC17A5	0.45	3.83163E-06	2.12996E-05
ENSG00000184574	LPAR5	1.13	3.8346E-06	2.13102E-05
ENSG00000184489	PTP4A3	-0.75	3.84306E-06	2.13513E-05
ENSG00000155380	SLC16A1	-0.42	3.87558E-06	2.1526E-05
ENSG00000183735	TBK1	0.50	3.88986E-06	2.15993E-05
ENSG00000179021	C3orf38	0.76	3.92197E-06	2.17716E-05
ENSG00000146587	RBAK	0.60	3.97951E-06	2.20849E-05
ENSG00000125779	PANK2	0.47	3.99375E-06	2.21577E-05
ENSG00000130487	KLHDC7B	-7.28	4.00067E-06	2.219E-05
ENSG00000253958	CLDN23	1.15	4.02019E-06	2.22921E-05
ENSG00000097007	ABL1	-0.42	4.04852E-06	2.24429E-05
ENSG00000168005	SPINDOC	0.40	4.05952E-06	2.24977E-05
ENSG00000137413	TAF8	0.51	4.0654E-06	2.2524E-05
ENSG00000124226	RNF114	0.36	4.07463E-06	2.25689E-05
ENSG00000181588	MEX3D	0.42	4.08246E-06	2.2606E-05
ENSG00000130052	STARD8	0.81	4.0959E-06	2.26707E-05
ENSG00000169247	SH3TC2	-0.77	4.0964E-06	2.26707E-05
ENSG00000118200	CAMSAP2	0.47	4.10528E-06	2.27136E-05
ENSG00000198113	TOR4A	0.44	4.12267E-06	2.28034E-05
ENSG00000154102	C16orf74	-0.87	4.21705E-06	2.3319E-05
ENSG00000139641	ESYT1	0.31	4.22063E-06	2.33324E-05
ENSG00000165512	ZNF22	-0.90	4.22432E-06	2.33463E-05
ENSG00000111186	WNT5B	2.52	4.24168E-06	2.34358E-05
ENSG00000169490	TM2D2	0.46	4.26149E-06	2.35387E-05
ENSG00000112561	TFEB	1.16	4.28222E-06	2.36467E-05
ENSG00000101596	SMCHD1	-0.47	4.28936E-06	2.36796E-05
ENSG00000112365	ZBTB24	-0.82	4.29846E-06	2.37233E-05
ENSG00000183111	ARHGEF37	-0.91	4.3137E-06	2.37957E-05
ENSG00000166881	NEMP1	0.46	4.31395E-06	2.37957E-05
ENSG00000156110	ADK	0.53	4.3256E-06	2.38534E-05
ENSG00000145623	OSMR	0.45	4.34619E-06	2.39603E-05
ENSG00000162413	KLHL21	-0.44	4.38175E-06	2.41497E-05
ENSG00000046604	DSG2	0.36	4.46157E-06	2.45829E-05
ENSG00000138835	RGS3	-0.50	4.48235E-06	2.46905E-05

ENSG00000040608	RTN4R	0.67	4.4895E-06	2.47232E-05
ENSG00000141551	CSNK1D	-0.29	4.51561E-06	2.48565E-05
ENSG00000185973	TMLHE	1.04	4.51619E-06	2.48565E-05
ENSG00000148985	PGAP2	-0.59	4.57413E-06	2.51684E-05
ENSG00000166136	NDUFB8	0.42	4.6225E-06	2.54276E-05
ENSG00000103496	STX4	0.65	4.64723E-06	2.55566E-05
ENSG00000153094	BCL2L11	0.61	4.65636E-06	2.55997E-05
ENSG00000259956	RBM15B	-0.40	4.66561E-06	2.56436E-05
ENSG00000239665	AL157392.3	-1.31	4.66944E-06	2.56576E-05
ENSG00000266173	STRADA	-0.80	4.69506E-06	2.57912E-05
ENSG00000136143	SUCLA2	0.50	4.7492E-06	2.60815E-05
ENSG00000149573	MPZL2	0.82	4.80286E-06	2.6369E-05
ENSG00000180822	PSMG4	-0.88	4.81527E-06	2.64298E-05
ENSG00000064703	DDX20	0.61	4.90083E-06	2.68921E-05
ENSG00000167113	COQ4	-0.53	4.93867E-06	2.70923E-05
ENSG00000125691	RPL23	-0.27	4.97821E-06	2.73017E-05
ENSG00000174132	FAM174A	0.80	5.08773E-06	2.78947E-05
ENSG00000173237	C11orf86	3.12	5.09171E-06	2.79088E-05
ENSG00000184575	XPOT	-0.35	5.0969E-06	2.79297E-05
ENSG00000172270	BSG	0.33	5.10496E-06	2.79662E-05
ENSG00000052795	FNIP2	0.86	5.15901E-06	2.82546E-05
ENSG00000198826	ARHGAP11A	0.38	5.18101E-06	2.83673E-05
ENSG00000124224	PPP4R1L	0.88	5.19489E-06	2.84355E-05
ENSG00000154803	FLCN	0.63	5.20888E-06	2.85043E-05
ENSG00000139567	ACVRL1	1.92	5.22384E-06	2.85783E-05
ENSG00000213015	ZNF580	-0.65	5.22689E-06	2.85872E-05
ENSG00000131747	TOP2A	0.34	5.3E-06	2.89791E-05
ENSG00000108094	CUL2	0.53	5.30798E-06	2.90148E-05
ENSG00000120509	PDZD11	0.51	5.31542E-06	2.90476E-05
ENSG00000135845	PIGC	-0.80	5.3273E-06	2.91045E-05
ENSG00000131981	LGALS3	-0.44	5.37092E-06	2.93348E-05
ENSG00000283064	AL353759.1	3.82	5.3817E-06	2.93857E-05
ENSG00000206075	SERPINB5	0.72	5.40202E-06	2.94886E-05
ENSG00000162745	OLFML2B	1.36	5.41642E-06	2.95591E-05
ENSG00000133704	IPO8	0.48	5.42193E-06	2.95811E-05
ENSG00000112624	BICRAL	-0.66	5.4562E-06	2.976E-05
ENSG00000151690	MFSD6	0.59	5.46496E-06	2.97996E-05
ENSG00000260121	AC138028.4	-1.56	5.48149E-06	2.98817E-05
ENSG00000163904	SENP2	0.44	5.48384E-06	2.98863E-05
ENSG00000087448	KLHL42	-0.58	5.49389E-06	2.99329E-05
ENSG00000172531	PPP1CA	-0.33	5.49554E-06	2.99338E-05
ENSG00000197008	ZNF138	0.88	5.50444E-06	2.99741E-05
ENSG00000285872	AC007240.3	3.17	5.50825E-06	2.99867E-05
ENSG00000204876	AC021218.1	1.71	5.51863E-06	3.0035E-05
ENSG00000245164	LINC00861	-4.57	5.52269E-06	3.0049E-05
ENSG00000183527	PSMG1	-0.47	5.57582E-06	3.03298E-05

ENSG00000125089	SH3TC1	0.96	5.58792E-06	3.03873E-05
ENSG00000178988	MRFAP1L1	-0.47	5.60256E-06	3.04587E-05
ENSG00000164597	COG5	0.60	5.6381E-06	3.06436E-05
ENSG00000067560	RHOA	-0.30	5.64651E-06	3.06809E-05
ENSG00000142632	ARHGEF19	-0.43	5.67157E-06	3.08088E-05
ENSG00000170100	ZNF778	-0.56	5.70351E-06	3.09728E-05
ENSG00000124333	VAMP7	0.49	5.70486E-06	3.09728E-05
ENSG00000106635	BCL7B	-0.50	5.73333E-06	3.11189E-05
ENSG00000127423	AUNIP	-0.66	5.7741E-06	3.13317E-05
ENSG00000149212	SESN3	1.39	5.78325E-06	3.13728E-05
ENSG00000100764	PSMC1	0.88	5.84535E-06	3.17011E-05
ENSG00000062038	CDH3	-0.31	5.88145E-06	3.18882E-05
ENSG00000254598	CSNK2A3	0.54	5.90926E-06	3.20304E-05
ENSG00000112893	MAN2A1	-0.52	5.92143E-06	3.20877E-05
ENSG00000058091	CDK14	0.77	5.93935E-06	3.2176E-05
ENSG00000123505	AMD1	-0.43	5.99602E-06	3.24742E-05
ENSG00000205423	CNEP1R1	0.66	6.03958E-06	3.27013E-05
ENSG00000110583	NAA40	-0.59	6.05826E-06	3.27936E-05
ENSG00000124006	OBSL1	0.68	6.08192E-06	3.29128E-05
ENSG00000189091	SF3B3	0.28	6.0976E-06	3.29887E-05
ENSG00000182580	EPHB3	0.92	6.11119E-06	3.30533E-05
ENSG00000115289	PCGF1	0.53	6.1514E-06	3.32618E-05
ENSG00000163156	SCNM1	0.57	6.17081E-06	3.33577E-05
ENSG00000131015	ULBP2	0.65	6.17387E-06	3.33652E-05
ENSG00000165813	CCDC186	0.60	6.20145E-06	3.35052E-05
ENSG00000115947	ORC4	0.57	6.22005E-06	3.35966E-05
ENSG00000099331	MYO9B	-0.38	6.23515E-06	3.36692E-05
ENSG00000100031	GGT1	1.16	6.25498E-06	3.37671E-05
ENSG00000146457	WTAP	-0.39	6.25947E-06	3.37822E-05
ENSG00000117242	PINK1-AS	-0.93	6.26847E-06	3.38217E-05
ENSG00000103253	HAGHL	-0.64	6.32938E-06	3.41411E-05
ENSG00000021762	OSBPL5	-0.53	6.34106E-06	3.41949E-05
ENSG00000264343	NOTCH2NLA	1.67	6.41781E-06	3.45995E-05
ENSG00000267519	AC020916.1	0.82	6.44525E-06	3.4738E-05
ENSG00000149054	ZNF215	-1.05	6.52102E-06	3.51279E-05
ENSG00000171517	LPAR3	-0.69	6.5211E-06	3.51279E-05
ENSG00000123352	SPATS2	0.41	6.5499E-06	3.52736E-05
ENSG00000166526	ZNF3	0.52	6.56747E-06	3.53587E-05
ENSG00000198890	PRMT6	-0.84	6.57147E-06	3.53707E-05
ENSG00000177954	RPS27	-0.30	6.59597E-06	3.5493E-05
ENSG00000261167	AC107027.3	-2.57	6.60686E-06	3.5542E-05
ENSG00000232320	MXRA7P1	-1.53	6.65323E-06	3.57819E-05
ENSG00000261061	AC092718.4	-1.07	6.65936E-06	3.58052E-05
ENSG00000147164	SNX12	0.42	6.67701E-06	3.58905E-05
ENSG00000111445	RFC5	-0.36	6.68685E-06	3.59338E-05
ENSG00000148411	NACC2	-0.39	6.70572E-06	3.60255E-05

ENSG00000247271	ZBED5-AS1	-1.15	6.78349E-06	3.64335E-05
ENSG00000136238	RAC1	-0.29	6.8079E-06	3.65548E-05
ENSG00000140395	WDR61	-0.43	6.81464E-06	3.65812E-05
ENSG00000118046	STK11	-0.36	6.82538E-06	3.6629E-05
ENSG00000142089	IFITM3	0.34	6.85729E-06	3.67904E-05
ENSG00000166902	MRPL16	-0.46	6.866E-06	3.68273E-05
ENSG00000060237	WNK1	-0.38	6.89814E-06	3.69897E-05
ENSG00000162642	C1orf52	0.54	6.9008E-06	3.6994E-05
ENSG00000100350	FOXRED2	-0.71	6.90856E-06	3.70257E-05
ENSG00000137656	BUD13	-0.55	6.91537E-06	3.70523E-05
ENSG00000163884	KLF15	-1.58	6.92989E-06	3.71202E-05
ENSG00000156253	RWDD2B	-0.61	6.98904E-06	3.7427E-05
ENSG00000089876	DHX32	-0.54	6.99749E-06	3.74622E-05
ENSG00000247626	MARS2	-0.62	7.02548E-06	3.75951E-05
ENSG00000100991	TRPC4AP	-0.36	7.02606E-06	3.75951E-05
ENSG00000070404	FSTL3	0.49	7.05305E-06	3.77294E-05
ENSG00000114520	SNX4	0.56	7.07321E-06	3.78271E-05
ENSG00000049618	ARID1B	-0.54	7.11438E-06	3.80371E-05
ENSG00000205593	DENND6B	-1.15	7.13426E-06	3.81332E-05
ENSG00000198586	TLK1	0.47	7.16174E-06	3.82699E-05
ENSG00000177463	NR2C2	-0.43	7.16487E-06	3.82764E-05
ENSG00000121653	MAPK8IP1	0.72	7.24621E-06	3.86935E-05
ENSG00000166337	TAF10	-0.50	7.24682E-06	3.86935E-05
ENSG00000124574	ABCC10	-0.67	7.30839E-06	3.90119E-05
ENSG00000150687	PRSS23	-1.02	7.32125E-06	3.90701E-05
ENSG00000167103	PIP5KL1	-0.61	7.34427E-06	3.91825E-05
ENSG00000048028	USP28	-0.63	7.35717E-06	3.92408E-05
ENSG00000134490	TMEM241	-0.86	7.37449E-06	3.93228E-05
ENSG00000087053	MTMR2	0.47	7.38964E-06	3.9393E-05
ENSG00000164062	APEH	-0.31	7.39869E-06	3.94308E-05
ENSG00000166451	CENPN	0.49	7.45043E-06	3.9696E-05
ENSG00000058063	ATP11B	0.46	7.46923E-06	3.97855E-05
ENSG00000141424	SLC39A6	0.34	7.5019E-06	3.99489E-05
ENSG00000237149	ZNF503-AS2	1.32	7.54687E-06	4.01777E-05
ENSG00000233276	GPX1	-0.31	7.64886E-06	4.07098E-05
ENSG00000260448	LCMT1-AS1	2.34	7.65387E-06	4.07257E-05
ENSG00000198740	ZNF652	0.49	7.66916E-06	4.07923E-05
ENSG00000122550	KLHL7	0.65	7.67047E-06	4.07923E-05
ENSG00000092203	TOX4	0.41	7.67316E-06	4.07958E-05
ENSG00000119446	RBM18	0.50	7.67591E-06	4.07996E-05
ENSG00000107223	EDF1	0.33	7.70143E-06	4.09243E-05
ENSG00000129187	DCTD	0.36	7.70669E-06	4.09414E-05
ENSG00000175182	FAM131A	-0.73	7.72506E-06	4.10281E-05
ENSG00000197714	ZNF460	1.57	7.74015E-06	4.10974E-05
ENSG00000129657	SEC14L1	0.43	7.76099E-06	4.11971E-05
ENSG00000185722	ANKFY1	-0.42	7.76372E-06	4.12006E-05

ENSG00000246263	UBR5-AS1	-4.05	7.77851E-06	4.12682E-05
ENSG00000141232	TOB1	0.41	7.78738E-06	4.13043E-05
ENSG00000103540	CCP110	0.51	7.80234E-06	4.13727E-05
ENSG00000244733	AL132656.2	-2.71	7.81477E-06	4.14276E-05
ENSG00000104408	EIF3E	0.27	7.8364E-06	4.15313E-05
ENSG00000034677	RNF19A	0.58	7.9219E-06	4.19733E-05
ENSG00000197818	SLC9A8	-0.67	8.00847E-06	4.24207E-05
ENSG00000122376	SHLD2	0.52	8.05878E-06	4.26759E-05
ENSG00000105248	YJU2	-0.61	8.0697E-06	4.27224E-05
ENSG00000119927	GPAM	0.47	8.09221E-06	4.28303E-05
ENSG00000137210	TMEM14B	-0.38	8.09598E-06	4.28314E-05
ENSG00000256060	TRAPPC2B	1.09	8.0967E-06	4.28314E-05
ENSG00000114978	MOB1A	-0.36	8.10196E-06	4.28479E-05
ENSG00000170312	CDK1	0.37	8.1283E-06	4.29758E-05
ENSG00000168785	TSPAN5	-0.34	8.13346E-06	4.29918E-05
ENSG00000172081	MOB3A	-0.61	8.17797E-06	4.32156E-05
ENSG00000265763	ZNF488	-1.37	8.20956E-06	4.33711E-05
ENSG00000115616	SLC9A2	1.23	8.26437E-06	4.36484E-05
ENSG00000118412	CASP8AP2	-0.70	8.26642E-06	4.36484E-05
ENSG00000105287	PRKD2	0.42	8.36658E-06	4.41656E-05
ENSG00000198576	ARC	0.61	8.37309E-06	4.41884E-05
ENSG00000204304	PBX2	0.34	8.39328E-06	4.42832E-05
ENSG00000112146	FBXO9	0.43	8.41185E-06	4.43695E-05
ENSG00000171634	BPTF	0.40	8.48379E-06	4.47372E-05
ENSG00000105997	HOXA3	1.53	8.49363E-06	4.47773E-05
ENSG00000270871	AC015849.3	-2.21	8.52291E-06	4.49198E-05
ENSG00000166173	LARP6	0.45	8.62339E-06	4.54374E-05
ENSG00000175203	DCTN2	0.36	8.64807E-06	4.55555E-05
ENSG00000010270	STARD3NL	-0.50	8.65174E-06	4.55628E-05
ENSG00000168310	IRF2	0.88	8.66777E-06	4.56352E-05
ENSG00000122507	BBS9	1.11	8.68364E-06	4.57068E-05
ENSG00000270629	NBPF14	0.83	8.69295E-06	4.57437E-05
ENSG00000173163	COMMD1	0.76	8.71908E-06	4.58691E-05
ENSG00000182919	C11orf54	0.59	8.73307E-06	4.59307E-05
ENSG00000166794	PPIB	0.29	8.75658E-06	4.60422E-05
ENSG00000196569	LAMA2	0.92	8.76188E-06	4.6058E-05
ENSG00000088325	TPX2	-0.29	8.85156E-06	4.65172E-05
ENSG00000176428	VPS37D	0.97	8.89316E-06	4.67235E-05
ENSG00000165795	NDRG2	0.65	8.99625E-06	4.72527E-05
ENSG00000087076	HSD17B14	1.12	9.00786E-06	4.73013E-05
ENSG00000112379	ARFGEF3	0.70	9.08822E-06	4.77108E-05
ENSG00000102780	DGKH	-0.77	9.09399E-06	4.77286E-05
ENSG00000086598	TMED2	0.27	9.10647E-06	4.77815E-05
ENSG00000180089	TMEM86B	-2.33	9.14813E-06	4.79875E-05
ENSG00000064652	SNX24	-0.87	9.15152E-06	4.79927E-05
ENSG00000136436	CALCOCO2	0.36	9.21293E-06	4.83021E-05

ENSG00000003400	CASP10	1.35	9.22371E-06	4.83459E-05
ENSG00000162928	PEX13	0.56	9.22994E-06	4.8366E-05
ENSG00000136451	VEZF1	-0.35	9.2629E-06	4.8526E-05
ENSG00000262879	AC005670.3	0.60	9.2862E-06	4.86353E-05
ENSG00000102858	MGRN1	-0.49	9.29342E-06	4.86604E-05
ENSG00000161653	NAGS	0.65	9.34287E-06	4.89065E-05
ENSG00000135083	CCNJL	0.86	9.38791E-06	4.91294E-05
ENSG00000064115	TM7SF3	0.39	9.40319E-06	4.91965E-05
ENSG00000184840	TMED9	0.33	9.45198E-06	4.94388E-05
ENSG00000198093	ZNF649	-1.37	9.45547E-06	4.94442E-05
ENSG00000196411	EPHB4	0.32	9.48259E-06	4.9573E-05
ENSG00000125746	EML2	-0.41	9.50522E-06	4.96784E-05
ENSG00000158483	FAM86C1P	-0.95	9.55019E-06	4.99004E-05
ENSG00000169129	AFAP1L2	2.08	9.60452E-06	5.01712E-05
ENSG00000112242	E2F3	0.40	9.68367E-06	5.05678E-05
ENSG00000065717	TLE2	0.48	9.6855E-06	5.05678E-05
ENSG00000159592	GPBP1L1	0.36	9.70189E-06	5.06401E-05
ENSG00000259583	AC015712.2	-1.36	9.71864E-06	5.07143E-05
ENSG00000265241	RBM8A	0.30	9.82433E-06	5.12525E-05
ENSG00000136717	BIN1	0.41	9.83211E-06	5.12797E-05
ENSG00000124596	OARD1	-0.59	9.97395E-06	5.20059E-05
ENSG00000155975	VPS37A	0.53	9.98521E-06	5.2051E-05
ENSG00000137601	NEK1	0.65	1.00229E-05	5.22341E-05
ENSG00000118523	CCN2	0.66	1.00557E-05	5.23912E-05
ENSG00000111371	SLC38A1	-0.32	1.01308E-05	5.27685E-05
ENSG00000187713	TMEM203	-0.46	1.01663E-05	5.29397E-05
ENSG00000167114	SLC27A4	0.46	1.02847E-05	5.35424E-05
ENSG00000288558	DUS4L-BCAP2	-2.17	1.03066E-05	5.36427E-05
ENSG00000092010	PSME1	-0.35	1.03171E-05	5.36831E-05
ENSG00000015171	ZMYND11	-0.60	1.0345E-05	5.38144E-05
ENSG00000111328	CDK2AP1	0.33	1.0356E-05	5.38579E-05
ENSG00000084073	ZMPSTE24	0.33	1.03654E-05	5.38928E-05
ENSG00000128298	BAIAP2L2	-1.96	1.03904E-05	5.40083E-05
ENSG00000119718	EIF2B2	-0.48	1.0449E-05	5.4299E-05
ENSG00000106346	USP42	0.55	1.0464E-05	5.43629E-05
ENSG00000259994	AL353796.1	-1.70	1.04695E-05	5.43773E-05
ENSG00000104140	RHOV	0.97	1.05684E-05	5.48766E-05
ENSG00000154229	PRKCA	-0.52	1.06865E-05	5.54754E-05
ENSG00000164105	SAP30	0.77	1.07362E-05	5.57193E-05
ENSG00000224631	RPS27AP16	-0.31	1.07455E-05	5.57528E-05
ENSG00000225968	ELFN1	-1.30	1.09205E-05	5.66464E-05
ENSG00000179965	ZNF771	0.77	1.10764E-05	5.74398E-05
ENSG00000169607	CKAP2L	0.52	1.10792E-05	5.74399E-05
ENSG00000163812	ZDHHC3	-0.37	1.11274E-05	5.76747E-05
ENSG00000134371	CDC73	0.43	1.12116E-05	5.80959E-05
ENSG00000162521	RBBP4	-0.29	1.12172E-05	5.81004E-05

ENSG00000148773	MKI67	0.26	1.12183E-05	5.81004E-05
ENSG00000111674	ENO2	0.84	1.12648E-05	5.83264E-05
ENSG00000175216	CKAP5	0.30	1.12793E-05	5.83755E-05
ENSG00000179598	PLD6	0.63	1.12801E-05	5.83755E-05
ENSG00000130021	PUDP	-0.59	1.12835E-05	5.83778E-05
ENSG00000198728	LDB1	-0.33	1.13341E-05	5.86247E-05
ENSG00000120925	RNF170	-1.04	1.13595E-05	5.87406E-05
ENSG00000233901	LINC01503	-2.44	1.13679E-05	5.87688E-05
ENSG00000221829	FANCG	-0.40	1.15033E-05	5.94538E-05
ENSG00000173153	ESRRA	-0.39	1.1524E-05	5.95452E-05
ENSG00000058272	PPP1R12A	-0.50	1.15572E-05	5.96874E-05
ENSG00000104320	NBN	-0.47	1.15575E-05	5.96874E-05
ENSG00000100030	MAPK1	-0.32	1.16242E-05	6.00161E-05
ENSG00000153531	ADPRHL1	-0.70	1.16923E-05	6.03371E-05
ENSG00000134440	NARS1	-0.29	1.16924E-05	6.03371E-05
ENSG00000080815	PSEN1	0.45	1.17768E-05	6.0757E-05
ENSG00000128510	CPA4	-0.88	1.18042E-05	6.08831E-05
ENSG00000100196	KDELR3	0.50	1.19079E-05	6.14017E-05
ENSG00000168306	ACOX2	0.72	1.19332E-05	6.15165E-05
ENSG00000185621	LMLN	-1.13	1.19759E-05	6.1721E-05
ENSG00000140553	UNC45A	0.31	1.19808E-05	6.17303E-05
ENSG00000011295	TTC19	-0.40	1.20943E-05	6.22986E-05
ENSG00000163626	COX18	-0.88	1.21546E-05	6.25931E-05
ENSG00000196420	S100A5	-2.95	1.21947E-05	6.27837E-05
ENSG00000090432	MUL1	-0.47	1.22022E-05	6.28064E-05
ENSG00000075975	MKRN2	0.46	1.22115E-05	6.28377E-05
ENSG00000175324	LSM1	0.46	1.22695E-05	6.31202E-05
ENSG00000170471	RALGAPB	0.37	1.23061E-05	6.32919E-05
ENSG00000249042	AC008771.1	1.85	1.23684E-05	6.35963E-05
ENSG00000157184	CPT2	-0.58	1.23719E-05	6.35978E-05
ENSG00000036054	TBC1D23	0.72	1.23973E-05	6.37121E-05
ENSG00000083799	CYLD	0.73	1.25159E-05	6.43049E-05
ENSG00000096070	BRPF3	-0.49	1.2542E-05	6.44224E-05
ENSG00000158458	NRG2	1.69	1.26403E-05	6.49107E-05
ENSG00000170456	DENND5B	0.60	1.26774E-05	6.50845E-05
ENSG00000107020	PLGRKT	-0.87	1.27093E-05	6.5232E-05
ENSG00000117475	BLZF1	0.69	1.27271E-05	6.53062E-05
ENSG00000145919	BOD1	0.45	1.27474E-05	6.53938E-05
ENSG00000135148	TRAFD1	0.43	1.27714E-05	6.54998E-05
ENSG00000171208	NETO2	0.71	1.27824E-05	6.55396E-05
ENSG00000088035	ALG6	-0.79	1.28457E-05	6.58476E-05
ENSG00000081087	OSTM1	0.67	1.29127E-05	6.61739E-05
ENSG00000198860	TSEN15	-0.47	1.29506E-05	6.63513E-05
ENSG00000116237	ICMT	-0.30	1.30421E-05	6.68027E-05
ENSG00000175110	MRPS22	0.45	1.30544E-05	6.68489E-05
ENSG00000161533	ACOX1	0.41	1.306E-05	6.68604E-05

ENSG00000215790	SLC35E2A	-1.12	1.30845E-05	6.69687E-05
ENSG00000204392	LSM2	-0.48	1.31117E-05	6.70904E-05
ENSG00000166886	NAB2	0.38	1.31484E-05	6.72612E-05
ENSG00000157992	KRTCAP3	0.55	1.31846E-05	6.7429E-05
ENSG00000155980	KIF5A	0.74	1.3203E-05	6.75062E-05
ENSG00000137055	PLAA	0.43	1.32265E-05	6.7609E-05
ENSG00000007541	PIGQ	-0.81	1.32327E-05	6.76232E-05
ENSG00000160323	ADAMTS13	-1.05	1.32581E-05	6.77312E-05
ENSG00000184432	COPB2	0.32	1.32606E-05	6.77312E-05
ENSG00000104331	BPNT2	-0.35	1.328E-05	6.7813E-05
ENSG00000084093	REST	0.47	1.33141E-05	6.79698E-05
ENSG00000104067	TJP1	0.38	1.34584E-05	6.8689E-05
ENSG00000105887	MTPN	0.30	1.35945E-05	6.9366E-05
ENSG00000166716	ZNF592	-0.44	1.36487E-05	6.96113E-05
ENSG00000144485	HES6	0.50	1.36496E-05	6.96113E-05
ENSG00000137154	RPS6	-0.25	1.36715E-05	6.97054E-05
ENSG00000189241	TSPYL1	-0.35	1.36885E-05	6.97742E-05
ENSG00000118181	RPS25	-0.25	1.36997E-05	6.98137E-05
ENSG00000108244	KRT23	-1.92	1.37905E-05	7.02586E-05
ENSG00000114735	HEMK1	-0.59	1.38601E-05	7.05952E-05
ENSG00000007866	TEAD3	-0.56	1.38871E-05	7.07146E-05
ENSG00000100280	AP1B1	-0.32	1.39032E-05	7.07787E-05
ENSG00000121390	PSPC1	-0.42	1.39148E-05	7.08198E-05
ENSG00000148297	MED22	-0.51	1.39543E-05	7.10025E-05
ENSG00000104679	R3HCC1	0.44	1.39906E-05	7.11693E-05
ENSG00000147162	OGT	-0.35	1.40816E-05	7.1614E-05
ENSG00000232931	LINC00342	-0.72	1.41277E-05	7.18199E-05
ENSG00000206527	HACD2	-0.44	1.41293E-05	7.18199E-05
ENSG00000042445	RETSAT	-0.38	1.41779E-05	7.20489E-05
ENSG00000178999	AURKB	0.38	1.41917E-05	7.21007E-05
ENSG00000026103	FAS	0.80	1.42598E-05	7.24283E-05
ENSG00000158079	PTPDC1	-0.58	1.44086E-05	7.31652E-05
ENSG00000287188	AC068989.1	2.08	1.44233E-05	7.32212E-05
ENSG00000053524	MCF2L2	1.89	1.44966E-05	7.3575E-05
ENSG00000145362	ANK2	0.83	1.479E-05	7.50449E-05
ENSG00000132646	PCNA	-0.28	1.48019E-05	7.50861E-05
ENSG00000215424	MCM3AP-AS1	-1.22	1.48224E-05	7.51712E-05
ENSG00000023171	GRAMD1B	0.62	1.48526E-05	7.53052E-05
ENSG00000127311	HELB	1.61	1.49551E-05	7.58058E-05
ENSG00000132434	LANCL2	-0.81	1.49747E-05	7.5873E-05
ENSG00000251669	FAM86EP	-1.28	1.49759E-05	7.5873E-05
ENSG00000131171	SH3BGRL	0.53	1.49801E-05	7.5875E-05
ENSG00000196922	ZNF252P	-0.69	1.50011E-05	7.59619E-05
ENSG00000124570	SERPINB6	-0.36	1.51016E-05	7.64515E-05
ENSG00000033627	ATP6V0A1	-0.43	1.51277E-05	7.65642E-05
ENSG00000141314	RHBDL3	-1.04	1.53164E-05	7.74999E-05

ENSG00000243716	NPIPB5	-0.56	1.53375E-05	7.75872E-05
ENSG00000186522	SEPTIN10	0.40	1.53442E-05	7.76013E-05
ENSG00000223722	IFITM3P2	1.92	1.53903E-05	7.78146E-05
ENSG00000100092	SH3BP1	-0.77	1.54083E-05	7.78863E-05
ENSG00000123395	ATG101	0.43	1.543E-05	7.79763E-05
ENSG00000153317	ASAP1	0.38	1.55456E-05	7.85405E-05
ENSG00000249992	TMEM158	0.78	1.56167E-05	7.88799E-05
ENSG00000165650	PDZD8	0.35	1.57431E-05	7.94979E-05
ENSG00000257727	CNPY2	-0.66	1.57661E-05	7.95942E-05
ENSG00000102096	PIM2	0.59	1.58027E-05	7.97586E-05
ENSG00000099290	WASHC2A	0.59	1.58891E-05	8.01748E-05
ENSG00000198612	COPS8	-0.46	1.59119E-05	8.02694E-05
ENSG00000164362	TERT	-1.03	1.60166E-05	8.07773E-05
ENSG00000171435	KSR2	-1.30	1.62649E-05	8.20088E-05
ENSG00000179222	MAGED1	-0.34	1.6293E-05	8.21297E-05
ENSG00000239440	LINC02008	1.64	1.6334E-05	8.23158E-05
ENSG00000125871	MGME1	0.45	1.63593E-05	8.24224E-05
ENSG00000188994	ZNF292	0.64	1.64733E-05	8.29566E-05
ENSG00000131467	PSME3	-0.31	1.64736E-05	8.29566E-05
ENSG00000171490	RSL1D1	-0.32	1.65366E-05	8.32528E-05
ENSG00000197321	SVIL	0.57	1.66043E-05	8.35727E-05
ENSG00000213903	LTB4R	-0.66	1.66828E-05	8.39466E-05
ENSG00000105559	PLEKHA4	0.66	1.67052E-05	8.40383E-05
ENSG00000130723	PRRC2B	-0.37	1.67197E-05	8.40902E-05
ENSG00000196639	HRH1	0.75	1.68497E-05	8.47227E-05
ENSG00000131196	NFATC1	0.63	1.68715E-05	8.48111E-05
ENSG00000230701	FBXW4P1	3.25	1.69205E-05	8.50361E-05
ENSG00000272720	AL022322.1	-2.69	1.69327E-05	8.50759E-05
ENSG00000166801	FAM111A	0.43	1.69823E-05	8.53037E-05
ENSG00000145244	CORIN	1.29	1.71043E-05	8.5895E-05
ENSG00000174243	DDX23	-0.34	1.71272E-05	8.59883E-05
ENSG00000114857	NKTR	-0.44	1.71435E-05	8.60485E-05
ENSG00000273314	AC005229.4	-2.01	1.71638E-05	8.61289E-05
ENSG00000137496	IL18BP	-1.17	1.72083E-05	8.63303E-05
ENSG00000157045	NTAN1	0.57	1.73148E-05	8.6843E-05
ENSG00000279443	AL513497.1	-2.67	1.73224E-05	8.68593E-05
ENSG00000279806	AC018629.1	-1.96	1.73368E-05	8.69097E-05
ENSG00000180370	PAK2	0.33	1.735E-05	8.6954E-05
ENSG00000115539	PDCL3	0.54	1.74286E-05	8.73264E-05
ENSG00000132664	POLR3F	0.73	1.74401E-05	8.73602E-05
ENSG00000100220	RTCB	0.38	1.74441E-05	8.73602E-05
ENSG00000135040	NAA35	-0.50	1.75064E-05	8.76504E-05
ENSG00000127125	PPCS	-0.52	1.76295E-05	8.82446E-05
ENSG00000153485	TMEM251	-0.77	1.7649E-05	8.83201E-05
ENSG00000163013	FBXO41	-0.43	1.76822E-05	8.8464E-05
ENSG00000101608	MYL12A	0.27	1.78775E-05	8.94188E-05

ENSG00000277639	AC007906.2	1.99	1.79167E-05	8.95924E-05
ENSG00000280239	AC011498.7	-1.49	1.79366E-05	8.96509E-05
ENSG00000147394	ZNF185	-0.60	1.79374E-05	8.96509E-05
ENSG00000173812	EIF1	0.29	1.80516E-05	9.01995E-05
ENSG00000137962	ARHGAP29	-0.32	1.81359E-05	9.0598E-05
ENSG00000164587	RPS14	-0.32	1.82212E-05	9.10017E-05
ENSG00000214087	ARL16	0.45	1.83472E-05	9.16081E-05
ENSG00000180596	H2BC4	2.32	1.87055E-05	9.33735E-05
ENSG00000162402	USP24	0.42	1.87423E-05	9.3534E-05
ENSG00000198856	OSTC	-0.34	1.87621E-05	9.36093E-05
ENSG00000272047	GTF2H5	-0.63	1.90804E-05	9.51735E-05
ENSG00000204394	VARS1	-0.30	1.92646E-05	9.60688E-05
ENSG00000186007	LEMD1	-1.28	1.92885E-05	9.6164E-05
ENSG00000211450	SELENOH	-0.37	1.93193E-05	9.62934E-05
ENSG00000131148	EMC8	-0.39	1.94472E-05	9.69066E-05
ENSG00000101773	RBBP8	-0.44	1.94621E-05	9.6957E-05
ENSG00000102241	HTATSF1	0.34	1.97244E-05	9.8239E-05
ENSG00000142544	CTU1	0.69	1.98674E-05	9.89265E-05
ENSG00000225663	MCRIP1	-0.60	1.98927E-05	9.90282E-05
ENSG00000168066	SF1	0.25	1.99908E-05	9.94915E-05
ENSG00000174808	BTC	-1.46	2.00055E-05	9.95398E-05
ENSG00000136068	FLNB	-0.28	2.00361E-05	9.96674E-05
ENSG00000159692	CTBP1	-0.31	2.00628E-05	9.97757E-05
ENSG00000232434	AJM1	0.80	2.00866E-05	9.98652E-05
ENSG00000152117	SMPD4BP	-0.54	2.00908E-05	9.98652E-05
ENSG00000197063	MAFG	0.30	2.01247E-05	0.000100009
ENSG00000073536	NLE1	-0.47	2.02402E-05	0.000100542
ENSG00000105676	ARMC6	0.45	2.0242E-05	0.000100542
ENSG00000177042	TMEM80	-0.96	2.03455E-05	0.000101007
ENSG00000132677	RHBG	1.01	2.03475E-05	0.000101007
ENSG00000163516	ANKZF1	-0.53	2.03508E-05	0.000101007
ENSG00000164093	PITX2	0.76	2.03665E-05	0.000101042
ENSG00000119125	GDA	-0.46	2.0368E-05	0.000101042
ENSG00000234694	AL139289.1	-2.00	2.04511E-05	0.00010143
ENSG00000180902	D2HGDH	-0.64	2.05919E-05	0.000102102
ENSG00000104490	NCALD	2.05	2.07076E-05	0.000102651
ENSG00000119640	ACYP1	-0.65	2.07563E-05	0.000102867
ENSG00000117748	RPA2	0.34	2.08039E-05	0.000103077
ENSG00000163083	INHBB	1.93	2.09306E-05	0.000103679
ENSG00000184967	NOC4L	-0.51	2.09635E-05	0.000103816
ENSG00000112294	ALDH5A1	-0.76	2.10246E-05	0.000104093
ENSG00000124659	TBCC	0.61	2.11402E-05	0.00010464
ENSG00000172935	MRGPRF	2.03	2.1184E-05	0.00010483
ENSG00000110801	PSMD9	0.45	2.12148E-05	0.000104957
ENSG00000105701	FKBP8	-0.32	2.12304E-05	0.000105008
ENSG00000167257	RNF214	0.53	2.1291E-05	0.000105282

ENSG00000197969	VPS13A	-0.46	2.13182E-05	0.00010539
ENSG00000278635	AC141557.2	-5.00	2.13419E-05	0.000105482
ENSG00000136758	YME1L1	0.28	2.13543E-05	0.000105517
ENSG00000267278	MAP3K14-AS1	-1.27	2.15795E-05	0.000106603
ENSG00000164190	NIPBL	0.43	2.16376E-05	0.000106864
ENSG00000197978	GOLGA6L9	-0.84	2.16459E-05	0.000106878
ENSG00000147140	NONO	-0.24	2.16757E-05	0.000106999
ENSG00000175048	ZDHHC14	0.83	2.17797E-05	0.000107486
ENSG00000103202	NME4	0.40	2.19855E-05	0.000108475
ENSG00000107672	NSMCE4A	-0.41	2.24343E-05	0.000110662
ENSG00000197894	ADH5	0.30	2.24669E-05	0.000110795
ENSG00000169710	FASN	-0.26	2.26889E-05	0.000111862
ENSG00000165417	GTF2A1	0.38	2.27225E-05	0.000112001
ENSG00000069849	ATP1B3	0.29	2.27296E-05	0.000112008
ENSG00000174943	KCTD13	-0.64	2.2736E-05	0.000112012
ENSG00000128833	MYO5C	-0.49	2.27451E-05	0.000112029
ENSG00000197265	GTF2E2	0.40	2.27556E-05	0.000112053
ENSG00000204267	TAP2	-0.51	2.28027E-05	0.000112258
ENSG00000108509	CAMTA2	-0.49	2.29223E-05	0.000112819
ENSG00000172831	CES2	-0.47	2.29799E-05	0.000113074
ENSG00000183726	TMEM50A	0.36	2.29964E-05	0.000113128
ENSG00000204438	GPANK1	0.52	2.31521E-05	0.000113866
ENSG00000103222	ABCC1	-0.32	2.32059E-05	0.000114102
ENSG00000183479	TREX2	-2.11	2.32243E-05	0.000114138
ENSG00000104517	UBR5	0.31	2.32246E-05	0.000114138
ENSG00000142945	KIF2C	0.29	2.34211E-05	0.000115076
ENSG00000144893	MED12L	0.97	2.35133E-05	0.0001155
ENSG00000071462	BUD23	0.29	2.35556E-05	0.000115679
ENSG00000091140	DLD	0.32	2.36449E-05	0.000116089
ENSG00000161509	GRIN2C	-1.63	2.36506E-05	0.000116089
ENSG00000125657	TNFSF9	0.51	2.36729E-05	0.00011617
ENSG00000176410	DNAJC30	-0.88	2.3686E-05	0.000116206
ENSG00000100578	KIAA0586	0.63	2.37573E-05	0.000116527
ENSG00000108679	LGALS3BP	-0.29	2.39443E-05	0.000117416
ENSG00000132824	SERINC3	0.31	2.39519E-05	0.000117424
ENSG00000178229	ZNF543	0.92	2.39986E-05	0.000117624
ENSG00000104892	KLC3	0.54	2.40939E-05	0.000118063
ENSG00000054116	TRAPPC3	-0.41	2.41099E-05	0.000118112
ENSG00000146072	TNFRSF21	0.37	2.41889E-05	0.00011847
ENSG00000247572	CKMT2-AS1	-1.04	2.43038E-05	0.000119004
ENSG00000079393	DUSP13	2.67	2.4357E-05	0.000119235
ENSG00000137812	KNL1	0.45	2.45475E-05	0.00012012
ENSG00000230513	THAP7-AS1	-1.29	2.45497E-05	0.00012012
ENSG00000032742	IFT88	-0.93	2.46088E-05	0.000120379
ENSG00000281490	CICP14	-1.11	2.46269E-05	0.000120438
ENSG00000163463	KRTCAP2	0.56	2.46958E-05	0.000120746

ENSG00000116793	PHTF1	0.62	2.48784E-05	0.000121609
ENSG00000100151	PICK1	0.47	2.48927E-05	0.000121649
ENSG00000184939	ZFP90	-0.65	2.49534E-05	0.000121916
ENSG00000218418	AL591135.1	1.65	2.49769E-05	0.000121965
ENSG00000241506	PSMC1P1	0.42	2.49788E-05	0.000121965
ENSG00000143156	NME7	0.58	2.49867E-05	0.000121965
ENSG00000163817	SLC6A20	1.97	2.49878E-05	0.000121965
ENSG00000086015	MAST2	-0.36	2.51449E-05	0.000122702
ENSG00000113569	NUP155	0.33	2.52488E-05	0.000123179
ENSG00000169241	SLC50A1	-0.39	2.53693E-05	0.000123736
ENSG00000171792	RHNO1	-0.57	2.54636E-05	0.000124166
ENSG00000256982	AC135782.1	2.18	2.57519E-05	0.000125541
ENSG00000130311	DDA1	0.34	2.60385E-05	0.000126908
ENSG00000129933	MAU2	-0.47	2.60895E-05	0.000127125
ENSG00000128346	C22orf23	-2.08	2.61201E-05	0.000127214
ENSG00000171503	ETFDH	0.55	2.61204E-05	0.000127214
ENSG00000177595	PIDD1	-0.56	2.6148E-05	0.000127317
ENSG00000131844	MCCC2	-0.40	2.62536E-05	0.0001278
ENSG00000157856	DRC1	6.82	2.63453E-05	0.000128216
ENSG00000121210	TMEM131L	-0.51	2.63699E-05	0.000128304
ENSG00000173456	RNF26	-0.39	2.64967E-05	0.000128889
ENSG00000123411	IKZF4	-0.94	2.66212E-05	0.000129464
ENSG00000161671	EMC10	-0.32	2.6629E-05	0.00012947
ENSG00000232023	LINC01807	1.88	2.67216E-05	0.000129889
ENSG00000107859	PITX3	1.43	2.67305E-05	0.000129901
ENSG00000171453	POLR1C	-0.43	2.68473E-05	0.000130436
ENSG00000137393	RNF144B	1.17	2.70497E-05	0.000131388
ENSG00000237595	LINC01275	2.61	2.71545E-05	0.000131865
ENSG00000137955	RABGGTB	-0.30	2.72464E-05	0.000132279
ENSG00000109944	JHY	0.99	2.72911E-05	0.000132464
ENSG00000152669	CCNO	0.79	2.73449E-05	0.000132693
ENSG00000176723	ZNF843	1.90	2.74827E-05	0.000133303
ENSG00000248049	UBA6-AS1	0.77	2.74839E-05	0.000133303
ENSG00000056586	RC3H2	-0.37	2.75104E-05	0.000133399
ENSG00000091732	ZC3HC1	0.57	2.75681E-05	0.000133646
ENSG00000276043	UHRF1	-0.31	2.76508E-05	0.000134015
ENSG00000261188	Z95115.1	-1.58	2.79609E-05	0.000135485
ENSG00000160799	CCDC12	0.41	2.79816E-05	0.000135553
ENSG00000152558	TMEM123	-0.32	2.80819E-05	0.000136006
ENSG00000104419	NDRG1	0.76	2.8429E-05	0.000137653
ENSG00000115163	CENPA	0.46	2.85764E-05	0.000138334
ENSG00000250132	AC004803.1	-1.97	2.8685E-05	0.000138826
ENSG00000174775	HRAS	0.38	2.87108E-05	0.000138917
ENSG00000204219	TCEA3	0.89	2.87268E-05	0.000138961
ENSG00000137693	YAP1	-0.33	2.88515E-05	0.00013953
ENSG00000173621	LRFN4	-0.56	2.91112E-05	0.000140752

ENSG00000185658	BRWD1	-0.45	2.93069E-05	0.000141664
ENSG00000170881	RNF139	0.46	2.93604E-05	0.000141888
ENSG00000185808	PIGP	-0.73	2.9431E-05	0.000142165
ENSG00000172765	TMCC1	-0.70	2.94317E-05	0.000142165
ENSG00000088305	DNMT3B	-0.52	2.94777E-05	0.000142352
ENSG00000257267	ZNF271P	0.59	2.95222E-05	0.000142533
ENSG00000108306	FBXL20	-0.62	2.96349E-05	0.000143042
ENSG00000166855	CLPX	0.41	2.97576E-05	0.0001436
ENSG00000130956	HABP4	0.45	3.00353E-05	0.000144905
ENSG00000006062	MAP3K14	0.52	3.02357E-05	0.000145837
ENSG00000181830	SLC35C1	-0.51	3.02813E-05	0.000146022
ENSG00000257242	LINC01619	4.25	3.03206E-05	0.000146176
ENSG00000250510	GPR162	0.81	3.04783E-05	0.000146901
ENSG00000228315	GUSBP11	-1.01	3.05115E-05	0.000147025
ENSG00000063241	ISOC2	0.42	3.10071E-05	0.000149377
ENSG00000151025	GPR158	1.57	3.11011E-05	0.000149794
ENSG00000100167	SEPTIN3	0.55	3.11474E-05	0.000149981
ENSG00000181135	ZNF707	0.63	3.11851E-05	0.000150127
ENSG00000177045	SIX5	-1.09	3.13695E-05	0.000150978
ENSG00000174032	SLC25A30	0.46	3.13782E-05	0.000150983
ENSG00000247081	BAALC-AS1	-1.24	3.13987E-05	0.000151046
ENSG00000164284	GRPEL2	0.36	3.15933E-05	0.000151945
ENSG00000047621	C12orf4	0.76	3.17137E-05	0.000152488
ENSG00000239653	PSMD6-AS2	2.59	3.19765E-05	0.000153714
ENSG00000086544	ITPKC	0.60	3.19872E-05	0.000153729
ENSG00000146733	PSPH	0.35	3.22226E-05	0.000154823
ENSG00000134910	STT3A	0.26	3.22641E-05	0.000154986
ENSG00000180818	HOXC10	1.01	3.23589E-05	0.000155404
ENSG00000242294	STAG3L5P	-0.73	3.24005E-05	0.000155566
ENSG00000078061	ARAF	-0.50	3.2729E-05	0.000157106
ENSG00000090263	MRPS33	-0.46	3.27472E-05	0.000157155
ENSG00000226200	SGMS1-AS1	-1.73	3.27835E-05	0.000157291
ENSG00000269556	TMEM185A	0.67	3.27946E-05	0.000157307
ENSG00000174444	RPL4	-0.25	3.28551E-05	0.00015756
ENSG00000166681	BEX3	0.32	3.28783E-05	0.000157633
ENSG00000145817	YIPF5	0.51	3.28995E-05	0.000157697
ENSG00000230487	PSMG3-AS1	-1.56	3.29511E-05	0.000157907
ENSG00000101546	RBFA	-0.47	3.30431E-05	0.000158309
ENSG00000166313	APBB1	0.75	3.31669E-05	0.000158865
ENSG00000006638	TBXA2R	1.22	3.32834E-05	0.000159385
ENSG00000140854	KATNB1	-0.48	3.34397E-05	0.000160095
ENSG00000163110	PDLIM5	-0.47	3.35399E-05	0.000160536
ENSG00000106012	IQCE	-0.50	3.3593E-05	0.000160752
ENSG00000168481	LGI3	1.31	3.3636E-05	0.000160919
ENSG00000070501	POLB	0.60	3.38867E-05	0.000162079
ENSG00000128607	KLHDC10	0.41	3.38994E-05	0.000162101

ENSG00000183172	SMDT1	0.64	3.39307E-05	0.000162212
ENSG00000163322	ABRAXAS1	-0.93	3.40027E-05	0.000162518
ENSG00000122966	CIT	0.41	3.40232E-05	0.000162577
ENSG00000108389	MTMR4	-0.34	3.41008E-05	0.000162909
ENSG00000092853	CLSPN	-0.44	3.41365E-05	0.00016304
ENSG00000146729	NIPSNAP2	-0.46	3.41446E-05	0.00016304
ENSG00000233016	SNHG7	-0.39	3.42139E-05	0.000163332
ENSG00000174177	CTU2	-0.41	3.43432E-05	0.000163911
ENSG00000249915	PDCD6	-0.93	3.45393E-05	0.000164807
ENSG00000144567	RETREG2	-0.37	3.46415E-05	0.000165255
ENSG00000141644	MBD1	0.38	3.50665E-05	0.000167243
ENSG00000083845	RPS5	-0.30	3.50944E-05	0.000167336
ENSG00000137135	ARHGEF39	-0.55	3.51819E-05	0.000167713
ENSG00000214655	ZSWIM8	-0.48	3.53359E-05	0.000168407
ENSG00000142046	TMEM91	-2.07	3.53561E-05	0.000168458
ENSG00000162407	PLPP3	0.92	3.53633E-05	0.000168458
ENSG00000204256	BRD2	0.24	3.53784E-05	0.00016849
ENSG00000143157	POGK	0.38	3.54018E-05	0.000168561
ENSG00000165219	GAPVD1	0.36	3.54273E-05	0.000168642
ENSG00000009844	VTA1	0.37	3.55981E-05	0.000169415
ENSG00000172922	RNASEH2C	0.47	3.57406E-05	0.000170052
ENSG00000175348	TMEM9B	0.39	3.58089E-05	0.000170337
ENSG00000054179	ENTPD2	-0.75	3.60527E-05	0.000171456
ENSG00000108950	FAM20A	2.36	3.60998E-05	0.000171639
ENSG00000213638	ADAT3	0.82	3.61379E-05	0.000171779
ENSG00000286019	NOTCH2NLB	2.41	3.62382E-05	0.000172215
ENSG00000118503	TNFAIP3	1.17	3.62639E-05	0.000172296
ENSG00000253540	FAM86HP	-2.11	3.63189E-05	0.000172517
ENSG00000133392	MYH11	1.68	3.64537E-05	0.000173116
ENSG00000154153	RETREG1	1.19	3.6853E-05	0.000174971
ENSG00000133265	HSPBP1	-0.48	3.68749E-05	0.000175034
ENSG00000119574	ZBTB45	-0.81	3.69599E-05	0.000175395
ENSG00000159714	ZDHHC1	-1.24	3.69757E-05	0.000175429
ENSG00000153250	RBMS1	0.51	3.70942E-05	0.000175949
ENSG00000012061	ERCC1	0.42	3.74358E-05	0.000177527
ENSG00000187957	DNER	1.58	3.77392E-05	0.000178924
ENSG00000198026	ZNF335	0.54	3.79082E-05	0.000179682
ENSG00000136536	MARCHF7	0.38	3.79449E-05	0.000179814
ENSG00000163683	SMIM14	0.54	3.81654E-05	0.000180816
ENSG00000116670	MAD2L2	0.36	3.819E-05	0.00018089
ENSG00000174099	MSRB3	0.57	3.83111E-05	0.00018142
ENSG00000203709	MIR29B2CHG	1.68	3.83365E-05	0.000181498
ENSG00000105855	ITGB8	-1.50	3.85284E-05	0.000182363
ENSG00000147234	FRMPD3	1.53	3.8725E-05	0.00018325
ENSG00000204228	HSD17B8	-0.76	3.88254E-05	0.000183682
ENSG00000084070	SMAP2	-0.48	3.89985E-05	0.000184457

ENSG00000181264	TLCD5	-0.64	3.92908E-05	0.000185796
ENSG00000186130	ZBTB6	0.54	3.94006E-05	0.000186271
ENSG00000100867	DHRS2	-0.57	3.94523E-05	0.000186471
ENSG00000136826	KLF4	0.45	3.95191E-05	0.000186743
ENSG00000149292	TTC12	-1.01	3.97415E-05	0.00018775
ENSG00000130255	RPL36	-0.32	3.98465E-05	0.000188201
ENSG00000141401	IMPA2	-1.61	3.98784E-05	0.000188307
ENSG00000198873	GRK5	0.72	4.00133E-05	0.0001889
ENSG00000198625	MDM4	-0.34	4.03133E-05	0.000190271
ENSG00000185664	PMEL	1.07	4.03394E-05	0.00019035
ENSG00000101986	ABCD1	0.67	4.03724E-05	0.00019046
ENSG00000078668	VDAC3	0.29	4.04158E-05	0.00019062
ENSG00000144228	SPOPL	0.45	4.08309E-05	0.000192533
ENSG00000114388	NPRL2	-0.55	4.09654E-05	0.000193121
ENSG00000167543	TP53I13	-0.57	4.10109E-05	0.00019329
ENSG00000114209	PDCD10	0.44	4.10661E-05	0.000193505
ENSG00000197483	ZNF628	0.86	4.1144E-05	0.000193826
ENSG00000137274	BPHL	-0.65	4.12506E-05	0.000194258
ENSG00000100813	ACIN1	0.25	4.1255E-05	0.000194258
ENSG00000185101	ANO9	-0.56	4.13709E-05	0.000194758
ENSG00000130348	QRSL1	0.61	4.15118E-05	0.000195375
ENSG00000138398	PPIG	0.36	4.17278E-05	0.000196345
ENSG00000084676	NCOA1	0.60	4.21621E-05	0.000198342
ENSG00000100258	LMF2	-0.44	4.23115E-05	0.000198979
ENSG00000120053	GOT1	-0.30	4.23174E-05	0.000198979
ENSG00000186812	ZNF397	-0.55	4.24146E-05	0.000199389
ENSG00000260136	AC008915.3	1.44	4.2524E-05	0.000199857
ENSG00000183617	MRPL54	0.43	4.27018E-05	0.000200645
ENSG00000175470	PPP2R2D	0.54	4.27471E-05	0.000200811
ENSG00000125447	GGA3	-0.37	4.28755E-05	0.000201367
ENSG00000164967	RPP25L	-0.53	4.29394E-05	0.00020162
ENSG00000124225	PMEPA1	1.52	4.32501E-05	0.000203031
ENSG00000156709	AIFM1	0.39	4.34183E-05	0.000203773
ENSG00000153246	PLA2R1	2.81	4.34553E-05	0.000203899
ENSG00000204370	SDHD	-0.41	4.35681E-05	0.00020438
ENSG00000157881	PANK4	0.47	4.37014E-05	0.000204957
ENSG00000105219	CCNP	-1.54	4.37302E-05	0.000205044
ENSG00000117280	RAB29	0.51	4.37525E-05	0.000205101
ENSG00000164010	ERMAP	-1.12	4.38095E-05	0.00020532
ENSG00000120029	ARMH3	-0.41	4.40604E-05	0.000206447
ENSG00000169692	AGPAT2	0.41	4.43236E-05	0.000207616
ENSG00000129084	PSMA1	0.27	4.43305E-05	0.000207616
ENSG00000183317	EPHA10	-0.87	4.44283E-05	0.000208025
ENSG00000120899	PTK2B	-0.73	4.45361E-05	0.000208481
ENSG00000166888	STAT6	-0.40	4.46807E-05	0.000209109
ENSG00000013810	TACC3	0.28	4.49105E-05	0.000210135

ENSG00000180626	ZNF594	-1.05	4.52732E-05	0.000211783
ENSG00000161981	SNRNP25	-0.52	4.53789E-05	0.0002122
ENSG00000105088	OLFM2	-0.82	4.53903E-05	0.0002122
ENSG00000081721	DUSP12	0.47	4.53941E-05	0.0002122
ENSG00000257732	AC089983.1	2.07	4.54193E-05	0.000212268
ENSG00000214253	FIS1	0.41	4.55674E-05	0.00021291
ENSG00000170049	KCNAB3	-2.01	4.55932E-05	0.000212981
ENSG00000113328	CCNG1	-0.38	4.57619E-05	0.000213682
ENSG00000111206	FOXM1	-0.32	4.57646E-05	0.000213682
ENSG00000101452	DHX35	-0.68	4.58019E-05	0.000213801
ENSG00000171793	CTPS1	-0.36	4.58114E-05	0.000213801
ENSG00000280206	AC026401.3	0.82	4.61231E-05	0.000215205
ENSG00000184924	PTRHD1	0.58	4.63373E-05	0.000216154
ENSG00000162614	NEXN	2.68	4.69565E-05	0.000218958
ENSG00000173805	HAP1	0.96	4.69602E-05	0.000218958
ENSG00000023909	GCLM	0.38	4.75251E-05	0.00022154
ENSG00000204348	DXO	-0.67	4.75534E-05	0.00022162
ENSG00000167862	MRPL58	-0.45	4.75794E-05	0.000221643
ENSG00000140545	MFGE8	0.39	4.75804E-05	0.000221643
ENSG00000166016	ABTB2	0.52	4.77396E-05	0.000222333
ENSG00000116898	MRPS15	0.34	4.82854E-05	0.000224823
ENSG00000205084	TMEM231	-1.11	4.83038E-05	0.000224856
ENSG00000114166	KAT2B	1.08	4.83363E-05	0.000224955
ENSG00000111319	SCNN1A	-0.37	4.84499E-05	0.000225431
ENSG00000268223	ARL14EPL	-1.21	4.86606E-05	0.000226359
ENSG00000165943	MOAP1	0.53	4.88731E-05	0.000227264
ENSG00000184110	EIF3C	0.33	4.88778E-05	0.000227264
ENSG00000167861	HID1	0.46	4.90578E-05	0.000228048
ENSG00000122482	ZNF644	0.45	4.91346E-05	0.000228314
ENSG00000183458	AC138932.1	-1.15	4.91379E-05	0.000228314
ENSG00000182511	FES	-0.82	4.9381E-05	0.00022939
ENSG00000127952	STYXL1	-0.58	4.96845E-05	0.000230746
ENSG00000188157	AGRN	-0.33	4.99937E-05	0.000232129
ENSG00000130714	POMT1	0.58	5.01919E-05	0.000232995
ENSG00000160551	TAOK1	0.38	5.03571E-05	0.000233664
ENSG00000117614	SYF2	0.38	5.03594E-05	0.000233664
ENSG00000099364	FBXL19	0.35	5.0871E-05	0.000235983
ENSG00000156508	EEF1A1	-0.36	5.09817E-05	0.000236442
ENSG00000021574	SPAST	0.55	5.11329E-05	0.000237088
ENSG00000102057	KCND1	-0.92	5.14372E-05	0.000238444
ENSG00000109255	NMU	0.44	5.16957E-05	0.000239586
ENSG00000122566	HNRNPA2B1	0.24	5.17794E-05	0.000239899
ENSG00000153107	ANAPC1	-0.40	5.17871E-05	0.000239899
ENSG00000123643	SLC36A1	0.76	5.18703E-05	0.000240229
ENSG00000186230	ZNF749	-0.73	5.19659E-05	0.000240616
ENSG00000115306	SPTBN1	0.31	5.21088E-05	0.000241222

ENSG00000178445	GLDC	0.73	5.28133E-05	0.000244426
ENSG00000143740	SNAP47	0.51	5.28592E-05	0.000244582
ENSG00000120253	NUP43	-0.40	5.29711E-05	0.000245043
ENSG00000171903	CYP4F11	0.51	5.33513E-05	0.000246745
ENSG00000254999	BRK1	0.34	5.35454E-05	0.000247586
ENSG00000136280	CCM2	-0.48	5.35652E-05	0.00024762
ENSG00000168517	HEXIM2	1.07	5.3862E-05	0.000248935
ENSG00000152332	UHMK1	0.27	5.41309E-05	0.00025012
ENSG00000100567	PSMA3	0.33	5.41689E-05	0.000250237
ENSG00000100147	CCDC134	0.97	5.42505E-05	0.000250557
ENSG00000228253	MT-ATP8	-0.34	5.43358E-05	0.000250893
ENSG00000110057	UNC93B1	-0.40	5.45915E-05	0.000252015
ENSG00000136379	ABHD17C	-0.55	5.49435E-05	0.000253581
ENSG00000165675	ENOX2	-0.92	5.49651E-05	0.000253592
ENSG00000131620	ANO1	-0.44	5.4971E-05	0.000253592
ENSG00000198901	PRC1	0.44	5.50121E-05	0.000253723
ENSG00000187833	C2orf78	2.18	5.50604E-05	0.000253887
ENSG00000167157	PRRX2	0.62	5.53639E-05	0.000255227
ENSG00000139437	TCHP	0.51	5.54917E-05	0.000255758
ENSG00000250917	AL035458.2	-3.15	5.55225E-05	0.000255823
ENSG00000066651	TRMT11	-0.50	5.55314E-05	0.000255823
ENSG00000178538	CA8	0.90	5.56705E-05	0.000256404
ENSG00000206417	H1-10-AS1	2.52	5.57945E-05	0.000256916
ENSG00000138622	HCN4	-1.15	5.58218E-05	0.000256983
ENSG00000140931	CMTM3	0.51	5.60617E-05	0.000258028
ENSG00000108774	RAB5C	0.30	5.60967E-05	0.00025813
ENSG00000100058	CRYBB2P1	0.63	5.62813E-05	0.00025892
ENSG00000132383	RPA1	0.25	5.63235E-05	0.000259054
ENSG00000173801	JUP	0.28	5.63523E-05	0.000259127
ENSG00000118260	CREB1	-0.61	5.68437E-05	0.000261327
ENSG00000218175	AC016739.1	-1.44	5.70447E-05	0.000262191
ENSG00000146707	POMZP3	-1.03	5.71803E-05	0.000262691
ENSG00000154723	ATP5PF	0.34	5.71903E-05	0.000262691
ENSG00000102218	RP2	0.67	5.7193E-05	0.000262691
ENSG00000001630	CYP51A1	0.81	5.73065E-05	0.000263152
ENSG00000139182	CLSTN3	0.44	5.74642E-05	0.000263816
ENSG00000183688	RFLNB	-0.42	5.7608E-05	0.000264415
ENSG00000113916	BCL6	0.57	5.76648E-05	0.000264615
ENSG00000138286	FAM149B1	-0.69	5.77438E-05	0.000264917
ENSG00000022567	SLC45A4	0.56	5.77626E-05	0.000264943
ENSG00000033011	ALG1	0.51	5.80538E-05	0.000266218
ENSG00000116729	WLS	0.41	5.80928E-05	0.000266335
ENSG00000113734	BNIP1	0.61	5.85064E-05	0.00026817
ENSG00000168071	CCDC88B	0.60	5.86475E-05	0.000268755
ENSG00000155621	C9orf85	0.75	5.90305E-05	0.000270448
ENSG00000165138	ANKS6	-0.41	5.91634E-05	0.000270995

ENSG00000260400	AL513534.2	1.46	5.91861E-05	0.000271037
ENSG00000161921	CXCL16	-0.40	5.92896E-05	0.000271449
ENSG00000170027	YWHAG	-0.25	5.93827E-05	0.000271813
ENSG00000112531	QKI	0.41	5.9816E-05	0.000273734
ENSG00000038210	PI4K2B	-0.55	5.99495E-05	0.000274282
ENSG00000143815	LBR	-0.29	6.01541E-05	0.000275155
ENSG00000068079	IFI35	0.58	6.02514E-05	0.00027548
ENSG00000112182	BACH2	1.17	6.02527E-05	0.00027548
ENSG00000172785	CBWD1	0.42	6.07224E-05	0.000277564
ENSG00000008323	PLEKHG6	0.63	6.07913E-05	0.000277816
ENSG00000128928	IVD	-0.37	6.08217E-05	0.000277891
ENSG00000153975	ZUP1	0.61	6.08517E-05	0.000277965
ENSG00000184508	HDDC3	-0.76	6.11461E-05	0.000279246
ENSG00000007168	PAFAH1B1	0.28	6.12607E-05	0.000279705
ENSG00000166147	FBN1	1.64	6.13673E-05	0.000280128
ENSG00000142867	BCL10	0.51	6.15026E-05	0.000280682
ENSG00000138629	UBL7	-0.42	6.15337E-05	0.00028076
ENSG00000205903	ZNF316	0.42	6.17956E-05	0.00028189
ENSG00000110700	RPS13	-0.27	6.19179E-05	0.000282384
ENSG00000172115	CYCS	-0.25	6.26092E-05	0.000285472
ENSG00000169762	TAPT1	0.91	6.28504E-05	0.000286506
ENSG00000188938	FAM120AOS	-0.46	6.30206E-05	0.000287217
ENSG00000241015	TPM3P9	-1.61	6.31338E-05	0.000287667
ENSG00000132952	USPL1	0.61	6.34072E-05	0.000288847
ENSG00000242247	ARFGAP3	0.58	6.35727E-05	0.000289535
ENSG00000205746	AC126755.1	-1.29	6.37031E-05	0.000290063
ENSG00000131061	ZNF341	0.92	6.40034E-05	0.000291364
ENSG00000166548	TK2	0.51	6.43296E-05	0.000292783
ENSG00000113068	PFDN1	0.42	6.47008E-05	0.000294405
ENSG00000243317	STMP1	-0.36	6.47333E-05	0.000294486
ENSG00000177238	TRIM72	0.36	6.55122E-05	0.000297961
ENSG00000122068	FYTTD1	0.33	6.5643E-05	0.000298489
ENSG00000175315	CST6	0.42	6.60829E-05	0.000300421
ENSG00000012822	CALCOCO1	-0.52	6.62406E-05	0.000301069
ENSG00000168502	MTCL1	-0.54	6.65113E-05	0.000302231
ENSG00000168802	CHTF8	-0.52	6.65382E-05	0.000302271
ENSG00000101464	PIGU	-0.57	6.65504E-05	0.000302271
ENSG00000183682	BMP8A	-0.80	6.69415E-05	0.000303978
ENSG00000230989	HSBP1	0.25	6.6967E-05	0.000304025
ENSG00000066027	PPP2R5A	0.41	6.77146E-05	0.00030735
ENSG00000109065	NAT9	-0.38	6.78464E-05	0.000307878
ENSG00000204427	ABHD16A	-1.08	6.85325E-05	0.000310921
ENSG00000140299	BNIP2	0.35	6.86405E-05	0.00031134
ENSG00000255320	AP000759.1	-2.41	6.89158E-05	0.000312518
ENSG00000112473	SLC39A7	0.26	6.89989E-05	0.000312758
ENSG00000163032	VSNL1	-0.76	6.89999E-05	0.000312758

ENSG00000136044	APPL2	-0.63	6.96538E-05	0.00031565
ENSG00000144909	OSBPL11	0.56	6.96999E-05	0.000315788
ENSG00000241127	YAE1	-1.00	6.9842E-05	0.00031636
ENSG00000128165	ADM2	-0.56	7.04913E-05	0.000319229
ENSG00000124067	SLC12A4	0.41	7.07471E-05	0.000320315
ENSG00000165272	AQP3	0.74	7.07867E-05	0.000320422
ENSG00000204287	HLA-DRA	1.44	7.10328E-05	0.000321463
ENSG00000203883	SOX18	0.97	7.10594E-05	0.00032151
ENSG00000226180	AC010536.1	-1.38	7.14658E-05	0.000323276
ENSG00000173486	FKBP2	0.78	7.15822E-05	0.000323729
ENSG00000132406	TMEM128	-0.55	7.18575E-05	0.000324901
ENSG00000274211	SOCS7	0.34	7.20229E-05	0.000325575
ENSG00000270240	AC015849.1	4.12	7.21634E-05	0.000326137
ENSG00000102103	PQBP1	0.37	7.31473E-05	0.000330509
ENSG00000188747	NOXA1	-0.88	7.42367E-05	0.000335355
ENSG00000198498	TMA16	0.43	7.4328E-05	0.000335683
ENSG00000123130	ACOT9	0.34	7.43429E-05	0.000335683
ENSG00000150764	DIXDC1	0.75	7.44273E-05	0.000335959
ENSG00000106723	SPIN1	0.36	7.44375E-05	0.000335959
ENSG00000100744	GSKIP	0.56	7.48231E-05	0.000337624
ENSG00000171160	MORN4	-0.83	7.48508E-05	0.000337672
ENSG00000104885	DOT1L	0.35	7.5079E-05	0.000338626
ENSG00000125841	NRSN2	-2.61	7.51098E-05	0.000338688
ENSG00000100335	MIEF1	-0.34	7.53993E-05	0.000339917
ENSG00000164048	ZNF589	-0.55	7.56716E-05	0.000341068
ENSG00000239887	C1orf226	-0.58	7.58061E-05	0.000341597
ENSG00000173674	EIF1AX	-0.37	7.58579E-05	0.000341753
ENSG00000004848	ARX	2.13	7.5933E-05	0.000342015
ENSG00000154781	CCDC174	0.55	7.61987E-05	0.000343134
ENSG00000107829	FBXW4	-0.50	7.70041E-05	0.000346683
ENSG00000111832	RWDD1	-0.64	7.70896E-05	0.00034699
ENSG00000164638	SLC29A4	-0.63	7.72576E-05	0.000347668
ENSG00000173327	MAP3K11	0.34	7.73652E-05	0.000348074
ENSG00000065518	NDUFB4	0.32	7.74495E-05	0.000348375
ENSG00000106683	LIMK1	-0.39	7.76331E-05	0.000349122
ENSG00000111199	TRPV4	2.13	7.82113E-05	0.000351643
ENSG00000101935	AMMECR1	0.53	7.96335E-05	0.000357869
ENSG00000141759	TXNL4A	0.27	7.96486E-05	0.000357869
ENSG00000274523	RCC1L	0.40	7.96496E-05	0.000357869
ENSG00000163781	TOPBP1	0.34	7.96778E-05	0.000357915
ENSG00000109320	NFKB1	-0.44	7.97672E-05	0.000358236
ENSG00000223705	NSUN5P1	-0.50	7.9919E-05	0.000358838
ENSG00000278615	C11orf98	-0.42	7.99905E-05	0.000359078
ENSG00000163485	ADORA1	-1.20	8.03353E-05	0.000360545
ENSG00000111801	BTN3A3	1.07	8.05184E-05	0.000361286
ENSG00000160867	FGFR4	-0.42	8.08454E-05	0.000362672

ENSG00000185591	SP1	-0.29	8.15115E-05	0.000365578
ENSG00000267002	AC060780.1	-0.81	8.16553E-05	0.000366132
ENSG00000097046	CDC7	-0.47	8.16716E-05	0.000366132
ENSG00000109814	UGDH	0.38	8.18605E-05	0.000366896
ENSG00000233834	AC005083.1	1.67	8.20248E-05	0.00036755
ENSG00000164211	STARD4	0.38	8.21951E-05	0.000368231
ENSG00000196465	MYL6B	0.39	8.23469E-05	0.000368828
ENSG00000183049	CAMK1D	-0.77	8.24114E-05	0.000369035
ENSG00000144791	LIMD1	-0.61	8.25544E-05	0.000369593
ENSG00000276672	AL161891.1	-2.08	8.28673E-05	0.00037091
ENSG00000167635	ZNF146	-0.33	8.29747E-05	0.000371308
ENSG00000197594	ENPP1	-0.79	8.32636E-05	0.000372518
ENSG00000141570	CBX8	0.43	8.34302E-05	0.00037318
ENSG00000153786	ZDHHC7	-0.33	8.34935E-05	0.000373379
ENSG00000163328	GPR155	0.97	8.40853E-05	0.000375942
ENSG00000244026	FAM86DP	-0.89	8.44291E-05	0.000377394
ENSG00000158711	ELK4	-0.38	8.4514E-05	0.000377689
ENSG00000163479	SSR2	0.33	8.45864E-05	0.000377929
ENSG00000269609	RPARP-AS1	-0.56	8.49076E-05	0.000379279
ENSG00000135775	COG2	-0.59	8.54006E-05	0.000381396
ENSG00000182512	GLRX5	-0.35	8.54618E-05	0.000381585
ENSG00000280099	AL603750.1	-2.66	8.55408E-05	0.000381852
ENSG00000151287	TEX30	-0.60	8.59742E-05	0.000383701
ENSG00000102910	LONP2	0.38	8.61007E-05	0.00038418
ENSG00000150907	FOXO1	-0.84	8.6402E-05	0.000385438
ENSG00000124357	NAGK	0.42	8.65458E-05	0.000385994
ENSG00000175390	EIF3F	0.32	8.69377E-05	0.000387655
ENSG00000085185	BCORL1	0.38	8.72019E-05	0.000388747
ENSG00000166471	TMEM41B	-0.35	8.72343E-05	0.000388804
ENSG00000133731	IMPA1	-0.38	8.74873E-05	0.000389846
ENSG00000204685	STARD7-AS1	-1.17	8.75728E-05	0.000390139
ENSG00000173269	MMRN2	0.95	8.77382E-05	0.000390789
ENSG00000109066	TMEM104	-0.54	8.79846E-05	0.0003918
ENSG00000085733	CTTN	0.25	8.80167E-05	0.000391855
ENSG00000112079	STK38	-0.40	8.8042E-05	0.000391881
ENSG00000226510	UPK1A-AS1	1.37	8.82961E-05	0.000392924
ENSG00000128610	FEZF1	1.83	8.83554E-05	0.000393101
ENSG00000065150	IPO5	-0.24	8.85861E-05	0.000394032
ENSG00000103199	ZNF500	0.66	8.86126E-05	0.000394032
ENSG00000206053	JPT2	-0.25	8.86237E-05	0.000394032
ENSG00000111880	RNGTT	0.44	8.88836E-05	0.0003951
ENSG00000198804	MT-CO1	-0.45	8.90439E-05	0.000395724
ENSG00000127152	BCL11B	1.81	8.90926E-05	0.000395853
ENSG00000125885	MCM8	-0.73	8.96201E-05	0.000398108
ENSG00000261587	TMEM249	1.43	8.96431E-05	0.000398122
ENSG00000186908	ZDHHC17	-0.57	8.98254E-05	0.000398843

ENSG00000132478	UNK	-0.41	9.01856E-05	0.000400354
ENSG00000170775	GPR37	1.09	9.03107E-05	0.00040082
ENSG00000158286	RNF207	-0.51	9.03592E-05	0.000400947
ENSG00000134901	POGLUT2	0.80	9.17215E-05	0.000406901
ENSG00000114784	EIF1B	0.44	9.19526E-05	0.000407836
ENSG00000145247	OCIAD2	0.47	9.23405E-05	0.000409465
ENSG00000182903	ZNF721	0.51	9.26602E-05	0.000410792
ENSG00000189143	CLDN4	0.30	9.30053E-05	0.000412231
ENSG00000164414	SLC35A1	-0.76	9.38583E-05	0.000415919
ENSG00000105698	USF2	0.34	9.40506E-05	0.000416679
ENSG00000103245	CIAO3	-0.48	9.41656E-05	0.000417097
ENSG00000163132	MSX1	0.30	9.50348E-05	0.000420854
ENSG00000267056	AC005336.1	-1.61	9.5127E-05	0.000421168
ENSG00000132359	RAP1GAP2	-0.35	9.5163E-05	0.000421235
ENSG00000101346	POFUT1	-0.36	9.54944E-05	0.000422608
ENSG00000173083	HPSE	0.47	9.58999E-05	0.000424309
ENSG00000123545	NDUFAF4	-0.54	9.62567E-05	0.000425793
ENSG00000179240	GVQW3	-0.83	9.63009E-05	0.000425809
ENSG00000130725	UBE2M	0.32	9.63029E-05	0.000425809
ENSG00000121578	B4GALT4	0.65	9.65845E-05	0.00042696
ENSG00000183386	FHL3	0.64	9.71076E-05	0.000429178
ENSG00000198018	ENTPD7	0.46	9.75958E-05	0.00043124
ENSG00000150403	TMCO3	0.35	9.7895E-05	0.000432467
ENSG00000140367	UBE2Q2	0.62	9.79461E-05	0.000432515
ENSG00000244716	BX679664.3	-0.28	9.79492E-05	0.000432515
ENSG00000156642	NPTN	0.29	9.80356E-05	0.000432801
ENSG00000102882	MAPK3	-0.44	9.8539E-05	0.000434928
ENSG00000165886	UBTD1	0.57	9.88451E-05	0.000436183
ENSG00000172046	USP19	-0.31	9.92997E-05	0.000438092
ENSG00000050130	JKAMP	0.34	9.96856E-05	0.000439698
ENSG00000198874	TYW1	0.50	9.98284E-05	0.00044023
ENSG00000148843	PDCD11	-0.27	0.000100033	0.000441038
ENSG00000049449	RCN1	0.29	0.000100239	0.000441848
ENSG00000131351	HAUS8	0.48	0.000101032	0.000445243
ENSG00000127364	TAS2R4	-3.27	0.000101288	0.000446275
ENSG00000134996	OSTF1	0.57	0.000101988	0.000449259
ENSG00000168938	PPIC	0.50	0.000102596	0.000451837
ENSG00000122861	PLAU	-0.34	0.000102968	0.000453378
ENSG00000179889	PDXDC1	0.28	0.000103547	0.000455825
ENSG00000144395	CCDC150	-0.69	0.000103756	0.000456647
ENSG00000166200	COPS2	0.36	0.000103904	0.000457198
ENSG00000119771	KLHL29	-0.47	0.000104057	0.00045777
ENSG00000171877	FRMD5	0.35	0.000105385	0.00046351
ENSG00000126804	ZBTB1	0.62	0.000105528	0.000464035
ENSG00000132000	PODNL1	2.00	0.000106512	0.000468261
ENSG00000083750	RRAGB	0.85	0.000106637	0.000468706

ENSG00000166860	ZBTB39	-0.54	0.000106842	0.000469506
ENSG00000167460	TPM4	0.25	0.000107024	0.0004702
ENSG00000132024	CC2D1A	-0.38	0.000107161	0.000470701
ENSG00000198729	PPP1R14C	0.42	0.000107208	0.000470803
ENSG00000167642	SPINT2	0.28	0.000107458	0.000471799
ENSG00000188993	LRRC66	2.80	0.000108602	0.000476717
ENSG00000223669	AL357033.2	1.66	0.000108883	0.000477843
ENSG00000149182	ARFGAP2	-0.38	0.00010902	0.00047834
ENSG00000198417	MT1F	1.15	0.000109123	0.000478662
ENSG00000115325	DOK1	0.49	0.000109141	0.000478662
ENSG00000076685	NT5C2	0.45	0.000109434	0.000479843
ENSG00000133985	TTC9	0.88	0.000110521	0.000484501
ENSG00000236924	AL162411.1	1.57	0.000110652	0.000484915
ENSG00000163482	STK36	-0.62	0.000110664	0.000484915
ENSG00000136048	DRAM1	-0.55	0.000111468	0.00048833
ENSG00000113597	TRAPPC13	1.22	0.000111504	0.000488383
ENSG00000083223	TUT7	0.50	0.000111655	0.000488936
ENSG00000166477	LEO1	0.42	0.000112212	0.000491267
ENSG00000125430	HS3ST3B1	1.18	0.000113227	0.000495604
ENSG00000129460	NGDN	0.43	0.000113378	0.000496155
ENSG00000015413	DPEP1	2.78	0.000113459	0.000496402
ENSG00000087095	NLK	-0.74	0.000113691	0.00049731
ENSG00000188021	UBQLN2	-0.49	0.00011376	0.000497452
ENSG00000205922	ONECUT3	-0.47	0.000113774	0.000497452
ENSG00000164167	LSM6	-0.43	0.000114165	0.000499055
ENSG00000240230	COX19	-0.54	0.000114329	0.000499661
ENSG00000159173	TNNI1	-2.03	0.000114961	0.000502314
ENSG00000215041	NEURL4	-0.39	0.00011499	0.00050233
ENSG00000167566	NCKAP5L	-0.60	0.000115041	0.000502444
ENSG00000168818	STX18	0.50	0.000116331	0.000507875
ENSG00000185267	CDNF	-2.47	0.000116335	0.000507875
ENSG00000021355	SERPINB1	-0.34	0.000116883	0.000510154
ENSG00000130787	HIP1R	-0.36	0.000117017	0.000510628
ENSG00000125107	CNOT1	-0.30	0.000117248	0.000511527
ENSG00000196505	GDAP2	0.58	0.000117523	0.000512613
ENSG00000161249	DMKN	-0.28	0.000118103	0.00051503
ENSG00000099849	RASSF7	-0.52	0.000118172	0.00051522
ENSG00000171861	MRM3	0.44	0.000118242	0.000515413
ENSG00000198455	ZXDB	-0.72	0.000118783	0.000517658
ENSG00000215421	ZNF407	0.86	0.00011979	0.000521934
ENSG00000110080	ST3GAL4	0.61	0.000119953	0.000522527
ENSG00000246363	LINC02458	-1.00	0.000120844	0.000526295
ENSG00000182326	C1S	1.37	0.000121325	0.000528233
ENSG00000108830	RND2	1.23	0.000121342	0.000528233
ENSG00000250697	AC010343.3	3.08	0.000121594	0.000529215
ENSG00000117523	PRRC2C	0.27	0.000121875	0.000530325

ENSG00000074964	ARHGEF10L	-0.60	0.000122641	0.000533542
ENSG00000145216	FIP1L1	0.43	0.000122702	0.000533693
ENSG00000112640	PPP2R5D	0.32	0.000122978	0.000534775
ENSG00000124313	IQSEC2	-0.53	0.000123188	0.000535571
ENSG00000253276	CCDC71L	0.54	0.000123499	0.000536809
ENSG00000108829	LRRC59	-0.22	0.000123775	0.000537891
ENSG00000241990	PRR34-AS1	0.93	0.000123979	0.000538659
ENSG00000151224	MAT1A	-1.32	0.000124482	0.000540727
ENSG00000172331	BPGM	0.55	0.000126067	0.000547496
ENSG00000169592	INO80E	0.32	0.000126693	0.000550092
ENSG00000169689	CENPX	0.33	0.000126877	0.000550771
ENSG00000107643	MAPK8	0.36	0.000126922	0.000550846
ENSG00000018610	CXorf56	0.46	0.000126949	0.000550846
ENSG00000184988	TMEM106A	1.70	0.000127164	0.000551661
ENSG00000089289	IGBP1	-0.44	0.00012727	0.000551999
ENSG00000158417	EIF5B	0.30	0.000127429	0.000552571
ENSG00000288096	AC109322.2	-6.45	0.00012798	0.000554841
ENSG00000120137	PANK3	-0.39	0.000128951	0.000558927
ENSG00000174796	THAP6	-0.68	0.000130037	0.000563512
ENSG00000128654	MTX2	0.36	0.000130434	0.000565108
ENSG00000169251	NMD3	0.33	0.000130556	0.000565483
ENSG00000255302	EID1	-0.31	0.000130576	0.000565483
ENSG00000182600	SNORC	-1.14	0.000130858	0.000566579
ENSG00000139211	AMIGO2	1.14	0.000132013	0.000571457
ENSG00000124541	RRP36	0.35	0.000132382	0.000572932
ENSG00000163808	KIF15	-0.70	0.000132556	0.000573557
ENSG00000069329	VPS35	0.31	0.000133017	0.000575427
ENSG00000197622	CDC42SE1	-0.33	0.000134222	0.000580516
ENSG00000125814	NAPB	0.54	0.000134975	0.000583649
ENSG00000149929	HIRIP3	0.34	0.000135556	0.000586035
ENSG00000141458	NPC1	0.29	0.000136348	0.000589331
ENSG00000070182	SPTB	0.73	0.000136405	0.00058945
ENSG00000134452	FBH1	-0.36	0.000136467	0.000589587
ENSG00000119318	RAD23B	0.23	0.000137518	0.000594001
ENSG00000157152	SYN2	-1.02	0.000137625	0.000594336
ENSG00000067533	RRP15	0.42	0.000138143	0.000596443
ENSG00000244560	AC004890.2	-1.69	0.000138316	0.000597062
ENSG00000137628	DDX60	0.80	0.000138601	0.000598162
ENSG00000078902	TOLLIP	0.40	0.000139063	0.000600026
ENSG00000090372	STRN4	-0.30	0.000139364	0.000601198
ENSG00000145979	TBC1D7	0.60	0.000139737	0.000602677
ENSG00000185010	F8	-1.70	0.000139991	0.000603641
ENSG00000205707	ETFRF1	-1.28	0.000140175	0.000604305
ENSG00000109270	LAMTOR3	0.49	0.000140238	0.000604448
ENSG00000169750	RAC3	0.61	0.000140315	0.000604647
ENSG00000054793	ATP9A	0.46	0.000140452	0.00060511

ENSG00000101421	CHMP4B	0.32	0.000140528	0.000605304
ENSG00000226833	AC092164.1	-2.31	0.000140946	0.000606977
ENSG00000188687	SLC4A5	-2.21	0.000141414	0.000608859
ENSG00000267123	SCAT1	1.03	0.000141861	0.000610653
ENSG00000100441	KHNYN	-0.37	0.000141972	0.000610998
ENSG00000065029	ZNF76	-0.41	0.000142263	0.000612119
ENSG00000198736	MSRB1	0.44	0.000142363	0.000612418
ENSG00000135698	MPHOSPH6	-0.40	0.000142806	0.000614194
ENSG00000118495	PLAGL1	-0.48	0.000142903	0.000614477
ENSG00000185504	FAAP100	-0.41	0.00014311	0.000615235
ENSG00000117335	CD46	-0.35	0.000143915	0.000618564
ENSG00000136450	SRSF1	-0.24	0.000144014	0.000618854
ENSG00000205269	TMEM170B	1.01	0.00014417	0.000619392
ENSG00000272913	AC009237.14	-2.06	0.000144201	0.000619392
ENSG00000205808	PLPP6	-1.02	0.000144735	0.000621421
ENSG00000117419	ERI3	-0.36	0.000144735	0.000621421
ENSG00000149289	ZC3H12C	0.61	0.000145969	0.000626482
ENSG00000177721	ANXA2R	1.80	0.000145977	0.000626482
ENSG00000116906	GNPAT	-0.39	0.00014734	0.000632199
ENSG00000258643	BCL2L2-PABP	2.51	0.000147498	0.000632741
ENSG00000186010	NDUFA13	0.39	0.000147602	0.000633049
ENSG00000174891	RSRC1	0.59	0.00014765	0.000633119
ENSG00000152133	GPATCH11	0.68	0.000148417	0.000636271
ENSG00000246082	NUDT16P1	-0.95	0.000148508	0.000636528
ENSG00000105607	GCDH	-0.56	0.000148555	0.000636591
ENSG00000173227	SYT12	0.86	0.000149451	0.000640295
ENSG00000132256	TRIM5	-0.62	0.000149729	0.000641349
ENSG00000146376	ARHGAP18	-0.34	0.000150245	0.000643419
ENSG00000163820	FYCO1	-0.48	0.000152595	0.000653343
ENSG00000254639	AC116021.1	1.96	0.000152734	0.0006538
ENSG00000237438	CECR7	-0.99	0.000152892	0.000654337
ENSG00000178980	SELENOW	0.57	0.000152981	0.000654578
ENSG00000143801	PSEN2	-0.65	0.000153378	0.000656004
ENSG00000127804	METTL16	0.36	0.00015338	0.000656004
ENSG00000008853	RHOBTB2	-0.64	0.000154362	0.000660063
ENSG00000147471	PLPBP	0.37	0.000154568	0.000660801
ENSG00000116455	WDR77	0.37	0.000154707	0.000661256
ENSG00000105991	HOXA1	0.98	0.00015493	0.000662065
ENSG00000105516	DBP	0.54	0.000155441	0.000664111
ENSG00000086300	SNX10	0.42	0.000155538	0.00066438
ENSG00000183060	LYSMD4	-0.81	0.000155886	0.000665728
ENSG00000214756	CSKMT	-1.04	0.000156345	0.000667543
ENSG00000100292	HMOX1	0.43	0.00015686	0.000669597
ENSG00000167536	DHRS13	-0.91	0.00015755	0.000672401
ENSG00000114650	SCAP	-0.36	0.000157799	0.000673321
ENSG00000175061	SNHG29	-0.25	0.000157849	0.000673389

ENSG00000175984	DENND2C	0.99	0.000158122	0.00067441
ENSG00000198948	MFAP3L	0.63	0.000159831	0.000681553
ENSG00000164919	COX6C	0.25	0.000160544	0.000684449
ENSG00000131375	CAPN7	0.64	0.000161571	0.00068868
ENSG00000125898	FAM110A	0.56	0.000161731	0.000689215
ENSG00000268812	AC004264.1	-1.61	0.000162087	0.000690586
ENSG00000174718	RESF1	0.44	0.000162934	0.000694049
ENSG00000107651	SEC23IP	0.31	0.000163208	0.000695065
ENSG00000168412	MTNR1A	6.40	0.000163399	0.000695733
ENSG00000151233	GXYLT1	-0.60	0.000163448	0.000695791
ENSG00000159788	RGS12	-0.61	0.000163496	0.00069585
ENSG00000031698	SARS1	0.26	0.000164048	0.000698052
ENSG00000197472	ZNF695	0.73	0.000165472	0.000703958
ENSG00000196236	XPNPEP3	0.40	0.000165646	0.000704494
ENSG00000167646	DNAAF3	0.47	0.000165668	0.000704494
ENSG00000178163	ZNF518B	-0.44	0.000166212	0.000706658
ENSG00000158773	USF1	0.40	0.000166259	0.000706709
ENSG00000153904	DDAH1	0.40	0.000166374	0.000707022
ENSG00000137806	NDUFAF1	0.48	0.000166404	0.000707022
ENSG00000246451	AL049840.2	-1.75	0.000166729	0.000708252
ENSG00000136883	KIF12	1.07	0.000167112	0.000709728
ENSG00000163710	PCOLCE2	0.61	0.00016757	0.000711524
ENSG00000175309	PHYKPL	-0.52	0.000168008	0.000713234
ENSG00000151062	CACNA2D4	-3.17	0.00016805	0.000713258
ENSG00000100360	IFT27	0.48	0.000169833	0.000720675
ENSG00000203485	INF2	-0.34	0.000170933	0.000725189
ENSG00000253930	TNFRSF10A-AS	-1.22	0.000171161	0.000726001
ENSG00000203999	LINC01270	-0.87	0.000171287	0.000726382
ENSG00000204315	FKBPL	0.57	0.000171541	0.000727306
ENSG00000233223	AC016876.2	0.96	0.000172106	0.000729544
ENSG00000146858	ZC3HAV1L	-0.97	0.000172157	0.000729606
ENSG00000237973	MTCO1P12	-0.52	0.000172282	0.000729984
ENSG00000232859	LYRM9	-1.89	0.000172329	0.000730028
ENSG00000134717	BTF3L4	-0.31	0.000172379	0.000730084
ENSG00000245849	RAD51-AS1	-0.95	0.000173113	0.000733029
ENSG00000279662	AC131649.2	-1.90	0.000173148	0.000733029
ENSG00000154642	C21orf91	-0.66	0.00017331	0.000733562
ENSG00000099385	BCL7C	0.72	0.000173493	0.00073418
ENSG00000127337	YEATS4	-0.59	0.000173712	0.000734951
ENSG00000229931	ATXN1-AS1	2.76	0.000174221	0.000736948
ENSG00000124172	ATP5F1E	0.24	0.000174882	0.000739589
ENSG00000144741	SLC25A26	-0.54	0.000174956	0.000739745
ENSG00000139083	ETV6	-0.58	0.000175285	0.000740979
ENSG00000176845	METRNL	0.40	0.000175703	0.000742549
ENSG00000128159	TUBGCP6	-0.39	0.00017573	0.000742549
ENSG00000169116	PARM1	-0.57	0.000176193	0.000744347

ENSG00000089123	TASP1	-0.77	0.000176607	0.000745941
ENSG00000176076	KCNE5	2.93	0.000177468	0.000749418
ENSG00000131944	FAAP24	0.87	0.000177564	0.000749665
ENSG00000170608	FOXA3	0.68	0.000178555	0.000753691
ENSG00000197587	DMBX1	-0.94	0.000178957	0.000755227
ENSG00000142677	IL22RA1	0.93	0.000179344	0.000756701
ENSG00000013583	HEBP1	-0.48	0.00017943	0.000756903
ENSG00000198105	ZNF248	-0.87	0.000179952	0.000758947
ENSG00000053918	KCNQ1	-0.96	0.000180788	0.00076231
ENSG00000228065	LINC01515	-0.96	0.000183276	0.000772639
ENSG00000213693	SEC14L1P1	1.77	0.000184296	0.000776778
ENSG00000160072	ATAD3B	-0.36	0.000185274	0.000780735
ENSG00000279207	AC015813.6	-0.66	0.000186935	0.000787566
ENSG00000231607	DLEU2	-0.49	0.000187617	0.000790133
ENSG00000256538	AC046130.2	4.60	0.000187623	0.000790133
ENSG00000173442	EHBP1L1	0.26	0.000188022	0.000791645
ENSG00000162063	CCNF	0.30	0.000188909	0.000795215
ENSG00000227619	AL391056.1	-6.35	0.000189665	0.000798228
ENSG00000105204	DYRK1B	-1.65	0.000190243	0.000800495
ENSG00000103855	CD276	0.32	0.000190639	0.000801992
ENSG00000148498	PARD3	0.35	0.000190756	0.000802313
ENSG00000259768	AC004943.2	-0.76	0.000191105	0.000803615
ENSG00000137404	NRM	-0.76	0.000191159	0.000803671
ENSG00000226964	RHEBP2	0.42	0.000191337	0.000804252
ENSG00000140057	AK7	-1.87	0.000193571	0.000813468
ENSG00000126432	PRDX5	0.62	0.000193744	0.000814026
ENSG00000103005	USB1	0.33	0.000195067	0.00081925
ENSG00000184545	DUSP8	0.64	0.000195069	0.00081925
ENSG00000244198	ARHGEF35-AS	-1.12	0.000195313	0.000819989
ENSG00000055044	NOP58	0.28	0.000195327	0.000819989
ENSG00000103269	RHBDL1	-1.05	0.000195556	0.000820776
ENSG00000256916	AP000851.1	1.91	0.000195759	0.000821456
ENSG00000100926	TM9SF1	-0.69	0.000196651	0.000825029
ENSG00000243927	MRPS6	-0.57	0.000197034	0.000826459
ENSG00000205981	DNAJC19	-0.58	0.000197389	0.000827778
ENSG00000146909	NOM1	-0.36	0.000197817	0.000829397
ENSG00000128274	A4GALT	0.57	0.000197997	0.000829976
ENSG00000116691	MIIP	0.45	0.000198329	0.000831197
ENSG00000204209	DAXX	-0.34	0.000198686	0.000832505
ENSG00000133895	MEN1	-0.33	0.000198725	0.000832505
ENSG00000102967	DHODH	-0.51	0.000198994	0.000833461
ENSG00000185728	YTHDF3	0.38	0.000200743	0.00084061
ENSG00000205339	IPO7	-0.25	0.000201007	0.000841538
ENSG00000259865	AL390728.6	-0.95	0.000202478	0.00084752
ENSG00000182973	CNOT10	-0.44	0.00020301	0.000849421
ENSG00000131462	TUBG1	-0.31	0.000203017	0.000849421

ENSG00000174886	NDUFA11	0.38	0.000203907	0.000852965
ENSG00000105220	GPI	-0.68	0.000204273	0.00085432
ENSG00000197763	TXNRD3	-0.62	0.000204518	0.000855162
ENSG00000137331	IER3	0.26	0.000205759	0.00086017
ENSG00000213963	AC019080.1	-1.36	0.000205805	0.00086017
ENSG00000153140	CETN3	0.42	0.000205844	0.00086017
ENSG00000241322	CDRT1	-3.64	0.000206973	0.000864705
ENSG00000116171	SCP2	0.37	0.000207891	0.000868361
ENSG00000130731	METTL26	-0.51	0.000208593	0.000871109
ENSG00000213588	ZBTB9	-0.44	0.000208706	0.0008714
ENSG00000176783	RUFY1	-0.51	0.000209878	0.000876111
ENSG00000133460	SLC2A11	-0.66	0.000211872	0.000884249
ENSG00000138400	MDH1B	-1.49	0.000211935	0.000884331
ENSG00000152457	DCLRE1C	-0.69	0.000212688	0.000887284
ENSG00000267940	AC022762.2	-2.97	0.000214751	0.000895707
ENSG00000163961	RNF168	0.39	0.000215216	0.000897458
ENSG00000163378	EOGT	0.57	0.000215627	0.000898937
ENSG00000173557	FAM166C	0.93	0.00021566	0.000898937
ENSG00000136243	NUP42	0.51	0.000218251	0.000909545
ENSG00000121931	LRIF1	0.47	0.000218508	0.000910427
ENSG00000088832	FKBP1A	-0.24	0.000219157	0.000912943
ENSG00000178761	FAM219B	-0.68	0.000219214	0.000912988
ENSG00000138600	SPPL2A	0.33	0.000219663	0.000914666
ENSG00000130772	MED18	0.45	0.000220176	0.000916612
ENSG00000150594	ADRA2A	3.61	0.000221003	0.000919864
ENSG00000286214	AUXG0100005[illegible]	-1.96	0.000221715	0.000922638
ENSG00000138798	EGF	2.60	0.000222415	0.000925358
ENSG00000117153	KLHL12	-0.38	0.0002229	0.000927183
ENSG00000139117	CPNE8	0.63	0.00022301	0.000927446
ENSG00000215788	TNFRSF25	-0.59	0.000224512	0.000933499
ENSG00000120318	ARAP3	-0.57	0.000224906	0.000934945
ENSG00000141013	GAS8	-0.48	0.000225004	0.000935091
ENSG00000285979	AC009090.6	-1.07	0.000225035	0.000935091
ENSG00000136718	IMP4	-0.28	0.000226065	0.000939121
ENSG00000162735	PEX19	-0.39	0.000226099	0.000939121
ENSG00000070718	AP3M2	-0.42	0.000226986	0.000942613
ENSG00000170322	NFRKB	0.45	0.000227472	0.000944435
ENSG00000073861	TBX21	6.34	0.00022864	0.000949087
ENSG00000116991	SIPA1L2	-0.47	0.000230035	0.000954678
ENSG00000004700	RECQL	0.56	0.00023058	0.000956741
ENSG00000167074	TEF	-0.50	0.000230976	0.000958187
ENSG00000126464	PRR12	-0.42	0.000232274	0.000963374
ENSG00000163159	VPS72	0.39	0.000233194	0.000966988
ENSG00000184903	IMMP2L	-0.94	0.000233342	0.0009674
ENSG00000148468	FAM171A1	-0.36	0.000233421	0.000967525
ENSG00000052841	TTC17	-0.45	0.000233677	0.000968387

ENSG00000133059	DSTYK	0.49	0.000234051	0.000969739
ENSG00000180329	CCDC43	0.47	0.000234327	0.000970681
ENSG00000135250	SRPK2	0.38	0.000235437	0.000975078
ENSG00000151131	C12orf45	-0.55	0.00023766	0.000984077
ENSG00000184678	H2BC21	1.49	0.000237961	0.000985122
ENSG00000164134	NAA15	0.34	0.00023827	0.000986194
ENSG00000254788	CKLF-CMTM1	2.02	0.000238319	0.000986194
ENSG00000134762	DSC3	0.50	0.000238737	0.000987721
ENSG00000164985	PSIP1	0.25	0.000240925	0.000996568
ENSG00000224397	PELATON	-3.95	0.00024104	0.000996838
ENSG00000079277	MKNK1	-0.48	0.000241378	0.000998028
ENSG00000149716	LTO1	-0.56	0.000241834	0.000999709
ENSG00000141968	VAV1	0.41	0.000242299	0.001001422
ENSG00000144040	SFXN5	0.62	0.000242536	0.001002194
ENSG00000003509	NDUFAF7	0.51	0.000243431	0.001005685
ENSG00000174804	FZD4	-0.72	0.000244309	0.001009105
ENSG00000161048	NAPEPLD	-0.69	0.000244536	0.001009833
ENSG00000168779	SHOX2	0.57	0.000246415	0.001017384
ENSG00000171631	P2RY6	3.54	0.000247019	0.001019666
ENSG00000189223	PAX8-AS1	-0.38	0.000247279	0.001020529
ENSG00000162757	C1orf74	-0.74	0.000247808	0.0010225
ENSG00000113558	SKP1	0.25	0.000248093	0.001023466
ENSG00000174165	ZDHHC24	-0.70	0.000248991	0.001026961
ENSG00000109670	FBXW7	0.47	0.000249584	0.001029195
ENSG00000163879	DNALI1	2.36	0.000249945	0.00103047
ENSG00000198830	HMGN2	-0.28	0.000250003	0.001030496
ENSG00000237978	KCNMB2-AS1	0.95	0.000250567	0.001032607
ENSG00000181027	FKRP	-0.61	0.000251235	0.00103515
ENSG00000109381	ELF2	0.58	0.000251363	0.001035463
ENSG00000083844	ZNF264	0.40	0.000251615	0.001036286
ENSG00000100749	VRK1	-0.49	0.000251673	0.001036313
ENSG00000148356	LRSAM1	-0.52	0.000251793	0.001036593
ENSG00000138279	ANXA7	0.29	0.000251887	0.001036767
ENSG00000188372	ZP3	-0.91	0.000252381	0.001038586
ENSG00000054392	HHAT	-1.21	0.000252605	0.001039297
ENSG00000112144	CILK1	-0.59	0.000255376	0.00105048
ENSG00000166295	ANAPC16	-0.29	0.000255906	0.001052446
ENSG00000111696	NT5DC3	-0.46	0.000257194	0.001057522
ENSG00000075188	NUP37	-0.36	0.00025757	0.001058703
ENSG00000048649	RSF1	0.40	0.000257587	0.001058703
ENSG00000227855	DPY19L2P3	-1.45	0.000258047	0.001060377
ENSG00000054282	SDCCAG8	0.57	0.000258659	0.001062657
ENSG00000229989	MIR181A1HG	6.25	0.000258708	0.001062657
ENSG00000162775	RBM15	0.39	0.00025927	0.001064745
ENSG00000103266	STUB1	-0.39	0.000260249	0.001068548
ENSG00000148248	SURF4	0.24	0.000260867	0.001070865

ENSG00000174007	CEP19	1.04	0.000261114	0.001071658
ENSG00000159128	IFNGR2	-0.45	0.000262198	0.001075845
ENSG00000068001	HYAL2	0.37	0.000262241	0.001075845
ENSG00000016864	GLT8D1	-0.48	0.0002629	0.001078226
ENSG00000141076	UTP4	-0.29	0.000262929	0.001078226
ENSG00000108055	SMC3	0.29	0.000264178	0.001083122
ENSG00000183186	C2CD4C	0.59	0.000264306	0.001083426
ENSG00000179010	MRFAP1	0.21	0.000265075	0.001086358
ENSG00000138587	MNS1	-1.74	0.000265524	0.001087973
ENSG00000249476	AC008467.1	2.17	0.000265808	0.001088914
ENSG00000101076	HNF4A	0.95	0.000266202	0.001090305
ENSG00000187109	NAP1L1	-0.25	0.000266273	0.001090372
ENSG00000205464	ATP6AP1L	-0.74	0.000266571	0.001091368
ENSG00000111664	GNB3	-1.12	0.000266826	0.001092193
ENSG00000118620	ZNF430	0.81	0.00026715	0.001093295
ENSG00000116586	LAMTOR2	-0.41	0.000268408	0.001098219
ENSG00000101442	ACTR5	-0.43	0.000268505	0.001098389
ENSG00000165730	STOX1	-1.25	0.000269222	0.001101099
ENSG00000236213	AC006369.1	4.47	0.000270146	0.001104649
ENSG00000142864	SERBP1	0.22	0.000271029	0.001108034
ENSG00000087884	AAMDC	0.86	0.00027131	0.001108959
ENSG00000187266	EPOR	0.52	0.000271665	0.001110182
ENSG00000177548	RABEP2	0.67	0.000271786	0.001110447
ENSG00000242259	C22orf39	-0.54	0.000273003	0.001115194
ENSG00000112218	GPR63	-0.68	0.000275339	0.001124317
ENSG00000126970	ZC4H2	-0.95	0.000275349	0.001124317
ENSG00000196159	FAT4	1.24	0.00027731	0.001132096
ENSG00000110719	TCIRG1	-0.44	0.000277863	0.001134119
ENSG00000163319	MRPS18C	0.40	0.000279186	0.001139288
ENSG00000140525	FANCI	-0.31	0.000279774	0.001141456
ENSG00000112031	MTRF1L	0.65	0.00027991	0.001141777
ENSG00000143479	DYRK3	0.56	0.000280943	0.001145755
ENSG00000131788	PIAS3	-0.47	0.000282775	0.001152992
ENSG00000147403	RPL10	-0.24	0.000282972	0.001153563
ENSG00000135870	RC3H1	0.40	0.000285065	0.001161857
ENSG00000197006	METTL9	-0.40	0.000287183	0.00117018
ENSG00000111530	CAND1	0.30	0.000287224	0.00117018
ENSG00000213337	ANKRD39	0.54	0.000287389	0.001170613
ENSG00000059728	MXD1	0.76	0.000287521	0.001170916
ENSG00000141642	ELAC1	-0.90	0.000287711	0.001171449
ENSG00000185972	CCIN	3.95	0.000287843	0.001171749
ENSG00000127445	PIN1	0.32	0.000288476	0.001174088
ENSG00000138032	PPM1B	0.45	0.000288621	0.001174436
ENSG00000105373	NOP53	-0.26	0.000288722	0.001174609
ENSG00000147883	CDKN2B	0.70	0.000289706	0.001178372
ENSG00000125445	MRPS7	0.29	0.000290069	0.001179608

ENSG00000150977	RILPL2	0.54	0.000290969	0.001183032
ENSG00000110076	NRXN2	1.55	0.00029144	0.001184703
ENSG00000100462	PRMT5	0.23	0.000291635	0.001185084
ENSG00000105127	AKAP8	0.36	0.000291652	0.001185084
ENSG00000167985	SDHAF2	-0.72	0.00029203	0.001186271
ENSG00000072849	DERL2	0.35	0.000292063	0.001186271
ENSG00000149231	CCDC82	0.49	0.000292695	0.001188596
ENSG00000164136	IL15	1.17	0.000293707	0.001192323
ENSG00000100815	TRIP11	0.48	0.000293731	0.001192323
ENSG00000145416	MARCHF1	-1.08	0.000295013	0.001197282
ENSG00000147676	MAL2	-0.28	0.000297909	0.001208791
ENSG00000184292	TACSTD2	3.09	0.000299674	0.001215704
ENSG00000135116	HRK	-0.80	0.000299811	0.001216015
ENSG00000092847	AGO1	0.28	0.000300311	0.001217795
ENSG00000023697	DERA	-0.48	0.000301012	0.001220391
ENSG00000143842	SOX13	-0.51	0.000301134	0.001220637
ENSG00000247240	UBL7-AS1	0.85	0.000301521	0.00122196
ENSG00000224531	SMIM13	0.40	0.000301725	0.00122254
ENSG00000136052	SLC41A2	0.66	0.000303172	0.001228043
ENSG00000054598	FOXC1	-0.43	0.000303206	0.001228043
ENSG00000180992	MRPL14	0.36	0.000303377	0.001228488
ENSG00000144713	RPL32	-0.21	0.000304056	0.001230988
ENSG00000224411	HSP90AA2P	1.10	0.000304926	0.001234058
ENSG00000169062	UPF3A	0.40	0.000304938	0.001234058
ENSG00000152102	FAM168B	0.25	0.000305024	0.001234159
ENSG00000186716	BCR	-0.39	0.00030766	0.001244571
ENSG00000136021	SCYL2	0.40	0.000309806	0.001252776
ENSG00000234420	ZNF37BP	-0.69	0.000309814	0.001252776
ENSG00000155189	AGPAT5	-0.29	0.000312563	0.001263637
ENSG00000104522	TSTA3	-0.31	0.000312714	0.001263995
ENSG00000121067	SPOP	0.29	0.000313819	0.001268204
ENSG00000136425	CIB2	0.54	0.000314425	0.001270396
ENSG00000107960	STN1	0.39	0.000314795	0.001271633
ENSG00000174233	ADCY6	0.38	0.000315401	0.001273824
ENSG00000105325	FZR1	0.30	0.00031735	0.001281439
ENSG00000279133	AC018628.1	-2.81	0.000318301	0.001285019
ENSG00000261239	ANKRD26P1	2.79	0.000318645	0.001286147
ENSG00000101294	HM13	-0.26	0.000318755	0.001286332
ENSG00000163702	IL17RC	0.57	0.000319898	0.001290686
ENSG00000106100	NOD1	-0.80	0.00032021	0.001291528
ENSG00000231789	PIK3CD-AS2	0.68	0.000320236	0.001291528
ENSG00000140988	RPS2	-0.32	0.000320513	0.001292385
ENSG00000139644	TMBIM6	-0.22	0.000320584	0.001292411
ENSG00000177663	IL17RA	-0.43	0.000320756	0.001292733
ENSG00000121053	EPX	-2.44	0.000320793	0.001292733
ENSG00000135336	ORC3	-0.49	0.000321443	0.001295089

ENSG00000142494	SLC47A1	3.24	0.000321628	0.001295577
ENSG00000088247	KHSRP	-0.22	0.000323341	0.001302214
ENSG00000241627	UBQLN4P1	-2.20	0.000323975	0.001304505
ENSG00000116288	PARK7	-0.23	0.00032446	0.001306196
ENSG00000196776	CD47	0.38	0.00032457	0.001306375
ENSG00000104763	ASAH1	0.33	0.000325312	0.001309097
ENSG00000259953	AL138756.1	-1.23	0.000325428	0.001309303
ENSG00000108091	CCDC6	-0.33	0.000325653	0.001309943
ENSG00000168264	IRF2BP2	-0.39	0.000327543	0.001317282
ENSG00000117758	STX12	0.45	0.000327847	0.001318238
ENSG00000023287	RB1CC1	0.47	0.00032884	0.001321965
ENSG00000111850	SMIM8	-1.01	0.000329842	0.001325485
ENSG00000129538	RNASE1	1.10	0.000329848	0.001325485
ENSG00000066379	ZNRD1	-0.51	0.000330823	0.00132897
ENSG00000164329	TENT2	-0.49	0.000330904	0.00132897
ENSG00000109390	NDUFC1	0.34	0.000330914	0.00132897
ENSG00000186687	LYRM7	-0.58	0.000331185	0.001329789
ENSG00000107554	DNMBP	0.26	0.000331569	0.001331066
ENSG00000179271	GADD45GIP1	0.32	0.000331889	0.001332083
ENSG00000279483	AC090498.1	-0.78	0.000332491	0.001334232
ENSG00000172273	HINFP	-0.49	0.000336024	0.001348138
ENSG00000089157	RPLP0	-0.28	0.000336335	0.001349116
ENSG00000126005	MMP24OS	-0.48	0.000337724	0.001354415
ENSG00000204175	GPRIN2	1.90	0.000338806	0.001358481
ENSG00000225137	DYNC1I2P1	1.43	0.000339788	0.001362146
ENSG00000076513	ANKRD13A	-0.50	0.00034307	0.001375027
ENSG00000139531	SUOX	0.48	0.000344376	0.001379988
ENSG00000136010	ALDH1L2	-1.29	0.000345858	0.001385649
ENSG00000113649	TCERG1	0.30	0.000346511	0.001387986
ENSG00000185800	DMWD	-0.46	0.000347123	0.001390158
ENSG00000140718	FTO	0.34	0.000347394	0.001390968
ENSG00000138162	TACC2	-0.37	0.000347491	0.001391075
ENSG00000174547	MRPL11	-0.30	0.000348853	0.001396247
ENSG00000198142	SOWAHC	0.41	0.000348932	0.001396286
ENSG00000185127	C6orf120	-0.57	0.000350679	0.001402997
ENSG00000170802	FOXN2	0.50	0.000351104	0.001404415
ENSG00000004487	KDM1A	0.23	0.000352148	0.001408196
ENSG00000101150	TPD52L2	-0.24	0.00035219	0.001408196
ENSG00000162885	B3GALNT2	-0.46	0.00035315	0.001411755
ENSG00000230409	TCEA1P2	-0.56	0.00035381	0.001414111
ENSG00000157613	CREB3L1	1.95	0.000353914	0.001414242
ENSG00000277363	SRCIN1	0.57	0.000354195	0.001415083
ENSG00000178691	SUZ12	0.30	0.00035564	0.001420458
ENSG00000147804	SLC39A4	0.40	0.000355682	0.001420458
ENSG00000125354	SEPTIN6	0.55	0.000355963	0.001421296
ENSG00000134313	KIDINS220	0.39	0.000356204	0.001421784

ENSG00000111644	ACRBP	-3.86	0.000356227	0.001421784
ENSG00000225177	AL590617.2	-1.22	0.000356322	0.001421877
ENSG00000157020	SEC13	0.29	0.000357355	0.001425717
ENSG00000151445	VIPAS39	-0.56	0.000359766	0.001435048
ENSG00000106305	AIMP2	-0.34	0.000359888	0.001435248
ENSG00000139163	ETNK1	0.29	0.000360422	0.001437093
ENSG00000141522	ARHGDIA	0.26	0.000360733	0.001438047
ENSG00000143793	C1orf35	-0.42	0.000361262	0.001439644
ENSG00000130638	ATXN10	0.28	0.000361278	0.001439644
ENSG00000130479	MAP1S	0.36	0.00036293	0.00144594
ENSG00000108784	NAGLU	-0.57	0.00036383	0.001449237
ENSG00000276529	AP001505.1	-1.17	0.000364414	0.001451272
ENSG00000198467	TPM2	0.24	0.000365343	0.001454686
ENSG00000100479	POLE2	-0.58	0.000365789	0.001456171
ENSG00000167202	TBC1D2B	-0.44	0.000365924	0.001456416
ENSG00000188001	TPRG1	1.81	0.000366151	0.001457033
ENSG00000115750	TAF1B	0.53	0.000366496	0.001458117
ENSG00000115556	PLCD4	-0.70	0.000368529	0.001465913
ENSG00000112796	ENPP5	0.72	0.000371073	0.001475737
ENSG00000196972	SMIM10L2B	-2.77	0.00037296	0.001482668
ENSG00000204469	PRRC2A	-0.21	0.000372964	0.001482668
ENSG00000158882	TOMM40L	0.52	0.000374164	0.001487144
ENSG00000143368	SF3B4	-0.31	0.000377885	0.001501635
ENSG00000157778	PSMG3	0.34	0.000378468	0.001503652
ENSG00000165322	ARHGAP12	0.30	0.000380486	0.001511295
ENSG00000103642	LACTB	0.67	0.000380542	0.001511295
ENSG00000116560	SFPQ	0.21	0.00038077	0.001511899
ENSG00000089022	MAPKAPK5	-0.42	0.000381021	0.001512587
ENSG00000280832	GSEC	-1.22	0.000381095	0.001512587
ENSG00000257086	AP001453.3	0.89	0.000381377	0.001513408
ENSG00000170085	SIMC1	-0.67	0.000381659	0.001514228
ENSG00000225614	ZNF469	-0.77	0.000382226	0.001516177
ENSG00000114302	PRKAR2A	-0.37	0.000382626	0.001517461
ENSG00000186862	PDZD7	0.68	0.000382788	0.001517803
ENSG00000101182	PSMA7	0.21	0.000385139	0.001526821
ENSG00000150779	TIMM8B	0.32	0.000386845	0.001533283
ENSG00000144369	FAM171B	-0.61	0.000386977	0.001533502
ENSG00000177181	RIMKLA	-0.69	0.000388005	0.001537269
ENSG00000198668	CALM1	0.22	0.000388705	0.001539741
ENSG00000170266	GLB1	-0.38	0.000388816	0.001539873
ENSG00000101158	NELFCD	-0.28	0.000389329	0.001541601
ENSG00000196422	PPP1R26	0.32	0.000391039	0.001548065
ENSG00000143952	VPS54	0.45	0.00039114	0.001548158
ENSG00000087116	ADAMTS2	0.81	0.000391434	0.001549016
ENSG00000203722	RAET1G	2.17	0.000391992	0.001550919
ENSG00000123338	NCKAP1L	2.20	0.000392099	0.001551035

ENSG00000188313	PLSCR1	-0.51	0.000392727	0.001553213
ENSG00000100554	ATP6V1D	0.37	0.000393209	0.001554812
ENSG00000185100	ADSS1	-0.97	0.00039349	0.001555614
ENSG00000067900	ROCK1	0.40	0.000394219	0.001558188
ENSG00000185088	RPS27L	-0.36	0.000394626	0.001559489
ENSG00000109323	MANBA	-0.47	0.000395207	0.001561477
ENSG00000102984	ZNF821	1.19	0.000396836	0.001567602
ENSG00000178502	KLHL11	0.65	0.000397701	0.001570712
ENSG00000169504	CLIC4	-0.65	0.000397872	0.001571078
ENSG00000099974	DDTL	-1.07	0.000399216	0.001576071
ENSG00000101638	ST8SIA5	5.59	0.00039963	0.001577394
ENSG00000039650	PNKP	-0.37	0.000401359	0.00158391
ENSG00000206573	THUMPD3-AS1	-0.59	0.000401592	0.001584515
ENSG00000155868	MED7	0.69	0.000402253	0.001586809
ENSG00000138685	FGF2	-0.61	0.000405299	0.001598509
ENSG00000106266	SNX8	0.34	0.000405717	0.001599845
ENSG00000113615	SEC24A	0.43	0.000406265	0.001601691
ENSG00000146090	RASGEF1C	-1.93	0.000407499	0.001606239
ENSG00000100028	SNRPD3	-0.36	0.000407739	0.001606868
ENSG00000141441	GAREM1	-0.75	0.000407996	0.001607562
ENSG00000182774	RPS17	-0.22	0.000408222	0.001608137
ENSG00000168792	ABHD15	-0.64	0.000408413	0.001608572
ENSG00000095564	BTAF1	-0.40	0.000409096	0.001610947
ENSG00000102471	NDFIP2	0.34	0.000410044	0.001614363
ENSG00000123219	CENPK	-0.63	0.000411736	0.001620238
ENSG00000231625	SLC47A1P2	2.43	0.000411779	0.001620238
ENSG00000169696	ASPSCR1	-0.44	0.00041178	0.001620238
ENSG00000171126	KCNG3	1.37	0.000412631	0.001623269
ENSG00000260948	AL390195.2	-1.65	0.000412871	0.001623894
ENSG00000173757	STAT5B	-0.95	0.00041518	0.001632654
ENSG00000154839	SKA1	-0.34	0.000415554	0.001633806
ENSG00000132275	RRP8	-0.61	0.000415833	0.001634582
ENSG00000177359	AC024940.1	0.72	0.00041698	0.001638768
ENSG00000116874	WARS2	-0.85	0.000417246	0.001639492
ENSG00000136490	LIMD2	0.80	0.000418043	0.001642166
ENSG00000150556	LYPD6B	-0.97	0.000418091	0.001642166
ENSG00000167173	C15orf39	-0.43	0.000418194	0.001642249
ENSG00000079313	REXO1	-0.37	0.000418936	0.001644839
ENSG00000198130	HIBCH	0.49	0.000419287	0.001645893
ENSG00000125378	BMP4	0.33	0.000419847	0.001647772
ENSG00000163545	NUAK2	0.66	0.000420013	0.0016481
ENSG00000176209	SMIM19	-0.71	0.000420315	0.001648961
ENSG00000124228	DDX27	0.32	0.000420656	0.001649972
ENSG00000178921	PFAS	-0.37	0.000420852	0.001650418
ENSG00000109220	CHIC2	0.70	0.000424858	0.001665801
ENSG00000117791	MTARC2	-0.61	0.00042539	0.001667562

ENSG00000198087	CD2AP	0.39	0.000426121	0.001670099
ENSG00000168427	KLHL30	2.10	0.000428791	0.001680235
ENSG00000082438	COBLL1	-1.06	0.000429228	0.001681618
ENSG00000154217	PITPNC1	0.51	0.000429782	0.001683461
ENSG00000263004	AC007114.1	-1.58	0.000430301	0.001685165
ENSG00000158555	GDPD5	-0.56	0.000430736	0.001686538
ENSG00000110274	CEP164	-0.50	0.000431043	0.001687244
ENSG00000259803	SLC22A31	-0.51	0.000431085	0.001687244
ENSG00000148735	PLEKHS1	4.37	0.000432343	0.001691836
ENSG00000176624	MEX3C	0.34	0.000432543	0.001692288
ENSG00000112996	MRPS30	-0.34	0.000432907	0.001693381
ENSG00000280924	LINC00628	6.16	0.000433746	0.001696331
ENSG00000187908	DMBT1	2.53	0.000435322	0.00170188
ENSG00000164050	PLXNB1	-0.51	0.000435335	0.00170188
ENSG00000187325	TAF9B	-0.54	0.000435457	0.001701994
ENSG00000100836	PABPN1	0.26	0.000435534	0.001701994
ENSG00000158457	TSPAN33	0.46	0.000435926	0.001703194
ENSG00000170835	CEL	-2.05	0.000436018	0.001703219
ENSG00000171150	SOCS5	0.57	0.000436603	0.00170517
ENSG00000203667	COX20	0.54	0.000437892	0.001709872
ENSG00000183520	UTP11	0.34	0.000439487	0.001715764
ENSG00000236859	NIFK-AS1	-1.31	0.000441488	0.001723243
ENSG00000177169	ULK1	-0.49	0.000442266	0.00172594
ENSG00000090238	YPEL3	0.89	0.00044508	0.001736584
ENSG00000120526	NUDCD1	0.36	0.000445471	0.001737772
ENSG00000185009	AP3M1	0.31	0.000445795	0.001738694
ENSG00000186765	FSCN2	1.04	0.000448386	0.001748461
ENSG00000269837	IPO5P1	-1.62	0.000448605	0.001748746
ENSG00000120437	ACAT2	-0.41	0.000448634	0.001748746
ENSG00000244558	KCNK15-AS1	2.00	0.000448969	0.001749709
ENSG00000184557	SOCS3	0.41	0.000451729	0.001760124
ENSG00000147044	CASK	-0.43	0.000451929	0.001760559
ENSG00000011638	TMEM159	0.44	0.000452615	0.001762889
ENSG00000157450	RNF111	0.44	0.000453121	0.001764201
ENSG00000135898	GPR55	3.83	0.000453128	0.001764201
ENSG00000021300	PLEKHB1	0.39	0.000454738	0.001770125
ENSG00000117362	APH1A	0.26	0.000455503	0.001772761
ENSG00000127314	RAP1B	0.33	0.000456232	0.001775249
ENSG00000164466	SFXN1	0.26	0.000459702	0.001788404
ENSG00000148702	HABP2	4.54	0.0004609	0.001792719
ENSG00000114439	BBX	0.38	0.000463448	0.001802279
ENSG00000008394	MGST1	-0.31	0.000465721	0.001810766
ENSG00000185670	ZBTB3	0.79	0.000470075	0.001827341
ENSG00000159593	NAE1	-0.31	0.000470462	0.001828489
ENSG00000161847	RAVER1	-0.39	0.000471946	0.0018339
ENSG00000157741	UBN2	-0.52	0.000472158	0.001834368

ENSG00000136754	ABI1	0.41	0.000472682	0.001836046
ENSG00000164073	MFSD8	0.53	0.000476624	0.001851001
ENSG00000251059	PRKG2-AS1	6.09	0.00047716	0.001852721
ENSG00000181396	OGFOD3	0.32	0.000478249	0.00185659
ENSG00000180537	RNF182	0.56	0.000479215	0.001859978
ENSG00000164077	MON1A	-0.61	0.000480898	0.001865947
ENSG00000147592	LACTB2	-0.51	0.000480939	0.001865947
ENSG00000272338	AC067838.1	-1.32	0.000481542	0.001867922
ENSG00000041988	THAP3	0.48	0.000482333	0.001870628
ENSG00000132170	PPARG	0.47	0.000483956	0.001876561
ENSG00000121716	PILRB	0.53	0.000484387	0.001877869
ENSG00000169668	BCRP2	1.96	0.000484938	0.001879641
ENSG00000197620	EOLA1	0.69	0.000489346	0.001896357
ENSG00000174839	DENND6A	0.37	0.000489759	0.001897592
ENSG00000257390	AC023055.1	1.13	0.000491236	0.001902946
ENSG00000133026	MYH10	0.31	0.000492778	0.001908549
ENSG00000125863	MKKS	0.39	0.000493515	0.00191094
ENSG00000064655	EYA2	-4.38	0.000493675	0.00191094
ENSG00000137770	CTDSPL2	0.33	0.000493682	0.00191094
ENSG00000266964	FXYD1	1.26	0.000494783	0.001914833
ENSG00000117643	MAN1C1	1.32	0.000495679	0.001917929
ENSG00000115540	MOB4	-0.48	0.0004973	0.001923768
ENSG00000104915	STX10	0.31	0.00049738	0.001923768
ENSG00000198324	PHETA1	0.61	0.000498148	0.001926365
ENSG00000028310	BRD9	0.35	0.000499121	0.001929753
ENSG00000101084	RAB5IF	0.30	0.000499229	0.0019298
ENSG00000080603	SRCAP	-0.82	0.000499528	0.001930581
ENSG00000153015	CWC27	0.44	0.000500231	0.001932926
ENSG00000184831	APOO	0.50	0.000502215	0.001940219
ENSG00000178694	NSUN3	0.63	0.000502568	0.001941206
ENSG00000025156	HSF2	0.47	0.000503561	0.001944668
ENSG00000130589	HELZ2	-0.40	0.000506105	0.001954112
ENSG00000145391	SETD7	-0.32	0.000506876	0.001956712
ENSG00000189180	ZNF33A	-0.50	0.000507447	0.001958539
ENSG00000103152	MPG	-0.67	0.00050867	0.001962883
ENSG00000106526	ACTR3C	2.52	0.000508933	0.001963519
ENSG00000145002	FAM86B2	-3.02	0.000513135	0.001979346
ENSG00000279863	AC069547.1	-3.79	0.000515657	0.001988693
ENSG00000274605	PCCA-DT	0.77	0.000517957	0.001996834
ENSG00000169371	SNUPN	0.41	0.000517968	0.001996834
ENSG00000105443	CYTH2	0.28	0.000518152	0.001996926
ENSG00000225975	LINC01534	2.12	0.000518191	0.001996926
ENSG00000101290	CDS2	0.27	0.000519471	0.002001474
ENSG00000172932	ANKRD13D	0.40	0.000521407	0.002008546
ENSG00000007392	LUC7L	0.47	0.000522098	0.002010821
ENSG00000260807	CEROX1	0.90	0.00052246	0.002011826

ENSG00000225791	TRAM2-AS1	-1.36	0.00052337	0.002014944
ENSG00000088002	SULT2B1	1.16	0.000527079	0.002028835
ENSG00000113141	IK	0.27	0.000527375	0.002029404
ENSG00000186567	CEACAM19	-0.89	0.00052743	0.002029404
ENSG00000127415	IDUA	-0.90	0.000528366	0.002032614
ENSG00000106355	LSM5	0.30	0.000528696	0.002033493
ENSG00000168765	GSTM4	0.56	0.00052897	0.002034156
ENSG00000172339	ALG14	0.59	0.000529485	0.002035746
ENSG00000168090	COPS6	0.26	0.000529844	0.002036734
ENSG00000113240	CLK4	0.68	0.000533088	0.00204881
ENSG00000146066	HIGD2A	0.29	0.000533311	0.002049274
ENSG00000148019	CEP78	-0.41	0.000533605	0.002050012
ENSG00000107438	PDLIM1	-0.39	0.000533898	0.002050742
ENSG00000113194	FAF2	0.24	0.000535544	0.002056669
ENSG00000013374	NUB1	-0.37	0.000535926	0.002057743
ENSG00000188511	C22orf34	-0.60	0.000536407	0.002059194
ENSG00000254837	AP001372.2	1.13	0.000538563	0.002067072
ENSG00000123213	NLN	-0.33	0.000539468	0.002070152
ENSG00000136231	IGF2BP3	0.38	0.000540239	0.002072711
ENSG00000174206	C12orf66	-0.54	0.000542341	0.002080376
ENSG00000163395	IGFN1	3.41	0.000544029	0.002086454
ENSG00000248712	CCDC153	1.38	0.00054427	0.002086976
ENSG00000223612	AC241585.1	1.79	0.000544611	0.002087886
ENSG00000175970	UNC119B	-0.29	0.000548266	0.002101494
ENSG00000007968	E2F2	-0.41	0.000549179	0.002104591
ENSG00000145996	CDKAL1	0.51	0.000549926	0.002107048
ENSG00000174123	TLR10	2.74	0.000552094	0.002114952
ENSG00000135124	P2RX4	-0.54	0.000552258	0.002115174
ENSG00000155287	SLC25A28	-0.37	0.000552846	0.00211702
ENSG00000109171	SLAIN2	0.39	0.000553415	0.002118794
ENSG00000175066	GK5	-0.54	0.000555463	0.002126229
ENSG00000254612	DNAJB6P1	0.81	0.00055708	0.002132011
ENSG00000198798	MAGEB3	3.78	0.000557225	0.002132157
ENSG00000003402	CFLAR	0.37	0.00055792	0.00213441
ENSG00000171566	PLRG1	0.25	0.000559506	0.002140069
ENSG00000007255	TRAPPC6A	-0.74	0.000560053	0.00214175
ENSG00000067066	SP100	0.46	0.000561677	0.002147552
ENSG00000176595	KBTBD11	0.60	0.000562286	0.002149467
ENSG00000043591	ADRB1	1.08	0.000562579	0.002150176
ENSG00000129646	QRICH2	-0.90	0.000563146	0.002151936
ENSG00000164091	WDR82	0.22	0.000563741	0.002153796
ENSG00000167703	SLC43A2	-0.54	0.000567647	0.002168304
ENSG00000159905	ZNF221	1.56	0.000569161	0.002173674
ENSG00000100784	RPS6KA5	-0.60	0.000569347	0.00217397
ENSG00000129680	MAP7D3	-0.59	0.000570273	0.002177091
ENSG00000198840	MT-ND3	-0.24	0.000570509	0.002177574

ENSG00000167595	PROSER3	-0.56	0.000571695	0.002181684
ENSG00000169045	HNRNPH1	-0.20	0.000573442	0.002187937
ENSG00000153714	LURAP1L	-1.00	0.000574193	0.002190383
ENSG00000141858	SAMD1	-0.40	0.000574416	0.002190815
ENSG00000183742	MACC1	0.86	0.000574867	0.002192118
ENSG00000146476	ARMT1	-0.47	0.000575295	0.002193334
ENSG00000111911	HINT3	0.43	0.000575832	0.002194963
ENSG00000179029	TMEM107	-0.55	0.000576456	0.002196921
ENSG00000143512	HHIPL2	3.00	0.000577206	0.002199362
ENSG00000163694	RBM47	0.45	0.00058011	0.002210005
ENSG00000130449	ZSWIM6	0.59	0.000580671	0.002211722
ENSG00000165194	PCDH19	1.58	0.000581273	0.002213593
ENSG00000140995	DEF8	0.25	0.000581691	0.002214763
ENSG00000196975	ANXA4	-0.37	0.000583031	0.002219263
ENSG00000179094	PER1	-0.41	0.000583094	0.002219263
ENSG00000117395	EBNA1BP2	-0.27	0.00058387	0.002221791
ENSG00000164305	CASP3	0.58	0.0005841	0.002222246
ENSG00000214595	EML6	0.81	0.000585732	0.002228031
ENSG00000196636	SDHAF3	-0.72	0.000586659	0.002231131
ENSG00000178409	BEND3	-0.66	0.000587841	0.002235202
ENSG00000169609	C15orf40	0.56	0.000589341	0.002240482
ENSG00000145016	RUBCN	0.52	0.000591305	0.00224752
ENSG00000077097	TOP2B	0.29	0.000595412	0.0022627
ENSG00000143198	MGST3	0.32	0.000597875	0.002271628
ENSG00000073792	IGF2BP2	-0.25	0.000599049	0.002275657
ENSG00000153558	FBXL2	-0.69	0.000599829	0.002278187
ENSG00000170522	ELOVL6	0.33	0.000600525	0.002280399
ENSG00000102547	CAB39L	0.79	0.00060069	0.002280594
ENSG00000163634	THOC7	0.35	0.000601215	0.002282153
ENSG00000101003	GINS1	0.29	0.000601663	0.002283423
ENSG00000160588	MPZL3	0.62	0.000601883	0.002283824
ENSG00000167608	TMC4	-0.58	0.000608153	0.002307176
ENSG00000151689	INPP1	0.47	0.00060909	0.002310293
ENSG00000106049	HIBADH	-0.39	0.000610408	0.002314856
ENSG00000188897	AC099489.1	-1.92	0.000611026	0.002316759
ENSG00000187609	EXD3	-0.83	0.000611379	0.002317658
ENSG00000124562	SNRPC	0.50	0.000611735	0.00231857
ENSG00000163882	POLR2H	-0.28	0.000614824	0.002329836
ENSG00000270170	NCBP2AS2	-0.51	0.00061674	0.002336654
ENSG00000058262	SEC61A1	0.21	0.000616929	0.002336928
ENSG00000118193	KIF14	0.39	0.000619285	0.002345406
ENSG00000231074	HCG18	-0.50	0.000622792	0.002357854
ENSG00000181392	SYNE4	0.46	0.000622807	0.002357854
ENSG00000122741	DCAF10	-0.47	0.000624534	0.002363943
ENSG00000112186	CAP2	0.72	0.000625554	0.002367359
ENSG00000123552	USP45	-0.57	0.000627774	0.00237531

ENSG00000085382	HACE1	-0.65	0.000628833	0.002378865
ENSG00000139636	LMBR1L	-0.52	0.000629245	0.002379974
ENSG00000127824	TUBA4A	0.37	0.000630806	0.00238543
ENSG00000169891	REPS2	-0.97	0.000632315	0.002390683
ENSG00000125149	C16orf70	0.42	0.000633931	0.002396342
ENSG00000171169	NAIF1	0.52	0.000635046	0.0024001
ENSG00000274265	AC245297.3	0.97	0.00063691	0.002406693
ENSG00000150051	MKX	-0.69	0.000637489	0.002408426
ENSG00000185818	NAT8L	0.56	0.000638598	0.002412161
ENSG00000198715	GLMP	0.51	0.00064114	0.002421307
ENSG00000121897	LIAS	-0.54	0.0006431	0.002428248
ENSG00000285928	AC103591.4	2.13	0.000646166	0.002439364
ENSG00000177370	TIMM22	0.41	0.000647334	0.002443038
ENSG00000138463	SLC49A4	0.51	0.000647383	0.002443038
ENSG00000148516	ZEB1	0.85	0.000647798	0.002444142
ENSG00000207870	MIR221	1.56	0.000648578	0.002446626
ENSG00000135678	CPM	0.98	0.00064904	0.002447904
ENSG00000143753	DEGS1	0.32	0.000650107	0.002451468
ENSG00000267321	SNHG30	0.62	0.000650539	0.002452634
ENSG00000112697	TMEM30A	0.25	0.000651179	0.002454585
ENSG00000165661	QSOX2	-0.31	0.000653266	0.002461987
ENSG00000134419	RPS15A	-0.20	0.000654727	0.002467031
ENSG00000143669	LYST	0.70	0.000656581	0.00247355
ENSG00000055917	PUM2	0.29	0.000657047	0.002474839
ENSG00000139133	ALG10	-0.97	0.000657533	0.002476205
ENSG00000023572	GLRX2	-0.34	0.000659093	0.002481277
ENSG00000125743	SNRPD2	0.20	0.000659128	0.002481277
ENSG00000010704	HFE	-0.64	0.000659551	0.002482403
ENSG00000126756	UXT	0.37	0.000661214	0.002488194
ENSG00000055609	KMT2C	0.40	0.000664379	0.002499634
ENSG00000006007	GDE1	0.33	0.000665965	0.002505132
ENSG00000023330	ALAS1	0.26	0.000666254	0.002505747
ENSG00000254057	AC084346.1	1.33	0.000666545	0.002506369
ENSG00000185252	ZNF74	-0.63	0.000667437	0.002508837
ENSG00000105671	DDX49	0.32	0.000667452	0.002508837
ENSG00000106400	ZNHIT1	0.32	0.000667768	0.002509553
ENSG00000280433	FP565260.6	-1.46	0.000668124	0.00251042
ENSG00000140691	ARMC5	0.60	0.000668726	0.002512211
ENSG00000171148	TADA3	0.25	0.000669182	0.00251345
ENSG00000163444	TMEM183A	-0.28	0.000671541	0.002521838
ENSG00000115525	ST3GAL5	0.63	0.000674599	0.002532849
ENSG00000213553	RPLP0P6	-0.28	0.000674959	0.002533723
ENSG00000221955	SLC12A8	-0.58	0.000675616	0.002535713
ENSG00000260877	AP005233.2	-1.39	0.000677283	0.002541492
ENSG00000144048	DUSP11	0.44	0.000678547	0.002545759
ENSG00000215126	CBWD6	0.69	0.000680889	0.002554067

ENSG00000175575	PAAF1	0.53	0.000682372	0.002559149
ENSG00000165572	KBTBD6	-0.52	0.00068272	0.002559975
ENSG00000180354	MTURN	-0.70	0.000683156	0.002561129
ENSG00000168970	JMJD7-PLA2G	-1.03	0.000683646	0.002562487
ENSG00000009724	MASP2	-1.94	0.00068675	0.002573641
ENSG00000182108	DEXI	1.23	0.000689162	0.002582196
ENSG00000095752	IL11	1.10	0.000691089	0.002588932
ENSG00000112592	TBP	0.45	0.000691447	0.002589786
ENSG00000137976	DNASE2B	4.28	0.000691637	0.002590016
ENSG00000177098	SCN4B	1.83	0.000693174	0.002595285
ENSG00000106608	URGCP	0.37	0.000694628	0.002600243
ENSG00000114127	XRN1	-0.52	0.000695114	0.002601576
ENSG00000099889	ARVCF	-0.40	0.000695667	0.002603156
ENSG00000167565	SERTAD3	0.42	0.000696579	0.002606083
ENSG00000101160	CTSZ	0.24	0.000698261	0.002611889
ENSG00000171132	PRKCE	0.59	0.000698469	0.002612178
ENSG00000168807	SNTB2	-0.37	0.000699834	0.002616123
ENSG00000225676	AC002378.1	-6.02	0.000699993	0.002616123
ENSG00000276272	AC024884.2	-6.02	0.000699993	0.002616123
ENSG00000108107	RPL28	-0.51	0.000700047	0.002616123
ENSG00000279059	AC007485.2	-0.97	0.000700339	0.002616724
ENSG00000115839	RAB3GAP1	0.27	0.000701894	0.002622048
ENSG00000155957	TMBIM4	-0.59	0.000702848	0.002625118
ENSG00000085982	USP40	-0.33	0.000703495	0.002626759
ENSG00000261170	AC009053.3	-1.58	0.000703549	0.002626759
ENSG00000064102	INTS13	0.41	0.000706793	0.002638379
ENSG00000260852	FBXL19-AS1	0.65	0.000707578	0.002640815
ENSG00000147813	NAPRT	-0.31	0.000708006	0.002641919
ENSG00000176749	CDK5R1	0.61	0.000708403	0.002642907
ENSG00000255031	AP002807.1	-2.59	0.000709291	0.002645542
ENSG00000179292	TMEM151A	0.67	0.000709373	0.002645542
ENSG00000130713	EXOSC2	-0.30	0.000709541	0.002645674
ENSG00000147050	KDM6A	0.48	0.000710722	0.002649585
ENSG00000134333	LDHA	0.26	0.000713396	0.002659059
ENSG00000132676	DAP3	0.23	0.000716576	0.002670412
ENSG00000249565	SERBP1P5	0.56	0.000718665	0.002677701
ENSG00000162944	RFTN2	1.82	0.000720857	0.002685365
ENSG00000111666	CHPT1	0.53	0.000721758	0.002688223
ENSG00000183048	SLC25A10	-0.42	0.00072474	0.002698829
ENSG00000104093	DMXL2	-0.54	0.000725442	0.002700939
ENSG00000274419	TBC1D3D	-1.29	0.000725671	0.00270129
ENSG00000168890	TMEM150A	-0.99	0.000725965	0.00270188
ENSG00000223478	AL441992.1	0.64	0.000729821	0.002715727
ENSG00000162066	AMDHD2	0.45	0.0007313	0.002720724
ENSG00000113812	ACTR8	0.46	0.000735495	0.002735822
ENSG00000247796	AC008966.1	-1.19	0.000737764	0.002743753

ENSG00000267073	AC005256.1	0.70	0.0007393	0.002748774
ENSG00000275993	SIK1B	1.09	0.000739419	0.002748774
ENSG00000139620	KANSL2	0.35	0.000739526	0.002748774
ENSG00000178057	NDUFAF3	0.32	0.000742913	0.00276085
ENSG00000187742	SECISBP2	-0.34	0.000748963	0.002782816
ENSG00000211460	TSN	0.23	0.00074917	0.00278307
ENSG00000275074	NUDT18	-0.87	0.000749421	0.002783348
ENSG00000140807	NKD1	-0.51	0.000749523	0.002783348
ENSG00000165233	CARD19	0.41	0.000752112	0.002792442
ENSG00000108771	DHX58	1.72	0.000752588	0.002793693
ENSG00000160075	SSU72	-0.29	0.000753442	0.002796343
ENSG00000106617	PRKAG2	-0.37	0.000755166	0.002802222
ENSG00000198074	AKR1B10	2.23	0.0007556	0.002803311
ENSG00000213713	PIGCP1	-1.62	0.000756814	0.002807296
ENSG00000153789	CIBAR2	2.30	0.000757136	0.002807969
ENSG00000243896	OR2A7	-2.69	0.000758194	0.002811373
ENSG00000160602	NEK8	-1.05	0.000761948	0.002824768
ENSG00000175581	MRPL48	0.38	0.000765085	0.002835873
ENSG00000142178	SIK1	1.18	0.000765743	0.002837785
ENSG00000125356	NDUFA1	0.27	0.000766496	0.00284005
ENSG00000162244	RPL29	-0.25	0.00076825	0.002846025
ENSG00000189343	RPS2P46	-0.27	0.000772519	0.002861309
ENSG00000133316	WDR74	-0.32	0.000773789	0.002865483
ENSG00000173137	ADCK5	-0.57	0.000774561	0.002867812
ENSG00000175707	KDF1	-0.57	0.00077492	0.002868608
ENSG00000102738	MRPS31	0.61	0.000775609	0.002870628
ENSG00000163964	PIGX	-0.46	0.000777538	0.002877236
ENSG00000159314	ARHGAP27	0.33	0.000778778	0.002881293
ENSG00000232593	KANTR	-0.71	0.000779168	0.002882202
ENSG00000176208	ATAD5	-0.62	0.000779983	0.002884682
ENSG00000159335	PTMS	-0.48	0.000782331	0.002892834
ENSG00000142449	FBN3	-0.84	0.000787637	0.002911913
ENSG00000110395	CBL	0.32	0.000790152	0.002920673
ENSG00000131115	ZNF227	0.57	0.000791505	0.002925132
ENSG00000197847	SLC22A20P	-2.30	0.000794936	0.002937271
ENSG00000102678	FGF9	-0.74	0.000795232	0.002937823
ENSG00000102144	PGK1	0.22	0.000795755	0.002939213
ENSG00000223960	CHROMR	-0.67	0.000796348	0.002940861
ENSG00000140104	CLBA1	-0.63	0.000796657	0.002941457
ENSG00000105576	TNPO2	0.25	0.000797344	0.002943452
ENSG00000203791	EEF1AKMT2	-0.39	0.000797724	0.00294431
ENSG00000125868	DSTN	-0.23	0.000798836	0.00294787
ENSG00000163623	NKX6-1	0.62	0.000799304	0.002949054
ENSG00000130675	MNX1	0.65	0.000802624	0.002960756
ENSG00000197258	EIF4BP6	0.49	0.000805481	0.00297075
ENSG00000227036	LINC00511	-0.35	0.000806667	0.002974576

ENSG00000196456	ZNF775	0.72	0.000807933	0.002978696
ENSG00000033800	PIAS1	0.44	0.000813443	0.002998288
ENSG00000240053	LY6G5B	-1.14	0.000813547	0.002998288
ENSG00000092978	GPATCH2	-0.64	0.000815752	0.003005862
ENSG00000129910	CDH15	0.81	0.000818321	0.003014404
ENSG00000225605	AC092813.1	3.41	0.000818372	0.003014404
ENSG00000083457	ITGAE	0.37	0.000818578	0.00301461
ENSG00000259495	AC016705.2	-2.56	0.00081905	0.003015793
ENSG00000163762	TM4SF18	-0.75	0.000821631	0.003024489
ENSG00000283050	GTF2IP12	-1.04	0.000821714	0.003024489
ENSG00000165275	TRMT10B	-0.70	0.000822822	0.00302801
ENSG00000168234	TTC39C	0.50	0.000823586	0.003030262
ENSG00000269427	AC024075.3	-2.43	0.000824412	0.003032746
ENSG00000064687	ABCA7	-0.33	0.000826297	0.00303912
ENSG00000182552	RWDD4	-0.75	0.000826571	0.003039571
ENSG00000183856	IQGAP3	-0.35	0.000828635	0.003046599
ENSG00000186073	C15orf41	-0.77	0.000836853	0.003075816
ENSG00000137203	TFAP2A	-0.54	0.000836889	0.003075816
ENSG00000184916	JAG2	-0.26	0.000837561	0.003077722
ENSG00000248643	RBM14-RBM4	1.25	0.000845929	0.003107901
ENSG00000143353	LYPLAL1	-0.55	0.00084641	0.003109097
ENSG00000173786	CNP	-0.32	0.000848753	0.003117131
ENSG00000205138	SDHAF1	-0.51	0.000849498	0.003119293
ENSG00000101751	POLI	-0.56	0.00084971	0.003119502
ENSG00000115446	UNC50	0.36	0.000852199	0.003128063
ENSG00000217801	AL390719.1	0.54	0.000853121	0.003130874
ENSG00000182372	CLN8	0.42	0.000855062	0.003137423
ENSG00000070061	ELP1	-0.41	0.000856347	0.00314156
ENSG00000164900	GBX1	1.43	0.000857258	0.003144326
ENSG00000113648	MACROH2A1	-0.21	0.000858784	0.003149347
ENSG00000236698	EIF1AXP1	0.48	0.000860859	0.003156379
ENSG00000137767	SQOR	0.66	0.000866869	0.003177831
ENSG00000184160	ADRA2C	0.55	0.000868033	0.003181514
ENSG00000122483	CCDC18	0.63	0.000868527	0.003182742
ENSG00000250934	AC016924.1	2.04	0.000868976	0.003183805
ENSG00000180263	FGD6	-0.51	0.000870872	0.003190168
ENSG00000100393	EP300	-0.36	0.000871821	0.003193062
ENSG00000102893	PHKB	0.38	0.000874411	0.003201961
ENSG00000135740	SLC9A5	0.70	0.000878356	0.003215819
ENSG00000147099	HDAC8	0.68	0.0008786	0.003216122
ENSG00000119396	RAB14	-0.26	0.000879001	0.003217003
ENSG00000095637	SORBS1	0.77	0.000879624	0.003218693
ENSG00000247679	AC139795.2	-1.27	0.000880041	0.003219633
ENSG00000153214	TMEM87B	-0.63	0.000881143	0.003223074
ENSG00000172795	DCP2	0.30	0.000882807	0.003228571
ENSG00000133812	SBF2	0.46	0.000884497	0.003234158

ENSG00000027075	PRKCH	0.44	0.000884988	0.003235362
ENSG00000075413	MARK3	0.30	0.000889117	0.003249864
ENSG00000206262	FOXL2NB	-2.99	0.000889507	0.003250697
ENSG00000105928	GSDME	-0.69	0.000890006	0.003251927
ENSG00000158715	SLC45A3	-0.62	0.000892829	0.003261413
ENSG00000168913	ENHO	0.77	0.000892928	0.003261413
ENSG00000218510	LINC00339	-1.01	0.000895435	0.00326997
ENSG00000213928	IRF9	1.02	0.000898167	0.003279349
ENSG00000131067	GGT7	0.54	0.000908994	0.003318274
ENSG00000284648	AC097493.3	1.76	0.000910214	0.003322125
ENSG00000184634	MED12	-0.44	0.000911649	0.003326753
ENSG00000197301	HMGA2-AS1	-1.59	0.000912992	0.003331047
ENSG00000284606	AC105233.4	-1.90	0.000913564	0.003332525
ENSG00000172250	SERHL	-2.23	0.000915203	0.003337898
ENSG00000141564	RPTOR	0.29	0.000916493	0.003341994
ENSG00000258744	AL132800.1	-1.77	0.000917704	0.003345545
ENSG00000230091	TMEM254-AS1	-2.12	0.000917801	0.003345545
ENSG00000085871	MGST2	0.57	0.00092058	0.003354845
ENSG00000160221	GATD3A	-0.46	0.000920688	0.003354845
ENSG00000244471	AL137058.1	4.22	0.000921387	0.00335678
ENSG00000012983	MAP4K5	-0.42	0.000922341	0.003359645
ENSG00000184060	ADAP2	0.56	0.000923619	0.003363689
ENSG00000253651	SOD1P3	-3.41	0.000923889	0.003364058
ENSG00000237452	BHMG1	-1.61	0.000924255	0.003364778
ENSG00000249942	AC239584.1	1.73	0.000925205	0.003367625
ENSG00000186529	CYP4F3	-1.01	0.000926284	0.00337045
ENSG00000215256	DHRS4-AS1	-0.59	0.000926318	0.00337045
ENSG00000177888	ZBTB41	0.44	0.000928119	0.00337639
ENSG00000037241	RPL26L1	0.36	0.000928952	0.003378807
ENSG00000047056	WDR37	0.48	0.000929516	0.003380242
ENSG00000011260	UTP18	0.27	0.000930327	0.003382107
ENSG00000170356	OR2A20P	-1.67	0.000930471	0.003382107
ENSG00000287307	AC005772.2	3.69	0.000930536	0.003382107
ENSG00000139266	MARCHF9	0.86	0.000933596	0.003392615
ENSG00000236778	INTS6-AS1	-1.46	0.000937436	0.003405951
ENSG00000092470	WDR76	0.32	0.00093771	0.003406325
ENSG00000161681	SHANK1	1.22	0.000938557	0.003408783
ENSG00000163956	LRPAP1	0.23	0.000939212	0.003410546
ENSG00000163354	DCST2	-1.95	0.00094049	0.003414567
ENSG00000185414	MRPL30	-0.37	0.000942321	0.003420594
ENSG00000147155	EBP	0.41	0.000944304	0.003427169
ENSG00000163602	RYBP	0.37	0.000945623	0.003430592
ENSG00000198932	GPRASP1	-3.72	0.000945661	0.003430592
ENSG00000112659	CUL9	-0.48	0.000945761	0.003430592
ENSG00000068885	IFT80	-0.60	0.000947676	0.003436913
ENSG00000166986	MARS1	0.20	0.000949497	0.003442894

ENSG00000224051	CPTP	-0.50	0.000952741	0.003454032
ENSG00000161551	ZNF577	-1.16	0.000953184	0.00345501
ENSG00000120217	CD274	1.12	0.000955434	0.003462541
ENSG00000186470	BTN3A2	0.46	0.000955661	0.003462735
ENSG00000105738	SIPA1L3	-0.38	0.000963062	0.003488921
ENSG00000100034	PPM1F	0.31	0.000972762	0.003523423
ENSG00000163072	NOSTRIN	-1.31	0.000977077	0.003538412
ENSG00000160191	PDE9A	-0.47	0.000978524	0.00354294
ENSG00000168917	SLC35G2	0.77	0.000978681	0.00354294
ENSG00000244509	APOBEC3C	0.29	0.000980622	0.003549324
ENSG00000269473	AC012313.8	-1.45	0.000983888	0.0035605
ENSG00000271122	AC018647.2	-0.68	0.000987358	0.003572413
ENSG00000101901	ALG13	0.43	0.000991459	0.003586166
ENSG00000129167	TPH1	-2.79	0.000991518	0.003586166
ENSG00000196455	PIK3R4	0.42	0.000992983	0.003590817
ENSG00000130706	ADRM1	0.25	0.000993415	0.003591729
ENSG00000205559	CHKB-DT	-1.07	0.000994799	0.003596084
ENSG00000110811	P3H3	0.38	0.000995717	0.003598252
ENSG00000254901	BORCS8	0.83	0.000995759	0.003598252
ENSG00000166268	MYRFL	3.31	0.001003686	0.003626243
ENSG00000171720	HDAC3	-0.28	0.00100786	0.003640666
ENSG00000023902	PLEKHO1	0.60	0.001011076	0.003651623
ENSG00000087903	RFX2	-0.56	0.001013007	0.003657938
ENSG00000102043	MTMR8	1.40	0.001013276	0.003658112
ENSG00000180673	EXOC5P1	2.12	0.001013421	0.003658112
ENSG00000179262	RAD23A	-0.27	0.001014165	0.003660138
ENSG00000127334	DYRK2	-0.40	0.001014979	0.003662416
ENSG00000180198	RCC1	-0.22	0.001015981	0.003665372
ENSG00000102524	TNFSF13B	3.05	0.001019064	0.003675832
ENSG00000162980	ARL5A	-0.40	0.001021331	0.003683344
ENSG00000163635	ATXN7	-0.42	0.001025201	0.003696635
ENSG00000162783	IER5	0.27	0.001028164	0.00370665
ENSG00000162444	RBP7	0.40	0.001032306	0.003720911
ENSG00000074071	MRPS34	-0.27	0.001035134	0.003730435
ENSG00000272263	AC034198.2	-4.18	0.001036539	0.003734826
ENSG00000146950	SHROOM2	-0.88	0.00103853	0.003741325
ENSG00000118705	RPN2	-0.20	0.001039544	0.003744304
ENSG00000128253	RFPL2	2.22	0.001040966	0.003748752
ENSG00000132432	SEC61G	0.26	0.001042052	0.003751987
ENSG00000083937	CHMP2B	0.35	0.001042301	0.00375221
ENSG00000188064	WNT7B	0.58	0.001044743	0.003760215
ENSG00000138814	PPP3CA	0.23	0.001044901	0.003760215
ENSG00000125740	FOSB	1.03	0.001045247	0.003760783
ENSG00000269929	MIRLET7A1HG	0.74	0.00104857	0.003772063
ENSG00000047932	GOPC	0.38	0.001050649	0.003778862
ENSG00000132906	CASP9	0.54	0.001051731	0.003782075

ENSG00000283486	FAM95C	-0.61	0.001052086	0.003782672
ENSG00000141034	GID4	-0.52	0.001053381	0.003786646
ENSG00000103740	ACSBG1	1.52	0.001055409	0.003793256
ENSG00000017260	ATP2C1	0.26	0.001058299	0.003802958
ENSG00000113845	TIMMDC1	-0.31	0.001059202	0.00380552
ENSG00000160972	PPP1R16A	-0.39	0.001064265	0.003823025
ENSG00000184209	SNRNP35	0.58	0.001067064	0.003832391
ENSG00000228594	FNDC10	-1.20	0.001067729	0.003834091
ENSG00000241157	AC104763.1	5.91	0.001070788	0.003844385
ENSG00000173598	NUDT4	-0.35	0.001075137	0.003859309
ENSG00000229180	AC006001.3	0.40	0.001078943	0.003872276
ENSG00000138443	ABI2	-0.26	0.001081274	0.003879945
ENSG00000158246	TENT5B	0.41	0.001084401	0.003890468
ENSG00000073711	PPP2R3A	-0.93	0.001085001	0.003891924
ENSG00000198624	CCDC69	0.62	0.00108578	0.003894019
ENSG00000185813	PCYT2	-0.32	0.001086442	0.003895697
ENSG00000163002	NUP35	0.51	0.001087195	0.003897698
ENSG00000101558	VAPA	0.22	0.001088133	0.00390036
ENSG00000197111	PCBP2	0.23	0.001090954	0.003909774
ENSG00000170248	PDCD6IP	0.23	0.00109576	0.003926294
ENSG00000160961	ZNF333	-0.73	0.001097862	0.003933121
ENSG00000176853	FAM91A1	0.27	0.001106542	0.003963509
ENSG00000003756	RBM5	0.28	0.001106828	0.003963822
ENSG00000106462	EZH2	0.26	0.001110132	0.003974943
ENSG00000067057	PFKP	0.20	0.001115132	0.003992133
ENSG00000169139	UBE2V2	0.30	0.00111793	0.004001435
ENSG00000172244	C5orf34	0.67	0.001118182	0.004001621
ENSG00000103363	ELOB	0.28	0.001122933	0.004017905
ENSG00000189079	ARID2	0.39	0.001123832	0.004020401
ENSG00000217555	CKLF	0.67	0.00112406	0.004020498
ENSG00000081307	UBA5	-0.39	0.00112897	0.004037338
ENSG00000287271	AC011455.6	1.29	0.001130591	0.004042414
ENSG00000125388	GRK4	0.82	0.001131285	0.004044171
ENSG00000083099	LYRM2	-0.49	0.001133058	0.004049786
ENSG00000117602	RCAN3	0.43	0.001133959	0.004052283
ENSG00000115355	CCDC88A	0.41	0.001139485	0.004071304
ENSG00000134056	MRPS36	0.48	0.001139716	0.004071403
ENSG00000078804	TP53INP2	-0.62	0.001142682	0.004081269
ENSG00000103550	KNOP1	-0.43	0.001144807	0.004088129
ENSG00000198910	L1CAM	0.42	0.001145691	0.004090556
ENSG00000171940	ZNF217	0.38	0.001149731	0.004104249
ENSG00000169020	ATP5ME	0.31	0.001154396	0.004120167
ENSG00000107611	CUBN	1.45	0.001155282	0.004122595
ENSG00000280789	PAGR1	-0.43	0.001158378	0.004132905
ENSG00000176058	TPRN	0.43	0.001161423	0.004143031
ENSG00000198258	UBL5	0.27	0.001164947	0.004154861

ENSG00000134597	RBMX2	0.44	0.001165509	0.004156125
ENSG00000140382	HMG20A	0.40	0.001168694	0.00416674
ENSG00000157240	FZD1	-0.67	0.001169234	0.004167923
ENSG00000188763	FZD9	1.94	0.001172728	0.004178972
ENSG00000288056	AL023806.4	2.26	0.001172752	0.004178972
ENSG00000164556	FAM183BP	5.85	0.001176082	0.004190093
ENSG00000092295	TGM1	-1.66	0.001177318	0.004193749
ENSG00000198162	MAN1A2	0.39	0.00118171	0.004208645
ENSG00000179988	PSTK	1.00	0.001183575	0.004214537
ENSG00000187808	SOWAHD	1.62	0.001190371	0.004237982
ENSG00000177426	TGIF1	-0.22	0.001192552	0.004244991
ENSG00000198198	SZT2	-0.40	0.001195514	0.004254781
ENSG00000136856	SLC2A8	0.47	0.001196083	0.004256048
ENSG00000148841	ITPRIP	0.37	0.001197364	0.00425985
ENSG00000154719	MRPL39	0.45	0.001197759	0.004260495
ENSG00000144028	SNRNP200	0.19	0.001201717	0.004273816
ENSG00000154222	CC2D1B	0.33	0.001207874	0.00429495
ENSG00000188428	BLOC1S5	0.53	0.001208399	0.004296051
ENSG00000090621	PABPC4	-0.21	0.001211326	0.004305695
ENSG00000119616	FCF1	0.28	0.0012143	0.004315497
ENSG00000228793	AL033523.1	3.65	0.001216623	0.004322814
ENSG00000170089	AC106795.1	-0.57	0.001216791	0.004322814
ENSG00000286251	AC097528.1	5.85	0.001219096	0.004330235
ENSG00000128694	OSGEPL1	-0.68	0.001220335	0.004333866
ENSG00000154640	BTG3	0.28	0.001224245	0.004346445
ENSG00000065413	ANKRD44	1.10	0.001224311	0.004346445
ENSG00000108883	EFTUD2	0.20	0.001226106	0.004352044
ENSG00000204564	C6orf136	-0.52	0.001226681	0.004353314
ENSG00000175697	GPR156	3.61	0.001226946	0.004353483
ENSG00000006576	PHTF2	0.70	0.001228503	0.004358233
ENSG00000268798	AC027307.3	-5.26	0.001230443	0.004364343
ENSG00000231113	AL035587.1	-1.56	0.001233557	0.004374613
ENSG00000213465	ARL2	-0.41	0.001234345	0.004376631
ENSG00000145284	SCD5	0.31	0.001240572	0.004397931
ENSG00000150768	DLAT	0.27	0.00124317	0.004406362
ENSG00000108733	PEX12	0.66	0.001245486	0.004413788
ENSG00000145107	TM4SF19	1.19	0.001252385	0.004437452
ENSG00000169972	PUSL1	-0.38	0.001253434	0.004440383
ENSG00000147324	MFHAS1	-0.34	0.001258999	0.004459306
ENSG00000138617	PARP16	0.56	0.001259467	0.004460176
ENSG00000162148	PPP1R32	-0.96	0.001260574	0.004463307
ENSG00000079246	XRCC5	0.19	0.00126362	0.0044733
ENSG00000151240	DIP2C	0.34	0.001271845	0.00450162
ENSG00000092445	TYRO3	-0.33	0.001273595	0.004507018
ENSG00000272807	AC007038.1	-2.30	0.001274118	0.004507539
ENSG00000178038	ALS2CL	-0.60	0.001274193	0.004507539

ENSG00000177640	CASC2	-1.46	0.001275742	0.00451222
ENSG00000070018	LRP6	0.37	0.001281694	0.004532472
ENSG00000184178	SCFD2	0.57	0.00128204	0.004532896
ENSG00000165775	FUNDC2	0.34	0.001282994	0.004535468
ENSG00000132635	PCED1A	-0.54	0.001286627	0.004547506
ENSG00000155254	MARVELD1	-0.35	0.001287918	0.004551264
ENSG00000141179	PCTP	0.32	0.001289256	0.004555187
ENSG00000258674	AC011448.1	-1.78	0.001290289	0.004558035
ENSG00000131650	KREMEN2	0.48	0.001291347	0.004560738
ENSG00000065526	SPEN	-0.26	0.00129151	0.004560738
ENSG00000160062	ZBTB8A	-0.83	0.001297295	0.004580358
ENSG00000264175	MIR3189	-2.44	0.001298781	0.004584796
ENSG00000164897	TMUB1	0.30	0.001303877	0.004601973
ENSG00000137821	LRRC49	0.94	0.001311228	0.004626995
ENSG00000227081	AC005912.1	-0.32	0.001311569	0.004626995
ENSG00000121207	LRAT	-0.92	0.00131166	0.004626995
ENSG00000048544	MRPS10	0.25	0.001314696	0.004636886
ENSG00000267127	AC090360.1	2.23	0.001315193	0.004637822
ENSG00000168275	COA6	0.48	0.00131586	0.004639358
ENSG00000136463	TACO1	0.31	0.001319752	0.004652259
ENSG00000163785	RYK	-0.33	0.001320013	0.00465236
ENSG00000135540	NHSL1	-0.43	0.001325098	0.004669461
ENSG00000117013	KCNQ4	-0.84	0.001326788	0.004674592
ENSG00000117593	DARS2	0.33	0.001328819	0.004680922
ENSG00000168268	NT5DC2	-0.54	0.001329718	0.004683266
ENSG00000110002	VWA5A	0.86	0.00133173	0.004689526
ENSG00000230551	AC021078.1	0.51	0.001337724	0.004709489
ENSG00000143367	TUFT1	0.37	0.001337869	0.004709489
ENSG00000184584	STING1	0.55	0.001338808	0.004711965
ENSG00000183251	OR51B4	-0.80	0.001339456	0.004713416
ENSG00000118707	TGIF2	0.49	0.001344577	0.004730245
ENSG00000128602	SMO	-0.46	0.001344711	0.004730245
ENSG00000213740	SERBP1P1	0.55	0.001345333	0.0047316
ENSG00000107745	MICU1	-0.33	0.001345835	0.004732193
ENSG00000135249	RINT1	0.48	0.001345975	0.004732193
ENSG00000117118	SDHB	-0.23	0.001347303	0.004736032
ENSG00000096654	ZNF184	0.76	0.001349114	0.004741564
ENSG00000181191	PJA1	0.40	0.00135064	0.004746093
ENSG00000196684	HSH2D	0.51	0.001352585	0.004752095
ENSG00000140526	ABHD2	-0.33	0.0013545	0.004757989
ENSG00000121680	PEX16	-0.44	0.001355106	0.004759279
ENSG00000066230	SLC9A3	-0.91	0.001356357	0.00476284
ENSG00000171262	FAM98B	0.28	0.001356947	0.00476377
ENSG00000093217	XYLB	-0.86	0.001357098	0.00476377
ENSG00000260231	KDM7A-DT	-1.63	0.001357939	0.004765886
ENSG00000167272	POP5	-0.37	0.001363719	0.004785332

ENSG00000185495	AC138393.1	-1.17	0.00136589	0.00479211
ENSG00000198561	CTNND1	-0.87	0.001368845	0.004801635
ENSG00000256673	AC141557.1	-2.58	0.001370169	0.004805438
ENSG00000148584	A1CF	2.36	0.001370999	0.004807506
ENSG00000166435	XRRA1	0.60	0.001372253	0.004811061
ENSG00000169435	RASSF6	1.17	0.001373064	0.004813059
ENSG00000154127	UBASH3B	0.34	0.001373458	0.004813599
ENSG00000105613	MAST1	0.45	0.001373808	0.004813982
ENSG00000072195	SPEG	-0.35	0.001375182	0.004817951
ENSG00000111816	FRK	0.57	0.001384008	0.004847624
ENSG00000156050	FAM161B	0.93	0.001384136	0.004847624
ENSG00000228544	CCDC183-AS1	-1.84	0.001384405	0.004847719
ENSG00000164307	ERAP1	-0.93	0.001385417	0.004850413
ENSG00000139190	VAMP1	0.60	0.001386893	0.004854732
ENSG00000117899	MESD	-0.28	0.001388701	0.004860212
ENSG00000132780	NASP	-0.22	0.001389573	0.004862412
ENSG00000096746	HNRNPH3	0.21	0.001390123	0.004863486
ENSG00000115226	FNDC4	0.89	0.001393181	0.004873332
ENSG00000170270	GON7	-0.72	0.001395643	0.004881092
ENSG00000145198	VWA5B2	-0.71	0.001400508	0.004897251
ENSG00000069966	GNB5	-0.40	0.00140351	0.004906892
ENSG00000076928	ARHGEF1	-0.28	0.001405721	0.00491376
ENSG00000286699	AC084198.4	-1.06	0.001415143	0.004945833
ENSG00000141504	SAT2	0.41	0.001419702	0.004960899
ENSG00000117010	ZNF684	0.96	0.001420074	0.004961334
ENSG00000066629	EML1	-0.63	0.001422847	0.004970155
ENSG00000168806	LCMT2	-0.74	0.001426205	0.004981015
ENSG00000259431	THTPA	0.97	0.001426562	0.004981391
ENSG00000230074	AL162231.2	-0.91	0.001428554	0.004987478
ENSG00000115423	DNAH6	-1.45	0.001432738	0.005001211
ENSG00000085644	ZNF213	-0.58	0.00143522	0.005009005
ENSG00000138658	ZGRF1	-0.49	0.001435838	0.005010286
ENSG00000128965	CHAC1	0.29	0.001438175	0.005017565
ENSG00000080823	MOK	0.70	0.001439198	0.005020259
ENSG00000182872	RBM10	-0.26	0.001441543	0.005027563
ENSG00000179532	DNHD1	-0.66	0.001442456	0.00502987
ENSG00000146872	TLK2	0.31	0.001445958	0.00504116
ENSG00000160752	FDPS	0.22	0.001446197	0.00504116
ENSG00000129007	CALML4	-1.51	0.001448462	0.005048177
ENSG00000115350	POLE4	0.51	0.001453869	0.005066136
ENSG00000138193	PLCE1	-0.67	0.001456037	0.005072808
ENSG00000022840	RNF10	0.23	0.001457244	0.005076129
ENSG00000135070	ISCA1	-0.38	0.001458215	0.00507863
ENSG00000174529	TMEM81	0.80	0.001462435	0.005092439
ENSG00000287356	AL590822.3	-1.52	0.001464318	0.00509811
ENSG00000129675	ARHGEF6	-1.09	0.001466275	0.005104035

ENSG00000204305	AGER	-0.84	0.001467337	0.005106842
ENSG00000278768	BACE1-AS	-0.85	0.001474677	0.005131497
ENSG00000165392	WRN	-0.50	0.001476185	0.005135849
ENSG00000212719	LINC02693	-0.42	0.001478253	0.005142153
ENSG00000147421	HMBOX1	0.57	0.001483019	0.005157834
ENSG00000172725	CORO1B	-0.27	0.001485963	0.005167174
ENSG00000109762	SNX25	-0.48	0.001487074	0.00517014
ENSG00000160087	UBE2J2	0.28	0.001489789	0.005178679
ENSG00000055130	CUL1	0.29	0.001492629	0.005187649
ENSG00000173120	KDM2A	-0.27	0.001499684	0.005211267
ENSG00000142188	TMEM50B	-0.36	0.001502243	0.00521925
ENSG00000162601	MYSM1	-0.45	0.001507627	0.005237047
ENSG00000106686	SPATA6L	2.19	0.001509668	0.005243226
ENSG00000223804	AC244669.1	0.93	0.001513176	0.005254499
ENSG00000146776	ATXN7L1	-0.70	0.00152839	0.005306407
ENSG00000175065	DSG4	5.84	0.001529433	0.005309111
ENSG00000204588	LINC01123	-1.06	0.001534074	0.005324295
ENSG00000116521	SCAMP3	0.28	0.001537859	0.005336508
ENSG00000095321	CRAT	-0.50	0.001538754	0.005338685
ENSG00000267006	AC008507.2	-5.81	0.001540546	0.005343977
ENSG00000076770	MBNL3	0.87	0.001541569	0.005346599
ENSG00000003249	DBNDD1	0.30	0.001544596	0.005356169
ENSG00000240857	RDH14	-0.56	0.001547185	0.005364217
ENSG00000070371	CLTCL1	-0.70	0.001547528	0.005364479
ENSG00000127481	UBR4	-0.21	0.001548606	0.005367285
ENSG00000283312	AC017104.3	-2.86	0.001550972	0.005374555
ENSG00000163082	SGPP2	-0.81	0.001552464	0.005378794
ENSG00000138061	CYP1B1	1.08	0.001553116	0.005380122
ENSG00000142751	GPN2	-0.34	0.001555308	0.00538678
ENSG00000164144	ARFIP1	0.36	0.001556167	0.005388823
ENSG00000145782	ATG12	-0.26	0.001559473	0.005399338
ENSG00000261342	AC006538.1	-1.76	0.001565103	0.005417892
ENSG00000157911	PEX10	-0.44	0.00156661	0.005422172
ENSG00000076108	BAZ2A	0.21	0.001566954	0.005422424
ENSG00000128463	EMC4	0.31	0.001568358	0.005426344
ENSG00000137824	RMDN3	-0.35	0.001575622	0.005450536
ENSG00000144355	DLX1	1.01	0.001581357	0.00546943
ENSG00000107372	ZFAND5	0.23	0.00158313	0.005474616
ENSG00000143514	TP53BP2	0.34	0.001584781	0.005479379
ENSG00000171574	ZNF584	-0.55	0.001585409	0.005480601
ENSG00000117481	NSUN4	0.36	0.001586525	0.005483514
ENSG00000280399	AC022497.1	-2.49	0.001586876	0.00548378
ENSG00000129317	PUS7L	-0.49	0.001591348	0.005498283
ENSG00000211445	GPX3	-5.87	0.001596042	0.005513548
ENSG00000171421	MRPL36	-0.47	0.001596886	0.005515513
ENSG00000198420	TCAF1	0.31	0.001601364	0.005529747

ENSG00000135452	TSPAN31	0.45	0.00160156	0.005529747
ENSG00000105717	PBX4	0.64	0.001603592	0.00553581
ENSG00000145861	C1QTNF2	2.04	0.001607366	0.005547881
ENSG00000165516	KLHDC2	-0.46	0.00160801	0.005549147
ENSG00000213339	QTRT1	-0.41	0.001611887	0.005561566
ENSG00000233369	GTF2IP4	0.56	0.00161251	0.005562758
ENSG00000160113	NR2F6	0.28	0.001612977	0.005563409
ENSG00000184076	UQCR10	0.29	0.001613705	0.005564961
ENSG00000230002	ALMS1-IT1	-5.21	0.001614405	0.005566414
ENSG00000287787	AC092275.1	1.79	0.00161743	0.005575735
ENSG00000185499	MUC1	1.22	0.001617665	0.005575735
ENSG00000099954	CECR2	0.56	0.001622518	0.005591498
ENSG00000138030	KHK	0.60	0.001624196	0.005596316
ENSG00000135972	MRPS9	0.32	0.001630214	0.005616087
ENSG00000235316	DUSP8P5	1.53	0.001632087	0.005621572
ENSG00000107937	GTPBP4	0.24	0.001632469	0.005621919
ENSG00000117543	DPH5	-0.39	0.001635474	0.005631299
ENSG00000163166	IWS1	0.31	0.001636332	0.005633285
ENSG00000162227	TAF6L	0.44	0.001637851	0.005637543
ENSG00000184368	MAP7D2	3.54	0.001640472	0.00564478
ENSG00000129493	HEATR5A	0.55	0.001640518	0.00564478
ENSG00000171450	CDK5R2	1.31	0.001643598	0.005654407
ENSG00000185532	PRKG1	1.61	0.001644157	0.005655359
ENSG00000005339	CREBBP	0.34	0.001646652	0.005662966
ENSG00000197461	PDGFA	0.43	0.00164825	0.005667487
ENSG00000111802	TDP2	0.35	0.001651341	0.005677142
ENSG00000049283	EPN3	-0.77	0.001652147	0.005678935
ENSG00000171862	PTEN	0.28	0.001653567	0.005682841
ENSG00000188735	TMEM120B	-0.36	0.001653941	0.005683151
ENSG00000145779	TNFAIP8	-0.62	0.001657576	0.005694664
ENSG00000085449	WDFY1	-0.41	0.001658016	0.005695197
ENSG00000127080	IPPK	0.49	0.001660578	0.005703017
ENSG00000179387	ELMOD2	-0.39	0.001663586	0.00571237
ENSG00000180044	C3orf80	-1.42	0.00166458	0.0057148
ENSG00000168398	BDKRB2	1.52	0.001665249	0.005716117
ENSG00000183751	TBL3	-0.28	0.001666378	0.005719012
ENSG00000117676	RPS6KA1	0.26	0.001672205	0.005738027
ENSG00000197601	FAR1	-0.30	0.001675195	0.005747299
ENSG00000228393	LINC01004	1.71	0.00167859	0.005757961
ENSG00000111331	OAS3	-0.30	0.001681254	0.005766112
ENSG00000161920	MED11	-0.73	0.001685164	0.00577853
ENSG00000136720	HS6ST1	-0.32	0.001686545	0.005782273
ENSG00000211456	SACM1L	0.43	0.001687842	0.005785729
ENSG00000232860	SMG7-AS1	-2.03	0.00169437	0.005807112
ENSG00000158805	ZNF276	-0.37	0.00169522	0.005809033
ENSG00000105711	SCN1B	0.68	0.001700806	0.005827175

ENSG00000102931	ARL2BP	-0.63	0.0017011	0.005827186
ENSG00000137760	ALKBH8	0.56	0.001705169	0.005840124
ENSG00000188559	RALGAPA2	-0.50	0.001705819	0.00584135
ENSG00000170873	MTSS1	-0.92	0.00170643	0.005842442
ENSG00000184271	POU6F1	-0.89	0.001709222	0.005851
ENSG00000185839	AL035411.1	0.79	0.001712118	0.005859913
ENSG00000187091	PLCD1	-0.82	0.001712454	0.005860059
ENSG00000168438	CDC40	0.39	0.001713698	0.005863315
ENSG00000197785	ATAD3A	0.26	0.001722959	0.005893994
ENSG00000186193	SAPCD2	0.23	0.001725244	0.005900801
ENSG00000267100	ILF3-DT	-0.52	0.001726019	0.005902443
ENSG00000213853	EMP2	-0.36	0.001728025	0.005908294
ENSG00000182117	NOP10	0.24	0.001729709	0.005913039
ENSG00000181722	ZBTB20	1.86	0.001730467	0.005914621
ENSG00000114771	AADAC	1.03	0.001737713	0.005938372
ENSG00000101665	SMAD7	0.66	0.001742768	0.005954631
ENSG00000179562	GCC1	0.30	0.001745604	0.005963301
ENSG00000116095	PLEKHA3	0.39	0.001746138	0.005963416
ENSG00000204524	ZNF805	0.51	0.001746348	0.005963416
ENSG00000169733	RFNG	-0.36	0.001746721	0.005963416
ENSG00000128881	TTBK2	0.52	0.001746829	0.005963416
ENSG00000162545	CAMK2N1	-0.45	0.001748467	0.005967988
ENSG00000124688	MAD2L1BP	0.34	0.001748931	0.0059682
ENSG00000276337	AC105429.1	1.64	0.001749125	0.0059682
ENSG00000102743	SLC25A15	-0.35	0.001751686	0.005975918
ENSG00000059145	UNKL	0.42	0.001752279	0.005976922
ENSG00000066923	STAG3	1.05	0.001762542	0.006010905
ENSG00000171428	NAT1	1.13	0.001768043	0.006028638
ENSG00000273749	CYFIP1	-0.22	0.001771065	0.006036912
ENSG00000184047	DIABLO	0.69	0.001771073	0.006036912
ENSG00000185238	PRMT3	-0.49	0.001773452	0.006043993
ENSG00000104611	SH2D4A	-0.40	0.001777223	0.006055814
ENSG00000151881	TMEM267	-0.50	0.001781398	0.006069005
ENSG00000117425	PTCH2	-1.85	0.001784769	0.006079456
ENSG00000177311	ZBTB38	0.40	0.001791114	0.006100031
ENSG00000156471	PTDSS1	0.25	0.001792844	0.006104882
ENSG00000104889	RNASEH2A	-0.35	0.001796371	0.006115852
ENSG00000260912	AL158206.1	-1.08	0.001797138	0.006117422
ENSG00000100522	GNPNAT1	-0.35	0.001799007	0.006122741
ENSG00000020633	RUNX3	-0.39	0.001805306	0.006142293
ENSG00000077238	IL4R	0.36	0.001805365	0.006142293
ENSG00000133067	LGR6	-2.07	0.001805712	0.006142428
ENSG00000185950	IRS2	-0.51	0.00180646	0.006143928
ENSG00000069399	BCL3	0.54	0.001815792	0.006174617
ENSG00000285108	AC103718.1	-1.18	0.00181696	0.00617754
ENSG00000187954	CYHR1	-0.32	0.001829494	0.0062191

ENSG00000176018	LYSMD3	0.45	0.001832679	0.006228868
ENSG00000185340	GAS2L1	-0.42	0.001837863	0.006245425
ENSG00000165655	ZNF503	0.31	0.00183969	0.006250574
ENSG00000213145	CRIP1	0.94	0.001840992	0.006253935
ENSG00000124588	NQO2	0.24	0.001855687	0.006302784
ENSG00000188610	FAM72B	0.56	0.001857866	0.006309116
ENSG00000119471	HSDL2	-0.40	0.001864681	0.006331185
ENSG00000068438	FTSJ1	-0.32	0.001869388	0.00634609
ENSG00000084733	RAB10	-0.49	0.001874215	0.006361395
ENSG00000175606	TMEM70	0.29	0.001880321	0.006380835
ENSG00000122140	MRPS2	-0.25	0.00188058	0.006380835
ENSG00000160404	TOR2A	-0.61	0.001882708	0.006386972
ENSG00000196155	PLEKHG4	-0.43	0.001883501	0.006387669
ENSG00000140043	PTGR2	-0.81	0.001883552	0.006387669
ENSG00000197747	S100A10	0.43	0.001891034	0.006411958
ENSG00000120068	HOXB8	-0.33	0.00189174	0.006413265
ENSG00000198695	MT-ND6	-0.43	0.001892326	0.006414165
ENSG00000271576	AL359504.1	-1.14	0.001896348	0.00642671
ENSG00000157538	VPS26C	-0.31	0.001898185	0.006431475
ENSG00000042088	TDP1	-0.41	0.001898397	0.006431475
ENSG00000185885	IFITM1	1.91	0.00190127	0.006440119
ENSG00000140534	TICRR	-0.33	0.001903683	0.006447199
ENSG00000123908	AGO2	0.22	0.001907071	0.00645758
ENSG00000105227	PRX	-0.66	0.001907749	0.006458786
ENSG00000104826	LHB	2.17	0.001928543	0.006528078
ENSG00000170776	AKAP13	0.29	0.001928901	0.006528187
ENSG00000140400	MAN2C1	-0.39	0.00192965	0.006529509
ENSG00000215417	MIR17HG	-0.80	0.001929944	0.006529509
ENSG00000143947	RPS27A	-0.21	0.001931427	0.006533421
ENSG00000163832	ELP6	0.34	0.001933017	0.006537695
ENSG00000170448	NFXL1	-0.51	0.001935679	0.006545593
ENSG00000174799	CEP135	0.50	0.001937521	0.006550641
ENSG00000182010	RTKN2	0.46	0.001938072	0.006550641
ENSG00000177464	GPR4	5.72	0.001938154	0.006550641
ENSG00000182400	TRAPPC6B	0.68	0.001949498	0.006587867
ENSG00000160209	PDXK	0.24	0.001952103	0.006595557
ENSG00000148218	ALAD	-0.42	0.00195348	0.006599095
ENSG00000151366	NDUFC2	0.38	0.001956478	0.006608107
ENSG00000104133	SPG11	-0.39	0.001960966	0.006622147
ENSG00000271447	MMP28	-2.48	0.001961702	0.00662333
ENSG00000248275	TRIM52-AS1	-0.75	0.001962196	0.00662333
ENSG00000184588	PDE4B	0.25	0.001962309	0.00662333
ENSG00000114204	SERPINI2	5.72	0.001963509	0.006626263
ENSG00000166398	GARRE1	-0.46	0.001967124	0.006637342
ENSG00000115020	PIKFYVE	0.37	0.001967866	0.006638728
ENSG00000085224	ATRX	0.33	0.001968229	0.006638832

ENSG00000269896	AL513477.1	-1.74	0.001971246	0.006647891
ENSG00000183873	SCN5A	-0.46	0.001971807	0.006648661
ENSG00000255508	AP002990.1	0.59	0.001973342	0.006652716
ENSG00000204673	AKT1S1	0.30	0.001979335	0.006671797
ENSG00000100473	COCH	-1.10	0.00198086	0.00667581
ENSG00000051009	FAM160A2	-0.38	0.001989912	0.006705188
ENSG00000180806	HOXC9	0.60	0.001992311	0.006712143
ENSG00000269951	AC090181.2	-3.59	0.001994324	0.006717193
ENSG00000122873	CISD1	0.40	0.001994481	0.006717193
ENSG00000100577	GSTZ1	-0.40	0.002002722	0.006743812
ENSG00000143771	CNIH4	0.29	0.002003825	0.006746389
ENSG00000105619	TFPT	0.60	0.002006012	0.006752619
ENSG00000108604	SMARCD2	-0.22	0.002006906	0.006754492
ENSG00000171786	NHLH1	1.47	0.002009032	0.006760038
ENSG00000258701	LINC00638	-2.69	0.00200923	0.006760038
ENSG00000169919	GUSB	-0.35	0.002012283	0.006769174
ENSG00000005100	DHX33	-0.32	0.002013922	0.006773549
ENSG00000184983	NDUFA6	0.23	0.002021218	0.006796946
ENSG00000100320	RBFOX2	0.22	0.002022474	0.006800024
ENSG00000144959	NCEH1	0.40	0.002023767	0.006802368
ENSG00000056972	TRAF3IP2	-0.57	0.002023851	0.006802368
ENSG00000005007	UPF1	0.20	0.002024709	0.00680411
ENSG00000196730	DAPK1	-0.85	0.002028179	0.006814627
ENSG00000257135	ODC1-DT	-0.90	0.002029448	0.006817745
ENSG00000170396	ZNF804A	1.88	0.002035772	0.006837844
ENSG00000272822	AC073610.2	2.30	0.002046138	0.006871508
ENSG00000156469	MTERF3	-0.41	0.002049705	0.006882331
ENSG00000198001	IRAK4	-0.64	0.002056072	0.006902552
ENSG00000123095	BHLHE41	1.03	0.002057705	0.006906877
ENSG00000095574	IKZF5	-0.43	0.00206291	0.006923187
ENSG00000081148	IMPG2	1.65	0.002064858	0.006928561
ENSG00000197417	SHPK	0.70	0.002068164	0.006938491
ENSG00000106682	EIF4H	0.17	0.002068956	0.006939984
ENSG00000064601	CTSA	0.26	0.002072557	0.006950897
ENSG00000126218	F10	0.90	0.002073778	0.006953827
ENSG00000099203	TMED1	0.44	0.002077326	0.006964558
ENSG00000180071	ANKRD18A	-0.91	0.002078571	0.006966979
ENSG00000114573	ATP6V1A	0.26	0.002078744	0.006966979
ENSG00000032389	EIPR1	0.30	0.002080298	0.00697102
ENSG00000152409	JMY	-0.44	0.00208412	0.006982657
ENSG00000242616	GNG10	-0.42	0.00208901	0.006997867
ENSG00000124782	RREB1	0.33	0.00209023	0.006999766
ENSG00000175877	TMEM270	1.76	0.002090378	0.006999766
ENSG00000173898	SPTBN2	-0.41	0.002090626	0.006999766
ENSG00000165119	HNRNPK	0.18	0.002094575	0.007011815
ENSG00000267107	PCAT19	2.15	0.002099182	0.007026064

ENSG00000144455	SUMF1	-0.41	0.002099941	0.00702743
ENSG00000144535	DIS3L2	-0.38	0.002105007	0.007043203
ENSG00000149927	DOC2A	-0.51	0.002106938	0.007048487
ENSG00000131791	PRKAB2	0.33	0.002108256	0.007051573
ENSG00000114126	TFDP2	-0.42	0.002108565	0.007051573
ENSG00000117616	RSRP1	-0.38	0.002114112	0.00706894
ENSG00000136522	MRPL47	0.37	0.002120692	0.007089759
ENSG00000100156	SLC16A8	-1.85	0.00212335	0.007097459
ENSG00000177692	DNAJC28	1.30	0.002126781	0.007107738
ENSG00000102309	PIN4	0.52	0.002127162	0.007107827
ENSG00000165801	ARHGEF40	0.47	0.002129351	0.007113953
ENSG00000101868	POLA1	-0.34	0.002137812	0.007141026
ENSG00000205683	DPF3	1.91	0.002139541	0.00714561
ENSG00000074800	ENO1	0.28	0.002141389	0.007150587
ENSG00000128228	SDF2L1	0.34	0.002151899	0.007184484
ENSG00000163050	COQ8A	-0.39	0.002153422	0.007188372
ENSG00000111615	KRR1	-0.28	0.002163545	0.007220957
ENSG00000122783	CYREN	-0.37	0.00216477	0.007223842
ENSG00000160953	PWWP3A	0.26	0.002168712	0.007235788
ENSG00000159231	CBR3	1.32	0.002171529	0.00724398
ENSG00000156958	GALK2	0.64	0.002172378	0.007245604
ENSG00000197599	CCDC154	-3.56	0.002184677	0.007285411
ENSG00000174442	ZWILCH	-0.40	0.002189462	0.00730009
ENSG00000181061	HIGD1A	-0.28	0.002190007	0.00730009
ENSG00000183426	NPIPA1	-0.66	0.002190173	0.00730009
ENSG00000165055	METTL2B	-0.35	0.002196919	0.007321355
ENSG00000283709	FAM238C	-1.96	0.002197652	0.007322578
ENSG00000068903	SIRT2	-0.46	0.002199188	0.007326477
ENSG00000258920	FOXN3-AS1	0.92	0.002203589	0.007339916
ENSG00000167549	CORO6	-0.72	0.002205022	0.007343467
ENSG00000171621	SPSB1	-0.49	0.002206289	0.007346467
ENSG00000285230	RALY-AS1	-1.02	0.002208275	0.007351854
ENSG00000213625	LEPROT	-0.41	0.002214291	0.007370658
ENSG00000111424	VDR	0.42	0.00221814	0.007382242
ENSG00000213080	AL354714.2	0.96	0.002219904	0.007386883
ENSG00000122299	ZC3H7A	0.31	0.002222917	0.00739568
ENSG00000171798	KNDC1	-1.13	0.002224622	0.007400122
ENSG00000155508	CNOT8	0.27	0.002225435	0.007401595
ENSG00000181751	MACIR	-0.47	0.002237282	0.00743976
ENSG00000276007	AC079414.3	-2.54	0.002238249	0.007441741
ENSG00000168496	FEN1	-0.20	0.002240404	0.007447669
ENSG00000109756	RAPGEF2	0.39	0.002242433	0.007453175
ENSG00000143797	MBOAT2	-0.42	0.002244945	0.007460286
ENSG00000173214	MFSD4B	0.49	0.002247181	0.007466478
ENSG00000239672	NME1	0.21	0.002248035	0.007467411
ENSG00000166579	NDEL1	-0.35	0.002248209	0.007467411

ENSG00000099995	SF3A1	-0.22	0.002259568	0.007503896
ENSG00000135624	CCT7	0.20	0.002261565	0.007509281
ENSG00000177570	SAMD12	0.61	0.002268462	0.007530932
ENSG00000126391	FRMD8	-0.35	0.002269947	0.007534611
ENSG00000249395	CASC9	1.19	0.002275134	0.007549549
ENSG00000119508	NR4A3	2.22	0.002275202	0.007549549
ENSG00000160505	NLRP4	-1.16	0.002280538	0.007566001
ENSG00000165209	STRBP	-0.38	0.002281185	0.007566894
ENSG00000272347	AC116351.2	3.21	0.002282935	0.007571443
ENSG00000130762	ARHGEF16	0.35	0.002288744	0.007589452
ENSG00000159184	HOXB13	0.48	0.002291317	0.007596727
ENSG00000141447	OSBPL1A	-0.48	0.00229184	0.007597201
ENSG00000273142	LINC02604	-0.87	0.002292439	0.007597927
ENSG00000170265	ZNF282	-0.33	0.00229983	0.007621163
ENSG00000123374	CDK2	-0.21	0.002312806	0.007661739
ENSG00000140678	ITGAX	1.32	0.002312841	0.007661739
ENSG00000183207	RUVBL2	0.21	0.002315207	0.007668308
ENSG00000169826	CSGALNACT2	0.40	0.002316361	0.00767086
ENSG00000137218	FRS3	-0.81	0.002321426	0.007686363
ENSG00000271976	AC012467.2	-0.96	0.002322174	0.007687568
ENSG00000136100	VPS36	-0.31	0.002329467	0.007710437
ENSG00000242265	PEG10	0.35	0.002337251	0.007734399
ENSG00000215246	AC116351.1	1.35	0.002337709	0.007734399
ENSG00000170017	ALCAM	0.27	0.002337866	0.007734399
ENSG00000276386	CNTNAP3P2	0.94	0.002346172	0.007760594
ENSG00000261499	AC233699.1	1.69	0.002350747	0.007774444
ENSG00000231503	PTMAP4	-1.65	0.002359177	0.007799949
ENSG00000151304	SRFBP1	0.74	0.002359239	0.007799949
ENSG00000185716	MOSMO	-0.40	0.00236515	0.007817902
ENSG00000153827	TRIP12	0.21	0.00236545	0.007817902
ENSG00000134247	PTGFRN	0.22	0.002369269	0.007829229
ENSG00000240668	KRT8P36	2.00	0.002384681	0.007878856
ENSG00000128944	KNSTRN	0.29	0.002388538	0.007889211
ENSG00000128016	ZFP36	0.34	0.002388603	0.007889211
ENSG00000196914	ARHGEF12	-0.22	0.002389502	0.007890876
ENSG00000138709	LARP1B	0.33	0.002395529	0.007909474
ENSG00000174130	TLR6	0.78	0.002401508	0.007927907
ENSG00000000457	SCYL3	-0.57	0.00240432	0.007935882
ENSG00000166562	SEC11C	0.33	0.002408926	0.007949775
ENSG00000124098	FAM210B	-0.52	0.002412306	0.007959617
ENSG00000198208	RPS6KL1	-0.68	0.002413539	0.007962373
ENSG00000108395	TRIM37	0.26	0.002414682	0.00796483
ENSG00000102393	GLA	0.36	0.002418572	0.007976347
ENSG00000153395	LPCAT1	0.21	0.002419537	0.007978214
ENSG00000196850	PPTC7	0.33	0.002430755	0.008013883
ENSG00000134278	SPIRE1	0.48	0.002435991	0.008029823

ENSG00000162595	DIRAS3	1.61	0.002436832	0.00803127
ENSG00000053254	FOXN3	-0.38	0.002438046	0.008033951
ENSG00000178567	EPM2AIP1	-0.48	0.002445497	0.008057177
ENSG00000242485	MRPL20	-0.26	0.002447196	0.008061447
ENSG00000088836	SLC4A11	-0.51	0.00244825	0.00806359
ENSG00000143494	VASH2	0.68	0.002453264	0.008078776
ENSG00000197245	FAM110D	1.41	0.002456569	0.008088329
ENSG00000177406	NINJ2-AS1	-1.15	0.002459159	0.008095523
ENSG00000139436	GIT2	0.43	0.002463872	0.008109706
ENSG00000143727	ACP1	0.23	0.002468901	0.00812492
ENSG00000072415	MPP5	0.33	0.002473056	0.008137255
ENSG00000179388	EGR3	1.63	0.002473535	0.008137495
ENSG00000147912	FBXO10	-0.77	0.002489742	0.008188604
ENSG00000063169	BICRA	-0.62	0.002489889	0.008188604
ENSG00000243323	PTPRVP	2.75	0.002493251	0.008198315
ENSG00000172732	MUS81	0.33	0.002496392	0.008207293
ENSG00000105270	CLIP3	-2.27	0.002502421	0.008224758
ENSG00000205572	SERF1B	1.57	0.002502526	0.008224758
ENSG00000009413	REV3L	-0.42	0.002512054	0.008254716
ENSG00000131446	MGAT1	-0.26	0.002512889	0.008256105
ENSG00000120647	CCDC77	0.52	0.002527703	0.008303413
ENSG00000286507	AC004947.3	3.12	0.002535055	0.008326196
ENSG00000100949	RABGGTA	-0.40	0.002546473	0.008362325
ENSG00000183773	AIFM3	-1.34	0.002549185	0.008369859
ENSG00000248774	AC097534.1	-3.14	0.002553042	0.00838087
ENSG00000084693	AGBL5	0.29	0.002553376	0.00838087
ENSG00000166783	MARF1	0.30	0.00255552	0.008386532
ENSG00000148459	PDSS1	-0.56	0.002557528	0.008391746
ENSG00000153162	BMP6	0.98	0.002573569	0.008442993
ENSG00000267561	AC093155.3	5.64	0.002581251	0.008465695
ENSG00000230027	AC092813.2	5.65	0.002581335	0.008465695
ENSG00000167302	TEPSIN	-0.50	0.002591968	0.008499174
ENSG00000127561	SYNGR3	0.63	0.002596232	0.008511761
ENSG00000286602	AC021660.4	1.07	0.002599723	0.008521811
ENSG00000160145	KALRN	0.79	0.002603299	0.008532134
ENSG00000253981	ALG1L13P	-1.88	0.002609179	0.008550005
ENSG00000196704	AMZ2	-0.26	0.002613062	0.008561329
ENSG00000147799	ARHGAP39	-0.44	0.002613806	0.008562364
ENSG00000128203	ASPHD2	-0.67	0.002616528	0.008569877
ENSG00000133103	COG6	0.53	0.002620427	0.008581245
ENSG00000272808	AC015712.6	-1.70	0.002622284	0.008585922
ENSG00000197191	CYSRT1	0.98	0.002623331	0.008587945
ENSG00000272777	AC019131.2	-3.51	0.002624856	0.008591531
ENSG00000157800	SLC37A3	0.31	0.002631753	0.008612696
ENSG00000124104	SNX21	0.61	0.002635401	0.008623224
ENSG00000129116	PALLD	0.31	0.002637355	0.008628209

ENSG00000237441	RGL2	0.30	0.002637941	0.008628713
ENSG00000166130	IKBIP	0.43	0.002641766	0.008639813
ENSG00000110367	DDX6	-0.23	0.002642344	0.008640293
ENSG00000136104	RNASEH2B	-0.40	0.002650001	0.008663914
ENSG00000147601	TERF1	-0.37	0.002652571	0.008670899
ENSG00000285967	NIPBL-DT	-0.83	0.00265456	0.008675986
ENSG00000089159	PXN	-0.20	0.002662374	0.008700102
ENSG00000176485	PLAAT3	0.39	0.002666233	0.00871129
ENSG00000184792	OSBP2	-0.39	0.002667469	0.008713906
ENSG00000103485	QPRT	3.12	0.002668675	0.008716424
ENSG00000167604	NFKBID	0.79	0.002672179	0.008726443
ENSG00000178096	BOLA1	-0.51	0.002675118	0.008734617
ENSG00000278948	AL031587.5	-1.52	0.0026764	0.008737376
ENSG00000133983	COX16	0.36	0.002677362	0.00873909
ENSG00000144036	EXOC6B	0.32	0.002680154	0.008746777
ENSG00000275322	AC103746.1	2.47	0.002684511	0.008759569
ENSG00000136478	TEX2	-0.34	0.002686455	0.008764482
ENSG00000261236	BOP1	-0.23	0.002703089	0.008817313
ENSG00000196497	IPO4	-0.67	0.002708946	0.008834977
ENSG00000147872	PLIN2	-0.47	0.002715466	0.008854145
ENSG00000158769	F11R	-0.25	0.002715708	0.008854145
ENSG00000110675	ELMOD1	1.15	0.002717429	0.008858315
ENSG00000186283	TOR3A	0.28	0.002718396	0.008860024
ENSG00000226856	THORLNC	-1.75	0.002733738	0.008908576
ENSG00000226380	AC016831.1	0.92	0.002738937	0.008924064
ENSG00000151748	SAV1	0.31	0.002739469	0.008924346
ENSG00000175155	YPEL2	0.70	0.002741995	0.00893112
ENSG00000263934	SNORD3A	2.90	0.002749959	0.008955604
ENSG00000088256	GNA11	0.20	0.00275962	0.008985603
ENSG00000096060	FKBP5	-0.24	0.002762584	0.008993792
ENSG00000029363	BCLAF1	-0.24	0.00276572	0.009002537
ENSG00000103657	HERC1	-0.43	0.002772588	0.009023424
ENSG00000165660	ABRAXAS2	0.38	0.002783453	0.009057309
ENSG00000159307	SCUBE1	0.75	0.002787107	0.009067725
ENSG00000184731	FAM110C	0.42	0.002787908	0.009068858
ENSG00000150938	CRIM1	0.32	0.002789838	0.00907366
ENSG00000203721	LINC00862	2.91	0.002792176	0.009079788
ENSG00000065911	MTHFD2	-0.20	0.002795162	0.009088024
ENSG00000135437	RDH5	-2.71	0.002799496	0.009100637
ENSG00000170091	NSG2	5.63	0.002803435	0.009111961
ENSG00000275234	AC010503.5	-0.73	0.002810516	0.009133493
ENSG00000160325	CACFD1	0.61	0.002811705	0.009135871
ENSG00000177034	MTX3	-0.32	0.002815704	0.00914738
ENSG00000146426	TIAM2	0.64	0.002822861	0.009169145
ENSG00000148346	LCN2	-3.47	0.002826114	0.009178219
ENSG00000169871	TRIM56	-0.29	0.002827855	0.009182383

ENSG00000149084	HSD17B12	0.29	0.002831715	0.009193426
ENSG00000037757	MRI1	-0.54	0.002832396	0.009194144
ENSG00000106123	EPHB6	0.51	0.002836748	0.009206779
ENSG00000176248	ANAPC2	-0.30	0.002848002	0.009241807
ENSG00000116918	TSNAX	0.35	0.002854509	0.009259969
ENSG00000251141	MRPS30-DT	0.88	0.002854525	0.009259969
ENSG00000144224	UBXN4	0.24	0.002859035	0.009272268
ENSG00000013441	CLK1	0.33	0.002859243	0.009272268
ENSG00000131943	C19orf12	0.45	0.002870914	0.009308607
ENSG00000144712	CAND2	-3.14	0.002873213	0.009314552
ENSG00000198952	SMG5	0.21	0.002875371	0.00932004
ENSG00000135185	TMEM243	-0.50	0.002878708	0.009329345
ENSG00000184863	RBM33	-0.28	0.002883206	0.009342411
ENSG00000118418	HMGN3	0.32	0.002895366	0.009380292
ENSG00000254685	FPGT	-0.73	0.002897043	0.009383176
ENSG00000139168	ZCRB1	0.31	0.002897194	0.009383176
ENSG00000164651	SP8	1.66	0.002900777	0.009393261
ENSG00000108379	WNT3	0.51	0.002902572	0.009397376
ENSG00000232533	AC093673.1	0.77	0.002902987	0.009397376
ENSG00000198408	OGA	-0.19	0.002917297	0.009442173
ENSG00000077454	LRCH4	-0.57	0.002917954	0.009442772
ENSG00000155428	TRIM74	-2.57	0.00292177	0.009453592
ENSG00000107882	SUFU	-0.33	0.002929018	0.00947551
ENSG00000117281	CD160	-3.17	0.002930002	0.00947716
ENSG00000213463	SYNJ2BP	-0.44	0.002933831	0.009488015
ENSG00000180900	SCRIB	-0.20	0.002945741	0.009524991
ENSG00000272482	AC254633.1	-1.73	0.00294817	0.009531305
ENSG00000139734	DIAPH3	0.34	0.002948745	0.009531626
ENSG00000204843	DCTN1	-0.21	0.002969381	0.009596779
ENSG00000215190	LINC00680	-1.57	0.002971552	0.009602246
ENSG00000117601	SERPINC1	2.45	0.002977383	0.009619533
ENSG00000028528	SNX1	-0.24	0.00297787	0.009619553
ENSG00000164111	ANXA5	0.18	0.00298647	0.009644081
ENSG00000234745	HLA-B	0.29	0.002986847	0.009644081
ENSG00000163512	AZI2	0.58	0.002986908	0.009644081
ENSG00000105852	PON3	-1.36	0.002988541	0.009647795
ENSG00000141503	MINK1	0.23	0.002989333	0.009648796
ENSG00000178464	RPL10P16	-0.28	0.002991881	0.009655464
ENSG00000203896	LIME1	-0.99	0.002994947	0.0096638
ENSG00000233589	AL138789.1	-0.62	0.003000313	0.009679551
ENSG00000165917	RAPSN	1.82	0.003002978	0.009686588
ENSG00000225151	GOLGA2P7	-1.18	0.003004685	0.009690532
ENSG00000141012	GALNS	-0.36	0.003015294	0.009723182
ENSG00000181555	SETD2	0.32	0.003018708	0.009731532
ENSG00000070669	ASNS	-0.37	0.003019011	0.009731532
ENSG00000124743	KLHL31	1.23	0.003019343	0.009731532

ENSG00000179950	PUF60	0.21	0.003020522	0.009733765
ENSG00000134138	MEIS2	0.61	0.003023396	0.009741457
ENSG00000060762	MPC1	-0.62	0.003027029	0.009751593
ENSG00000167182	SP2	-0.39	0.003028638	0.009755207
ENSG00000213139	CRYGS	-1.06	0.003032153	0.009764957
ENSG00000078674	PCM1	0.28	0.003033962	0.00976921
ENSG00000169403	PTAFR	-1.50	0.003037603	0.009779361
ENSG00000177971	IMP3	-0.31	0.003038533	0.009780781
ENSG00000167011	NAT16	-0.99	0.003043792	0.009796135
ENSG00000135638	EMX1	-1.06	0.003056326	0.009834894
ENSG00000185104	FAF1	0.28	0.003062663	0.009853698
ENSG00000112782	CLIC5	2.68	0.003067679	0.009868251
ENSG00000167283	ATP5MG	0.24	0.003071675	0.009879519
ENSG00000278730	AC005332.6	0.33	0.003073935	0.009885197
ENSG00000141580	WDR45B	0.19	0.003077608	0.009895419
ENSG00000057608	GDI2	0.20	0.003084208	0.009915049
ENSG00000166260	COX11	-0.30	0.003089802	0.009931437
ENSG00000158526	TSR2	0.29	0.003097996	0.009956175
ENSG00000142303	ADAMTS10	-0.96	0.003104659	0.009975985
ENSG00000100105	PATZ1	0.32	0.003106143	0.00997915
ENSG00000000419	DPM1	-0.30	0.003116768	0.010011678
ENSG00000135441	BLOC1S1	0.37	0.003133488	0.010063773
ENSG00000148335	NTMT1	0.27	0.003136018	0.010070282
ENSG00000089818	NECAP1	0.26	0.003143734	0.010093438
ENSG00000132305	IMMT	-0.23	0.003151109	0.010115495
ENSG00000112651	MRPL2	-0.40	0.003153111	0.010119502
ENSG00000223573	TINCR	0.71	0.003153569	0.010119502
ENSG00000113108	APBB3	-0.51	0.003153874	0.010119502
ENSG00000096063	SRPK1	-0.30	0.003166536	0.010158499
ENSG00000204130	RUFY2	0.55	0.003172453	0.010175851
ENSG00000261141	AC092718.5	-3.42	0.0031836	0.010209365
ENSG00000163607	GTPBP8	0.53	0.003183922	0.010209365
ENSG00000108511	HOXB6	-0.34	0.003184759	0.010210412
ENSG00000134330	IAH1	0.52	0.003211417	0.01029423
ENSG00000070831	CDC42	0.17	0.003219827	0.010317381
ENSG00000006607	FARP2	0.39	0.003220179	0.010317381
ENSG00000121966	CXCR4	1.75	0.003220186	0.010317381
ENSG00000123104	ITPR2	0.52	0.003225169	0.010331693
ENSG00000068489	PRR11	0.25	0.003226416	0.010334032
ENSG00000034693	PEX3	0.50	0.00322759	0.010336137
ENSG00000065491	TBC1D22B	-0.44	0.003233381	0.010352224
ENSG00000165113	GKAP1	0.77	0.003233647	0.010352224
ENSG00000102226	USP11	0.24	0.003249273	0.010400584
ENSG00000226419	SLC16A1-AS1	-1.07	0.003252609	0.010409597
ENSG00000186451	SPATA12	1.50	0.003254794	0.010414062
ENSG00000258890	CEP95	-0.28	0.003255045	0.010414062

ENSG00000179862	CITED4	0.97	0.003261269	0.010432307
ENSG00000131013	PPIL4	0.37	0.00326201	0.010433011
ENSG00000173064	HECTD4	0.34	0.003263526	0.010436192
ENSG00000076650	GPATCH1	0.55	0.00326546	0.010440706
ENSG00000160753	RUSC1	-0.28	0.00327063	0.010455566
ENSG00000261455	LINC01003	-1.12	0.003273567	0.010463284
ENSG00000135052	GOLM1	0.21	0.003275373	0.010467386
ENSG00000051341	POLQ	-0.39	0.003277437	0.010472307
ENSG00000135778	NTPCR	-0.39	0.003287213	0.010501868
ENSG00000181045	SLC26A11	0.47	0.003301437	0.010545626
ENSG00000116661	FBXO2	0.24	0.003303405	0.010550229
ENSG00000275632	AL035461.2	-1.88	0.003325259	0.010618331
ENSG00000241685	ARPC1A	-0.30	0.003328609	0.010627198
ENSG00000106244	PDAP1	0.22	0.003329098	0.010627198
ENSG00000122477	LRRC39	1.55	0.003332161	0.01063528
ENSG00000113163	CERT1	0.31	0.003337985	0.010652169
ENSG00000105085	MED26	0.52	0.003360114	0.010721079
ENSG00000082014	SMARCD3	-0.33	0.003363856	0.010731307
ENSG00000137776	SLTM	0.23	0.00337037	0.010750374
ENSG00000147905	ZCCHC7	-0.43	0.003382194	0.01078637
ENSG00000242021	AC112493.1	1.29	0.003393237	0.010819862
ENSG00000243789	JMJD7	1.98	0.003395383	0.010824982
ENSG00000169410	PTPN9	-0.31	0.003400248	0.010838765
ENSG00000108932	SLC16A6	0.72	0.003408603	0.010863198
ENSG00000149636	DSN1	-0.31	0.003408999	0.010863198
ENSG00000160993	ALKBH4	0.45	0.003410542	0.010866386
ENSG00000090061	CCNK	-0.41	0.003419801	0.010894152
ENSG00000111596	CNOT2	0.32	0.003420726	0.010895363
ENSG00000153291	SLC25A27	-0.88	0.003422584	0.010899545
ENSG00000159055	MIS18A	0.32	0.003423469	0.010900629
ENSG00000163946	TASOR	0.30	0.003424573	0.01090241
ENSG00000123143	PKN1	-0.19	0.003427377	0.010909603
ENSG00000011028	MRC2	0.38	0.003429521	0.01091455
ENSG00000149600	COMMD7	0.47	0.003430022	0.01091455
ENSG00000005810	MYCBP2	-0.32	0.003436463	0.010933305
ENSG00000274561	AC005332.3	-1.73	0.003446565	0.010963704
ENSG00000180957	PITPNB	0.24	0.003451408	0.010975715
ENSG00000180964	TCEAL8	0.32	0.003451438	0.010975715
ENSG00000133639	BTG1	-0.31	0.003454485	0.010983658
ENSG00000168646	AXIN2	0.39	0.0034647	0.011014389
ENSG00000157823	AP3S2	-0.45	0.003478439	0.011056308
ENSG00000273367	AL355472.3	-5.58	0.003495144	0.011107644
ENSG00000110987	BCL7A	-0.43	0.003507415	0.011144869
ENSG00000155827	RNF20	-0.35	0.003509332	0.011149192
ENSG00000144579	CTDSP1	-0.30	0.003510214	0.011150223
ENSG00000147649	MTDH	0.19	0.003518621	0.011174204

ENSG00000165863	C10orf82	3.86	0.00351888	0.011174204
ENSG00000123342	MMP19	1.36	0.003523309	0.011186491
ENSG00000183647	ZNF530	0.77	0.003527855	0.011199149
ENSG00000272695	GAS6-DT	1.34	0.003529074	0.011201241
ENSG00000132153	DHX30	0.20	0.003532495	0.011210323
ENSG00000089094	KDM2B	-0.29	0.003533591	0.01121202
ENSG00000127366	TAS2R5	-3.90	0.00353922	0.011228102
ENSG00000136999	CCN3	1.24	0.003540659	0.011229382
ENSG00000241818	SCG5-AS1	2.65	0.003540746	0.011229382
ENSG00000116273	PHF13	-0.29	0.003543213	0.011235428
ENSG00000204237	OXLD1	-0.30	0.00354558	0.011241151
ENSG00000158062	UBXN11	-0.49	0.003548166	0.011247569
ENSG00000158748	HTR6	-2.18	0.003549639	0.011250457
ENSG00000175395	ZNF25	-1.18	0.003551744	0.011255343
ENSG00000111726	CMAS	0.28	0.003553663	0.011259644
ENSG00000221968	FADS3	-0.29	0.003557764	0.011270371
ENSG00000077235	GTF3C1	-0.21	0.003558502	0.011270371
ENSG00000146676	PURB	0.30	0.003558738	0.011270371
ENSG00000181817	LSM10	-0.37	0.003578226	0.011330295
ENSG00000234160	AL513165.1	-0.67	0.00358187	0.01134004
ENSG00000199024	MIR103A2	5.55	0.003588142	0.011358098
ENSG00000176396	EID2	-0.41	0.003598441	0.011388898
ENSG00000270055	AC127502.2	-1.38	0.003603758	0.01140382
ENSG00000015153	YAF2	0.55	0.003604295	0.01140382
ENSG00000166592	RRAD	1.00	0.003606431	0.011408772
ENSG00000167759	KLK13	-2.01	0.003617859	0.011443115
ENSG00000176542	USF3	-0.43	0.003622507	0.011456006
ENSG00000268087	AC008764.2	-1.30	0.003626959	0.011468272
ENSG00000016402	IL20RA	-1.66	0.003630474	0.011477574
ENSG00000106991	ENG	-1.92	0.003634802	0.011489441
ENSG00000120675	DNAJC15	0.42	0.003635995	0.011491399
ENSG00000250506	CDK3	-1.67	0.003638634	0.011497922
ENSG00000137040	RANBP6	0.35	0.003639295	0.011498196
ENSG00000139350	NEDD1	0.36	0.003650784	0.011532674
ENSG00000100979	PLTP	-0.77	0.003654463	0.011541395
ENSG00000228022	HCG20	3.36	0.003654698	0.011541395
ENSG00000154358	OBSCN	0.41	0.003657152	0.01154732
ENSG00000122958	VPS26A	0.25	0.003658884	0.011549232
ENSG00000288065	AC007881.4	-2.14	0.003658911	0.011549232
ENSG00000144730	IL17RD	-0.41	0.003662042	0.011557291
ENSG00000074855	ANO8	0.38	0.003667166	0.011571638
ENSG00000126062	TMEM115	-0.38	0.003670841	0.011581409
ENSG00000146282	RARS2	0.38	0.00367609	0.011596141
ENSG00000236671	PRKG1-AS1	1.15	0.003685841	0.011625068
ENSG00000117133	RPF1	0.30	0.003689376	0.011633829
ENSG00000196110	ZNF699	0.57	0.003689782	0.011633829

ENSG00000103160	HSDL1	0.26	0.003692459	0.011640435
ENSG00000173848	NET1	0.26	0.00369414	0.0116439
ENSG00000256576	LINC02361	0.84	0.00369538	0.011645975
ENSG00000198814	GK	-0.88	0.003698812	0.011653302
ENSG00000119737	GPR75	1.57	0.003698869	0.011653302
ENSG00000148842	CNNM2	0.32	0.003700543	0.01165674
ENSG00000204178	MACO1	0.47	0.003702163	0.011660008
ENSG00000135341	MAP3K7	0.31	0.003703416	0.011662119
ENSG00000124557	BTN1A1	2.66	0.003723902	0.011724785
ENSG00000122779	TRIM24	0.43	0.003724605	0.011725153
ENSG00000212724	KRTAP2-3	-1.06	0.003735122	0.011756413
ENSG00000124217	MOCS3	-0.46	0.003742731	0.011778507
ENSG00000171603	CLSTN1	0.18	0.003744721	0.011782337
ENSG00000165458	INPPL1	-0.24	0.003745125	0.011782337
ENSG00000141337	ARSG	-0.67	0.003751562	0.011800732
ENSG00000114030	KPNA1	0.28	0.003760093	0.01182571
ENSG00000108861	DUSP3	-0.28	0.003769953	0.011854854
ENSG00000185187	SIGIRR	0.37	0.003777206	0.011875796
ENSG00000247137	AP000873.2	0.97	0.00378272	0.011891263
ENSG00000136156	ITM2B	0.25	0.003798861	0.011939843
ENSG00000165733	BMS1	0.22	0.003799366	0.011939843
ENSG00000213742	ZNF337-AS1	-1.55	0.00380214	0.011946682
ENSG00000197416	FABP12	5.55	0.003805213	0.011954462
ENSG00000167842	MIS12	-0.41	0.003815433	0.011984689
ENSG00000184216	IRAK1	-0.22	0.003819218	0.011994693
ENSG00000103326	CAPN15	-0.35	0.003820494	0.011996818
ENSG00000138182	KIF20B	0.34	0.003821574	0.011998329
ENSG00000088356	PDRG1	0.28	0.003830252	0.012023686
ENSG00000250420	AACSP1	-1.08	0.003853686	0.012095353
ENSG00000073921	PICALM	0.26	0.003860967	0.012116304
ENSG00000198169	ZNF251	-0.45	0.003867113	0.012133689
ENSG00000171466	ZNF562	-0.38	0.003878761	0.012165985
ENSG00000166797	CIAO2A	0.28	0.003879019	0.012165985
ENSG00000164346	NSA2	0.24	0.00387923	0.012165985
ENSG00000204054	LINC00963	-0.27	0.003901384	0.012233548
ENSG00000130699	TAF4	-0.38	0.003904029	0.012239924
ENSG00000172915	NBEA	0.71	0.00392825	0.012312408
ENSG00000143344	RGL1	0.90	0.003928379	0.012312408
ENSG00000159461	AMFR	0.23	0.003936929	0.012337272
ENSG00000105339	DENND3	0.40	0.003937583	0.012337391
ENSG00000156711	MAPK13	0.25	0.003940386	0.012341372
ENSG00000105640	RPL18A	-0.22	0.003940474	0.012341372
ENSG00000136193	SCRN1	-0.22	0.003940917	0.012341372
ENSG00000177683	THAP5	-0.39	0.003941321	0.012341372
ENSG00000177854	TMEM187	-0.62	0.003942248	0.012342344
ENSG00000188112	C6orf132	-0.44	0.00394397	0.012345806

ENSG00000134243	SORT1	0.26	0.003950602	0.01236463
ENSG00000166974	MAPRE2	0.34	0.003952799	0.012369571
ENSG00000186312	CA5BP1	0.62	0.003958561	0.012385666
ENSG00000167992	VWCE	-1.38	0.003979219	0.012448356
ENSG00000101132	PFDN4	0.42	0.003987624	0.012472699
ENSG00000236714	LINC01844	-2.53	0.003988775	0.012474351
ENSG00000125844	RRBP1	0.18	0.003991026	0.012479438
ENSG00000132423	COQ3	-0.44	0.003992422	0.012481856
ENSG00000173726	TOMM20	-0.19	0.003996256	0.012491888
ENSG00000106853	PTGR1	0.28	0.004004193	0.01251114
ENSG00000274849	AC023043.4	-2.41	0.004004206	0.01251114
ENSG00000074657	ZNF532	0.42	0.00400429	0.01251114
ENSG00000143553	SNAPIN	0.31	0.00401701	0.012548923
ENSG00000165591	FAAH2	0.80	0.004024965	0.012571811
ENSG00000037042	TUBG2	0.41	0.004026644	0.012575095
ENSG00000267858	MZF1-AS1	-0.87	0.004035547	0.012600496
ENSG00000056097	ZFR	0.27	0.004036037	0.012600496
ENSG00000125962	ARMCX5	-0.68	0.004040483	0.012612409
ENSG00000169189	NSMCE1	-0.27	0.004044735	0.012623711
ENSG00000183032	SLC25A21	1.14	0.004048298	0.01263286
ENSG00000170889	RPS9	-0.20	0.00405484	0.012651303
ENSG00000148672	GLUD1	0.22	0.004057075	0.012656303
ENSG00000185630	PBX1	-0.80	0.004064847	0.012678573
ENSG00000157954	WIPI2	-0.23	0.004071571	0.012697565
ENSG00000138821	SLC39A8	0.41	0.004073811	0.01270257
ENSG00000268471	MIR4453HG	-1.16	0.004080819	0.01272244
ENSG00000120688	WBP4	0.35	0.004087384	0.012740924
ENSG00000129295	LRRC6	1.22	0.004094305	0.012760511
ENSG00000186314	PRELID2	-0.62	0.004101292	0.012780295
ENSG00000112357	PEX7	-0.67	0.004106219	0.012793657
ENSG00000075702	WDR62	0.28	0.004125044	0.012850309
ENSG00000237187	NR2F1-AS1	0.42	0.004133611	0.012874992
ENSG00000214425	LRRC37A4P	0.65	0.004141264	0.01289682
ENSG00000162188	GNG3	-1.80	0.004142571	0.012898884
ENSG00000182796	TMEM198B	-0.65	0.004143729	0.012900484
ENSG00000197275	RAD54B	-0.67	0.004144895	0.012902105
ENSG00000249740	OSMR-AS1	1.64	0.004148731	0.012912038
ENSG00000286289	AL133482.1	4.89	0.004160033	0.012945199
ENSG00000138495	COX17	0.28	0.004161854	0.012948852
ENSG00000198218	QRICH1	0.23	0.004178215	0.012997738
ENSG00000142961	MOB3C	0.60	0.004179258	0.012998961
ENSG00000282304	AC140479.4	-1.74	0.004190859	0.013033019
ENSG00000104936	DMPK	-0.27	0.004192416	0.013035833
ENSG00000196371	FUT4	-0.46	0.004195304	0.013042787
ENSG00000196208	GREB1	-0.61	0.004216371	0.013106246
ENSG00000170144	HNRNPA3	-0.16	0.004218913	0.013112113

ENSG00000132541	RIDA	-0.43	0.004227362	0.013136331
ENSG00000109929	SC5D	0.34	0.004234248	0.01315409
ENSG00000037474	NSUN2	-0.20	0.004234392	0.01315409
ENSG00000204514	ZNF814	-0.65	0.004235701	0.013156116
ENSG00000114737	CISH	0.82	0.004254028	0.013210989
ENSG00000125375	DMAC2L	-0.87	0.00425772	0.013220403
ENSG00000013288	MAN2B2	-0.29	0.004258961	0.013222204
ENSG00000151353	TMEM18	-0.35	0.004275224	0.013270636
ENSG00000132661	NXT1	-0.36	0.004286679	0.013304012
ENSG00000145832	SLC25A48	-1.61	0.004287306	0.013304012
ENSG00000242396	AC096536.2	2.24	0.004291049	0.013313561
ENSG00000131914	LIN28A	0.99	0.004293344	0.013318616
ENSG00000183020	AP2A2	0.23	0.004294819	0.013321129
ENSG00000224635	AL391095.1	-2.64	0.004295509	0.013321204
ENSG00000018510	AGPS	-0.27	0.004297251	0.013324541
ENSG00000240771	ARHGEF25	1.17	0.004304613	0.013343314
ENSG00000196656	AC004057.1	-0.27	0.004304639	0.013343314
ENSG00000198807	PAX9	0.78	0.004309121	0.013355139
ENSG00000139767	SRRM4	3.36	0.004317818	0.013380019
ENSG00000186907	RTN4RL2	-0.44	0.004322333	0.013391939
ENSG00000125912	NCLN	-0.23	0.004325265	0.013398949
ENSG00000169964	TMEM42	-0.56	0.004326353	0.013400244
ENSG00000145365	TIFA	-0.37	0.004341428	0.013444857
ENSG00000253250	C8orf88	5.48	0.004344113	0.013449008
ENSG00000260314	MRC1	5.48	0.004344113	0.013449008
ENSG00000119227	PIGZ	-1.75	0.004348309	0.013459915
ENSG00000196182	STK40	-0.29	0.004378328	0.013550742
ENSG00000186891	TNFRSF18	0.38	0.004385285	0.013570176
ENSG00000112877	CEP72	-0.47	0.004392798	0.013591323
ENSG00000079999	KEAP1	0.22	0.004395434	0.013597376
ENSG00000264522	OTUD7B	0.27	0.004396701	0.013599175
ENSG00000233429	HOTAIRM1	-0.60	0.004397425	0.013599175
ENSG00000224063	AC007319.1	1.53	0.004398054	0.013599175
ENSG00000109586	GALNT7	0.35	0.004401284	0.01360706
ENSG00000170899	GSTA4	-1.93	0.004405723	0.01361868
ENSG00000174106	LEMD3	0.29	0.004411771	0.013635269
ENSG00000110455	ACCS	-0.71	0.00442042	0.01365989
ENSG00000160446	ZDHHC12	-0.39	0.004426782	0.013677439
ENSG00000136158	SPRY2	0.62	0.004433992	0.013697599
ENSG00000153944	MSI2	-0.22	0.004441926	0.013719993
ENSG00000160741	CRTC2	0.31	0.004443832	0.013723761
ENSG00000135625	EGR4	3.32	0.00445455	0.013754739
ENSG00000099800	TIMM13	0.47	0.004471736	0.013805675
ENSG00000174586	ZNF497	-2.49	0.004478669	0.013824946
ENSG00000164778	EN2	1.55	0.004497218	0.013880063
ENSG00000159147	DONSON	-0.34	0.004504063	0.013899046

ENSG00000078403	MLLT10	0.30	0.004538174	0.014002071
ENSG00000006015	REX1BD	0.31	0.004538848	0.014002071
ENSG00000015676	NUDCD3	-0.23	0.004543153	0.014013191
ENSG00000239920	AC104389.5	-0.75	0.004545824	0.014019268
ENSG00000196220	SRGAP3	-0.76	0.004559595	0.014059132
ENSG00000116809	ZBTB17	0.34	0.004560155	0.014059132
ENSG00000127948	POR	-0.24	0.004564163	0.01406932
ENSG00000188554	NBR1	0.23	0.004566713	0.014075012
ENSG00000005893	LAMP2	0.27	0.004575484	0.014099875
ENSG00000270277	AC009948.1	-5.50	0.004579505	0.014110094
ENSG00000085377	PREP	-0.24	0.004580369	0.014110582
ENSG00000138381	ASNSD1	-0.43	0.004585587	0.014124483
ENSG00000265678	AC129510.1	-1.19	0.004607428	0.014189574
ENSG00000171522	PTGER4	1.10	0.00461319	0.014205132
ENSG00000104412	EMC2	0.35	0.004620417	0.014225196
ENSG00000271851	AC087501.4	-2.11	0.00462941	0.014250692
ENSG00000158106	RHPN1	-0.37	0.004645744	0.014298773
ENSG00000240457	RN7SL472P	5.49	0.004647304	0.014301375
ENSG00000198863	RUNDC1	0.37	0.004660177	0.014338785
ENSG00000011114	BTBD7	0.33	0.004661119	0.014339478
ENSG00000170515	PA2G4	0.18	0.004667163	0.014355865
ENSG00000168781	PPIP5K1	-0.39	0.004669524	0.014360276
ENSG00000263465	SRSF8	-0.30	0.004670032	0.014360276
ENSG00000152475	ZNF837	-1.67	0.004678762	0.014384911
ENSG00000253649	PRSS51	1.70	0.004681557	0.014391295
ENSG00000157353	FCSK	-0.49	0.004685929	0.014402519
ENSG00000144161	ZC3H8	-0.49	0.00469242	0.014420255
ENSG00000099949	LZTR1	-0.41	0.004694005	0.014422913
ENSG00000182612	TSPAN10	0.93	0.004700255	0.0144399
ENSG00000181991	MRPS11	-0.35	0.004709804	0.014467016
ENSG00000165424	ZCCHC24	-0.45	0.004713833	0.014477168
ENSG00000196205	EEF1A1P5	-0.45	0.004717129	0.014485068
ENSG00000232093	DCST1-AS1	-2.28	0.004727117	0.01451351
ENSG00000147400	CETN2	-0.40	0.004737603	0.014542404
ENSG00000264281	AC016596.2	-0.82	0.004737981	0.014542404
ENSG00000060491	OGFR	-0.23	0.004746822	0.014567305
ENSG00000197948	FCHSD1	0.36	0.004752758	0.014583287
ENSG00000126217	MCF2L	-0.47	0.004778498	0.014660019
ENSG00000140157	NIPA2	0.25	0.004782541	0.014670173
ENSG00000270800	RPS10-NUDT3	0.51	0.004803661	0.0147327
ENSG00000055211	GINM1	0.30	0.004805641	0.014736513
ENSG00000058404	CAMK2B	2.54	0.00480705	0.014738576
ENSG00000143341	HMCN1	1.64	0.00481193	0.01475128
ENSG00000181652	ATG9B	1.10	0.004828062	0.014798464
ENSG00000119523	ALG2	-0.32	0.004833224	0.014812019
ENSG00000197405	C5AR1	-0.98	0.004838357	0.014825477

ENSG00000134001	EIF2S1	0.20	0.0048557	0.014876344
ENSG00000168872	DDX19A	0.32	0.004870126	0.014918256
ENSG00000170425	ADORA2B	0.26	0.004877433	0.01493835
ENSG00000184307	ZDHHC23	-0.40	0.00488087	0.01494659
ENSG00000133250	ZNF414	0.49	0.004882804	0.014950225
ENSG00000167779	IGFBP6	0.31	0.004885569	0.014956404
ENSG00000060138	YBX3	-0.18	0.004892201	0.014974414
ENSG00000105647	PIK3R2	-1.41	0.004896525	0.014985359
ENSG00000206562	METTL6	0.43	0.004900119	0.014994065
ENSG00000173209	AHSA2P	0.33	0.004902286	0.014995921
ENSG00000163947	ARHGEF3	0.39	0.004902546	0.014995921
ENSG00000163806	SPDYA	1.37	0.004903688	0.014995921
ENSG00000151876	FBXO4	-0.71	0.004903723	0.014995921
ENSG00000125352	RNF113A	0.56	0.004911037	0.015015994
ENSG00000123684	LPGAT1	-0.26	0.004919571	0.015039787
ENSG00000269906	AL606834.1	-2.19	0.004920602	0.015040642
ENSG00000133083	DCLK1	5.49	0.004924355	0.015049814
ENSG00000117984	CTSD	0.65	0.004932426	0.01507218
ENSG00000125409	TEKT3	3.00	0.00494558	0.015110068
ENSG00000129219	PLD2	-0.30	0.004946451	0.015110421
ENSG00000163795	ZNF513	0.41	0.004948519	0.015114433
ENSG00000279821	AC145098.2	-1.54	0.004950262	0.015117327
ENSG00000269958	AL049840.6	-0.67	0.004950977	0.015117327
ENSG00000167468	GPX4	0.22	0.004964171	0.015155299
ENSG00000155097	ATP6V1C1	0.23	0.004976066	0.015189297
ENSG00000156521	TYSND1	-0.52	0.004977771	0.015192183
ENSG00000107551	RASSF4	1.06	0.004990083	0.015227437
ENSG00000168734	PKIG	0.39	0.004994834	0.015239614
ENSG00000247708	STX18-AS1	-0.82	0.004997179	0.015244444
ENSG00000013561	RNF14	0.34	0.005006229	0.015269724
ENSG00000166394	CYB5R2	0.45	0.005017626	0.015302152
ENSG00000138347	MYPN	2.46	0.005025647	0.01532428
ENSG00000249631	AC005699.1	-1.44	0.005041355	0.015369834
ENSG00000165476	REEP3	-0.33	0.005044148	0.015376007
ENSG00000263731	AC145207.5	-0.72	0.005051209	0.015395187
ENSG00000176222	ZNF404	2.79	0.005063189	0.01542935
ENSG00000257285	AL132780.1	-1.24	0.005073209	0.015457532
ENSG00000174851	YIF1A	-0.31	0.005078104	0.015468338
ENSG00000204371	EHMT2	-0.23	0.005078302	0.015468338
ENSG00000108375	RNF43	0.26	0.005089811	0.015501035
ENSG00000125827	TMX4	0.37	0.005099543	0.01552831
ENSG00000134864	GGACT	-0.98	0.005101521	0.015530119
ENSG00000140416	TPM1	0.42	0.005101689	0.015530119
ENSG00000141526	SLC16A3	0.19	0.00511735	0.015575424
ENSG00000038532	CLEC16A	0.31	0.005134567	0.015624605
ENSG00000125730	C3	1.52	0.005135393	0.015624605

ENSG00000163312	HELQ	0.58	0.005135851	0.015624605
ENSG00000284862	CCDC39	-2.77	0.005139612	0.015633671
ENSG00000221838	AP4M1	-0.43	0.005167201	0.015715202
ENSG00000166352	IFTAP	0.70	0.005171298	0.015725273
ENSG00000143442	POGZ	0.22	0.00517757	0.015741953
ENSG00000164684	ZNF704	-0.40	0.005186728	0.015765383
ENSG00000204560	DHX16	0.23	0.005186852	0.015765383
ENSG00000233916	ZDHHC20P1	3.03	0.005200058	0.015803123
ENSG00000269293	ZSCAN16-AS1	0.83	0.005201091	0.015803861
ENSG00000136816	TOR1B	0.33	0.005203769	0.0158096
ENSG00000254860	TMEM9B-AS1	-0.76	0.005227008	0.015877791
ENSG00000105520	PLPPR2	-0.39	0.005236874	0.015905345
ENSG00000010219	DYRK4	0.44	0.005241327	0.015916455
ENSG00000131849	ZNF132	-1.05	0.00524408	0.015922399
ENSG00000170325	PRDM10	0.50	0.005249646	0.015936881
ENSG00000111752	PHC1	-0.59	0.005264098	0.015978329
ENSG00000181274	FRAT2	-0.79	0.005268029	0.015987838
ENSG00000144026	ZNF514	-0.63	0.005269638	0.015990294
ENSG00000135837	CEP350	0.35	0.005270508	0.015990509
ENSG00000196247	ZNF107	0.52	0.005274813	0.015999417
ENSG00000204272	NBDY	0.29	0.005275043	0.015999417
ENSG00000099817	POLR2E	-0.22	0.005276343	0.016000937
ENSG00000068383	INPP5A	0.37	0.005282032	0.016015762
ENSG00000095209	TMEM38B	-0.34	0.005285869	0.016024968
ENSG00000148399	DPH7	-0.30	0.005295527	0.016051816
ENSG00000109606	DHX15	0.21	0.005304964	0.016077986
ENSG00000089916	GPATCH2L	0.32	0.005313853	0.016102486
ENSG00000163171	CDC42EP3	-0.28	0.005321965	0.016124628
ENSG00000157637	SLC38A10	-0.19	0.005336402	0.016165922
ENSG00000074181	NOTCH3	0.30	0.005338008	0.01616834
ENSG00000182809	CRIP2	0.43	0.005341566	0.016176668
ENSG00000089060	SLC8B1	-0.34	0.005346488	0.016187483
ENSG00000173992	CCS	-0.44	0.005346755	0.016187483
ENSG00000117480	FAAH	0.37	0.005362768	0.016232702
ENSG00000160813	PPP1R35	0.31	0.005363313	0.016232702
ENSG00000131931	THAP1	0.59	0.005373775	0.016261907
ENSG00000107862	GBF1	0.19	0.005382496	0.016285833
ENSG00000198794	SCAMP5	-0.68	0.00538966	0.016305045
ENSG00000105429	MEGF8	-0.27	0.005410323	0.016364756
ENSG00000274070	CASTOR2	0.45	0.005411033	0.016364756
ENSG00000188529	SRSF10	-0.20	0.005415605	0.016376109
ENSG00000170681	CAVIN4	1.24	0.005424706	0.01640115
ENSG00000181350	LRRC75A	0.93	0.005430384	0.016415837
ENSG00000104290	FZD3	-0.54	0.005442065	0.016448664
ENSG00000126749	EMG1	-0.24	0.005458062	0.016493965
ENSG00000113396	SLC27A6	0.45	0.005458701	0.016493965

ENSG00000072182	ASIC4	-2.24	0.00546828	0.016520413
ENSG00000162817	C1orf115	0.27	0.005470796	0.016525521
ENSG00000163053	SLC16A14	-0.50	0.005477415	0.016543015
ENSG00000187122	SLIT1	1.14	0.005479082	0.016545556
ENSG00000167815	PRDX2	-0.23	0.005482912	0.016554623
ENSG00000178460	MCMDC2	-2.16	0.005491902	0.016579263
ENSG00000225377	NRSN2-AS1	-0.91	0.005497321	0.016593121
ENSG00000138594	TMOD3	0.24	0.005518176	0.016653191
ENSG00000146535	GNA12	0.26	0.005518887	0.016653191
ENSG00000129993	CBFA2T3	0.39	0.00552632	0.016673106
ENSG00000185112	FAM43A	0.43	0.005534565	0.016695465
ENSG00000184154	LRTOMT	-0.84	0.005539982	0.016709288
ENSG00000112200	ZNF451	-0.38	0.005543884	0.016718538
ENSG00000231890	DARS-AS1	-2.50	0.00555802	0.016758641
ENSG00000136240	KDELR2	0.20	0.00555919	0.016759642
ENSG00000266904	LINC00663	-2.36	0.005563934	0.016771418
ENSG00000271755	AL031118.1	1.48	0.005568878	0.016783794
ENSG00000248098	BCKDHA	-0.68	0.005573999	0.016796695
ENSG00000229644	NAMPTP1	0.67	0.005575503	0.016796695
ENSG00000216906	AL355312.1	2.26	0.005575677	0.016796695
ENSG00000236200	KDM4A-AS1	0.93	0.005576578	0.01679688
ENSG00000254531	FLJ20021	-0.54	0.005589855	0.01683434
ENSG00000239322	ATP6V1B1-AS1	2.57	0.005594616	0.016845943
ENSG00000105556	MIER2	-0.38	0.005596006	0.016845943
ENSG00000120733	KDM3B	-0.23	0.005596234	0.016845943
ENSG00000114739	ACVR2B	0.33	0.005609387	0.016882997
ENSG00000273891	AL731566.2	-1.68	0.005624737	0.016926651
ENSG00000183397	C19orf71	-1.05	0.005626573	0.01692963
ENSG00000242802	AP5Z1	-0.47	0.005630313	0.016938337
ENSG00000133056	PIK3C2B	-0.45	0.00563154	0.016939484
ENSG00000204536	CCHCR1	-0.30	0.005637193	0.016953939
ENSG00000187676	B3GLCT	-0.60	0.005641001	0.016962841
ENSG00000131778	CHD1L	-0.68	0.005662062	0.017023617
ENSG00000163867	ZMYM6	-0.56	0.005663571	0.017025594
ENSG00000206560	ANKRD28	-0.31	0.005674322	0.01705535
ENSG00000080839	RBL1	-0.38	0.005685269	0.017084176
ENSG00000270020	AC009108.3	-2.21	0.005685619	0.017084176
ENSG00000101187	SLCO4A1	-0.28	0.005711561	0.017159549
ENSG00000243749	TMEM35B	-1.04	0.005713993	0.017164278
ENSG00000075568	TMEM131	-0.21	0.005728405	0.017204988
ENSG00000182359	KBTBD3	0.84	0.005737031	0.017226934
ENSG00000141560	FN3KRP	-0.25	0.005737433	0.017226934
ENSG00000198081	ZBTB14	-0.69	0.005745312	0.017248003
ENSG00000006283	CACNA1G	0.63	0.005760859	0.017292083
ENSG00000166002	SMCO4	-0.51	0.005762825	0.017295388
ENSG00000128513	POT1	0.41	0.005766413	0.017303562

ENSG00000116213	WRAP73	-0.28	0.005787059	0.017362913
ENSG00000172403	SYNPO2	1.20	0.005799667	0.017398131
ENSG00000267764	AC093567.1	5.40	0.005800955	0.017399388
ENSG00000248641	HMGA1P2	-3.80	0.005804367	0.017407012
ENSG00000133740	E2F5	0.50	0.005831755	0.017486527
ENSG00000284024	HSPA14	-0.36	0.005838854	0.017505191
ENSG00000130856	ZNF236	0.51	0.005840359	0.017507079
ENSG00000170509	HSD17B13	1.57	0.005844732	0.017517565
ENSG00000115942	ORC2	0.27	0.005857294	0.017552585
ENSG00000128915	ICE2	-0.32	0.005870553	0.017589685
ENSG00000285446	Z84488.1	5.40	0.005888774	0.017641638
ENSG00000140092	FBLN5	0.68	0.005895112	0.017657983
ENSG00000131400	NAPSA	-1.68	0.005897047	0.017661135
ENSG00000245910	SNHG6	-0.19	0.005897955	0.017661211
ENSG00000108651	UTP6	-0.31	0.005899184	0.017662249
ENSG00000004534	RBM6	0.23	0.005911281	0.017695821
ENSG00000155158	TTC39B	-0.53	0.005919102	0.017716582
ENSG00000187049	TMEM216	-0.57	0.005920643	0.017718543
ENSG00000167325	RRM1	0.23	0.005928459	0.017739282
ENSG00000178184	PARD6G	-0.46	0.005929648	0.017740188
ENSG00000143867	OSR1	1.16	0.005932582	0.017746314
ENSG00000140396	NCOA2	-0.41	0.005942461	0.017773206
ENSG00000122484	RPAP2	-0.47	0.005968279	0.017847759
ENSG00000276390	AC004241.3	-3.27	0.005986054	0.017898238
ENSG00000228709	LINC02575	3.76	0.005991477	0.017911776
ENSG00000151665	PIGF	-0.40	0.00599511	0.01791996
ENSG00000188295	ZNF669	0.76	0.006000017	0.017931951
ENSG00000172661	WASHC2C	0.35	0.006014779	0.017973384
ENSG00000234851	RPL23AP42	0.25	0.006040016	0.018046103
ENSG00000131873	CHSY1	0.27	0.006042281	0.018049439
ENSG00000148296	SURF6	0.27	0.006042936	0.018049439
ENSG00000226329	AC005682.1	2.42	0.006047827	0.018061174
ENSG00000242612	DECR2	-0.56	0.00604867	0.018061174
ENSG00000247213	LINC01498	5.39	0.00607549	0.01813855
ENSG00000108759	KRT32	-1.91	0.006090606	0.018180968
ENSG00000204248	COL11A2	-0.65	0.006097162	0.018197822
ENSG00000138002	IFT172	-0.43	0.006101465	0.018207951
ENSG00000251562	MALAT1	-0.23	0.00610972	0.018229868
ENSG00000137941	TTLL7	0.37	0.006125933	0.018275516
ENSG00000103495	MAZ	-0.22	0.006146687	0.0183347
ENSG00000183476	SH2D7	1.89	0.006149055	0.018339029
ENSG00000169629	RGPD8	0.77	0.006165219	0.018384498
ENSG00000174749	FAM241A	-0.31	0.006168494	0.018391524
ENSG00000116044	NFE2L2	-0.24	0.006178428	0.018418396
ENSG00000214063	TSPAN4	0.28	0.006180145	0.018420539
ENSG00000159399	HK2	-0.48	0.006180987	0.018420539

ENSG00000232112	TMA7	0.18	0.006197731	0.018467687
ENSG00000111906	HDDC2	0.26	0.006206084	0.018489601
ENSG00000118420	UBE3D	0.66	0.006206933	0.018489601
ENSG00000184922	FMNL1	0.57	0.006217528	0.01851701
ENSG00000160194	NDUFV3	-0.31	0.006217985	0.01851701
ENSG00000171962	DRC3	-0.98	0.006220532	0.018519809
ENSG00000167702	KIFC2	-0.33	0.006220775	0.018519809
ENSG00000171055	FEZ2	0.38	0.006224783	0.018528984
ENSG00000196700	ZNF512B	0.36	0.006237983	0.018565515
ENSG00000169570	DTWD2	-0.71	0.006240141	0.018569174
ENSG00000262468	LINC01569	-0.89	0.006251975	0.018601624
ENSG00000214826	DDX12P	-0.46	0.006260638	0.01862463
ENSG00000165355	FBXO33	0.50	0.006270479	0.018651134
ENSG00000169249	ZRSR2	0.64	0.006286376	0.018695639
ENSG00000164970	FAM219A	0.33	0.006289487	0.018702113
ENSG00000151116	UEVLD	0.42	0.006310143	0.018760746
ENSG00000272604	AC073073.2	-2.45	0.006316616	0.018777201
ENSG00000068305	MEF2A	0.33	0.006332814	0.018822556
ENSG00000232388	SMIM26	-0.31	0.006353125	0.018879244
ENSG00000105879	CBLL1	-0.30	0.006353773	0.018879244
ENSG00000171206	TRIM8	-0.28	0.006355943	0.018882886
ENSG00000108406	DHX40	0.29	0.006361333	0.018896097
ENSG00000143278	F13B	5.38	0.006368728	0.018915255
ENSG00000277534	AC007996.1	-0.66	0.006384975	0.018960696
ENSG00000242852	ZNF709	-5.40	0.006387681	0.018965917
ENSG00000177602	HASPIN	-0.39	0.006396077	0.018988029
ENSG00000119547	ONECUT2	-0.43	0.00640475	0.019010955
ENSG00000116711	PLA2G4A	0.54	0.00640888	0.019020391
ENSG00000177788	AL162595.1	-0.78	0.006418542	0.019046242
ENSG00000215305	VPS16	-0.31	0.006433062	0.019085041
ENSG00000143036	SLC44A3	-0.60	0.006433524	0.019085041
ENSG00000157077	ZFYVE9	0.36	0.006463019	0.019169695
ENSG00000248333	CDK11B	0.24	0.006467237	0.019178324
ENSG00000091136	LAMB1	0.31	0.006467845	0.019178324
ENSG00000184007	PTP4A2	-0.17	0.006480012	0.019211557
ENSG00000119922	IFIT2	1.53	0.006512907	0.019306221
ENSG00000189114	BLOC1S3	0.42	0.006517263	0.019316273
ENSG00000158813	EDA	0.61	0.006519418	0.019319797
ENSG00000175772	LINC01106	-0.69	0.006539747	0.019377171
ENSG00000260328	AC104024.2	-0.91	0.006549849	0.01940423
ENSG00000090273	NUDC	0.37	0.006561821	0.019436092
ENSG00000163521	GLB1L	-0.69	0.006562546	0.019436092
ENSG00000243697	AC009108.1	-3.76	0.006599624	0.019543012
ENSG00000128340	RAC2	0.28	0.006614935	0.01958313
ENSG00000203724	C1orf53	-1.40	0.006615129	0.01958313
ENSG00000135049	AGTPBP1	0.40	0.006618792	0.019589794

ENSG00000108298	RPL19	-0.16	0.006619338	0.019589794
ENSG00000137338	PGBD1	0.61	0.006621147	0.019592251
ENSG00000128591	FLNC	-0.21	0.006624289	0.019594638
ENSG00000261150	EPPK1	0.37	0.006624687	0.019594638
ENSG00000006625	GGCT	-0.30	0.006624891	0.019594638
ENSG00000215251	FASTKD5	0.34	0.006639958	0.0196363
ENSG00000177189	RPS6KA3	-0.28	0.006647434	0.019655503
ENSG00000109332	UBE2D3	0.17	0.006649857	0.019659765
ENSG00000267480	AP001542.3	-5.39	0.006653109	0.019666473
ENSG00000125629	INSIG2	-0.44	0.006659882	0.019683585
ENSG00000118292	C1orf54	1.32	0.006668989	0.019707592
ENSG00000135976	ANKRD36	-0.89	0.006676896	0.019728044
ENSG00000069998	HDHD5	-0.52	0.006686489	0.019753473
ENSG00000251598	AC093916.1	3.02	0.006696206	0.019779258
ENSG00000092208	GEMIN2	0.36	0.006705292	0.019803173
ENSG00000188542	DUSP28	-0.78	0.006712659	0.019822005
ENSG00000160951	PTGER1	1.19	0.006716136	0.019829348
ENSG00000139597	N4BP2L1	1.76	0.006717353	0.019830013
ENSG00000110324	IL10RA	1.12	0.006721869	0.01984042
ENSG00000138642	HERC6	0.67	0.006724794	0.019846126
ENSG00000044459	CNTLN	-0.77	0.006730923	0.019861285
ENSG00000198520	ARMH1	-3.75	0.006735413	0.019871603
ENSG00000227449	FGF7P6	3.75	0.006756445	0.019930717
ENSG00000178033	CALHM5	2.54	0.006758729	0.019934516
ENSG00000141837	CACNA1A	0.78	0.006780613	0.019996113
ENSG00000114670	NEK11	1.37	0.006799356	0.020048433
ENSG00000159685	CHCHD6	-0.36	0.006800999	0.020050322
ENSG00000173905	GOLIM4	0.43	0.006812855	0.020082316
ENSG00000239264	TXNDC5	0.42	0.00681432	0.020083677
ENSG00000174405	LIG4	0.43	0.006829075	0.0201242
ENSG00000215187	FAM166B	-2.30	0.006830516	0.020125485
ENSG00000181894	ZNF329	0.91	0.006876901	0.020259171
ENSG00000119906	SLF2	0.23	0.006883261	0.020272356
ENSG00000063854	HAGH	0.29	0.006883406	0.020272356
ENSG00000177051	FBXO46	-0.37	0.006884416	0.020272356
ENSG00000165244	ZNF367	0.31	0.006893399	0.020295821
ENSG00000131019	ULBP3	0.39	0.006906634	0.020331797
ENSG00000151458	ANKRD50	0.33	0.006911965	0.020344497
ENSG00000095203	EPB41L4B	0.38	0.006922414	0.020369472
ENSG00000156172	C8orf37	-0.71	0.006922486	0.020369472
ENSG00000135446	CDK4	-0.20	0.006948969	0.020444393
ENSG00000063244	U2AF2	0.17	0.00696252	0.020481251
ENSG00000140307	GTF2A2	0.26	0.006965355	0.020486579
ENSG00000205740	AL359878.1	-2.72	0.00696718	0.020488936
ENSG00000167740	CYB5D2	0.47	0.006970512	0.020495721
ENSG00000134283	PPHLN1	0.28	0.00697493	0.020505698

ENSG00000092421	SEMA6A	0.53	0.007030693	0.020666601
ENSG00000148204	CRB2	2.20	0.007042656	0.020698727
ENSG00000283122	HYMAI	-5.39	0.007065423	0.020762591
ENSG00000259520	AC051619.4	-1.57	0.007079269	0.020800226
ENSG00000286388	AC026748.3	-0.68	0.00708867	0.02082479
ENSG00000177082	WDR73	-0.52	0.007129738	0.020942363
ENSG00000125462	C1orf61	-0.50	0.007138065	0.020963745
ENSG00000163157	TMOD4	-1.44	0.007160112	0.02102541
ENSG00000173465	ZNRD2	-0.35	0.007181403	0.021084834
ENSG00000175595	ERCC4	0.49	0.007196724	0.02112672
ENSG00000181220	ZNF746	0.32	0.007205459	0.021149258
ENSG00000205913	SRRM2-AS1	-1.20	0.007214388	0.021172361
ENSG00000153721	CNKSR3	-0.51	0.007219684	0.021184796
ENSG00000197381	ADARB1	-0.44	0.007235797	0.021228966
ENSG00000196295	GARS1-DT	0.48	0.007240448	0.021239498
ENSG00000033867	SLC4A7	0.79	0.007246609	0.021254455
ENSG00000109689	STIM2	0.27	0.00726878	0.021316357
ENSG00000225969	ABHD11-AS1	1.60	0.007313806	0.021444074
ENSG00000101017	CD40	0.36	0.007315179	0.021444074
ENSG00000229970	AC007128.1	-1.15	0.007315545	0.021444074
ENSG00000112584	FAM120B	-0.34	0.007320507	0.021455476
ENSG00000102081	FMR1	0.31	0.007324428	0.021463824
ENSG00000134697	GNL2	0.24	0.00733072	0.021479118
ENSG00000135245	HILPDA	-0.52	0.007338104	0.021497605
ENSG00000124788	ATXN1	-0.31	0.007339182	0.021497616
ENSG00000197816	CCDC180	-0.87	0.007341035	0.021499897
ENSG00000155313	USP25	0.37	0.00734648	0.021510538
ENSG00000196367	TRRAP	-0.23	0.007346818	0.021510538
ENSG00000103037	SETD6	-0.37	0.007353038	0.021525599
ENSG00000163590	PPM1L	-0.48	0.00736233	0.021549649
ENSG00000171766	GATM	-1.27	0.007379069	0.021595485
ENSG00000178764	ZHX2	0.36	0.007393534	0.021634653
ENSG00000135241	PNPLA8	0.45	0.007406128	0.021668337
ENSG00000133398	MED10	0.28	0.007412071	0.021682557
ENSG00000259030	FPGT-TNNI3K	2.20	0.007434278	0.02174434
ENSG00000204366	ZBTB12	-0.59	0.007436309	0.021744665
ENSG00000215769	ARHGAP27P1-	-1.01	0.007436562	0.021744665
ENSG00000189403	HMGB1	-0.17	0.007453922	0.021792239
ENSG00000164683	HEY1	0.75	0.007463003	0.021815601
ENSG00000101144	BMP7	0.47	0.007486324	0.021880578
ENSG00000099812	MISP	0.26	0.007494394	0.021900965
ENSG00000100532	CGRRF1	0.60	0.00750976	0.021942666
ENSG00000213160	KLHL23	-0.36	0.007523194	0.021978709
ENSG00000173085	COQ2	0.34	0.007535645	0.022011871
ENSG00000171700	RGS19	0.45	0.007545593	0.022037712
ENSG00000163041	H3-3A	0.78	0.007562967	0.022085231

ENSG00000183763	TRAIP	-0.35	0.007571502	0.022106931
ENSG00000125850	OVOL2	0.97	0.007593357	0.022167507
ENSG00000197056	ZMYM1	0.38	0.007606251	0.022201909
ENSG00000112706	IMPG1	-2.96	0.007637988	0.022291294
ENSG00000140836	ZFHX3	0.41	0.007650156	0.022323552
ENSG00000230798	FOXD3-AS1	1.25	0.007658236	0.022343873
ENSG00000225077	LINC00337	0.90	0.00766285	0.022354076
ENSG00000286259	AC078820.2	-0.97	0.007667499	0.02236374
ENSG00000103365	GGA2	0.21	0.007668398	0.02236374
ENSG00000110756	HPS5	0.35	0.007670146	0.022365579
ENSG00000198060	MARCHF5	0.23	0.007692175	0.022426546
ENSG00000142235	LMTK3	0.34	0.007693508	0.022427163
ENSG00000173611	SCAI	-0.39	0.007697325	0.022435024
ENSG00000279233	AC122688.3	0.97	0.007705588	0.022455837
ENSG00000170092	SPDYE5	-2.56	0.007723294	0.022504158
ENSG00000114270	COL7A1	-0.52	0.007731863	0.022525848
ENSG00000162729	IGSF8	-0.35	0.007739559	0.022544987
ENSG00000164484	TMEM200A	0.46	0.007746972	0.022563296
ENSG00000196199	MPHOSPH8	-0.31	0.007786263	0.022674431
ENSG00000160539	PLPP7	3.23	0.007791094	0.022685201
ENSG00000068654	POLR1A	-0.24	0.00780059	0.022709543
ENSG00000155629	PIK3AP1	0.51	0.007819735	0.022761968
ENSG00000148143	ZNF462	0.40	0.007839602	0.022816481
ENSG00000019995	ZRANB1	0.36	0.007843932	0.022825763
ENSG00000147647	DPYS	5.30	0.007845343	0.022826549
ENSG00000183258	DDX41	0.20	0.007850859	0.022831042
ENSG00000185933	CALHM1	5.30	0.007851327	0.022831042
ENSG00000215612	HMX1	5.30	0.007851327	0.022831042
ENSG00000115827	DCAF17	-0.46	0.007851451	0.022831042
ENSG00000272316	AL021368.2	-1.35	0.007858118	0.02284711
ENSG00000258469	CHMP4BP1	2.92	0.007859695	0.022848376
ENSG00000197149	AC107956.1	4.60	0.007862085	0.022851482
ENSG00000203867	RBM20	0.50	0.007863047	0.022851482
ENSG00000150961	SEC24D	0.29	0.007870702	0.022870407
ENSG00000169330	MINAR1	0.86	0.007894564	0.022936415
ENSG00000231925	TAPBP	0.20	0.007929409	0.023034305
ENSG00000228998	AC091167.1	-2.68	0.007930897	0.023035284
ENSG00000110628	SLC22A18	-0.43	0.007935595	0.023045585
ENSG00000072786	STK10	-0.38	0.007942689	0.023062839
ENSG00000171135	JAGN1	-0.29	0.007948754	0.02307533
ENSG00000179141	MTUS2-AS1	3.17	0.007949297	0.02307533
ENSG00000134109	EDEM1	-0.32	0.00795563	0.023090364
ENSG00000141026	MED9	-0.44	0.007959309	0.023097694
ENSG00000233426	EIF3FP3	0.43	0.007965265	0.023111627
ENSG00000288612	AL133351.4	-1.79	0.008007391	0.023230488
ENSG00000260630	SNAI3-AS1	-1.11	0.008035695	0.023309222

ENSG00000231793	DOC2GP	-2.68	0.00804541	0.023334021
ENSG00000154553	PDLIM3	0.46	0.008054959	0.02335833
ENSG00000137720	C11orf1	-0.49	0.008071695	0.023403472
ENSG00000127993	RBM48	0.45	0.008084487	0.023437166
ENSG00000178127	NDUFV2	0.31	0.00808831	0.023444852
ENSG00000135269	TES	-0.20	0.008105156	0.023490281
ENSG00000265194	AL359922.2	-1.34	0.008121115	0.023533125
ENSG00000146067	FAM193B	-0.34	0.008124781	0.023538603
ENSG00000155542	SETD9	-0.72	0.008125358	0.023538603
ENSG00000278970	HEIH	-0.62	0.008134288	0.023561062
ENSG00000133247	KMT5C	-0.40	0.008149349	0.02360127
ENSG00000128585	MKLN1	-0.38	0.008157328	0.023620688
ENSG00000182446	NPLOC4	0.17	0.008159278	0.023620688
ENSG00000279347	AC021945.1	-1.79	0.008159595	0.023620688
ENSG00000066455	GOLGA5	0.41	0.008167084	0.023638949
ENSG00000039319	ZFYVE16	0.39	0.008170545	0.023645546
ENSG00000196510	ANAPC7	0.24	0.008187836	0.023692159
ENSG00000165125	TRPV6	-1.94	0.008214383	0.02376554
ENSG00000172757	CFL1	0.21	0.008228733	0.023803613
ENSG00000168137	SETD5	-0.19	0.008233823	0.023814897
ENSG00000185608	MRPL40	-0.29	0.008239443	0.023827707
ENSG00000243224	AC006252.1	-1.20	0.008263308	0.023893269
ENSG00000280417	AC096887.2	-4.65	0.008278892	0.023933831
ENSG00000079785	DDX1	0.20	0.008279728	0.023933831
ENSG00000181016	LSMEM1	1.49	0.008281937	0.023936759
ENSG00000049247	UTS2	1.82	0.008287807	0.023950266
ENSG00000064199	SPA17	-0.63	0.008312789	0.024018992
ENSG00000268655	AC008687.4	1.11	0.00832963	0.024064177
ENSG00000158006	PAFAH2	0.33	0.00833519	0.024074159
ENSG00000004864	SLC25A13	0.38	0.008335491	0.024074159
ENSG00000087152	ATXN7L3	0.20	0.008358406	0.024136858
ENSG00000088876	ZNF343	-0.59	0.00836247	0.024145108
ENSG00000121057	AKAP1	-0.21	0.008366928	0.024154495
ENSG00000236824	BCYRN1	1.31	0.008373896	0.024171123
ENSG00000182957	SPATA13	-0.43	0.008387802	0.024207774
ENSG00000169905	TOR1AIP2	0.20	0.008389515	0.024209224
ENSG00000160298	C21orf58	-0.51	0.00839464	0.024220521
ENSG00000100714	MTHFD1	-0.18	0.008399229	0.024230268
ENSG00000101871	MID1	0.27	0.008407656	0.024251082
ENSG00000221909	FAM200A	0.62	0.00843082	0.024314391
ENSG00000136897	MRPL50	0.35	0.008433721	0.024319254
ENSG00000087299	L2HGDH	-0.39	0.008439248	0.024331687
ENSG00000242372	EIF6	-0.20	0.008450931	0.02436186
ENSG00000224903	RNF32-AS1	-1.01	0.008460761	0.024383592
ENSG00000228775	WEE2-AS1	1.64	0.008460907	0.024383592
ENSG00000169446	MMGT1	-0.25	0.008465588	0.02439357

ENSG00000086758	HUWE1	-0.18	0.008471029	0.024402993
ENSG00000178927	CYBC1	-0.25	0.008472451	0.024402993
ENSG00000143320	CRABP2	0.38	0.008472516	0.024402993
ENSG00000100625	SIX4	0.37	0.0084801	0.024421322
ENSG00000167193	CRK	0.26	0.008490601	0.024448043
ENSG00000173517	PEAK1	-0.33	0.008499972	0.024471506
ENSG00000176915	ANKLE2	0.22	0.00850407	0.024479782
ENSG00000105618	PRPF31	-0.25	0.008510084	0.024493571
ENSG00000047644	WWC3	0.90	0.008512807	0.024497886
ENSG00000103175	WFDC1	2.68	0.008524962	0.024526965
ENSG00000149136	SSRP1	-0.16	0.008525363	0.024526965
ENSG00000162006	MSLNL	-1.29	0.008550639	0.024596147
ENSG00000172469	MANEA	0.44	0.008563937	0.024630858
ENSG00000132361	CLUH	-0.19	0.008583154	0.024681078
ENSG00000214530	STARD10	0.32	0.008583865	0.024681078
ENSG00000008282	SYPL1	0.19	0.008600389	0.024725037
ENSG00000175701	MTLN	0.40	0.008612921	0.024757508
ENSG00000179886	TIGD5	0.41	0.008614519	0.024758545
ENSG00000157349	DDX19B	0.35	0.008618429	0.024766227
ENSG00000126603	GLIS2	-0.44	0.008635095	0.0248067
ENSG00000245573	BDNF-AS	-1.27	0.008636433	0.0248067
ENSG00000063587	ZNF275	-0.36	0.00863685	0.0248067
ENSG00000092096	SLC22A17	0.54	0.008637472	0.0248067
ENSG00000134962	KLB	-5.30	0.008639549	0.024809106
ENSG00000164867	NOS3	-0.35	0.008652097	0.024841574
ENSG00000186326	RGS9BP	2.06	0.008663507	0.024870766
ENSG00000136237	RAPGEF5	-0.47	0.008681819	0.024919758
ENSG00000078081	LAMP3	-0.51	0.008688895	0.024936494
ENSG00000242861	AL591895.1	-1.57	0.008691021	0.02493902
ENSG00000135387	CAPRIN1	0.17	0.008697089	0.024952852
ENSG00000164008	C1orf50	-0.98	0.008699858	0.024957218
ENSG00000133315	MACROD1	-0.31	0.008709483	0.02498125
ENSG00000118482	PHF3	0.28	0.008716933	0.024999035
ENSG00000147586	MRPS28	0.29	0.00871892	0.025001151
ENSG00000075336	TIMM21	0.32	0.008727375	0.025021808
ENSG00000105926	MPP6	0.35	0.008729606	0.02502462
ENSG00000114686	MRPL3	0.21	0.00876406	0.025119789
ENSG00000249863	AC021106.1	-2.51	0.008770977	0.025136015
ENSG00000135686	KLHL36	-0.22	0.008782072	0.025164129
ENSG00000146530	VWDE	-0.48	0.008783302	0.025164129
ENSG00000116857	TMEM9	0.25	0.008804903	0.025222405
ENSG00000135211	TMEM60	0.39	0.008812618	0.025240892
ENSG00000197217	ENTPD4	0.23	0.008816636	0.025248787
ENSG00000143158	MPC2	0.27	0.008819753	0.025254098
ENSG00000143919	CAMKMT	0.57	0.008825335	0.025266466
ENSG00000152154	TMEM178A	-3.17	0.008831765	0.025281258

ENSG00000115963	RND3	-0.46	0.008836539	0.025291308
ENSG00000204852	TCTN1	-0.50	0.008874144	0.025395305
ENSG00000182712	CMC4	0.99	0.008876328	0.025397922
ENSG00000267476	AC104365.2	2.86	0.008894154	0.025445291
ENSG00000173418	NAA20	0.24	0.008900614	0.025460134
ENSG00000223343	AC137630.1	-5.29	0.008933592	0.025544265
ENSG00000261757	AC005592.1	-5.29	0.008933592	0.025544265
ENSG00000185168	LINC00482	0.68	0.008933855	0.025544265
ENSG00000149761	NUDT22	-0.38	0.008975724	0.025660313
ENSG00000138231	DBR1	0.32	0.008997928	0.0257173
ENSG00000174327	SLC16A13	-0.69	0.008998228	0.0257173
ENSG00000117222	RBBP5	0.27	0.009006057	0.025734678
ENSG00000197355	UAP1L1	0.34	0.00900688	0.025734678
ENSG00000115844	DLX2	-1.44	0.009009311	0.025737949
ENSG00000250988	SNHG21	0.82	0.009019473	0.025760792
ENSG00000133606	MKRN1	-0.20	0.009019882	0.025760792
ENSG00000162702	ZNF281	0.31	0.009036859	0.025805596
ENSG00000286834	AL360020.1	-3.64	0.009044877	0.025824807
ENSG00000211448	DIO2	2.86	0.009064114	0.025876042
ENSG00000049860	HEXB	0.20	0.009067271	0.025881363
ENSG00000184857	TMEM186	-0.43	0.009088325	0.025937758
ENSG00000255008	LINC02739	2.63	0.009090549	0.025939138
ENSG00000054267	ARID4B	0.35	0.009092373	0.025939138
ENSG00000143816	WNT9A	0.57	0.009092697	0.025939138
ENSG00000179818	PCBP1-AS1	0.35	0.009102662	0.025963863
ENSG00000023839	ABCC2	-0.23	0.009113594	0.025991343
ENSG00000102974	CTCF	0.22	0.009119394	0.026004177
ENSG00000134897	BIVM	-0.55	0.009145314	0.026074372
ENSG00000230457	PA2G4P4	0.86	0.009210769	0.026257252
ENSG00000231638	LUARIS	2.35	0.00922064	0.026281511
ENSG00000227885	AL590652.1	-1.59	0.009221905	0.026281511
ENSG00000166025	AMOTL1	-0.25	0.009231754	0.026305832
ENSG00000083535	PIBF1	0.49	0.009247244	0.02634622
ENSG00000181467	RAP2B	-0.22	0.009254749	0.026363849
ENSG00000120063	GNA13	0.24	0.00925912	0.026372545
ENSG00000213073	AL353625.1	1.35	0.009273146	0.026408092
ENSG00000121542	SEC22A	0.42	0.009274239	0.026408092
ENSG00000103061	SLC7A6OS	0.51	0.00927728	0.026412992
ENSG00000064270	ATP2C2	-0.52	0.009280038	0.026417087
ENSG00000198951	NAGA	-0.28	0.009304416	0.026482717
ENSG00000111641	NOP2	-0.42	0.009319204	0.026521035
ENSG00000083520	DIS3	-0.26	0.009346693	0.026595481
ENSG00000124920	MYRF	-0.22	0.009350277	0.026601898
ENSG00000096401	CDC5L	0.27	0.009356011	0.026614428
ENSG00000115267	IFIH1	0.84	0.009359756	0.026621296
ENSG00000128917	DLL4	0.77	0.009371568	0.026651104

ENSG00000261211	AL031123.2	-1.91	0.009401805	0.026733292
ENSG00000135931	ARMC9	0.42	0.009411627	0.02675742
ENSG00000264538	SUZ12P1	0.51	0.009413448	0.026758795
ENSG00000174173	TRMT10C	0.33	0.009426129	0.026791036
ENSG00000146414	SHPRH	-0.78	0.009432507	0.026804068
ENSG00000214413	BBIP1	0.39	0.009433392	0.026804068
ENSG00000177352	CCDC71	0.32	0.009438665	0.026815243
ENSG00000159403	C1R	0.90	0.009445458	0.026830731
ENSG00000140832	MARVELD3	-0.43	0.009459651	0.026867235
ENSG00000166949	SMAD3	-0.20	0.009462184	0.026870616
ENSG00000048405	ZNF800	0.39	0.009464179	0.026872468
ENSG00000133466	C1QTNF6	0.38	0.009477649	0.026906897
ENSG00000107954	NEURL1	1.01	0.009480675	0.026911669
ENSG00000178966	RMI1	-0.32	0.009493984	0.026945627
ENSG00000279488	AC004623.1	1.85	0.009513397	0.026996896
ENSG00000103150	MLYCD	-0.55	0.009537548	0.027061594
ENSG00000205309	NT5M	-0.68	0.009543774	0.027075418
ENSG00000156011	PSD3	-0.39	0.009549435	0.027084951
ENSG00000165996	HACD1	0.37	0.009549841	0.027084951
ENSG00000246922	UBAP1L	-0.59	0.009568883	0.027135111
ENSG00000150281	CTF1	-1.06	0.009600396	0.027220619
ENSG00000286116	AL157394.3	4.53	0.009605445	0.027231075
ENSG00000174373	RALGAPA1	0.45	0.009611576	0.027244597
ENSG00000258875	AL135818.1	-2.37	0.009625503	0.02728021
ENSG00000085788	DDHD2	-0.27	0.009631874	0.027291325
ENSG00000183579	ZNRF3	-0.35	0.009632152	0.027291325
ENSG00000176623	RMDN1	-0.26	0.009634179	0.027293203
ENSG00000134046	MBD2	0.24	0.009642275	0.027312273
ENSG00000182568	SATB1	0.58	0.009654711	0.027343628
ENSG00000255769	GOLGA2P10	-0.43	0.009661382	0.02735865
ENSG00000168769	TET2	0.48	0.00967004	0.027379293
ENSG00000106460	TMEM106B	-0.34	0.009693437	0.027437878
ENSG00000280571	AC006059.2	0.83	0.009693474	0.027437878
ENSG00000153029	MR1	-0.60	0.009730532	0.027538878
ENSG00000138772	ANXA3	-0.18	0.009756051	0.027607197
ENSG00000129946	SHC2	-1.92	0.009772211	0.027649017
ENSG00000139926	FRMD6	0.51	0.00977853	0.027662983
ENSG00000184517	ZFP1	0.55	0.009781907	0.027666664
ENSG00000223509	AC135983.3	-1.04	0.009782596	0.027666664
ENSG00000163697	APBB2	-0.24	0.009804332	0.027724219
ENSG00000181790	ADGRB1	-1.07	0.009807467	0.027729164
ENSG00000127527	EPS15L1	-0.29	0.009810272	0.027733177
ENSG00000101624	CEP76	0.43	0.009820713	0.027758771
ENSG00000205758	CRYZL1	0.39	0.009840919	0.027811956
ENSG00000167748	KLK1	0.77	0.009850476	0.027835035
ENSG00000169252	ADRB2	1.82	0.009863514	0.027867943

ENSG00000145349	CAMK2D	-0.24	0.009874661	0.0278955
ENSG00000162078	ZG16B	0.94	0.009925743	0.028035845
ENSG00000063127	SLC6A16	-0.86	0.009936644	0.028062674
ENSG00000237510	GPAT2P1	0.50	0.009942908	0.028076403
ENSG00000249035	CLMAT3	-1.99	0.00994488	0.028078011
ENSG00000248866	USP46-DT	-1.73	0.009959516	0.028112864
ENSG00000144736	SHQ1	0.34	0.009960034	0.028112864
ENSG00000188917	TRMT2B	0.40	0.01001432	0.028262103
ENSG00000236409	NRADDP	-2.22	0.010035272	0.02831724
ENSG00000103512	NOMO1	0.17	0.010059214	0.028380797
ENSG00000102003	SYP	-1.20	0.01007427	0.028419267
ENSG00000245571	FAM111A-DT	1.06	0.01008159	0.02843591
ENSG00000135835	KIAA1614	-1.12	0.010087394	0.028448272
ENSG00000258057	BCDIN3D-AS1	-1.27	0.010099654	0.028478833
ENSG00000243479	MNX1-AS1	-0.94	0.010101081	0.028478845
ENSG00000163466	ARPC2	-0.18	0.010121722	0.02853302
ENSG00000213398	LCAT	-0.81	0.010128114	0.028547016
ENSG00000048140	TSPAN17	0.20	0.010135046	0.028562532
ENSG00000166343	MSS51	0.90	0.010151375	0.028604522
ENSG00000177485	ZBTB33	-0.40	0.010163328	0.028631891
ENSG00000286129	AC245060.7	0.50	0.010163949	0.028631891
ENSG00000137207	YIPF3	-0.23	0.010175686	0.028658946
ENSG00000137440	FGFBP1	0.82	0.010176418	0.028658946
ENSG00000196747	H2AC13	3.12	0.010187357	0.028685718
ENSG00000178053	MLF1	0.32	0.010195314	0.028704083
ENSG00000167716	WDR81	-0.36	0.010197736	0.028704266
ENSG00000180881	CAPS2	0.48	0.010198248	0.028704266
ENSG00000049656	CLPTM1L	0.18	0.01021232	0.028739831
ENSG00000101161	PRPF6	0.18	0.01023815	0.028808473
ENSG00000130338	TULP4	0.34	0.01024656	0.028828083
ENSG00000115840	SLC25A12	0.32	0.010249585	0.028832542
ENSG00000133612	AGAP3	-0.24	0.010254739	0.028842985
ENSG00000176386	CDC26	0.41	0.010271434	0.028885881
ENSG00000102786	INTS6	0.35	0.010285335	0.028920911
ENSG00000067113	PLPP1	0.45	0.01031241	0.028992967
ENSG00000197912	SPG7	0.22	0.010314591	0.028995028
ENSG00000261437	AC108860.2	-1.21	0.010316313	0.028995795
ENSG00000119917	IFIT3	1.31	0.010318432	0.028997677
ENSG00000186416	NKRF	0.34	0.010328365	0.029018921
ENSG00000204839	MROH6	-0.36	0.010328891	0.029018921
ENSG00000144591	GMPPA	-0.25	0.010337049	0.029037763
ENSG00000278811	LINC00624	-1.72	0.010347017	0.029061686
ENSG00000224023	EDRF1-DT	-4.51	0.010356643	0.029084638
ENSG00000101310	SEC23B	0.20	0.010360599	0.029091666
ENSG00000198825	INPP5F	0.28	0.010379602	0.029140937
ENSG00000145494	NDUFS6	0.51	0.010413281	0.029231391

ENSG00000235651	G3BP1P1	-4.51	0.010444029	0.029313591
ENSG00000231359	AC072052.1	-3.65	0.010446938	0.029317646
ENSG00000142233	NTN5	-1.11	0.010465367	0.029365245
ENSG00000174977	AC026271.1	0.63	0.010480195	0.029402728
ENSG00000084754	HADHA	0.16	0.010489839	0.02942566
ENSG00000221821	C6orf226	-0.73	0.010506864	0.029469287
ENSG00000116514	RNF19B	0.35	0.010536661	0.029548718
ENSG00000204396	VWA7	0.47	0.010568925	0.029635045
ENSG00000118961	LDAH	0.39	0.010571194	0.029637256
ENSG00000135899	SP110	0.69	0.010589493	0.029681462
ENSG00000101977	MCF2	5.20	0.010592414	0.029681462
ENSG00000236512	AL390026.1	5.20	0.010592414	0.029681462
ENSG00000214967	NPIPA7	-2.26	0.010592894	0.029681462
ENSG00000271013	LRRC37A9P	-2.63	0.010605117	0.029711551
ENSG00000128394	APOBEC3F	-0.44	0.010607232	0.029713316
ENSG00000187642	PERM1	2.91	0.010621194	0.029748262
ENSG00000151327	FAM177A1	0.27	0.010626956	0.029760236
ENSG00000164038	SLC9B2	-0.38	0.010629499	0.029763192
ENSG00000174353	STAG3L3	0.63	0.010640645	0.029790235
ENSG00000102359	SRPX2	0.71	0.010655543	0.02982777
ENSG00000058799	YIPF1	0.36	0.010666727	0.029851663
ENSG00000276550	HERC2P2	-0.27	0.010667062	0.029851663
ENSG00000164961	WASHC5	-0.28	0.010669755	0.029855024
ENSG00000102189	EEA1	0.42	0.010680093	0.029879774
ENSG00000234899	SOX9-AS1	-1.16	0.01068512	0.02988966
ENSG00000085831	TTC39A	-0.35	0.01072198	0.029988576
ENSG00000178607	ERN1	-0.31	0.010724477	0.029991368
ENSG00000082512	TRAF5	0.48	0.010728996	0.029999813
ENSG00000204650	LINC02210	-0.27	0.010737685	0.030019916
ENSG00000133794	ARNTL	-0.39	0.0107499	0.030049868
ENSG00000270052	BX546450.2	5.21	0.01075714	0.030065906
ENSG00000275778	AC018630.2	1.49	0.010767137	0.030089643
ENSG00000151806	GUF1	-0.29	0.010805936	0.030191353
ENSG00000228623	ZNF883	5.28	0.010806549	0.030191353
ENSG00000042832	TG	4.47	0.010815955	0.030213413
ENSG00000165264	NDUFB6	0.24	0.010824357	0.03023163
ENSG00000104976	SNAPC2	0.30	0.010825498	0.03023163
ENSG00000008324	SS18L2	-0.35	0.010830526	0.030241452
ENSG00000101363	MANBAL	-0.27	0.010833013	0.030244177
ENSG00000184220	CMSS1	-0.30	0.010841429	0.030263451
ENSG00000243547	HNRNPKP4	0.93	0.010843917	0.030266175
ENSG00000172428	COPS9	0.25	0.010848268	0.030274095
ENSG00000065183	WDR3	-0.25	0.010866486	0.030320708
ENSG00000120699	EXOSC8	-0.23	0.010877915	0.030348368
ENSG00000121892	PDS5A	0.18	0.010883055	0.030358475
ENSG00000159915	ZNF233	1.65	0.010898822	0.030398221

ENSG00000205189	ZBTB10	-0.30	0.010906668	0.030415864
ENSG00000268364	SMC5-AS1	2.85	0.010922977	0.0304571
ENSG00000135999	EPC2	0.42	0.010941623	0.030504842
ENSG00000273154	AL121845.3	-1.30	0.010956499	0.030541526
ENSG00000283399	AC004381.3	4.47	0.010957833	0.030541526
ENSG00000204947	ZNF425	-1.45	0.01096757	0.030564407
ENSG00000140280	LYSMD2	0.40	0.010978714	0.030591204
ENSG00000267244	AC012615.6	0.95	0.01098131	0.030594179
ENSG00000196562	SULF2	-0.45	0.010984402	0.030598531
ENSG00000115760	BIRC6	0.24	0.010999644	0.030636725
ENSG00000280407	AC132872.4	-1.35	0.011030579	0.030718613
ENSG00000104866	PPP1R37	0.26	0.01103673	0.030731466
ENSG00000188522	FAM83G	0.21	0.011044793	0.030749639
ENSG00000142528	ZNF473	0.41	0.011076467	0.030829264
ENSG00000236914	LINC01852	-1.48	0.011076474	0.030829264
ENSG00000163870	TPRA1	-0.31	0.011081066	0.030837754
ENSG00000130150	MOSPD2	0.48	0.011084894	0.030844117
ENSG00000275764	AC092747.4	-1.81	0.011095749	0.030870031
ENSG00000136352	NKX2-1	1.32	0.011101048	0.030880481
ENSG00000160563	MED27	0.32	0.011129682	0.030950281
ENSG00000078237	TIGAR	0.44	0.011133237	0.030950281
ENSG00000100078	PLA2G3	5.21	0.011133256	0.030950281
ENSG00000258588	TRIM6-TRIM34	5.21	0.011133256	0.030950281
ENSG00000122592	HOXA7	0.43	0.011134173	0.030950281
ENSG00000287853	AL031668.2	5.19	0.01113542	0.030950281
ENSG00000226416	MRPL23-AS1	-2.36	0.011144719	0.030971824
ENSG00000199691	RN7SKP173	-1.45	0.011151749	0.03098706
ENSG00000140459	CYP11A1	4.47	0.011156132	0.030994934
ENSG00000131746	TNS4	-0.31	0.011175203	0.03104361
ENSG00000143379	SETDB1	0.25	0.011181911	0.031057933
ENSG00000262580	AC087741.1	-0.60	0.011191831	0.031081171
ENSG00000185864	NPIPB4	-1.88	0.011198037	0.031094092
ENSG00000130758	MAP3K10	0.40	0.011206018	0.031111936
ENSG00000162627	SNX7	0.30	0.011214193	0.031130314
ENSG00000183475	ASB7	0.39	0.011221578	0.031146494
ENSG00000259305	ZHX1-C8orf76	1.21	0.011239151	0.031190944
ENSG00000138613	APH1B	0.46	0.011244872	0.031202492
ENSG00000124587	PEX6	-0.33	0.011284584	0.031308346
ENSG00000134489	HRH4	2.87	0.011312781	0.031382224
ENSG00000273899	NOL12	0.44	0.011330302	0.031426472
ENSG00000088881	EBF4	-0.70	0.011336809	0.031440164
ENSG00000232237	ASCL5	1.50	0.011352649	0.031479729
ENSG00000143418	CERS2	-0.16	0.011358282	0.031489839
ENSG00000079150	FKBP7	0.96	0.011359442	0.031489839
ENSG00000170633	RNF34	0.24	0.011376736	0.031533412
ENSG00000285053	TBCE	0.66	0.01140061	0.031595211

ENSG00000275496	CU633906.1	4.47	0.01140892	0.031613862
ENSG00000288596	C8orf44	0.77	0.011421032	0.031643043
ENSG00000134909	ARHGAP32	0.30	0.011431029	0.031666355
ENSG00000232611	AL683813.2	-1.34	0.011445493	0.031702036
ENSG00000168385	SEPTIN2	0.15	0.011454259	0.031721926
ENSG00000125457	MIF4GD	0.34	0.011462095	0.031739234
ENSG00000171681	ATF7IP	0.24	0.011463746	0.031739414
ENSG00000054611	TBC1D22A	-0.32	0.011466754	0.031743351
ENSG00000169957	ZNF768	0.21	0.011480416	0.031776775
ENSG00000186866	POFUT2	-0.32	0.011485417	0.031785809
ENSG00000283103	AC010642.2	-0.48	0.011486856	0.031785809
ENSG00000122406	RPL5	-0.14	0.011508503	0.031841305
ENSG00000151773	CCDC122	-1.21	0.011539031	0.031921355
ENSG00000254635	WAC-AS1	-0.61	0.011543994	0.031930672
ENSG00000206344	HCG27	0.95	0.011562211	0.031976641
ENSG00000079432	CIC	-0.25	0.011565435	0.031981138
ENSG00000269378	AC022149.1	0.36	0.011573104	0.031997921
ENSG00000180747	SMG1P3	-0.99	0.01158744	0.032033134
ENSG00000085465	OVGP1	0.63	0.01159018	0.032036281
ENSG00000165487	MICU2	0.25	0.011618011	0.032108775
ENSG00000177106	EPS8L2	-0.21	0.01162018	0.032110335
ENSG00000227560	RPS15AP30	3.13	0.011652491	0.032195174
ENSG00000102981	PARD6A	0.62	0.011679001	0.032263965
ENSG00000160959	LRRC14	0.26	0.011694863	0.032303325
ENSG00000236751	LINC01186	0.76	0.011697734	0.032306796
ENSG00000052723	SIKE1	-0.28	0.011704984	0.03232236
ENSG00000005073	HOXA11	0.43	0.011707106	0.03232376
ENSG00000020426	MNAT1	0.37	0.011722728	0.032362427
ENSG00000126243	LRFN3	0.36	0.011727309	0.032370609
ENSG00000260276	AC022167.2	-1.48	0.0117394	0.032399514
ENSG00000164548	TRA2A	-0.20	0.011751134	0.032427425
ENSG00000090097	PCBP4	-0.24	0.011775164	0.032489256
ENSG00000115042	FAHD2A	-0.36	0.011796935	0.032540593
ENSG00000102032	RENBP	1.28	0.011797022	0.032540593
ENSG00000163539	CLASP2	-0.38	0.01180882	0.032568649
ENSG00000004455	AK2	0.15	0.011852584	0.032684844
ENSG00000136114	THSD1	2.34	0.011859906	0.032700529
ENSG00000033178	UBA6	0.24	0.011871071	0.032726803
ENSG00000257921	AC025165.3	-5.20	0.011875342	0.032734069
ENSG00000234537	AL354751.1	2.41	0.011878651	0.032738681
ENSG00000134884	ARGLU1	-0.21	0.011894883	0.032778903
ENSG00000148429	USP6NL	0.27	0.011897306	0.032781068
ENSG00000205795	CYS1	-0.83	0.011905215	0.032798342
ENSG00000179954	SSC5D	-3.57	0.011947784	0.032911088
ENSG00000105778	AVL9	-0.24	0.011962325	0.032946607
ENSG00000273045	C2orf15	1.40	0.011993814	0.033028788

ENSG00000258101	AC010173.1	-1.52	0.012041912	0.033155397
ENSG00000006757	PNPLA4	-0.47	0.012043103	0.033155397
ENSG00000131724	IL13RA1	-0.27	0.012074869	0.033238279
ENSG00000109536	FRG1	0.30	0.01211379	0.03334083
ENSG00000225953	SATB2-AS1	0.68	0.012151139	0.033439026
ENSG00000145335	SNCA	0.87	0.012173519	0.033496006
ENSG00000142173	COL6A2	-0.20	0.012203352	0.03357348
ENSG00000178105	DDX10	0.31	0.012211024	0.033589968
ENSG00000112062	MAPK14	0.22	0.012240376	0.03366608
ENSG00000164078	MST1R	-0.25	0.012267601	0.033736324
ENSG00000065923	SLC9A7	0.28	0.012281673	0.033767128
ENSG00000143222	UFC1	-0.24	0.012282177	0.033767128
ENSG00000176024	ZNF613	1.00	0.012304378	0.033822916
ENSG00000185420	SMYD3	0.41	0.012305849	0.033822916
ENSG00000181649	PHLDA2	-0.19	0.012327103	0.033874905
ENSG00000174938	SEZ6L2	0.26	0.01232815	0.033874905
ENSG00000105202	FBL	0.17	0.012412683	0.0341025
ENSG00000125531	FNDC11	0.90	0.012414615	0.034103126
ENSG00000176182	MYPOP	0.47	0.012416359	0.034103236
ENSG00000270574	AC010680.2	-2.65	0.012445416	0.034178354
ENSG00000114853	ZBTB47	0.44	0.01247463	0.034250674
ENSG00000148396	SEC16A	-0.21	0.012475173	0.034250674
ENSG00000089220	PEBP1	-0.17	0.012528385	0.03439205
ENSG00000250462	LRRC37BP1	-0.59	0.012534639	0.034404497
ENSG00000139289	PHLDA1	-0.15	0.012538669	0.034405862
ENSG00000187699	C2orf88	-1.07	0.012540411	0.034405862
ENSG00000197283	SYNGAP1	0.42	0.012540523	0.034405862
ENSG00000234498	RPL13AP20	1.08	0.012542013	0.034405862
ENSG00000177738	AC025171.1	0.91	0.012549563	0.034421855
ENSG00000124222	STX16	-0.21	0.012571687	0.034477813
ENSG00000267879	AC011483.1	-1.81	0.012577775	0.034489783
ENSG00000110721	CHKA	0.28	0.012605203	0.034560259
ENSG00000179431	FJX1	0.42	0.012611021	0.034571474
ENSG00000140471	LINS1	0.47	0.012634976	0.0346324
ENSG00000168454	TXNDC2	2.76	0.012640795	0.034643603
ENSG00000267751	AC009005.1	0.71	0.012691799	0.034778624
ENSG00000165695	AK8	-1.36	0.012694182	0.03478039
ENSG00000251023	AC114980.1	-5.19	0.012705874	0.034807659
ENSG00000251580	LINC02482	-2.18	0.012712245	0.03481583
ENSG00000198556	ZNF789	-0.52	0.012712336	0.03481583
ENSG00000274049	INO80B-WBP1	4.41	0.012729712	0.034858648
ENSG00000186660	ZFP91	-0.19	0.012775658	0.03497968
ENSG00000234147	AL035446.1	0.94	0.012780079	0.034986997
ENSG00000110169	HPX	-1.32	0.012786345	0.034999362
ENSG00000145740	SLC30A5	0.24	0.012842611	0.035148567
ENSG00000172262	ZNF131	0.31	0.012882975	0.035254219

ENSG00000100379	KCTD17	-0.39	0.012888181	0.035263643
ENSG00000184990	SIVA1	-0.23	0.012947276	0.035420491
ENSG00000164163	ABCE1	0.17	0.012956478	0.035440821
ENSG00000180867	PDIA3P1	0.40	0.012976766	0.035491464
ENSG00000103254	ANTKMT	0.39	0.012978859	0.035492337
ENSG00000188641	DPYD	1.18	0.01300603	0.035561781
ENSG00000156384	SFR1	-0.34	0.013021314	0.035598707
ENSG00000175093	SPSB4	1.34	0.013041931	0.035650202
ENSG00000140153	WDR20	0.37	0.013053347	0.035675214
ENSG00000255346	NOX5	1.30	0.013054647	0.035675214
ENSG00000099992	TBC1D10A	0.42	0.013058275	0.035680257
ENSG00000164209	SLC25A46	0.33	0.01311904	0.035841397
ENSG00000164054	SHISA5	0.20	0.013126638	0.035857259
ENSG00000196139	AKR1C3	1.21	0.013139766	0.035888222
ENSG00000130175	PRKCSH	-0.18	0.013142775	0.035891541
ENSG00000057663	ATG5	0.28	0.013151211	0.035909679
ENSG00000151208	DLG5	-0.50	0.013162143	0.035934625
ENSG00000148660	CAMK2G	0.25	0.013167611	0.035944649
ENSG00000260423	LINC02367	-2.01	0.013184329	0.035985377
ENSG00000152904	GGPS1	0.42	0.013203039	0.03603153
ENSG00000196873	CBWD3	0.61	0.013205091	0.036032216
ENSG00000075624	ACTB	0.20	0.013231859	0.036100335
ENSG00000143155	TIPRL	0.24	0.01324301	0.036125833
ENSG00000142039	CCDC97	-0.30	0.013249534	0.036138704
ENSG00000095370	SH2D3C	1.10	0.013262114	0.036168087
ENSG00000059573	ALDH18A1	-0.17	0.013263949	0.036168163
ENSG00000250461	AC122718.1	0.82	0.013269356	0.036177977
ENSG00000138448	ITGAV	0.26	0.013273362	0.036183968
ENSG00000172586	CHCHD1	-0.27	0.013290529	0.036225833
ENSG00000068308	OTUD5	0.25	0.01329555	0.036234584
ENSG00000165525	NEMF	0.32	0.01332666	0.036314422
ENSG00000067225	PKM	0.19	0.013346771	0.036364273
ENSG00000176697	BDNF	-0.35	0.013373396	0.036431853
ENSG00000181513	ACBD4	-0.55	0.013377234	0.036432762
ENSG00000114541	FRMD4B	1.30	0.01337737	0.036432762
ENSG00000135108	FBXO21	-0.25	0.013380852	0.036437284
ENSG00000111144	LTA4H	-0.17	0.013415176	0.036522111
ENSG00000185482	STAC3	0.63	0.013415653	0.036522111
ENSG00000278000	AC139100.2	1.26	0.013439167	0.036581149
ENSG00000233532	LINC00460	0.50	0.013446869	0.036595751
ENSG00000137558	PI15	1.64	0.013448189	0.036595751
ENSG00000267796	LIN37	0.52	0.013455773	0.03661141
ENSG00000187626	ZKSCAN4	0.56	0.013463209	0.036626664
ENSG00000205937	RNPS1	-0.17	0.013477013	0.036659235
ENSG00000279369	AC046185.3	3.47	0.013483326	0.036671423
ENSG00000124802	EEF1E1	-0.32	0.013533437	0.03680271

ENSG00000105193	RPS16	-0.38	0.013549828	0.036842277
ENSG00000159377	PSMB4	0.19	0.013564199	0.036876341
ENSG00000075292	ZNF638	0.22	0.013607811	0.036989882
ENSG00000066405	CLDN18	2.15	0.013622657	0.037025209
ENSG00000022267	FHL1	0.40	0.013638361	0.037062858
ENSG00000126778	SIX1	-0.78	0.013653599	0.037099229
ENSG00000122026	RPL21	-0.31	0.013657466	0.037104698
ENSG00000179456	ZBTB18	0.63	0.013659463	0.037105085
ENSG00000278771	RN7SL3	1.10	0.01368106	0.037158709
ENSG00000169860	P2RY1	-0.76	0.01370036	0.037204931
ENSG00000170412	GPRC5C	0.61	0.013701796	0.037204931
ENSG00000148814	LRRC27	-0.82	0.013707706	0.037215929
ENSG00000182185	RAD51B	0.60	0.01377232	0.037386281
ENSG00000217165	ANKRD18EP	-0.86	0.013779366	0.037400336
ENSG00000035499	DEPDC1B	-0.28	0.013796493	0.037441744
ENSG00000205755	CRLF2	2.57	0.01381194	0.037478581
ENSG00000135974	C2orf49	-0.35	0.013821333	0.037498984
ENSG00000164845	FAM86FP	-1.74	0.013830019	0.037517465
ENSG00000206195	DUXAP8	-0.80	0.013836283	0.03752937
ENSG00000116120	FARSB	0.19	0.013847908	0.037550992
ENSG00000272512	AL645608.7	-1.01	0.013848007	0.037550992
ENSG00000171790	SLFNL1	-1.34	0.013871204	0.037608797
ENSG00000126883	NUP214	-0.29	0.013885424	0.037642253
ENSG00000227827	AC138969.2	-0.91	0.013896729	0.037667798
ENSG00000135521	LTV1	0.28	0.01390303	0.037679772
ENSG00000173482	PTPRM	-0.57	0.013912485	0.037700292
ENSG00000165533	TTC8	-0.44	0.013921963	0.037720868
ENSG00000171813	PWWP2B	0.43	0.013933911	0.037748129
ENSG00000142279	WTIP	0.49	0.013956192	0.037803373
ENSG00000170500	LONRF2	-0.50	0.013959653	0.037804973
ENSG00000119760	SUPT7L	0.26	0.013961712	0.037804973
ENSG00000210082	MT-RNR2	0.20	0.01396245	0.037804973
ENSG00000141219	C17orf80	0.34	0.014035728	0.03799824
ENSG00000184371	CSF1	0.60	0.014046015	0.038020946
ENSG00000168393	DTYMK	-0.22	0.014053153	0.038035122
ENSG00000136485	DCAF7	-0.15	0.014071727	0.038080244
ENSG00000164889	SLC4A2	0.15	0.014093858	0.038134976
ENSG00000226396	AL031727.1	-1.73	0.014107782	0.038167492
ENSG00000136870	ZNF189	0.45	0.014118836	0.038192233
ENSG00000237624	OXCT2P1	1.47	0.014135746	0.03823103
ENSG00000114942	EEF1B2	-0.15	0.014136999	0.03823103
ENSG00000204569	PPP1R10	-0.19	0.01413903	0.038231356
ENSG00000186132	C2orf76	0.69	0.014154455	0.038267894
ENSG00000235381	AL596202.1	-1.86	0.01417373	0.03831483
ENSG00000215859	PDZK1P1	-2.25	0.014182618	0.038329738
ENSG00000196419	XRCC6	-0.14	0.014183076	0.038329738

ENSG00000213930	GALT	-0.42	0.014210477	0.038394077
ENSG00000174446	SNAPC5	-0.37	0.01421072	0.038394077
ENSG00000163467	TSACC	-1.42	0.014249865	0.038491483
ENSG00000260265	LINC02562	1.34	0.014250619	0.038491483
ENSG00000158435	CNOT11	-0.20	0.014258001	0.038506224
ENSG00000153006	SREK1IP1	-0.35	0.014294525	0.038599653
ENSG00000083642	PDS5B	0.33	0.014300918	0.038611708
ENSG00000183840	GPR39	0.74	0.014339036	0.0387094
ENSG00000137460	FHDC1	0.69	0.014344901	0.038720012
ENSG00000221643	SNORA77	3.07	0.014358651	0.03875015
ENSG00000135951	TSGA10	0.82	0.01435994	0.03875015
ENSG00000119509	INVS	-0.40	0.014380752	0.03880108
ENSG00000065135	GNAI3	0.18	0.014396925	0.038839479
ENSG00000109133	TMEM33	-0.21	0.014421809	0.03889835
ENSG00000257913	DDN-AS1	-0.62	0.014422634	0.03889835
ENSG00000231389	HLA-DPA1	2.27	0.014431196	0.038916197
ENSG00000122545	SEPTIN7	0.18	0.014448324	0.038957136
ENSG00000158164	TMSB15A	0.47	0.014494934	0.039077544
ENSG00000248112	AC108174.1	-0.84	0.014500234	0.039086567
ENSG00000064651	SLC12A2	-0.27	0.014504047	0.039091582
ENSG00000273014	AC018645.3	-1.14	0.014514611	0.039114785
ENSG00000186187	ZNRF1	-0.30	0.014525998	0.039140202
ENSG00000067369	TP53BP1	0.27	0.014532522	0.039150558
ENSG00000099899	TRMT2A	-0.28	0.014535522	0.039150558
ENSG00000054523	KIF1B	0.22	0.014535711	0.039150558
ENSG00000262703	AC009121.3	-6.18	0.014548231	0.039179008
ENSG00000250317	SMIM20	0.31	0.01456358	0.039215065
ENSG00000107796	ACTA2	-1.07	0.014579323	0.039247753
ENSG00000135297	MTO1	0.26	0.014579642	0.039247753
ENSG00000225329	LHFPL3-AS2	5.10	0.014589645	0.039264118
ENSG00000240497	AC092919.1	5.10	0.014589645	0.039264118
ENSG00000198648	STK39	-0.16	0.014593137	0.039268234
ENSG00000278616	BEND3P3	-1.09	0.014600103	0.039281696
ENSG00000137960	GIPC2	3.01	0.01460735	0.039295911
ENSG00000159176	CSRP1	-0.20	0.014609422	0.039296202
ENSG00000138380	CARF	-0.83	0.014630092	0.039346513
ENSG00000120802	TMPO	-0.18	0.014634401	0.039352813
ENSG00000140848	CPNE2	-0.24	0.014649075	0.039386979
ENSG00000167904	TMEM68	-0.39	0.014652345	0.039390478
ENSG00000177125	ZBTB34	0.33	0.014668226	0.039427876
ENSG00000111052	LIN7A	-0.75	0.014678179	0.03944933
ENSG00000144481	TRPM8	2.06	0.014695834	0.039491477
ENSG00000154727	GABPA	0.32	0.014701494	0.039501383
ENSG00000222365	SNORD12B	-4.37	0.014708183	0.039514049
ENSG00000177374	HIC1	1.52	0.014710515	0.039515011
ENSG00000122574	WIPF3	-0.44	0.01472012	0.039535505

ENSG00000169857	AVEN	0.41	0.014741093	0.039586522
ENSG00000164828	SUN1	-0.17	0.014752603	0.039612115
ENSG00000108344	PSMD3	0.17	0.014764301	0.039638207
ENSG00000108479	GALK1	-0.29	0.014771963	0.039653458
ENSG00000169093	ASMTL	-0.28	0.014786841	0.039688071
ENSG00000105058	FAM32A	-0.26	0.014792766	0.039698649
ENSG00000129197	RPAIN	-0.25	0.014807667	0.039733305
ENSG00000109016	DHRS7B	-0.43	0.01480965	0.039733305
ENSG00000075089	ACTR6	0.33	0.014827204	0.039775069
ENSG00000089169	RPH3A	2.05	0.014831898	0.039782328
ENSG00000100077	GRK3	0.30	0.014842641	0.039803421
ENSG00000153207	AHCTF1	-0.22	0.01484374	0.039803421
ENSG00000137574	TGS1	-0.36	0.014890396	0.039920949
ENSG00000130783	CCDC62	0.95	0.014891559	0.039920949
ENSG00000120686	UFM1	-0.29	0.014908731	0.03996163
ENSG00000099968	BCL2L13	0.23	0.014927849	0.040007515
ENSG00000287200	AC022506.2	0.86	0.014936186	0.040024498
ENSG00000266278	LINC01910	-0.96	0.014961839	0.040087873
ENSG00000229618	AC011287.1	5.09	0.014990214	0.040158523
ENSG00000236670	KRT18P5	2.53	0.01499559	0.040164014
ENSG00000183291	SELENOF	0.19	0.014996278	0.040164014
ENSG00000274286	ADRA2B	1.56	0.015014949	0.040208639
ENSG00000244607	CCDC13	1.77	0.015085795	0.040392953
ENSG00000196943	NOP9	-0.23	0.015102333	0.04043058
ENSG00000254180	AC004083.1	4.34	0.015103889	0.04043058
ENSG00000164045	CDC25A	0.26	0.015116949	0.040460128
ENSG00000125257	ABCC4	-0.30	0.015120907	0.040465312
ENSG00000104848	KCNA7	2.12	0.015125323	0.040471715
ENSG00000205268	PDE7A	0.32	0.015128879	0.04047582
ENSG00000070731	ST6GALNAC2	-1.04	0.015142304	0.040506322
ENSG00000110427	KIAA1549L	1.27	0.01514967	0.040520608
ENSG00000117407	ARTN	0.29	0.015152678	0.040523239
ENSG00000100209	HSCB	0.49	0.015158929	0.040529499
ENSG00000257489	AC010203.1	-2.05	0.015159069	0.040529499
ENSG00000182272	B4GALNT4	-0.37	0.015162458	0.040533144
ENSG00000203326	ZNF525	-0.54	0.015190559	0.04060284
ENSG00000105289	TJP3	-0.28	0.01519668	0.040613776
ENSG00000224940	PRRT4	-0.90	0.015215394	0.040658362
ENSG00000089327	FXYD5	-0.27	0.0152329	0.040699707
ENSG00000107863	ARHGAP21	0.26	0.01523793	0.040707709
ENSG00000156873	PHKG2	-0.34	0.015240776	0.040709879
ENSG00000279088	AC022400.6	-0.38	0.015260056	0.040755936
ENSG00000224420	ADM5	-0.84	0.015278865	0.040800726
ENSG00000164898	FMC1	0.31	0.015324747	0.040917791
ENSG00000103888	CEMIP	-0.74	0.015336048	0.040942501
ENSG00000173889	PHC3	-0.32	0.015340284	0.040948348

ENSG00000100376	FAM118A	-0.29	0.015358024	0.040990234
ENSG00000173376	NDNF	2.71	0.015372542	0.041023512
ENSG00000197580	BCO2	0.72	0.015376612	0.041028901
ENSG00000175505	CLCF1	-0.52	0.015388528	0.041055223
ENSG00000120215	MLANA	1.85	0.015391938	0.041058845
ENSG00000164061	BSN	-0.94	0.015402174	0.041080676
ENSG00000274386	TMEM269	-1.78	0.015408095	0.041087622
ENSG00000135821	GLUL	-0.17	0.015408885	0.041087622
ENSG00000179611	DGKZP1	-0.40	0.015411993	0.041090436
ENSG00000263002	ZNF234	0.91	0.015442224	0.041165549
ENSG00000170836	PPM1D	-0.29	0.015477666	0.041254536
ENSG00000147383	NSDHL	0.30	0.015492471	0.041288498
ENSG00000163885	CFAP100	4.35	0.015495122	0.041290065
ENSG00000198435	NRARP	-0.32	0.015514327	0.041335736
ENSG00000231185	SPRY4-AS1	-1.65	0.015524527	0.041357405
ENSG00000124074	ENKD1	-0.46	0.015576137	0.041489373
ENSG00000166946	CCNDBP1	-0.28	0.015579348	0.041492402
ENSG00000204991	SPIRE2	-0.35	0.015595448	0.041529756
ENSG00000283761	AC118553.2	3.46	0.015633097	0.041618936
ENSG00000286558	AC098679.4	3.46	0.015633097	0.041618936
ENSG00000197927	C2orf27A	-2.48	0.015635677	0.041620268
ENSG00000074410	CA12	-0.63	0.01566937	0.041704406
ENSG00000156042	CFAP70	0.64	0.015675647	0.041715566
ENSG00000166407	LMO1	1.88	0.015677902	0.041716019
ENSG00000164074	ABHD18	-0.50	0.015685153	0.041726361
ENSG00000278997	AL662907.1	-2.54	0.015685959	0.041726361
ENSG00000113742	CPEB4	0.34	0.015717434	0.041800059
ENSG00000197694	SPTAN1	0.17	0.015719605	0.041800059
ENSG00000226086	EIF3LP3	5.11	0.01571993	0.041800059
ENSG00000243970	PPIEL	-0.62	0.015751239	0.041877745
ENSG00000285970	AC106820.6	3.00	0.015782771	0.041956005
ENSG00000277161	PIGW	-0.34	0.015800898	0.041998616
ENSG00000127980	PEX1	-0.41	0.015821291	0.042047234
ENSG00000281026	N4BP2L2-IT2	-0.94	0.015829226	0.04205618
ENSG00000149115	TNKS1BP1	-0.18	0.015830199	0.04205618
ENSG00000254505	CHMP4A	0.78	0.015830962	0.04205618
ENSG00000136159	NUDT15	0.20	0.015852292	0.042107257
ENSG00000171469	ZNF561	0.33	0.015861124	0.042125124
ENSG00000158301	GPRASP2	-1.09	0.015866958	0.042135028
ENSG00000182134	TDRKH	0.41	0.015889923	0.042185869
ENSG00000103544	VPS35L	-0.31	0.01589032	0.042185869
ENSG00000244425	RN7SL268P	-4.37	0.015893478	0.042188656
ENSG00000266412	NCOA4	0.21	0.01592399	0.042264044
ENSG00000251169	LINC01843	2.13	0.01593125	0.042277706
ENSG00000172840	PDP2	-0.32	0.015969041	0.042369877
ENSG00000145293	ENOPH1	-0.26	0.015970217	0.042369877

ENSG00000136908	DPM2	-0.25	0.015983574	0.042399693
ENSG00000005889	ZFX	0.36	0.015987992	0.042401577
ENSG00000272872	AP000525.1	-4.38	0.015988522	0.042401577
ENSG00000080298	RFX3	0.43	0.015995625	0.042414795
ENSG00000122696	SLC25A51	0.39	0.016000027	0.042420845
ENSG00000279696	AP001273.1	-1.17	0.01601233	0.04244784
ENSG00000127928	GNGT1	1.21	0.016033193	0.042497517
ENSG00000131459	GFPT2	-0.39	0.016053	0.042544382
ENSG00000167107	ACSF2	-0.36	0.016069431	0.042582289
ENSG00000176978	DPP7	-0.19	0.016077603	0.042598303
ENSG00000163932	PRKCD	0.22	0.016086275	0.042615638
ENSG00000166441	RPL27A	-0.16	0.016106108	0.042662532
ENSG00000172315	TP53RK	-0.33	0.016116714	0.042684976
ENSG00000205659	LIN52	0.33	0.016123638	0.042697664
ENSG00000143258	USP21	-0.40	0.016126735	0.042700215
ENSG00000135205	CCDC146	-0.75	0.016151567	0.042760306
ENSG00000116198	CEP104	-0.29	0.016154776	0.042763145
ENSG00000176809	LRRC37A3	0.73	0.016157237	0.042764002
ENSG00000239789	MRPS17	-0.47	0.016162463	0.042772178
ENSG00000196544	BORCS6	0.46	0.016188779	0.042836155
ENSG00000224020	MIR181A2HG	3.50	0.016198967	0.042857448
ENSG00000074935	TUBE1	-0.68	0.016204425	0.04286622
ENSG00000109790	KLHL5	0.29	0.016217049	0.042893944
ENSG00000183305	MAGEA2B	1.04	0.016227052	0.04291473
ENSG00000153179	RASSF3	-0.22	0.016232905	0.042924537
ENSG00000072954	TMEM38A	-0.47	0.016276569	0.043034311
ENSG00000113196	HAND1	3.08	0.016280848	0.043039939
ENSG00000160256	FAM207A	0.28	0.01628313	0.043040285
ENSG00000214357	NEURL1B	0.36	0.016293853	0.043062942
ENSG00000172869	DMXL1	0.36	0.016317053	0.04311856
ENSG00000167394	ZNF668	0.51	0.016357066	0.04321859
ENSG00000245060	LINC00847	-0.78	0.016383725	0.043283314
ENSG00000279759	AC118344.2	-1.08	0.016394959	0.043307277
ENSG00000172375	C2CD2L	0.31	0.016437857	0.043414861
ENSG00000213366	GSTM2	-1.50	0.016454452	0.043452954
ENSG00000271741	AC114490.2	-5.08	0.016460979	0.043459137
ENSG00000086827	ZW10	-0.31	0.016461136	0.043459137
ENSG00000161905	ALOX15	2.70	0.016468378	0.043472521
ENSG00000166135	HIF1AN	-0.17	0.016472099	0.043476608
ENSG00000270540	AC093915.1	-5.08	0.01648077	0.043488024
ENSG00000286988	AC024933.2	-5.08	0.01648077	0.043488024
ENSG00000156804	FBXO32	0.82	0.01648698	0.043498673
ENSG00000230882	AC005077.4	3.00	0.016489591	0.043499827
ENSG00000248538	AC022784.1	1.01	0.016511949	0.043553068
ENSG00000275029	HMGB1P24	3.41	0.016577969	0.043721443
ENSG00000119878	CRIPT	0.39	0.016588657	0.043743865

ENSG00000084112	SSH1	-0.17	0.016616325	0.043811054
ENSG00000197893	NRAP	3.02	0.01663408	0.04385209
ENSG00000138311	ZNF365	0.85	0.016665455	0.043929016
ENSG00000167749	KLK4	1.94	0.016687596	0.043978373
ENSG00000226539	MLXP1	1.41	0.016691329	0.043978373
ENSG00000221983	UBA52	-0.17	0.016692821	0.043978373
ENSG00000165097	KDM1B	-0.33	0.01669297	0.043978373
ENSG00000196504	PRPF40A	0.18	0.016740137	0.04409683
ENSG00000215784	FAM72D	0.42	0.016803347	0.044257512
ENSG00000185475	TMEM179B	-0.30	0.016818708	0.044286549
ENSG00000128563	PRKRIP1	0.29	0.016818797	0.044286549
ENSG00000147677	EIF3H	0.14	0.016843276	0.044342474
ENSG00000028277	POU2F2	1.13	0.016844468	0.044342474
ENSG00000232233	LINC02043	-1.78	0.016867222	0.044396535
ENSG00000088179	PTPN4	0.35	0.01690711	0.044495671
ENSG00000112559	MDFI	-0.34	0.016945777	0.044591569
ENSG00000139508	SLC46A3	0.65	0.01697597	0.044665147
ENSG00000149016	TUT1	-0.42	0.017013615	0.04475831
ENSG00000104983	CCDC61	0.78	0.01701841	0.044765041
ENSG00000031691	CENPQ	0.42	0.017035037	0.044802887
ENSG00000287431	AC027601.5	-0.75	0.017045482	0.044824465
ENSG00000226051	ZNF503-AS1	-1.01	0.017073726	0.04489284
ENSG00000112406	HECA	0.34	0.017104753	0.044968512
ENSG00000178809	TRIM73	-1.46	0.017111563	0.044980508
ENSG00000143322	ABL2	0.28	0.017129307	0.045021236
ENSG00000112851	ERBIN	-0.24	0.017136333	0.045033788
ENSG00000216937	CCDC7	0.96	0.017174509	0.045128188
ENSG00000140374	ETFA	0.20	0.01718335	0.04514549
ENSG00000148300	REXO4	0.25	0.017234288	0.045273375
ENSG00000113838	TBCCD1	0.34	0.017265055	0.045348245
ENSG00000168653	NDUFS5	0.17	0.017296825	0.04542573
ENSG00000121743	GJA3	-0.36	0.01730344	0.045437139
ENSG00000104221	BRF2	0.43	0.017322611	0.045481513
ENSG00000144908	ALDH1L1	-5.07	0.017367022	0.045586154
ENSG00000183281	PLGLB1	-5.07	0.017367022	0.045586154
ENSG00000064932	SBNO2	0.22	0.017391181	0.045643583
ENSG00000135750	KCNK1	0.34	0.017398258	0.045653101
ENSG00000111361	EIF2B1	-0.26	0.01739937	0.045653101
ENSG00000059588	TARBP1	-0.40	0.01742251	0.045707821
ENSG00000204946	ZNF783	-0.58	0.017463277	0.04580877
ENSG00000008018	PSMB1	0.16	0.017503212	0.045907506
ENSG00000131966	ACTR10	-0.25	0.017542906	0.046005587
ENSG00000167772	ANGPTL4	1.41	0.017565387	0.046053606
ENSG00000263033	AC007220.1	-5.09	0.017565819	0.046053606
ENSG00000139880	CDH24	-0.29	0.017573573	0.046067899
ENSG00000142512	SIGLEC10	-1.74	0.017595907	0.046118075

ENSG00000112511	PHF1	0.23	0.017597323	0.046118075
ENSG00000169372	CRADD	-0.46	0.017622289	0.046167276
ENSG00000130305	NSUN5	0.24	0.017622538	0.046167276
ENSG00000133112	TPT1	0.15	0.017623378	0.046167276
ENSG00000101440	ASIP	-3.45	0.017625324	0.046167276
ENSG00000184752	NDUFA12	0.30	0.017627841	0.046167826
ENSG00000213028	AL354983.1	1.99	0.017650974	0.046222363
ENSG00000120370	GORAB	0.56	0.017656043	0.046224005
ENSG00000172301	COPRS	-0.30	0.017658022	0.046224005
ENSG00000125454	SLC25A19	-0.31	0.017658531	0.046224005
ENSG00000176945	MUC20	-1.30	0.017681082	0.046276984
ENSG00000036448	MYOM2	0.62	0.017685192	0.046278681
ENSG00000131051	RBM39	0.16	0.017686356	0.046278681
ENSG00000091127	PUS7	-0.30	0.017737639	0.046406802
ENSG00000130640	TUBGCP2	-0.25	0.017765196	0.046472823
ENSG00000013364	MVP	-0.21	0.017769546	0.046478128
ENSG00000178297	TMPRSS9	-1.00	0.01778168	0.046503788
ENSG00000140521	POLG	0.20	0.017792056	0.046524843
ENSG00000110921	MVK	-0.30	0.017795051	0.046526595
ENSG00000143436	MRPL9	0.22	0.017818118	0.046580821
ENSG00000138379	MSTN	2.95	0.017828053	0.046591954
ENSG00000134146	DPH6	-0.47	0.017828774	0.046591954
ENSG00000163689	CFAP20DC	0.69	0.017829362	0.046591954
ENSG00000105865	DUS4L	-0.67	0.017843233	0.046619413
ENSG00000139618	BRCA2	-0.39	0.017844529	0.046619413
ENSG00000138083	SIX3	0.94	0.017873181	0.046688173
ENSG00000129103	SUMF2	-0.18	0.017890639	0.046727677
ENSG00000131263	RLIM	0.24	0.01789708	0.046738401
ENSG00000107104	KANK1	-0.36	0.017911019	0.046768701
ENSG00000130985	UBA1	0.14	0.017930685	0.046813943
ENSG00000145241	CENPC	0.40	0.017936539	0.046819656
ENSG00000132205	EMILIN2	0.33	0.017937552	0.046819656
ENSG00000270872	SRGAP2D	0.97	0.01795455	0.04685239
ENSG00000143387	CTSK	0.73	0.017954776	0.04685239
ENSG00000213062	Z99572.1	-1.43	0.017957851	0.046854305
ENSG00000285644	AC108448.3	-2.49	0.017962634	0.046860675
ENSG00000272040	AC010245.2	-2.33	0.01796643	0.046864468
ENSG00000198839	ZNF277	0.43	0.017972533	0.046874277
ENSG00000166575	TMEM135	0.24	0.017987584	0.04690742
ENSG00000183978	COA3	0.28	0.018002255	0.04693956
ENSG00000231966	LINC02818	4.27	0.018007252	0.046946472
ENSG00000128829	EIF2AK4	-0.23	0.018022276	0.046979521
ENSG00000225507	AC069282.1	-0.98	0.018027941	0.046988167
ENSG00000174226	SNX31	4.27	0.018037018	0.046999942
ENSG00000224080	UBE2FP1	-2.69	0.018037155	0.046999942
ENSG00000134769	DTNA	0.57	0.018052997	0.047035096

ENSG00000164418	GRIK2	2.49	0.018072296	0.047079248
ENSG00000164332	UBLCP1	0.29	0.018075444	0.047081321
ENSG00000132849	PATJ	-0.31	0.018122439	0.047197585
ENSG00000250571	GLI4	-0.34	0.018147354	0.047256325
ENSG00000162415	ZSWIM5	0.92	0.01818648	0.047352049
ENSG00000236287	ZBED5	0.31	0.01820611	0.047396991
ENSG00000116014	KISS1R	0.69	0.018237297	0.047466897
ENSG00000088930	XRN2	-0.18	0.018237706	0.047466897
ENSG00000173175	ADCY5	-1.55	0.018261363	0.047522289
ENSG00000187634	SAMD11	0.93	0.018266877	0.047530456
ENSG00000227262	HCG4B	4.28	0.018280865	0.047560671
ENSG00000108176	DNAJC12	0.66	0.018305417	0.047613692
ENSG00000092201	SUPT16H	0.15	0.018306004	0.047613692
ENSG00000103966	EHD4	0.18	0.018326296	0.047660279
ENSG00000175193	PARL	0.34	0.018340047	0.047689843
ENSG00000135097	MSI1	-0.35	0.018436652	0.047934817
ENSG00000259146	AC005476.2	-1.63	0.018466462	0.048006085
ENSG00000251521	IMPA1P1	2.94	0.018470843	0.048011236
ENSG00000118855	MFSD1	0.30	0.018494123	0.048065506
ENSG00000197857	ZNF44	0.68	0.018508149	0.048095711
ENSG00000166987	MBD6	-0.30	0.018526507	0.048137166
ENSG00000112343	TRIM38	0.34	0.018533329	0.04814864
ENSG00000010256	UQCRC1	-0.16	0.018548203	0.04817523
ENSG00000125977	EIF2S2	0.15	0.018548378	0.04817523
ENSG00000248161	AC098487.1	-0.88	0.018604216	0.048308298
ENSG00000237493	AC034102.1	0.50	0.01860444	0.048308298
ENSG00000263874	LINC00672	-2.96	0.018615927	0.048331853
ENSG00000166997	CNPY4	0.73	0.018635044	0.048375211
ENSG00000002746	HECW1	0.49	0.018646311	0.048398181
ENSG00000009954	BAZ1B	0.15	0.018660771	0.04842323
ENSG00000169738	DCXR	-0.21	0.018660801	0.04842323
ENSG00000010322	NISCH	-0.21	0.018676277	0.048457105
ENSG00000119041	GTF3C3	0.25	0.018695975	0.048499068
ENSG00000233845	AC093732.1	-2.69	0.018697297	0.048499068
ENSG00000116260	QSOX1	-0.18	0.018734456	0.048589156
ENSG00000272733	AP000345.2	-2.49	0.01878256	0.048707604
ENSG00000130724	CHMP2A	-0.20	0.01882007	0.048798553
ENSG00000076604	TRAF4	0.18	0.018856717	0.048887241
ENSG00000250474	WBP1LP2	1.85	0.018875311	0.04892911
ENSG00000196116	TDRD7	0.45	0.018926764	0.049056132
ENSG00000124356	STAMBP	-0.24	0.018932294	0.049064112
ENSG00000250548	LINC01303	1.09	0.018964481	0.049141161
ENSG00000242242	NECTIN3-AS1	-3.45	0.019014823	0.04926523
ENSG00000133216	EPHB2	-0.29	0.019104357	0.049490795
ENSG00000147548	NSD3	-0.24	0.019116902	0.049516884
ENSG00000157895	C12orf43	0.29	0.019132864	0.04954801

ENSG00000156026	MCU	-0.27	0.019133871	0.04954801
ENSG00000120820	GLT8D2	0.54	0.01917067	0.049629646
ENSG00000101224	CDC25B	0.16	0.019170946	0.049629646
ENSG00000008311	AASS	0.58	0.019172836	0.049629646
ENSG00000171443	ZNF524	-0.52	0.019177734	0.049635903
ENSG00000205702	CYP2D7	-1.19	0.019184826	0.049647839
ENSG00000129473	BCL2L2	0.22	0.019204333	0.049691894
ENSG00000287016	AC010996.1	-2.48	0.019224615	0.04973585
ENSG00000171729	TMEM51	0.28	0.019226291	0.04973585
ENSG00000119335	SET	-0.13	0.019247132	0.049783327
ENSG00000163481	RNF25	0.30	0.019268118	0.049831168
ENSG00000186642	PDE2A	-0.46	0.019295196	0.049892667
ENSG00000283828	AL137002.2	1.80	0.019296884	0.049892667
ENSG00000127124	HIVEP3	-0.45	0.019366732	0.050066792
ENSG00000273002	AL355388.1	-1.02	0.019383731	0.050104265
ENSG00000276141	WHAMMP3	-1.10	0.019402495	0.050146292
ENSG00000269374	AC011497.2	1.12	0.019420185	0.050185529
ENSG00000106688	SLC1A1	0.53	0.019437281	0.050223225
ENSG00000004799	PDK4	1.15	0.019447874	0.050244107
ENSG00000187045	TMPRSS6	-1.13	0.019462864	0.050272329
ENSG00000152520	PAN3	0.26	0.019463822	0.050272329
ENSG00000181104	F2R	0.33	0.019471176	0.050284834
ENSG00000130962	PRRG1	-0.37	0.019477222	0.050293958
ENSG00000198933	TBKBP1	-0.38	0.019520451	0.05039908
ENSG00000134852	CLOCK	-0.27	0.019570764	0.050522462
ENSG00000102908	NFAT5	-0.42	0.019631451	0.050672591
ENSG00000161082	CELF5	0.60	0.019644311	0.050699247
ENSG00000160710	ADAR	-0.16	0.019669577	0.050757908
ENSG00000100902	PSMA6	0.22	0.019765198	0.050991555
ENSG00000128408	RIBC2	-0.75	0.019765215	0.050991555
ENSG00000040341	STAU2	0.29	0.01978785	0.051043369
ENSG00000138101	DTNB	-0.40	0.019852854	0.051204448
ENSG00000129521	EGLN3	0.56	0.0198591	0.051209111
ENSG00000130881	LRP3	0.28	0.01985978	0.051209111
ENSG00000133065	SLC41A1	0.21	0.019879944	0.0512545
ENSG00000174306	ZHX3	-0.35	0.019885573	0.051262411
ENSG00000278558	TMEM191B	1.85	0.019891566	0.051271255
ENSG00000130818	ZNF426	-0.78	0.019896464	0.051277275
ENSG00000225062	CATIP-AS1	-3.38	0.01992235	0.051337378
ENSG00000138100	TRIM54	-1.27	0.019938151	0.051371479
ENSG00000105519	CAPS	-1.20	0.019979332	0.051470958
ENSG00000143622	RIT1	-0.46	0.01998717	0.051484522
ENSG00000266680	AL135905.1	-1.87	0.019991696	0.051489552
ENSG00000273253	AL022328.4	-0.75	0.020022008	0.051560987
ENSG00000240882	AC063952.2	4.98	0.020044541	0.051612371
ENSG00000243648	AC109454.1	4.98	0.020061153	0.051641857

ENSG00000255663	AP002373.1	4.98	0.020061153	0.051641857
ENSG00000212802	RPL15P3	0.20	0.020064878	0.051644803
ENSG00000112139	MDGA1	0.61	0.020116498	0.051771008
ENSG00000152642	GPD1L	-0.30	0.020144132	0.05183546
ENSG00000162604	TM2D1	0.35	0.020153157	0.051852014
ENSG00000110344	UBE4A	0.27	0.020179844	0.051911026
ENSG00000171100	MTM1	0.60	0.02018128	0.051911026
ENSG00000224861	YBX1P1	0.37	0.020203783	0.05196223
ENSG00000067704	IARS2	-0.16	0.02020951	0.05197028
ENSG00000180644	PRF1	0.44	0.020212729	0.051971879
ENSG00000130803	ZNF317	-0.26	0.020233261	0.052017987
ENSG00000163606	CD200R1	4.28	0.020237826	0.05202304
ENSG00000182405	PGBD4	-0.82	0.02026393	0.052083453
ENSG00000100916	BRMS1L	0.48	0.020272268	0.052098193
ENSG00000181090	EHMT1	0.21	0.020334036	0.052250221
ENSG00000185753	CXorf38	-0.37	0.020381965	0.052366656
ENSG00000232756	AC004990.1	4.97	0.020427905	0.052477573
ENSG00000287009	AC021037.1	1.95	0.02043038	0.052477573
ENSG00000141873	SLC39A3	0.27	0.020439041	0.052493082
ENSG00000171951	SCG2	0.46	0.020469655	0.052560551
ENSG00000205352	PRR13	-0.20	0.020471287	0.052560551
ENSG00000060566	CREB3L3	1.32	0.020475193	0.052560551
ENSG00000259291	ZNF710-AS1	-1.53	0.020475817	0.052560551
ENSG00000132463	GRSF1	-0.15	0.02049535	0.052603944
ENSG00000103275	UBE2I	-0.17	0.020518717	0.052652745
ENSG00000127774	EMC6	-0.31	0.020519626	0.052652745
ENSG00000183354	KIAA2026	0.37	0.020559737	0.052748904
ENSG00000137944	KYAT3	-0.40	0.020582989	0.052801791
ENSG00000135932	CAB39	0.20	0.020614419	0.052875641
ENSG00000132017	DCAF15	0.24	0.020675657	0.053019482
ENSG00000232104	RFX3-AS1	1.84	0.020675796	0.053019482
ENSG00000097033	SH3GLB1	0.22	0.020695921	0.053058759
ENSG00000175868	CALCB	-2.04	0.020696762	0.053058759
ENSG00000149273	RPS3	-0.17	0.020699067	0.053058759
ENSG00000278974	AC093909.6	0.86	0.020704647	0.053066265
ENSG00000065534	MYLK	-0.67	0.020722795	0.053105976
ENSG00000110046	ATG2A	0.32	0.020759685	0.053193704
ENSG00000172172	MRPL13	0.20	0.020791301	0.053267893
ENSG00000126790	L3HYPDH	0.52	0.020860552	0.053438474
ENSG00000175600	SUGCT	-0.79	0.020936504	0.053619944
ENSG00000113712	CSNK1A1	0.39	0.02093675	0.053619944
ENSG00000171456	ASXL1	0.19	0.020964261	0.053681538
ENSG00000141098	GFOD2	0.33	0.020966165	0.053681538
ENSG00000104808	DHDH	0.57	0.020970532	0.053685849
ENSG00000183340	JRKL	-0.42	0.02097822	0.053698663
ENSG00000075790	BCAP29	0.74	0.020983248	0.053704662

ENSG00000184194	GPR173	-0.59	0.021006051	0.053756151
ENSG00000174776	WDR49	3.41	0.021012881	0.053766753
ENSG00000183337	BCOR	-0.29	0.021019777	0.053777522
ENSG00000261716	H2BC20P	-0.62	0.021025629	0.053785618
ENSG00000151575	TEX9	-0.87	0.021057607	0.053860536
ENSG00000157216	SSBP3	-0.28	0.02106469	0.053871767
ENSG00000126878	AIF1L	0.19	0.021093608	0.053934995
ENSG00000162676	GFI1	-0.46	0.021094803	0.053934995
ENSG00000081320	STK17B	0.38	0.021171536	0.05412427
ENSG00000264391	RN7SL208P	4.97	0.021176286	0.054129497
ENSG00000243038	AL117692.1	4.99	0.021201609	0.054180384
ENSG00000258556	LINC02322	4.99	0.021201609	0.054180384
ENSG00000148700	ADD3	0.27	0.021207597	0.054188767
ENSG00000103653	CSK	0.23	0.021280393	0.054367832
ENSG00000132561	MATN2	-0.78	0.021289294	0.054383629
ENSG00000261189	AL031058.1	1.09	0.021309	0.054427021
ENSG00000207975	MIR181B1	4.20	0.021315855	0.054430635
ENSG00000214249	CTAGE11P	4.20	0.021315855	0.054430635
ENSG00000139154	AEBP2	-0.30	0.021319357	0.054432633
ENSG00000235944	ZNF815P	1.30	0.021364596	0.054541177
ENSG00000163913	IFT122	0.31	0.021376594	0.054564846
ENSG00000128590	DNAJB9	0.61	0.021383705	0.054576036
ENSG00000113272	THG1L	-0.31	0.021402767	0.054617721
ENSG00000180035	ZNF48	0.30	0.02142733	0.054673432
ENSG00000237296	SMG1P1	-0.43	0.021430955	0.054675711
ENSG00000170209	ANKK1	-2.64	0.021437629	0.054685766
ENSG00000187123	LYPD6	-0.50	0.02146533	0.054749451
ENSG00000109861	CTSC	-0.28	0.021569507	0.055008153
ENSG00000251365	LINC02236	1.75	0.021588666	0.055049997
ENSG00000175931	UBE2O	-0.18	0.021652957	0.055206902
ENSG00000163864	NMNAT3	-0.76	0.021708708	0.055335927
ENSG00000225630	MTND2P28	0.17	0.021709093	0.055335927
ENSG00000225484	NUTM2B-AS1	0.40	0.02171844	0.055352701
ENSG00000267040	AC027097.1	-0.95	0.021725339	0.055363236
ENSG00000185339	TCN2	0.64	0.021782057	0.055500703
ENSG00000285712	AC068707.1	-1.71	0.021853834	0.055676502
ENSG00000171109	MFN1	-0.27	0.021917731	0.055832185
ENSG00000236675	MTX1P1	-1.04	0.021945734	0.055896401
ENSG00000132122	SPATA6	-0.73	0.021971899	0.055955923
ENSG00000253683	AC027309.2	0.35	0.021977245	0.055962415
ENSG00000272916	AC022400.5	-2.27	0.022029808	0.056089124
ENSG00000187824	TMEM220	-2.97	0.022063022	0.056166542
ENSG00000078246	TULP3	-0.27	0.022077476	0.05619619
ENSG00000134569	LRP4	0.36	0.022102058	0.056251608
ENSG00000260118	AL157700.1	-2.43	0.022151711	0.056370809
ENSG00000003147	ICA1	-0.50	0.02216589	0.056396015

ENSG00000259877	AC009113.1	0.74	0.022167252	0.056396015
ENSG00000132702	HAPLN2	-2.92	0.022209247	0.056495672
ENSG00000227479	AC124861.1	1.90	0.022232156	0.05654343
ENSG00000123349	PFDN5	0.17	0.022233672	0.05654343
ENSG00000183570	PCBP3	-1.26	0.022285534	0.056668121
ENSG00000131845	ZNF304	0.48	0.022297101	0.05669033
ENSG00000117298	ECE1	-0.16	0.022335819	0.056781556
ENSG00000099256	PRTFDC1	4.97	0.022415966	0.056978065
ENSG00000126107	HECTD3	-0.25	0.022442143	0.057037359
ENSG00000260669	AL096870.2	1.07	0.022467087	0.057092186
ENSG00000167617	CDC42EP5	1.86	0.022469421	0.057092186
ENSG00000180178	FAR2P1	-1.46	0.022480803	0.057113855
ENSG00000078043	PIAS2	-0.33	0.022494669	0.057141827
ENSG00000145020	AMT	-2.04	0.022512739	0.057180472
ENSG00000185332	TMEM105	1.04	0.022531623	0.057221173
ENSG00000250479	CHCHD10	-0.24	0.022541462	0.057233974
ENSG00000106852	LHX6	0.53	0.022542384	0.057233974
ENSG00000150054	MPP7	0.24	0.022582112	0.05732757
ENSG00000095380	NANS	0.21	0.02259817	0.057361058
ENSG00000111907	TPD52L1	-0.25	0.022604086	0.057368798
ENSG00000139625	MAP3K12	0.28	0.022614513	0.057387983
ENSG00000167984	NLRC3	-1.90	0.02266703	0.057513595
ENSG00000225156	AC012354.1	4.21	0.02266976	0.057513595
ENSG00000205810	KLRC3	-2.63	0.02270952	0.057607164
ENSG00000159921	GNE	0.33	0.022715539	0.05761513
ENSG00000103264	FBXO31	0.21	0.022720196	0.057619639
ENSG00000272977	AL008721.2	-1.34	0.022732169	0.057642698
ENSG00000225526	MKRN2OS	-2.48	0.022766165	0.057721588
ENSG00000198134	PTMAP9	-1.75	0.022797734	0.057794307
ENSG00000127995	CASD1	-0.60	0.02280599	0.057807914
ENSG00000243696	AC006254.1	-1.59	0.022841175	0.0578869
ENSG00000105854	PON2	0.24	0.022842937	0.0578869
ENSG00000107738	VSIR	-0.30	0.022853838	0.057903463
ENSG00000160345	C9orf116	-0.49	0.02285771	0.057903463
ENSG00000213190	MLLT11	1.19	0.022860644	0.057903463
ENSG00000143162	CREG1	-0.22	0.022861047	0.057903463
ENSG00000168564	CDKN2AIP	0.50	0.022872305	0.057924647
ENSG00000267462	AC090377.1	3.34	0.022892141	0.057967547
ENSG00000260645	AL359715.2	-2.63	0.022906636	0.057996912
ENSG00000183506	PI4KAP2	-0.37	0.022953698	0.058104228
ENSG00000123810	B9D2	0.57	0.022954828	0.058104228
ENSG00000113013	HSPA9	0.13	0.022969891	0.058135001
ENSG00000226824	AC006001.2	-1.32	0.022982982	0.058160778
ENSG00000164124	TMEM144	-0.51	0.023007727	0.058216037
ENSG00000121749	TBC1D15	0.27	0.023012082	0.058219694
ENSG00000186352	ANKRD37	0.69	0.023035921	0.058272638

ENSG00000006194	ZNF263	0.25	0.023059525	0.058324977
ENSG00000213453	FTH1P3	1.82	0.023129791	0.05849531
ENSG00000170638	TRABD	-0.21	0.023151279	0.058542254
ENSG00000115129	TP53I3	0.41	0.02320262	0.058664665
ENSG00000114026	OGG1	-0.33	0.023212033	0.058681051
ENSG00000170310	STX8	0.38	0.023246932	0.058761854
ENSG00000056736	IL17RB	-0.44	0.023263379	0.058796002
ENSG00000182199	SHMT2	-0.16	0.023301305	0.05888442
ENSG00000170498	KISS1	1.77	0.023308584	0.058891463
ENSG00000277840	AC026368.1	-5.06	0.023309978	0.058891463
ENSG00000010626	LRRC23	-0.47	0.023342182	0.058965381
ENSG00000274828	AC068473.5	-0.68	0.023348215	0.058973178
ENSG00000205078	SYCE1L	-0.64	0.023385648	0.059060272
ENSG00000090612	ZNF268	0.48	0.023404675	0.059099031
ENSG00000275700	AATF	0.17	0.023409451	0.059099031
ENSG00000258289	CHURC1	-0.31	0.023409855	0.059099031
ENSG00000119280	C1orf198	-0.23	0.023416348	0.059107495
ENSG00000117115	PADI2	-0.73	0.023419115	0.059107495
ENSG00000111669	TPI1	-0.34	0.023422322	0.059108136
ENSG00000131174	COX7B	0.21	0.023435356	0.059133569
ENSG00000232712	KIZ-AS1	-4.96	0.023454612	0.059174696
ENSG00000186665	C17orf58	-0.26	0.023477167	0.059224134
ENSG00000258454	AC016526.2	3.34	0.023485123	0.059236738
ENSG00000103034	NDRG4	0.40	0.023492914	0.059242337
ENSG00000106268	NUDT1	-0.30	0.023493263	0.059242337
ENSG00000096872	IFT74	0.41	0.023583035	0.059461219
ENSG00000263316	AC027763.2	-2.36	0.023589815	0.059470821
ENSG00000140694	PARN	-0.23	0.0235955	0.059477661
ENSG00000068078	FGFR3	-0.26	0.023613868	0.059516463
ENSG00000136938	ANP32B	0.14	0.023624076	0.059533382
ENSG00000176399	DMRTA1	2.88	0.02362653	0.059533382
ENSG00000280374	AC019080.5	-1.70	0.023670214	0.059635947
ENSG00000119326	CTNNAL1	0.20	0.023702373	0.059709452
ENSG00000213214	ARHGEF35	-0.44	0.023708813	0.059718158
ENSG00000074527	NTN4	0.92	0.02371817	0.059734208
ENSG00000116922	C1orf109	0.24	0.023729106	0.059754231
ENSG00000251034	AC087854.1	-2.43	0.02375127	0.059802518
ENSG00000152104	PTPN14	0.50	0.023777272	0.059860456
ENSG00000213762	ZNF134	-0.53	0.023783093	0.05986758
ENSG00000221817	PPP3CB-AS1	-0.96	0.023798546	0.059893599
ENSG00000268756	AC104534.1	-0.99	0.023799415	0.059893599
ENSG00000105835	NAMPT	0.18	0.023832787	0.059962541
ENSG00000204356	NELFE	0.19	0.023832803	0.059962541
ENSG00000170579	DLGAP1	-2.19	0.02383977	0.059972531
ENSG00000204227	RING1	0.19	0.023933697	0.060201249
ENSG00000066697	MSANTD3	-0.27	0.023976904	0.06030235

ENSG00000161328	LRRC56	-0.61	0.023996279	0.060343495
ENSG00000274414	AL121772.1	-1.90	0.024003725	0.060354636
ENSG00000100219	XBP1	-0.19	0.024010337	0.060363677
ENSG00000262700	AC133552.3	-4.95	0.024052074	0.060461011
ENSG00000148688	RPP30	0.24	0.024064191	0.060483872
ENSG00000095383	TBC1D2	-0.38	0.024068121	0.060486154
ENSG00000133028	SCO1	-0.23	0.024088741	0.060525288
ENSG00000198964	SGMS1	0.27	0.024089742	0.060525288
ENSG00000105750	ZNF85	-1.02	0.024096135	0.060533749
ENSG00000103274	NUBP1	0.28	0.024156647	0.06067815
ENSG00000221886	ZBED8	-0.62	0.024164077	0.060689195
ENSG00000122042	UBL3	0.38	0.024178258	0.060717191
ENSG00000236790	LINC00299	-4.96	0.024253814	0.060891649
ENSG00000267152	AC093227.1	-4.96	0.024253814	0.060891649
ENSG00000275494	AC133552.5	-1.34	0.02428309	0.060950894
ENSG00000260442	ATP2A1-AS1	-0.69	0.024286734	0.060950894
ENSG00000154099	DNAAF1	-1.61	0.02428753	0.060950894
ENSG00000285676	AL158212.5	-1.80	0.024289595	0.060950894
ENSG00000177200	CHD9	0.35	0.024365182	0.061132902
ENSG00000074603	DPP8	0.25	0.024368924	0.061134626
ENSG00000184163	C1QTNF12	-1.33	0.024381612	0.061158789
ENSG00000137880	GCHFR	1.16	0.024405115	0.061203206
ENSG00000233621	LINC01137	0.96	0.024405436	0.061203206
ENSG00000067365	METTL22	-0.39	0.024430659	0.061258783
ENSG00000029725	RABEP1	0.23	0.024513559	0.061458951
ENSG00000169885	CALML6	-1.67	0.024520022	0.061467453
ENSG00000163121	NEURL3	2.59	0.024551939	0.061539356
ENSG00000111224	PARP11	-0.89	0.024554855	0.061539356
ENSG00000011376	LARS2	-0.23	0.024570969	0.061572031
ENSG00000166963	MAP1A	0.73	0.024599236	0.061635146
ENSG00000140950	MEAK7	0.25	0.024614071	0.061664596
ENSG00000223735	OR51B3P	-2.42	0.024660627	0.061773498
ENSG00000163357	DCST1	-1.67	0.024685494	0.061828049
ENSG00000112739	PRPF4B	-0.21	0.024689336	0.061829933
ENSG00000101152	DNAJC5	0.17	0.02469703	0.061841462
ENSG00000245694	CRNDE	-0.32	0.024718672	0.06188791
ENSG00000133138	TBC1D8B	0.40	0.024753029	0.061966178
ENSG00000133962	CATSPERB	2.30	0.024775917	0.062015715
ENSG00000164291	ARSK	0.51	0.024783998	0.062028186
ENSG00000221930	DENND10P1	-0.88	0.024816171	0.06210094
ENSG00000129636	ITFG1	0.27	0.024856196	0.062193322
ENSG00000228532	AC005000.1	0.72	0.02490418	0.062305594
ENSG00000284879	AC133644.2	0.95	0.024929493	0.062360037
ENSG00000159625	DRC7	-1.93	0.024932173	0.062360037
ENSG00000151151	IPMK	0.38	0.024968425	0.062442905
ENSG00000124496	TRERF1	-0.36	0.024979374	0.062462481

ENSG00000186625	KATNA1	0.32	0.024995652	0.062490245
ENSG00000152234	ATP5F1A	0.14	0.024996722	0.062490245
ENSG00000137942	FNBP1L	-0.24	0.025011327	0.062518945
ENSG00000169895	SYAP1	0.23	0.025050653	0.062609425
ENSG00000228649	SNHG26	-0.47	0.025075702	0.062664204
ENSG00000287710	AC067852.5	-1.00	0.025100068	0.062717264
ENSG00000146233	CYP39A1	1.08	0.025103476	0.062717947
ENSG00000261949	GFY	0.47	0.025136071	0.062791542
ENSG00000287735	AL355075.6	-1.33	0.025144028	0.06280358
ENSG00000160213	CSTB	-0.19	0.025169867	0.062860274
ENSG00000256043	CTSO	-1.60	0.025177988	0.06287271
ENSG00000113657	DPYSL3	-0.28	0.025189164	0.06289277
ENSG00000260428	SCX	-1.33	0.025224213	0.062969302
ENSG00000175213	ZNF408	0.36	0.025227457	0.062969302
ENSG00000067182	TNFRSF1A	0.16	0.025230348	0.062969302
ENSG00000280347	AC000123.3	-1.27	0.025232402	0.062969302
ENSG00000170293	CMTM8	-0.39	0.025237369	0.062973844
ENSG00000129315	CCNT1	0.21	0.025272438	0.063053489
ENSG00000278011	AC024941.1	-4.94	0.025281645	0.063066515
ENSG00000187446	CHP1	0.17	0.025283962	0.063066515
ENSG00000106404	CLDN15	-0.43	0.025288024	0.063068786
ENSG00000118762	PKD2	-0.36	0.025294581	0.063077279
ENSG00000285875	AL035446.2	0.97	0.025329492	0.063156465
ENSG00000171848	RRM2	-0.14	0.025459834	0.063473552
ENSG00000164039	BDH2	0.71	0.025463904	0.06347579
ENSG00000237118	CYP2F2P	1.99	0.025512055	0.063587899
ENSG00000184451	CCR10	1.08	0.025536363	0.06364056
ENSG00000288559	AC016394.2	-0.80	0.025540433	0.063642777
ENSG00000167244	IGF2	1.85	0.0255597	0.063682858
ENSG00000200087	SNORA73B	-1.07	0.025569106	0.063698363
ENSG00000047346	FAM214A	0.74	0.025590246	0.063743092
ENSG00000119705	SLIRP	0.21	0.025597373	0.06375291
ENSG00000115211	EIF2B4	0.22	0.025609928	0.063776241
ENSG00000152404	CWF19L2	0.53	0.025634727	0.063830057
ENSG00000205213	LGR4	0.47	0.025659785	0.0638845
ENSG00000237412	PRSS56	4.12	0.025729992	0.064051324
ENSG00000259408	AC010809.2	-4.14	0.025767838	0.064137558
ENSG00000181085	MAPK15	-0.43	0.025790536	0.064185261
ENSG00000243667	WDR92	-0.74	0.025795256	0.064185261
ENSG00000177363	LRRN4CL	-4.14	0.025796625	0.064185261
ENSG00000005471	ABCB4	4.11	0.025805992	0.064197742
ENSG00000166821	PEX11A	0.48	0.025808057	0.064197742
ENSG00000238123	MID1IP1-AS1	-1.31	0.025813864	0.064198024
ENSG00000025770	NCAPH2	-0.17	0.025814586	0.064198024
ENSG00000162378	ZYG11B	-0.33	0.025818755	0.064200414
ENSG00000276853	AC026124.2	-4.96	0.02582396	0.064205379

ENSG00000116685	KIAA2013	-0.19	0.025844071	0.064247398
ENSG00000008086	CDKL5	0.56	0.025890622	0.064355128
ENSG00000108312	UBTF	0.15	0.025908325	0.064391132
ENSG00000158077	NLRP14	1.99	0.025931702	0.06444123
ENSG00000214192	UBE2V1P2	-0.93	0.025955741	0.064490035
ENSG00000163633	C4orf36	-2.00	0.025957787	0.064490035
ENSG00000135823	STX6	0.23	0.025968604	0.064502575
ENSG00000276404	MIR6835	-1.81	0.025970319	0.064502575
ENSG00000100246	DNAL4	-0.31	0.025972504	0.064502575
ENSG00000128886	ELL3	0.68	0.025980328	0.064509541
ENSG00000135723	FHOD1	0.23	0.025981756	0.064509541
ENSG00000143153	ATP1B1	0.20	0.026003702	0.064556021
ENSG00000148488	ST8SIA6	-1.02	0.026047892	0.064657703
ENSG00000131495	NDUFA2	-0.22	0.02606516	0.064686723
ENSG00000129351	ILF3	-0.13	0.026066047	0.064686723
ENSG00000052126	PLEKHA5	0.34	0.026149465	0.06488569
ENSG00000278493	AC039056.2	2.43	0.026176885	0.064945677
ENSG00000225190	PLEKHM1	-0.29	0.026191396	0.064973624
ENSG00000163472	TMEM79	-0.42	0.026224477	0.065047624
ENSG00000161956	SENP3	-0.21	0.026234222	0.065063732
ENSG00000279792	AC015909.5	-2.26	0.026256217	0.065110212
ENSG00000224892	RPS4XP16	-4.14	0.026264913	0.065118492
ENSG00000136931	NR5A1	2.23	0.026266064	0.065118492
ENSG00000082212	ME2	-0.24	0.026324932	0.065252265
ENSG00000180855	ZNF443	0.91	0.026326544	0.065252265
ENSG00000161016	RPL8	-0.18	0.026333404	0.065261187
ENSG00000282458	WASH5P	0.54	0.026379745	0.065367937
ENSG00000188185	LINC00265	-0.68	0.026407378	0.065427402
ENSG00000023516	AKAP11	-0.26	0.026412314	0.065427402
ENSG00000273486	AC096992.2	-2.28	0.026413551	0.065427402
ENSG00000049883	PTCD2	-0.35	0.026437596	0.065478859
ENSG00000261338	AC021016.1	-2.01	0.026453189	0.06550937
ENSG00000130147	SH3BP4	0.18	0.026462757	0.065521707
ENSG00000111788	AC009533.1	-0.43	0.026467813	0.065521707
ENSG00000248690	HAS2-AS1	-2.86	0.026467993	0.065521707
ENSG00000123607	TTC21B	-0.44	0.026521639	0.065646386
ENSG00000151465	CDC123	-0.17	0.026542641	0.065684736
ENSG00000124713	GNMT	1.95	0.026543697	0.065684736
ENSG00000168101	NUDT16L1	-0.33	0.026566178	0.06573224
ENSG00000142405	NLRP12	-2.57	0.026574735	0.065745283
ENSG00000101040	ZMYND8	0.25	0.026588443	0.065771066
ENSG00000176732	PFN4	-2.87	0.026595265	0.065779811
ENSG00000186951	PPARA	0.31	0.02665775	0.065926211
ENSG00000213906	LTB4R2	-0.64	0.026693195	0.066005711
ENSG00000102349	KLF8	1.02	0.026746437	0.066129193
ENSG00000197114	ZGPAT	0.36	0.026834122	0.066337795

ENSG00000121851	POLR3GL	-0.32	0.0268605	0.066394803
ENSG00000137764	MAP2K5	-0.40	0.026866792	0.066402154
ENSG00000107815	TWNK	-0.19	0.026893539	0.066460053
ENSG00000257027	AC010186.3	-4.14	0.026915804	0.066506861
ENSG00000204262	COL5A2	-0.78	0.026992679	0.066684105
ENSG00000125249	RAP2A	-0.28	0.0269942	0.066684105
ENSG00000123612	ACVR1C	0.82	0.027012362	0.066715181
ENSG00000182993	C12orf60	-0.86	0.027014139	0.066715181
ENSG00000165072	MAMDC2	1.14	0.027016781	0.066715181
ENSG00000114841	DNAH1	-0.45	0.027040678	0.066765952
ENSG00000135414	GDF11	0.37	0.027055661	0.066788989
ENSG00000233328	PFN1P1	0.92	0.027056683	0.066788989
ENSG00000166261	ZNF202	-0.33	0.027062783	0.066795808
ENSG00000137275	RIPK1	0.26	0.027072776	0.066812232
ENSG00000069696	DRD4	-1.26	0.027091378	0.066849897
ENSG00000078269	SYNJ2	0.26	0.027120793	0.066914229
ENSG00000197586	ENTPD6	-0.18	0.027130038	0.066928787
ENSG00000118514	ALDH8A1	0.84	0.027160213	0.06699497
ENSG00000137869	CYP19A1	1.51	0.027221949	0.067138975
ENSG00000287051	AC121757.2	-2.34	0.027254252	0.067210361
ENSG00000163520	FBLN2	2.82	0.02734123	0.067416547
ENSG00000152380	FAM151B	1.14	0.027349644	0.067428986
ENSG00000147475	ERLIN2	-0.29	0.027356288	0.067437057
ENSG00000215835	AL596087.1	-2.10	0.027363477	0.067446473
ENSG00000224165	DNAJC27-AS1	-1.18	0.027378271	0.067474626
ENSG00000234664	HMGN2P5	-0.22	0.027403656	0.067528872
ENSG00000114491	UMPS	-0.21	0.02743526	0.067593908
ENSG00000149262	INTS4	0.25	0.027438157	0.067593908
ENSG00000155393	HEATR3	-0.33	0.027440181	0.067593908
ENSG00000116983	HPCAL4	0.33	0.027474568	0.067657356
ENSG00000233822	H2BC15	0.98	0.027475857	0.067657356
ENSG00000236814	AC046176.1	1.10	0.027476081	0.067657356
ENSG00000107201	DDX58	0.50	0.027497143	0.067700889
ENSG00000091009	RBM27	0.32	0.027555695	0.067836705
ENSG00000273783	AL136040.1	-1.16	0.027615698	0.067976058
ENSG00000227051	C14orf132	0.63	0.027636184	0.068018117
ENSG00000198841	KTI12	0.49	0.027653222	0.068051681
ENSG00000113555	PCDH12	-0.90	0.027728852	0.06822941
ENSG00000165568	AKR1E2	-0.88	0.027737412	0.068242081
ENSG00000214765	SEPTIN7P2	-0.49	0.027748515	0.068261007
ENSG00000156049	GNA14	3.27	0.027851789	0.068498994
ENSG00000081248	CACNA1S	3.26	0.027852104	0.068498994
ENSG00000197930	ERO1A	-0.17	0.027876986	0.068551761
ENSG00000135677	GNS	0.16	0.027894314	0.068582841
ENSG00000248124	RRN3P1	-0.67	0.02790032	0.068582841
ENSG00000268941	LINC01711	4.85	0.027903333	0.068582841

ENSG00000283057	AC010653.3	4.85	0.027903333	0.068582841
ENSG00000145734	BDP1	0.31	0.027944068	0.068674529
ENSG00000161544	CYGB	-2.91	0.027956834	0.068697466
ENSG00000268942	CKS1BP3	0.86	0.027970073	0.068721559
ENSG00000007237	GAS7	0.48	0.028006653	0.068799721
ENSG00000197959	DNM3	0.62	0.028008761	0.068799721
ENSG00000214022	REPIN1	0.16	0.028022611	0.068819197
ENSG00000231721	LINC-PINT	0.68	0.028024251	0.068819197
ENSG00000131848	ZSCAN5A	0.59	0.028027007	0.068819197
ENSG00000163681	SLMAP	0.22	0.028048517	0.068863566
ENSG00000230316	FEZF1-AS1	0.53	0.028094526	0.068968064
ENSG00000078369	GNB1	-0.13	0.028103529	0.068981702
ENSG00000112541	PDE10A	1.75	0.028106996	0.068981752
ENSG00000168421	RHOH	4.86	0.028130302	0.069013559
ENSG00000255222	SETP17	4.86	0.028130302	0.069013559
ENSG00000285737	LINC02680	4.86	0.028130302	0.069013559
ENSG00000237238	BMS1P10	1.53	0.028164246	0.069088366
ENSG00000171094	ALK	4.85	0.028181369	0.069098544
ENSG00000229930	AC138393.2	4.85	0.028181369	0.069098544
ENSG00000259884	NR4A1AS	4.85	0.028181369	0.069098544
ENSG00000048342	CC2D2A	0.46	0.028182207	0.069098544
ENSG00000125843	AP5S1	0.40	0.028185982	0.069099336
ENSG00000120254	MTHFD1L	-0.15	0.028193083	0.069108276
ENSG00000096717	SIRT1	0.30	0.028234205	0.069200602
ENSG00000135457	TFCP2	-0.22	0.028295233	0.069341685
ENSG00000173320	STOX2	0.52	0.028299569	0.06934382
ENSG00000258682	AL132989.1	-1.54	0.028308436	0.069350962
ENSG00000095794	CREM	0.35	0.028315761	0.069350962
ENSG00000118473	SGIP1	1.43	0.028316798	0.069350962
ENSG00000240449	AC005586.1	1.54	0.028318253	0.069350962
ENSG00000239521	CASTOR3	0.41	0.028321192	0.069350962
ENSG00000119699	TGFB3	0.79	0.028323277	0.069350962
ENSG00000159199	ATP5MC1	0.32	0.028401949	0.069535087
ENSG00000128951	DUT	-0.19	0.028424399	0.069581539
ENSG00000247199	AC011346.1	1.91	0.028435549	0.06960032
ENSG00000131797	CLUHP3	-0.37	0.028443018	0.069604738
ENSG00000261461	UBE2MP1	0.58	0.028444311	0.069604738
ENSG00000116903	EXOC8	0.30	0.02847058	0.069660502
ENSG00000125870	SNRPB2	0.20	0.028510602	0.069749811
ENSG00000101654	RNMT	-0.22	0.028514052	0.069749811
ENSG00000101945	SUV39H1	-0.30	0.028523683	0.069759089
ENSG00000240602	AADACP1	1.14	0.028524816	0.069759089
ENSG00000100461	RBM23	0.18	0.028556907	0.069829035
ENSG00000133805	AMPD3	0.27	0.028591166	0.069904265
ENSG00000164932	CTHRC1	0.51	0.028625489	0.069979633
ENSG00000072121	ZFYVE26	-0.34	0.028635937	0.069996625

ENSG00000150776	NKAPD1	0.29	0.028649114	0.07001894
ENSG00000176641	RNF152	1.29	0.028652064	0.07001894
ENSG00000130590	SAMD10	-0.98	0.028697882	0.070122345
ENSG00000124201	ZNFX1	0.20	0.028709129	0.070141264
ENSG00000102878	HSF4	-0.65	0.028723834	0.070168624
ENSG00000170545	SMAGP	0.28	0.028785186	0.070309917
ENSG00000166454	ATMIN	-0.21	0.028834268	0.070414735
ENSG00000129480	DTD2	-0.63	0.028835137	0.070414735
ENSG00000253741	LNCOC1	-1.59	0.028872476	0.070497315
ENSG00000278311	GGNBP2	0.20	0.028883075	0.07051459
ENSG00000226029	LINC01772	-1.60	0.0289394	0.070643484
ENSG00000173926	MARCHF3	0.82	0.028946209	0.070651219
ENSG00000275835	TUBGCP5	-0.36	0.02894963	0.070651219
ENSG00000233251	AC007743.1	4.86	0.028969372	0.070682161
ENSG00000286089	AC095057.4	4.86	0.028969372	0.070682161
ENSG00000144642	RBMS3	4.85	0.028975839	0.07068932
ENSG00000180616	SSTR2	-2.64	0.028982429	0.070696778
ENSG00000169884	WNT10B	0.31	0.028997421	0.070724727
ENSG00000113790	EHHADH	-0.43	0.029002896	0.070729461
ENSG00000115109	EPB41L5	0.30	0.02900971	0.070737457
ENSG00000146063	TRIM41	-0.17	0.029024907	0.070765892
ENSG00000265452	MIR3682	2.05	0.029072852	0.070874153
ENSG00000273654	AC020904.2	-1.09	0.029092263	0.070912835
ENSG00000109118	PHF12	-0.25	0.029127422	0.070989887
ENSG00000130222	GADD45G	1.48	0.029147651	0.071030539
ENSG00000255893	AP000786.1	-1.42	0.029156994	0.071044657
ENSG00000224032	EPB41L4A-AS1	-0.32	0.029204078	0.07115072
ENSG00000179922	ZNF784	0.58	0.029219796	0.071180347
ENSG00000118804	STBD1	0.69	0.02923237	0.071202311
ENSG00000140332	TLE3	0.24	0.029260142	0.071261283
ENSG00000126581	BECN1	-0.24	0.029302125	0.071354846
ENSG00000152292	SH2D6	-1.27	0.029313216	0.071373169
ENSG00000161010	MRNIP	0.26	0.029355962	0.071468554
ENSG00000259207	ITGB3	-2.43	0.029369548	0.071492931
ENSG00000028116	VRK2	-0.34	0.029409683	0.071581923
ENSG00000225434	LINC01504	0.86	0.029437239	0.071640281
ENSG00000182518	FAM104B	0.42	0.029445402	0.071651434
ENSG00000203727	SAMD5	-0.68	0.029469124	0.071700439
ENSG00000149930	TAOK2	-0.18	0.0294831	0.071725034
ENSG00000133657	ATP13A3	-0.35	0.029486401	0.071725034
ENSG00000091490	SEL1L3	-0.19	0.029528581	0.071818905
ENSG00000130177	CDC16	0.20	0.029576878	0.071927633
ENSG00000046653	GPM6B	-1.38	0.029638885	0.072069668
ENSG00000123178	SPRYD7	0.22	0.029652013	0.07209283
ENSG00000205363	INSYN1	0.62	0.029714312	0.072235521
ENSG00000049249	TNFRSF9	1.23	0.029734975	0.072275528

ENSG00000160336	ZNF761	-0.49	0.029737992	0.072275528
ENSG00000167723	TRPV3	0.77	0.029746576	0.072287611
ENSG00000155636	RBM45	-0.50	0.029821378	0.072460591
ENSG00000180879	SSR4	0.17	0.029855997	0.072535901
ENSG00000087842	PIR	-0.28	0.029860317	0.07253759
ENSG00000126705	AHDC1	0.25	0.029911209	0.072652399
ENSG00000146757	ZNF92	0.39	0.030003102	0.072866757
ENSG00000163617	CCDC191	-0.67	0.030019603	0.072891963
ENSG00000183542	KLRC4	-2.57	0.030020765	0.072891963
ENSG00000183828	NUDT14	-0.34	0.030028484	0.07290186
ENSG00000187790	FANCM	0.51	0.030042914	0.072928044
ENSG00000161960	EIF4A1	-0.37	0.030052114	0.07294153
ENSG00000175106	TVP23C	0.80	0.030089329	0.073022999
ENSG00000168710	AHCYL1	-0.16	0.030131576	0.07311666
ENSG00000265458	AC132938.4	-1.05	0.030182789	0.073232054
ENSG00000125744	RTN2	-0.38	0.03020751	0.073283149
ENSG00000117697	NSL1	-0.29	0.030248128	0.073372792
ENSG00000163170	BOLA3	-0.27	0.030269629	0.073416049
ENSG00000277399	GPR179	-1.58	0.030297657	0.073475123
ENSG00000117143	UAP1	0.22	0.030371396	0.073645024
ENSG00000111877	MCM9	-0.43	0.030378632	0.073646951
ENSG00000272141	AL390719.2	0.91	0.030379551	0.073646951
ENSG00000170037	CNTROB	-0.21	0.030393104	0.07367088
ENSG00000127540	UQCR11	-0.18	0.030412522	0.073709021
ENSG00000260317	AC009812.4	0.63	0.030419363	0.073716675
ENSG00000100056	ESS2	0.26	0.030425268	0.073722057
ENSG00000167232	ZNF91	-0.58	0.030437411	0.073739469
ENSG00000174738	NR1D2	-0.23	0.030439824	0.073739469
ENSG00000224660	SH3BP5-AS1	-0.56	0.030455735	0.073753225
ENSG00000278192	AL118505.1	-2.87	0.030456364	0.073753225
ENSG00000189362	NEMP2	0.39	0.030456559	0.073753225
ENSG00000203780	FANK1	0.91	0.030565078	0.073992842
ENSG00000213430	HSPD1P1	0.49	0.030566412	0.073992842
ENSG00000115234	SNX17	-0.19	0.030566601	0.073992842
ENSG00000186889	TMEM17	-0.57	0.030621563	0.074116922
ENSG00000234840	LINC01239	4.04	0.030662834	0.07420784
ENSG00000170439	METTL7B	0.44	0.030693947	0.074274155
ENSG00000109572	CLCN3	0.35	0.030706444	0.074295413
ENSG00000287407	AL596442.3	0.71	0.030714388	0.074305006
ENSG00000130829	DUSP9	0.33	0.030717835	0.074305006
ENSG00000197748	CFAP43	-0.76	0.030735019	0.074337586
ENSG00000099624	ATP5F1D	-0.19	0.030809466	0.074505356
ENSG00000147570	DNAJC5B	4.04	0.030814446	0.074505356
ENSG00000157350	ST3GAL2	0.26	0.030815552	0.074505356
ENSG00000101955	SRPX	0.29	0.030825334	0.074520003
ENSG00000270728	AL035413.1	-1.60	0.030840778	0.074548332

ENSG00000105792	CFAP69	-1.16	0.030850711	0.074563338
ENSG00000196924	FLNA	-0.13	0.03087634	0.074616269
ENSG00000184319	RPL23AP82	0.34	0.030885204	0.074628676
ENSG00000099984	GSTT2	-0.90	0.030896988	0.074648137
ENSG00000232713	RPS12P3	3.19	0.030909094	0.074659389
ENSG00000284686	AC119674.2	4.03	0.030909107	0.074659389
ENSG00000139323	POC1B	0.35	0.030921422	0.074680122
ENSG00000155545	MIER3	0.28	0.030927367	0.074685467
ENSG00000172590	MRPL52	0.19	0.030944854	0.074718679
ENSG00000072756	TRNT1	-0.42	0.030963216	0.074753997
ENSG00000112367	FIG4	0.30	0.031027897	0.07490112
ENSG00000254615	AC027031.2	-0.48	0.03104444	0.074924617
ENSG00000132003	ZSWIM4	0.64	0.031045119	0.074924617
ENSG00000184058	TBX1	0.95	0.03112933	0.075118793
ENSG00000116266	STXBP3	0.30	0.031136161	0.075124285
ENSG00000155561	NUP205	0.16	0.031139114	0.075124285
ENSG00000113319	RASGRF2	-1.42	0.031216975	0.075303049
ENSG00000146112	PPP1R18	0.20	0.031225729	0.075315088
ENSG00000196335	STK31	4.04	0.031253791	0.075370385
ENSG00000237424	FOXD2-AS1	-0.61	0.031259549	0.075370385
ENSG00000161835	TAMALIN	1.30	0.031259954	0.075370385
ENSG00000027869	SH2D2A	-1.05	0.031272291	0.075391048
ENSG00000188312	CENPP	-0.61	0.031280866	0.075402637
ENSG00000165185	KIAA1958	-0.44	0.031319266	0.075486106
ENSG00000234608	MAPKAPK5-AS	-0.31	0.031329054	0.075500605
ENSG00000280173	AC104447.1	-3.57	0.031375356	0.075603084
ENSG00000168237	GLYCTK	0.65	0.031389769	0.075627138
ENSG00000197467	COL13A1	0.35	0.031392896	0.075627138
ENSG00000170965	PLAC1	1.07	0.031405823	0.075649174
ENSG00000182858	ALG12	-0.35	0.031418041	0.075669496
ENSG00000172818	OVOL1	0.44	0.031438372	0.07570935
ENSG00000162390	ACOT11	-0.34	0.031457619	0.075746587
ENSG00000163755	HPS3	-0.36	0.031477514	0.075785371
ENSG00000148408	CACNA1B	-0.89	0.03149155	0.075810045
ENSG00000214944	ARHGEF28	0.54	0.031496863	0.075813716
ENSG00000234183	LINC01952	-3.23	0.031501031	0.075814628
ENSG00000145860	RNF145	0.13	0.031518514	0.075847583
ENSG00000280184	AL023806.3	1.79	0.031600736	0.076036304
ENSG00000256683	ZNF350	1.02	0.031675719	0.076207562
ENSG00000246203	AL353807.3	-4.04	0.03170622	0.076260923
ENSG00000272502	AC104958.2	-4.04	0.03170622	0.076260923
ENSG00000203706	SERTAD4-AS1	3.21	0.031709331	0.076260923
ENSG00000126453	BCL2L12	0.22	0.031719793	0.07627008
ENSG00000234719	NPIPB2	-2.86	0.031722698	0.07627008
ENSG00000135605	TEC	0.56	0.031724573	0.07627008
ENSG00000142347	MYO1F	-0.92	0.031755744	0.076335849

ENSG00000185022	MAFF	0.22	0.031771207	0.076363848
ENSG00000149809	TM7SF2	-0.29	0.03178775	0.076394434
ENSG00000051180	RAD51	-0.26	0.031799067	0.076412456
ENSG00000279953	AC117503.3	-2.57	0.031869303	0.076565264
ENSG00000234500	AC008267.3	-2.42	0.03187031	0.076565264
ENSG00000254469	AP002495.1	-1.03	0.031882498	0.076585351
ENSG00000240356	RPL23AP7	0.49	0.031908628	0.076630996
ENSG00000132128	LRRC41	0.21	0.031909159	0.076630996
ENSG00000119411	BSPRY	0.24	0.031934416	0.076676645
ENSG00000138138	ATAD1	-0.20	0.031939085	0.076676645
ENSG00000206754	SNORD101	-1.76	0.031942384	0.076676645
ENSG00000186501	TMEM222	0.26	0.031943493	0.076676645
ENSG00000137364	TPMT	0.29	0.031957329	0.076700656
ENSG00000117152	RGS4	4.03	0.032032566	0.076865445
ENSG00000093000	NUP50	-0.17	0.03203367	0.076865445
ENSG00000149489	ROM1	0.99	0.032059787	0.076918891
ENSG00000279880	AC134407.3	2.82	0.03207381	0.07694331
ENSG00000235072	ARNILA	-4.05	0.032172602	0.077171056
ENSG00000070476	ZXDC	0.29	0.032218749	0.077272483
ENSG00000175279	CENPS	-0.52	0.03233521	0.077542508
ENSG00000176244	ACBD7	0.46	0.032341208	0.077547598
ENSG00000229939	AL589880.1	-1.83	0.032349549	0.077558306
ENSG00000211459	MT-RNR1	-0.15	0.032432527	0.077747932
ENSG00000257954	AC125611.2	1.71	0.032470361	0.077829304
ENSG00000160838	LRRC71	1.43	0.032531031	0.077961301
ENSG00000245954	LINC02273	1.20	0.032533221	0.077961301
ENSG00000248544	AC008676.1	1.79	0.032539956	0.077968104
ENSG00000254614	AP003068.2	-0.64	0.032638383	0.078194578
ENSG00000183971	NPW	0.40	0.032646768	0.078205305
ENSG00000185246	PRPF39	-0.25	0.032676559	0.0782673
ENSG00000271335	AL117336.2	-1.05	0.032684721	0.078277483
ENSG00000084072	PPIE	0.17	0.032690593	0.078282177
ENSG00000214013	GANC	0.41	0.032727952	0.078362261
ENSG00000174501	ANKRD36C	-0.94	0.032770077	0.078453737
ENSG00000152193	OBI1	0.32	0.032803977	0.078525502
ENSG00000149925	ALDOA	-0.36	0.032852592	0.07863247
ENSG00000102158	MAGT1	-0.19	0.032901602	0.078740355
ENSG00000076864	RAP1GAP	0.26	0.032947515	0.078840806
ENSG00000170043	TRAPPC1	-0.21	0.033033952	0.079038193
ENSG00000206633	SNORA80B	-4.82	0.033168083	0.079340148
ENSG00000273305	AC009237.15	-4.82	0.033168083	0.079340148
ENSG00000163875	MEAF6	0.26	0.033185845	0.07936419
ENSG00000182685	BRICD5	-0.72	0.033186066	0.07936419
ENSG00000224186	C5orf66	-1.46	0.033201038	0.07939051
ENSG00000111679	PTPN6	-0.26	0.033212651	0.07940879
ENSG00000170748	RBMXL2	2.76	0.033244624	0.079471785

ENSG00000198513	ATL1	0.80	0.033248181	0.079471785
ENSG00000281189	GHET1	-2.38	0.033250912	0.079471785
ENSG00000056277	ZNF280C	-0.43	0.033283225	0.079539516
ENSG00000232573	RPL3P4	-0.40	0.033294918	0.079557957
ENSG00000225489	AL354707.1	-0.67	0.033331951	0.07963694
ENSG00000239467	AC007405.3	-0.75	0.033386122	0.079756844
ENSG00000162772	ATF3	0.21	0.033404521	0.079791273
ENSG00000181625	SLX1B	-1.97	0.033428753	0.079836738
ENSG00000143079	CTTNBP2NL	0.40	0.033431534	0.079836738
ENSG00000109805	NCAPG	0.16	0.033474038	0.079928701
ENSG00000130475	FCHO1	-0.30	0.033487367	0.079950989
ENSG00000204540	PSORS1C1	-0.61	0.033526657	0.080035246
ENSG00000231563	AL139288.1	1.76	0.033531557	0.080037394
ENSG00000118939	UCHL3	0.43	0.033547191	0.080065162
ENSG00000269947	AC135178.6	-1.77	0.033556306	0.080076476
ENSG00000157315	TMED6	-1.56	0.033559934	0.080076476
ENSG00000160783	PMF1	0.32	0.033594824	0.080150168
ENSG00000207340	RNVU1-1	2.76	0.033631553	0.080228232
ENSG00000111885	MAN1A1	0.22	0.03370078	0.080378329
ENSG00000103035	PSMD7	-0.16	0.033702506	0.080378329
ENSG00000131480	AOC2	-0.54	0.033717757	0.080405118
ENSG00000179361	ARID3B	0.37	0.033758282	0.080492165
ENSG00000144827	ABHD10	-0.14	0.033770362	0.080511375
ENSG00000099337	KCNK6	0.24	0.033838768	0.080664851
ENSG00000137992	DBT	-0.31	0.033856687	0.080697953
ENSG00000119862	LGALSL	0.30	0.033895794	0.080781545
ENSG00000225376	TMEM246-AS1	-4.82	0.033922941	0.080826983
ENSG00000234684	SDCBP2-AS1	-0.62	0.033922976	0.080826983
ENSG00000125991	ERGIC3	0.17	0.033926977	0.080826983
ENSG00000272030	AL162258.2	-2.51	0.033988147	0.080963075
ENSG00000138449	SLC40A1	1.81	0.034011395	0.081008811
ENSG00000182307	C8orf33	0.17	0.034017526	0.081013771
ENSG00000284624	AC092902.4	1.40	0.034104861	0.081212099
ENSG00000280213	UCKL1-AS1	2.18	0.034128027	0.081257595
ENSG00000154945	ANKRD40	-0.17	0.034147509	0.081294308
ENSG00000253671	AC027117.1	-1.04	0.034156433	0.08130588
ENSG00000100767	PAPLN	-0.40	0.034166408	0.081319951
ENSG00000133275	CSNK1G2	-0.16	0.03417753	0.081336749
ENSG00000168096	ANKS3	0.34	0.034185069	0.081345018
ENSG00000011021	CLCN6	0.33	0.034287945	0.081580115
ENSG00000236333	TRHDE-AS1	-0.75	0.034300831	0.081601072
ENSG00000157617	C2CD2	-0.26	0.034313721	0.081622035
ENSG00000118496	FBXO30	-0.28	0.034389575	0.081792747
ENSG00000288640	AC005192.1	4.03	0.034405254	0.081820315
ENSG00000100968	NFATC4	0.56	0.034416086	0.081833834
ENSG00000155329	ZCCHC10	0.39	0.034419118	0.081833834

ENSG00000112695	COX7A2	0.16	0.034432504	0.081849682
ENSG00000236810	ELOA-AS1	-0.99	0.034433963	0.081849682
ENSG00000145147	SLIT2	0.68	0.034475923	0.081939687
ENSG00000187193	MT1X	-0.36	0.034523806	0.082034015
ENSG00000063180	CA11	-0.26	0.034523809	0.082034015
ENSG00000141376	BCAS3	-0.29	0.034550266	0.082087133
ENSG00000198466	ZNF587	-0.32	0.034602901	0.082202429
ENSG00000266941	AC024592.1	-4.04	0.03461504	0.082221505
ENSG00000169900	PYDC1	1.76	0.034655879	0.082303967
ENSG00000213079	SCAF8	-0.27	0.034657982	0.082303967
ENSG00000170145	SIK2	-0.23	0.034716914	0.082434134
ENSG00000273472	AC096733.2	2.75	0.034730707	0.082457101
ENSG00000145425	RPS3A	-0.12	0.034770318	0.082536693
ENSG00000138459	SLC35A5	-0.31	0.034772479	0.082536693
ENSG00000257698	GIHCG	0.55	0.034809796	0.082615468
ENSG00000265354	TIMM23	0.17	0.034816301	0.082621109
ENSG00000137801	THBS1	0.34	0.034853683	0.082700012
ENSG00000165887	ANKRD2	-2.06	0.034879481	0.082751415
ENSG00000254876	SUGT1P4-STR	1.13	0.034921976	0.082842413
ENSG00000113070	HBEGF	0.33	0.034951015	0.08290147
ENSG00000142102	PGGHG	-0.28	0.034999331	0.083006236
ENSG00000140262	TCF12	-0.23	0.035045033	0.083104776
ENSG00000136630	HLX	0.99	0.035053355	0.083114662
ENSG00000107959	PITRM1	0.18	0.035090303	0.083192412
ENSG00000240399	AC004801.2	-2.56	0.035140766	0.083302181
ENSG00000175895	PLEKHF2	0.29	0.03516225	0.083343236
ENSG00000170275	CRTAP	0.14	0.035205605	0.083431612
ENSG00000065883	CDK13	0.27	0.035207874	0.083431612
ENSG00000237862	Z94160.1	-2.04	0.035220927	0.083452663
ENSG00000227252	AC012063.1	-1.07	0.035269688	0.083553137
ENSG00000144199	FAHD2B	-0.38	0.035271682	0.083553137
ENSG00000167112	TRUB2	-0.26	0.035280711	0.083564634
ENSG00000275318	AL136981.2	-0.88	0.035333245	0.08367916
ENSG00000198133	TMEM229B	-1.21	0.035359506	0.083731445
ENSG00000188493	C19orf54	-0.32	0.035393151	0.083801199
ENSG00000183091	NEB	0.77	0.035490026	0.084020632
ENSG00000253102	AC004707.1	1.05	0.035502389	0.084039957
ENSG00000229759	MRPS18AP1	-2.09	0.035578543	0.084210266
ENSG00000158815	FGF17	-1.01	0.035600986	0.08425342
ENSG00000178605	GTPBP6	-0.25	0.035657654	0.084377553
ENSG00000144834	TAGLN3	2.00	0.035689314	0.084432504
ENSG00000179168	GGN	2.00	0.035689314	0.084432504
ENSG00000258926	AL355916.2	-4.83	0.035703415	0.084454422
ENSG00000180066	LINC02870	0.69	0.035707019	0.084454422
ENSG00000066827	ZFAT	0.52	0.035716904	0.08446782
ENSG00000197208	SLC22A4	1.41	0.035727884	0.084483801

ENSG00000124593	AL365205.1	0.50	0.035737764	0.084493042
ENSG00000164109	MAD2L1	0.16	0.035740236	0.084493042
ENSG00000115464	USP34	0.22	0.0357587	0.084526707
ENSG00000105700	KXD1	-0.15	0.035782742	0.084573549
ENSG00000111962	UST	0.36	0.03579427	0.084590805
ENSG00000237854	LINC00674	0.46	0.035829645	0.084664407
ENSG00000227615	AP001324.1	-0.50	0.035850708	0.084704176
ENSG00000143632	ACTA1	1.83	0.03586661	0.084731744
ENSG00000173264	GPR137	0.36	0.035894398	0.084787381
ENSG00000163738	MTHFD2L	0.35	0.035976339	0.084970908
ENSG00000101049	SGK2	-1.27	0.036002117	0.085021757
ENSG00000116138	DNAJC16	-0.24	0.036028614	0.085074292
ENSG00000189319	FAM53B	0.24	0.036045993	0.085105288
ENSG00000163728	TTC14	-0.30	0.036058672	0.085125179
ENSG00000002549	LAP3	0.20	0.036079815	0.085165045
ENSG00000101190	TCFL5	-0.33	0.036097141	0.085195895
ENSG00000166866	MYO1A	2.00	0.03613591	0.085277338
ENSG00000246273	SBF2-AS1	0.51	0.036215477	0.085455032
ENSG00000274269	AL031665.1	3.20	0.036254672	0.085537432
ENSG00000268912	AC012313.5	-1.60	0.036269286	0.085561824
ENSG00000115425	PECR	-0.48	0.036371243	0.085792234
ENSG00000119772	DNMT3A	-0.28	0.036388983	0.085809577
ENSG00000072163	LIMS2	-0.91	0.036395341	0.085809577
ENSG00000284874	AC000093.1	2.47	0.036399672	0.085809577
ENSG00000118308	IRAG2	2.27	0.03640254	0.085809577
ENSG00000178752	ERFE	-0.33	0.03640322	0.085809577
ENSG00000111845	PAK1IP1	0.29	0.036404323	0.085809577
ENSG00000117691	NENF	0.20	0.036509922	0.086048352
ENSG00000215817	ZC3H11B	0.60	0.036516295	0.086053238
ENSG00000198754	OXCT2	0.80	0.036528735	0.086072417
ENSG00000275491	LINC01730	-1.09	0.036539599	0.086087881
ENSG00000102572	STK24	-0.15	0.036565804	0.086139479
ENSG00000212864	RNF208	-0.44	0.036609427	0.086231564
ENSG00000253716	MINCR	-0.39	0.036613512	0.086231564
ENSG00000229953	AL590666.2	0.38	0.03663869	0.086280709
ENSG00000151552	QDPR	0.24	0.036648548	0.086293769
ENSG00000127837	AAMP	0.16	0.03674006	0.086499069
ENSG00000224462	C4BPAP1	-1.18	0.036769597	0.086558426
ENSG00000181856	SLC2A4	0.68	0.036794417	0.08659978
ENSG00000214776	AC092821.1	-1.27	0.036795819	0.08659978
ENSG00000132510	KDM6B	-0.24	0.036806826	0.0866155
ENSG00000128309	MPST	0.20	0.036827494	0.086653945
ENSG00000251595	ABCA11P	-1.18	0.036843364	0.086681097
ENSG00000091656	ZFHX4	-0.32	0.036872631	0.086739754
ENSG00000100485	SOS2	-0.36	0.036895404	0.086783124
ENSG00000197713	RPE	0.23	0.036957362	0.086918642

ENSG00000106546	AHR	-0.36	0.037004333	0.087018885
ENSG00000244754	N4BP2L2	-0.20	0.037013064	0.087029189
ENSG00000173928	SWSAP1	0.70	0.037028182	0.087054508
ENSG00000059122	FLYWCH1	-0.18	0.03708429	0.08717618
ENSG00000234805	AC090505.1	1.75	0.037117121	0.087235805
ENSG00000226578	AL132657.1	3.96	0.037118373	0.087235805
ENSG00000274020	LINC01138	0.48	0.037138173	0.087271604
ENSG00000121310	ECHDC2	-0.26	0.037142327	0.087271604
ENSG00000169087	HSPBAP1	0.48	0.037180472	0.087350976
ENSG00000217733	CCT7P1	4.83	0.037193236	0.087370706
ENSG00000279161	AC093503.2	2.31	0.037198124	0.087371931
ENSG00000171763	SPATA5L1	-0.37	0.037220177	0.087413471
ENSG00000130176	CNN1	3.95	0.037265386	0.087509377
ENSG00000031003	FAM13B	0.31	0.037298202	0.087576162
ENSG00000122390	NAA60	0.24	0.037350235	0.087678445
ENSG00000232363	AL021391.1	-3.22	0.037350527	0.087678445
ENSG00000236360	RRAS2P1	-1.47	0.037361516	0.08769265
ENSG00000105767	CADM4	0.21	0.037365342	0.08769265
ENSG00000131095	GFAP	1.70	0.037397825	0.087758592
ENSG00000150459	SAP18	0.14	0.037408457	0.087773248
ENSG00000279766	AC067931.1	3.96	0.037424285	0.087800094
ENSG00000241112	RPL29P14	-2.31	0.0374304	0.087804146
ENSG00000178700	DHFR2	-0.75	0.03744566	0.08782923
ENSG00000139405	RITA1	-0.27	0.037449871	0.08782923
ENSG00000146242	TPBG	-0.25	0.037495798	0.087926635
ENSG00000243978	RTL9	3.95	0.03750136	0.087929375
ENSG00000181938	GINS3	-0.31	0.037506319	0.0879307
ENSG00000107758	PPP3CB	0.22	0.037549547	0.088021733
ENSG00000186566	GPATCH8	-0.22	0.037554271	0.088022497
ENSG00000072422	RHOBTB1	0.65	0.037569645	0.088048218
ENSG00000119912	IDE	-0.22	0.037596991	0.08810199
ENSG00000281332	LINC00997	-0.79	0.037604798	0.088109966
ENSG00000181449	SOX2	3.94	0.037621936	0.088139801
ENSG00000131711	MAP1B	0.22	0.037629978	0.088148322
ENSG00000105550	FGF21	-1.43	0.037702577	0.088308049
ENSG00000100478	AP4S1	-0.68	0.037708564	0.088310946
ENSG00000180264	ADGRD2	1.06	0.03771264	0.088310946
ENSG00000125247	TMTC4	-0.33	0.037725323	0.08833031
ENSG00000243902	ELFN2	-1.20	0.037730088	0.088331133
ENSG00000197121	PGAP1	-0.46	0.037738519	0.088340537
ENSG00000198961	PJA2	-0.22	0.037770496	0.088405049
ENSG00000261655	AC100803.3	0.95	0.037860705	0.088605826
ENSG00000164879	CA3	1.49	0.037920097	0.088734445
ENSG00000146648	EGFR	-0.18	0.037949729	0.088793403
ENSG00000113504	SLC12A7	-0.22	0.037979245	0.088852075
ENSG00000102053	ZC3H12B	-0.93	0.037994019	0.088876248

ENSG00000174456	C12orf76	-0.47	0.03800969	0.088902515
ENSG00000112419	PHACTR2	-0.30	0.03808456	0.089067222
ENSG00000155265	GOLGA7B	0.59	0.038110574	0.089117645
ENSG00000279716	AC006128.1	-1.06	0.038119793	0.089128788
ENSG00000123119	NECAB1	1.82	0.03813176	0.089146353
ENSG00000100726	TELO2	-0.22	0.038179892	0.089248452
ENSG00000223403	MEG9	3.95	0.038199475	0.089273373
ENSG00000285091	AL109840.2	3.95	0.038199475	0.089273373
ENSG00000275223	AL121906.2	-1.83	0.038212856	0.089294217
ENSG00000213995	NAXD	-0.22	0.038269709	0.089416628
ENSG00000284719	AL033527.3	-1.46	0.038278975	0.089427837
ENSG00000174705	SH3PXD2B	-0.21	0.038292822	0.089441769
ENSG00000196369	SRGAP2B	0.48	0.038293878	0.089441769
ENSG00000163794	UCN	-1.05	0.038304804	0.089456848
ENSG00000004139	SARM1	-0.96	0.038315665	0.089471771
ENSG00000209482	SNORD83A	-2.06	0.038331509	0.089498326
ENSG00000077044	DGKD	0.18	0.038347912	0.08952618
ENSG00000111215	PRR4	-1.31	0.038358677	0.089540867
ENSG00000278703	AC100847.1	-3.95	0.038442095	0.089725122
ENSG00000047365	ARAP2	-0.45	0.038451045	0.08972782
ENSG00000185607	ACTBP7	1.42	0.038452218	0.08972782
ENSG00000272885	AC092574.1	-3.96	0.038486762	0.089790551
ENSG00000114654	EFCC1	-0.61	0.038488075	0.089790551
ENSG00000072858	SIDT1	3.12	0.038528823	0.089875138
ENSG00000140543	DET1	-1.56	0.038537476	0.089884844
ENSG00000230061	TRPM2-AS	0.96	0.038557366	0.089920756
ENSG00000138735	PDE5A	-0.66	0.038589617	0.089985483
ENSG00000267681	AC135721.1	-3.95	0.038616645	0.090038016
ENSG00000141452	RMC1	0.22	0.038625128	0.090047304
ENSG00000168118	RAB4A	0.20	0.038631605	0.090051913
ENSG00000244187	TMEM141	-0.22	0.038712387	0.090229709
ENSG00000137500	CCDC90B	0.32	0.038747446	0.090300906
ENSG00000144580	CNOT9	-0.16	0.038801504	0.09041636
ENSG00000141425	RPRD1A	-0.19	0.03882013	0.090449232
ENSG00000132294	EFR3A	-0.17	0.038849557	0.090507257
ENSG00000166823	MESP1	0.39	0.038880071	0.090567801
ENSG00000249485	RBBP4P1	-0.85	0.038906419	0.090618628
ENSG00000023892	DEF6	-0.23	0.038922528	0.090645598
ENSG00000164199	ADGRV1	0.51	0.038972087	0.090750455
ENSG00000211454	AKR7L	-1.77	0.039075193	0.090979961
ENSG00000171714	ANO5	-0.89	0.039080468	0.090981656
ENSG00000198818	SFT2D1	-0.20	0.039087353	0.090987101
ENSG00000124198	ARFGEF2	-0.24	0.039099706	0.091005271
ENSG00000096384	HSP90AB1	-0.15	0.039128322	0.091061283
ENSG00000167971	CASKIN1	0.50	0.039134553	0.091065195
ENSG00000189269	DRICH1	-2.00	0.03914669	0.091082845

ENSG00000115761	NOL10	0.24	0.039158789	0.091100405
ENSG00000135077	HAVCR2	1.94	0.039169214	0.091110793
ENSG00000276571	AC002550.2	-3.95	0.03917236	0.091110793
ENSG00000180423	HARBI1	0.71	0.039193143	0.091148539
ENSG00000140406	TLNRD1	0.23	0.039210566	0.091178461
ENSG00000146670	CDCA5	-0.16	0.03926687	0.091294904
ENSG00000196754	S100A2	0.76	0.039269765	0.091294904
ENSG00000181619	GPR135	0.68	0.039313767	0.091356351
ENSG00000253251	SHLD3	-1.32	0.039315416	0.091356351
ENSG00000091129	NRCAM	4.72	0.039323586	0.091356351
ENSG00000226755	VPS25P1	4.72	0.039323586	0.091356351
ENSG00000257474	AC027288.1	4.72	0.039323586	0.091356351
ENSG00000272908	AC006033.2	4.72	0.039323586	0.091356351
ENSG00000220201	ZGLP1	-1.18	0.039375507	0.091426963
ENSG00000225535	LINC01393	4.71	0.039376385	0.091426963
ENSG00000239473	RPL7P38	4.71	0.039376385	0.091426963
ENSG00000287856	AL445524.2	4.71	0.039376385	0.091426963
ENSG00000100124	ANKRD54	0.24	0.039378511	0.091426963
ENSG00000128652	HOXD3	1.47	0.039381392	0.091426963
ENSG00000106263	EIF3B	0.12	0.039409747	0.091482179
ENSG00000243635	AC091804.1	1.94	0.039526641	0.091742884
ENSG00000198919	DZIP3	0.28	0.039534099	0.091749552
ENSG00000196998	WDR45	0.26	0.039596468	0.091883641
ENSG00000157181	ODR4	0.32	0.039618121	0.091918312
ENSG00000133111	RFXAP	-0.64	0.039620596	0.091918312
ENSG00000164032	H2AZ1	-0.13	0.03965551	0.091980912
ENSG00000206337	HCP5	-0.60	0.039656771	0.091980912
ENSG00000208028	MIR616	-1.45	0.039673955	0.092010105
ENSG00000214960	CRPPA	-1.32	0.039694425	0.092046598
ENSG00000112078	KCTD20	0.19	0.03969889	0.092046598
ENSG00000173141	MRPL57	-0.20	0.039711648	0.092065511
ENSG00000157303	SUSD3	0.60	0.039728809	0.092094627
ENSG00000111859	NEDD9	3.11	0.039735768	0.092097326
ENSG00000149639	SOGA1	0.22	0.039739177	0.092097326
ENSG00000147036	LANCL3	0.78	0.039766958	0.092151039
ENSG00000143630	HCN3	-0.41	0.039796097	0.092207885
ENSG00000235888	AF064858.1	1.25	0.039840803	0.092300781
ENSG00000157933	SKI	0.20	0.039850804	0.092313263
ENSG00000126001	CEP250	-0.23	0.039882966	0.092377074
ENSG00000134444	RELCH	0.29	0.039931212	0.092478119
ENSG00000169282	KCNAB1	-1.95	0.039950143	0.092511254
ENSG00000161643	SIGLEC16	-3.95	0.03998011	0.092569936
ENSG00000204435	CSNK2B	0.22	0.040013874	0.092637395
ENSG00000163684	RPP14	0.32	0.040040858	0.092689145
ENSG00000155592	ZKSCAN2	0.29	0.040050524	0.092700798
ENSG00000136932	TRMO	0.46	0.040070204	0.09270825

ENSG00000144460	NYAP2	4.72	0.040072275	0.09270825
ENSG00000170214	ADRA1B	4.72	0.040072275	0.09270825
ENSG00000257252	AC124947.1	4.72	0.040072275	0.09270825
ENSG00000099834	CDHR5	-2.72	0.040101338	0.092756121
ENSG00000005302	MSL3	0.32	0.040102237	0.092756121
ENSG00000242086	MUC20-OT1	-0.30	0.040118672	0.092783412
ENSG00000136051	WASHC4	-0.30	0.040139741	0.092811085
ENSG00000163746	PLSCR2	4.71	0.040147892	0.092811085
ENSG00000255240	AP001636.3	4.71	0.040147892	0.092811085
ENSG00000117174	ZNHIT6	0.27	0.040149188	0.092811085
ENSG00000110723	EXPH5	0.34	0.040169178	0.092846568
ENSG00000184677	ZBTB40	-0.21	0.040247068	0.09301477
ENSG00000136271	DDX56	0.18	0.040251245	0.09301477
ENSG00000171735	CAMTA1	0.22	0.040275308	0.09305963
ENSG00000196712	NF1	0.27	0.040353372	0.093229241
ENSG00000136643	RPS6KC1	-0.32	0.040367475	0.093251057
ENSG00000101888	NXT2	0.34	0.040427772	0.093379568
ENSG00000185278	ZBTB37	-0.29	0.040436127	0.093381955
ENSG00000179715	PCED1B	1.24	0.040438138	0.093381955
ENSG00000144452	ABCA12	-1.32	0.04045767	0.093408294
ENSG00000232987	LINC01219	-2.71	0.040461727	0.093408294
ENSG00000137819	PAQR5	-0.25	0.040463547	0.093408294
ENSG00000165555	NOXRED1	-2.44	0.040483017	0.093442462
ENSG00000179841	AKAP5	-1.50	0.040510145	0.093494294
ENSG00000254682	AP002387.1	-0.45	0.040536438	0.093544189
ENSG00000172167	MTBP	-0.34	0.040555388	0.093577127
ENSG00000261488	TBILA	-1.32	0.040563744	0.093585618
ENSG00000168014	C2CD3	-0.24	0.040575427	0.093593778
ENSG00000183077	AFMID	-0.24	0.040580406	0.093593778
ENSG00000230896	AL604028.1	-1.25	0.040581312	0.093593778
ENSG00000204084	INPP5B	0.26	0.040592635	0.093609104
ENSG00000107404	DVL1	-0.14	0.040635963	0.093698225
ENSG00000141527	CARD14	-1.68	0.0406484	0.093716104
ENSG00000213047	DENND1B	0.41	0.040674058	0.093764456
ENSG00000115419	GLS	0.17	0.040727631	0.093877143
ENSG00000269091	AC010624.3	2.32	0.040752753	0.09392423
ENSG00000172318	B3GALT1	2.46	0.040831936	0.094090281
ENSG00000176087	SLC35A4	-0.20	0.040834204	0.094090281
ENSG00000181458	TMEM45A	0.41	0.040869487	0.094160739
ENSG00000246985	SOCS2-AS1	0.92	0.040929628	0.094288445
ENSG00000162869	PPP1R21	-0.39	0.040971426	0.09437387
ENSG00000127884	ECHS1	0.19	0.040984628	0.094393414
ENSG00000120471	TP53AIP1	3.94	0.040990605	0.094394456
ENSG00000125818	PSMF1	-0.16	0.040994514	0.094394456
ENSG00000153093	ACOXL	0.62	0.04101208	0.094424039
ENSG00000122870	BICC1	2.46	0.04102177	0.094435484

ENSG00000225697	SLC26A6	-0.24	0.041027897	0.094438726
ENSG00000166922	SCG5	0.95	0.041033429	0.094440595
ENSG00000139428	MMAB	-0.26	0.041056362	0.094482511
ENSG00000197296	FITM2	-0.37	0.041119351	0.094616586
ENSG00000128245	YWHAH	-0.14	0.041126077	0.094621183
ENSG00000223653	AL078459.1	3.13	0.041166181	0.094700324
ENSG00000266028	SRGAP2	-0.21	0.041169939	0.094700324
ENSG00000203644	AC083799.1	0.67	0.041237449	0.094844711
ENSG00000181472	ZBTB2	-0.39	0.041281915	0.094936071
ENSG00000234199	LINC01191	2.69	0.041311339	0.09499282
ENSG00000174672	BRSK2	-0.36	0.041421468	0.095235112
ENSG00000269982	AC018809.2	-1.59	0.041433308	0.095251392
ENSG00000178636	AC092656.1	-3.95	0.041446246	0.09527019
ENSG00000197746	PSAP	0.12	0.041479297	0.095327381
ENSG00000127743	IL17B	1.11	0.041480653	0.095327381
ENSG00000134317	GRHL1	0.45	0.041592792	0.095574114
ENSG00000114646	CSPG5	-0.58	0.041600003	0.095579709
ENSG00000188305	PEAK3	-3.94	0.041625658	0.095627675
ENSG00000172006	ZNF554	-0.65	0.041726217	0.095830506
ENSG00000159733	ZFYVE28	-0.32	0.041741359	0.095830506
ENSG00000168538	TRAPPC11	-0.35	0.04174478	0.095830506
ENSG00000188959	C9orf152	4.71	0.041752258	0.095830506
ENSG00000197273	GUCA2A	4.71	0.041752258	0.095830506
ENSG00000205500	MAPRE3-AS1	4.71	0.041752258	0.095830506
ENSG00000234065	MTND4P26	4.71	0.041752258	0.095830506
ENSG00000276289	KCNE1B	4.71	0.041752258	0.095830506
ENSG00000198763	MT-ND2	0.19	0.041765776	0.095850539
ENSG00000104823	ECH1	-0.22	0.041822221	0.095969073
ENSG00000158470	B4GALT5	-0.12	0.041916223	0.09617375
ENSG00000253825	AC097173.2	4.73	0.041937529	0.096211606
ENSG00000064933	PMS1	-0.27	0.041947926	0.096224426
ENSG00000197535	MYO5A	-0.27	0.041963015	0.09624184
ENSG00000185567	AHNAK2	0.17	0.041965136	0.09624184
ENSG00000163682	RPL9	0.13	0.041984985	0.096276329
ENSG00000162607	USP1	-0.19	0.042146943	0.096636643
ENSG00000288271	AL136981.4	-0.96	0.042189438	0.096722995
ENSG00000244723	ASLP1	-4.80	0.04222178	0.096774969
ENSG00000268230	AC012313.3	-4.80	0.04222178	0.096774969
ENSG00000267419	ZNF56	1.58	0.042234326	0.096792639
ENSG00000091844	RGS17	0.47	0.042241165	0.096797228
ENSG00000152749	GPR180	-0.30	0.042281932	0.096879553
ENSG00000176142	TMEM39A	-0.25	0.042303917	0.09691883
ENSG00000273448	AC006480.2	1.78	0.042343751	0.096995667
ENSG00000131097	HIGD1B	-2.70	0.042347149	0.096995667
ENSG00000163686	ABHD6	0.33	0.042365769	0.097027212
ENSG00000131437	KIF3A	0.30	0.042378347	0.097036861

ENSG00000241837	ATP5PO	-0.20	0.042379681	0.097036861
ENSG00000099783	HNRNPM	0.13	0.042408486	0.097087553
ENSG00000172216	CEBPB	-0.15	0.042411523	0.097087553
ENSG00000108387	SEPTIN4	0.44	0.042469165	0.097208387
ENSG00000245848	CEBPA	-0.43	0.042481422	0.097225322
ENSG00000127616	SMARCA4	-0.15	0.042514827	0.097290648
ENSG00000272305	AC096887.1	-2.45	0.042524731	0.097302187
ENSG00000286261	AC022137.3	1.06	0.042552725	0.097355108
ENSG00000126858	RHOT1	0.23	0.042598673	0.097449091
ENSG00000175793	SFN	-0.16	0.042711785	0.097696679
ENSG00000279672	AP006621.5	-0.83	0.042780735	0.09783317
ENSG00000163703	CRELD1	-0.65	0.042781235	0.09783317
ENSG00000114631	PODXL2	1.15	0.042927788	0.098157094
ENSG00000158636	EMSY	-0.30	0.042944954	0.098182326
ENSG00000170745	KCNS3	0.49	0.042948635	0.098182326
ENSG00000285722	AC207130.1	0.54	0.04297652	0.09823485
ENSG00000258644	SYNJ2BP-COX	2.47	0.043008338	0.098296353
ENSG00000237115	AL139805.1	2.22	0.04310418	0.098492903
ENSG00000271871	AC005740.4	2.22	0.04310418	0.098492903
ENSG00000261762	AC027228.2	-0.78	0.043110285	0.098495481
ENSG00000198003	CCDC151	1.09	0.043115152	0.098495481
ENSG00000008735	MAPK8IP2	0.31	0.043193402	0.098662979
ENSG00000272829	AC002470.1	-2.45	0.043257642	0.09879844
ENSG00000234494	SP2-AS1	-1.19	0.04326431	0.098802393
ENSG00000115596	WNT6	1.65	0.043301716	0.098876535
ENSG00000176658	MYO1D	-0.21	0.043310736	0.098885848
ENSG00000248671	ALG1L9P	-1.15	0.043375859	0.099015755
ENSG00000269858	EGLN2	0.34	0.04337753	0.099015755
ENSG00000053900	ANAPC4	-0.26	0.043385706	0.099023124
ENSG00000242325	RPS12P31	2.68	0.043408807	0.099053256
ENSG00000273812	BX640514.2	2.68	0.043408807	0.099053256
ENSG00000260698	AL591848.3	1.78	0.043477897	0.099189174
ENSG00000133519	ZDHHC8P1	-0.96	0.043478285	0.099189174
ENSG00000167754	KLK5	-3.20	0.043492818	0.099204259
ENSG00000229036	VDAC1P8	-0.53	0.043494812	0.099204259
ENSG00000136710	CCDC115	0.28	0.043529431	0.099271907
ENSG00000254978	ALG1L9P	-1.99	0.043566133	0.099344285
ENSG00000170604	IRF2BP1	0.21	0.043599243	0.09940846
ENSG00000082458	DLG3	-0.18	0.043614687	0.099432343
ENSG00000247809	NR2F2-AS1	0.79	0.043628771	0.099453122
ENSG00000114023	FAM162A	-0.25	0.043693836	0.099590097
ENSG00000275557	AC242842.1	1.11	0.043707074	0.099608925
ENSG00000229692	SOS1-IT1	1.17	0.043749202	0.099693581
ENSG00000100628	ASB2	3.12	0.043789161	0.099773277
ENSG00000185730	ZNF696	-0.27	0.043798348	0.099782848
ENSG00000068028	RASSF1	0.23	0.04385382	0.099897854

ENSG00000277972	CISD3	-0.23	0.04391236	0.100019821
ENSG00000258317	AC034102.6	2.26	0.043920958	0.10002802
ENSG00000267904	AC024075.1	-1.45	0.043934631	0.100047774
ENSG00000198604	BAZ1A	0.24	0.043943004	0.100055456
ENSG00000125966	MMP24	-2.70	0.043974017	0.10011468
ENSG00000205771	CATSPER2P1	-1.27	0.04402205	0.100212633
ENSG00000167281	RBFOX3	-1.16	0.044037987	0.100237512
ENSG00000118785	SPP1	0.86	0.04404946	0.100252222
ENSG00000102543	CDADC1	0.42	0.044136351	0.100438555
ENSG00000284773	AC114490.3	-2.33	0.044175232	0.100515605
ENSG00000143382	ADAMTSL4	-0.79	0.044218738	0.100603159
ENSG00000138376	BARD1	-0.29	0.044261801	0.100689685
ENSG00000123009	NME2P1	-1.52	0.044280342	0.100711416
ENSG00000260404	AC110079.1	-0.42	0.04428635	0.100711416
ENSG00000160714	UBE2Q1	0.16	0.044286451	0.100711416
ENSG00000163666	HESX1	-0.93	0.044306736	0.100746096
ENSG00000184056	VPS33B	-0.52	0.044319395	0.100763433
ENSG00000215529	EFCAB8	2.46	0.044331529	0.10077957
ENSG00000089041	P2RX7	1.25	0.044350953	0.100807916
ENSG00000144320	LNPK	-0.25	0.044355716	0.100807916
ENSG00000185219	ZNF445	-0.26	0.04435911	0.100807916
ENSG00000273179	AC092535.5	-3.15	0.0443809	0.100845984
ENSG00000240038	AMY2B	-1.73	0.044408892	0.100898133
ENSG00000234072	AC074117.1	0.35	0.044456224	0.100994206
ENSG00000236305	SLC12A9-AS1	-1.79	0.044472624	0.101019995
ENSG00000101181	MTG2	-0.22	0.04453963	0.101160717
ENSG00000239542	RN7SL399P	4.70	0.044554358	0.101182684
ENSG00000104312	RIPK2	-0.24	0.044602852	0.10128132
ENSG00000173480	ZNF417	-0.60	0.044673641	0.101430554
ENSG00000188167	TMPPE	-0.96	0.044744946	0.101580926
ENSG00000176473	WDR25	0.43	0.044784887	0.101660067
ENSG00000162620	LRRIQ3	-1.39	0.044810266	0.101706141
ENSG00000142871	CCN1	-0.25	0.044839054	0.10175994
ENSG00000123144	TRIR	-0.15	0.044844328	0.101760368
ENSG00000165792	METTL17	0.19	0.044867681	0.101801818
ENSG00000275038	AC091980.2	2.41	0.044905185	0.101875361
ENSG00000184923	NUTM2A	-2.43	0.044923173	0.101898053
ENSG00000183955	KMT5A	0.17	0.044927122	0.101898053
ENSG00000015520	NPC1L1	1.95	0.044930966	0.101898053
ENSG00000246090	AP002026.1	-0.98	0.044935554	0.101898053
ENSG00000230615	AL139220.2	-1.32	0.045100109	0.102255193
ENSG00000214654	B3GNT10	-0.51	0.045103268	0.102255193
ENSG00000156136	DCK	0.21	0.045111865	0.102263101
ENSG00000037897	METTL1	0.22	0.045198472	0.102447822
ENSG00000163273	NPPC	2.07	0.045271617	0.102601992
ENSG00000238197	PAXBP1-AS1	-0.80	0.045293302	0.102639516

ENSG00000287670	AC108479.1	0.84	0.045310151	0.102663573
ENSG00000270179	AP002840.2	-2.44	0.045314179	0.102663573
ENSG00000114107	CEP70	0.24	0.045320207	0.102665609
ENSG00000005108	THSD7A	3.86	0.045329511	0.102675063
ENSG00000219626	FAM228B	-1.25	0.045339499	0.102686062
ENSG00000253320	AZIN1-AS1	-0.92	0.045350231	0.102695925
ENSG00000213901	SLC23A3	-0.93	0.045354117	0.102695925
ENSG00000273387	AC005005.3	-1.33	0.045449656	0.102900614
ENSG00000149582	TMEM25	0.31	0.04545799	0.102907839
ENSG00000167528	ZNF641	0.26	0.045581248	0.103175199
ENSG00000225932	CTAGE4	-0.90	0.045617168	0.10323401
ENSG00000262712	AC012676.1	-0.93	0.045617547	0.10323401
ENSG00000164244	PRRC1	0.19	0.045626742	0.103243144
ENSG00000243742	RPLP0P2	0.81	0.045751709	0.103514211
ENSG00000270021	AC026691.1	-2.71	0.045806558	0.103626592
ENSG00000197386	HTT	-0.19	0.04583759	0.103685074
ENSG00000146373	RNF217	-0.29	0.04589996	0.103810162
ENSG00000099308	MAST3	0.29	0.045903264	0.103810162
ENSG00000166747	AP1G1	0.15	0.045926046	0.103849946
ENSG00000167178	ISLR2	2.06	0.045951425	0.103895595
ENSG00000134780	DAGLA	-0.55	0.045981652	0.103952193
ENSG00000171159	C9orf16	-0.21	0.046012974	0.104011254
ENSG00000080573	COL5A3	1.41	0.046065181	0.104117507
ENSG00000159885	ZNF222	0.73	0.046106623	0.104199406
ENSG00000196092	PAX5	3.85	0.046145209	0.104274833
ENSG00000103549	RNF40	-0.16	0.046199146	0.104384929
ENSG00000134363	FST	1.58	0.046283995	0.104564835
ENSG00000104946	TBC1D17	0.25	0.046329224	0.104655201
ENSG00000228794	LINC01128	-0.30	0.046404587	0.104802133
ENSG00000090857	PDPR	-0.19	0.046404742	0.104802133
ENSG00000019549	SNAI2	1.62	0.046424908	0.104832431
ENSG00000240891	PLCXD2	0.30	0.046428635	0.104832431
ENSG00000116473	RAP1A	0.21	0.046442627	0.104852194
ENSG00000284968	AC093827.4	1.53	0.046468821	0.104899497
ENSG00000080493	SLC4A4	3.12	0.046520281	0.105003819
ENSG00000077152	UBE2T	0.16	0.046590073	0.105149493
ENSG00000256393	RPL41P5	-0.83	0.046603578	0.105168111
ENSG00000270392	PFN1P2	-2.01	0.046622584	0.105199139
ENSG00000100036	SLC35E4	0.42	0.046675804	0.105307349
ENSG00000162669	HFM1	-3.14	0.046860436	0.105711987
ENSG00000123685	BATF3	0.65	0.046892095	0.105771483
ENSG00000226465	AL390198.1	2.70	0.046956157	0.105904049
ENSG00000271452	AC005034.5	-1.95	0.046972911	0.105929896
ENSG00000286061	RCAN3AS	3.04	0.046989772	0.105946379
ENSG00000177303	CASKIN2	-0.22	0.046990809	0.105946379
ENSG00000225806	AL121917.1	-2.18	0.047000406	0.10595608

ENSG00000030110	BAK1	0.23	0.047006586	0.105958077
ENSG00000248424	OR51K1P	-2.10	0.047038912	0.106015016
ENSG00000235314	LINC00957	1.62	0.047042442	0.106015016
ENSG00000168002	POLR2G	-0.19	0.047069002	0.106062928
ENSG00000246596	AC139795.1	-1.71	0.047084557	0.106086033
ENSG00000185761	ADAMTSL5	-0.34	0.047150574	0.106222817
ENSG00000147687	TATDN1	0.22	0.047172626	0.106260536
ENSG00000130726	TRIM28	0.14	0.047180402	0.106266089
ENSG00000162105	SHANK2	0.37	0.047194538	0.106285967
ENSG00000100109	TFIP11	0.21	0.047262473	0.106426985
ENSG00000226383	LINC01876	-1.18	0.047284914	0.106465538
ENSG00000188352	FOCAD	0.20	0.047316377	0.106523503
ENSG00000125384	PTGER2	1.04	0.047321304	0.106523503
ENSG00000167136	ENDOG	-0.37	0.047346155	0.106567456
ENSG00000136383	ALPK3	-1.05	0.047356174	0.106578021
ENSG00000126602	TRAP1	-0.12	0.047459424	0.10679838
ENSG00000099956	SMARCB1	0.16	0.047501424	0.106880875
ENSG00000257335	MGAM	-1.95	0.047511707	0.106891994
ENSG00000183150	GPR19	-1.09	0.047547893	0.10696138
ENSG00000016082	ISL1	0.87	0.047583529	0.107029513
ENSG00000006042	TMEM98	-0.24	0.047611865	0.107070649
ENSG00000233232	NPIPB7	-4.66	0.047612518	0.107070649
ENSG00000137642	SORL1	-0.20	0.047688475	0.107229411
ENSG00000284747	AL034417.4	0.89	0.047700857	0.107235053
ENSG00000184908	CLCNKB	1.10	0.047701701	0.107235053
ENSG00000275106	AC025594.2	-3.05	0.047910412	0.107692146
ENSG00000109854	HTATIP2	0.18	0.047928077	0.107719753
ENSG00000256980	KHDC1L	1.00	0.047945257	0.107746264
ENSG00000093010	COMT	-0.21	0.047959829	0.107754973
ENSG00000107957	SH3PXD2A	0.26	0.047959901	0.107754973
ENSG00000228933	AC107419.1	1.10	0.04804351	0.107908577
ENSG00000185352	HS6ST3	2.68	0.048051343	0.107908577
ENSG00000004866	ST7	0.34	0.04805406	0.107908577
ENSG00000200714	Y_RNA	-4.66	0.048055229	0.107908577
ENSG00000265094	AC007922.2	-4.66	0.048055229	0.107908577
ENSG00000158292	GPR153	-0.47	0.048079993	0.107952072
ENSG00000156411	ATP5MPL	0.17	0.048126943	0.108045365
ENSG00000234705	HMGA1P4	-1.63	0.048139638	0.108061742
ENSG00000270587	AC046185.2	-1.56	0.048153946	0.108065576
ENSG00000260219	CD2BP2-DT	0.83	0.048161656	0.108065576
ENSG00000236045	AL031283.2	-4.67	0.048168346	0.108065576
ENSG00000250740	AC109361.2	-4.67	0.048168346	0.108065576
ENSG00000279206	AC004943.3	-4.67	0.048168346	0.108065576
ENSG00000119943	PYROXD2	-0.45	0.048194368	0.108111835
ENSG00000163131	CTSS	1.49	0.048320826	0.1083772
ENSG00000180098	TRNAU1AP	-0.25	0.048328208	0.1083772

ENSG00000143337	TOR1AIP1	0.23	0.048328909	0.1083772
ENSG00000139438	FAM222A	0.45	0.048351379	0.10841544
ENSG00000120159	CAAP1	0.27	0.048388761	0.108487103
ENSG00000128872	TMOD2	-0.37	0.048443227	0.10859705
ENSG00000274290	H2BC6	1.47	0.048504733	0.108722749
ENSG00000266777	SH3GL1P1	1.38	0.048554452	0.108822007
ENSG00000125434	SLC25A35	0.42	0.048588817	0.108886831
ENSG00000144840	RABL3	0.29	0.048641825	0.10898567
ENSG00000080345	RIF1	-0.21	0.048643814	0.10898567
ENSG00000123094	RASSF8	0.36	0.04866102	0.109012014
ENSG00000107341	UBE2R2	-0.16	0.04868785	0.109059913
ENSG00000239305	RNF103	0.28	0.048706955	0.109090497
ENSG00000206113	CFAP99	1.95	0.048775818	0.109232505
ENSG00000151461	UPF2	0.22	0.048803249	0.109281707
ENSG00000267078	AC015802.1	-3.85	0.048823096	0.109313918
ENSG00000205809	KLRC2	-0.62	0.048864527	0.109394442
ENSG00000171202	TMEM126A	-0.43	0.048877619	0.109411511
ENSG00000143457	GOLPH3L	0.26	0.048884329	0.109414294
ENSG00000129566	TEP1	0.23	0.048892206	0.109419685
ENSG00000285825	CCDC15-DT	-0.94	0.048913307	0.109454668
ENSG00000287923	AL138701.2	1.36	0.048979916	0.109591466
ENSG00000288534	AP001931.2	0.75	0.049049585	0.109735081
ENSG00000077721	UBE2A	-0.19	0.049146759	0.109940191
ENSG00000101846	STS	1.17	0.04922427	0.110101272
ENSG00000158122	PRXL2C	-0.29	0.049232251	0.110103376
ENSG00000137414	FAM8A1	0.27	0.049236214	0.110103376
ENSG00000099822	HCN2	-0.81	0.049262857	0.110150649
ENSG00000230445	LRRC37A6P	1.58	0.049317536	0.11026059
ENSG00000108510	MED13	-0.19	0.049347536	0.110315337
ENSG00000021826	CPS1	0.23	0.049416719	0.110457654
ENSG00000091039	OSBPL8	0.17	0.049434049	0.110484052
ENSG00000167523	SPATA33	-0.34	0.049443346	0.11049249
ENSG00000162909	CAPN2	-0.13	0.049454781	0.110505702
ENSG00000156970	BUB1B	0.17	0.049470526	0.110528543
ENSG00000172466	ZNF24	0.18	0.049502125	0.110582781
ENSG00000117682	DHDDS	-0.21	0.049505853	0.110582781
ENSG00000153132	CLGN	-0.56	0.049556865	0.110684373
ENSG00000256628	ZBTB11-AS1	-0.50	0.049602563	0.110774075
ENSG00000223813	AC007255.1	1.22	0.049615433	0.110790455
ENSG00000117600	PLPPR4	1.13	0.049634279	0.110815667
ENSG00000157625	TAB3	0.30	0.049638539	0.110815667
ENSG00000121690	DEPDC7	0.44	0.049643337	0.110815667
ENSG00000074266	EED	0.24	0.049654056	0.110827232
ENSG00000271858	CYB561D2	1.32	0.049699354	0.110915967
ENSG00000143127	ITGA10	-1.50	0.049716995	0.11093219
ENSG00000152256	PDK1	-0.34	0.04971771	0.11093219

ENSG00000183826	BTBD9	0.34	0.049779116	0.111056819
ENSG00000231584	FAHD2CP	-0.52	0.049812283	0.111118427
ENSG00000163406	SLC15A2	-1.07	0.049858814	0.11120983
ENSG00000272054	AC007390.1	-1.23	0.049868646	0.111219364
ENSG00000069943	PIGB	0.37	0.049894809	0.111265313
ENSG00000183648	NDUFB1	0.24	0.049973029	0.111427326
